# Development of a set of community-informed Ebola messages for Sierra Leone

**DOI:** 10.1371/journal.pntd.0005742

**Published:** 2017-08-07

**Authors:** John Kinsman, Kars de Bruijne, Alpha M. Jalloh, Muriel Harris, Hussainatu Abdullah, Titus Boye-Thompson, Osman Sankoh, Abdul K. Jalloh, Heidi Jalloh-Vos

**Affiliations:** 1 Epidemiology and Global Health Unit, Department of Public Health and Clinical Medicine, Umeå University, Umeå, Sweden; 2 Centre for Health and Research Training, Sierra Leone (CHaRT-SL), Freetown, Sierra Leone; 3 School of Global Studies, Department of Geography, University of Sussex, Brighton, United Kingdom; 4 Medical Research Centre (MRC), 5 Frazier Davis Drive, Freetown, Sierra Leone; 5 School of Public Health and Information Sciences, University of Louisville, Louisville, Kentucky, United States America; 6 Independent social development consultant, Dakar, Senegal; 7 Biltfaden UK Ltd, Communications Consultancy, Bradford, United Kingdom; 8 INDEPTH Network, East Legon, Accra, Ghana; 9 Department of Mathematics and Statistics, Njala University, Njala, Sierra Leone; Santa Fe Institute, UNITED STATES

## Abstract

The West African Ebola epidemic of 2013–2016 was by far the largest outbreak of the disease on record. Sierra Leone suffered nearly half of the 28,646 reported cases. This paper presents a set of culturally contextualized Ebola messages that are based on the findings of qualitative interviews and focus group discussions conducted in 'hotspot' areas of rural Bombali District and urban Freetown in Sierra Leone, between January and March 2015. An iterative approach was taken in the message development process, whereby (i) data from formative research was subjected to thematic analysis to identify areas of community concern about Ebola and the national response; (ii) draft messages to address these concerns were produced; (iii) the messages were field tested; (iv) the messages were refined; and (v) a final set of messages on 14 topics was disseminated to relevant national and international stakeholders. Each message included details of its rationale, audience, dissemination channels, messengers, and associated operational issues that need to be taken into account. While developing the 14 messages, a set of recommendations emerged that could be adopted in future public health emergencies. These included the importance of embedding systematic, iterative qualitative research fully into the message development process; communication of the subsequent messages through a two-way dialogue with communities, using trusted messengers, and not only through a one-way, top-down communication process; provision of good, parallel operational services; and engagement with senior policy makers and managers as well as people in key operational positions to ensure national ownership of the messages, and to maximize the chance of their being utilised. The methodological approach that we used to develop our messages along with our suggested recommendations constitute a set of tools that could be incorporated into international and national public health emergency preparedness and response plans.

## Author summary

The work on which the paper is based was conducted in two Ebola ‘hotspots’ in Sierra Leone (urban Freetown and rural Bombali district) between January and April 2015. Numerous misperceptions about Ebola had developed in the community over the course of 2014, and it was becoming increasingly clear that the largely top-down messaging strategy in use was not as effective as it could have been. Our project aimed to actively include the community in the development of a set of actionable Ebola messages that responded directly to their needs and concerns. While developing our messages on such topics as ambulances, burial teams, and the use of chlorine, we derived a set of recommendations that could also be applicable to control efforts relating to other infectious disease outbreaks. These include the importance of listening to community concerns during an outbreak, communicating on a two-way basis using trusted messengers, and engaging with relevant officials throughout the project in order to maximise the likelihood of the findings being utilised. While our work is based empirically on one disease in one setting, the messaging principles that emerged out of it are to a large degree generic.

## Introduction

The West African Ebola outbreak of 2013–2016 was by far the largest outbreak of the disease on record, with a total of 28,646 reported cases and 11,323 deaths [1]. Sierra Leone suffered nearly half of all the reported cases (14,124, of whom 3,956 died); while Liberia and Guinea had 10,675 and 3,811 cases respectively [1]. In addition to the incalculable suffering brought about by Ebola itself, severe disruptions to the affected countries’ already weak health systems undermined maternal and reproductive health services, malaria and diarrhoea treatment services, as well as vaccination programmes, which in turn resulted in increased mortality and morbidity in all these spheres [2].

The huge scale of the epidemic was driven in large part by the long delay in mounting an effective response, both by the international community and by the affected countries themselves, which in turn resulted in inadequate diagnostic capabilities and consequent delays in disease detection [3]. Eight months passed between the first case in December 2013 [4] and the announcement by WHO in August 2014 that the epidemic constituted a Public Health Emergency of International Concern [5]. By then the epidemic was already well established in several major urban centres [6, 7]. The poor living conditions and complex social environments to be found in many parts of the country greatly increased the difficulties in bringing the outbreak under control, even when significant resources finally became available [8].

Some of the responses during the early months of the epidemic had had unanticipated consequences, and collectively these had fuelled its spread. In the area of public health messaging, for example, there was initially a persistent and widespread denial of the reality of Ebola in Sierra Leone, with claims that the disease was in fact a government conspiracy [9, 10] aimed at ′killing civilians′ [11, 12] and ′making money′ [13]. A variant that blamed Ebola on western governments was also popular [14]. The messages that sought to counter these perceptions emphasised that the disease existed, that it was deadly, and that there was no vaccine, treatment, or cure [15]. While these messages were intended to promote acceptance of the reality of Ebola and concomitant preventive behaviours, they inadvertently reduced faith in the health system’s capacity to successfully treat Ebola patients. After all, if there was no vaccine, treatment, or cure, why should patients go to formal Ebola treatment centres? Thus these messages unintentionally promoted home-based care for people who fell sick with Ebola [15], which contributed to many of the hundreds of new cases per week being treated at home by family members, without adequate protection, and which in turn led to many new infections [15, 16]. The funerals of those who died at home also constituted a major source of infection. WHO estimated in November 2014 that 80% of all Ebola cases in Sierra Leone were linked to traditional burials and funeral practices [15].

Once WHO declared Ebola to be a Public Health Emergency of International Concern and the pace of the response began to pick up, there was an increasing recognition that local knowledge and full engagement with the affected communities needed to be at the heart of Ebola control efforts [13, 16, 17, 18]. This recognition specifically included the process of producing and providing Ebola messages as a means of avoiding the costly messaging mistakes that had been made early in the epidemic.

This is the context for the present paper, which describes a project conducted in Sierra Leone in early 2015 that was designed to receive direct community input into the development of messages aimed at preventing the spread of Ebola and promoting Ebola treatment-seeking behaviours. The work was conducted in collaboration with relevant national and international institutions, on which basis the messaging strategy aimed at being relevant, actionable, and acceptable to all the key stakeholders. We present the methods by which we developed the messages, the format of the messages, and some of the main lessons learned in the process of developing them.

While the West African epidemic is now over, it is essential that the lessons learned from it are incorporated into the preparedness plans of both international agencies and national governments. This paper is intended to contribute to that process by presenting our key findings as a toolbox that can be used for supporting the development of evidence-based, culturally contextualized messages in future public health emergencies.

## Methods

### The call for proposals, and our consortium

Our consortium was formed in response to a call for proposals on 21 August 2014, from Research for Health in Humanitarian Crises (R2HC), “to produce robust research findings that could contribute to the effectiveness of the response to the current outbreak, and help to draw lessons for future outbreaks of Ebola and other communicable diseases” [19].

The consortium comprised three institutional members, all of which were involved in proposal development and data analysis. These were the Epidemiology and Global Health Unit, Umeå University, Sweden, which led the work (http://www.phmed.umu.se/english/units/epidemiology/?languageId=1); the Medical Research Centre (MRC), Sierra Leone, which ran all operational issues and the field work (http://www.mrc-sl.org/); and the Centre for Health and Research Training, Sierra Leone (CHaRT-SL) (http://www.chart-sl.org/), which provided analytical expertise. Collectively, the consortium had skills in health promotion, communications, tropical diseases, medical anthropology, Sierra Leonean culture and history, and political science.

### Ethics statement

Ethical clearance was provided for the study by the Sierra Leone Ethics and Scientific Review Committee on November 21 2014.

### Development of the messages

**Fig 1:**
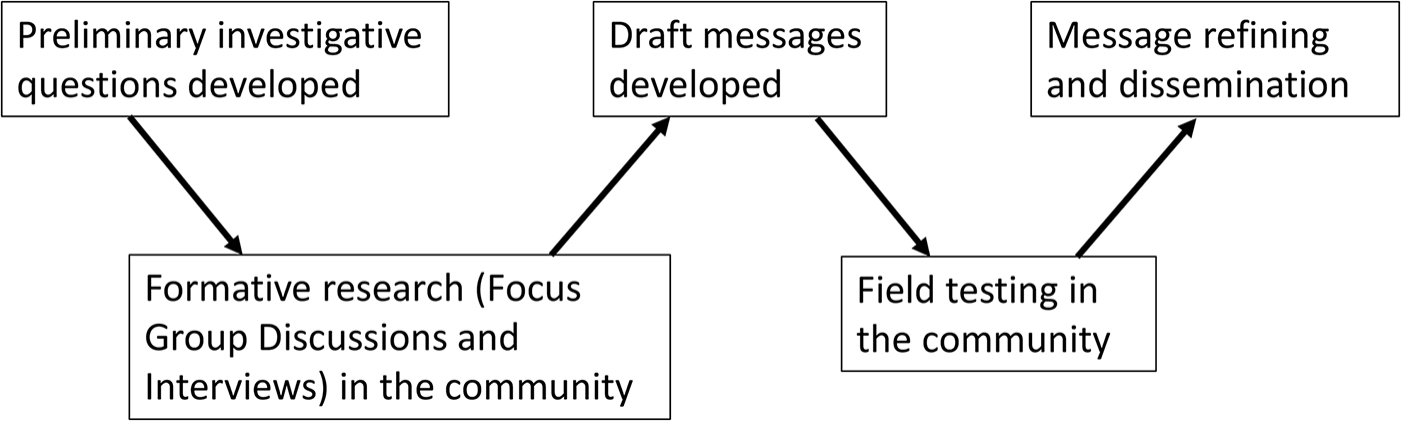
Schematic representation of the study design.

As indicated above, communication about Ebola to the Sierra Leonean population during the early phases of the epidemic had been conducted on a top-down basis, and there had emerged a recognised need to take into account the concerns, views and experiences of ordinary people in order to ensure that the messages being disseminated were both relevant and understood as intended. On this basis, our 3-month message development project took an iterative approach, as illustrated in Fig 1: the top row represents the study team and the national and international level stakeholders, while the bottom row represents the community. The arrows linking the five boxes show how the process moved between the levels, whereb*y* preliminary investigations were conducted into community perceptions of Ebola, Ebola messages, and the Ebola response. The findings from this formative research informed our development of draft messages aimed at promoting Ebola treatment-seeking behaviour. These draft messages were then field-tested, refined, and disseminated. The rest of this section presents this process in more detail.

The project was formally launched during a meeting in Freetown on January 17, 2015, attended by consortium members as well as representatives from a number of national and international organisations, including the Ministry of Health and Sanitation (MoHS) and the National Ebola Response Centre (NERC). The meeting was intended to raise awareness of the project among key stakeholders, and to seek their input into the process.

**Fig 2:**
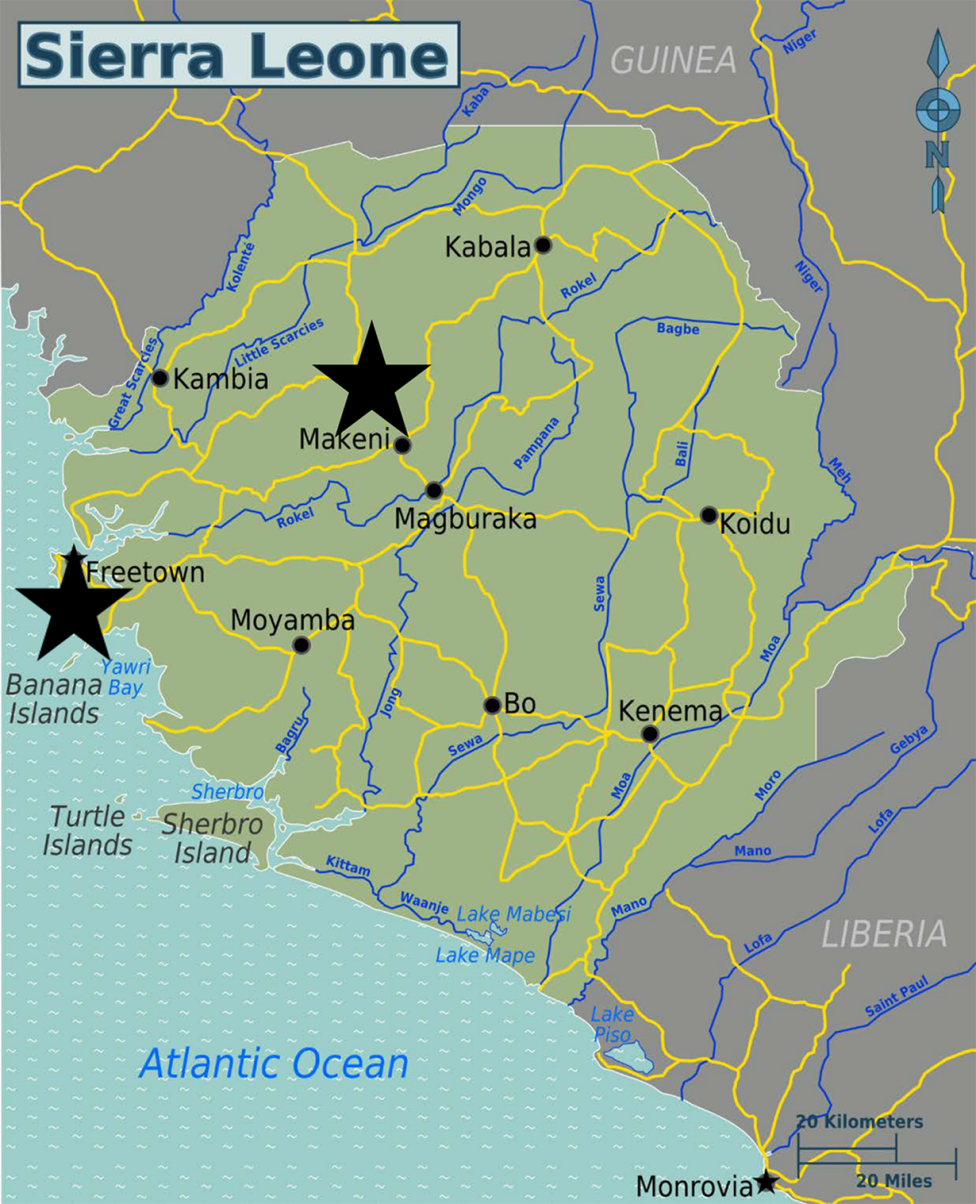
Map of Sierra Leone with stars indicating the two study areas. [Source: World of Maps, licensed under the Creative Commons Attribution-Share Alike 3.0 license].

Seven MRC research assistants and transcribers, all experienced in qualitative research, were then trained, with sessions on the study methodology, safety and ethical issues (especially consent and confidentiality), and data security. Formative field work was undertaken during late January and February, in two study areas: urban Freetown, and rural Bombali (see Fig 2). Both urban and rural areas were included because we wanted to understand the different opportunities and challenges that these contrasting settings can present for messaging. The specific study sites were chosen because they were currently experiencing or had recently experienced active Ebola transmission.

The formative research phase included 16 Focus Group Discussions (FGDs) comprised of people without any sort of leadership position in their respective communities. The FGDs were stratified by age (18±25 years, and >25 years) and sex, and were attended by 118 people (60 male, 58 female; mean = 7.4 participants per FGD). We also held a total of 24 individual, in-depth interviews with community leaders of various sorts (12 in each study area; 13 male, 11 female). These included religious leaders, traditional leaders, traditional healers, women’s and youth leaders, and medical staff engaged in the Ebola response. The reason for interviewing the latter group one-on-one was primarily for logistical reasons: such people are often quite time-constrained, so it can be challenging to bring several of them together in one place and at one time to take part in an FGD. However, this approach also permitted us to seek their individual professional perspectives and experiences, while the FGDs provided us with insights into the norms and social processes encountered in the wider community [20]. The educational level of our respondents was higher than the national average, in part because of our inclusion of the leaders and health workers: 17% had no education (as compared to 49% nationally [21]), 14% had at least some primary education, 38% had at least some secondary education, and 31% had at least some tertiary education.

The recruitment process for both FGDs and interviews followed a similar pattern in each participating community. First, we introduced the study to the respective village chief, who then called a meeting with key stakeholders, including traditional leaders, imams, pastors, women's leaders, youth leaders, health personnel, and teachers. During this meeting, our team explained the reason for the research, and that we wanted participants for both the FGDs and the interviews to include a mix of tribes, families, different geographical areas of the village, and people with different socioeconomic status and occupations. The key stakeholders then identified a list of possible participants, who we subsequently visited, accompanied by someone from the community to show us where they lived and to provide us with an introduction. We engaged individually with each suggested participant and confirmed that they met the selection criteria: willing and able to talk Krio, to talk about Ebola, and to take part. (A participant’s ability to communicate in Krio was necessary as it was spoken by all our interviewers, while not all of them spoke all of the other vernacular languages in the study areas. Some well-informed people who could not speak Krio may therefore have been excluded, but since as many as 95% of the Sierra Leonean population know the language [22], it is unlikely that this would have introduced any significant bias.) This process continued until we had the full quota for that community. The participant categories are presented in Table 1.

**Table 1:** Participant categories in the formative research.

**In-depth interviews**	Urban Freetown	Rural Bombali
Imam/pastor, Traditional community leader, youth leader, women’s group	5	5
Medical staff, including in hospitals and Primary Health Units; Community Health Workers; Health Management Committee	5	5
Traditional healers	2	2
**FGDs (6–8 ‘ordinary people’ in each)**	Urban Freetown	Rural Bombali
Male, <25	2	2
Male, 25+	2	2
Female, <25	2	2
Female, 25+	2	2

Questions in the FGDs and interviews were concerned with awareness of Ebola itself, Ebola messaging, and–based on knowledge from our in-country team members–issues to do with the Ebola response, such as treatment, ambulances and burial teams. Discussions and interviews were conducted primarily in Krio, but some discussions also included Mende, Tembe, Limba, or Loko. The data were recorded digitally, and transcribed directly into English; data quality was ensured by comparing audio recordings with the English language transcriptions, and correcting all transcription anomalies. Participants took part on a voluntary basis, having first provided written informed consent. As a token of gratitude for their contribution to the research, all participants received two bars of soap. Although some participants told of distressing experiences or stories, none were so upsetting that referral for counselling was deemed necessary.

A message development workshop was held in Freetown in mid-March, attended by eight consortium team members, the seven research assistants and their field supervisor, as well as representatives from the MoHS, the US Centers for Disease Control, and local NGOs such as Focus 1000. Four team members had been assigned to closely review all the transcripts prior to the meeting. Following the established principles of thematic analysis [23], these team members independently generated a list of recurring themes that emerged from the data. These were supplementary to the themes that had informed our questions as a starting point (awareness of Ebola itself, Ebola messaging, and issues to do with the Ebola response, such as treatment, ambulances and burial teams).

At the start of the workshop we collectively synthesised the four individual sets of themes into a single set of core themes for use in the message development process. These included issues such as trust and distrust, bribery and corruption, misperceptions about people being killed within the health system, and ‘seeing is believing’ (i.e. that people who had initially denied that Ebola was real changed their minds when they saw survivors). After reflecting on some basic health promotion and communications concepts (for example, gain- vs loss-framed messaging [24], cultural competence [25], and audience segmentation [26]), we then worked over five days in two teams of around eight people, each focusing on a different group of the core themes. Through this, a total of 26 draft messages was produced, each with an accompanying rationale, audience, dissemination channel/s, messengers, and list of associated operational issues. We define ‘Channel’ here as the means of distributing a given message, such as a poster, leaflet, radio jingle, or house-to-house meetings etc.; while the ‘Messenger’ is either an individual such as a traditional leader or an Ebola survivor who is depicted in or who articulates the message, or an institution such as the Ministry of Health and Sanitation which is seen to be responsible for it. Both English and Krio versions of the messages were produced, and two artists provided sketches for some of the main messages.

During late March and early April 2015, the 26 draft messages were field-tested in a series of eight FGDs in the same communities where the formative research had been conducted (four FGDs in each of the two study districts, with a total of 32 male and 32 female participants–these FGDs were not age-stratified). All of these people had participated in the initial FGDs, so they were already familiar with the project and its objectives. Each message was discussed in two different FGDs: we sought the perspectives of both women and men as well as both urban and rural participants on each message. For example, one message might be reviewed by rural men and urban women, while another could be discussed by rural women and urban men. In order to keep the discussions focused, each FGD covered no more than five different message topics.

Field testing sought to assess understanding, acceptability, perceived likely effectiveness of the messages, as well as appropriate distribution channels and messengers. Although the process identified a number of issues in the messages, all of them were seen as broadly acceptable and none were removed altogether. With this feedback, we reworked the messages into 14 topic areas and then formally presented the final document to the MoHS at a multi-stakeholder meeting, on April 15, 2015.

## Results

This section is presented in four sub-sections: (i) a summary of some major principles of message development that emerged through the data, and that we took into account in our work; (ii) an explanation of the different components of the messages, and of how these fitted together; (iii) an example of one of the messages that we developed as an illustration of our output; and (iv) the dissemination process, which highlighted some important lessons for future public health emergencies.

### Principles of message development

Four cross-cutting principles emerged through the message development process, which were of relevance for all the topics addressed.

#### Trusted messengers

There were indications from some respondents in both the formative research and the field-testing that people wanted to hear messages from people they trust: they argued that the value of the message is enhanced if the messenger is trusted. As one man explained: “If you trust that person who carries the message, any message he or she is giving you, you can rely on it and believe in it” [FGD Urban Males >25].

Certain categories of people were seen as inherently trustworthy, and therefore good as messengers, while others were seen as inherently untrustworthy, and they were therefore inappropriate for use as messengers. For example, one person’s opinion was that: “The Chiefs, Imams, and Pastors are very important to talk to the people in the community. But a lot do not trust the medical people” [FGD Rural Males, mixed age]. This lack of trust in medical workers was quite widely expressed by our respondents–as one woman said: “Truly speaking, this is from inside my heart, when they carry people [to the hospital], they kill them there” [FGD Urban Female >25]—and it may have been linked to reports of poor care in the health services, both prior to and during the Ebola epidemic. However, as with all categories of putative messengers, the views about their trustworthiness were not universal: some people trusted the medical workers while others did not; equally, some trusted the Chiefs, while others did not. This has important but complex implications when considering who should act as the messenger for a given message.

#### Two-way communication

Connected to the previous point, our respondents made clear that in addition to including one-way messaging strategies such as radio discussions, jingles, and billboards, they also wanted the messages to be disseminated through community meetings, house-to-house visits, and other such face-to-face dissemination strategies: “The best is when they hold it [the poster or leaflet] in their hands and move door to door, explaining to the people. But just looking at the picture, it will not be properly understood” [FGD Urban Females, mixed age]. Not only do such two-way approaches provide the possibility for a dialogue about the issue with someone who hopefully is perceived as being trustworthy, but there are also strong practical justifications for such strategies: radio discussions may be missed by people who have no access to a radio, especially in the rural areas; and the country’s low literacy rate (36% for women and 54% for men [27]) means that text-based posters and billboards will simply be missed by a majority of the population. An additional potential asset of two-way communication is that it can provide a means for the ‘messenger’ to act as a channel between the population and those involved operationally in managing or delivering the response. By informing the people at operational level about any community concerns, efforts can then be made to address perceived or actual shortcomings in the response.

#### Parallel operational services

Messages to promote treatment-seeking behaviour must be matched by good services, otherwise the credibility of those messages will be undermined. For example, our respondents reported frustration at poor ambulance and hospital services, and disrespectful burial teams, perceptions which could make people reluctant to call upon these services even if there were messages encouraging them to do so. As one respondent said during field testing, “Please, you have to train the burial team to be more professional, because if you send this message and the burial team takes bribes, then the message will be meaningless” [FGD Urban Males, mixed age]; while another said, “Well, for people to accept this message, government should improve on all the services in the hospital” [FGD Urban Males, mixed age]. Thus, ensuring a good quality of any parallel operational services should be an integral component of any messaging strategy in an outbreak situation.

#### Local factors

Two locally salient issues emerged, the first of which concerned messaging colours. Our respondents stressed during field testing that posters and leaflets should not be coloured predominantly in either red or green, as these are the colours of the government and main opposition parties respectively. If a given message was associated in people’s minds with any particular political party, it may undermine the validity of that message in the minds of people who support another party. Respondents voiced a preference instead for posters and leaflets to be either in other bright colours, or in black and white: “For me I prefer black and white, no political party colour… not red and green” [FGD Urban Males 18–25].

The second local issue concerned language. We provided both English and Krio versions of all the messages in our final messaging document. As indicated above, many people cannot understand, let alone read English. As one respondent explained to us (in Krio), “Me, I want everything to be in Krio” [FGD Rural Males >25]; while another said, “People will understand the Krio, instead of big English” [FGD Rural Females, mixed age]. Others stated a preference for the messages to be in Mende or Temne.

These specific local issues would not necessarily apply in other settings facing a public health emergency, but other local issues would likely be relevant, and these should be identified and taken into account in the message development process.

### The different components of the messages

The final report that we presented to the MoHS and to other national and international level actors includes full details of all the 14 messages that we developed, and this is included as Supplementary Material to the article. This section provides details of the standardised format in which we presented the messages. The format included six components: (i) the rationale for the message; (ii) the audience to whom the message was directed; (iii) the channel/s through which the message would be disseminated; (iv) the messenger/s; (v) the message content; and (vi) associated operational issues that would need to be taken into account. This generic format would be relevant for message development in any future public health emergency.

#### Message rationale

As a starting point, it was important to provide a rationale for why each message was necessary. To illustrate this, we heard many complaints in the formative research about perceived disrespectful behaviour of burial teams towards the dead, which could lead to families deciding to conduct unsafe burials at home. As one person said, “Even when they put them in the body bags, if you don’t want to cry you will cry, because when a person has died, though they are gone, the person should receive respect. But their way of handling is like throwing the dog in a dust bin, so actually, I don’t feel happy” [FGD Rural Males, >25]. Such comments reportedly referred to incidents early in the epidemic, since when protocols had been improved; but our data indicated that doubts still remained in some people’s minds. Thus there was a clear rationale for developing a message to reduce unsafe, family-led burials by promoting officially designated burial teams.

#### The audience

The desired content of most of the messages, as expressed by our respondents, was more or less generic for (a) women and men, and (b) for urban and rural dwellers: in most cases, there was no real need to provide different messages for different target populations. However, some messages were intended for urban audiences (such as those that sought to dissuade people from making prank calls to the ‘117’ Ebola hotline, which we learned during the formative research was primarily an urban phenomenon); some targeted women, as the primary care givers; and some focused on older people, who were chiefly responsible for conducting illegal burials in the community. Traditional, religious, cultural, youth, and women’s leaders were also targeted in some cases.

#### The channels

We recommended using two different types of mutually reinforcing channels for each message: one-way channels that acted as straightforward means of providing information, and two-way channels that facilitated active dialogue. Some channels were contingent on locality. For example, social media were seen as an attractive channel for reaching out to urban youth, who had much greater access than their rural peers to digital technologies. Table 2 presents the different channels we recommended.

**Table 2:** One-way and two-way channels for message dissemination.

One-way channels	Two-way channels
Posters and leaflets	Community meetings
Plastic bags with a message printed on	Face to face discussions with community/peer mobilisers, supported by posters/leaflets
Radio discussions involving pastors, imams, and/or youth and community leaders	Participatory theatre drama in the community followed by questions and answers session
Jingles disseminated via the radio or mobile PA systems	Radio and TV call-ins
Radio drama	
Sermons in church/mosque	
Wristbands with a message	
Text messaging	
Photo album showing, for example, what happens in the Ebola Treatment Unit, or in the ambulance etc.	
Social media such as Facebook, What’s app, etc. [can also be used as two-way channels]	

#### The messengers

During field testing, one informant stated the following: “I want to suggest, in passing on this message, let the religious leaders pass it on to their congregational members, let the youth leaders target the youths, the old target the old, teachers and lecturers pass on it on to their students. With this, it will be nice and the message will pass on to everyone” [FGD Urban Males, mixed age]. This highlights the fact, as discussed above, that no single messenger will reach all members of the community: complementary messengers are needed for each setting. On this basis, we applied the principle of audience segmentation to the messengers, with recommendations for different messengers to disseminate the messages to specific audiences for each setting and for each message.

If the messenger was an individual, they would likely be speaking, face to face or on the radio, or they would be depicted visually. Possible categories of individual messengers included, for example, Ebola survivors; people involved in the Ebola response (i.e. health care workers, burial team members, ambulance staff etc.); religious, youth, traditional, women’s, and other community leaders; music stars; and football stars. If the messenger was an institution, such as the Ministry of Health and Sanitation, we felt it important that they should be represented on the basis of a logo on a poster or leaflet, or some other official endorsement of the message.

Gender was seen as important only in relation to certain specific topics, such as care-giving, which falls largely within the domain of women; and as such, messages to do with care-giving should feature a female messenger [28]. For topics that do not have a clear gender component, such as stigma, we found that it would be important to feature both female and male messengers.

#### Message content

The messages addressing the 14 topics that emerged through the data were intended to be used either verbatim in any one-way communications, or as talking points in two-way dialogues. The topics and their accompanying messages (each of which was presented in both English and Krio) are context-specific in contrast to the more universal messages that WHO has produced on, for example, Ebola signs and symptoms, and the basic facts about transmission [29]. They are summarised below:

1. Burial teams and respect. There was concern about disrespectful treatment of both Ebola corpses and the bereaved families by official burial teams. The messages were “We treat corpses with respect” and “Let us work together to ensure safe burial”; and they were accompanied by drawings of a Christian and a Moslem burial and a respectful burial team.
2. Burial teams and bribery. Family members had been offering money to burial teams, and burial teams had been asking for money, in contravention of the bylaws. The message was “Safe burial is free; Do not pay or receive money for safe burial”; and this was accompanied by a picture of a bribe being handed over at a burial, covered by a large red cross.
3. Fear of ambulances. Many people complained about different aspects of the ambulance service, including over-speeding, drunk driving, disrespectful drivers, lack of fresh air in the vehicle, and excessive use of chlorine while in transit. The message was “The ambulance is the best and safest way to go to the hospital to receive treatment. The ambulance is well ventilated so that the patient will not have trouble breathing”, and this was accompanied by a picture depicting good airflow in the ambulance.
4. Misperception: killing of patients. Misperceptions emerged during the formative research about the killing of patients by the health system. The message was provided through a photo album with pictures of the process from falling sick to either being confirmed as an Ebola patient or not. Photos included the ambulance interior; Smiling medics; Blood tests and/or swab; Holding centre (inside and outside); Ebola Treatment Centre (inside and outside); Lying in bed with drips, water/ORS and/or tablets; Food; Phone conversation with family members; Going home. The photo album embodied the principle of ‘seeing is believing’ that our respondents described, whereby people were sceptical about many aspects of Ebola and the Ebola response until they saw convincing evidence to the contrary with their own eyes.
5. Distrust of health system. Related to (4) above, there were beliefs about a generalised mistreatment of patients within the health system. The messages were “Go to the hospital when you’re sick. You’ll be well looked after”; and “Go in the ambulance when you’re sick. You’ll be well looked after.”
6. Fear of chlorine. See the sub-section below, entitled ‘An example of one of the messages: Mr Chlorine’, for full details of this message.
7. Stigma against survivors. In spite of considerable efforts to reduce stigma against Ebola survivors, it was clear that stigma persisted in some communities. The messages were “Welcome our brothers and sisters who have survived Ebola back into their homes, jobs and communities”; “Do not laugh at or avoid Ebola survivors”; and “Don’t Spread gossip about Ebola survivors”.
8. Stigma against Ebola workers. This message aimed to address the stigma against people working in the Ebola response (health care workers, burial team members, ambulance staff) by pointing out that Ebola response workers are heroes, and they should be embraced, not stigmatised. Note that we used the word ‘hero’ here on the basis that Ebola workers made an active choice to contribute by doing very dangerous work. Not all survivors would have been heroes in the same sense, since many did not become infected when performing their duties (professional or personal), fully aware of the potential danger. The messages were “Our Ebola workers are our heroes!”; and, accompanying a poster of a nurse, “Nurse Susan is a hero, who helped many Ebola patients survive”.
9. Get early treatment. For many reasons–including distance to health facilities and perceived quality of care–people did not always go for early treatment, and this reduced their own chances of survival while also risking the infection spreading to their families. The message was based on a picture of a survivor with a certificate saying: “If you go to hospital early, you improve your chances of recovery, like I did.”
10. Call 117. One of the challenges mentioned by our informants was the early, non-specific symptoms of Ebola, which can be similar to those of, for example, malaria, which in turn can make it difficult to know how to respond in terms of seeking treatment. This message aimed to encourage people to call 117, the widely known Ebola hotline, as a first step towards receiving proper medical attention. The message, as a jingle, was “Call 117 for any illness you have.”
11. Caring for the sick while waiting for ambulance. This was a prevention message aimed at helping people to care safely for those who are sick at home after they have called 117. The message provides an actionable set of steps for keeping a household safe, including designating one dedicated carer for the patient, and simple instructions for barrier nursing as well as safe disposal of soiled materials.
12. Staying safe while waiting for burial team. This message provided step-by-step practical guidance to families when somebody dies at home, including advice not to touch the body and to call for the burial team.
13. Ebola denial. Our formative research indicated that direct experience of Ebola convinced many people who were initially doubtful about the existence of the disease that it is in fact real. We wanted to make use of these people’s experiences to convince other doubters of the reality of Ebola. Using survivors or religious/traditional leaders who previously hadn’t believed in Ebola as messengers, the message was “You don’t have to see someone with Ebola to believe that it is real”.
14. 117 prank calls. Up to 85% of all calls to the 117 Ebola hotline were defined as ‘prank calls’. Our respondents recognised that such calls slow down the Ebola response, and they suggested that people will be more sympathetic towards 117 if they knew the extent of this problem. The message was “Be a good citizen–don’t make fake calls to 117, it disrupts the Ebola response.” Note that this message was not intended in any way to undermine Message 10 (“Call 117 for any illness you have”), which was specific about the need to call if someone had any symptoms. The point here was to highlight the importance of not overloading the 117 switchboard with unnecessary calls.

#### Operational issues and associated risks

As indicated above, parallel operational issues need to be considered in relation to any messages being disseminated. These concern both the infrastructure related to the topic in question (e.g. burial teams, ambulances etc.) as well as the messengers and channels through which the message will be disseminated. Ensuring that these operational issues are taken into account upholds the credibility of the message/s in question as well as other messages from the same source, while also maintaining community confidence in the wider Ebola response. Table 3 gives examples of operational requirements and associated risks for message themes, channels, and messengers.

**Table 3:** Operational requirements and associated risks for message themes, channels, and messengers.

Message theme, channel, or messenger	Operational requirements	Risk of operational failure
**Message**: Promoting trust in burial teams	Burial teams must be trained and supervised regarding how to behave to bereaved families and/or communities, and how to perform a respectful burial	If good operational standards are not met, communities will not be willing to participate in safe burials, and the risk of secret burials will continue.
**Channel**: Drama shows	Professionals need to be available to train drama groups how to do community-based theatre	If training is inadequate, no drama shows will be conducted, or those that are conducted may not carry the desired message
**Messenger**: Community leader	Messengers must be known to and trusted by the community, as well as trained and knowledgeable on the topic.	Unscreened and/or untrained messengers may end up perpetuating misperceptions to do with Ebola and the Ebola response, thereby undermining community confidence and trust

### An example of one of the messages: Mr Chlorine

One of our messages, concerning the use of chlorine when handling patients with Ebola, is presented here in full detail to illustrate the standardised, six-component format used.

#### Rationale

Fear of chlorine in ambulances and by burial teams arose as a major issue in the formative research: ironically, an intervention designed to protect people was perceived by some as being part of the problem. Several informants indicated that they believed the chlorine used to disinfect the ambulances was killing patients in transit to treatment centres, which would clearly act as a significant disincentive to go for treatment if it was necessary. The rationale for this message was therefore to address these concerns by presenting chlorine as a friendly and safe support in the fight against Ebola.

#### Audience

General public

#### Channels

Both one-way and two-way channels are included, thereby providing mutual reinforcement. One-way channels included posters distributed by Non-Governmental Organisations, Community-Based Organisations, District Health Management Teams, and Public Health Units, using local youth groups, chiefdom councillors, and social mobilisers. They were to be posted at community gathering points, ataya bases (makeshift cafés/meeting places for young people), court barries (local open village courts presided over by a chief), pharmacies, parks/bus stops, and inside/on public transport. Two-way channels included leaflets, combined with community house-to-house visits/meetings by community members.

#### Messenger

Mr Chlorine is a cartoon character, and he is the individual messenger. The institutional messengers would be whoever is responsible for disseminating it (e.g. the MoHS and partners), who should make their logos visible so that the audience knows the source.

#### Message

**Fig 3:**
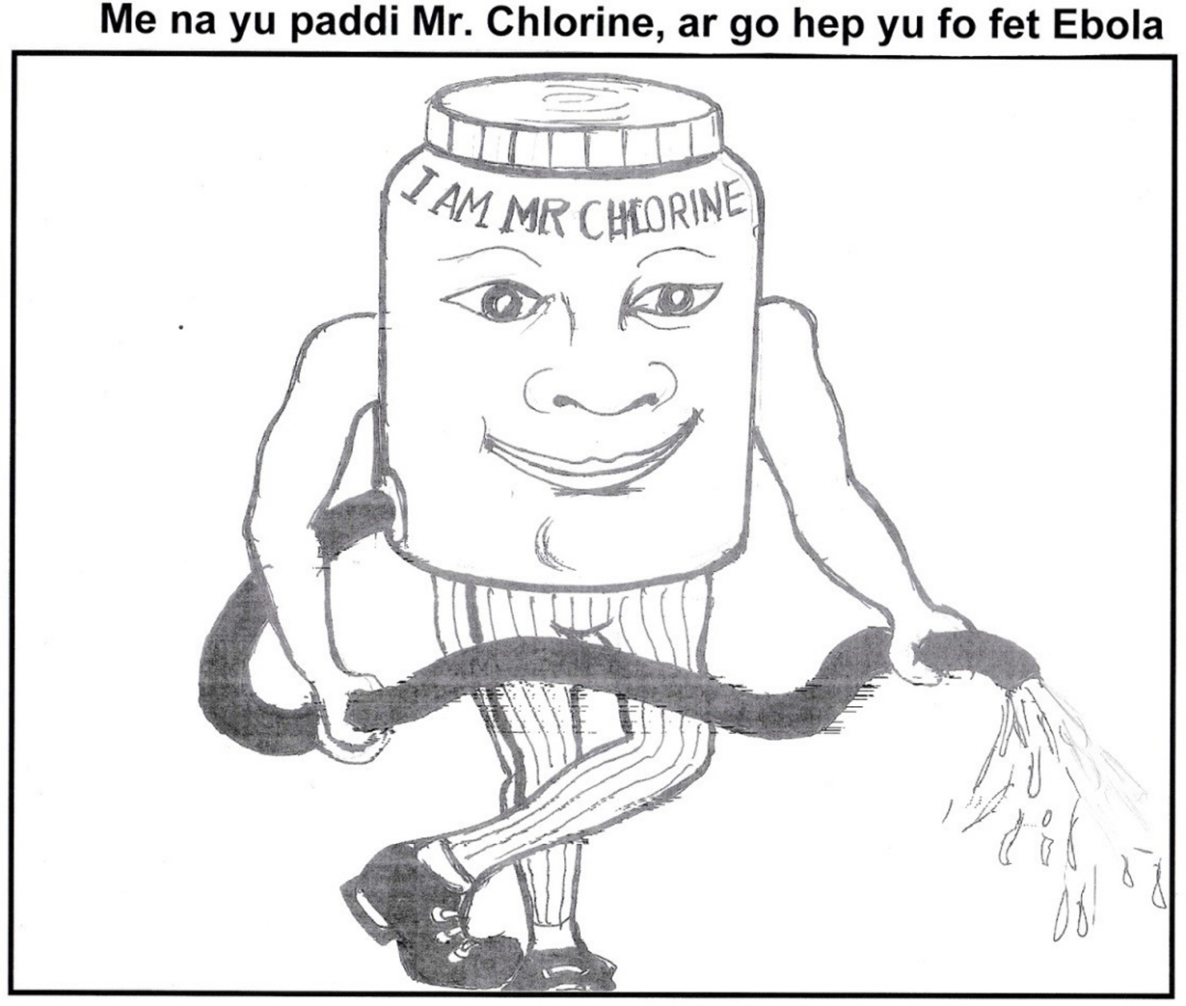
‘Mr Chlorine’ promoting chlorine as an important support in the fight against Ebola.

Cartoon character with friendly character, ‘Mr Chlorine’. The Mr Chlorine image was produced by one of our project artists–see Fig 3 –with the accompanying text: “I am Mr. Chlorine, your friend in the fight against Ebola”. / “Mi na yu paddi Mr. Chlorine, ar go hep yu fo fet Ebola”

#### Operational issues

Ambulance and burial teams should use chlorine in the right way and with the right dose; and ventilation should be provided inside ambulances, with teams waiting for some time after spraying before people board the ambulance.

### The dissemination process

This project was conducted with the full knowledge and support of both the MoHS and the National Ebola Response Centre (NERC). Key actors from both institutions, including the Deputy Minister of Health and the NERC Operations Manager, contributed to the workshops, and we also met with them and other senior colleagues individually over the course of the project to keep them appraised and to receive their input. We hoped that the chances of our messages being used would be maximised through this process.

Two progress updates were given at the NERC Daily Briefing, in January [30] and March [31]. After the second briefing, the NERC and the in-country UN Mission for Ebola Emergency Response (UNMEER) leadership requested that we fast-track the development of our messages so that they could be utilised during a nationwide lockdown the following week. Their interest in our messages was specifically on the basis of their being empirically derived, which was apparently unique in the country at that stage in the epidemic. During the lockdown, which was aimed at curbing the spread of Ebola, the entire population of the country was ordered to stay at home while volunteers went house-to-house looking for Ebola cases and conducting awareness-raising activities [32]. We duly submitted our draft messages, but they were not in fact used. We learned later that one of the key individuals responsible for collating the messages for the lockdown had not been aware of our contribution.

The final messaging document was presented at a meeting at the MoHS in mid-April, attended by all the main government, UN, and NGO Ebola actors in the country. The work appeared to be well received, and adapted versions of our messages on ambulances, chlorine, calling 117, and staying safe while waiting for the ambulance or burial team were subsequently incorporated into the Social Mobilisation Action Consortium’s Consolidated Message Guide for Ebola Communication. This was an official, regularly updated document whose purpose was “to provide a reference of accurate, standardised information in simple language and key message format” [33]; but it gave no details of audience, channels, messengers, or operational issues.

Although we do not know whether or the extent to which any of the messages were actually put into use (we did not have the resources to conduct an impact evaluation of the work), we did hear anecdotally that a poster based on one of our images on respectful burial had been seen upcountry. For our informant, the poster was appealing both because of the depiction of people “like us”, but also because the obviously local production of the poster was seen as attractive. Based on this, it seems possible that more of our messages may have found their way into use, perhaps through the aforementioned Consolidated Message Guide for Ebola Communication.

## Discussion

This project developed a comprehensive Ebola messaging strategy in Sierra Leone, based on a substantial qualitative database collected in two areas of the country where Ebola infections were ongoing. As such, it explicitly aimed to address concerns raised by the community about Ebola itself and about different aspects of the Ebola response. Our strategy also entailed close networking and engagement with key national level stakeholders as a means of raising awareness of the work, receiving relevant input, and thereby enhancing the likelihood of the product being adopted into practice [34].

The overall methodological approach for our project was not conceptually complex, but we discovered that this sort of iterative, qualitative message development for Ebola had not, until we presented it, been attempted in the country; and it is this that appealed to the leadership of the national Ebola response. Our work had identified several issues of real community concern about the Ebola response, that had not yet been included in the country’s official messaging database [33]. A series of national Knowledge, Attitude and Practice surveys had previously been conducted [35], but although these generated an important source of information, the surveys were by definition based on pre-defined questions, and they did not give people the opportunity to raise their own concerns.

This points to one of the major lessons learned from both our project and from others engaged in the West African Ebola response: the fact that communication with affected communities should be conducted on a two-way basis, not–as it had been early on in the epidemic–only in a top-down, “authoritarian” fashion [8, 17]. This applies both to the message development process itself and to message dissemination. The process of “meeting people where they are” [18] allows those who are developing the Ebola messages to understand the challenges faced by the community, while also providing a means for them to learn of rumours or myths that may be circulating. This in turn affords the opportunity to counter or dispel such misunderstandings [18].

Further, two-way communication can provide an understanding of the complex but very real challenges that people encounter when faced with, for example, a sick family member at home, or the corpse of an Ebola victim. The messages that can emerge from a two-way process are more likely to be practical, relevant, and actionable, and less likely to focus on the potentially less effective ‘low-hanging fruit’ of health communications, such as those based on slogans like ‘Ebola is real’ or ‘Stop Ebola’ [36]. In addition, although two-way communication of this nature requires more capacity and organisation than top-down mass media approaches, it allows for direct community sensitisation, which has the capacity to increase people’s ability to adhere to prevention practices, while also offering the possibility to develop and maintain trust in the messengers [37]. Looking in the other direction, it also provides the possibility for the messengers to inform those responsible for operational decisions–concerning, for example, ambulances, burial teams, or Ebola Treatment Centres–about any community concerns, and thereby for improving these essential services.

In sum, therefore, systematic, iterative qualitative research should be fully embedded into the message development process from the outset of the response to any future public health emergency, and the communication of those messages should also be conducted on a two-way basis.

One of the striking features of the Ebola epidemic in Sierra Leone was the fact that, especially early in the epidemic, lessons learned from messaging in previous viral haemorrhagic epidemics were not taken into account, and this undoubtedly contributed to prolonging the outbreak. Remarkable parallels can be seen in the responses to the Sierra Leone Ebola epidemic and an outbreak of Marburg Haemorrhagic Fever (MHF) in Angola in 2005, with 374 cases and 329 deaths [38]. Initial messaging in the Angolan MHF outbreak claimed–as in Sierra Leone–that “There is no cure for this disease,” which was reportedly understood by community members to mean that even if they accepted hospitalization, death would be inevitable [38]. Once again, as in Sierra Leone, many people therefore felt it would be preferable to die at home surrounded by loved ones as opposed to in an anonymous hospital setting, and this continued the cycle of infection and prolonged the outbreak. Such unanticipated and counter-productive responses to messaging have been observed in other health promotion campaigns–for example, child vaccination programmes [39] and warnings about the health effects of alcohol abuse [40]–so it is essential that message developers do not simply assume that the worst outcome from their efforts will be no effect at all.

Based on their experiences in Angola, Roddy et al. concluded that “An effective IEC program [‘Information, Education, and Communication’–a term used to describe messaging strategies] is crucial to the control of the outbreak and should be implemented from the start of the intervention… Actively involving all key stakeholders from the beginning is crucial” [38]. In spite of that clear recommendation, precisely the same mistake was made during the Ebola epidemic in Sierra Leone as had been made in Angola, even when the consequences could quite easily have been foreseen. This raises an important question concerning why lessons learned in public health emergencies are not taken on board and institutionalized for use in future situations: in many respects, it appears not to be a question of an overall lack of knowledge about what to do, but rather of ensuring that the right people have (a) access to the right knowledge and (b) the ability to operationalise it.

The tragic repetition of this mistake during the Ebola outbreak also highlights the imperative of ensuring that there will be no such failure to intervene in timely fashion in any future public health emergency, and that this intervention will be based on appropriate community engagement and messaging strategies. A significant challenge inherent in this, however, is that such activities take time to establish: our message development activities took three months to complete (in addition to the time needed to obtain ethical clearance before we could start), which is of course far too long to wait during an emergency. For future Ebola outbreaks, the universal messages from WHO on, for example, signs and symptoms, and modes of transmission [29] are instantly available; while the culturally contextualized materials from our study, combined with other messages that were produced during the West African outbreak, could be utilized as a starting point in the rapid production of an initial round of messages. These would need to be subjected to field-testing so as to ensure relevance and to minimise the risk of unanticipated consequences. Simultaneously, the more time-consuming approach detailed above could be utilised to develop a comprehensive and empirically-grounded set of messages. In all cases, however, messages should be field tested, as they were in our study: experience has shown that disseminating untested messages can turn out to be an expensive false economy.

We learned some important lessons about dissemination over the course of our project. We had made significant efforts to engage with key national level stakeholders, based on our connections through the MRC leadership to many senior individuals, both to receive their input and thereby enhance the relevance of in the work, but also to maximize the chances that the messages would actually be taken up when finalized [34]. However, in spite of our best efforts, we nonetheless faced significant challenges in ensuring that our messages were adopted and disseminated. We eventually established that the reason for the non-inclusion of our messages during the lockdown was because we did not know the one key individual who oversaw the messaging at that time, and that person had simply been unaware of our product. There were, by that stage, many local and international institutional actors engaged in Sierra Leone’s Ebola response, and this created a somewhat overcrowded ‘marketplace’ for initiatives, which inevitably made it more difficult to be seen, and to raise awareness of and interest in one’s work. But, perhaps more importantly, our mistake could have been our focus on interacting with the top echelons of the key institutions, as opposed to the operational people, or the ‘street level bureaucrats’ [41] who actually did the day-to-day work. Researchers in similar situations in the future may benefit from taking this point into account in their dissemination efforts.

### Study limitations

Several potential limitations of our work need to be acknowledged and discussed. First, the study was conducted in two parts of the country that had been affected by Ebola, and these cannot necessarily be seen collectively as representative of the country as a whole. Thus, while the messages may have had relevance for other settings in Sierra Leone, we do not know whether or not this was the case. That said, our study population was relatively well educated by comparison with the national average, and since even these people appreciated the visual messaging that we produced (as indicated during the field testing), this important non-literary component of our approach would therefore likely have had at least as much appeal to the wider population.

Second, while all our interviewers were Sierra Leoneans, trained and experienced in community-based qualitative field work, they were nonetheless ‘outsiders’ to the individuals they interviewed. Thus, there is the possibility that, especially under the highly stressful conditions that prevailed during the Ebola outbreak, there could have been some suspicion of them and their motives, or people may have had expectations from the research team that could not be met, or they may simply not have been willing to tell strangers what they really felt was happening. In any of these cases, some bias could have been introduced into the dataset. Similarly, our recruitment procedure necessarily involved the village chiefs, who provided permission for us to work in their chiefdoms while also connecting us to community leaders who then helped identify potential participants. Through this process, we may have been directed towards people with an agenda beyond simply answering our questions and sharing their experiences–although it is important to note that we never found any indication of this. In mitigation, the Medical Research Centre (which was operationally in charge of data collection) has a history of engagement in and around the communities where we conducted field work, including building (in 2010) and providing ongoing support for the new midwifery school in Makeni, the capital of Bombali District. This may have facilitated their being perceived by our participants as an ‘honest broker’ who could be trusted. Further, some participants made strong negative remarks about the chiefs, which indicates that they were not speaking on their behalf. On this basis, we believe that any potential bias in the dataset, while possible, would be minimal, and that the material on which we based our analysis can be accepted as broadly representing the actual views and experiences of the people we spoke with.

Third, we field-tested our messages with some of the same people who contributed to their development through participation in the initial formative research: they were already familiar with the project and its process, and, given our tight timeframe, we did not consider it viable to recruit another group of participants. We also hoped that their prior involvement would give them a sense of ownership of the messages, and an enthusiasm to help develop them further. Equally, however, this could have created a social obligation whereby participants felt that they were obliged to confirm that the messages were ‘good’. Thus it is possible that we could have ended up with a more positive view of the messages during field testing than would have been seen in the general population.

Finally, by the time we started our data collection, a fairly extensive health communication and social mobilisation process was already underway in the country, and the messages we produced need to be seen within this context. Indeed, we specifically asked participants about the Ebola messages that they had heard, seen, or read, as well as about their clarity, acceptability, the extent to which they could be understood, and the way they had been disseminated. Through this, we deliberately aimed to build on what had already been produced–pre-existing messages that were part of the contemporary social landscape–rather than trying to start completely afresh. Not all our message topics were therefore necessarily completely original, but, based on the participants’ comments, we sought to optimise the messengers and the channels through which they were disseminated.

### Conclusions

An understanding of community perceptions of and responses to a serious public health threat should lie at the core of attempts to inform the population, both about the disease itself and about control efforts. A message development process that actively includes the community must therefore be systematically included in the response to any serious outbreak, right from the start. This has been stated previously, but, given the extremely serious implications of the messaging failures during the early phases of the Sierra Leonean Ebola outbreak, this approach must now be formally institutionalized into international and national outbreak preparedness and response plans.

Our conceptually straightforward methodological approach to developing messages during a public health emergency, as well as the lessons we have learned during this process, constitute a toolbox that could be incorporated into such plans. The five major components of this toolbox include:

1. Institutionalisation of systematic, qualitative, community-based research early in the outbreak to determine community perceptions and concerns about the disease in question, as well as the response to it.
2. Development and field testing of a set of messages, based on these perceptions and concerns, which should include six components: (i) the rationale for each message; (ii) the audience to whom the message is directed; (iii) the channel/s through which the message would be disseminated; (iv) the messenger/s; (v) the message content; and (vi) associated operational issues that would need to be taken into account to make it effective.
3. Communication with the community and message dissemination should be conducted on a two-way basis, with the use of trusted messengers for each segment of the population.
4. Ensuring a good quality of parallel operational services should be an integral component of any messaging strategy in an outbreak situation, otherwise the credibility of current and future health promoting messages will be undermined.
5. Engagement with both the political and the operational leadership within the Ministry of Health. Senior policy makers and managers need to be involved to ensure national ownership and relevance of the messages, while the people in key operational positions need to be properly aware of the product, so that the messages may thereby be utilised.

## Supporting information

S1 Appendix Ebola messages, FGD and interview transcripts. (ZIP)

S2 Appendix Ebola messages for Sierra Leone, final report. (PDF)
