# Supplementary material for: Development of a set of community-informed Ebola messages for Sierra Leone
Source: PLoS Negl Trop Dis. 2017 Aug 7;11(8):e0005742. doi: 10.1371/journal.pntd.0005742 (PMC5560759; doi:10.1371/journal.pntd.0005742)
Supplement: S1 Appendix — (ZIP) [file pntd.0005742.s001.zip › Ebola messages - FGD and interview transcripts/R2HC Ebola Fieldwork 1/ICs and cover sheets R2HC Ebola first fieldwork Jan 2015 FINAL.docx]

# DRAFT Coversheet and informed consent form for community leaders

*Ask informed consent; fill in the personal data sheet, then start the interview.*

| CODE |  |
| --- | --- |
| DATE | …………………../………………………/………………………… (DD/MM/ YYYY) |
| TIME – start | (hh:mm) |
| TIME – end | (hh:mm) |
| DURATION (minutes) | (minutes) |
| Voice recorder number |  |
| NAME MODERATOR |  |
| NAME TRANSCRIBER |  |
| District / CHIEFDOM |  |
| COMMUNITY |  |
| LANGUAGE INTERVIEW |  |

**PERSONAL DATA RESPONDENT** (*circle answer)*

| Age *(in whole years)* | …………………..years |
| --- | --- |
| Sex (F = Female, M= Male) | …………. |
| Religion |  |
| Closest health facility | Name……………………………………………………….  Type facility: CHC, CHP, MCHP, other specify……… |
| How much time does it take you to walk from your house to the nearest PHU? | …………… minutes |
| Mother tongue: | ……………………………………… |
| Education level (circle) | None / Primary / Secondary / Tertiary |
| Role in community: | ………………………………………. |
| Do you know anybody who had Ebola? | Yes / No |
| If Yes, what is your relation to that person? | Family / Friend / Neighbour / Other, specify ……….. |

**Consent form - Key Informant Interviews with Community Leaders**

The Umea University (Sweden), Medical Research Centre (MRC, Sierra Leone) and CHaRT-SL (Sierra Leone), are conducting a study regarding messages to improve Ebola treatment seeking behavior in Sierra Leone. The study looks at the various Ebola messages and treatments in use and in development for use in Sierra Leone. We would like to know what you and others think about these Ebola messages and treatments. We would also like to know what you think about attending health services for Ebola disease. If you agree to participate, we would like you to take part in an individual interview.

*Procedures including confidentiality*

This interview is expected to last less than an hour and will be conducted in a private space you find comfortable and safe. If you choose to take part in the interview, your name will only be recorded on this form and will be kept separate and locked away from other documents. To make sure that your information is correctly used, we will record the interview on a tape recorder in addition to taking notes. Your name will never be mentioned in relation to anything that will be said, written down or taped.

*Risk, discomforts and right to withdraw*

During the interview you can refuse to address any question or withdraw from the discussion whenever you wish to do so.

*Benefits*

This study may not help you or your community directly but the results will help to improve Ebola health services and Ebola messages in the future.

*Consent and contact*

- Have you got any questions that you would like to ask?
- Are there any things you would like to be explained further?
- If you do not want to take part in this interview you can refuse to do so and it will not be held against you in any way.

**_________________________________________________________________________**

**DECLARATION: TO BE SIGNED BY THE RESPONDENT**

**Agreement respondent**

**The purpose of the interview was explained to me and I agree to be interviewed.** *(thumb print)*


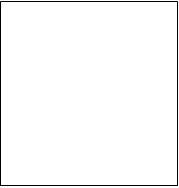
**_____________________________________ _______________**

Signed Date

WITNESS SIGNATURE

_________________________________ _______________

Signed Date

**If you have any questions or want to file a complaint about the research you may contact:**

Mr. Abdul K. Jalloh, Investigator Ebola study and Director, Medical Research Centre (MRC), 5 Frazier Davis Drive, off King Street, Congo Cross, PO Box 536 Freetown, Tel: XXX-XXXXXX, Email: abduljalloh@mrc-sl.org

OR

Sierra Leone Ethics and Scientific Review Committee, Secretariat at Directorate of Research,

Close to ward 10, Connaught Hospital, Freetown, XXX-XXXXXX

# DRAFT Coversheet and informed consent form for health workers and health volunteers

*Ask informed consent, fill in the personal data sheet, then start the interview.*

| CODE |  |
| --- | --- |
| DATE | …………………../………………………/………………………… (DD/MM/ YYYY) |
| TIME – start | (hh:mm) |
| TIME – end | (hh:mm) |
| DURATION (minutes) | (minutes) |
| Voice recorder number |  |
| NAME MODERATOR |  |
| NAME TRANSCRIBER |  |
| District / CHIEFDOM |  |
| COMMUNITY |  |
| LANGUAGE INTERVIEW |  |

**PERSONAL DATA RESPONDENT** (*circle answer)*

| Age *(in whole years)* | …………………..years |
| --- | --- |
| Sex (Female = F, Male = M) - circle | F / M |
| Religion |  |
| Health facility you are attached to: | Name……………………………………………………….  Type facility: CHC, CHP, MCHP, other specify……… |
| How much time does it take you to walk from your house to the nearest PHU? | …………… minutes |
| Mother tongue: | ……………………………………… |
| Role in the health facility / health: | ………………………………………. |
| Education level (circle) | None / Primary / Secondary / Tertiary |
| Do you know anybody who had Ebola? | Yes / No |
| If Yes, what is your relation to that person? | Family / Friend / Neighbour / Other, specify ……….. |

**Consent form Key Informant Interviews with Health Workers and Health volunteers**

The Umea University (Sweden), Medical Research Centre (MRC, Sierra Leone) and CHaRT-SL (Sierra Leone), are conducting a study regarding messages to improve Ebola treatment seeking behavior in Sierra Leone. The study looks at the various Ebola messages and treatments in use and in development for use in Sierra Leone. We would like to know what you and others think about these Ebola messages and treatments. We would also like to know what you think about attending health services for Ebola disease. If you agree to ticipate, we would like you to take part in an individual interview.

*Procedures including confidentiality*

This interview is expected to last less than an hour and will be conducted in a private space you find comfortable and safe. If you choose to take part in the interview, your name will only be recorded on this form and will be kept separate and locked away from other documents. To make sure that your information is correctly used, we will record the interview on a tape recorder in addition to taking notes. Your name will never be mentioned in relation to anything that will be said, written down or taped.

*Risk, discomforts and right to withdraw*

During the interview you can refuse to address any question or withdraw from the discussion whenever you wish to do so.

*Benefits*

This study may not help you or your community directly but the results will help to improve Ebola health services and Ebola messages in the future.

*Consent and contact*

- Have you got any questions that you would like to ask?
- Are there any things you would like to be explained further?
- If you do not want to take part in this interview you can refuse to do so and it will not be held against you in any way.

**_________________________________________________________________________**

**DECLARATION: TO BE SIGNED BY THE RESPONDENT**

**Agreement respondent**

**The purpose of the interview was explained to me and I agree to be interviewed.** *(thumb print)*


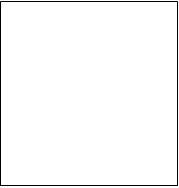
**_____________________________________ _______________**

Signed Date

WITNESS SIGNATURE

_________________________________ _______________

Signed Date

**If you have any questions or want to file a complaint about the research you may contact:**

Mr. Abdul K. Jalloh, Investigator Ebola study and Director, Medical Research Centre (MRC), 5 Frazier Davis Drive, off King Street, Congo Cross, PO Box 536 Freetown, Tel: XXX-XXXXXX, Email: abduljalloh@mrc-sl.org

OR

Sierra Leone Ethics and Scientific Review Committee, Secretariat at Directorate of Research,

Close to ward 10, Connaught Hospital, Freetown, XXX-XXXXXX

# DRAFT Coversheet and informed consent form for FGD - community members

*Ask informed consent, fill in the personal data sheet, then start the interview.*

| CODE |  |
| --- | --- |
| DATE | …………………../………………………/………………………… (DD/MM/ YYYY) |
| TIME – start | (hh:mm) |
| TIME – end | (hh:mm) |
| DURATION (minutes) | (minutes) |
| Voice recorder number |  |
| NAME MODERATOR |  |
| NAME TRANSCRIBER |  |
| District / CHIEFDOM |  |
| COMMUNITY |  |
| LANGUAGE INTERVIEW |  |
| **TYPE FGD** | Male / Female Young (<25 years) / Old (> 25 years) |

**PERSONAL DATA PARTICIPANTS**

| Nr | Sex  (*F/ M*) | Age  (*in years*) | Education Level (*e.g. none, Primary, secondary, tertiary*) | Language (*e.g. Mende, Temne, Krio)* | Religion | Job / Employment (*how they earn their living e.g. farmer, teacher, trader*) | Role in community  (*e.g. youth leader*) |
| --- | --- | --- | --- | --- | --- | --- | --- |
| 1 |  |  |  |  |  |  |  |
| 2 |  |  |  |  |  |  |  |
| 3 |  |  |  |  |  |  |  |
| 4 |  |  |  |  |  |  |  |
| 5 |  |  |  |  |  |  |  |
| 6 |  |  |  |  |  |  |  |
| 7 |  |  |  |  |  |  |  |
| 8 |  |  |  |  |  |  |  |
| 9 |  |  |  |  |  |  |  |
| 10 |  |  |  |  |  |  |  |

**Consent form Focus Group Discussion with community**

The Umea University (Sweden), Medical Research Centre (MRC, Sierra Leone) and CHaRT-SL (Sierra Leone), are conducting a study regarding messages to improve Ebola treatment seeking behavior in Sierra Leone. The study looks at the various Ebola messages and treatments in use and in development for use in Sierra Leone. We would like to know what you and others think about these Ebola messages and treatments. We would also like to know the what you think about attending health services for Ebola disease. If you agree to participate, we would like you to take part in an interview inside a group.

*Procedures including confidentiality*

This focus group discussion or group interview is expected to last between 1 to 2 hours and will be conducted in a private space you find comfortable and safe. If you choose to take part in the interview, your name will only be recorded on this form and will be kept separate and locked away from other documents. To make sure that your information is correctly used, we will record the interview on a tape recorder in addition to taking notes. Your name will never be mentioned in relation to anything that will be said, written down or taped.

*Risk, discomforts and right to withdraw*

During the interview you can refuse to address any question or withdraw from the discussion whenever you wish to do so. This will not influence in any way the care you or your family/community members receive from the health facility.

*Benefits*

This study may not help you or your community directly but the results will help to improve Ebola health services and Ebola messages in the future.

*Consent and contact*

- Have you got any questions that you would like to ask?
- Are there any things you would like to be explained further?
- If you do not want to take part in this interview you can refuse to do so and it will not be held against you in any way.

**_________________________________________________________________________**

**DECLARATION: TO BE SIGNED BY THE RESPONDENT**

**Agreement respondent- The purpose of the interview was explained to me and I agree to be interviewed.**

*(thumb print)*


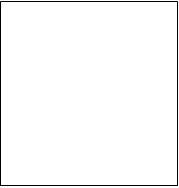
**_____________________________________ _______________**

Signed Date

WITNESS SIGNATURE

_________________________________ _______________

Signed Date

**If you have any questions or want to file a complaint about the research you may contact:**

Mr. Abdul K. Jalloh, Investigator Ebola study and Director, Medical Research Centre (MRC), 5 Frazier Davis Drive, off King Street, Congo Cross, PO Box 536 Freetown, Tel: XXX-XXXXXX, Email: abduljalloh@mrc-sl.org

OR

Sierra Leone Ethics and Scientific Review Committee, Secretariat at Directorate of Research,

Close to ward 10, Connaught Hospital, Freetown, XXX-XXXXXX
