# Supplementary material for: Development of a set of community-informed Ebola messages for Sierra Leone
Source: PLoS Negl Trop Dis. 2017 Aug 7;11(8):e0005742. doi: 10.1371/journal.pntd.0005742 (PMC5560759; doi:10.1371/journal.pntd.0005742)
Supplement: S1 Appendix — (ZIP) [file pntd.0005742.s001.zip › Ebola messages - FGD and interview transcripts/R2HC Ebola Fieldwork 1/R2HC Ebola F1 COM-Rural2 V2 ADD PROBE.docx]

| CODE | **R2HC Ebola F1 COM-Rural2 V2 ADD PROBE**  **(rural semi-structured interview with community leader)**  **V2 - 1^st^ March 2015 - Probing added** |
| --- | --- |
| DATE | January 2015 |
| DURATION (minutes) | 60 |
| Collector nr | 4 |
| LANGUAGE INTERVIEW | Krio |

**PERSONAL DATA RESPONDENT**

| Age *(in whole years)* | 35 |
| --- | --- |
| Sex (F = Female, M= Male) | Female |
| Religion | Muslim |
| How much time does it take you to walk from your house to the nearest PHU? (minutes) | 4 |
| Mother tongue: | Mende/Temne |
| Education level: | Secondary |
| Role in community: | Women’s Leader |
| Do you know anybody who had Ebola? | Yes |
| If Yes, what is your relation to that person? | Family |

**TRANSCRIPT: (M= Moderator, R=Respondent)**

M: My sister morning

R:” Morning”.

M: let’s talk a bit louder

R: “Okay”.

M: the first thing I will ask, when did you hear about Ebola for the first time?

R: “First, last year”.

M: When last Year?

R: “Me, the time I heard about Ebola was April”.

M: Which year?

R: “19 mm, 2014”.

M: So how was the sick described to you?

R: “First, they said you will “upperrate” (frequent stooling), you will get rashes and you will get headache”.

M: So what was your thinking when you first hear about it?

R: “I think badly, I said this is a bad sick that has enter Sierra Leone”.

M: so in which ways Ebola has affected your community?

R:” It has affected us bad, because it has killed most of our people in this country, this community, mostly two of my uncle’s children have died”.

M: Have you personally seen or know a person who has had Ebola?

R:”Yes”.

M: Why do you think Ebola has spread in this country Sierra Leone?

R: Why do I think that Ebola has “scatta” (scattered, spread)”?

M: Yes, what are the reasons?

R: “People are dying too much now, anyone they test, and they says it is Ebola”.

M: You think that is the reason for the spread of Ebola?

R: “Yes, when they advise us not to do this and you do it that has spread the Ebola”.

M: Okay, what again?

R:”Second, when they tell you to wash hand, we will not agree to wash hands, don’t touch dead bodies we will not agree, this has make the Ebola to spread”.

M: What do you think is the best way to stop Ebola from spreading?

R:”One, we have to listen to advise”.

M: Which kind of advice?

R:”The advise that is given by the medical people”.

M: Will you name one?

R:”Don’t touch body, so if we take that advise, it will be better”.

M: What do you think is the best way to treat a person that has Ebola?

R:”one, you have to carry the person to the centre as soon as you hear that the person had an Ebola, carry the person straight to the centre or call 117 to come and take the person”.

M: Is there any way you called Ebola in your language?

R: “No, it is the same thing”.

M: What do you mean by the same thing?

R:”Ebola, English man says Ebola, and we also say Ebola”.

M: Some people do not believe Ebola exists. Do you know people in this community?

R:” No”.

*(Long silence)*

M: Please can you give some examples of the Ebola messages that you heard?

R: “like”?

M: The messages you have been hearing about Ebola, will you give me some examples?

R :”( )”.

M: The messages you have been hearing / seeing about Ebola, will you give me some examples?

R: “about the sickness?”.

M: “Yes so examples?

R: “you will get plenty stool that is one, two, it will give you rash, three your head will ache, four it will make you vomit and toilet”.

M: What do you think of those messages that they are telling you?

R:” it good, is a good advice if you take to it”.

M: What about the way of disseminating this message?

R:”Is fine”.

M: What is the best Ebola messages you have seen and come across?

R:”The best Ebola message, the advice given to us not to touch body, I believe that is the best Ebola message”.

M:”Which Ebola message that you think is not working?

R:”All is working fine”.

M: Is there anyone that is not working fine?

R:”All is working”

M: What do you think would be a good message to encourage people to bring patients to treatment centre/holding centres?

R:”If you have sick person, go and meet the person, talk to the sick person, talk to the parents”.

M: Like which kind of talk you will talk to the parents to encouraged them to take the sick person to hospital?

R:”Tell the person that medicine have come, they are treating people well at the treatment centres, after they will return you fine, give you work, that all is enough when your person(s) sick, take them to hospital for the sickness to stop”.

M: Okay, in event of Ebola infection do you think that people would prefer to go first to the traditional healer?

R:”No, don’t force to a “medicine man”(traditional healer), take the person to the health centre”.

M: What about the hospitals that have been there before, do you think the people will prefer to go there first?

R:”Hence you have the centre in your town, go to the centre at once”.

M: What about the Ebola treatment centres that they have open, do you think if a person is infected with Ebola, the person will go there straight away?

R:”Yes”.

M: Why do you think so?

R:”Because it is the centre where they treat Ebola”.

M: Some people prefer to stay at home when they think they may have Ebola?

R:” That is not fine”.

M: Why do you think they are doing so?

R: “Some of them are fear, but now there is no fear again, because you will see they take your companion, treat the person and come with the person back, and you will see the way they come the person, with convoy, good, and what they give them, that all have given cause for you to go direct to the hospital, don’t be afraid again”.

M: What do you think could be done to encourage them to come to treatment centre, people that prefer to stay home when they have Ebola?

R: “You will go and talk to the person”.

M: Like which kind of talk we should tell them?

R: “Go to the hospital, they are curing this sick now, don’t stay at home or be in hiding then you die here, talk to the person like “you compin mortal man” (like you are all human being)”.

M: Okay, what do you think would be the best channel to get your new Ebola messages?

R: “One, meet the community people, talk to them or you call them on workshop, tell them about Ebola message

M: I want to know the good things people talk about the Ambulance service?

R:”Yes”.

M: like what?

R: “Yes they have the other bad thing gain that they talk about them, but now they have stopped, before this time when they come to collect patient, they will spray plenty”.

M: What do they spray?

R:”They have that chlorine, they spray chlorine that was bringing about the affection, it was affecting the patient badly, but now is better they are not spraying again”.

M: So now what is the good thing they talk about them?

R:”Now they will tell the patient, go inside the Ambulance, we are not spraying again, people were afraid of the spray, they don’t spray again, and they will carry you slowly”.

M: What is the good thing they talk about the treatment centres/holding centres?

R:”They will encourage patient, give you food because you will not go with food and cloths, they will give you all that”.

M: What about the bad things they talk?

R:” This new time no bad yet”.

M: What about the Ebola burial team, what good thing people talk about them?

R:”Now, they are doing the bury fine, first time, they say they do not bury people fine, they will treat the dead bodies of people, but now they are talking fine about them, if you were praying before you die, they will tell people that, okay lets pray the body”.

M: In this community do you think people do secret burial?

R:”No”.

M: Like even those secret societal people, you don’t think they will bury them in the society bush when they die?

R:”No”.

M: What about the 117 phone line Government said we should call in case of Ebola, what are their good things?

R:”They always talk good about them because when you call at once, the message has pass, if it is a sick person come and take or a dead body they will come and maintain”.

M: Any bad thing about them?

R:”No”.

M: What about the health facilities, the health centres that have been there before, what are the good things people talk about them?

R: they are not bringing “bad haat” (indifferences) into the programme, they will treat people well even not with Ebola”.

M: What about the nurses/ staff that worked there, anything good they talk about them?

R:”Yes, they are talking to people fine, when you go there sick, if its Ebola, they will say go over there that is the place they treat Ebola and they will not be harsh with the people”.

M: How do they treat and act to people that have survived Ebola in this community?

R: “When they come with them we will be glad, we will all run to meet and welcome them, they have said when somebody has sick of Ebola and survive they will ignore them, we don’t do that in this our own community, in our own community we will welcome you fine and say since you have come back, we are brothers and sisters”.

M: So they do not treat them bad at all?

R: “uhmmuh”(No)”.

M: Have you heard of any new treatment for Ebola that may become available soon?

R:”No”

M: Have you heard of any new ways to prevent Ebola?

R: “I have not heard of any new functions, we are dealing with the old ones”.

M: Have you heard of any vaccines that may be coming into this country to stop Ebola?

R:”No, unless malaria treatment”.

M: So you have not heard of any vaccines for Ebola?

R: “Uhmmuh”(No)”

M: let ‘talk louder so the recorder will catch it.

R:”Okay”.

M: So you not heard of any vaccines for Ebola?

R: No. I have not heard of it

M: As a Women’s leader, what are the common questions they ask about Ebola in this community?

R: “How Ebola is…”?

M: As a Women’s leader, what are the common questions they ask about Ebola in this community?

R:” They will ask, “De Mammy” (mother) now this Ebola has come, we have got good treatment centres, good Ambulance, we have got good Doctors”.

M: So these are the questions they ask you?

R:”Yes”

M: So how do you respond to those questions asked?

R:” We have got good Doctors, good facilities, is left with you now when you are sick, to go to the treatment centre”.

M: As a Leader, what do you feel you need to know to enable you to be responds more?

R: “They have to ask for the “Marklate” (vaccines), I want to know, how I am going to answer them”.

M: Is there anything specific about Ebola that you think your people need to understand better?

R:” All the things on floor now, everybody understand, because we attend workshops, the medical people also come and tell us, unless the news that have come that we don’t understand”.

M: Is there any good way to explain this to people?

R:” Like When you people come, call meeting and talk to them, like even those that do not hear krio, we have our own person that translate for them, for the ”old Mammys and old Pas”( old women and old men)”.

M: Okay, thanks for taking your time patiently to answer to my question, I don’t have nothing to give you, but this information you have given will benefit all of us, once again thank you.

**ADDITIONAL PART OF INTERVIEW, OBTAINED BY COLLECTOR 1 AFTER CONSENT IN PERSON, February 2015:**

M: Yes ma, I want to know as you said initially, why are the people not washing their hands before now?

R: “Because they did not understand what was going on, and they do not understand the about sick, that was why, they were not washing their hands”

M: They did not understand?

R: “uhum”

M: Ok, what about, when they said they should not touch their body?

R: “Before when the thing started, they were touching their bodies, but now, they do not”.

M: Why they were touching each other’s body?

R: “Because they do not believe, they do not believe whether this sickness kills or it exists, that was the reason of touching each other’s body”.

M: They people said, they are afraid of the spray, I don’t know which kind of spray?

R: “They were spraying spray that has a lot of power”.

M: But do you know which kind of spray?

R: “It was having the smell of a chlorine”

M: So it was having the smell of a chlorine?

R: “Yes”.

M: So they were spraying?

R: “Yes”.

M: They said, why are they spraying it?

R: “First they said the sprayed it, where the dead body was lying, where the corpse was placed. They sprayed the entire house, the rooms, and the leaves outside”.

M: Hum?

R: “Yes, in this community, so it happens”.

M: Ok, I understands the chlorine affects the patients badly, my this chlorine affects?

R: “Because they said the mixture is highly powered” *(A mobile phone ringing)*

M: But why do you think, they are using chlorine?

R: “Why are they using the chlorine”?

M: uhumm?

R: “Because it killed the disease”.

M: Ok, why they are not spraying again?

R: “Because they found out that people died with the spraying of the chlorine”.

M: They have died by the…..?

R: “Yes, people reported that, the chlorine they sprayed, that has led to the death of many people”.

M: Do you know or heard of anybody that I have gone to the traditional healer for healing?

R: “In this of our environment”?

M: Yes, if not here, but have you heard of a person that had gone to the traditional healer for healing, even not in this environment?

R: “No”.

M: Some people prefer to stay at home, when they think they may have Ebola, do you know their fear?

R: “Yes, the spray they sprayed the people inside the ambulance, (*not clear*)”.

M: Ok?

R: “So people were afraid to go to the hospital when they are sick because when they took them to the hospital for cure, they will not come again, unless they come and tell us that person is dead, that again gave caused to people to be afraid”.(*A heavy noise*)

M: Why are the people not fearing again?

R: “Because we have seen when they took a person to the treatment centre, they brought the person back survived”.

M: OK?

R: “It is the good thing”.

M: Can you please give me examples of people of this nature?

R: “They went with one woman (- - name of the woman- -)”.

M: Where did they go with her?

R: “They took her to Kailahun (= first district in Sierra Leone with Ebola cases)”.

M: Ok, so was she cured?

R: “Yes, they brought her back”.

M: Ok, so what about those that died, did they come with them?

R: “No”.

M: But they told you the information?

R: “Yes”.

M: The people accepted that?

R: “Yes”

M: How do they treat the Ebola survivors?

R: “Well they are treating them well, because after they had come back, they gave them job”

M: They give them job?

R: “Yes, they worked at the centre” (*Mobile phone ringing*)

M: The treatment centre?

R: “Yes”.

M: Ok, How do you think of the Ebola survivors, how do you they treat them in the other neighbouring communities?

R: “I thought it is good, because as sooner they come back, we will go and informed the surrounding villages, that this people had been cured, they do not have Ebola again”.

M: Well the positive treatment of Ebola survivors in this village, is it the same in the other communities?

R: “Ebola only broke out here, people died and it is only here we have Ebola survivors”

M: Here?

R: “Yes”.

M: Do you know how many people died?

R: “Eighteen”.

M: Eighteen?

R: “Yes”

M: Eighteen People died?

R: “Yes”.

M: You are talking of this community treating Ebola survivors well, how do the neighbouring chiefdom treat them?

R: “They treat them fine”.

M: But have you heard anything about pointing of fingers on them?

R: “Yes, one village called (- - name of village- -) one old woman was passing and they pointed fingers on her that this old woman was having Ebola, but they were not talking it to the hearing of other people, but they are pointing hands on them”.

M: How do the survivors feel?

R: “They showed up understanding, because we using tell them, this Ebola sick is not in some villages, so if they point fingers on them, they also pretends”.

M: Thank you very much ma that was what I wanted to clarified.

R: ooooh
