# Supplementary material for: Development of a set of community-informed Ebola messages for Sierra Leone
Source: PLoS Negl Trop Dis. 2017 Aug 7;11(8):e0005742. doi: 10.1371/journal.pntd.0005742 (PMC5560759; doi:10.1371/journal.pntd.0005742)
Supplement: S1 Appendix — (ZIP) [file pntd.0005742.s001.zip › Ebola messages - FGD and interview transcripts/R2HC Ebola Fieldwork 1/R2HC Ebola F1 COM-Rural3 V2 ADD PROBE.docx]

| CODE | **R2HC Ebola F1 COM-Rural3 (rural semi-structured interview with community leader)**  **V2 – 2^nd^ March 2015 – Probing added** |
| --- | --- |
| DATE | January 2015 |
| DURATION (minutes) | 41 |
| Collector nr | 3 |
| LANGUAGE INTERVIEW | Krio |

**PERSONAL DATA RESPONDENT**

| Age *(in whole years)* | 65 |
| --- | --- |
| Sex (F = Female, M= Male) | Male |
| Religion | Christian |
| How much time does it take you to walk from your house to the nearest PHU? (minutes) | 10 |
| Mother tongue: | Temne |
| Education level: | Tertiary |
| Role in community: | Village Development Chairman and Pastor |
| Do you know anybody who had Ebola? | Yes |
| If Yes, what is your relation to that person? | Friend |

**TRANSCRIPT: (M=Moderator, R=Respondent)**

M: Interviewing a pastor, Good Morning Pastor?

R: “Good Morning”.

*(Someone striking an iron)*

M: When did you hear about Ebola for the first time?

R:”Hmm, Ebola we heard of it, the time I heard of it, it has taken long now, because we heard of Ebola the time it hits part of the Mende side, when it hits there, it’s the time we begin hearing about Ebola”.

M: You?

R:” Yes”.

M: How do they tell you about Ebola?

R:” We have been hearing it from radio, then other people themselves come and tell the people about this sickness”.

M: What did they talk?

R:”They will tell the people that sickness have come which it kind have ever come in the world, which is Ebola and is a killer disease and really the way we have seen it and we have seen the way Ebola has almost destroy plenty families”.

M: Okay, what were your first thoughts of this Ebola?

R: “Well me, although I am a Pastor but I was feeling that sometime the bad things we have been doing too much, that has make God to vex on us”.

M: In what ways has Ebola affected your community?

R: “Well it has make even the worst of it now, it has stopped education, there is no free movement, plenty things people do even trading like “luma”(special market day) they have closed them, even sometime will come government we say let them sit down for “so so” number of days person will not move and “boku boku”(plenty) things, it has stopped plenty things which is for development and even the social activities, no dance, no football and “boku boku”(plenty) when a person has rest can able to do, that has been banned, we are not getting those recreational activities again, all that is a set back because a human being will be working for the whole day, when time comes you need to relax, those things are not happening again”.

M: Have you personally seen a person who has Ebola?

R:”Yes”.

*(Long silence)*

M: Why do you think Ebola has spread throughout Sierra Leone?

R:”Well Ebola, because of the way people don’t believe that Ebola is a reality, the message some people don’t take and the precautions which they tell them do this, don’t do this not “boku boku”(plenty) people that adhere to those things that is why Ebola has spread, look like, let me compare a place like Nigeria which their population you don’t even compare it to Sierra Leone but when they took the corrective measures which they told them, they were able to fight it quick and finish, but we, our people still adhere to this native customs, to wash body, to touch dead bodies and all the other things, the burial, they said let person do not bury again but some people are still doing it, some when they go burial they will decide to go, we have been hearing it from other places they go and pull back the body , they say they do give the correct burial so they will come and do it, all this one makes “boku” (plenty) people to get it, we have heard it in other places when people are doing things like this one, all that did this havoc themselves, they all will die”

M: uhmm, okay, the other question I will like to ask again is this, what is the best way to prevent Ebola from spreading?

R:”Well to me, I feel that Ebola to me is an acronym, that E there means for educate, they have to educate the people, though the people ready to take the Education, the B is to believe that Ebola is a killer disease and the O, you have to obey, why you have to obey, that is why they says “ABC” avoid body contact, “APC” avoid people’s compound but people are to agree to those things, they are not obeying what they are telling them, then the L is for listening, people are listening but they are not adhering what they listen and what they are telling them about how dangerous is the sick and the A is to avoid, avoid all other practices which they say if you do it you will contact Ebola. If they adhere to this one and if the really know and understand what is the meaning of this Ebola (E.B.O.L.A), if they take all the precautions they tell them, I think that this sick should not spread like the way it is now”.

M: Okay, thank you for that message, then what I want to ask again, what you think is the best way to treat somebody with Ebola?

R:”Well to me what I know, because they have shown us, when you know a person have contacted Ebola, they said you have to isolate, but one person will get other preventive measures, you will take plastic and put it in your hand, if you have to help any help, to prevent yourself from getting the disease but that person will not be with you people again, leave the person space where they will be coming to collect the person immediately, because you will not say when a family member is sick, you have to avoid or abandon the person and leave at the house, please they need to give some help, because even the mishandling they have been mishandling the patients that makes “boku”(plenty) people to die and we know that “boku”(plenty) people that died not Ebola that really kill them, some of them is the mishandling that they were mishandling them and the same time the way they were travelling with them, long long distances , those long distance, so obviously they come and take patient from here and take far away to Kailahun and with heavy speed and when they will be going they don’t give water, only medicine, they will travel with the patient until the patient die, sometime some are malaria patients( *voice at the background*)”.

M: thank you, what I am asking again, do you have any other word for Ebola in your language?

R: “Well we don’t ever have this word in our own Temne language only that we know it, we call it in Temne (disaster) “Gbalow”

M: What that means?

R: “trouble”.

M: Okay thank you, some people do not believe Ebola exists?

R:” I know that some still don’t believe unto this very moment”.

M: Can you tell me why they don’t believe?

R: well you see why some of them don’t believe, one is this lack of education, and the illiteracy rate is high in this our community and people still adhere to those native customs, they don’t want to leave this their native customs”.(*horn of a motor bike*)

M: Which kind of people that think so?

R:”mmm, let me say like the Muslims, they usually say when someone dies like a sheik, they supposed to give him that last respect, they have to wash, dress, and pray on the body then go for burial and it happens if they do that, they will get trouble and some again because of those societies, they are still feeling that the person, the society that he belongs to when he dies they have ceremony to perform, so if they don’t perform it for that person it means they don’t bury the right way”.

M: Okay thank you, please can you give some examples of the Ebola messages that have you heard, seen or read?

R:”Yes”.

M: What are they?

R:”Like how I said because I have being going to plenty of those workshop of Ebola, they have been telling us that avoid body contact and even this one we don’t able to avoid people’s compound because if you are at your house you will not able to go and get trouble if there is trouble in the house. That is why even in the bible, this quarantine is there, you will hear in the old testament when someone get leprosy, they were isolating them, they will not allow the person to join the other people, they were feeling that it is a contagious disease, so they are telling us all those things”.

M: What do you think about these messages?

R: “These messages I feel are beneficial to those that take them, what they say to do and what they say not to do, so if you adhere to those advises I think it is beneficial to you, then not only to you but even the community because Ebola was brought to town by only one person which people did not know, straight away the ignorance that makes some of them contacted but if people had known before as long as they know that this had get Ebola, all the precautions, they would have just avoid that that person then send, some people hides sick person that time, but now there are contact tracers and those surveillance officer also come, first time some people is sick, some will decide to hide the sick person, as you see a person have started development those symptoms, stooling, vomiting pulling blood, so if you contact that person the possibility is there to get that trouble”.

M: Okay, with all the Ebola messages what do you think is the best for you?

R:”The Ebola messages”?

M: Yes, when you see and read?

R:”Well the best, like how I said, Ebola is an acronym, if you adhere to all those things, the E-B-O-L-A, if you adhere all this things, that I feel is the best message which I have been getting”.

M: Are there any Ebola messages that you see, read, you think have not worked?

R:”Me, I don’t see anyone that is not working”.

M: in this community?

R:”uhmmmuh”

M: Are they all working

R:”Yes, because they told us that even someone has got Ebola and well, when the person comes back you have to treat the person as your “fambul” (family member) you don’t need to stigmatise the person”.

M: What I am saying as pastor among the Ebola messages you see and read, which ones that worked best or the one that do not work?

R:” The one which I know is working, like the one they said Avoid body contact is very important because, but that has created problem for us, fantasy go to the market and see even though they are warning them, you will see them rubbing skin, touching, some people even this greeting, they say we don’t have greet, up to this moment they are greeting, they said they have used to it, is a problem. Some will even go and grip their companion, all these things you don’t know the person you are doing this thing to have the trouble”.

M: What do you think would be the best message to encourage people to bring their patients to the hospital?

R:”To us here it has help plenty people, because some people really are not Ebola cases because sickness like typhoid, malaria they have almost the same symptoms if its attacks a person, they will able take the person straight away and call 117, if they carry the person to care centre like this community care centre, they will give the person treatment and observe the person one to two days if still, that is why they have these thermometers , they will check the person with the symptoms which the really know the person’s temperature has risen, they give medicines to the person, but still complaining headache even when they give medicines, they are some of the symptoms, the other people is malaria, we “mortal man” (human being) is natural, if you have eaten something that do not fit the stomach you will vomit or “kaka run bele”(frequent stooling), a person should not be persistence now”.

M: Okay, in the event of Ebola infection, where do some people prefer to go first, to a traditional healer?

R:”Some will go to a “medicine man” (traditional healer) because they may not believe that they have got Ebola, other people as you have some of brothers here and sister that will volunteer to go to the health centre and they go they will check if the symptoms are of Ebola they knows where to send the person and if you go earlier for treatment, I have seen people in our community who have gone and come back survived”.

M: Some people decide to stay at home when they think they have Ebola, do you why?

R:”Well sometime other people are for the stigma, some feel when you got that sick, that is why government has said if someone has got Ebola and survive don’t call that person as an Ebola patient again, that is why government has passed that law, because stigma is bad to “mortal man”(human being), lets fancy the war that was there, the RUF came and rough us, when they say peace and reconciliation, we forgive and forget although we will not forget the atrocities they did to us”.

M: okay, what makes people to have courage to go to the treatment centre?

R:”When a person(s) is sick, they need encouragement, they need education, to tell the person,”bayo bayo”(pamper) the person that the best way is let go and find medicine”.

M: What do you think would be the best channel to get new Ebola Messages out?

R:”Well because the best message that I feel”.

M: The best way or channel they send the Ebola messages them out either through radio anybody which one will they use?

R:”Well I think not everyman is able to get radio, but I think that the people that pass with the vehicle, that makes advertisement to tell the people the dangers of Ebola or how if we see a person sick how to take that person to the hospital and I feel that it helps because not everyman has access to radio but the vehicle that passed and enter in these villages they will go and tell the people, the people will hear and if they are hearing, because some have seen the danger of what happen with Ebola and some are seeing Ebola patients”.

M: Have you ever heard anything about the Ebola Ambulances that pass?

R:”Yes, what people have been talking, I myself will discourage of that sound, because the way they pull the sound, I will know that another trouble has fall on another person, especially the ones that, we were having one nurse at (--Name of a nearby village--) her children always feel bad when their mother has die and they have quarantine them, at any time they hear that “Matoca”(vehicle) passing with that sound, its reflects on the them badly and they will cry, that is why they have reduced the “hala” (sound, siren), when they get Ebola patient to start “the hala”(sound, siren) “whooooo”, they have come for a person or if an Ebola person dies when they come to collect, that was discouraging the people”.

M: Do they have anything good or bad they talk about them?

R:”Yes of course, people were saying, some were not still believing that when they put a person in the ambulance, they will spray and look the ambulance that suffocation kills the patient”.

M: Okay, what about the burial team?

R:” well the burial team, the way they are burying now is better, before now when they come, as they come dress and enter the place pump the entire were the person is lying and when they were taken the person to bury, it was not going down well with the people, because they were not handling the corpse with respect, although is a law that government has past that anybody dies they should put the person in plastic, but the way they were doing it people were not really happy. At one time a problems has been arise here in this village, they were having confrontations with other youths”.

M: How it happens?

*(Someone greeting)*

R:”Well the way they came and take one woman, the family did not feel satisfied, so they started grumbling and quarrelling arise between them and the burial team, they become vex and started throwing stones but the police was able to control the situation, there were soldier also”.

M: What has change now, when you said before it has been happening?

R:”What has change now, first when they come to collect they will not even say to prayers for the person, but now they will allow, they will even give them that “srold” which they can even wrap the person with before they put into the plastic, then they will put the person to a distance then if the person is a Christian, they will ask the pastor, he will be far off and pray for the person, he will go back to the grave side, they will put the person far off and pray, but first they were not accepting that, hence they come and take the person they will just go”.

M: What about the secret society people, which will like to perform their ceremony, will they do that?

R:”No, they are not allowing it, now from when Ebola breakout they are not allowing that, we now, like before when everybody have aware of this danger, when a person dies there is no society business involved, but of course other areas am still hearing, like in Port Loko they are carrying those societal business, that is why problems are still arising in the area, but us here, it is not happening no society business is not include again, when someone dies they will just call and they come and collect, if it is that scrold they call and wrap the person and put in the plastic, if it outside they call for prayers and they go for burial, they will place the body in the grave, undress themselves and put the cloths in the grave and put sticks and leaves, the people will cover it now and they will go”.

M: What people talk about the holding centres/ treatment centres?

R:”Like the caring centre is better, but the holding centres they were really holding the patients carelessly, when they take them there they just leave them, they don’t give them medicines, and they don’t give them food, the patient will left there and die, even the other boy ran away from the centre were they took him”.

M: Which side, was it far?

R:”No, it is in Makeni, that Arabic hospital, where they took him, but because of the mishandling he ran away and where he went that night the people already knew him that he has that trouble so they were able to alert, call 117 and the police, they came and took him back, the other bad thing about it again, when it happens last, when we were having a workshop, I gave the command centre that information, then the patient that they take, people will not get feedback whether the person had die and you may not know, and people has the interest to know if he still lives, like one boy which they took to Kailahun, we don’t know even if he has died or not ,I even gave all his details but up to date we don’t know whether he has died or not”.

M: But any good things about the holding Centre, because you have explained the bad ones any good?

R” well the good things now, that am seeing, the survivors, are given plenty things before they return home and they will advise them again to give helping hands at the care centres”.

M: Thank you, what about the 117 phone line, what is the good and bad things people are talking about it?

R” Well the good thing, is to help the community, you are not buying credits it is a free line, because you will be at the place where there is no credit that may be a problem to you, any time you call is a free call although there are delays because I don’t know if it was work pressure or they stretched them too much, because they will call them but don’t come earlier, I don’t know if it is the workload”.

M: Any aspects of the existing health facilities/staff that is now working on Ebola care and treatment?

R:”Well this care centre, when you go there, they will check you, if your temperature is too high they will put you somewhere”.

M: What is the name of the care centre?

R:”Community care centre (--Name of the interview village--), there you have the wet zone and the dry zone, wet zone is the place, they put people whose sickness is not worsen, when they take you to the dry zone it means the sick has worsen, you are only waiting for ambulance to take you or it happens you are at the point of death. They also have mortuary where they will put you. They will lay there at the sometime give the patient medicine, give food three times per day. There is also provision for the family members to talk to the patient, there is a line of demarcation between you and the patient”.

M: How do people in this community act to people that have been infected with Ebola and survive?

R:”For me they are just accepting them back as their family member and they are thanking God for serving them for that sickness, people are really appreciating that have seen them again, because some people that they took away, did not return back, so we must appreciate the ones that survive. Like during the war when you and your people separate then meet again, you will be happy. The survivors also are happy because they have come back to their community, and they are happy to join their community back maybe where they took them, they don’t know anybody and they do not hear or speak their language”.

M: In future, if there will be any person that will think of treating the survivors badly, what will be your message for that person?

R:” what I have to talk to the person, is that what you mean”?

M:I said the person that is infected and survives what would be your message to people not to treat them badly?

R:”I will tell them this, nobody is praying to get sick, nobody is praying to get trouble, they did not do anything bad, is sickness and this sickness if person is infected, let’s say it is God, it is God that have destine it, like other people will play with a sick person. Like we have a child here who has lost the father from Ebola, but the child survives, they do not even take the child to treatment centre, they were under quarantine until the child survives”.

M: Have you heard of any new treatments for Ebola that may become available soon?

R:”we have been hearing because I have not seen that they discover medicines to come and fight this Ebola”.

M: You heard of it?

R:”Yes I heard it”.

M: what were they saying?

R:” they said they have got medicine to come and help the person with Ebola, but I did not see it with my own eyes because am not working at the centre, although at the centre they do not have the medicine there, they only give you treatment to reduce the fever, if it is pain, headache, “runbele”(frequent stooling), vomiting to stop, if it stops, they will say is not Ebola, but if it continues and persist is the time they will know that this person should not be under observation, they will call at once to come and collect the person”.

M: What will be the concern of you community people for this new treatment?

R:”The concern of our people, they really appreciate, in fact what we did here, we were able to help the centre of itself, supply them with water and provide fire wood for them, the other quarantine house around, we will alert everybody that is around to be assisting them, supply them with water. Even the last house that they quarantine, I organised the youth to go to their farm, harvest and “flag” (thresh) the rice they planted and pack it for them”.

M: Do you have any way to prevent Ebola in this community?

R:”I don’t have any new one”.

M: But have you heard any new way to prevent Ebola?

R:”I have not heard or have any new way to prevent Ebola, unless the ones I know, that is wash your hands with soap, if you have sanitizer use it, then the other precautions like when a person shares cloths with another person, that you will expect to get the trouble”.

M: Have you heard of any vaccines for Ebola that may coming into the country soon?

R:” I have not heard of the vaccines yet”.

M: As a pastor, what are the questions they usually ask you about Ebola?

R:” they ask questions like, why did the Ebola come, for me I just take it as, they have to know even in the bible, we have been hearing about the ten plagues, which came to the Egyptians God was annoy with them, and I was saying that the bad things we have been doing, even the code of dressing is not good, people are not happy with it, and God itself is not happy about it, because each human being was made in God’s image and likeness”.

M: How do you feel about the questions/messages that they ask?

R:”For me, I don’t feel bad, I felt fine because I am able to explain to them, those that do not know, I will let them know, so that they will avoid of getting the trouble like how I was saying, if they adhere to all this, I think that will help them”.

M: What do you feel you need to know as a pastor to enable you to respond more effectively?

R:” I want to know, what really cause of this Ebola, although we are getting messages about the places where Ebola first hits as way back in the 70s. According to history they said 1776, I don’t know if the date is correct, is the time that Ebola first started until it spreads all over and DR Congo is the first place I hear about Ebola, so if they educate the people about this Ebola, how it started and spread, that will be of good help, especially if they print them on leaflet, paper and distribute them to the communities and even the precautions should be printed on papers, leaflet and give it to people that can read, they will read and pass the messages to those who cannot read. Not all the pictures that is drawn, though necessary at times, more messages need to come, to educate the people”.

M: Which message you will like?

R:”Let people believe that is not about lie, Ebola is a real, let them continue to get this education and continue to say it. Because Ebola is more than the war that we were having”.

M: Thank you, what is really specific about Ebola that you think people need to understand better?

R:” what is specific that I think, people should know?

M: Yes?

R:” people may bear in mind that, it is by luck for people to survive when they get Ebola, let say if ten people have infected with ebola,

out of the ten only one or two will survive, so let people know that Ebola is “family monddo” when it enter a family it will destroy all of you”.

M: How do you explain this to them now?

R:” Some of them this thing is a practical issues to them, they have seen it in their villages. Some other villages have migrated because of what Ebola has cause, for example in one house four to five people will die for the day”.

M: thank you, this is the end of this interview.

**ADDITIONAL PART OF INTERVIEW, OBTAINED BY COLLECTOR 1 AFTER CONSENT IN PERSON, February 2015:**

M: Sir this just an addition to what my friend asked you the last time. Why is it that some of them do not believe that Ebola is real?

R: “Well this has not started today; from time immemorial even when HIV AIDS came, people did not believe even though people proved it to be reality, and some because of the culture they just think that they should hold on to the culture”.

M: Like what aspect do you think is their culture that they holding on to?

R: “Like those of our societies that we have, just like when HIV AIDS came people just took it as an acronym when they said it was America’s Intention to Destroy Sex (AIDS) so they held on to that even though it has been proven by scientists and is the thing with this even though they have seen what Ebola is doing, which is a family killer, and where ever Ebola kills a person, it will not only kill one person?

M: But what is the reason why people do not want to leave their culture?

R: “Some of them feel that they have been living by these cultures and is something that is heritage left by their great parent, this culture about them and it has been with them they cannot leave that behaviour and it will continue except the other generation, is just because of the Ebola outbreak like at this time now in this area like the Poro (=men’s secret society) would have gone hay ware, the Bondo (=women’s secret society) would have gone hay ware and also the ‘Degba’ (Positioned women in the Bondo society), Ojeh or what not, but with Ebola now when government put a stop because of the gathering”.

M: Is it true when they say people go to tradition healer when they are sick?

R: “Some people still have that belief”.

M: Ok.

R: “Because some people it is not their first time they have been going to those people so when they go there with the person’s advise and with help of God because it is not the person’s own help but with the help of God, and he or she happens to see something like healing he or she will recover, even though many cases they fail, they still hold on that. Some can tell you that they believe the person, that whatever the person says he will not be saying lie”.

M: Can you give me examples of some of the things they say when they believe the person?

R: “Well the example is when some of them go to do harm to their companion. When they clash with their companion he or she can go there for the person to cause trouble to his or her companion, when the person gets the trouble so he or she can believe that man”.

M: So who the group of people who goes to those people?

R: ”Well to me, I cannot just put it that they are the ones who are not Christians or Muslims because they are the only ones who pretend that they are Muslims or Christians but they not believers those are the type of people because any problem which you have, if you know that there is God you have to pray to God that is why God told us that ‘Seek and you will find’, ‘Knock and the door will open to you’, ‘Ask and it will be given to you’. If you really ask in good faith, AI am sure God will help you”.

M: Despite religious people, who has that faith, is there any other kind of people who you suspect that they are the ones go goes to those traditional healers? Who are that people, which kind of people are they?

R: “They were the ones I have just mentioned when I told you that those who pretend to be but he or she is not”

M: What about rich people? Do the poor, men women, which kind of category of people?

R: “Even rich people, some people go there for security or go to continue to get wealth and some go there to secure his or her life, say other people go there to wash like when they say they are going to steam a person which we call in Temne ‘Akulkor’, they have those leaves which they put on you, the person believes that after washing even if they fire him or her with a witch gun it will not act against him or her”

M: Ok, have you heard about any secret burial taking place?

R: “Like where I came to realize that people do not still belief that Ebola is real, one case arouse in (- - name of headquarter town of the interview district - -) when a child died and it was a place that had been quarantined, the people held the body of the child about eight years or so, they held the child since security was around so they were now trying to dump the body into the toilet but it was not possible because the hole of the toilet was too small so the secret leaked and I know the people are now in government hands”.

M: What do you think is the reasons why they are doing that?

R: “I can’t say the reason is, in this of our own area it is illiteracy. The literate people are dormant; the illiterate people are in greater number because if a person can’t read and write some of those things you can know”.

M: Apart from what you have just told me, do you know of any secret burial that had happened before?

R: “No, in our area here that is not happening, because we as pastors play a very great role. We preach it in the Church for them to know that Ebola is real and I even you to tell them that to me that word; ‘EBOLA’ is an acronym every letter has a meaning”.

MM: Ok, if you were an Ebola survivor, which kind of problems do you think you were going to encounter in the community?

R: “Well in other areas, if I were an Ebola survivor, I think that stigma is the thing. Thank God government put a law that anybody who tells an Ebola survivor that he has Ebola that person will have problem with government and that has been there always there it did not start today. That stigma is always there and a person does not pray to God for sickness, even accident but if it comes your way maybe even your enemy would want to provoke you”.

M: But how do you think Ebola survivors are treated when they return into the community”.

R: ”Well to us here, think when they returned we received them just as we received our brothers who had done bad to us because the Ebola survivors have not created any problem but some of our brothers who joined the rebel war, but when government said we have to forgive and forget, although we cannot forget because if you had wronged me and you come back my mind will not forget but I will pray to God to forget what you have done to me but it is not easy to forget easily, especially if somebody had burnt my house and took all what I had and he comes back he met me suffering if I see that person it is not easy for me to forget my mind will go there that why am I suffering because of this person”.

M: But how are they treating survivors here?

R: ”No for us here as they came we accepted them because for us here government give them paper to show that the person had survived they come here directly to the police station and hand the person over to the chiefs then they can take them to their houses and we do talk to the people, even those who are presently who happens to be survivors we were able to let them be at the community health centre here working there, at least they can get food there and even if it is a small token they give them there. And other programs are coming up like TRUCARE we hope to slot them there, to come and assess them first in agriculture and later they will come and give them something like Micro Credit or what not, they are on that, in fact I am supervising that and I am second focal person at the Community Caring Centre”.

M: But how do the other villages look at them?

R: “Well to me when Ebola break out in this part of our country in (- - name of interview district - -) we are the we are the first victim, (- - name of interview community - -) and the house where it entered I think they lost about eighteen lives there, eighteen in the sense that there were two pregnant women who did not survive so it is just approximation”.

M: Ok, so in total how many died here?

R: “The total of people who died here, let me say is nineteen, eighteen in one house and then one man who was working at (- - name of a big commercial company working in the interview district - -) who also went and get his own trouble but he alone died there at the other house even though they quarantine the house. Even his child who was taking care of him, God helped him the child is still there nothing is wrong with him”.

M: Ok, but how do they treat these survivors in other areas like for you, you share boundary with (- - name of neighbouring chiefdom - -), how do the next Chiefdom treat the survivors?

R: “Well I know that these are not people who have been labelled say, these are people who have been tagged that they are survivors except a person who knows him in the other chiefdom can say that person had Ebola but God helped him to survive but I would not know how other chiefdoms are treating them to be frank”.

M: But what do you think how survivors are treated outside your village?

R: “No that individual difference is there”.

M: What are the things which you feel or suspect that is happening in the other villages?

R: “Well if the person who understands the person that he is a survivor, that is how human mind behaves no sooner he see the person his mind will strike and he not only try to keep quiet he try to tell the other people that this man is so, so, so - not to the hearing of the person but will talk about it for the other people to know”.

M: Thank you very much Sir, this was just what I wanted to ask.

R: “Thank you, you are welcome”.
