# Supplementary material for: Development of a set of community-informed Ebola messages for Sierra Leone
Source: PLoS Negl Trop Dis. 2017 Aug 7;11(8):e0005742. doi: 10.1371/journal.pntd.0005742 (PMC5560759; doi:10.1371/journal.pntd.0005742)
Supplement: S1 Appendix — (ZIP) [file pntd.0005742.s001.zip › Ebola messages - FGD and interview transcripts/R2HC Ebola Fieldwork 1/R2HC Ebola F1 COM-Rural4.docx]

| CODE | **R2HC Ebola F1 COM-Rural4.docx (rural semi-structured interview with community leader)** |
| --- | --- |
| DATE | February 2015 |
| DURATION (minutes) | 44 |
| Collector nr | 1 |
| LANGUAGE INTERVIEW | Krio |

**PERSONAL DATA RESPONDENT**

| Age *(in whole years)* | 32 |
| --- | --- |
| Sex (F = Female, M= Male) | Male |
| Religion | Muslim |
| How much time does it take you to walk from your house to the nearest PHU? (minutes) | 30 |
| Mother tongue: | Limba |
| Education level: | None |
| Role in community: | Imam |
| Do you know anybody who had Ebola? | yes |
| If Yes, what is your relation to that person? | neighbour |

**TRANSCRIPT: (M= Moderator, R =respondent)**

M: Which time did you hear about Ebola?

R:”May”.

M: You heard of it from May?

R:” Yes sir”.

M: What did they tell you about the sickness?

R:”First when this sickness was in Guinea, they told us that “boku” (plenty) people have died in Guinea, even that time our sisters in Guinea came to Sierra Leone to play”. We were told this sickness has no cure, as soon you got the sickness you will die.

M: How was Ebola described to you?

R:”They told us that if Ebola catches you, blood will run from the nose and all other part of the body, you will get fever, you will catch cold, you will “runbele”(Frequent stooling, diarrhoea)”.

M: When you heard of it, what came into your mind?

R:”Well I was feeling that, this sickness which has come, all of us have to prevent it, because this is not the first dangerous sickness that have come, there are other sicknesses that have come and have the same act as Ebola, like Lassa fever and our forefathers were having leprosy, there were other sicknesses, which are the same when you get them. The person that has sickness should not touch the person that does not have sickness.

M: Which way Ebola has affected you in the (--Name of interview village--) Community?

R:”It has affected us a lot, education is not moving forward again and even the things that we are managing our lives with, when we were working, plenty of them has spoiled, so it has affected us a lot”.

M: Like what?

R:”Like when we were harvesting some cassava and other things, they were taken to town for sale but this year nothing , we did not have movement like that because they lockdown District to District”.

M: Why do you think about the spread of Ebola throughout Sierra Leone?

R:”it is denial, people were denying, some people were saying Government is just finding money but for us that know something, we know it is not a place to find money, if they were not denying, like when the sickness reaches in Kailahun (first district with Ebola cases in Sierra Leone), it was not going to spread all over, it should have stopped there”.

M: Why are the people denying?

R:” We in Sierra Leone, why people deny because of the political parties, we are divided by party lines, as long as you are not supporting the party in government , whatever the government say the others who are in the opposite camp will deny ,this was the reason why it was spread from Kailahun (first district with Ebola cases in Sierra Leone) to the entire country , one will just sit down and say government just want to do something different and the others will come with theirs and people will deny that this sickness does not exist it is just a way of finding money. That was what affected us the more, until it has spread all over the country, that is the time people started believing and up till this some people do not believe”.

M: Why do you think some people are still denying despite the information given to them

R: some will say these symptoms of ebola are similar to other sicknesses, and by touching someone who is sick, coming closer to your loved one by sympathising with him or her and that can only be done by touching, so it is not easy to say don’t touch , for e.g. if one is sleeping with his wife and suddenly he got attacked by fever , can you say don’t touch the person ,if the wife got well she will say you only love me when am not sick , but when am sick you will avoid me, and this will bring separation between wife and husband, not so ?

M: Will you tell me in your way, how to prevent Ebola from spreading?

R:”Yes, what I was thinking in mind, one, and the government has tried and has done everything for the people, but when government is not “wicked”(taking strict measures) with the people they will not take it serious”.

M: What do you mean when you said “wicked”?

R:”For now, what makes Ebola to spread of Ebola, like those who have survived from the Ebola, they have told them not to do “Mammy and Daddy bizness”(sexual intercourse) for three months but they are denying, right now these are the case that is “hamburging”(disturbing) us in XXXXXXXXXX (district name)”.

M: Any other method you can think of to prevent ebola?

R: cleanliness is something that should be applied, make sure we clean our toilets, houses, and have a health facility at every village

M: Which is the best way will you recommend to stop Ebola from spreading?

R: ”Well one, I want the government to remove the business of money from Ebola, and the money they are paying the people is too much, if that continues, Ebola will not quickly stop, because here in Sierra Leone things are difficult, and any countries where there is difficulty, if they are to not paying you five hundred thousand Leones per month and they start paying you five hundred thousand Leone a week, plenty of them will not want this sickness to get finished because they want to get rich and we the poor ones that do not have suffers, and we are suffering”.

M: Any other way you think can stop Ebola from spreading?

R: All of should continue to talk to our people, as for me every Friday it is part of my sermon to educate the people about the dangers of the sickness, and this has helped, because we have some verses that will tell the people that if you go against government laws, more so on this sickness, God will hold you for your actions, because you have been warned, and this has helped us greatly to prevent people from touching sick ones.

M: What do you think is the best way to treat to somebody with Ebola?

R:”They have to take the person first to the holding centre, after which they will transfer the person to the treatment centre, but for us in the village or you that is having that sickness, should not touch, you have to find the medical people, who will take and take care of the person. If the person has cured, what they are telling, let the person come and sit down for that three month. If that happened Ebola will leave Sierra Leone and people should stop attending burials, stop touching dead bodies so Ebola will leave us”.

M: Are there any local names in this community people use to describe Ebola?

R:”No, here we all called it Ebola”.

M: You don’t have any other term use for it instead of Ebola?

R:”No, We don’t have any other name or term for it, unless in the Limba language, “bola” is a Limba language/ word meaning “BUY”. “Bola” that is how they take it”.

M: So the people take it in this community?

R:”Yes, when they say “BOLA” (go and buy) and if you don’t buy it, you wouldn’t get it, like when they said don’t touch, I will not touch and I will not get it, when they said don’t go to burials, look at me sitting down, all my aunts had die in (--Name of another District--), I am not going here, look at me sitting, so that is how I came to believe that this sickness exist, they said “bolo” go and buy and if I don’t buy, I don’t get”.

M: Some people do not believe Ebola exists, do you know those kind of people in this your community that have this believe?

R:”To us here in our own village we tell God thanks, at first they were denying, but those who denied are no longer alive , they are dead, with only one survivor , so because of that people in this community no longer have that denial anymore, because of the deaths of these people I told you, has changed the minds of our people, when this sick woman came to our village , we told her relatives that we are not going to allow this woman into the village , they decided to travel with the woman in the bush giving her traditional medicine and later the woman died , she was buried secretly in the bush , without our knowledge , just few days those that were part of the burial got infected and they were taken to Kailahun( (first district with Ebola cases in Sierra Leone) where they died ,and the entire village was quarantined for 21 days, now we are over 32 days without a single case.

M: So do you know, why they did that?

R:”Well, only them that did the act will tell, but I can’t tell,

M:I know you can’t tell , but what do you think that may have prompt them to take such a risk ?

R: oh my brother let Allah help us

M: AMEEN (let Allah answer)

R: It is because the sick woman is their blood sister, that is why they attended her and help to cure her.

M: please can you give me some examples of Ebola messages that you heard, seen or read about?

R:”The Ebola message that I have seen and what I believe is that, if a person don’t touch, you will not be affected. Even the ones that were dying here with the sickness, when they said we should take them to (--name of larger town in the same chiefdom as the interview village with the highest level of PHU – a CHC --), they called me because I have two bikes here and I said no”.

M: What I mean have you seen any Ebola messages?

R:”Yes sir”.

M: What did the messages read to you, and what did they tell you?

R:”Well, the messages tell me that, if you don’t touch, you don’t attend burials, you do not involve yourself in secret societies movements, and you stop all the activities, we will not get Ebola”.

M:” What do you think of those messages that they tell you?

R:” They are very good, because when we in the villages are not doing those things again, we tell God thanks, and let we don’t lodge a stranger that have left here for one week, or when the person comes, we have to put the person in a separate place for twenty one days, if we are ready to lodge the person, but if we are not ready to lodge, we will tell the person to go back where he/she came from”.

M: But are people accepting those messages?

R:”Yes, they were accepting it, if do not accept, there are laws then we go with you”.

M: Well apart from the law, like how you and I are talking one to one, do you feel people are accepting that?

R:”Well in this village they have accepted it”.

M: The people understand the message?

R:”Yes sir, this message went through, because we are telling them in the churches, we have two churches here and also for us in the Mosque we tell them every Friday”.

M: How are you sending the message out to Ebola?

R:”After I have given the sermon, because we have two sermon in the Friday prayers, I will tell them on second sermon that sickness is part of humanity and the prophet has reported on similar sicknesses. Prophet Muhammad ”salala wai le wa salam” ( peace and blessing be with him) he was having people that were sick with leprosy, during that time he was not allowing the people with leprosy to join the “jamat”(congregation), they prepared special place for them. I was telling them that, it is not that when a person dies and they do not wash the person, will stop the person from going to heaven, what you were doing before you die will take you to heaven is not when you are washed and the people believed that, so when a person’s die they should not touch the dead body until the burial team comes”.

M: What of all those messages you have heard, which one you think is the best?

R:” the best is, when somebody is sick and showing the symptoms of Ebola or any other sickness, it has not be confirmed, don’t touch that person, be giving the person “ORS” (oral rehydration salt) until they call 117 to come and take the person”.

M: What of all the Ebola messages you heard which one you think that have not worked so well?

R:”Well to me with all the Ebola message, that I have seen and heard, don’t touch, don’t attend burials, they have not worked

M: why has it not worked?

R: It is simple to say but to act on it is very difficult.

M: Is there any way you can think of that could help people to accept the don’t touch, and to attend burials?

R: that is what we are doing by preaching to them to stop, but it is difficult for people to accept at once, but I think with the preaching we are making progress, in the church and mosque

M:”What do you think would be the good message to encourage people to bring patients to treatment centre, holding centre, community care centres?

R:” Well the problem was, they were telling the people that Ebola don’t have medicine and you will die when you get Ebola ,so they prefer to stay home and die when they sick, but now when they are seeing survivors from Ebola, so everyone has aware that when you are sick you should go to the hospital”.

M:” so which message do you think will encourage the people to go to the treatment centre?

R:”The message more is to empower the religious leaders to continue”.

M: Which type of religion?

R:” the Muslims believes their Imam and the Christian believe their pastor, what they tell them they will believe, so let them take this messages to the ones people believes the more”.

*(A voice of a baby)*

M: The way people in this community our viewing things, in the event of Ebola infection, where do you think the people would prefer first to go?

R:”For us here when someone is sick, the person will straight away look for someone that have phone if the person do not have, and say I am sick, I don’t want to laid down at home, let them take me to the holding centre, because we have holding centre at (--name of larger town in the same chiefdom as the interview village with the highest level of PHU – a CHC --)”.

M: So (-- same name as above --)?

R:”Yes”.

*(A phone about to ring)*

M: Will they first go to the holding centre? The holding centre or to the traditional healers which one they will first go?

R:”No, No, we were having people here that are doing things like that, but we are watching them, if you have strangers we must have to know why is the person here and we are even not allowing strangers here and no “native” (traditional healers) should work at this moment, everybody should go to the treatment centre, if it is not Ebola but other sicknesses for “native”(traditional healers), when we see the test result paper (with a negative Ebola test) and the paramount chief also sees it, then you will sent to a “native”(traditional healer) but without that you should go to the treatment centre”.

M: If not for the laws that the paramount chief have passed, in their own thinking, where you think they will prefer to go?

R:” When they are sick, where they should go, they will go to the herbalist and say let them go and “look in gron” (look into their future), because we here, everybody believe that they are “fankay” (fire with witch gun) him/ her, because here there is no other sickness but “fankay”(fired with witch gun), when some feel headache the person will say “fankay(fired with witch gun), when the person catches fever is also “fankay(fired with witch gun), as the person get sick, person will go to “morayman”(sorcerer ) to make bottle for protection and rub other things on the person’s body for the same protection because the person said is “Fankay”(witch)”.

M: Why do people think so?

R:”People really believes this here and they take it as guaranteed, they do not that other sickness exist and even kills someone”.

M: Some people stay at home when they think they may have Ebola?

R:”Yes, they will stay there and say the sickness they are having is not Ebola”,

M: Why Would they think so?

R:”They don’t believe Ebola exists and it is real”.

M: They don’t believe?

R:”Yes”.

M: What do you do to your own common sense make the people to stay home when they are sick, and what will encourage them to come to the treatment centre?

R:”The number one I think if it happens in my compound if so someone is sick me as an elder or my wife as an elder, we should be watching on each and every one that will go out today and did not see the person, tomorrow again the same time, we will ask what has happen with the person, if the person says he/she is sick, will we tell the person to go to hospital this is not time for laying down when you are sick, we will encourage the person to go to the hospital and he is going to be cured”.

M: What do you think would be the best channel to pass on Ebola messages in this community so that everybody will get the message

R:”Well the best way that happens here which make people to believe, like how I was just telling you, Ebola people were denying and even Ebola messages was brought here through FOCUS1000 an NGO came and train the Imams, Pastors, after they came with the message, now nobody is attending burial again, because we are watching them, when anyone about to leave the town, we will ask them where are you going, if they said they going to attend burials, we will say no, you are not going, this what they told in church, we will work as one, if the pastor meet you”.

M: Which way they will be passing these messages?

R:”let them pass the message, through IMAMS and PASTORS, these are the most trusted in any community , the Muslims and the Christians will get it clearly, because the first way which government passed the message did not go through, like for me the minister since he left here after we voted for him, up to now he has not come, but when they trained the Imam and Pastors they are really passing the message, the people from one place to the other to pass on the message, so this is the only way to pass message that everybody will believe”.

M: Have you ever heard of people talking in either good or bad way about the Ebola ambulance?

R:”Yes, they said Ebola ambulance move with high speed, so that affect people and die, even our own Brother that was sick here, he was having stomach pain, but they thought it was Ebola, they travel with him from here to Kailahun (first district with Ebola cases in Sierra Leone) and they test him, it was not Ebola but , he say that makes him live but he said the type of high speed the driver were running with that led to the death of five patients, only him survives because he was stronger at that time, that is why we are encouraging to going to the treatment, don’t allow the sickness to become worst, because if it is worst, the speed and gallops of the ambulance will kill you on the way”.

M: If I am getting right, it is just the speed?

R:” No, they said they sprayed chlorine inside the ambulance that really disturbs and was killing people the more, it is sprayed all over the vehicle”.

M: Where they saying any good thing about them?

R:” No, they were not saying any good things, they always run with high speed, they drink alcohol and whenever they drink when they are running in high speed, even when they talk to them to reduce the speed they will not listen.as we were told by the man that went and return, that at any village they go by, they will stop there and buy rum, stout (type of beer) and drink, after they have drunk, they will just run with high speed, they will be not listen to any patient, when you say driver slow down, they just keep quiet and move with high speed

M: How are people talking about the holding centres?

R:”OO, they are talking fine about them, those that have gone there and returned, they said they are holding the patient fine, they eat three times a day, but when you are sick and go there earlier you would be cured that they always say”.

M: What did they talk about the treatment centre?

R:”Before when everybody was not aware, the doctors were afraid, when they take you there, you will be laying unattended there then you die. So when everybody has become aware that this is the way they treat someone, people are getting relief”.

M: So what were they saying before about the treatment centre?

R:”They said when you are admitted there, they don’t care about you, when you reach, they will just put you on the bed pushed you, they wouldn’t care even if you hits, everybody was afraid to touch fearing not to be infected and die. They were not treating people fine”.

*(Voice of people at the background)*

M: What about now?

R:” If even we were not told, but how we are seeing the survivors, we believe that they are treating people fine”.

M: What about the Community care centres, how are they talking about them?

R:”They are not admitting people there, all of us has believe and we have spread the message all over the chiefdom, this person will move from one place to other just to pass the message. So we not seeing any sick person admitted there, unless other sickness, but they will still treat you cure you at the treatment centre we were having at (--name of larger town in the same chiefdom as the interview village with the highest level of PHU – a CHC --).”.

M: So what about the burial team, what do you hear about them?

R:” the burial when Red Cross has taken over burial team, the time they came to bury our brother, and they did not buried him fine at all we were not happy”.

M: What makes you not to happy?

R:”Because our brother died in the hospital, they give us that favour to bring and they told us to deep the grave (dig the grave) and we did that before they came and they just put the body in grave they said they are not covering the grave”.

M: Who said that?

R:”The Red Cross burial team that came, so we become “tawa” (defiant) over their decision, we said you have gloves and chlorine so you are supposed to cover the grave not us and they said no they were not trained like that. So since that time they left, so we went and put the case across to the paramount chief and we told the paramount chief: let that particular team don’t come here again, but after, all the others that are coming now, are doing the burial properly and fine, they will cover the grave, they will allow the people to pray the dead person at a distance if the person was a Muslim and also they ask us to provide “casankay” (a white clothing used to wrap the dead) so them, we tell God thanks but for the first ones that do not treat our man fine”.

M: Have you heard or seen any community that do secret burials?

R:”We heard of it in this of our area, like (--name of a village in same area as the interview village--) when they were doing it, they said its witch gun or witch plane, so “boku”(plenty) of them die like in the (-- name of another area in the same district as the interview village---) area, there is a village called (--name of village--), even my friends that we were riding bike together died “boku”(plenty) because they were denying, when they told them not to load a sick person, they were still loading sick people on their bikes”.

M: Where they doing secret burials?

R:”Well yes, the way we see they were dying, that tells us that they were doing secret burial and that was the reason they are having plenty sick cases, but since they get an eye on them, we tell God thanks, Everybody has the believe that when someone dies they have to call the burial team, they should not do it themselves”.

M: How is the 117 phone line, what are they talking about them?

R:”We here,117 when we’re calling them they don’t act quickly, but in that case, I will fuel my bike, me and the youth leader we go and report at (--name of larger town in the same chiefdom as the interview village with the highest level of PHU – a CHC –), so they will call them, but when we call them the phone will ring nobody will pick the call, at time when they pick, they will just say: “we are coming” and they will not come unless we fuel the bike and go with the report at the holding centre”.

*(A long silence)(Fowl crowing)*

M: Any aspects of that of the existing health facilities/staffs that is working on Ebola care and the treatment centre what were people talking about them?

R:”Some people were saying the staffs working at this holding centre, they do not know anything, it just because they have put a lot of money business that is why they are working as there is no work in Sierra Leone so when any work comes people will do it, and they are even not treating people the way the people wants to be treated so that is the problem”.

*(A sound of a paper shaking)*

M: In this community how are people treating the Ebola Survivors?

R:”We have one, and we are holding and appreciating the person fine, we thank God the person is a child, so we will not worry that the person is going to do “mama and daddy biznes” (sexual intercourse). So we are holding that person fine and we always welcome the person”.

M: Are they not doing something to the person that shows that they do not accept the person?

R:”No everywhere the person goes, the person is welcome, we will not drive the person away or that about the person that “you were sick and you cured, move from here” - no we will not accept him, we will always encourage the person”.

M: Have you heard of any new treatment on Ebola that may become in Sierra Leone very soon?

R:”Yes, I heard of a vaccine which they say they are coming with next month to come and use it for Ebola”.

M: What do you think about this vaccine?

R:”If they have approve it and see that it works, let them come with it before our people continue this sufferings, because most of the traditional people are the people that have left with this sickness because of the ceremonies who have in Sierra Leone, when someone dies, they will pass all the ceremonies first before they call 117. But if they give everybody the medicines we will be happy and free from that”.

M: What concerns do other people raise when they heard of the coming of this vaccine?

R:”Some people say they are not taking it, even when they were distributing the soap people refused to take the soap, unless we will talk to them to take the soap, we told them “this soap is not going kill you”. But if it is a different person that came with the soap, they will hold the soap wrap it and throw it, they said government wants to kill them, so we will tell them Government is not doing that, if they kill you, they will not have any people to rule. So the government will not do that. So let us use this soap to prevent us from Ebola, and they later took it”.

M: Have you heard of any new ways to prevent Ebola?

R:”Unless those precautions, that we should not attend burials, we should not touch dead bodies, don’t touch sick person, as they have freed the road to travel, when someone comes, let us know where the person is from, and where should the person lodge, we have to keep the person only in a special place and observe the person for twenty-one days. I think that is the best way”.

M: Do you know the way they treat, the treatment they give people when they go?

R:”No, I don’t know, and they have not told me that this is the medicine they give and this is the way they treat, they did not tell me”.

M: As an Imam what are the questions people ask you about Ebola?

R:”They were asking me that, Imam, they have told us not to wash dead bodies and how do we wash a “Janaba” (holy bathing), because some people before they die, they will disgrace, will they go to heaven?

M: what is JANABA?

R:”Janaba means to wash someone when they have died, and we were they telling them yes, a person will go to heaven, even when the Prophet Muhammad “sala wa le wa salam”(peace and blessing be upon be with him) uncle’s died, they do not wash him, they buried him like that, and we have to follow that, and there is a confidence that anyone they did not, hence the person did good, the person will go to heaven. That was the people’s concern, that if they don’t wash someone when he dies how will that person go to heaven”.

M: Do you have any other specific thing about Ebola that you think people need to understand better about Ebola?

R:”Yes sir, for me what I have and I want people to understand, when a sickness has come, is not only us, God has tested, God has done it to all our past prophets and people, so if God has given us our own test, let us all believe, when they said don’t touch, don’t touch, if we do that, we will live long than Ebola finish us in sierra Leone”.

M: What is best way to explain to your people?

R:”The best I will explain, to my people, let them don’t attend any activities, let them don’t attend burials, that is what usually i tell them, if our uncle dies, let sit down and wait let don’t go there, when Ebola is finish in Sierra Leone, we will do the burials but for now let leave it to the burial team to bury. Even one of my uncle died at the Bank when he went to collect money he fell down and died, he was buried by the burial team, until now I have not gone there”.

M: What will be your last message to the people to understand better?

R:” My last word is, my people of Sierra Leone lets us stop denying and let us believe that Ebola exists and it is real, it is a punishment from God, and if we believe God, he will the save us from this punishment, it was not started with us”.

M: Thank you very much Imam for given me this opportunity to talk to you

R:”Thank your sir”.

M:” Thank you very much.
