# Supplementary material for: Development of a set of community-informed Ebola messages for Sierra Leone
Source: PLoS Negl Trop Dis. 2017 Aug 7;11(8):e0005742. doi: 10.1371/journal.pntd.0005742 (PMC5560759; doi:10.1371/journal.pntd.0005742)
Supplement: S1 Appendix — (ZIP) [file pntd.0005742.s001.zip › Ebola messages - FGD and interview transcripts/R2HC Ebola Fieldwork 1/R2HC Ebola F1 COM-Rural5 V2 CORR.docx]

| CODE | **R2HC Ebola F1 COM-Rural5 V2 CORR (rural semi-structured interview with community leader)**  **V2 – 11^th^ March 2015 – correction personal data respondent** |
| --- | --- |
| DATE | January 2015 |
| DURATION | 28 |
| Collector nr | 4 |
| LANGUAGE INTERVIEW | Krio |

**PERSONAL DATA RESPONDENT**

| Age *(in whole years)* | 29 |
| --- | --- |
| Sex (F = Female, M= Male) | Male |
| Religion | Muslim |
| How much time does it take you to walk from your house to the nearest PHU? (minutes) | 120 |
| Mother tongue: | Limba |
| Education level: | Primary |
| Role in community: | Youth Leader |
| Do you know anybody who had Ebola? | Yes |
| If Yes, what is your relation to that person? | Family |

**TRANSCRIPT: (M= Moderator, R= Respondent)**

*(Voice of a goat)*

M: When did you hear about Ebola for the first time?

R:”Well I started of Ebola from 2014”.

M: Which Month?

R:”In the month of August”.

M: So when you heard Ebola how was it described to you?

R: During the time I heard of this Ebola, at that time we were not knowing what was this Ebola, because it was far from us, they were trying to describe the Ebola that if it catches you, it is a virus that enters your body and when it enters your body, it will affect you by “Pulling” (hosing) blood from your eyes, nose and all parts of your body”.

M: Ok, when they told you this, what did you think personally about Ebola?

R:”Well the first thing that came into my mind, it came that “Ah”, and how can we fight this sick, when it “pulls”(hose) blood from all over the Mortal man’s body”.

M:”uhum” (*showing sorrow*)

R:”So I found it difficult “.

M: In this community, in what ways has Ebola affected you?

R:”Well the way Ebola has affected us, one, the time they quarantined that house, where those people died and they found them positive, all our works spoiled and business was not ongoing and nothing was ongoing, so we were just sitting down in the village intact looking after this particular sick not to extend in the village”.

M: Ok, what is reason for Ebola to spread throughout Sierra Leone?

R:”Well the reason is the denial, there was not much believe with the people”.

M:”uhum, ok?

R:”Because when a person denies, we were not thinking that a doctor will die, which takes care of a person when sick, so if the doctor dies, what about you that does not know how to handle a needle, will believe”.

M: Ok, apart from the denial, what again?

R:”Well the measures that the medical people gave us, people were not taking it, in measures, until later now”.

M: which measures, I want you to talk clear?

R:”They were giving us advices, that we should avoid body contact, try to wash your hands with soap and water, not to attend burials, we don’t need to wash dead bodies, when a person is sick we have to isolate the person, call medical team 117, then they will come and collect the body or the sick person to the holding centre, they will take the person’s sample, after taking the sample and proven positive then they will take the person to the treatment centre”.

M: Were people doing all what you said?

R:”Yes”.

M: Ok, What do you think is the best way to stop Ebola from spreading?

R:” The best way to stop Ebola, let still listen to the advices of the medical people “.

M: Like which advice?

R:”Very good, like to wash our hands with soap and water, let’s don’t allow to play with sick person, thirdly, let’s still stop interacting, touching of each other, because they are telling us that even you sweat, when you sweat and touch a person, you will transfer the virus”.

M: Ok, what is the best way to treat a person with Ebola?

R:”The best way, when you see the sign and symptoms, you find the medical area, we send the person to either a holding centre or a treatment centre “.

M:”So in this your community, do you have any local terms used to describe Ebola?

R:”Yes, well it is similar to the way we call it in Limba “Abole”

M:” What is this “Abole”?

R:”What is “abole”, is the same as that particular virus “Ebola”

M: I want you to explain to me why do you call it that way?

R:” this is the same problem I was trying to say to you Ebola, some mammy (……………..) do not know how call the word, but some of us call it Ebola and we don’t know, they said Ebola is a virus and some grandmother that don’t know how to talk the grammar, they call “abole, abola, ibola” are you getting me, but some of us call it Ebola”.

M: Some people don’t believe Ebola exist, do you have those people in this your community?

R:”No, No, because why, if I should have known about that, we should have taken action”.

M: Can you give me some examples of Ebola you have heard, seen or read?

R:”Ebola message,they are giving us messages”.

M:” Like which message?

R:”Like how I said first, when someone is sick, the person feels the body warm (has fever, high body temperature), then the eyes become red, let’s take the person to the treatment centre, let them do medical check-up”.

M: What do you think of the way of disseminating this message?

R”I am thinking that is a good advice they are giving, because the life is very importance, somebody that plays with life, tells you that don’t do this and that will your life will not strain, if you work on those measure, God will help”.

M: What do you think has been the best message you have come across to date?

R:”The best message is, I am not to touch body, I don’t need to wash body

M: Why do you think is the best message?

R:”Why do I think is the best, because they told us, the virus will not live in a dead blood, when someone dies all the “Tumbo” (virus) will come up and when you touch that body, the virus will transfers to you, that why I take it as the best”.

M:”Are there any Ebola messages that you think have not worked so well at all?

R:”Well am not sure if those messages are not working, because all those measures are advices, who takes the advice may live long”.

M: What do you think would be a good message to encourage people to bring patients, sick people to the hospital?

R:”That side we are all working on it, even we are sending messages, who has a person that is sick at the house, that person should not encourage that at the house, don’t touch the person, call 117 or you call the “CHO” (COMMUNITY HEALTH OFFICER) at the holding centre to call ambulance to come and take the person”.

M: What will be the message that will encourage and give confidence to the person/people that have the sick patient to come to the hospital?

R:”I will go and meet the person and encourage the person that: - “do I beg (please I beg you), I don’t want you to be discouraged, this is a sickness that has come to you, you don’t know whether it is Ebola or not Ebola, so do, go to the hospital and do test please, before you sit down (……………..), we don’t know the problem, don’t lay down here maybe it is the same sickness, but if you go there now and they prove that it not the sickness, we will accommodates you, but if you go there they prove that it is the sickness, they will take to the treatment centre and give you medicines, because they said when you go earlier you will be able to survive and there is time for you to survive, you see it”-.

M: In case of Ebola infection, do you think people will prefer to go first to a traditional healer, to existing health facilities or the Ebola treatment centre?

R:”first, the person needs to go to the holding centre

M: Is that the first thing people will remember in this Community?

R:”Yes”. Holding centres”.

M: What about the Ebola centres they have opened?

R:”Is the Ebola centres that came up with the holding centres, at the holding centre they will take your blood sample and send it for test and when the sample results comes back, they have to take the person to the treatment centre”.

M: Why do you think, the people may prefer to go to the holding centre first?

R:”Because the person’s life is at risk and doesn’t know the particular sickness that he/she is having”.

M: Some people stay at home when they think they may have Ebola, why do you think they do so?

R:”Well they alone know why they think so, I know that when they build hospital or centre is for “well bodi” (good health) when you feel your body, you have to go there”.

M: But you don’t know some people if even they are feeling they body, they will prefer to stay home?

R:”If that is happening I have not yet seen it in this of my community”.

M: What you think would be the best channel to get your new message to people quickly?

R:”The way to give message for Ebola to stop in this country quick, well as how they are telling us, like how two of us are talking, you are preventing yourself and I am preventing myself, So tell the person that do not understand, give the person the message that, this messages they are giving us, let’s carry on with it and see what God will do for us, like this “sick business”, burials, when a person dies, not to touch the dead person, if we are able to do that, Ebola will finally leave us”.

M: What I want to know is the channel, when you talk will it reach the people quickly?

R:”The channel now like we are trying to say, the channel we are going to use, for the people to get the message faster, like how you have started doing, giving us the message and we will pass on the message to the people, because most of these messages have met us and we have put in place measures, and we are doing it”.

M: I want you to tell me the good things people talking about the ambulance service?

R:”The good thing”?

M:”uhum” (yes)?

R:”I have not met were ambulance is taking a person, but according to the way people are grumbling, saying that when they load you in the ambulance they will not check- off”.

M: What do you mean by check-off?

R:”The speed, when you load a person that is not well and the person do not have anybody near him/her, then started running in a full speed, you don’t check for gallop how do you think the person will be”?

M: That is the bad aspect?

R:”Yes sir”.

M: Do you have any good about the ambulance service?

R: Well the good thing about the ambulance service, it will stop the sick from spreading, because they will not load a sick person on “okada” (motor bike) when the ambulance have come and collect the person and go, that is the good thing”.

M: What about the holding centres they open for Ebola patients, do you have any good thing about?

R:”Well, since am not working at the holding centre, that is what I will say”.

M: But have you not heard anything about then?

R:”No, I have not heard of any bad things about them”.

M: So about the treatment centres?

R:”The treatment centre again, I have not heard of anything bad about them, they are taking good care of patients, even one survivor boy, told us”.

M: what did he say?

R:”That when they went with him there, he was eating three times a day, they took care of him fine till he left there and came”.

M: What about the burial teams that the government has assigned to be doing burials, have you heard any good thing about them?

R:”The burial team, because I have witness how they bury, they say, because we have people that tells us first, but that when they come, if it is here the person dies, they will come with bag, put the dead body inside, then they will tell the community to prepare a grave, after the community people finish preparing the grave, they will come and dress the person, put the person in the bag, take the person to the cemetery, place the person in the hole and allow the community to cover”.

M: Uhum, but you don’t have any good thing to tell me about them?

R:”Well the good thing, they are doing well, because if someone has died, I don’t know if it is Ebola or not Ebola, I have taken the person for burial, then I will be affected, but the best thing is that, even when they risk they take come is one of the good thing the good things for us”.

M: In this your community, people do secret burial here?

R:”No”.

M: Even the secret society people they don’t bury them secretly?

R:”No, even two society men die inside here”.

M: How did they bury them?

R:”The one was bury by the Ebola burial team, and the other one did not die here, he died at the treatment centre, they did not even come with his body here”.

M: What about the 117 phone line, is there any good people talk about them?

R:”The good thing about 117, they have a good role they are playing, one they are spoiling their work and they are wasting their time, because if you listen they called you at once, then you come, you take that moves and come, is a good thing for us, because if they not measures, they should have gone on their own business, but because themselves have interest in us and people in this country, they love their country, as they call them, they will move, that is a good thing to us”.

M: So you have not heard any bad thing that people talk about them?

R:”At all”.

M: So what about the existing health centres and their staffs working there like the nurses and doctors, do you have any good thing or bad things people talk of them?

R:”Well the good thing I have about them, I have never heard that a patient have gone there and they have treated the persons badly”.

M: So what about the staffs working Ebola treatment centres have you not heard of good talking for them?

R:”They are talking about them good, because they are taking good care of the patients”.

M: What about the bad things; are they not talking bad things about them?

R:”Like how we first started, when this sickness came, took us unaware, people were not in any known about the sickness, at that time, people were grumbling that they were not taking proper care of patients, but we did not blamed them, at that time, there was no awareness”.

M: In this community, how do people react to Ebola Survivors?

R:”ooh, that side I praise God, we are accommodating them fine, even the boy which I told you about, when he came, we went to the paramount chief, then the paramount chief passed his own order, and we accommodated him and be with him?

M: Like which kind of order did the paramount chief pass?

R:”He said that nobody should provoke that “pekin” (child), let us hold him, as how we were being before”.

M: uhummm Ok

R:”Yes sir”

M: Have you heard of any new treatment for Ebola they may become available soon?

R:”uhuum, I heard of a treatment coming for Ebola, but it is a “Marklate” (vaccine), they told me about the “Marklate” (vaccines)

M: What did you think in your mind of this vaccines?

R:”If they come with this “marklate” (vaccine), I be the first person that will be ready to take the vaccine, I don’t have any thinking in mind about it, because I know that before it reaches here, one or two people have used it”.

M: Ok?

R:”but if nothing did happen to them, I don’t think it may happen to me also”.

M: So will other people have thinking in their mind for this Ebola vaccines?

R:”It will happen, they will think”.

M: Like what would they think?

R:”They will think that, after the vaccines may have come, other things may arise”.

M:” Like which other thing?

R:”The Ebola, like to me as how I told you just now, I don’t think a person will get that intentions, like government will pull out something to come and finish all of its people”.

M: Have you heard of any new way to prevent Ebola?

R:”Apart from the old measures, I have not got any other one, the old measures as how I was just talking here, to wash hands, body, not to touch sick person, I have not got any new ones yet”.

M: So you have spoken about the vaccine that it is coming?

R:”Yes, I have heard of it”.

M: So as a youth leader in this community, what are the common questions asked by your colleague youths?

R:” Well the questions they come and ask, “chairman what are we going to do?”, especially the time they came and quarantined houses in this village, “chairman what are we going to do?”, I tell them, “you know what are we going to do, let’s tighten the security in this village”

M: What do you mean by security?

R:”those people they have quarantined they have their farms and there are thieves passing, so I told them to be patient to secure their properties”.

M: Before they quarantined those houses, you know there has been Ebola before, and which questions were they asking you?

R:”Well any way let me don’t lie to you, because we heard of the sickness, but if you are not faced with it, I don’t think that will come to you, because we are busy, in the morning we go into the bush, as you see they are just gathering us; we were in the bush, so I don’t, we gather together, discussed things and asked questions”.

M: What do you feel you need to know to enable you respond to more questions effectively?

R:”What I need to know”.

M: Yes?

R:”What I want to know, for now, I want to know if this Ebola will end this year 2015”.

M: Ok, apart from your own knowing, is there anything specific about Ebola that you think people need to understand better I this community?

R:”Well they need to know better, is these prevention measures”.

M: Which ones?

R:” like how I was just trying to say, let’s wash hands, let us don’t touch body, and let us don’t keep sick people, I know if these messages go to them, but I know it has reached to the people, because now there is awareness”.

M: what do think is the best way to explain this measures better to their understanding?

R:”How I think of the best way”?

M: Yes?

R:”I have to call them, like in this “barry”(local hall), let them space themselves as they do now, and give them the encouragingly, let nobody be discouraged, this sickness came and took us unawares but is the saying of God, but anything God have marked in this “Dunyia”(world) will not pass, so if this sick has come so let’s take those measures, God will help us”.

M: So I say thanks to you for patiently talking to me and answering my questions, I don’t have anything specific to give, but the information given, we look in it and be good things for me and you.
