# Supplementary material for: Development of a set of community-informed Ebola messages for Sierra Leone
Source: PLoS Negl Trop Dis. 2017 Aug 7;11(8):e0005742. doi: 10.1371/journal.pntd.0005742 (PMC5560759; doi:10.1371/journal.pntd.0005742)
Supplement: S1 Appendix — (ZIP) [file pntd.0005742.s001.zip › Ebola messages - FGD and interview transcripts/R2HC Ebola Fieldwork 1/R2HC Ebola F1 COM-Trad1.docx]

| CODE | **R2HC Ebola F1 COM-Trad1 (semi-structured interview with traditional healer in / linked to interview community)** |
| --- | --- |
| DATE | February 2015 |
| DURATION (minutes) | 60 |
| Collector nr | 1 |
| LANGUAGE INTERVIEW | Krio |

**PERSONAL DATA RESPONDENT**

| Age *(in whole years)* | 53 |
| --- | --- |
| Sex (F = Female, M= Male) | Male |
| Religion | Muslim |
| How much time does it take you to walk from your house to the nearest PHU? (minutes) | 15 |
| Mother tongue: | Temne |
| Education level: | Arabic |
| Role in community: | Traditional healer |
| Do you know anybody who had Ebola? | yes |
| If Yes, what is your relation to that person? | Neighbour |

**TRANSCRIPT:**

M: Yes Pa, when did you first hear about Ebola?

R: “The time I heard about Ebola, it has taken time now”.

M: Will you be able to know if it was this year or last year?

R: “Well, let me say this year”.

M: Is the time you heard about Ebola?

R: “Yes, it has taken over seven months, eemmm eight months now”.

M: Eight months now, when you heard of Ebola?

R: “Yes”.

M: How did they tell you about this sickness?

R: “The way I heard of this Ebola and the way the sickness affects, they said a person experiences vomiting, frequent stooling, stomach pain and all the joints got weak, you understand”.

M: Yes sir?

R: “and then head ache”.

M: What came into your mind when you heard of the symptoms of this sickness?

R: (*a vehicle passed with heavy noise*) “Well, what came into my mind, I said Ebola is real, because the ones who are affected and the symptoms I have seen, there was a time, one woman was sick with this kind of sickness and she vomited and when she vomits, she has a child who is a woman. The time she died, so the child held her, as the people entered into the room, they told her leave that mammy she is dead but the child do not know that the sickness is transferable. When the mammy was laid to rest, it took three days, the child was also infected, within two to three days, she died and the person that was living with her in the same house, which gave water to drink, was also infected and died the same day”.

M: Ok, Allahubakaar(God is great), in which ways Ebola has affected your community?

R: “Well through by washing dead bodies”.

M: The Ebola has affected your community?

R: “Yes”.

M: Which ways Ebola has affected you here?

R: “Well the ways it affected us”.

M: Yes?

R: “We have lost the education, hmmm two, the farming did not continue again, the traders are not functioning”.

M: Have you personally seen or known a person who has had Ebola?

R: “Yes, due to the symptoms which they told me I have seen with my eyes, when it affects a person, vomiting, stooling”.

M: What do you think Ebola has spread throughout Sierra Leone?

R: “Well, it about too much of denial, two - the way people move from one place to the other, then- touching”.

M: Ok, What is the best way to prevent Ebola from spreading?

R: “Well the only way I viewed, is to obey the law they told us”.

M: Like which ones?

R: “Avoid people’s compound, desist from washing dead bodies”.

M: Ok, What do you think is the best to treat somebody with Ebola?

R: “Well the only way is to take the person to the health centre, where they cured”. (*Vehicle passing*)

M: That is the best way?

R: “Yes”.

M: To your own way?

R: “Yes”.

M: Are there any local names that people use to describe Ebola?

R: “Yes “kroobola”.

M: They called it as what?

R: “kroobola”.

M: Is it a Temne word?

R: “Yes”.

M: What it is the meaning of that “kroobola”?

R: “Well, the sickness that affects you, the kind of way it makes [you]”.

M: In which way does it makes you?

R: “Vomiting”.

M: it makes you vomits that is the kroobola?

R: “Yes”.

M: So this is a Temne word?

R: “Yes”.

M: Some people do not believe Ebola exists. Do you know people in your community?

R: “Well before this time there were people that do not believe, but at present time now, I have not seen these kind of people”.

M: So you have not seen them?

R: “Yes”.

M: But they were there before this time?

R: “Yes”.

M: But before this time why they do not believe, do you know the reasons for not believing Ebola is real?

R: “Yes because they did not do the test”.

M: Because they did not do the test?

R: “Yes”.

M: That was why they do not believe?

R: “Yes”.

M: Which test?

R: “Well when somebody dies, before the person is buried, they have to test that person, the test results will tell you that this person has Ebola, but they will just come and collect the person then buried without testing and there would be no result”.

M: Who will just come and collect the person?

R: “Well the burial team”.

M: You mean the burial team, without coming with result?

R: “Yes and the ones that are working, the health workers, when they had buried the person, they will just come and quarantined without result, so people are still denying”.

M: Ok, Have you seen Ebola messages?

R: “Yes, plenty”.

M: OK, what do you think of these messages?

R: “Well there were no problem with the messages and people followed the messages”

M: Did people accept the messages?

R: “Yes”.

M: Do people understand the messages?

R: “Yes, because before this time, when the messages had not come, a lot of people died but when they came and sensitized us, the death rate reduced”.

M: Where you satisfied the way they message was passed on?

R: “Yes”.

M: (*moderator coughs*) what all the messages which ones do you like more?

R: “The message I like most, is about doing the testing”.

M: Doing the testing?

R: “Yes”.

M: Which test?

R: “Like the one they did that…………..”(*Long silence*)

M: Which one of the test?

R: “uhmmmmmmmmmmm, I have forgotten”.

M: How does it looks like, do they test the person on the hand?

R: “No, so they make it like”.

M: oooooh, the thermometer gun, which they point?

R: “Yes, that is also fine”.

M: Ok, that is also fine?

R: “Yes they will really confirm that a person does not have Ebola, if the person has, they will say sit down here and wait”.

M: Ok, the gun that they test the temperature of a person?

R: “Yes”.

M: What about the other messages you have seen on posters, do you have any one you like among them?

R: “Yes”.

M: Which ones do you like?

R: “The ones I like most, to avoid those things, you understand, stooling”.

M: Ok, the one you seen someone vomiting?

R: “Stooling”.

M: Ok, someone getting frequent stooling?

R: “kurawkuraw” (rashes of the body).

M: The rashes, ok, but is there any message you do not like?

R: “No, there is no one”.

M: What do you think would be a good message to encourage people to bring patients to the treatment centre?

R: “To talk to them well”.

M: How do you think we have to talk to them? (*Vehicle passing with heavy noise*)

R: “Well they have to put forward examples of the people that have survived, how they went there and came back, let the survivors share their experienced with the people”.

M: In the event of Ebola infection, who do you think the people will go first? *(An undue noise*)

R: “Well, the only thing we will choose one person if there is no way the sickness had pressured the person, we will appoint one person, and the person will used black plastic”.

M: uhum?

R: “If it is medicines, the person will administer it to the patient; then we will call”.

M: who did you call?

R: “The health workers to take the person to the health centre”.

M: What about the traditional healers, they do not go to them?

R: “Well before now, they were going to the traditional healers, but now it has stopped”.

M: Ok, Some people stay at home when they think they may have Ebola, why do you think this is?

R: “Well if somebody sick and did not go to treatment centre, it will create more problems, because the sick gets circulated?

M: But why the person will not go to the treatment centre?

R: “Because before now, when they are taken to the treatment centre, people says, they are not treated fine, so that is the reason they are afraid to go”

M: When they said they are not treated fine, what do they mean?

R: “Well at times, they leave them without giving them medicines until they died and they were sprayed, so when people heard those information, they will be afraid to go, but now it is better, people are eager to go to the centre”.

M: What will be the encouragement to give these people to go to the treatment centre?

R: “Well to they have to “bayor bayor” (pampered) them, cite examples on the people that have survived”.

M: What do you think would be the best channel to get your new Ebola messages to people?

R: “You to greet first”.

M: In this community if you want to pass on information about Ebola, how do you do it, is it by radio or through what?

R: “Well, we will call the chiefs and the chiefs also will call his or her own people in the town and talk to them”. (*An undue noise*)

M: How are people talking about the ambulance service, are they talking in a good way or bad way?

R: “Mmm, some people expressed their dissatisfaction because of the siren that is sound by the ambulance, some people heart stocked (=stopped/halted) because of the way other people are buried, so as they saw the ambulance, they will not feel good”.

M: Apart from this, is there any other dissatisfaction about the ambulance?

R: “Well the ambulance, the way they treat people, when they went for burials, they will just throw the corpse and spray”.

M: What do they spray?

R: “The chlorine”.

M: “Is there any other thing about the ambulance?

R: “No, it is the only thing”.

M: Ok, Is there anything good people talked about the ambulance? (*An undue noise*)

R: “Mmmm, well before now, but now they are treating the people good”

M: Now?

R: “Is better than before”.

M: What do you heard people talked about the burial teams?

R: “Well, the way burial teams treat people is not fine, yes, because when they went for burials, the dresses that was with the dead body, they will remove it and leave it at the house”.

M: Ok?

R: “And they will took the dead body away”.

M: Ok, have you heard of any secret burial?

R: “I heard about it before but now it is not easy again”.

M: How do you heard people talking about the 117?

R: “Well the 117 were creating problem, because some people may not call for the right purpose”.

M: they will not call for right purpose?

R: “uhmm because before now” (*a vehicle passing by*)

M: Why are people not calling 117 for the right purpose?

R: “Because they have called 117 for me”.

M: For you?

R: “Yes, they said I am seriously sick”.

M: You the traditional healer?

R: “Yes”.

M: “They called 117 for you?

R: “Yes, they called for me 117, if you saw people that particular day”.

M: They came?

R: “Yes, they came along with soldiers”.

M: With soldiers?

R: “Yes”.

M: To do what?

R: “They came to collect me; they said I am not well”

M: But where you sick?

R: “No, I was not even there, I went to look for food, my brother called me. When I came, the other day they came and met me sitting down at my house, they were even about to hit the mosque by looking at me, that this was the man that was sick”.

M: What did they do to you?

R: “They just leave me, because they met me writing, so that was the wrong information they got”.

M: Ok, Is there anything good about the 117 phone line?

R: “Well yes”.

M: Like what?

R: “Because when they call them for the right purpose they will meet the sick person”

M: Ok?

R: “Obviously”

M: The health facilities where they take people, how do you hear of the nurses that work there?

R: “The way they talked about the nurses before is not fine but now no problem, even one of my sister, she was sick last month, they took her to the centre, there was no problem, the caring no problem”.

M: what were the bad things they do talk about the nurse?

R: we were told the nurses don’t come closer to them ,and when they want to give food or medicine they have a long tray that they push to them and the patient will collect it and eat, some because of that people were scared ,and thought that the treatment centre is a centre for killing people and not treatment .

M: what might have brought the change of mind from bad to good?

R: people do report, and with the coming of the equipment they wear, they too have now got confidence that if they wear their clothes properly that will not be infected, that is why they have changed.

M: How do people react to Ebola survivors?

R: “When the survivors came, they did not provoke them”.

M: They did not provoke them?

R: “At all, they were well embraced and they talked to them fine”.

M: Have you heard about any new treatment for Ebola? *(An undue noise)*

R: “Well yes, before this time”

M: What did you hear of it?

R: Well they went to the treatment centre, there were no medicines, and they were cured”.

M: But have you heard of any treatment that may become available soon?

R: “No”

M: You did not hear of it?

R: “No”

M: Ok, have you heard of any new ways to prevent Ebola*? (Vehicle passing)*

R: “Yes…….one, to wash your hands with soap”.

M: Ok, have you heard of any vaccines for Ebola that may be coming into the country soon? (*Heavy noise of a truck)*

R: “Well I heard of that talked, I just heard the information”.

M: Ok, like how they were doing for polio and the others, so they came with it again for this, you heard about it?

R: “Yes”

M: What do you think about them, if they bring it?

R: “Some people said they will not take it?

M: Why they said they will not take?

R: “They said, because they are coming to give them the sickness again”.

M: You as traditional healer, what are the common questions people asked you about Ebola?

R: “Well, when they come to me, they will always asked, if I will cure, I said no”?

M: What do you cured?

R: “The sick people, I always say, I am not healing again, because they have stopped us”

M: Did they ask any questions that you may not answered?

R: “Yes”.

M: What are some of these questions?

R: “They always [ask] again, if someone has been fired with witch gun, if I am able to heal that person, I told them no, presently I would not answer that question, they attempted but no way”.

M: Is there anything you think your community people need to understand better about Ebola which they have not?

R: “No, I am not sure”.

M: Ok, Which way do you think is a very good way to talk to your people?

R: “At the mosque”

M: Mosque?

R: “Yes”

M: So there they will talk?

R: “Yes”.

M: Has somebody come to you for healing Ebola?

R: “The person came, but I did not allow”.

M: Do you know why they came to you?

R: “They said let me come and look, what has happened with them”.

M: To look?

R: “Yes”.

M: what has happened with them?

R: “Yes, I do sorcery”.

M: Ok, you do sorcery?

R:”yes, I said no”

M: But do you carry on the sorcery again?

R: “No”.

M: Who are the types of people that come to you?

R: “They are mainly women”.

M: Ok, Women?

R: “Yes”

M: What did they normally come and complained about to you?

R: “At times stomach pain, menstrual disorder”.

M: But before you were treating them?

R: “Yes, before now”.

M: Before now? (*Heavy noise*)

R: “Yes”.

M: I thank you very much for taking your time

R: “oooh”
