# Supplementary material for: Development of a set of community-informed Ebola messages for Sierra Leone
Source: PLoS Negl Trop Dis. 2017 Aug 7;11(8):e0005742. doi: 10.1371/journal.pntd.0005742 (PMC5560759; doi:10.1371/journal.pntd.0005742)
Supplement: S1 Appendix — (ZIP) [file pntd.0005742.s001.zip › Ebola messages - FGD and interview transcripts/R2HC Ebola Fieldwork 1/R2HC Ebola F1 COM-Trad2 V2 CORR.docx]

| CODE | **R2HC Ebola F1 COM-Trad2 V2 CORR(semi-structured interview with traditional healer in the interview community)**  **V2 – 11^th^ March 2015 – correction personal data respondent** |
| --- | --- |
| DATE | February 2015 |
| DURATION (minutes) | 60 |
| Collector nr | 1 |
| LANGUAGE INTERVIEW | KRIO |

**PERSONAL DATA RESPONDENT**

| Age *(in whole years)* | 63 |
| --- | --- |
| Sex (F = Female, M= Male) | M |
| Religion | Muslim |
| How much time does it take you to walk from your house to the nearest PHU? (minutes) | 30 |
| Mother tongue: | Limba |
| Education level: | Arabic |
| Role in community: | Traditional Healer |
| Do you know anybody who had Ebola? | yes |
| If Yes, what is your relation to that person? | friend |

**TRANSCRIPT:**

M: Eh thank you for giving me the opportunity to interview you. We are going start the interview; I just want to know when did you first hear about this Ebola?

R: ‘It was last year in the rainy season”.

M: How did they tell you about this sick?

R: “Automatically when this sickness broke out, we were the first people they called, we the traditional healers who used leaf to cure. They are gathered us and gave us a paper that we should stop curing, that we should stop washing dead body and to stop eating money, and to stop eating those bush meat or anything which has blood we should not eat, if somebody dies in the village let us not wash the body, and also if your brother is far away from your house he should stay away, he should not come to our house again when he comes drive him and tell him to go where he is coming from and that we should not greet each other again so we had to return and call al the towns, all the villages we called them where we held the meeting and told them everything and that was the time the traditional healers who gives out medicines began saying but we want to ask you a question now that you attended the meeting, if somebody is sick and they did not give him or her a medicine which is a leaf, won’t the person die? But I said it is the government that has said we should give medicines but wait a minute, we know about sicknesses that has come to this country; we have had Leprosy, we have had HIV AIDS, government never did that, they can be tired with somebody in the hospital but they can come with the person to the traditional healer and he or she can be cured. But this sickness now they said we should not cure, then we said wait a minute, we can abide by it because Pa Kabba, Sulayman Kabba who was our boss, our overall boss, is the government that gave him those papers so we cannot deny them”.

M: So how you feel when they told you about this? What came to your mind? When they told you that you should not touch dead bodies, you should not cure a person, what happened? What came to your mind?

R: “I just felt that my mind is confused, one if government was ready for us to help them in this fight for this sick, they would have told us, like they did at the hospital those who come to bury they give them hand gloves, they give them, they give them caps, what they put on their faces, what they put on their nostrils, what they put in their mouth, they have the chance to go and attend to the dead body to give it clothes, go and dig grave for the person and bury it, they have the chance. What about us now, if they had given us that advise, that allowance that here is hand gloves, here is what you can have to go and touch the dead body and then they carry us to go and train us like the they did to the Doctors, to let us practice how to give medicine to the sick man, they did not do that for us they waited until the sickness got worst so we do not feel pleased, we are not feeling right all, they would have empowered us just like how they practiced those Doctors, do you understand me Sir?”

M: I do understand you.

R: “But they have blocked us and we do not have anywhere to eat. Is through this medicine that we eat, some people’s foot can die, some their ??? can die, we cure it, they can get high fever they get tired with it at government level and they will phone us here and bring them to us and we go into the bush and take the medicine and give it to the person and before one week the person will be well, we used to get what we eat, but since the time when they stop us we do not have anything to eat again that is how we are suffering. But we cannot blame government they have the power but let them know that if they had trained us like they did to the Doctors, they give us the permission, they give us the gloves like I have seen they give hand gloves, they give chlorine, they give anything that is a uniform, what they wear to attend to the body until they put it into the bag for burial, if they had done that for us maybe we would have come up with a method when we go into the bush to get medicine, we would ask God and the devils when we ask the devils can be able to give us the correct, they can be able to show us the road to pass for the sick, so we can ask God that the devils have said let us pass this way for this sick so as to get answer to this sick ,but we are not allow to do that. If they practice us how to cure and how to hold dead body and how to hold the person who is sick, we have our hand gloves like the once they gave to the Doctors, so at that time we would be able to sit down at night, if I have to buy three packets candle I buy it, I buy incense, if it is one white wrapper I buy it and I first call God, the devils will come standing in line and they ask me why did I call them and I will tell them that this is the trouble in Sierra Leone which we cannot see with our own eyes and that is why I have called you to say we asking you and the government to give us solution and this is what we are following but government did not do it, government just condemned us like that, if they meet you giving medicine here they will jail you so we are now afraid, so all our instruments have been kept, where we used to get we are no longer getting ”

M: Eh, how has Ebola affected this of your community?

R: “The way Ebola has affected us we look sorry”.

M: What do you mean when you said you look sorry?

R: “A sickness attack us which we cannot see with our eye, when a person dies we cannot touch it, we cannot wash dead body and they do not allow us to give medicine the native way that has confused us. it is fine my brother like you have come is nice to grip and hug you but there is no chance now, since you have been here today you and I have not come closer we are far apart, you are my brother like when you have come I will embrace you and laugh all that has been cut off to us we are confused and Doctors have found out for the virus, that virus that comes into your body for that sick, Doctor have done his own until Doctors themselves are dying, we are confused, we look sorry. If government had allowed that let we the traditional herbalist also cure we will ask God and later we ask the devils when we ask the devils they can direct us to tell government that this is how we should go, this sick would not have lasted. That is why when the HIV came luckily the government [played and told us to try from all fronts to cure, some people foot can be rotten and nearly cut, we use all those medicine to cure it. HIV did not take up to one year in our country we were curing it secretly, some person used to come there and come to us we can put him or her somewhere for three days we cannot touch him or her, we just go with medicine for him to drink and wash the person gets well and go back. But we were doing it secretly for government not to know, we cured it and HIV is no longer around .government did not know how we passed, the man met me with the sick and I need money I will cure him to be able to get money to feed my people, to be able to pay school fees for my children, there is where we get. Man like us we are in the bush but we don’t touch hoe, we hope on the leaf. Even when they fire somebody with witch gun when they carry the person they will say is Ebola. When they fire somebody with witch gun and they carry the person Doctor cannot know that he or she has been fired by a witch bullet they will hold him there till he or she die. When one has tuberculosis, government will not know that it is tuberculosis they hold him or her there and he or she will die. One foot will be dead and the hand dead unable to get up and as he or she begins to tremble they say is Ebola they hold him or her there and she dies. All those things is shocking, we look sorry. Those whose feet are dead and is tremble or has been hit by witch gun we can remove the bullet, like you are sitting right now if they fire you with a witch gun bullet and you begin to tremble not able to get up and your body get warm and everywhere get blood and boil on your body, I swear when I go and get that medicine from the bush and give it to you, you drink some, and I put some around you and make like this then you see the thing coming out everybody will see it before you say some hours the person will begin to talk, before they say the whole day the person will begin to work but the Doctor will be turning around going here to sign that book, go there and get this and they delay and when the person dies of Ebola. Many sickness have combined with Ebola, but the government is afraid having seen that the sickness is just passing and you do not see it, the virus to see it is not easy. They come to us that we should not cure again. That is the part we are so hurt , now is hunger that is going to kill us, when I am cure a person, like when you have come this morning when you come with a patient I give him or her medicine and you give me money and I eat what I want to eat. Why has this sicken become rampant this is the reason, they did not call us to teach us how they learn the Doctor so that we can work together “.

M: Have you seen somebody who is an Ebola patient?

R: “Yes, I have seen somebody who is an Ebola patient, but I don’t have mind to go closer to him, if they say this house has Ebola even if it were you, if your mother is there you will not go there again”.

M: What is the reason why you think this Ebola is spreading?

R: “One, some people are saying is a lie is not a sickness and that government is lying to say Ebola is real but we understand that any kind of sickness is on earth. In 1960 there was an outbreak of a sickness they call it in Limba ‘Bomboroso’ ok Temne man call it ‘Kabomoroso’ Krio man call it a powerful sickness which has no medicine but during that time there was no too many laws so they decided to go to those who gives leaves, those traditional healers and they asked them to help. The person who is affected by it nobody touches the person they carry him or her into the bush, and the person who gives him or her the medicine is somebody who has been affected by the sickness once when he makes the medicine he will call people who had been a survivor will be the person to come and give the medicine. Within one week to one month whoever dies there will not be brought to the town again, nobody will touch the person again and they will go and look for an anthill where there are those ants that can bite people and smah all their bones there they will gid and bury the person there it will take one year without anybody entering that bush, so it in 1964 that the sickness was ended. That was the time when another sickness came caleed ‘FETEKPEH”.in Limba”.

M: What does that mean ‘FETEKPEH’?

R: “FETEKPEH? Temne man call it ANKAKA”.

M: ANKAKA?

R: “Yes the white man call it mizzles (=measles), when that sick came it was cured by ‘Omolay’ (alcohol), that alcohol but white men did not get the Marklate (=vaccine) quickly if that sickness fall into this house even if there twenty children there they will all die. So just like this Ebola. So the people sat down, they went to the bush and asked the God, they asked the devil and quickly the medicine acted right away, so it is with this alcohol and the water used by blacksmiths that they use to rub on the person then they use the alcohol and then rub the dust all over the body, and before one week and then take lime and mix it with honey and he can be drinking that. So that house they will hang one lime this way and one lime the other way that sickness will not enter there again until when the white man found out the medicine and they started Marklate (=vaccinating) people. When a woman is pregnant they give her Marklate (=vaccine) and when they give birth to that child it cannot be affected by polio again. So with all those sicknesses, Sierra Leone has got the sense to drive this sickness but everything started with the herbalists. They begin to doubt that we do not have Doctors here but this sickness is not affecting them, then they showed them the tactics and the method they applied and the white man took note and went”.

M: To your sense now, which way do you think is the best way to treat Ebola?

R: “The best what to treat Ebola, you and I have to be united”.

M: What do you mean when you said you and I should be united?

R: “Let us believe that Ebola is real that is one thing. And two let us know that there are medicines that can cure Ebola which is a native one. Let us believe there are native medicine that can cure Ebola. In the first place we must believe that there is a medicine to drive Ebola. We do not ask Doctors, we do not ask those who write with a pen, but we will ask God and ask the devils the devil must direct us who to drive Ebola, they can show us the leaf truly”.

M: But for now can you show me any way which we can use to stop Ebola from spreading?

R: “I tell you thanks, we were all ready to meet government to help them but we are afraid. Like when you are here now do you know I did not go to the farm or run away?

M: No.

R: “Is because you are a black man, if I had seen a white man, who does not know me I will not sit I would have ran away, but since you are black even if I mistake and say anything here, you will not go and prosecute me for them to come and arrest me”.

M: No, no, no, I will never do that.

R: “But the white man he is not my brother, he is not black if I talk anything here which he is not satisfied he is going to take me to government and they will come and handle me so we have all this fear. Since they told us to stop curing. We have seen some of our companion who did not listen they took them and put them into the motor car and carry them”.

M: Oh, those who cure?

R: “Yes after the warning, they see anybody who mounts a pot to cure, I swear they will carry him so we have all this fear. But to say we can get the mind again for us to come together and work to fight Ebola, we are afraid. It is only you who can give us the zeal to do that if you do that we can be behind you. But if you did not give us the zeal and tell us to cure anybody in Sierra Leone, except this government says it”.

M: But to your own common sense, I just want to know you view or perhaps the way any other person, what do you think is the way to drive this Ebola?

R: “We have to contribute, why I said we have to contribute when they give us the chance to go and train, we cannot just contribute without government training us o’.

M: You mean government should training you?

R: “Yes, government should train us to give us zeal that whatever we are doing is done by power but that I am doing it for somebody. If they come and hold me with the paper and disgrace me the government which gave me the paper can fight for me. But we cannot come and grip the work because we think we know the medicine we come and grip it without government knowing”,

M: But which way do you thing is the best way to treat somebody who has Ebola?

R: “If government did not train us and give us the power, when you make the medicine for the person you cannot go close to the person since you do not have hand gloves, you do not have uniform, you do not have what to cover your nose and mouth and your eyes with you wear long trousers and make sure that you have two socks and them you double the trousers and you now have three trousers not so?

M: Yes, Sir.

R: You now have three clothes not so?”

M: Yes Sir.

R: “When you are finished you wear another cloth which stops here and when you are mixing the medicine you mix it with a stick but the stick you just make it like this not for the Ebola it is when we are curing those wonderful sicknesses after making it with the stick if it is for drinking you do not go near to the person you put it down and the person take it, if it for rubbing you put it on the stick and put it down for the person to rub and tell the person not wash for three days, after three days the person can go and wash but we cannot go close to him, you cannot go nearer to the person for the sickness not to transfer to you again, you can come and greeting him in the morning but remember that the clothes you were wearing yesterday should be taken to a particular place like the water side you cannot bring it to town again. That sickness is wonderful and they call it ‘Kabombo’. If it enters this town it will kill everybody but the native medicine have driven it completely”.

M: Do they have any other name apart from the name Ebola?

R: “May God help us?”

M: Amin.

R: “This Ebola has a name, but it is in Limba”.

M: How do you call it?

R: “FURU” and also ‘KUDUTU’

M: ‘KUDUTU’?

R: “KUDUTU’.

M: What does that mean?

R: “KUDUTU?”

M: Yes.

R: “Do you know what Limba man call KUDUTU?”

M: No I don’t know.

R: “Like how we are now they can see you well, every part on you is well you can be sitting and begins to tremble and vomit by the time they think of it if I come and touch you me too will begin to vomit if another comes and touch me he too will begin to vomit, what comes up next is that you begin to sleep no sooner the head goes down you are dead. When you die the person who would come to bury you he too will die. If it is in this area all the people in this area will end up dying. They call it KUDUTU. But when we got to understand those kind of sickness a white man cannot, what the white man knows is to test and when you go and test that sick and when you are affected any hospital they carry you they will not get the medicine but when we go to the bush, we got the medicine, when we get the medicine it is now with us, we can drink it and take the lime, honey we drink it freely, we even take some cut it and put of palm oil and eat it. The sick comes at the start of the rainy season but now before the first rain comes now it will not affect anybody again it will affect the fowls. KUDUTU will never affect human being again it now affect fowls. Why does it affect the fowls, the sickness is air bound, when they air filled the nostrils of the fowl it will not be able to breathe again and it will eventually die, and the other fowl which will come to eat around a again will die, even if there are hundreds of fowls they will all die. But at that time government used to allow us to cure people so it was not difficult. Now it cannot affect human beings forever, for that we say thanks to God now”.

M: Eh, some people do not believe that Ebola is real.

R: “And these are the people who the made the Ebola to stay long”.

M: But do you know of people in this community who do not believe that Ebola is real?

R: “But people have not said it before me, like how we are talking, we have seen a lot who do not believe and they died”.

M: No, they cannot talk it in front of you but there are people who do not believe.

R: “We have seen a lot who do not believe and they died from this even in this town when they say Ebola the do not believe”.

M: Why do they not believe?

R: “When the Ebola is, one woman went and brought her sister and hide her in the bush until she died there, when he died all of them died, the other when they were carrying him he died on the road, the Okada man who load him on the bike also died on the way. So by now everybody should know that this sick is real but you and I should not deny”.

M: But why don’t they believe?

R: “I thank you, let you be my father and let me be your brother. Do you know why people do not believe that it is Ebola, as you are sitting now you are well and they are not seeing you picking here picking there, but the man who knows what is Ebola, they will come and look at you keenly when they come for the test they will just look at and say oh this has Ebola let us go and test him. When they carry that person and he dies there and they did not see him they say oh they have gone to kill that person and they will not be sure that it is the killed him. If they did not see the person’s grave because of lack of understanding, they have to make them understand that they should not bring the body back home for the sickness not to spread here again. As long as they did not see the grave they will it is a lie it is not Ebola. They will think that they threw the person on the way or they had an accident. They would say they took the person away and they did not see his grave. That is why people are afraid and they are saying it is not Ebola”.

M: Can you give me some of the Ebola messages that they have been sending out?

R: “I have heard a lot. Like you, you came normal and asked to say the truth about this thing. But some will come when they have given him his transport to come here, when you tell them to come and treat us gently, they will come with a kind of tension and when the people see them they run away they say the machine has come”.

M: Which machine?

R: “They come here with machine they say they are going to test blood. So they are afraid that when they carry their brother, they carry my sister she did not come back so they are afraid, so when they see the machine they run away”.

M: They came with a machine?

R: “Yes, they say they have come with the machine to kill them, if they go with the person they will not see him or her again”.

M: Which machine is that?

R: “It makes like this ‘papap-papap’.

M: Is it a machine?

R: “No it is not a machine, they just make it like this ‘kpelek’ and draw the blood, so people are all afraid”.

M: So apart from the blood which they draw, have you seen any Ebola messages here?

R: “Yes”.

M: How do you see those messages?

R: “Some people say it is correct”.

M: The messages?

R: “Yes it is correct it saved us”.

M: Which messages that saved you?

R: “One, like they said we should clean our hands, anything you do we do we have to clean our hands, that is one. Two, like me and you even when I know you today you are my brother, but if we were not living together I should not come closer to you again that saved us. And two, a person should not eat those animals in the bush; cutting grass (=grass cutter or cane rat), baboon and monkey, we should not eat them, that also saved us. Then two, if you are ten in a house everybody should have his own bed, we tell thanks to God”.

M: Do people accept that?

R: “Yes, here many of them, since we have to go to the meeting when we come from the meeting we come and hold the meeting and inform them about what the government has said. We should not go nearer to each other except when we go to the Mosque, even that they have warned us not to go closer to ourselves, we should not gather, so we tell them thanks for that it saved us. Since the death of those two men, one over there and one here, one of the child whom they carried away saved and they brought her back”.

M: How many people died here?

R: “They were about; (- - name of Ebola victim 1- -), one, (- - name of Ebola victim 2 - -), two, (- - name of Ebola victim 3 - -) and that boy (- - name of boy survivor - -) who carried but they brought him he is well”.

M: So three people died here?

R: “Yes, three people died here”.

M: The one survived, isn’t it?

R: “Yes, he survived”.

M: Em, among all the messages which one do you like?

R: “The one which I Iike most on this Ebola is the cleanliness, I like that but they are plenty the ones I like”.

M: What do you mean?

R: “By the cleanliness?”

M: Yes.

R: “When we leave here and we want to enter here, we wash our hands”.

M: Ok, that wash hand that one is fine to you?

R: “It is fine to me”.

M: Ok, among those messages which one do you think is fine for you?

R: “Hay, to go and bury my father, bury my brother and nobody did not see his grave that is not fine to me at all. Either let them tell me to go and look how they are going to bury my father, go and look how they are going to bury my brother, how they are going to bury my sister but I did not see grave you just come here and take them away saying it is Ebola. I did not see his grave; I am not satisfied with that. I can be standing from afar to see where they bury and they can tell me is because they do not want the sickness to spread again but me I want them to call me for me to see how they are going to bury him”.

M: To your own think, which message do you think we can tell people to encourage people to come with patients to the Treatment Centre?

R: “One, they should not deal with the white man. If our people see the white man they will be afraid”.

M: What do you mean?

R: “Like when they send somebody to come and learn us, if they are sending somebody to come and teach us about Ebola they should not send a white man”.

M: They should not come with a white man?

R: “No, you are black and I am black, I will know that it is my brother I will not be afraid and two they should not send somebody who carries gun like a soldier, if they see somebody whom they say is coming to check here they will run away”.

M: They should not send a soldier?

R: “Yes if they see a soldier who they say are coming to treat them or to come and look here they will be afraid. The police man, you know that they have too many laws, we will be afraid but if it is you. If he speaks Temne, he hears Limba, he hears Krio as we are exchanging Krio, I become happy, anything you tell me I will take it, I will not be afraid again but when I know that you are black so if you come to ask me I will not be afraid again, I will not run again. When I hear that it is not a police, it is not a soldier is my companion black who does not wear uniform will be coming to interview me. That is why I did not run. But if it were those people coming, we will be afraid because when these people come is the law that they know, they will just fling you into the motor car and go even if you die they will call to say this person is dead but I want you to come closer for us to know how we are going to attend the person in the hospital so that I can see him or her, so when the person dies they can call me to say this person is dead but we want you to come closer we cannot carry him or her to the town and we want to bury him or her here, then I can say ok it is true, I have seen the grave I am satisfied".

M: Ok, eh in the event where Ebola infection, should there be any case about Ebola, who do you think is the first person that people go to ask for help?

R: “The first person whom they go to is that they will ask for the herbalist “.

M: No here should there be any sick person in this community, where do you think the sick person will first go?

R: “The first person they will go to is the Chief and tell him that Chief we thought that this thing was over but we now see that it is coming up again how are going to do? The Chief will then say let us call”.

M: Who are you going to call?

R: “The Doctors”.

M: So they first call the Doctors, what about the Traditional Healers?

R: “Is because government did not give you right to go closer to the dead body, to go closer to the sick, we are trained for it, government did not allow to go and get medicine to cure the person, even though we have the medicine you will not have the mind to go close to the body or the sick. But if the government had gathered us together and train us and give us the uniform, when the person fall sick we will try when we try and cannot be able then we send to you government to say this person is at so, so place”.

M: Why do you think people first go to hospital before coming to you but they go to the treatment Centre?

R: “Well the mind come from us, we have to tell them come nearer to us and maybe government will say”.

M: No the thing is if the government has not trained you, why is it that the people do not come to you but will go straight away to the Treatment Centre?

R: “Well we do not have the document to prove that we have right to cure them”.

M: Ok, is because of the document which you do not have that is why people do not come to you?

R: “Yes is because government has not allowed that we can continue to cure people. So we do not even have the mind to touch the person except he or she has to go”.

M: Ok, some people prefer to stay at home when they have Ebola, why do people like to stay at home when they have Ebola? Why do they not go to the Treatment Centre?

R: “They are afraid”.

M: They are afraid of going to the Treatment Centre?

R: “Maybe he has seen that if he goes there he will not get the right medicine, and this is a sickness when it affect you before one hour you will die, before he or she goes there he or she is dead and they do not believe that Ebola is real”.

M: So that is why they are afraid to go there?

R: “Yes, they are afraid”.

M: So how can we encourage so that they cannot be afraid to go to hospital?

R: “We are here some of us we go to the meetings and they tell us that we should not touch sick body, that anybody who is sick of Ebola we should not near to him but we should tell him or her to find the hospital and when you tell him or her that the machine they are going to use is not anything that will kill you to cure you, that of your brother who died and they did not bring him here was to save you own life because the sick will become rampant in the town that is why they did not bring him to the town but do not think that when they take you, you are not coming back , when they carry you if it is bot the sick they will bring you back here so that we can cure but if it is the sick them they can cure you but if you dies they are going to bury you there. So do not think that when you they are going to treat you bad”.

M: Which way do you think is the best way for us to pass messages to our people?

R: “Well I tell you thinks, like this it is not the first time of coming, what should the government do, they have to give us zeal, call a meeting, sit down with us and tell us all what we are supposed to do and give us paper and tell us what to do to our parents. When I come back I will call them, since government has given me allowance I will call all of them, I will call the town Headman, I will call the Mami Queen, I will call the Youth Leader and I will call all the town leaders and tell them that this is what government has said. But in my own common sense I will not have the mind to tell person to come to me for treatment”.

M: Yeah, well now how do we pass this message, how do we communicate this thing, through radio?

R: “And one, like me sitting here I do not have a phone, my phone is spoilt”.

M: But how do we communicate now through radio stations?

R: “If we do it on the radio station it is poor”.

M: Is poor?

R: “Yes, some do not have radio, some towns it is not everybody who has radio, some people have radio is for music in the villages, like those villages and beyond”.

M: So which way do you think is the best to pass on these messages?

R: “Like me so, I know all the towns, when government gives me the allowance, to do that government will ask me can a Honda (=motorbike) go there I say yes, can a bicycle go there and I say yes, he gives me the bicycle. But then some village roads are narrow any motor able because of streams but if it is a bicycle you can cover six to ten miles even where the road is bad you can take the bicycle and pass and reach places like (- - two names of communities in the area - -), you sit down and call everybody and tell them that you will be coming to give them the paper sent by government to tell them about Ebola and how we can drive”.

M: So the radio cannot do that for us?

R: “No, it can talk but it is not everybody that understands, some cannot hear Krio, even that FM and that Good Morning Salone they cannot understand the time that they start the program so if the put FM they begin to play music , story there is where he or she will listen, he or she will listen from somewhere. But people like us we need all these interest to have radio at home, but since my radio got spoilt I am not working so I don’t have radio. But if it is you when you come we talk one on one and try to put it in an important way not so?“

M: Yes Sir.

R: “So if you have told me to go to (name of 2 villages in the areas), go to (-- another village --), go to (-- another village--), go to (--another village--) go (--another village--), go there go to all these 24 town onto (--another village--) go there for the people to see you, when I go since the people know me because I was born there one, they will not run, like you when you have come here today, they all know you now any time you come here nobody will run again. But if it was an extra person who comes here or the white man or uniform man or a police believe me when you came yesterday today they would not have met anybody, but you are my brother when you have come where ever I see you now I will prepare for you whatever you want to eat, but if we live it to the radio that sickness will not end. We should have people, if they have Honda (=motorbike) let them move with it, if like it is a village where Honda (=motorbike) cannot go but you can use a bicycle. Like me, early in the morning I will ride, I don’t know how to ride a Honda(=motorbike( but I know how to ride a bicycle early in the morning I will ride to go to those villages where a bicycle cannot pass I will lift it and pass and then go to the Chief and tell him that government has sent you and you will be having a meeting for him to call everybody be it a child, be it an old person let them come so that I can tell them about Ebola, they will be and say this is the son of the Chief in (- - name of interview community - -), that herbalist, the one who cure our children, that son of a chief so I can gather them and tell them”.

M: So how do the people feel when they hear about the Ambulance?

R: “It is the Ambulance that killed many”.

M: What do people say about the Ambulance?

R: “It is bad, it is not the Ebola, the Ebola they can carry the person and cure him or her. But do you know what they did with the Ambulance?“

M: No, I don’t know.

R: “That thing which they pump”.

M: Which thing is that?

R: “Chlorine”.

M: Oh, chlorine

R: “Yes, they pump too much of it now, it has a chemical plenty, a person can be inside the Ambulance and you will not be able to breathe again. And where they put the patient has too much gallop”.

M: Oh, the gallops?

R: “Yes, they run at break neck speed, they don’t check, they go on galloping the person like this ‘Gbebeh, gbebeh’ by the time they Ambulance the sick man is already dead, this happened during this sickness. Before they open the Ambulance the person will be dead, if there are ten people by the time they reach believe me five or six would have been dead. Some who did not die when you bring them out they are hope and will not be able to walk again. Except later when they realized that when a person gets sick here before taking him or her away let them test the person in (- - name of headquarter town of the interview district - -) first. Now they say when somebody dies of Ebola let them make a tapolien (= …………..) and bury the person for everybody to see his grave. That is the time people began to realize that the sick is real. It is not the sickness that people are afraid of to enter the Ambulance, it is the gallop and that chlorine which they pump, the power is too much it makes them hopeless so they are afraid to go into the Ambulance, instead even he or she has the sickness he or she will run away. I remember when a woman was sick and they call, said she was really sick but she was afraid of the Ambulance so when the Ambulance came the woman said let the Ambulance be at the front she will follow behind the Ambulance by foot wald, and the woman walk unto the Centre at (- - name of bigger town in the interview chiefdom with a CHC- -) they went and test and they said it was not Ebola they cured her she was there for three months and now she is back, even this morning she greeted me and passed”.

M: She did not agree to enter the Ambulance?

R: “She did not agree, she said the Ambulance has too much speed and that chlorine and that medicine is harsh, she had to walk to (- - name of bigger town in the interview chiefdom with a CHC - -), her name is (- - name of woman - -) in (- - name of her village - -). When she went to hospital the Doctors held her and tested her and said it was chronic fever, she used to drink too much alcohol and they cured her. Now we thank God she is well now and doing her normal work. So she did not agree to sit into the Ambulance at all”.

M: Is there any good thing about the Ambulance?

R: “There is”.

M: Like what?

R: “If they do not have speed, they do not pump that medicine the Ambulance is good. When a person is sick here and there is no transport, there is no money when you call them they come if the person is a pregnant woman they come and collect her quickly do the operation and remove the baby alive. But where the Ambulance discourage us is that of the speed and the hash medicine which they pump that is why they are afraid of the Ambulance”.

M: Which harsh medicine is that?

R: “That chlorine, except when they changed it now they only put five tablets when they mix it if a dead body is here they come and bury they test it before us and we can see that it has no power again so they come with the perpolien (=……) lose it and put the body there and go to bury it quietly. Now we tell God thanks but at first, they did not treat right, the Ambulance killed, we did not feel fine about it. At first they did not understand when they send you inside it the gas is too much and the gallop is heavy”.

M: How do you hear about the Treatment Centres?

R: “The Treatment Centres, sometimes you can meet fine Doctors he will courage you, some others, some of the Doctors they have rooms one of the room is written ‘Condemn Room’ if they carry you there it will be very difficult for you to get well, so they do not like that room. There is the other room if they take you there the Doctor who is there can treat you fine, he will come closer to you and treat you fine. But that room that room where it is written ‘Condemn Room’ if they take you there even if it is your child, your mother, your sister, just begin to cry now, the Doctor who is there does not [en]courage any sick man. The other room, they said there is a big room there all the bads are there and hang all the medicine on them, drips are also there, the Doctor who is there can treat you fine but that room where they wrote ‘Condemn Room’”

M: ‘Condemn Room?

R: “Yes, if they take you there for you to come out now you have to be very lucky”.

M: How about the Burial Team, how do people talk about the Burial Team?

R: “The Burial Team, at first they were bad”.

M: How were they bad?

R: “At first they were not using their hands to take dead body, they put a machine outside there, when the put the machine here it will just go and jug the body and put it they do not give it that bag, they do not dress it, if the person was wearing short trousers when he died if he was not wearing clothes that is how they will carry him. So the people feel sorry, the people cry when they take their people away. It is by the machine not man it will just go and jug it and bring it very sorrowful. So we didn’t like that part of the Burial Team. So at last they said that should not be the case, now they come and test the body and they will dress it they the Burial Team will dress it not us. After dressing it if the person is a Muslim they pray over it, if it is Christian, tyey pray over it and they will take it to the grave side and put it into the hole after that they will tell us to cover it. So on that, they warned the Burial Team to stop and they have stop”.

M: So is secret burial happening here?

R: “Anybody, like in one town when a ppor (=………) person dies they bury him or her secretly”.

M: Why do they do that?

R: “The person who touched him, those who washed the body all of them died”.

M: Why do they bury them secretly?

R: “They are afraid, is the fear”.

M: Afraid of what?

R: “If they call the Ambulance, it is lack of understanding, if they call the Ambulance they will come with the machine to come and jug the person. They sit down until, they will begin to say what kind of sick is this that we cannot see, a sickness that cannot be cured they will just come and say Ebola. But they have never seen it even the grave where they bury the person we do not know. So they will decide to hide from government and bury that person secretly. There is one town here it happened about 61 of them, a whole village nobody did not survive, except the one who did not go near, they have an understanding, but all of them died those who buried secretly".

M: 61?

R: “There is one village here in this (- - name of interview chiefdom - -), it happened to us, I have just forgotten the name of the town when you pass (- - name of bigger town in the interview chiefdom with a CHC- -), all of them died. Not until when government came and warned them, they went to graves and spray medicine there”.

M: Right now is it happening?

R: “No, since that time they put a law that nobody should bury again. Now we tell thanks to God it is normal”.

M: But which type of people do carry out this secret burials?

R: “The people who do not understand that this sick is real, they are the ones. To deny what they write on paper , for a person who did not go to school, like you and I, I only understands the quran (=Koran), but for some people to understand is a problem so they do not believe since they are not seen the person is only the paper that warns them. Like we have seen face to face whatever you tell me I can believe but to say you write it on paper and live it for us to study we will not do it. That is why many of them died”.

M: You told me about Ebola survivors, do you have them here?

R: “Yes we have one here, the boy who survived”.

M: But how do people look at him when he came newly?

R: “When they brought him in the evening they told us they were going come back in the morning. That morning they came and called the entire town that the man was now well and will never sick Ebola again, that we should provoke him, that we should not push him and that we should embrace him and talk to him fine, so I called a meeting and told the people that the boy government said that we should not push him or drive him, and that we should not be calling him that Ebola man, that Ebola man, they said we should not do that. I told them that if anybody does that we will take him or her to government. That was the law we put, we should provoke him, we should not drive him from us”.

M: Since his return has anybody provoked him?

R: “No”.

M: They did not drive it at all?

R: “No, he was here with us playing, he has even started to work now”.

M: “Have you heard about any new treatment for Ebola?

R: “We have not heard about treatment, we just heard that they were going to give medicines”.

M: They said they were going to give medicine?

R: “When they give this medicine you take it one after the other. But for us in (- - name of interview chiefdom - -) Chiefdom and this village we are sitting till date we have not got those medicines. Some of the town in the surrounding have got it, but we tell God thanks”.

M: Which kind of medicine is that?

R: “It is a seed, it is a tablet but they have not given us. But why we tell God thanks”.

M: Hmmm.

R: “We in the first place we took control, we abided by all the laws that put. Among the three people who died here two was buried without our knowledge, the one was buried here it is not an Ebola case but we tell God thanks when they did not come with the medicine and so Ebola is not too rampant here”.

M: So, what do you think about that medicine?

R: “Well they provided the medicine, we have seen many take it and they are no longer get sick again. Some of our brother who are far away when they took the medicine they came and told us that the medicine is fine. He said when they take it they sleep and before morning now that body will be light”.

M: That medicine?

R: “Yes”.

M: But when you ask the other people what are their concerns about those medicines?

R: “Some say when they take the medicine, I mean like when they are giving somebody a measurement and tell you to take one and you go and take four just for the to get well quick, some die as a result. They advise us to take one for the day some take all the four or five in one day”.

M: But here how is and what are the responses that you get from people about that medicine? Did they say they want it?

R: “Yes, we want it, but we have people since the incident they come and study us for any other case of Ebola. Maybe they have seen that Ebola is not rampant here that is why they did not come with the medicine here”.

M: Have you heard about any new way how to prevent Ebola?

R: “It is plenty”.

M: Have you heard about any new way?

R: “We have not heard about any new away or anybody to come and study us”.

M: No, have you heard about any new way which they have come with on how to prevent Ebola?

R: “Yes”.

M: Which one?

R: “The one which I heard they said let us take time”.

M: To take time about what?

R: “That any town where there is Ebola we should report quickly”.

M: That is the new message which you have heard?

R: “We can go to the place and see the person we can call 117 to come and take the man if it is Ebola, we call government and that we should not waste time”.

M: Have you heard about any vaccine?

R: “What is vaccine?”

M: Something like Marklate (=vaccine) which they say they are coming to give for the Ebola?

R: “No, we have not heard about that”.

M: But have you heard about it?

R: “Yes I heard about it”.

M: How do you think about it?

R: “Many people are not going to agree”.

M: Why would they not going to agree?

R: “I am not saying any other thing but like you and I now they can agree but if it is the white man they will not agree they will be afraid”.

M: Why would they be afraid?

R: “They don’t have the believing”.

M: Why don’t they believe?

R: “They said let nobody touches his or her companion but maybe the man who is coming since is a white man they will not believe him but if it is black man you cannot do me bad, but if it is another person or the white man that is giving the Marklate (=vaccine) believe me - man will run away”.

M: They will run away?

R: “But you I know you, I know you will not do me anything we are all in Sierra Leone, but if it is the white man I will just peep and see the white man, those old women when they see him they will say they are afraid of injection. If it is you, you can call me we sit down and you give the Marklate (=vaccine)”.

M: What are some of the question that your … ask you as a Traditional Healer?

R: “Some can come to me ask me to cure them so that they cannot get the sickness that is passing but I tell them no because we do not have the allowance, the government said we should not care if I give you anything if it disturbes you government will arrest me. That if I give any medicine and Ebola broke out in that area government will arrest me. The day government will ask us to cure you they will come and take and train us before we cure you”.

M: Apart from that is there any other question which they ask you?

R: “Yes”.

M: About the Ebola sickness?

R: “Yes”.

M: What are the questions that they ask you?

R: “Government said we should avoid touching a=each other, and we should avoid gathering let us avoid eating animal like bats, beef and even dead body we should not wash it but government have not trained us how to wash dead body so that we cannot get Ebola”.

M: Is there anything that ask you about Ebola which you are not able to answer properly/

R: “Yes”.

M: Like what?

R: “They said how does Ebola affect the human being? They ask me they say if a person is crazy you can cure him, if a person is paralyzed you can cure him, when a person has stomach you can cure him, a person is vomiting you can cure him but why is it that you cannot cure this Ebola?”

M: How do you answer that question?

R: “I tell them that nay person who is older then you if he tells you not to do something, don’t do it, the day they ask you to do it do it. But if you are warned that do not cure this sick, do not touch the sick”.

M: Is there anything that the people need understand better?

R: “Yes”.

M: Like what?

R: “People believe that if the cure this sickness the native way many have the belief that we can drive the sick it will not come to Sierra Leone”.

M: Is there any information which the people need to know about Ebola?

R: “Yes”.

M: Which one is that?

R: “Like you now you are here sitting with me face to face and you tell me what is the bad thing and me too telling you what is the correct thing”.

M: Which way do you think is the best way to explain to these people?

R: “You have come we know you now that you come here to tell us how we can drive Ebola so when we see you we are used to you, if any other person wants to come and see us we will be afraid but if it is you anything you tell us we will agree and accept, we have now Taken you like a father and mother and we believe you now we will have no fear if they see me go around and call them to a meeting they will believe but if another person comes we will begin to ask that every day is a new face”.

M” Do people come to you for this Ebola business?

R: “They and ask me but I tell them that this sickness is for my type”.

M: Why do they come to you?

R: “Is because they know that I know how to get medicine and I know how to cure, so they have the confidence that if this sick attacks them I can cure them but I will not have the mind to tell him that I have medicine for Ebola.”

M: When those people come to you what do you tell them?

R: “I will tell them that please if there is anybody who feels fever for two day to three you should go near to the hospital I tell them that if there is anybody feeling those sign should go to hospital if the person did not go and anything happen in that town when I go there the town Chief, the Mami Queen, the Youth Leader I will carry of them but our overall boos wanted to keep the sick in this town but I told them that I report. But I will tell them that I don’t have the power to cure them, if government gives us the power to cure them we can cure them. We cannot just get up and begin to cure people that means we don’t want the sickness to leave this country, it is the educated that shows us how to do thing and we do it”.

M: Thank you very much for when we are able to sit down to talk these things, this is a frank talk. And be rest assured like I told you, I am not a police, I am not a makeover and I am not government at all. Be rest assured that what we have said here is confidential nobody is going to get this information, so we are all just trying to develop a marketing strategy to promote Ebola treatment seeking behaviour, that is the main thing why we are here. I want to thank you very much for taking this opportunity to sit and talk with your small brother, I thank you very much Sir.

R: “Thank you, let us make ‘dwau’ (= A religious sermon)
