# Supplementary material for: Development of a set of community-informed Ebola messages for Sierra Leone
Source: PLoS Negl Trop Dis. 2017 Aug 7;11(8):e0005742. doi: 10.1371/journal.pntd.0005742 (PMC5560759; doi:10.1371/journal.pntd.0005742)
Supplement: S1 Appendix — (ZIP) [file pntd.0005742.s001.zip › Ebola messages - FGD and interview transcripts/R2HC Ebola Fieldwork 1/R2HC Ebola F1 COM-Trad3.docx]

| CODE | **R2HC Ebola F1 COM-Trad3 (semi-structured interview with traditional healer in the interview community)** |
| --- | --- |
| DATE | February 2015 |
| DURATION (minutes) | 60 |
| Collector nr | 1 |
| LANGUAGE INTERVIEW | Krio |

**PERSONAL DATA RESPONDENT**

| Age *(in whole years)* | 47 |
| --- | --- |
| Sex (F = Female, M= Male) | M |
| Religion | Muslim |
| How much time does it take you to walk from your house to the nearest PHU? (minutes) | 30 |
| Mother tongue: | Temne |
| Education level: | Primary |
| Role in community: | Traditional healer |
| Do you know anybody who had Ebola? | Yes |
| If Yes, what is your relation to that person? | Friend |

**TRANSCRIPT: (M= Moderator, R=Respondent)**

M: Yes sir, when did you first hear about Ebola?

R: This Ebola, it has taken a long time I heard of it”.

M: What did they tell you about this Ebola Sickness?

R: “This sickness, they said you should not touch a sick person, they said if you touched a sick person, you will be affected, the sickness will be transferred to you, so you don’t have to touch”.

M: That is all what you were told about this sickness, how it is?

R: “They said if you touch a sick, or when a person is sick, you are with that sick person given assistance, the sickness will be also transferred to you”.

M: Do they tell you about the signs and symptoms of this sickness?

R: “Yes, they showed me the signs and symptoms of this sickness”.

M: What did say?

R: “They said, if you starts experiencing frequents stooling, vomiting, automatic head ache and back pain, then you have be infected with the sick”.

M: When they told you these, what came into your mind?

R: “The things that came into my mind, because we are dealing with people, well I saw a case, which I dealt with somebody that was sick”.

M: You said they told of signs and symptoms of this sickness, which came into mind, when you heard of this?

R: “Well automatically I have being affected by the Ebola disease, what I mean, automatically I had contacted the disease because I had played with somebody that is experiencing frequent stooling and vomiting”.

M: Oooh, you have played with somebody who is experiencing frequent stooling and vomiting?

R: “Yes, I have played with somebody that is vomiting and experiencing frequent stooling automatically”.

M: What really happened with that person?

R: “The person was vomiting and stooling frequently I took care of the person, did everything for the person, this person passed away”.

M: So the person died?

R: “The person died”

M: Ok ….yes sir?

R: “The person died”.

M: Ok, how has Ebola affected your community?

R: “This Ebola has affected my community greatly, because it has killed plenty people”.

M: Have you seen people that had died from Ebola?

R: “I have seen a person that has died from Ebola, but what doubts me presently, which people are still denying, there is someone who had a wife that was sick of Ebola, the person was living in the same house with his child and the sick woman, he was assisting the woman do everything, but this man before the sick got worsen on the woman they had a sex, after having sex, within the week the woman got sick and die, when she died, the child and the man that was assisting her, were quarantined, these two people, nothing happened to, they are in existence, the place was quarantined for twenty-one days, those people did not sick, even a simple head ache they did not experience, this had put me in the state of confusion and people are denying that Ebola does not exist”

M: So you mean, the husband survived and the woman died?

R: “Yes, the woman died but the husband and the child survived, nothing happened to them”.

M: Nothing happened to them?

R: “Yes they survived; as I am speaking presently they are alive”.

M: Why do you think Ebola has spread throughout Sierra Leone?

R: “Why do I think Ebola has spread, Ebola has spread because of the touching, because the people we love, if they got sick and we are living together, we just have to render assistance to these people, for this person not to die, you have to help the person one way or the other and through these assistances, you may fall victim, the virus will be transferred to you”.

M: How do you assist the person?

R: “Assisting the person?

M: Yes, how?

R: “You may hold the person’s hand, the person may want to ease up, may want to drank medicine but very weak, they person may want to stand-up or laydown and also may want to change the position by helping the person to do all these, you may end up contacting the disease”.

M: What do you think is the best way to prevent Ebola from spreading in Sierra Leone?

R: What do I think is best, now the best thing to do, when our relatives are sick, let’s try by all possible means to protect ourselves, give the person a separate room and be assisting the person, so you and the other people will be protected, and before the sickness get worst we will called for assistance”.

M: Which way do you think is the best way to treat somebody with Ebola?

R: “The best way to treat somebody with Ebola, is to take the person to hospital, the centre, immediately when you suspected an Ebola from your person, called the medical team immediately to come and pick the person, if we continuing doing so, that will cut tail the chain of transmission”.

M: Do you have any local term you described Ebola in this community?

R: “Well, we don’t have any other name but only Ebola, Ebola, Ebola, even you asked a little child, they are aware of that”.

M: Some people do not believe Ebola exists. Do you know people in your community?

R: “Yes, people are doubtful of this Ebola”.

M: Why the doubt?

R: “If a wife of your friend had got sick and they were living in the same house assisting, and later the person was taken to the treatment centre and died, and her husband and child do not sick through the start to the end of the quarantined person, you must have a difference thoughts and you will not believe for that reason”.

M: Please can you give some examples of the Ebola messages that you heard, seen or read?

R: “Yes”.

M: Like which ones?

R: “In this community, our brothers went for training, they came and said Ebola is real, do not touch, they came with these slogans and we picked it up and told our people, we explained to our native people, that Ebola is real, we do not need to touch, so we were told by the medical people and let abide by it”.

M: What all the Ebola message you have across, which ones do you think worked well?

R: “The one they said do not touch, to my feeling, the don’t touch message worked well, if somebody do not touch the affected person of Ebola, the person will not contact the sick”.

M: Are there any Ebola messages that you think do not worked so well?

R: “Like how I said, somebody has been with his wife and the wife died of Ebola and there were children assisting this woman, and this person died, so this thing really confused me that Ebola is real, this person is still alive with the children nothing has happened with them, this is really confusing, when they said Ebola is real don’t touch and the people that were assisting the woman that died of Ebola are still alive, they touched, everybody touched but yet they are alive, this confused me”.

M: Ok, what do you think would be a good message to encourage people to bring patients to the treatment centre?

R: I want to encourage everybody, at this present moment to end the Ebola thing in our community, I am pleading to my people, if you are not feeling to good or one way or the please report yourself for check-up, they will collect the blood sample and test, so they will be free of Ebola, it will be better”.

M: Is that the message you think, you tell the people so that they will be encouraged to go to the treatment centre?

R: “Yes, for the people to please go and test, test is free, in any area, all the people are free to do their test, do your and they will give you certificate that you are Ebola free, so everybody will know their status that they are free from Ebola”.

M: In the event of Ebola infection, do you think people would prefer to first to a traditional healer, or to the hospital, what do you think this is?

R: “Well we have our native people that have stronger believe in the traditional healers, they prefer going to the traditional healer to that of the hospital, because they knew the traditional herbs”.

M: “I know you are a traditional healer, are people coming to you for healing?

R: “Yes, people are coming to me for healing”

M: Ok, when people are sick in this community, where do you think they will first go?

R: “People will come to me and also go to the hospital, and they do not give priority to a single place but both places, they will touch the two sides, to the traditional healer and the hospital”.

M: But where they will first go?

R: “To me the traditional healer, before they go to the medical people”.

M: Why do you think they go first to you the traditional healer?

R: “It is because we are experts of herbs and we had the know-how to play with it, so that is why they usually come for check-up for them to be satisfied, they really believe the herbs than the medical treatment”.

M: some people stay at home when they think they may have Ebola, why do they think this is?

R: “They are afraid because of the messages, they heard before now”.

M: Which messages they heard before now?

R: “The messages they heard that when you go to the hospital they will administer injection to you and you will die”.

M: What do you think could be the good message to encourage them to come to a treatment centre?

R: “The message I them to tell the people is, we are administering treatment to everybody that go the treatment centre and will take better and good care of the patients, let them come, we have human beings at the centres, they are there to render assistance to the patients, the medical people are ready to serve their country, let the patient go, they will well receive”.

M: What do you think would be the best channel to get your new Ebola messages to the people?

R: “They have to pass through us the traditional leaders, because our people had so much believe in us, so if the message come through us, we will pass it on to the people for their understanding because they believe in us, any instructions we gave them, they will listen”.

M: But do you think this is the best way to pass on Ebola messages?

R: “Yes, because if they pass the message through the traditional healers, it will filter down to the people, the people that are down there, they really have the believe in us, they will say that traditional healer over there, they knew this and that, so let go to them, so if they give the message to the traditional healers, it will reached the last person down there”.

M: Have you ever heard people talking either a good or bad way about the Ebola ambulance service?

R: “Some people are not happy the way ambulance people come and collect the patients or a corpse, the people that are with ambulance, do not treat the patients or corpse well, they will shout on them and be arrogant to the people”.

M: Have you heard any good thing about them?

R: “Well they said the ambulance, people are happy with the chlorine they spray, after spraying the chlorine, on your way to the treatment, if you don’t have better resistance, you will die in the ambulance before they arrive with you. If the sickness is Ebola or not Ebola”.

M: Have you heard a good thing the ambulance have done for them?

R: “They do not have any good they speak about the ambulance, they only say, you will [be] locked up in the ambulance, that is disturbing most of the people, everyone is saying the chlorine, the chlorine”.

M: Have you ever heard people talking ever good or bad about the Ebola holding and/or Treatment centre?

R: “The people that were at the holding centre and/or treatment Centre, before now they were not treating people very well at all, but it is only now they are treating well, people were grumbling about the bad treatment and the centres, but I think, they had talked to them and they are treating the people welled”.

M: Were they telling you the reasons why they were not treated well?

R: “Well they were given them their food with respect, they will push the food towards them, like feeding any dog”.

M: What had changed them from these unfair treatment to better treatment?

R: “Well they spoke to them to change their way of treating the patients, their bosses spoke to them that is why they changed their way”.

M: What about the burial teams, is there any good or bad about them?

R: “The burial teams, they were not respecting the dead bodies, they will just treating the dead bodies as how they like, they will just send and packed the dead bodies, and they had no respect for them, they will just throw the dead bodies in that disrespectful manner, they will just packed them like sardine fish”.

M: Are they talking good things about the burial team?

R: “Yes, they are talking good about the burial teams”

M: What are talking?

R: “Now, the burial teams they handled and treated the dead bodies well and take the bodies to the grave yard and bury well?

M: Have you heard of any secret burials?

R: “Yes, I have had of secret burial in other parts of the country and they bury and people we go and exhumed the body and bury again, we have been hearing about that”.

M: Do you know why were they doing that?

R:”The people do not believe that Ebola is real, that was the reason they were exhuming the body and bury again “.

M: Have that happened in your community

R: “No, it has never happened in our community”.

M: How are people talking about the Ebola phone line 117?

R: “Well the 117 Ebola phone line was not working well before, the phone is always engaged, and it is a busy line, you will tried and tried, but to no avail. They will answered you, but they will not response to the called, so the dead bodies will be in the home, for one day, two days and almost three days before they come and pick it up”

M: What about now?

R: “Now it is better, as they called, they will response to the call immediately”.

M: What they talk about the nurses that work at the treatment centre?

R: “The nurse, they were not doing well before but now the nurses doing well”.

M: What do you mean, they are doing well?

R: “They said, the nurses were not talking to patients well, they just throw medicines on them, and they will not give them the medicines hand to hand they will just throw it on the patients”.

M: How do people react to Ebola survivors in this community?

R: “We are receiving our people that have survived well, I did not say anything odd to them, we are always with, and we are telling them to put into practices the medical advices given to them by the medical people, and they must stay off from sex for three months”.

M: Have you seen any difference between the people and the survivors?

R: “Well, only these days when we received a report that one survivor and his girl lover kiss and sucked their tongues popularly, I was at the scene when they were talking about that, then I asked, so it really happened? Well I said if this is true, they will do the same thing, when they are in their rooms, or what will happened when they are in the room”.

M: Are people pushing far off from the Ebola survivors?

R: People are receiving the survivors well, they playing and joking with them, sit together and they do not point fingers on them”.

M: Have you heard of any new treatment for Ebola that will become soon in the country?

R: “Yes, we heard of one vaccine, which they have come and I don’t, they said it is going to be administer first to the medical people, the nurses and the Doctors, so we heard”.

M: What kinds of concerns do you think people will have about the vaccines for Ebola?

R: “Well the said the vaccines would be taken first by the doctors and nurses, to protect themselves, it is a prevention for them, when treating the Ebola patients, so we understood”.

M: Have you heard people talking differently about the vaccines?

R: “Some are afraid to the take the vaccines, some say they will take vaccines, some are afraid to take it right now, they said they don’t know the reaction or side effects, that is the reasons they are afraid to take it”.

M: As a traditional healer, what are the questions that your people asked about Ebola?

R: “Well the people always say, they are doubtful of this Ebola, and what is doubts, they said, we should not touch, we should played with a person who is sick of Ebola, but we have seen somebody have got sick, they had played with that person, have sex with the person and the person had died, but the people that came in contact the sick person are alive, nothing happened to them, even a simple headache do not affects them, they are doing well, that is why they do not believe Ebola is exists”.

M: What do you think people need to understand, that they have not yet understood about Ebola?

R: “There is nothing that people wants to understand again, because they had preach it to them, that Ebola is real, Ebola if a person is sick, you don’t have to touch and played with the person, and all the people knew now that Ebola is real but people are just doubtful because of the things that have happened, they said when someone has is you should not touch and that has happened nobody died, so people became doubtful”.

M: If you were an Ebola survivor, what is the problem do you encounter?

R: “I will encounter problems, because people in certain areas are still afraid of the Ebola survivors, women will not want you for love, nobody will come near you, and they will be pointing fingers on you, all these things”

M: How do you feel, they should be treating the Ebola survivors in your community?

R: “They have to be treated well, give them a warm reception, and play with them, encouraged them, sit with them and advise them to patience to stop the extension of the problem”.

M: What do you mean to advise them, how to advise them?

R: “To advise them that what the medical people had said, that they should wait for three months without having sex with a woman, if it is a woman you should not have sex with any man after three months, that’ is ninety days, some will not patience for the three months, that why, when returned back, we will continue advising them and our eyes will always be on them to avoid a reoccurrence”.

M: Do you have anything to tell me again about this Ebola sickness?

R: “What I am telling you, we are begging, the government has done its own efforts, we are telling our people, this thing is real and this sick had killed people, although people has some doubt about this Ebola, some are saying Ebola is real and some are saying it is not real, this is the understanding of people about this Ebola, because of different things they have seen, people came in contact with this sick people but are still alive, so people do not believe”.

M: Do people come to you as traditional healer?

R: “Yes, people comes to me”.

M: What are they coming to do?

R: “They are coming for treatments”.

M: Who are the people that mostly come to you?

R: “They are mostly women”.

M: What are types of sickness they come to you for healing?

R: “Sickness like “allay”.

M: What is “allay”

R: “It is a powder like thing, it is a sickness that is sent on an individual, and this individual and this individual will experienced rashes all over the body, the body becomes swollen, you get bumps on the body, some may come and complained the head, some will complained about “Fankay” (=fired with witch gun), they will hose blood”.

M: But do you cure these sickness?

R: “yes, I cured these sickness, I will go and get medicines from them, I am able take care and cure these sickness”.

M: So even at present you are curing them?

R: “Yes, presently I am talking they brought a person to me that was fired with witch gun, the person was hosing blood from the nose, and I have cured that person”.

M: The person got cured?

R: “Yes, the person got cured, still this sick people are in doubt. They brought another woman to me again, she was vomiting they said, it is Ebola, the lady ran away from her community, she came to me, I got medicine for her and she was cured”.

M: OK pa, thank you very much for given me this opportunity to interview you.

R: “Ok pa, thank you, we are always glad when we saw you people, because you carried the message to the people that we the traditional healers exist and we are doing our own best, because if we were not around, we should have not able to cure some of the sickness, which should have led to lose of plenty life, but we have tried our best”.

M: I thank you sir.
