# Supplementary material for: Development of a set of community-informed Ebola messages for Sierra Leone
Source: PLoS Negl Trop Dis. 2017 Aug 7;11(8):e0005742. doi: 10.1371/journal.pntd.0005742 (PMC5560759; doi:10.1371/journal.pntd.0005742)
Supplement: S1 Appendix — (ZIP) [file pntd.0005742.s001.zip › Ebola messages - FGD and interview transcripts/R2HC Ebola Fieldwork 1/R2HC Ebola F1 COM-Trad4.docx]

| CODE | **R2HC Ebola F1 COM-Trad4 (semi-structured interview with traditional healer in/linked to interview community)** |
| --- | --- |
| DATE | February 2015 |
| DURATION (minutes) | 40 |
| Collector nr | 1 |
| LANGUAGE INTERVIEW | Krio |

**PERSONAL DATA RESPONDENT**

| Age *(in whole years)* | 45 |
| --- | --- |
| Sex (F = Female, M= Male) | Male |
| Religion | Muslim |
| How much time does it take you to walk from your house to the nearest PHU? (minutes) | 10 |
| Mother tongue: | Temne |
| Education level: | Primary |
| Role in community: | Traditional healer |
| Do you know anybody who had Ebola? | Yes |
| If Yes, what is your relation to that person? | Friend |

**TRANSCRIPT:**

M: When did you first hear about Ebola?

R: “Haaah Pa, I heard of Ebola from the 25^th^ of May 2014 when the government announced that we have got a sickness in our country called Ebola that was the time I heard of the sickness, and I don’t even know the name that they called”.

M: How was this disease described to you?

R: “Well according to the traditional healers “eeeh” medical people, what they told us is that, this sickness called Ebola has the symptoms called vomiting, frequent stooling, and diarrhoea, so it was called since they have the know-how, we did not get any understanding, bleeding will occurr from the ear, nose of a person, the head will become very hurt and any person(s) with these symptoms is a suspected case of Ebola, that person should not be touched by any other person, that is what I heard of this sickness”.

M: After you heard all this descriptions or explanations what came into your mind?

R: “Well with that, I took it as untruth because I have the understanding that we had headache, vomiting, and frequent stooling so if they come again and say we have Ebola, I took as a plan by the Government to kill us, so we do not have the believe that this sick exist, because there is nothing new about this thing, that is the reason we don’t believe the existence of this sickness called Ebola”.

M: Why you did not believe it at first?

R: “Aaah the reason, they said vomiting and there are a lots of people that vomit, they said headache and people always experience headache and then take medicine and get better but if they said these are symptoms of Ebola, that is why we don’t believe the existence of Ebola, lest I don’t tell lies, I do not believe Ebola is real and it exist, I don’t believe at all”.

M: How has Ebola affected your community?

R: “Aaaah, really it came and worked, because you can see the problems it caused and they said it is this sickness, but since they said it is Ebola, we saw they came and took, children, mothers, fathers, young boys and girls, they told us they are suspected Ebola victims and they later came and said most of people had died and they did not tell us anything convincing, only the following day, they came and told us they had died. That was the thing we made to understand”.

M: Have you personally seen or known people who have had Ebola?

R: “Yes, the wife of my brother which name is (- - name of the wife - -), she is short, fat and black in complexion, she complained that she is feeling her head, they took her to the hospital, the next day her sister’s daughter came and told us that the (- -name of woman- -) had died and we spoke to her the following day, the other morning they said (- -name of woman- -) is dead”.

M: Why do you think Ebola has spread throughout Sierra Leone?

R: “Well to my own understanding, the government said we should not touch, traditional healers we should not touch, and we cured people, and by curing them, is the only means we get our daily bread or our daily survival, like what happened, a woman came they said she was sick of “Gbahagba” and people has the believe this has been happening, a woman will be pregnant and not able to deliver, it has been happening and for the “gbahagba” we have being providing medicines and they have been rubbing it, so if they have said we should not touch, it is going to be difficult to be cured because we as traditional healers we will touch, if my child is sick, you will tell me not touch my child? My wife is sick I should not touch her? Do I have to run away from her? That is why I said, this sickness it is going to be difficult to finish in this country”.

M: What do you mean by “gbahagba”?

R: “In our native settings when they said “gbahagba” you may not able pass out faeces, urine, and your stomach will get a standstill, that is what we call “gbahagba”, and we have ceremonies which we perform, rubb the medicines on the stomach, that will enable the person to get normal stooling and urination as how the person was doing it before”.

M: What do you think is the best way to prevent Ebola from spreading?

R: “The only way to prevent Ebola, we has traditional healers need to bring inside, let them tell us and we will see, let me don’t tell you lies, people really believe, trust and respect me in my little corner, so if they are saying that, touching, the headache, the stomach pain, diarrhoea is a cause for the death of people, let them bring us inside, maybe we will know what is happening and we will amend and rub our hands”.

M: What do you mean by “Bring inside”?

R: “What I mean bring inside is, the government has stopped us from healing people and this is the work where I got my survival or daily bread, I have my children which I have to take care of, and the government has said we should not give treatment to people, so if they provide training for us, so that we will be part of the team, they are (have) incorporated other people that are treating people for Ebola, so if they can also involve us, it would be fine because this is how we survive, government will tell me not to touch yes I will agree with whatever they say, but in my own little corner, I will be giving healing to my patients small small”.

M: What do you think is the best way to treat somebody with Ebola?

R: “Well really…. that, this thing has become difficult to cure, difficult to treat, the only way unless government involved people from different area, they said even the people that have being cured from Ebola should not touch woman, why government should not provide a place for them to live, the sick people should be at side whilst the people that has been cured from the sick should be at another different side because they will be having contact with women and the sickness will continue spreading. If they are able to tackle this and bring in programmes how to solve this thing for us, that we may not be able to touch, it would better, let them provide us with kits, we may also try ours, that is the only way I believe the sickness will be tackled, let them provide this kits to the people like “combra” (mothers of children) whose children had got sick let them give them the kits, they will take care for themselves”.

M: Which kits are you talking of here?

R: “Well we have seen the ones the doctors wore that resembles “kaka debul” (masquerades) the one they wore which resembles those white men that goes up the sky, these are the kits we them to help us with”.

M: Are there any other names they called Ebola in this your community apart from word Ebola?

R: “As native people we called it “Taa buun nenee”

M: What do you mean by that?

R “Don’t touch, “taa buun nenee” the sickness that came is the sickness we have here”.

M: Some people still believe Ebola do not Exist, do you know those people in your community?

R: “Yes, not only once, twice or in thrice cases they wanted to beat me up, they have done it and it is real?

M: Why they wanted to beat you up?

R: “Government appointed us as taskforce to be explaining to our people, when we came and explained to them, they said we are telling lies, this sickness does not exist, government wants to embezzle money, they needed blood, this is word ooo”

M: Do you know why they have this view?

R: “The problem is before people said they want to see Ebola patients and now we have seen them and people are still saying it is untrue, it is a common headache, a native sickness and you know you have these sickness before, we will go into the bush, get some medicines put into the calabash, rub it, when you go home you feel better, so these are the ways but people don’t have that perception, although some are better off now compared to before but some are still not believing, myself there is no way, but just watching, because our bosses had told has do not this, and fearing not to lose my certificate that I have struggled for, our president has past that law, the traditional healer president”.

M: Have you seen some Ebola messages?

R: “Yes”.

M: Have you heard of them again?

R: “I have seen it in my phone, on television and I have been reading all of them”.

M: What do you think of these messages?

R: “All about these messages, I have not seen any one again, because when you go this side, they said wash your hands, when you go to the other side, they said no touch, do not touch sick person, do not touch dead bodies, all about the messages are just the same, there is no new ones, it is all about “taa bunne”

M: What do you mean by this “taa buune”?

R: “Don’t touch, that is what they mean in English, don’t touch sick person, don’t touch dead bodies, you have to avoid everything, when some is sick you have to avoid that person”

M: They are saying they should avoid and you are also telling they should, do you think people are avoiding?

R: “It is not easy”.

M: What is really making it not easy?

R: “We all have the traditional belief that when someone is sick, you should be by that person, if you are not by that person, it means you don’t have person’s interest and if you are not by that person, when the person gets well, the person will tell his/her grievance, that when he/she was sick you do not take care, so if the person is the one that is responsible for you, the person will just ask you out, saying when I was sick, even to greet or come around me was a problem it very difficult”(*long silence)*

M: Do you have any other messages that did not work well?

R: “uhhmm this is the thing government had created fear in us, all others that is come after, they said this particular sickness cannot be cured, that has made all our relatives had ran away, now when a person got sick, the person will run into the bush, but now if they come and tell us that it begins when, two to three days when a person is being taken to the treatment centre, the person is dead, so what is the thing? So for me the best way message is that, the government should do something, like what they are doing, when a person is sick immediately, they come and take the person that will be the best”.

M: What do you think would be a good message to encourage people to bring patients to treatment centre?

R: “Government came and said there is no medicine, and when there is medicine you will have the courage, but if they tell you, that person had gone and they had killed the person, this was what they were telling us, as you go with your patients, they have injected the person and the person will die. They had told us there is no medicine, now if the tell us, you have a chance of survival, this is what is creating fear in our people, let them be showing people on television that have survived, let them show the survivors for their families to see how they are treating them, let them show us, let them be encouraging people to go and see their loved ones and see how they are treating them, so that tomorrow I will be happy to take my patients to hospital.

M: In the event of Ebola infection, do you think people would prefer to go first to the traditional healer, to the hospital or treatment centre?

R: “They told us that we should call117 and the 117 has a waste of time and if you go to your councillor, the councillor will tell you to call 117 and when you go to the hospital; they will tell you go and wait, we have our waiting centre “opotokatty” so they say in English, we have our waiting centre and there is delay in the process and when somebody sick, the person needs medicine, if they should try and create a temporary place to be treating and checking people little by little it would be better”

M: In this your community, when someone is sick, where the person will prefer first to go?

R: “The first person I will meet is my councillor”

M: Apart from councillor, who do you first prefer to go to for cure?

R: “Unless I go to the hospital and in sometimes when you go to the hospital, they will ask you to go back to your house, have you seen, this is the issue, the men we called contact tracers when you go to them, they will tell you wait let me call and when you go to the hospital again, they will tell you to wait for them to call. So the best way, some days will go to the hospital and when we go to hospital we can’t able to get them and when you call on them, they will say, wait we are coming”.

M: So which place will you first go?

R: “To the “well bodi pipul” (The medical people)”

M: How do you see the centres they have established and you spoke about the medical people?

R: “Well really we have seen them, they are well constructed, like even our hospital has the way constructed it, they made one side separate and the other side separate for the people that are sick of different sicknesses, the people that they are not sure its Ebola, suspected cases of Ebola but the centres are fine and big we tell God thanks, the cases are getting less and am happy people are not going there again”.

M: How do you assess the nurses that work at the centres?

R: “Well in fact they are the ones that do not treat the people well, because when you go there they wouldn’t have time for you and they wouldn’t want to know about you, the nurses, as they said this is the one, they can’t even talk to you, if they are going to you, you will far off from them, if you tell them to give you water, they will say there is no water, with all these behaviours, the nurses are not treating us good”.

M: Why do you think they are doing this?

R: “Well they are also afraid because plenty of them had died, nurses had died, and you have to love yourself, you have to love yourself more than any other person”.

M: Some people stay at home when they think they may have Ebola, why do you think this is?

R: “Is nothing, but they are afraid”.

M: Afraid of what?

R: “Fearing that when they go there, they will die”.

M: What think could be done to encourage them to come to the treatment centre?

R: “The only way is, let them allow them to go there with their relatives, let them provide a separate place where their relatives will sit differently and a different place for the patients, so that their relatives will be watching them, so that they be confident”.

M: What do you think would be the best channel to get your new Ebola messages to people?

R: “The best thing, they have to bring in the traditional healers, when we are brought in, we have to spread, and some of us have ideas that we will exhibit, we believe the leaves (traditional herbs)”.

M: Besides the traditional healers is there any other channel to relay your messages to the people?

R: “Yes”.

M: Which ways?

R: “let go out and be talking in our local languages, tell the people the reasons, show them”.

M: Which medium should be used?

R: “Let them talk in Temne, Krio, and Mende, let them use the radio, television and tell the people what they announced last was a mistake and now we got the medicine, so the people will turn up”.

M: Which particular radio stations you think they will use in this community?

R: “We have the (- - name of local radio station - -), it is for (- -name of communities- -), let them tell the people to come all types of patients, be it Ebola or none Ebola sickness, let them the people, if they feel their head, let them come, if it is the sick or not”.

M: Have you ever heard people talking good or bad about the ambulance service?

R: “Like the ambulance, they are telling us that, they killed people because as they grasp you, board you in the ambulance then they spray chlorine on you, the chlorine they sprayed before you arrive you are weak and die. The ambulance are not treating well”.

M: Like how they are not treating people well?

R: “The chlorine that they sprayed when they open and put you in the ambulance and also they will spray where will pick you up, they will spray chlorine all over, and you get suffocated, the suffocation will cause weakness and it may kill them”.

M: Have you heard any good thing about the ambulance?

R: “The only thing they are helping in the transporting of people to the treatment centre for them to be cured”.

M: “How have you heard the people talking about the treatment centres and the holding centres?

R: “Now it is better we are hearing of that place where our brothers the soldiers are, they said, they had their own formula to cure but the treatment centres, the holding centres, they were not treating people well unless now when the white men have come, they are encouraging people, talk to you fine, give you what you need, before this time if there is water standing and tell a nurse to give you, they will not, they are not treating people well, they are just eating money free”.

M: But how are you hearing about them now?

R: “But now we are seeing our brothers, they said they are treating fine and they survived, it is better than before, so this has given the people the courage when they are seeing survivors and people have the zeal now to do their test, so now they are going willingly to do their test, if is it Ebola or not, if is it Ebola, they will go for treatment, knowing that, if they go earlier they will be cured. We have started viewing realities in what they said”.

M: What is the good thing about the treatment centre?

R: “they are telling that you should go earlier, when you go earlier to the treatment centre you have a better chance of survival, but if you delay to go is a problem to you, you have seen the way they are treating people, they gave water, medicine and food when they want to eat, it is better than before”.

M: How are people talking about the burial team?

R: “The burial team, the way they were handling our people when they died, they have discouraged the people, what we believe, you have to give the last respect to the dead, by washing, dressing and buried respectfully, but the burial team when they collect the corpse put in the bag, spray the chlorine and throw away the dead body in the ambulance, this was the problem with the burial team and the people”.

M: Have you heard about any secret burial?

R: “Yes, it is happening”

M: What do you think people are doing secret burial?

R: “Because in our tradition, the people says we have to wash the dead, after washing, then you bury respectfully, that is why people are doing burials at night. They spread-out the soil, they will not fold it as they do it before, they spread-out the old soil, people may not know but if there is any problem, they will solved it among themselves, this was another problem that raised up which they said we should bury, but we believed when you are dead, it is done, there is nothing that lives in the dead body”.

M: Are they still doing secret burial?

R: “Well now, it is getting better, it is not like before, it is better but in our own community they are not doing secret burial”.

M: How are people talking about the Ebola phone line 117?

R: “Well the problem of those people, they will answer your call but you will asked plenty questions, but to come to the place you are calling them it is a problem. You will called once, twice , thrice and even the whole day they will not come, so it has being happening, and some dead bodies even slept in some homes, you will called them, they will tell you they are coming they are at another place. When a person is sick, they will waste a lot of time to come, before they arrive the sickness had got worse”.

M: What are they talking about them now?

R: “Now it is much better than before, we are not getting that problem again, when you called they will response and you will also talk to their contact tracers and will called and after they had called they will come with their vehicle to collect the patient”.

M: How your heard anything about the staff that worked at the health facilities?

R: “The nurses, our nurses, thanks to God the soldiers stepped-in, they were not trained at all to handle the treatment, they were not having the training, and they were on the runs afraid of their lives, when you called them and go the place, they will say no, and not all the sickness are Ebola, because some of this nurses when you are sick, even if it is a minor sickness they will not treat you. So if you can find out not only Ebola sick that is killing people, all the other sicknesses are killing people”.

M: How are people in your community behaved to the Ebola survivors?

R: “Like really we a friend, is a survivor from Ebola, he just took the thing as a fun, because we always called and asked him, and we also called him as hero because he is the man that survives. Really our own community is better, we don’t make any different feelings towards them, we talked to them, they had been coming to us, they were with us, it is happening we are living very fine, we don’t have any separation again, they said when somebody has survived from Ebola, that person is free and totally freed and we have seen it because we sat down, talked and ate together, we don’t have any different way, we really don’t know if the survived person may think in a different way, but when someone always gave him a joke, telling him, you are an Ebola patient, a survivor and a freed person, he would laugh and take the situation funny, he will even explained to you what he came across, so we get our interactions”.

M: Ok do you touch these Ebola patients?

R: “Yes, I have a my man, in fact he was the last person that barbed me, which made be to be afraid and when he came back, we still sat, eat and played together I don’t have any problem with him”.

M: I have you heard of any new treatments for Ebola that may become available soon in this country?

R: “Yes, you heard of it, they said it is vaccines, so we heard”.

M: What do you about them?

R: “For me I wouldn’t take it”.

M: Why?

R: “I believe that they are the ones that came with this sickness, so if they come with another one again, maybe they had planned another thing again for us, so that is why I wouldn’t take it”,

M: Why you wouldn’t want to take it?

R: “They will just kill us, these people, they are the ones that brought Ebola, if they told us last there is no medicine for Ebola, now they said they have come medicine, paa, I wouldn’t take it - oooh, then I had even told my families, nobody is taking it, ahhh, I don’t believe again, let them just leave us as how we were”.

M: Have you heard of any new way to prevent Ebola?

R: “Well the only, they told us if you experienced fever, drink plenty of ORS (Oral rehydration salt) with some antibiotics, drink a lot of, so if it is Ebola, the person can gain a resistance until they you are taken to the treatment centre”.

M: As a traditional healer, what are the most common questions asked about Ebola?

R: “The common that asked is if really this Ebola sick exist, or it is a plan by the government to kill the people”

M: How do you respond to these questions and concerns?

R: “I always tell them what they told us that we should not touch, the sickness exists and I will not tell everybody that this sickness do not exist. I will tell them the sickness exists, because the government and our president has told to avoid”.

M: Which president?

R: “The traditional healer president, he has told us to avoid, if any person is caught treating secretly they will siege his or her paper, so these are some of the things but to me I do not believe Ebola exists, I seen they have come, but I am not sure if this Ebola which they said there is no medicine for and now there is medicine”.

M: What do you feel you need to know to enable you to respond more effectively?

R: “Paa, leave that one, you seen like that one, if they have told me of different thing which is not occurring on a person’s body, they should have convinced me, they should have abled to disturbed me a little, you are just telling me about vomit, stooling, head ache and pain, they do not exist? So these are some of the things, but if they should had told us about different symptoms that this and this happened, you will say ooh yes or you see someone fall on the street and bleed all over the body and blood hoses almost all parts of the body, you will say okay this is true”.

M: Do people come to you in relation with this Ebola?

R: “Yes, we got them, those that comes and tell to us, then will had another people?

M: What I mean, do patients visits you?

R: “Yes, this is what I am saying, we have our own small corner and the patients are coming”.

M: What is purpose their purpose of coming?

R: “They know that when they come, I am able to handle certain problems”.

M: Like which problem you are able to handle?

R: “I am able to cure gonorrhoea, syphilis, stomach pain, the “gbahaha, witch gun, fankay”. Some sickness that they put for some I will cure any sickness that has dealings with human being, if is human being that is responsible for the sick I will also cure, sickness, like head ache, stomach pain and vomiting, it is very common to cure”.

M: Can you please tell me the category of people that comes to you for cure?

R: “In fact we do not dealt with category, we deal with all types of people, like how you bring your problem”.

M: Like mostly which types of people that come to you?

R: “Young girls, old mammies’, young men, it is left to the problem you having, if you come and believe in me, I have my own to past, I will go in bush then come, I will know what I will do, because all my own medicine are from the bush, when I come if I am going to boil it or I am going to smoked you up, I will”

M: What do you mean by smoked-up?

R: “Well, We had what we called, after we had boiled the medicines, we will give bench to sit down, if is any sickness inside you or it was sent to on you, when will covered you with the cloth, the heat that will meet your body then you will sweat, that will removed the sickness in your body”.

M: OK, is there anything specific about Ebola that you think people need to understand better?

R: “Yes. We want the government to do these things, let them bring people, so we will see reality, let us be part of the process because we cured and people had believe in us, the others that are in different places is a different thing, we in our own community is a different thing and our people know these sicknesses, so if they give us the training and make us to see how this thing is treated and killed, we have our own idea and at least we can understand that this sickness is not a native sick, I have you understood”?

M: Yes sir, thank you very much for talking to me.

R: “Than you”.
