# Supplementary material for: Development of a set of community-informed Ebola messages for Sierra Leone
Source: PLoS Negl Trop Dis. 2017 Aug 7;11(8):e0005742. doi: 10.1371/journal.pntd.0005742 (PMC5560759; doi:10.1371/journal.pntd.0005742)
Supplement: S1 Appendix — (ZIP) [file pntd.0005742.s001.zip › Ebola messages - FGD and interview transcripts/R2HC Ebola Fieldwork 1/R2HC Ebola F1 COM-Urban1 V2 ADD PROBE.docx]

| CODE | **R2HC Ebola F1 COM-Urban1 (urban semi-structured interview with community leader)**  **V2 – 10^th^ March 2015 – ADD PROBE** |
| --- | --- |
| DATE | February 2015 |
| DURATION (minutes) | 33 |
| Collector nr | 6 |
| LANGUAGE INTERVIEW | Krio |

**PERSONAL DATA RESPONDENT**

| Age *(in whole years)* | 60 |
| --- | --- |
| Sex (F = Female, M= Male) | male |
| Religion | Christian |
| How much time does it take you to walk from your house to the nearest PHU? (minutes) | 20 |
| Mother tongue: | Mende |
| Education level: | Tertiary |
| Role in community: | Area chief |
| Do you know anybody who had Ebola? | Yes |
| If Yes, what is your relation to that person? | Neighbour |

**TRANSCRIPT:**

M: When did you first hear about Ebola?

R: “I heard about Ebola, from the time it entered Guinea and Liberia, because they were the first countries that got Ebola before it came to Sierra Leone, it was in February, 2014, and later in May 2014 I heard that it has entered Sierra Leone through Kailahun”.

M: After you heard of it, how was the disease described to you?

R: “They told us that it is a new sickness, it is bad and it kills quickly in a shortest possible time

M: What were your first thoughts about the disease?

R: “My thought was government wants to, to derail the census because in Kailahun it is the stronghold of the SLPP party and government announced that the Ebola sick is now in Kailahun, so some of us we started receiving calls from my district Kailahun and we inform people not to listen because it is government propaganda and we will give them information as soon as possible towards the manmade sickness”.

M: How do you think Ebola has affected this community?

R: “Ahhh” (in a sad mood), in plenty ways. it is really too much, one, it has even “hamburg” (=derailed) our economy, it did not only derail our economy, but resisted our movement, our social movement, I usually went to my village quickly in no time, but now I will take a whole day travelling, because you come down the vehicle for check-ups at any checking point, it is just like during the war and it has also stopped our children from going to school, this is the first time in my life I have seen eight months people did not attend schools, colleges, nobody is doing nothing, unless you are controlled by the government, our freedom is restricted, they said we have to work in line with the public state of health emergency, it has even made us to be afraid of our family members, we are afraid to visit our family members, with the fear that we will catch Ebola, work is difficult to get, it has given a bad name. A family friend told me that, when you travelled from Sierra Leone to America, they will look on you as a filthy rat, they will not come around you, and they will be afraid of even playing with you. So it has really affected us in many ways”.

M: Why do you think Ebola has spread throughout Sierra Leone?

R: “Ebola has spread throughout this country, because we were denying the fact that it’s real and it exists, and we were also politicizing it, thinking that it is the plan of government to reduce the population of the main opposition SLPP stronghold, some people believe it is manmade disease, but on a whole, it is an attribute of denial”.

M: What do you think is the best way to prevent Ebola from spreading?

R: “There are many ways my brother, let me tell you, one, we have to listen to health practitioners like the doctors, nurses, other health workers, and the Ministry of Health and Sanitation, if they said don’t do this, don’t do it, for instance they said we should not go closer and touch sick person, and we should not touch dead bodies, we should abide by all these measures. The other way is when you heard that Ebola is in your area, you have to abide by, what they tell you, wash your hands and your entire body frequently if possible as many as you could. Let’s us do things that will make the Ebola to leave us in a shortest time, if you can please give me some time to tell you, one, You don’t have to touch the fluids of certain people, I mean Ebola infected person, any fluid that is coming out from the person should not be touched, like saliva, blood, if for instance a brother has got injured you will afraid to touch the blood because you don’t know if he is having Ebola or not. Urine, wound, stool, saliva of an Ebola patient should not be touched, we should be careful to share things like razor blade, shaving sticks with our brothers, using these things in common should be stopped, we should be careful of the vomit of an Ebola patient. So these are the things we should be careful of”.

M: Do you have any term use to describe Ebola in this community?

R: “Well I have the youth calling it “Bolaa”.

M: “Bolaa” what is the meaning of this?

R: “Well, they said, it is the shortest way to call Ebola, the acronym of Ebola, some people call it “Debola” when it first came, this is nickname they gave, at times when they went to tell someone to avoid Ebola, they will say don’t go closer to “debola” oo, so that you will aware that, I have to keep away Ebola”.

M: Some people still believe that Ebola does not exist, are you aware of those people in your community?

R: “Yes, still the argument is going on, but it is not like before because they have seen physically the way Ebola is killing people. The denial is real; some people attributed it to witchcraft. We still have people denying that Ebola does not exist, they are there, and the denial is still around”.

M: Have you seen or heard any Ebola messages?

R: “Yes”.

M: Please can you give me some examples of the Ebola messages that you have heard, seen or read?

R: “Yes, the messages I have seen on posters, television are, Avoid Body Contact, don’t touch sick person, call 117 for help, avoid eating bush meats, and the signs and symptoms of Ebola, vomiting, fever, frequent stooling (= diarrhoea) and many more”.

M: Which one among those messages you think is the best, understandable and clear to the people?

R: “Well the best message is the ones on the posters, showing the signs and symptoms of Ebola like vomiting, fever and frequently stooling (= diarrhoea), it is the best, because it is clear and understandable to the people, they are talking of vomiting, on the poster you will see a person vomiting, having skin rash, fever all you see on the poster it is clearly shown”.

M: Are there any Ebola messages that you think have not worked so well?

R: “Yes, like the one which says Call 117 for help”.

M: Why not?

R: “Because before this time, when you call 117, the response is very poor, the message changes, instead of help is now stress and trouble, because if someone died, you will call 117, it will take three to four days before they come and collect the body, at that time all of you in the house will be in stress and trouble, the body should have gone bad and all the inmates in the household will be exposed to threat of infection”.

M: What do you think would be a good message to encourage people to bring patients to the treatment?

R: “The ones we see on posters which clearly says earlier treatment gives you the better chances of survival, when you feel sick, go straight away to the hospital for treatment, because if you decide to take treatment at home it will be bad for you”.

M: In the event of Ebola infection do you that people would prefer to go first to a traditional healer, the existing health facilities/ staff or the treatment centres?

R: “In the event the of Ebola infection, we are hearing from rumour that when the ambulance comes and collects a sick person, the chlorine that they spray disturbs people very seriously and it even kills, so they are afraid of going to the treatment centre, they will prefer the traditional healer, were there is no spray and nobody will see you, some people says the treatments are different, but these are all rumours”.

M: Some people stay at home when they think they may have Ebola, why do you think this is?

R: “Well some people think when they go to hospital, they will not survive and others believe Ebola does not exist. Others say the signs and symptoms of Ebola are similar to malaria, typhoid, so they will prefer to stay at home and take treatment”.

M: What do you think could be done to encourage them to come to the treatment?

R: “Well government through the Ministry of Health, should increase the level of sensitization, and get groups of survivors to move house to house telling the people that there is life after Ebola, and earlier treatment gives hope. So they themselves (will) go to hospital when they feel sick”.

M: What do you think would be the best channel to get new messages to people?

R: “Ehhh, one, through poster, why poster, even an illiterate, or literate you will see images of someone go to toilet on posters, also through newspapers, because 40% of Sierra Leoneans can read and always buy newspapers and the way is radio, who has radio will listen and hear. And we as community leaders are asking to be given someone precisely a nurse that knows more about Ebola, we will give the person megaphones/loudspeakers to move within the community sensitizing, educating our subjects about Ebola more in the morning when every is on in bed, we call that particular exercise with the megaphone as community radio, like we have one here, 5am they starts the sensitization, 6am they stopped”.

M: What all those channels you have stated, which one is the best?

R: “the posters”.

M: Why do you think this?

R: “Because images on the posters speak for themselves, even an uneducated person can understand an image of a man vomiting and having fever, not alone the educated person. So that why I said is the best”.

M: Have you heard people talking any good or bad way about the Ebola ambulance service?

R: “That will not end my brother, even these few days we heard that an Ebola ambulance got an accident, it fell down and the whole ambulance was damaged beyond repair, it was due to over speeding, and the other thing again, the ambulance is purely for sick people, but drivers are using it differently for another purpose”.

M: Which different ways or purposes you are talking of here?

R: “The other purpose I am talking is the drivers prefer loading other personal items instead of the sick person”.

M: items like what?

R: charcoal, transferring household materials like Beds, chairs, I am telling you for free, Sierra Leone was not having ambulance , I also told my wife, which is a nurse, that Sierra Leone was not having up to six ambulances in Sierra Leone, so people are now misusing the few ones we have, you can’t imagine loading wood is bad. But really this is by rumour I have heard but not yet seen it”.

M: Have you heard people talking either bad or good Ebola holding centre/treatment?

R: “I heard a comment and I am against that comment, like the holding centre or treatment centre they are no go areas. At (- - name of school - -), it position at that school a lot of people are grumbling, they should have preferred a playing ground or field; not a school. We have also heard that, when you went there, they will not treat you good and we have not found out if it’s true because it is not in my community and we were not invited, other people like the journalists are going there. I should have community heads or leaders, should be invited”.

M: Is there no good?

R: “Well I have not heard of it”.

M: Have you heard any good or bad talk about the burial teams?

R: “Well yes I have seen the way they bury on the television, people are grumbling, one, the way I saw it on the television, When the burial team take the corpse to grave, from the top of grave they will just throw the corpse “vaap”(= the sound of the body thrown), former it was put down easily, I was suggesting that they use a rope to put the dead body down the grave, we can’t imagine from my view on the television, I heard the sound “vaap”

M: Apart from that, is there any good about them?

R: “Now, it’s quiet different, because they are giving respect to dead bodies, they are not sending it in the grave as before”

M: What about the 117 Ebola phone line is there any good or bad?

R: “Well at first, you will call 117 for days before they respond, then it takes more time before they respond, well I think it is better off now. The other one issues again, you know Sierra Leone we are “tranga ace” (= stubborn), they call 117 to send ambulance, when the ambulance came, they said they have taken the body away, so the burial team were standing in front of my house grumbling and this bad, so if the people do not respond, this same story happens at (- - another area in the district - -), people made false call when the burial team went, they did not see any corpse. My brother people in this country are wicked so they told the guy that made the call that if there is no dead body, we will take you along with us”.

M: Any aspects of the existing health facilities/staff that is now working on the Ebola care and treatment centre?

R: “Well Yes”

M: how?

R: “Well I am not happy with the coming of Ebola, because only the government hospital could not be enough for this, they ban all private hospital, and these health centres, People are grumbling that it is not decent and the staffs there are very harsh with the patient, when these private hospital were existing they were helpful, at a time they help my wife deliver safely, but the government has put stop to all that, well the private hospital or pharmacy were very helpful, now they said they should not treat, everybody should go to the hospital, so with, there is a lot of overcrowding at these government hospitals, there is a lot of grumbling of not attending to patient, which has really led to death of some people, the nurses, doctors are not enough to attend to everybody, in fact most of them were afraid to touch a sick person fearing of contacting the Ebola virus”.

M: Are there people in your community that have survived from Ebola?

R: “Yes of course, a day before yesterday two of them passed here, and went to a place where they were quarantined and quarantined will finished today, two nurses”.

M: I am asking of the quarantined place but who has sick of Ebola and survived?

R: “Yes I am also talking about them here, this quarantined place, which should ending today, got two Ebola patient and the two patients returned home, a day before yesterday survived”

M: How is your community reacting to these survivors, are they encouraging or stigmatizing them?

R: “Well, stigma will never stop, it is part of us, I also got sick with a viral disease, there was that stigma but to say openly they have stigmatized those survivors, no, that has not happened in this community, people will be talking at their corners. Back biting but openly or physical attacked no, because government has put a great emphasis and preaching against stigmatization and medical practitioners are also advising”.

M: Have you heard of any new treatments for Ebola that may become available soon?

R: “Well yes, that was heard nationally, we have heard of the vaccines and the medicines, they call it ZMAP, I read about it on newspapers and I heard it on radio, the name of the medicines is ZMAP. And we also heard that towards the end of February, either Liberia or Guinea or another country will send for us vaccines to prevent Ebola, we are happy to hear that because through vaccines, we will be freed from certain illnesses like measles, polio and more has become less, we were calling it “korkor don go”(= the wicked one has gone)”.

M: What do you think about them?

R: “I think it is good because it is for cure and we are looking for a medicine to cure us from Ebola, it is just like when you want to drown

M: What about the concerns of your people about this treatment?

R: “The concerns are not bad, because they are all looking for cure, well the general concern here is, if the medicine will not create a side effect that will be another problem for the people”.

M: Have you heard of any new way to prevent Ebola?

R: “No, I have not heard of any new one, unless the old ones, don’t touch, avoid body contact and so on”.

M: As the area chief, what is the most common question about Ebola that you are faced with?

R: “They always asked, when Ebola is going to finish, because it is disturbing us, two, they ask what brought about Ebola in this country, some are asking are they doing something for the Ebola survivors and others ask, when schools are going to reopen for my children, some asked if the vaccines that is coming is for everybody not only specific people, they are asking if the treatment centres are going to be retain or they are going to be removing them, they asking if the medics that came to help are going to stay with us. They asked again, this Ebola sick is a new disease in our country, are they going to train our medical practitioners, and some were asking if this sick will not come again, a lot of question were as asked”.

M: How did you respond to those questions and concerns?

R: “Well, I told them, that all this boils down to adherence and acceptance, if all us listen and take to what the medical practitioners say the Ebola sick will come to an end, if we don’t, well we will continue suffering, the issue of bats, yes because any season we have bats, so ?it? true that we get Ebola from bats, all the question should be answer by the government, I don’t know and don’t want to lie”.

M: What do you feel you need to know to responds to more effectively?

R: “Government needs to be getting inform with any new information about Ebola, ?well? should be trained how to answer some of these question”.

M: Is there anything specific about Ebola that you think people need to understand better?

R: “I want to understand if there are any other signs and symptoms of Ebola despite the ones shared in common with malaria, and which mechanism government is putting in place to protect the school pupils after resuming schools. I as a parent, I have my last child in the high school, for us not to forget about Ebola, I want this Ebola virus disease to be part of the school curricula, teachers are educated people, they may able to pass the message to the pupils through teaching, if possible let write a book or pamphlet on Ebola, we don’t need to joke with this things, it is an after Ebola preparation. Let us retain the hospital and make then active, the sick will to not disappear like that, we need to be active, and let’s be sensitizing the community about Ebola especially our sanitary officers, let the government give them a technical training, they will be around the community sensitizing the people about the prevention of the reoccurrence of this Ebola sick, it happens in Congo, let God protect us that. It has really gets difficult, the challenges about Ebola is many, so let it be part of the school work, college work, let’s us be aware of Ebola, the other thing again, there should be continuity in Ebola prevention activities, like the washing of hands at checking point, avoid the body contact, let every schools has the wash hands point not only during the time of Ebola, let the washing of hands continue, prevent the little children from playing on the ground”.

M: What do you think would be a way to explain this to people?

R: “Well, I will explain to them that, there are no others signs and symptoms yet, unless the same ones, and I will also tell them that government will be putting better measures in place to protect the school pupils, by providing thermometer, soap and water in schools for washing hands and many more”.

M: I thank you very much for taking your time

R: “It is a pleasure”.

**ADDITIONAL PART OF INTERVIEW, OBTAINED BY COLLECTOR 2 AFTER CONSENT IN PERSON, March 2015:**

M: Yes sir, the last time when my colleague was interviewing you, you said people were afraid to play with their companions, what do you mean?

R: “Yeah… when people have gone, for instance your own wife and child, when I went to up country and came back, the child told her mother, I am afraid of papa (father), I am not sick but she is afraid, that means, she is afraid of Ebola, she had the feeling that I had contacted it from somewhere and I have come back at the house, this was a time when the child was afraid of the mother, mother was afraid of the child, papa(father) was afraid of the child, papa(father) was afraid of the mother and the mother was afraid of the Papa(Father), that was what it means, the child feels that I had caught it somewhere and I had come to give them”.

M: So the child do not go near, touched or played with you?

R: “The child does not even go closer to me”.

M: What do you think, when people were denying that Ebola exist?

R: “They were denying that Ebola exist, what were the reasons”?

M: Yes?

R: “One, our society hardly believed these things, what I am telling, plainly we are superstitious, sincerely speaking, Sierra Leonean men don’t even believe virus and bacterial, those kind of things. What they believe, witchcraft or other things they think about (*people talking at the background)*, and they had the feeling that witchcraft gave them sickness not virus”.

M: Ok, so that is what they were denying?

R: “Yes, is witchcraft they knew”.

M: Since the people denied at first, what made them to believe now that Ebola exist?

R: “Why they believe that it exist”?

M: Yes, why they changed their minds that Ebola exist?

R: “What the health practitioners are telling us, the number of person(s) that died per house is hardly thirty, forty or twenty, when they saw these things happening, people were dying, they saw the Whiteman that came to fight this thing, they saw the health practitioners like doctors, nurses are sensitizing, the radio had told them, they saw posters on the wall, that is the time some people got to believe that hmm this thing is true, they have heard it on radio, in fact this the time, it has never happened in this country, when they are stigmatised somebody, that don’t come here because your country have Ebola, for example, nobody went to Hajj or Mecca this year or last year because of Ebola”.

M: So this is the time they changed their minds?

R: “Yes, that this thing (Ebola) is real”.

M: What time or month was that?

R: “Towards October, the time for hajj, October, December, January after the rainy Season, between the rainy season and the dry season”.

M: October, November?

R: “Yes, when some people did not go, they said “aaaa” it is there we don’t need to joke, this is not a thing to joke with”.

M: Do you remember the first time, date that you had the first Ebola case in this community?

R: “Our own community”?

M: Yes?

R: “Hmmm, Yes I can remember the actual date but”

M: Like Which Month?

R: “Around August, we were hearing rumour that it is here, it is around us, it is in (- - name of a town in neighbouring district - -), hence it is that in that area, it will come to (- - name of the city of the interview community - -)”.

M: So people do not believe in this community that Ebola exist, unless they heard of these things in these areas?

R: “Yes, they started seeing people dying; it is very difficult to see people dying in that kind of large amount, it has not been happening”.

M: Why do people still not believe Ebola exists?

R: “Some still had that denial, let me tell there is bad politics in this country, some people think that those that are in Governance brought everything, they are still denying that it is politics and this and that, I will not hide it. Because I want Ebola to finish now, I will not hide anything I had seen for myself”.

M: So it is because of the political ideology that the people do not believe Ebola exist?

R:”Yes that Ebola does not exist, the other thing again, let me tell you point blank, Sierra Leonean does not believe germs, they believe “juju”(sorcery) that any death, they will attribute it to witchcraft, juju in fact even accident , they will not believe, you people just forget about it everybody will not believe that Ebola is real. Maybe most or some may believe”.

M: Last you spoke of “Debola” what language is that?

R: “Well it is just a name that comes up in societies, they called it “Debola”.

M: hmmmm?

R: “They call it again “Boola”

M: why?

R: “For instance, a boy touched a corpse, they took the corpse and they came laid it, after which, he was shunned by all the people, which gave him stigma, he was there without friends, whenever he was passing by, his companion they were provoking him, shouting at him “Boola-Boola-Boola”

M: Because he touched the dead corpse?

R: “Yes, because he touched the dead corpse, they took the corpse from their house and brought it nearer to our place, so they were calling “Boola”, some “Deboola, some called it Boola, Boola”.

M: So the Debola do not have any language?

R: “It is from this same word “Debola, Boola, some even called it witch plane (*respondent laugh*)”.

M: “You said last that some prefer going to the traditional healers, why do you think this is?

R: “Yes, one, they have been used to them, two, some people hide sickness, so this people prefer to go to a place where nobody will see them that could be another reason, the other reason I think, people have heard that if you got this sickness and go to the treatment centre, they will kill you, they will spray you, that was what everybody had in mind that when you are sick and go to the hospital, they will spray you with chlorine, some people thoughts that the chlorine kills”.

M: so because of that, they prefer going to the tradition healer?

R: “Yes, where they will not spray them. A boy around here, he is sitting over there, said, when he was loaded in the ambulance, when they arrived , before he climb down the ambulance, they sprayed and he was right in there. How can you spray a person? Life means something”. (*An undue noise at the background*)

M: Do you know any person(s) that has gone to a traditional healer for cure?

R: “I can’t tell you now, that if it has happened here, because we don’t have them around, some people went far off in places like (- - name of town- -) for healing, but I have not seen it”.

M: Do you have an example of that?

R: I don’t have an example, some were afraid because of the spray of chlorine, others were afraid of the rumour that when you go to the treatment centre, you may not come again, so instead they prefer going to traditional healers”.

M: You said again, some people prefer staying at home and take treatments. What kind of treatment do they take?

R: “They bought Panadol and other sets of tablets for treating themselves, I have seen it myself on television (TV), at (- - name of hospital- -), a sick person was laying down helplessly, there were no medical practitioners to attend to that sick person, the thing was very hot at that time everybody was confused, some prefer to stay at home and take Panadol, this means they were pepper doctoring (illegally / unqualified treating) themselves “

M: Do you know, seen anybody that had done the pepper doctoring (= unqualified / illegally treating) in this community?

R: “For any sickness”

M: Yes?

R: “These are the normal things we do, myself, I was sick of malaria, I did not go anywhere, and my wife is nurse, will I go to (- - name of hospital - -) and waste time, I prefer pepper doctoring(illegally treating) myself or buy Panadol and drink”.

M: You said last people were making false calls to the ambulance service, what do you mean?

R: “Yeah, some people will just sat down and falsely called the ambulance, when the ambulance service responds, they will come but will not meet or neither see any person(s) that made the call, it happened in this community, someone died up here, they called the ambulance, when the ambulance came, the people had taken the dead body somewhere else, so called it as false call, it happened again at (- - name of a road junction- -), some boys because they knew the 117 phone line, they called the ambulance and they came, but it was a false called, because there was no case of Ebola, so the nurse were very annoyed saying they have just wasted their time”.

M: Why do you think they were doing this?

R: “One example, the people called but the ambulance service does not respond quickly, so they moved with dead corpse”.

M: So mean the ambulance delayed to come?

R: “Yes I think so, it has been happening before, when someone died, they will call ambulance, it may take two to three days before the ambulance comes, and the other thing gain, some boys in this country like testing or they like making fun out of something which is very important”.

M: You said some people are “tranga ace” (= stubborn), what do you mean?

R: “Good, for instance, the medical people had told us not to wash dead bodies, let us don’t even touch, like that boy it was due to his “tranga ace” (= stubbornness), he grasped the dead, I don’t know if it was Ebola or not, of course the boy did not die, that showed it was not Ebola dead but once we had being told not to touch dead bodies of all type, you may not know the cause of the death. It was actually interesting, if I explained, you will laugh, the boy was holding a bottle of rum in his hands, and he was drunk to stupor, so he grabbed the dead body and laid it, and said he wants to give the dead body oxygen”. (*Respondent laugh)*

M: Given Oxygen to the dead body?

R: “Yes, that is true, it is not a story, I was not around, I went to town, when I came back, they explained to me, and the boy did not die, he is alive”.

M: Have you heard about any secret burial?

R: “In this area”?

M: Yes, or in other communities?

R: “Well yes, they were doing it, I have it once and twice when I was travelling from (- - name of town- -), I heard it in (- -name of town -- ), they buried someone and they exhumed body, they said the spirit of dead person was disturbing them, these are all rumours, I was not there, they exhumed and buried the dead body in their own way. Some people are doing secret burial, they are doing secret burials traditionally, society also does bury secretly, that is the reason my sister, they said the person is a secret society man and society man is not easily buried, so we learnt we were a child, only the members of that particular society may involve in the laying out”. (*Respondent laugh*)

M: What do people talk about the Ebola survivors in your community?

R: “I had contacted Lassa fever, I am a Lassa fever survivor, when I came back from hospital people were afraid of me, I had a neighbour who is a diamond dealer, he buys diamonds, he was afraid of me, whenever I went nearer to him, they were saying that man is sick of this particular sickness, even the time you were not born, there were illnesses like the small pox, people were trumpeting it that we had a big sick. They will be talking about you”.

M: What are the problems the Ebola survivors experiencing in this community?

R: “People are moving away from them”.

M: Stigmatizing them?

R: “Yes, in fact we have survivors here, a women and her child, people are even looking upon them as bad people, they said the woman and child were having the sickness but did not tell them, they all have that grudge. This particular issues you people really need to fight it, it is not easy let me don’t lie to you”.

M: So is it the same in other communities or not?

R: “Maybe it is worst in the other communities, for instance here is a city, people are not mindful of some petty things, if it is remote towns, people will not even eat with you, myself I went to my village, they said we in (- - name of city - -) give birth to Ebola, they are afraid of us, even when you went there in good health, they will not come nearer to you. The place where I established a small business enterprise, the distance is like from here to (- - name of place - -), I have gone to my home town twice, but can’t reach my village because of Ebola. If I go there, anything that happens they may say I took the sickness from (- - name of city - -) into my village, let me don’t hide it from you, some people do not even accept them”.

M: So they have not fully been accepted in the communities?

R: “In fact my sister, when you visit some communities, you will first greet and report yourself to the town chief to be aware of your visit , the section chief has to recognise your visits with money likewise the paramount chief. I heard it on the radio when a paramount announces that, if you are an indigene of his town and you are presently there, you must not go there until after Ebola or if you go there, we will be in the vehicle and returned, you will not climb down. That has been happening in these villages, they thought we that travelled into these villages had Ebola”.

M: Even when you are cured form the sickness, will they think that you still have the sick?

R: “Even a person that does not have the sickness, you wouldn’t be accepted in the community, there are places, I have also heard a female paramount chief announced that let everybody stay at the place where he or she is at the moment until the end of Ebola, believe me my sister, it is no joke”.
