# Supplementary material for: Development of a set of community-informed Ebola messages for Sierra Leone
Source: PLoS Negl Trop Dis. 2017 Aug 7;11(8):e0005742. doi: 10.1371/journal.pntd.0005742 (PMC5560759; doi:10.1371/journal.pntd.0005742)
Supplement: S1 Appendix — (ZIP) [file pntd.0005742.s001.zip › Ebola messages - FGD and interview transcripts/R2HC Ebola Fieldwork 1/R2HC Ebola F1 COM-Urban2.docx]

| CODE | **R2HC Ebola F1 COM-Urban2 (urban semi-structured interview with community leader)** |
| --- | --- |
| DATE | February 2015 |
| DURATION (minutes) | 51 |
| Collector nr | 4 |
| LANGUAGE INTERVIEW | Krio |

**PERSONAL DATA RESPONDENT**

| Age *(in whole years)* | 47 |
| --- | --- |
| Sex (F = Female, M= Male) | Female |
| Religion | Christian |
| How much time does it take you to walk from your house to the nearest PHU? (minutes) | 20 |
| Mother tongue: | Mende |
| Education level: | Secondary |
| Role in community: | Women’s Leader / Nurse with drugstore |
| Do you know anybody who had Ebola? | Yes |
| If Yes, what is your relation to that person? | Neighbour |

**TRANSCRIPT: (M= Moderator, R=Respondent)**

M: when did you first hear about Ebola?

R: “I first heard of Ebola on the radio in May 2014”.

M: How was this sickness described to you?

R: “Well the only time they described it to us, was in a meeting held by the Pharmacy Board, they told us that Ebola is caused by a deadly virus and there are no medicines for it”.

M: What was your first thought about it?

R: “It just created much fear in me, because I thought that I am a business woman and I interact with so many people, and I am dealing in medicine, people may come for treatment or come with prescription to buy, you must have to interact that person. It was wondering and afraid”.

M: In what ways has Ebola affected your community?

R: “Ebola has affected us in many ways, one, our children schooling, some of us have girl child, they have grown so big, and they have stay at home over two academic terms. Even a daughter of a close door neighbour, I was the one counselling her to attend church services all this time, but these few days I notice that she is pregnant. This was because they were not going to school, idling, going about other business. So most of these girls have become pregnant and the boys if they are not strong with the grace of God, they will join companies of bad guys”.

M: Have you personally seen or known people who have had Ebola?

R: “I have not yet seen or known any person who have had Ebola, unless the ones we hear on radio, that one neighbour has been confirmed Ebola positive and has been taken to the treatment, two people have died of Ebola. Like we I heard of one person that have survive, but presently the person can’t return home, because the house is under quarantine”.

M: Why do you think Ebola has spread throughout the country?

R: “One of the reasons, in (- - interview district - -) people do not take control and most of them do not listen and take to advice. When they advise them, that they should always wash hands, avoid body contact but some still continue to play with each other, even sometimes at the junction were my shop is, I stop them from playing with each other. Some of this people ignore the fact, they are still touching and playing with each other, move and move around, they don’t care to know”.

M: which ways do you think will prevent Ebola from spreading?

R: “For me, I think they have to make the bye-laws works more effective, because people are still holding on their traditions, washing dead bodies, like yesterday I heard over FM radio 98.1, at Waterloo some people washed a person that die, dress and put in a coffin, later call 117, they even called because they have announced that all burials should be undertaken by the Ebola burial. So if people in the (- - interview district - -) are doing this, what do you we expect from people in the remote villages? But if the bye-laws are not firmed they will still continue with their traditional practices, because some people are saying, when someone dies should be buried with respect, and once someone had die no amount of respect will bring the person back to life, or gained that die person heaven or heal, God is the only one that decides, due to your good work of that person”.

M: What do you think is the best way to treat somebody with Ebola?

R: “Well if you noticed that someone around has Ebola, avoid the body contact with that person, two, make sure you call 117 to send ambulance to come collect and take the person to the holding centre. Then, they have to give more health education to the people, so that they will have the awareness”.

M: Are there any local names or terms that people use to describe Ebola in this your community?

R: “Well, I heard our people call it “fitina hinbgeh” (= disaster or trouble sick)”.

M: What do you mean by that?

R: “Disaster or trouble sick that has come”.

M: Some people do not believe Ebola exist, do you have those people in this community?

R: “Yes, most of this people are the are the youths, I remember a boy came to buy a medicine, the boy met us discussing about Ebola, there were poster of Ebola signs, symptoms and precaution which they gave to me, and I was sensitizing the people to avoid body contact, wash your hands, this boy refuse to wash his hands, and want to enter the shop, I told the boy if you don’t wash your hands don’t enter into my shop, the boy even alter a statement that he does not believe Ebola exist, then I said him, you are saying at this very moment that Ebola do not exist, that is why you are quickly get infected, because you continues play with each other”.

M: Do you know why they view it this way?

R: “It is just because they are not under controll”.

M: Please can you give some examples of Ebola messages that you have seen, heard or read?

R: “they gave us poster, which shows the signs and symptoms like diarrhoea, vomiting, bleeding, and rashes and it is indicated that if you experience those signs, you have to go the hospital, other organisation like Red Cross, IRC, came and sensitized and ask the people to bring out sick persons and go with them for treatment”.

M: What do you think of those messages?

R: “These messages are really in place, but more needs to be done”.

M: What needs to be done as you said?

R: “They should target the youth’s groups and traditional healer with proper sensitization, because these two categories of people are contributing to the spread of this sick”.

M: What about the way of disseminating these messages?

R: “That is another one again, because some people you have to use their local languages to disseminate the message, again like how me and you are talking now, one on one. By using the one to one talks with a people, that persons will understand better”.

M: What do you think is the best Ebola messages you have come across to date?

R: “Well the best one is the one that Red Cross did, they came to my house and talk to us, even they knew I am a medical person they did not look to that they still continues”.

M: So you are the women’s leader, and at the same time a medical practitioner?

R: “Yes I am a nurse and I run a drug store in this community, so they announced their motive of coming, and started talking about Ebola. They ask if we believe that Ebola, they ask us if we have a sick person. So they gave us advices”.

M: So among these advices, which one you think is the best?

R: “Well the best one is to avoid body contact and the hands washing, I want this to be part of us, because it has not been part of our culture. But even after Ebola we continue these ones, it will help a lot”.

M: Are there any Ebola message you think they have not worked so well?

R: “Yes, because some of these messages show on the television, not everybody has access and not every will understand”.

M: what I mean, are there any Ebola message you think they have not worked so well?

R: “To my own knowledge all is working, I don’t know for other people”.

M: What do you think would be a good message to encourage people to bring patients to the treatment centre?

R: “I want the government to be using the religious leaders to pass on the messages, to tell people the different between the living and the dead, if people are aware of this, they should not be given priority to the dead forgotten them that has lives”.

M: What I am asking, what do you think would be a good message to encourage people to bring patients to the treatment centre?

R: “Well you have to give them the assurance that when they are taken to treatment centre they will be cured and their lives will be saved, even if the person has Ebola.”

M: In the event of Ebola infection, do you think that people will prefer to go first to a traditional healer or the existing health facilities/staff or the Ebola treatment centres?

R: “Most times when people fall sick, they prefer going to the health facilities in their community?

M: Why do you think this is?

R: “Because usually, when a person have headache, he or she will say let me go and buy medicine for head ache, so when they come to the health facility to report head ache, you will further enquire, from the person for other illness”.

M: Some people stay at home, when they think they may have Ebola, why do you think this is?

R: “Because they are afraid, they think that when they go out, they will died, and the Medical people said, there is no medicines for Ebola and people are dying every day of Ebola. Some of these people have the conviction that, there are other illnesses that have the same signs of Ebola”.

M: So what do you think will be the best way we should do, to encourage them to come to the treatment centre?

R: “Let there be good health facilities, Good care, some person will be taken the treatment but they need special care and also, let them have mobile phones to communicate with members of their family, so they will feel at home. Because some people think that, ones they are there, they are no more considered”.

M: What do you think would be the best channel to get your new Ebola Messages to the people?

R: “Let’s be using our traditional languages and the one to one Ebola talk”.

M: why do you think we should use our traditional languages?

R: Because the layman will understand and feel at home when listening to the language he or she speaks and understands better, he or she will be open up to you, and give you all necessary views”.

M: So what about the one to one interview you spoke about?

R: “Because the person will be comfortable to talk and share better views with you”.

M: Have you ever heard people talking good way about the ambulance services?

R: “Well the good way about the ambulance, it will stop the patient to join public transport to go to both the holding centre and the treatment, so that the person will not transfer the sick to other people. The bad way, when the ambulance comes to collect a patient, everybody will come to see that this particular person has Ebola, it presence will draw the attention of people, moreover the siren of ambulance when it around, every will be eager to know the house that ambulance is go to stop, and they will avoid that particular family because they have Ebola, even this behaviour traumatized that family”.

M: Have you ever heard people talking good way about the holding centre?

R: “Well like how I just said, I have not got closed contact or talk with any person, that have gone there and came back survived, unless that of our neighbour who is supposed to come home after surviving from this disease. I don’t have any idea about that”.

M: But apart from that, have you not heard other people talking about them?

R: “I don’t care, but some people are talking, they say the treatment there is not good, the medical people don’t even come near you, fearing of getting Ebola

M: I want you to tell me the good and bad thing you heard about the burial team?

R: “ well the good thing about the burial team, an incident occurred in our area, were a boy of 35 years get sick, and his parent were treating this guy privately, until the sick get worst now and they decided to take the boy to the hospital, on their way, he die under an electric pole along the road, so they called the burial team, they came and ask, later they sprayed the entire area where he was laying, put him into the body bag, put him in the ambulance and leave, so by disinfecting that particular gave people the confidence to walk along that side”.

M: What about the bad aspect?

R: “Well I have never heard of it, people just say they are aggressive when they are in action”.

M: Do you know about any secret burial in this community?

R: “We have never heard the and I has never happen in this community, (- - name of the interview community - - ) is an opened community, and in fact there is no land to burial do secret burials”.

M: What about the good and the bad talks about the 117 phone line?

R: “At the begin the responds was poor, people will call and call, they will not responds, even a boy died here, they call them many times, they did not respond early, until later they came and collect. But now, it is far better, as you call them, it will not even take 10mins they will be at your location”.

M: Any aspects of the existing health facilities/staff that is working on the care and treatment?

R: “Well more the government health facilities, the staffs and other medical practitioner don’t behave well to people, I have suffer that situations, though am a nurse, my mother was sick I told her to the hospital, after giving me the prescriptions paper, I went to drug store, I met a nurse, she did not even talk to me in any good manner”.

M: What about staffs working at the Ebola treatment centre?

R: “No, there is no bad about them, the last talk I heard from them, was praises, that they are doing very well”.

M: How do people treat Ebola Survivors in this community?

R: “Well in this community we don’t have any Ebola survivor, unless a pending Ebola survive case, which will be discharging from the treatment centre this weekend, so I don’t know how the community will act to them”.

M: But how do you think people may act to the person when she come in your community?

R: “Well few people were saying that, there was no Ebola in this community, so this lady went to waterloo to take care of her sister, because she is a nurse, were she was infected and she did report early unless she started showing signs, them her husband force her to go to the hospital, so people were not happy with her especially the ones under quarantined now, so I don’t know may happen”.

M: Have you heard of any new treatment for Ebola that may become available soon?

R: “Yes, we have heard of vaccines to become available, don’t know how true that is”.

M: How do you think people may be thinking about the vaccines?

R: “They are afraid right now, as their minds has been poisoned, that the vaccines itself has the virus. I ask one day that if this vaccines, what category of people will be given the vaccines, they said the people that have not infected with the virus, so people said they want to infect the remaining. So with these ideas, they should sensitize them thoroughly”.

M: Have you heard of any new way to prevent Ebola?

R: “Well the only new way is when you are travelling in public transport, used long sleeve, don’t expose your body”

M: As a women’s leader and health worker, what are the common questions ask by your people in this community?

R: “Sometime they ask, if this sick exist and it is real, some don’t believe, when I said yes, they will ask again, why now we are experiencing it, some people don’t believe, they just believe that it is a bio-weapon or chemical, but since we have documentary of this Ebola from Zaire. I was also explaining that scenario to them, and I even delivered talk on Ebola in one community sensitizing meeting, so I explained most important part on this video clip, telling them a hunter went to hunt and came in contact with a animals so he was later infected with Ebola. So we got Ebola, for Monkeys, bats etc.

M: So how do you respond to those questions?

R: “I always come down and tell them the true”.

M: Is there anything specific about Ebola you may want to know, so that you will be responds to questions better?

R: “What I want to know, when you are expecting the treatment for this Ebola sick, like when HIV came there was no medicine, but later there was treatment for it. So if we have that same type it would be better”.

M: is there anything specific about Ebola that you think people need to understand better?

R: “What really people want to know, how Ebola was originated in Sierra Leone. Even when they are telling them, it is man that went to Guinea and Liberia to cure a sick person and later brought the virus to Sierra Leone, they don’t believe”.

M: Which way you think should be explained to them for better understanding?

R: “Well the Ebola survivors should serve as witness to those people, because if they even what they have gone through, people will believe”.

M: Thank you very much for taking your time.
