# Supplementary material for: Development of a set of community-informed Ebola messages for Sierra Leone
Source: PLoS Negl Trop Dis. 2017 Aug 7;11(8):e0005742. doi: 10.1371/journal.pntd.0005742 (PMC5560759; doi:10.1371/journal.pntd.0005742)
Supplement: S1 Appendix — (ZIP) [file pntd.0005742.s001.zip › Ebola messages - FGD and interview transcripts/R2HC Ebola Fieldwork 1/R2HC Ebola F1 COM-Urban3 V2 ADD PROBE.docx]

| CODE | **R2HC Ebola F1 COM-Urban3 (urban semi-structured interview with community leader)**  **V2 – 10^th^ March 2015 – ADD PROBE** |
| --- | --- |
| DATE | February 2015 |
| DURATION ( minutes) | 40 |
| Collector nr | 3 |
| LANGUAGE INTERVIEW | Krio |

**PERSONAL DATA RESPONDENT**

| Age *(in whole years)* | 42 |
| --- | --- |
| Sex (F = Female, M= Male) | M |
| Religion | Muslim |
| How much time does it take you to walk from your house to the nearest PHU? (minutes) | 15 |
| Mother tongue: | Temne |
| Education level: | Secondary |
| Role in community: | Imam |
| Do you know anybody who had Ebola? | No |
| If Yes, what is your relation to that person? |  |

**TRANSCRIPT: (M= Moderator, R= Respondent)**

M: When did you hear about Ebola for the first time?

R: “The first time I heard about Ebola, it has taken some months now, I can’t remember the months now, since we got Ebola”.

M: How many months now?

R: “Six months ago”.

M: How was the disease described to you?

R: “They described Ebola to me that, when you are infected with this virus, your body will become warm, you will start vomiting and frequent stooling (=diarrhoea). When you have started experiencing these signs, go to the hospital for check-up that was the first thing they told me about Ebola”.

M: What were your first thoughts about it?

R: “Well, I feel that, the people who tell us, know much about Ebola, we believe Ebola exist and we were following the precautions, anytime we experience signs of that nature we go to the hospital”.

M: How has Ebola affected your community?

R: “Ebola has affected our community a lot, there is no work again, there were people who works in restaurants, hotels, all of these has stopped, people doing private business has stopped, the up and down movement of people is not going on well again, up to this day, we don’t even gather together again for meetings in the community, to avoid interaction as they said, this has led to backward development of this community, people have really suffered”.

M: Do you personally know a person that has Ebola?

R: “Well in this community, we have not had anybody with Ebola, but there is one of our neighbours here, that they said has Ebola, but I have never seen the person. But I am sure it’s Ebola because they have quarantined the house and they have taken them to the centre”.

M: Why do you think Ebola has spread throughout Sierra Leone?

R:”When Ebola breakout, the Doctors said, we don’t have to interact, we don’t have to touch dead bodies, no handshaking, when Ebola came, we were told of all these precautions, but most people did not still believe. They did that and it has affected them greatly”.

M: What do you think is the best way to prevent Ebola from spreading?

R: “Since Ebola breakout, Doctors gave the precautions, and we as religious leaders in our community, during the Friday prayers were so many worships gathered at mosque, standing at the “Meba” (=pulpit) in delivering sermons, we have been telling them that as we have been directed, to prevent the spread of Ebola, if a person has die, don’t touch that person, take a sick patient to the hospital early, when someone die, don’t wash the person, you don’t need to do anything, unless the Ebola burial teams or specialist in Ebola burial, that is their responsibility. So any person that insist touching and washing dead bodies will be infected with the virus. We were passing on those messages to the people and I believe now a lot of them are aware and conscious, because the number of cases as reduced. Most of the messages are passed on during Friday prayers at the pulpit we stand, when there are a lot of people at the mosque, we do it morning and evening”.

M: What do you think is the best way to treat somebody with Ebola?

R: “Well I think the best way to cure a person with Ebola is, when you go to the doctor, it is the doctor who knows how to treat you because he knows best and better but we know that when somebody gets Ebola, we should accept him or her in our community”.

M: In the community there is no way you think to treat a person with Ebola before going to doctor?

R: “No, you have understand that there is nothing to do in the community with a person that has Ebola, unless we go to the treatment centre, they are the only people that give the perfect treatment, but there is no private Doctor at the centre who cure Ebola unless the treatment centre, they know much”.

M: Are there any local terms that you use to describe in your language?

R: “Ehh, No, as it is called Ebola, so we also call it, I am a Temne by tribe, but we have never come across this type of sickness in the Temne community. Saying if I know another name for it, no because this our first time of getting this type of sick, so we don’t have another name for it in our language”.

M: Some people do not believe exist. Do you know people in your community who think so and why?

R: “Well yes, because we have never come across the sick in this country, this is the first time for everyone to hear of it, so this is reason most of the people do not believe Ebola exist, only until when it became worst and the doctors started the sensitizing. At that people get to believe that truly Ebola exists, because it kills a lot of people”.

M: Can you tell me the set of people that have this believe?

R: “When it started, the older people believe it existence as they know it is a sick that transfer, the younger people do not believe because they were always up and down until later when the sick started killing a whole lot of persons before they believed in the existence of the sick

M: please can you give some examples of the Ebola messages you have heard, seen or heard? What do you think about these messages?

R: “Yes, yes, ehhh, we always hear the messages from various medical personnel sensitising us that Ebola exist and it is real, according to the doctors, we should not touch, we should not wash dead bodies, no hand shaking no hugging and should stay one by one, no meeting and gathering together. So all those messages we listened and heard them, then relay it to our community people and also to the Muslims who come to the mosque”.

M: What do you think about those messages?

R: ‘’Well, we believe they are true messages because they are coming from either doctors or nurses and that is their job so we hope that whatever they tell us is the truth and we see the performance of the sick, that is what we believe”.

M: Are the messages clear to you?

R: “Yeah, they are clear to me because the way the pass is to me is the same way to pass it to other people for us to understand that they are saying the truth not a lie at all”.

M: What do you think has been the best Ebola message you have come across to date?

R: “Well ehhh, the best Ebola message I think is the best I have come across is to wash dead body because it happened in my own eyes, to my family, this is very important, and when somebody is sick to take him or her to the hospital, this is also very important, to avoid body contact, and public gathering, these are all very important and I appreciate them so much because they are true”.

M: So what Ebola message do you think have not worked so well?

R:”Well, those that worked well are the ones I have told you already, those that did not work well I have not come across them, and all those I told you about worked very well, yeah.

M: What do you think would be a good message to encourage people to bring patients to a treatment, or holding or community care centre?

R: Well ehhhh, what would be a good message to those people unless when a patient is sick, because when one fall sick he or she loses his or her senses so you who is healthy should encourage them to go to the hospital or a health centre by cajoling them that going to the doctor is the best place and staying home is more dangerous for your health so going to the doctor is the best”.

M: In the event of Ebola infection, do you think people would prefer to go first to a traditional healer, to existing health facilities?

R: “Yes, because if something have come across the country like this one we are having now, and they have never witnessed it and before this time, it is a tradition for people to take sick person to traditional healers, some people in this country are afraid to go to the doctors, they believe in the traditional medicines. People were in conviction that this sick Ebola will be cure by a traditional healer, not knowing that only the medical doctor can cure Ebola, so these were the mistakes people did, which lead to death of many people”.

M: Some people stay at home when they think they may have Ebola. Why do you this can you tell me?

R: “They do not have the understanding, if they have the understanding that they have got a problem like that, and they have told that if you got this problem, so and so place you have to go to attack the sick, but still staying at home, it means they don’t have the understanding that, they have been infected with the Ebola virus. They continue staying at home until the sick get worst. So this was the reason for the death of many people”.

M: How do you think they get the understanding, so that will encourage them go to the treatment centre?

R: “To encourage them, the leaders of the community should have the understanding to talk to the people, never mind it is problem that is not easy, you have to talk in good a tone to them, because some people will still decide stay at home, unless you “bayo bayo”(=pamper) them, to tell them going to the treatment centre is best, but if you force the person to treatment the person will deny going never mind you are doing this for his or her own good, they will be having a different thinking”.

M: What do you think would be the best channel to get your new Ebola messages to your people in this community?

R: “Well to get the best and more messages that we have to follow, the “master sabi people” (= medical expert) still needs to be coming around and talk to the people what is happening”.

M: They should be coming to you?

R: “Yes they need to come to us and tell us what is happening, because has the disease is still around, so they will have more knowledge as to how they treat it. So anytime they have the knowledge, they should come around and sensitize the people in the community that will be the best for us”.

M: Ok, the medical experts have to come around to talk to the people?

R: “Yes, they should”.

M: As a community leader, have you ever heard people talking either good or bad about the Ebola ambulance service?

R: “The bad and the good talk about the ambulance, this ambulance is the thing people are afraid that the Ambulance contains an infecting spray which they believe could kill them once they enter it so most of them are afraid of it. Well, the good thing is that, those who understand know that government has provided the Ambulances to save people’s life so, when their relatives is sick, they rush to call for the Ambulance so they may live, such people are happy for the Ambulance.

M: Have you ever heard people talking, in either or positive or negative way about the holding or Treatment centres?

R: “Well, yes we hear both good and bad news, we heard that some patients live after been taken to the holding centre, while some dies, these two messages, we do come across them that most patients survives but majority dies again. So people need to take great care to survive because government has so done well to get us those centres.

M: What about the burial teams?

R: “Well, the burial teams, it looks like government did well but, where people did not have the understanding when the burial team was introduced in this country, there are two religions in this country, Islam and Christianity, and we also have a tradition that we all believe in, when a Muslim dies, they wash the body before burial, but when the burial team came, we saw that this was not happening again, no more washing, no more dressing the dead with “kasangay”(= white wrapper), prayers for the dead so this was very difficult for the Muslim and similarly so for the Christians but later, the government intervened call the religious leader and started giving preference to none Ebola deaths to be buried the proper way, now they started praying the dead, dressing and washing and they allow five family members to follow them to the cemetery”.

M: What about the very burial team they come, have you heard any good or bad thing about them, like they are either this type of man or woman?

R: “Well the burial team comprises of all men and we have not heard any good or bad talk, but they are so fearful. When they come to collect someone their dress and face appeared so fearful”.

M: But do the community accept them that way?

R: “Yes the community accepted because this is about government, when government has passed the law, there is no person to deny, after God is Government so they were accepted by all the people”.

M: Which bad or good talks you have heard people talking about the phone line 117?

R: “The 117 phone line, first when Ebola started, people were grumbling because dead bodies were not taken quickly for burial, they will be at the home of people for days before they come and collect, but as time goes by they have understand their work, when someone dies, they will call them and they will respond quickly and collect the body. So the bad talk was the late collections of dead bodies at homes, but now it is not happening”.

M: What about the existing health centres and the staff that is working on Ebola care and treatment centre any good or bad?

R: “In my community, I don’t know anybody of that nature”.

M: Among the Ebola treatment centre, which one work best?

R: “The Ebola Treatment centre that has done well is the Hastings treatment centre, because most people have survived from that centre”.

M: Why?

R: “Because many Ebola patient have been cured by the Hastings centre, so we took them granted, they are working”.

M: How are people in your community responding to Ebola survivors?

R: “In this community government has passed the law that nobody should provoke the Ebola Survivors and everyone is aware of that, so everyone in community encouraged and mingled with every man that have survived from Ebola”.

M: But before government passed the law, where people acting bad to them?

R: “When government passed the law it was not happening, but when there was no law they were acting badly to the Ebola survivors. So everybody obeyed and listen the decisions of the government”.

M: They are not stigmatized?

R: “No”

M: What will be your own message in advising other people not to stigmatize the Ebola Survivors?

R: “As God as destine us as Religious leader, we offer prayers for people, not only prayer but to sit down and give people the lectures, and the lectures will not be just in the mosque, that is why we have loud speaker machines to take the message far and wide, so everyone passing out there will hear all the lectures we undertake. This messages are given every day for people not forget not forget. We will not do it this week and next week we do it, no, it is a continuous process until this problem is finish in the country before we forget this message, but every day we are disseminating this message”.

M: Ok, have you heard of any new treatments of Ebola that may become available soon?

R: “Well, we have not heard of it?

M: Have you heard of any new ways to prevent Ebola?

R: “Well the only ways we know about, is the ways we have been taught by the doctors as government said and anytime they bring new messages to us, we are ready to follow it”.

M: In this community you don’t any ways to prevent Ebola?

R: “Well don’t have any way”

M:”I am talking of your own ways?

R: “Really as you have aware that Ebola exist and it is real, what we do, is what we are told by the “masta sabi pipul”(= medical experts), that we will follow, we will not mingle, don’t gather for meetings, we don’t touch, no shaking of hands like in previous days, after prayers we shakes hands, now we don’t shake hands gain. That is what we are doing to keep Ebola far away from us as what they told us about the sick is true, that is why we will continue to follow any ways message given to us on Ebola”.

M: Have you heard of any new vaccines for Ebola?

R: “No, they have not yet come with that message for us”

M: But have you not heard of it?

R: “No, I have not”.

M: What are the most common questions about you are faced with in your capacity as Imam?

R: “They always ask, why the doctors are saying, we are to wash our hands, no shaking of hands, no touching and the others, I usually tell them, Why we don’t have to touch because this sick Ebola transfer through by touching. Because when someone dies, the virus will transfer from that dead body to the person that is alive, that is the reason doctors said we should not touch, we should not wash dead bodies. They will ask again, why should not wash dead bodies, I having been telling then that, when you washed a dead person you must have to touch and if the person have this virus, it will transfer to you, and you will die also, that is why they we should don’t touch. They asked again about the no shaking of hands and told why you should shake our hands, because body to body is a contact, hence that has happened, you will get the sick. For instance if I have Ebola, when we get in contact, you will also get it. That’s lead to the spread of Ebola Sick, so that is why we should not do that”.

M: What do you feel you need to know more to enable you respond to those questions?

R:“What I needs to know comes from the medical Experts, because what you learn, is what you know, they know better than us, so any time they gave us new messages, so we implement to other people so they themselves we will know, to prevent Ebola from Spreading”.

M: What do you want to know from the medical people?

R: “What I want to from the medical expects because this is their professor. And every day their knowledge increases more. They know that the medicine they produce this year, is different from the one they produced the other year. They said Ebola do not have medical, but now they are curing people, it means they have got more knowledge. So how we progresses in this fight, the more knowledge they get and so they will come and pass it on to us, we pass it on to our subordinates”.

M: So the medical expert should come and talk to you?

R: “Yes”.

M: But what do you think you have to know from them?

R: “Medical Experts”?

M: Yes?

R: “When you are holding a book and I am not holding it, you holding the book knows the book more than me, like how Allah did, we have two books the Bible and the Quran. So what I don’t know, and what you have learnt, you know better than I do. So this doctors are learned medical people that know more about this sick, so the knowledge they are having, with me that is not having the knowledge, we are not the same, so they need to come and tell us new message about Ebola so that we will understand better”.

M: What is really specific about Ebola that you think people need to understand?

R: “We may like to know, where the problem of Ebola came from, what and why this sickness should come into our country and what are the ways that lead to this problem, we may want to know all those things from the Medical expects, because they know more”.

M: How to explain this to the people?

R: “Anytime we meet, we will tell them the same way as they told us, we may try to know what brought about this problem that we have never come across, everybody is in doubt and it leads to denier to each other, this said it this and the other person said no it is so. The medical people needs to research to find out the cause of the problem. So at that time if they gave us the message, we will disseminate the message

M: Thank you very much this is the end of the interview, so what is your last Word?

R: “We are pleading to the government to make available to the (- - name of interview community - -) community a hospital, that will help greatly the community”.

M: Ok thank you.

**ADDITIONAL PART OF INTERVIEW, OBTAINED BY COLLECTOR 2 AFTER CONSENT IN PERSON, March 2015:**

M:Yes sir, when my colleague spoke to you last, you said the people do not believe Ebola exists and you said, you don’t believe also Ebola exists, so I want to ask, what changed the minds of the people and you that now Ebola exists?

R: “Yes, I have seen people dying that is why I got to believe that Ebola is real”.

M: Yes you were talking?

R: “Yes, I came to believe that Ebola exists, when I saw Ebola killing people; that is the time, I said it is true”.

M: So you personally saw someone that die of Ebola?

R: “Yes,”

M: Ok that was the time you changed your mind from belief to disbelief, which date was that?

R: “I can’t remember the date”

M: Can you remember the month, when someone died that you see and know?

R: “It was around December”.

M: December, ok, it was the time you saw a person die of Ebola?

R: “Yes”.

M: So it is the time you changed your mind that, this people are saying the truth that Ebola is real?

R: “The person was not a different person but a relative”.

M: A relative?

R: “Yes a relative to me”.

M: What was your relation with the person?

R: “The person was a cousin to me”.

M: Ok, why the other people in this community came to believe now that Ebola is real?

R: “Because they saw Ebola, killing other people, so everybody got to believe that what they have been saying about Ebola is true”.

M: So when they saw people dying, this is the time they came to believe that Ebola is real?

R: “Yes”.

M: Have you seen people going to traditional healers for healing?

R: “Well, I have never seen that”.

M: So you have not seen anybody going to traditional healer?

R: “No, since the government has announced that when a person is sick should go to the hospital, I have not seen people going to the traditional healers”.

M: And you have not heard of anybody?

R: “No, I have not heard of anybody”.

M: Ok, you also said when some people are sick, they stayed home, why do you think this is?

R: “First when Ebola broke out, people were not going to hospital, they prefer staying at home when they are sick, because they heard a rumour that there is no medicine to cure Ebola, so do not go “.

M: You also said, people were treating Ebola survivors badly, what do you mean?

R: “Ok, people were thinking that these survivors were sick and they have healed them, these Ebola survivors had been looked upon as different people; that is what the people were afraid of”.

M: They said the Ebola survivors will be infecting them, when they go closer to them?

R: “Well it appears like after they were sick, taken to the treatment centre, when they came back survive, the people thinks that they had the thoughts that the Ebola survivors would transfer the virus to them”.

M: “Have you heard about secret washing of dead bodies in this community?

R: “No”

M: What about secret burial?

R: “No, since government had announced that no person(s) should wash dead bodies, when somebody dies, call 117, that was what I heard, but I have not heard of a secret burial or a secret washing of corpse even a single day, I have not heard of it ”.

M: Are they still treating Ebola survivors badly or they have changed and they are treating them well in the community?

R: “Yes, now it has changed, first when people do not have the understanding they were treating badly, but is ok, because they have understood that anybody that had sickness of Ebola, and had survive, they do not transfer the virus from another person. So the Ebola survivors are in the community, nobody is disturbing them, they are living comfortably”.
