# Supplementary material for: Development of a set of community-informed Ebola messages for Sierra Leone
Source: PLoS Negl Trop Dis. 2017 Aug 7;11(8):e0005742. doi: 10.1371/journal.pntd.0005742 (PMC5560759; doi:10.1371/journal.pntd.0005742)
Supplement: S1 Appendix — (ZIP) [file pntd.0005742.s001.zip › Ebola messages - FGD and interview transcripts/R2HC Ebola Fieldwork 1/R2HC Ebola F1 COM-Urban4 V2 ADD PROBE.docx]

| CODE | **R2HC Ebola F1 COM-Urban4 V2 ADD PROBE (urban semi-structured interview with community leader)**  **V2 – 11^th^ March 2015 – ADD PROBE** |
| --- | --- |
| DATE | February 2015 |
| DURATION (minutes) | 22 |
| Collector nr | 2 |
| LANGUAGE INTERVIEW | Krio |

**PERSONAL DATA RESPONDENT**

| Age *(in whole years)* | 32 |
| --- | --- |
| Sex (F = Female, M= Male) | M |
| Religion | Muslim |
| How much time does it take you to walk from your house to the nearest PHU? (minutes) | 5 |
| Mother tongue: | Temne |
| Education level: | Tertiary |
| Role in community: | Youth Leader |
| Do you know anybody who had Ebola? | Yes |
| If Yes, what is your relation to that person? | Friend |

**TRANSCRIPT: (M= Moderator, R=Respondent)**

M: When did you first hear about Ebola?

R: “The first time I heard of Ebola”?

M: Yes?

R: “do you mean the month or year”?

M: Both?

R: hmmm, I started hearing about Ebola in Sub-region in March 2014,

M: How was the Disease described to you?

R: “They described the sick to me as a sickness that kills and it has bad reaction, and we came to understand the signs and symptoms, before they said one of the signs and symptoms was bleeding, but we were not able to experience that symptom in our country, the others they told us were high body temperature, red eyes, frequent stooling, and vomiting were some of the signs and symptoms of Ebola”.

M: What were your first thoughts about?

R: “I was worried, because for a sickness that breakout to people that does not have medication, there will be no person that will be happy about it”.

M: In what ways has Ebola affected your community?

R: “This community suffered a lot from Ebola, there is an area called (- -name of area - -) in this community, this area was quarantined, over one hundred houses and two to three thousand people were in that community”.

M: Have you personally seen or known someone who have had Ebola?

R: “Yes, I have seen someone with Ebola”.

M: Why do you think Ebola has spread throughout Sierra Leone?

R: “The main reason why Ebola has spread is reluctance; people are not complying with the advice given by the medical people”.

M: What do you think is the best way to prevent Ebola from spreading?

R: “The best way to prevent Ebola from spreading again, because it has already spread, the health workers have their role to play, but the people have contributed greatly to the spread, just like what I told, is because the people are stubborn, but if they adhere to the rules and regulations given to them, Ebola will not take more than a week in this country”.

M: What do you think is the best way to treat somebody with Ebola?

R: “The person that has already infected with Ebola”?

M: Yes?

R: “The best way is very simple, call 117”.

M: Are there any local terms that people use to describe Ebola?

R: “Well no, there is no term for it, but only the youths in the community make fun by calling it “Ebo, Ebo”.

M: Some people do not believe Ebola exists. Do you know people in this community?

R: “Some of us had a confrontation with people, and I as am speaking to you, I believe there are people that do not believe Ebola exist”.

M: Do you know why they have this view?

R: “Because the signs and symptoms they associated with Ebola before it came to the sub region, Guinea and Liberia were not similar to the signs and symptoms in Sierra Leone, so the people were in total confusion, they were not convinced that it is the Ebola they were talking about, they think it was another plan, so that made them not to believe Ebola exists”.

M: Please can you give some examples of the Ebola messages that you heard, seen or read?

R: “I have heard a lot of messages, the musician do it through music by advising people how to prevent yourselves from Ebola, other people do it traditionally in disseminating the messages in the community in different ways, like the operation surge, that was a practical message”.

M: What are some of the examples of these messages?

R: “They are all prevention messages, like don’t touch someone that is sick, and the most dangerous one is don’t touch someone that has died from Ebola, if your mother, father, son or daughter is sick, don’t touch them”.

M: Are these messages clear to you?

R: “Yes, the messages clear enough, it is just left with the people”.

M: What do you think has been the best Ebola you have come across to date?

R: “The best message I have come across to date, is avoid body contact, this message has worked so well, even myself no matter our strong is cordiality I will not touch you and you will touch me, though some people get unpleased and annoyed over it, but we want to control the transmission of the virus, and we the people have to do it, no matter the cordiality between you and another person, don’t allow the person to touch, the reason is simple, you don’t if the person that is about to touch you has Ebola, or you yourself have the Ebola virus”.

M: Are there any Ebola messages that you think have NOT worked so well?

R: “The messages that have not worked so well, but it is happening in the villages, is about the issue burial, people still believe that they should bury their love ones”.

M: What do you think would be a good message to encourage people to bring patients to a treatment centre?

R: “Well I will advise them to be calm that it is not Ebola, that will courage the them, I will tell them not to touch the sick person but call 117.they will come and collect the patient and move him/her to the hospital, and they will administer free medication, when you say free, that will motivate the person”.

M: In the event of Ebola infection, do you think that people would prefer to first to a traditional healer, to existing health facilities/staff?

R: “Well it makes no sense if someone decide to go the traditional healer, because the traditional healer at this moment will not touch you, instead call 117 to take you to holding centre and they will carry out the test, if the result confirmed positive, you will be treated, but don’t allow the ambulance to come and collect you, walk and go for yourself. Early treatment saves life”.

M: Some people stay at home when they think they may have Ebola, why do you think this is?

R: “The reason is, it has been happening before, People are afraid, they said in process of taking them from home to the ambulance on the way to the hospital, even though they are not infected with the virus, they will be infected with the Ebola virus, and they had thoughts that the doctors, nurses, the medical people generally are going to kill them, that is the reasons they prefer to stay home and died”.

M: What do you think would be the best channel to get your new messages to the people?

R: “The best channel to use is WhatsApp, it is very fast in communicating, and communicating through the mobile, these are some the ways of communicating fastest”.

M: Is there any other way to communicate, rather than the mobile phone, because not everybody have access to mobile phone and WhatsApp?

R: “I believe that, these are the common ways to communicate in this community”.

M: Have you heard people talking, in either a good or bad way about the ambulance service?

R: “Before it was bad, but now it is good?

M: What is the bad one?

R: “Most times when Ebola started in Sierra Leone, we were having one holding and a treatment centre that was located in (- - name of town - -) in the process of taking people who showed signs and symptoms to the holding centre (- - name of town- -), most of the patients will end up dying because of the chlorine, according to reports from other people, the patients were suffocated by the chlorine before their arrival, most of them had died. These are the bad talk about the ambulance”.

M: What about the Good aspect?

R: “The good thing about them is, they moved the patients faster for treatment”.

M: What about the holding and/or treatment and/or the community care centre?

R: “I have not heard anything bad about the holding centre, people said they are treating them with care, the resistance of people, because they are grumbling about the chlorine, they said the percentage of chlorine mixture is high”.

M: What about the burial teams?

R: “Before when we got this outbreak, the knowledge was very small, with me personally I came showers blame on them because we have never got this type of disease in our country, so people were grumbling about their performance, but now it is quiet more better, when a person died, they will go there and talk to the relatives in a polite manner, tell them that they can join them in the carrying out the burial proceedings, but the only thing they should not touch the dead corpse, they will lead and the family members will follow”.

M: What about the Ebola phone line 117?

R: “The Ebola phone line 117 respond now to people but not immediately, because they are dealing with you alone, there are other people”.

M: Any aspect of the existing health facilities/ staff that is now working on the care and treatment?

R: “Now is better, unlike before when people were grumbling about the (- - name of a health facility / hospital in the interview district - -) hospital that they do not treat different people, that was really a bad news, but due to the comments of the people, they decided to leave it opened to everyone”.

M: Do you have Ebola survivors in this community?

R: “Yes, we have them”

M: How do people react to them in this community?

R: “As am speaking to you, I just spoke to one of them before coming here, I even gave him some assistance, and they are not stigmatizing them”

M: Have you heard of any new treatment for Ebola that may become available soon?

R: “No”.

M: Have you heard of any vaccines for Ebola that may be coming into the country soon?

R: I have heard it in Liberia but have not yet arrived in this country”.

M: Have you heard of any way to prevent Ebola?

R: “No”

M: What are the concerns of people about Ebola, their fears, misconceptions?

R: “For now most of the people feel that, if the people decide to put an end Ebola, between two to three days Ebola will end, if they are ready to accept the measures of the medical people that is the discussions presently”.

M: They don’t have any question they asked?

R: “They usually asked about the daily update result of Ebola, our best result is four and the other day fifteen, twenty, thirty, it is really confusing”.

M: Is there anything specific about Ebola that you think people need to understand better?

R: “People have belief that when you get Ebola, you will survive it, and when you survive from Ebola, you will no more be infected again with the virus”.

**ADDITIONAL PART OF INTERVIEW, OBTAINED BY COLLECTOR 2 AFTER CONSENT IN PERSON, March 2015:**

M: The last you said the people were reluctant to comply with the advice given to them, I want to know, why are the people reluctant to comply with the advices given to them?

R: “To my own understanding, is about their beliefs”.

M: What do they believe?

R: “Most people do not believe Ebola exists”

M: Why they do not believe that Ebola exists?

R: “The messages that is circulating around”.

M: About what?

R: “That Ebola is something made by a man, Ebola is not real, it is about money finding, most people get convinced of what other people told them, that it is true”.

M: What do they mean by money finding?

R: “They viewed it on a political perspectives”

M: But later they changed their thoughts that Ebola is real, what led to these changes?

R: “Maybe some had seen it or experienced it from another people”.

M: Ok, maybe someone had die nearer to them?

R: “Yes, maybe one person had contacted it and end up infecting the whole family and it really happened and it was something sure”.

M: “That was the time they changed?

R: “Yes”.

M: What was the date these changes happened?

R: “I can’t remember”.

M: What about the month?

R: “I can remember either the month or the date”.

M: You can’t even remember a month in last year?

R: “Uhmmm, last year, around October to November, the time we had high cases”.

M: So during this time people got to believe?

R: “People were afraid, I knew one old man down (- - name of community- -), he was sitting down with his children and one of the child vomits, he was the very man that called 117 until they did test on the child and find out that the child was colic”.

M: Them you said people were calling it Ebo, Ebo, what do you mean?

R: “It was just the form of calling Ebola”.

M: Then you said it was another plan, what do you mean?

R: “Plan, what do you mean”?

M: The last time you said it was another plan, what do you mean?

R: “Before were thinking that this Ebola thing is not natural and it is not, people are thinking based on what they had been told before as I told you last, and Ebola signs and symptoms are one, you had big scar on your body, swelling all over your body and this signs do not resembles with they were told, so that brought the big doubts among the people”.

M: You said again, people had that thoughts they must bury their ones, why do you think this is?

R: “Traditionally they believe and I am also part of the traditions that believes that before, that when you loved ones died you must buried them, but it came to a time when government stopped it, so I just felt that it is necessary and all other people thoughts it necessary that when someone dies you should call the burial team”.

M: Have you heard of any secret burial or secret washing of dead bodies?

R: “No”.

M: You not heard it in the other communities?

R: “No”.

M: Then you said that, it does not make sense to go to the traditional healer, what do you mean?

R: “It does not make sense for”?

M: The last I asked you that when someone is sick, where the person prefer to go, either to the traditional healer or the hospitals, so you said it does not make sense to go to the traditional healers, what do you mean?

R: “The traditional healer do not diagnose whether the sickness is Ebola or not and that is the first thing, most people that are sick and taken to the treatment centres do not mean that they are positive already but they will take your blood sample and test you if at all you had Ebola or not, if you had Ebola, they will treat you, so if you go to the traditional healer, how will you know that you don’t have Ebola, so it does not make sense”.

M: In this community, have you heard of anybody going to the traditional healer?

R: “No, until the time we had this outbreak, a woman came from (- -name of town- -), she came on a business trip, but she was pregnant, stomach was aching whole of the night, she tried but could not give birth, they took her to a woman called (- -name of a woman- -), she is a traditional healer, the pregnant woman went for cure but not knowing that one of the signs and symptoms of Ebola when you are pregnant is blockage and you are not able to deliver on your own, they took the pregnant woman down, they tried and tried, but you the tradition, they said evil people are fighting the pregnant woman and at the end of the day, they all died ”.

M: The people at the house and the ones that came to touch her?

R: “More than fifteen people died”.

M: But they do not stigmatized the Ebola survivors in the community?

R: “No”.

M: If you were an Ebola survivor, which problems you will face within the community?

R: “In this community we are accommodating them, and do not have no way of stigmatizing them”.

M: “When they come back, are they treating them bad?

R: “No”.

M: They are accepting them?

R: “Yes”.

M: Are they stigmatizing them?

R: “No, you are living witness, I showed you one of the survivor among his friends, mingling together”.

M: Is this the same in the other communities?

R: “Yes, because we are not getting complains that they are stigmatizing the Ebola survivors”.

M: So it is not so again?

R: “Yes”.
