# Supplementary material for: Development of a set of community-informed Ebola messages for Sierra Leone
Source: PLoS Negl Trop Dis. 2017 Aug 7;11(8):e0005742. doi: 10.1371/journal.pntd.0005742 (PMC5560759; doi:10.1371/journal.pntd.0005742)
Supplement: S1 Appendix — (ZIP) [file pntd.0005742.s001.zip › Ebola messages - FGD and interview transcripts/R2HC Ebola Fieldwork 1/R2HC Ebola F1 COM-Urban5 V2 ADD PROBE.docx]

| CODE | **R2HC Ebola F1 COM-Urban5 V2 ADD PROBE (urban semi-structured interview with community leader)**  **V2 – 11^th^ March 2015 – ADD PROBE and correction personal data** |
| --- | --- |
| DATE | February 2015 |
| DURATION (minutes) | 40 |
| Collector nr | 3 |
| LANGUAGE INTERVIEW | Krio |

**PERSONAL DATA RESPONDENT**

| Age *(in whole years)* | 40 |
| --- | --- |
| Sex (F = Female, M= Male) | Male |
| Religion | Christian |
| How much time does it take you to walk from your house to the nearest PHU? (minutes) | 10 |
| Mother tongue: | Temne |
| Education level: | Secondary |
| Role in community: | Pastor |
| Do you know anybody who had Ebola? | Yes |
| If Yes, what is your relation to that person? | Neighbour |

**TRANSCRIPT: (M= Moderator, R= Respondent)**

M: When did you first hear about Ebola?

R: “I thank you very much coming to my community, I first heard the rumour of Ebola in April, when it’s spreads in the west region of Liberia, and in Sierra Leone I heard it in May 2014”.

M: How was it described to you?

R: “First they told us that it is a sickness we catch from wild animals like bats, monkey and chimpanzee. They said Ebola is spread through these animals, and they said if Ebola catch you through those animals that I have measured, you will experience symptoms like body rash, high body temperature, vomiting, and tiredness in your body system, so it was described to us”.

M: What were your first thoughts about it?

R: “Well my thoughts about it, they told us about animals, and these symptoms to me as being in Sierra Leone before, so to say this is the way through Ebola is transmitted, I strongly believed, my only doubt was when they measure the animals as way through which we get Ebola, because people were killing and eating these animals before and they did not get of Ebola, I am really in doubt”.

M: In which ways Ebola has affected your community?

R: “Ebola has affected our community greatly, one, this sickness has stopped our children from going to school, it has made the turnout of worshipers in church has dropped, there is problem in the business sector, and people don’t do business as a result of this Ebola outbreak, most of the people in my community are business people and they are not going on with their normal businesses again, nobody is moving again on business trip from one region to another, it has really affected us in terms schools, the business and the religious aspect”.

M: Have you personally seen or know people who have Ebola?

R: “Yes, I have seen someone with Ebola and that person even died. The person died they diagnosed the person, he was Ebola positive”.

M: Why do think Ebola has spread throughout Sierra Leone?

R: “The one key factor was, we did not understand the message properly, two, there was a total doubt of this sickness, the denials, people were denying, they were not going by the instructions given by the medical personnel, that gave the cause for the spread of Ebola, they said don’t touch sick person, don’t washed dead bodies, people did not follow all those instructions, that is the main reason for the spread of Ebola”.

M: What do you think is the best way to prevent Ebola from spreading?

R: “Well Sierra Leone as a nation lacked so many facilities”.

M: You said we lacked so many facilities?

R: “Yes the country has so many facilities that are lacking, one, people are not strong enough to live a private life, the community is so tight up, the environment we find our self very choked up, the houses are overcrowded, the environment is not conducive, at times, ten people to one bed room, all these lead to the spread of Ebola”.

M: So how do we prevent Ebola from spreading?

R: “One way is by doing massive sensitization to the people, so that they will believe that Ebola is real and it exist, go by the advice of the medical expert like the ones they said, don’t touch someone, wash your hands always, don’t washed dead bodies, If we do so, Ebola will not spread. But people are always choked up in public transports and in market places, but if you are in a public transport wearing you long sleeve Ebola will not spread”.

M: What do you think is the best way to treat someone with Ebola?

R: “Well, we have to listen to the medical experts because we don’t have the know-how and we as religious leaders should advice our people well, but for me the best way to treat Ebola is going to the treatment centre when you have Ebola, I believe those that are more trained know how to treat a person with Ebola”

M: As a community how do you treat somebody with Ebola?

R: “In our community when a person is sick of Ebola, we will call 117 to come and collect the person and take the person to the treatment centre”.

M: You don’t have any other way?

R: “uhmmm, we don’t have any other, because they have advise us not to touch sick person, so the best advice I will give is when someone is sick, call the medical experts to come and collect the person and move the person to hospital”

M: Are there any local terms you described Ebola in your language?

R: “I am a Temne by tribe, for me Ebola is similar to what we call “aawouuka”.

M: What does that mean?

R: “This is a disease that attacks fowls in our community, once they have being attacked by this disease, they will feel tired, weak, they will not able to walk, unless we give them “red and yellow capsule” (= tetracycline) to regain their health, similarly, the symptoms of a person with Ebola will be tiredness, weakness, not able to do anything unless we call the medical people to come and take care of the person, that is what we call “aawouuka”.

M: You said you give fowls the tetracycline to regain their health, why not human being?

R: “Well, fine, why not human being because this sick is transferable and can kill someone faster, we have seen the symptoms, as I told you last when it came, people do you have the knowledge to deal with it, that is why it spreads faster, because they were not taking the preventive measures, they said don’t touch, they will touch, most people were doing this, because of the love they had for their family, so they were even touching the sick, but know we have knowledge that when someone is sick, we should not touch the person, so that is the reason we don’t use this treatment on human being that are infected with Ebola”.

M: Some people do not believe Ebola exists. Do you know people in your community who think this?

R: “Yes, there are people who do not believe Ebola Exist”.

M: Which category of people has this sort of belief?

R: “The cultural people, people that believe in secret societies, so when someone died they have to observe the norms of burial of their secret societies, these are the people that do not believe Ebola exist. They proved it, a member died, they called the other secret society members, and they came, touched and passed all their secret societal ceremonies, which gave us the first confirmed case of Ebola in our community”.

M: Why do you think they are doing this?

R: “They do not believe Ebola exist, and they believe in their traditional or secret societal rights, they will rubbed their medicines on their colleague societal member and do the other procedures, they are doing this because they do not believe Ebola exists”.

M: Please can give me some examples of the Ebola that you have heard, seen, or read?

R: “Yes, one of the key messages are, Ebola is real, don’t touch sick person, Wash your hands clean with soap and water, don’t touch dead person, these are messages we got, most of the time on radio”.

M: What do you think about those messages?

R: “They are good messages. If you do not listen and put the messages into practiced, you will be infected with the Ebola virus disease, because these messages are telling us exactly preventive measures of Ebola and we have proven it and seen it. If you don’t apply to what’s the message says, automatically you are a victim of Ebola”.

M: Are these messages clear to you?

R: “Yes, they are clear”

M: Do you understand and accept these messages?

R: “We understand these messages better, because they are saying it in all local languages of people”.

M: All these Ebola messages you have highlighted, what you think has been the best message you have come across?

R: “The best messages to me are, Wash your hands regularly, avoid touching sick persons, don’t touch dead bodies, to me, I love and accept them?

M: Why do you think you love them?

R: “To me, washing your hands regularly is part of cleanliness, then when someone is sick, they said don’t touch, no person will touch because they do not want to get sick, I like the message because it is clear, its advise and protect your own life”.

M: Are there any Ebola messages that you think do not worked well?

R: “Yes, the one that have not worked well, initially the medical people came and shared a bucket for washing hands, but now it is not happening, the washing of hands is not effective, so it has not worked well, just about a month all washing of hands process stop. Now it is not effective but with area of don’t touch the sick, don’t wash dead bodies it has worked and it is working”.

M: What do you think would be a good message to encourage people to bring patients to a treatment centre, holding centre or community care centre?

R: “Well the only way, now people are afraid to load sick people motor bikes, private vehicle, so other Non-governmental organization, should provide special vehicle to be taking sick people to hospitals, so that will encouraged the sick person to go to the hospital, let them provide a message that, don’t worry vehicle will be available to collect sick person, which is not the ambulance”.

M: In the event of Ebola infection, do you think that people would prefer to go first to the traditional healer, to existing health facilities/staff, or the newly established Ebola health facilities?

R: “ I understand the question, plenty people as I told you, strongly do not believe that Ebola exist because they are stubborn so those set of people so up till this time if they find out that somebody is living with the disease, these set people will hide with the patient and take them to a different place . This is because they have their different believes so they still do not believe that Ebola exist

But yet still, taking the patients to the Health Centres is the best way, though some of them still take them to the traditional healer”.

M: Some people stay at home when they think they have Ebola. Why do you think this is?

R: “Yes. People were afraid because of the rumours spreading out that when you go to the treatment centre, you will lose your life, you understand? So all those misinformation were scary therefore, before they go to treatment centres and die, they will prefer to stay home for treatment. But now that the information has spread that when you go to the treatment centre you will survive, I think people are now taking their relatives to the treatment centres”.

M: What do you think could be done to encourage them to come to treatment centre?

R: “Let them provide special transportation and also let there be continuous display on television the survivors and let the survivors shared their experience and build confidence in other people and they will have that confidence that when they are sick of Ebola if they go to the hospital they will get cured. If they go to the hospital, to me they will get well and it is the best way”.

M: What do you think is the best channel to get new messages to the people?

R: “The best radio for that is SLBC (=Sierra Leone Broadcasting Cooperation), it is the most popular radio in the city, but people like listening to their local community radio, like in this community people like listening to citizen radio, every morning people listen to citizen radio, so if they are to use radio, citizen radio is the best” (*Someone shouting at the background*)

M: Have you ever heard people talking, in good or a bad way about the Ebola ambulance service?

R: “Yes people are talking a bad way about them, they said as they loaded you in that ambulance, they will spray you with chlorine, so people are afraid, as I told you, they are talking bad about them, and people are more talking about the chlorine that once you are loaded, they will spray the chlorine, before they reach you to the destination they are taking you, you are died, people are talking more about the chlorine. But plenty of the people have heard the news on the radio that the ambulance is there for your safety, to take you quickly to the treatment centre”.

M: What about the good talk?

R: “Well the good talk, they are fast, they take you quickly to the treatment centre”.

M: What about the holding and treatment centre, have you heard any good or bad people talking about them

R: “Yes, when the Ebola started they were afraid of treatment centre, they said when you to go to the treatment centre you will lose your life, at that time, the message were not disseminated properly, that is the bad talk, but now a lot of sensitization has been done by government, other non-governmental organization, people are not afraid again, they will go to the treatment centre by themselves, for them to be treated and the good part of the treatment centre people are talking now, they said when you go to the treatment centre, you will be cured”.

M: Have you heard people talking in any good or bad way about the Ebola burial team?

R: “The bad about the burial team happened during first few month, April, May and June, this was the time the Ebola burial team were undertaking bad, they were not treating the dead bodies of people properly, they were just throwing the dead body in the vehicle when they went and collect but after the spread of this message, they changed from bad to a decent way of treating the dead body. When they come and take the corpse, they will prepare the body fine, treat the body well and even this later part they will allow the family members to offer prayers for the dead corpse before the dead body is being put in the ambulance and take the body for burials, there will some family members to accompany them to bury, before people were talking in negative way about them”.

M: What about the Ebola burial team are people not talking in bad way?

R: “The Ebola burial team was not having good record before, people were not talking good about them, a quiet number of people were not believe about the existence of the sickness but the team they know about the sick and dangers of the sick, and they were trying to protect themselves so they were not treating the dead body in a way that pleases the family members, that was the misunderstanding between the family and the team, in recent days the present burial team, people are talking about them good in (- - interview district - -), I don’t know for the other part of country, they will come now, take the body and asked the people to pray for dead person in the their different religion they belong, after the prayers put the body in the vehicle and take it to cemetery for burials”.

M: You said first they were not doing well, does that gave the people the opportunity to do secret burials?

R: “Yes of course, well, when they come and collect the dead body throw it in the vehicle in front of the a family, they will say my brother or sister was not treated with respect, next time when any member of my family died, I will prefer to washed, dressed and burial the person secretly, that caused the spread of this disease, that was before”.

M: What about now, are people doing secret burial?

R: “As for now, they are not doing secret burial in this community; in fact we don’t even have a graveyard”.

M: What about the 117 phone line, is there any good or bad people talk?

R: “Well I have not heard any bad news, because it is the number you called, for them to send ambulance to come and collect either the a sick person or a dead body, but before now, people were disturbing the 117 phone line, calling them unnecessary, they will call, when an ambulance come, they will not see the person, 117 started listening to key people in the community now, when there is case, only that person will call them for them to respond, but on a whole to me, 117 doesn’t have any problem”.

M: Any aspects of the existing health facilities/staff that is now working on Ebola care and treatment centre?

R: “Well to me people are talking good about them and they are really trying, there is no bad talked I have heard of”

M: How do people react to Ebola survivors in this community?

R: “ Well now, they are encouraging them, because the messages, is when someone had survive from Ebola we should see that person as part of the community and let take the person as our brother and sisters and it is now happening”

M: What about before?

R: “People were afraid of them, they were afraid, don’t even want to see them, even to mingle with them was a problem, every as afraid but because of this message, people are now encouraging them”.

M: Are they stigmatizing them?

R: “No, they are not because everyone is aware due to the messages”.

M: Have you heard of any new treatments for Ebola that may become available soon?

R: “Yes, we have heard of a vaccine for Ebola, but I have not yet seen it with my eyes, they said they have discovered a vaccines for Ebola but we in this community have to see it, I don’t know if it is in the country now”.

M: What do you think about it?

R: “Well I am happy and I will appreciate if they come with it, because Ebola has really disturbed our whole life and we are all looking for cure”.

M: What kinds of concern do you think your people will have towards the vaccines?

R: “They will not have any bad concerns, they will be happy and welcome the vaccines with open hands because they are dying and they want to stop this Ebola”.

M: Have you heard of any new ways to prevent Ebola?

R: “I have not heard of any new ways

M: I mean in your community?

R: “No, only the old ways, don’t touch, avoid body contact, wash your hands regular, call 117 when some died, just the same thing/ we don’t have any new ones, only they are telling us to encouraged survivors”.

M: What are the most common questions about Ebola that you are faced (with) as a pastor of this community?

R: “They always ask, man of God do you believe that this sick exist? As a religious leader, I always say yes, because God even measure about this disease in the bible , from the time we the human being sinned against God, and God sent down disease has punishment for man ,for instance the story of Job, God punished him by making him sick. So there is no doubt about sickness because it was measure in the bible and it is real and you people have to believe”.

M: As a pastor, what do you feel you need to know to enable you to respond more effectively to question asked by your people?

R: “We need your own messages although we have pastors who medically oriented, who has done some medical aspects, but if you can ask some other pastor about a medical issues, they will not answer, unless they quote for you from the word of God, what we need to know, we believe that the sick is real, and that sickness is measure in the word of God, when the medical give us the information we will also pass it on to the people”.

M: What kinds of information are you expecting from the medical people?

R: “The information related to this sickness, the medical people have tried by telling us how to prevent this sick, that is what we want them to empowered us more, we have told the people to washed dead bodies is not good and they listened to us as man of God, I don’t know for the other sets of people, but as long as the medical people have told us not to touch sick person, washed dead bodies don’t do it and they listened to us, but we need more information from you, the more accurate information you give us so we will pass it on”.

M: What do you really want to know, so that you will respond well to questions asked by your people?

R: “Well, I want to know if we get Ebola through by mosquito bite, and again I want to know, how this frequent washing of hands will prevent you from getting Ebola”.

M: Ok, is there anything specific about Ebola that you think people need to understand better?

R: “We need to know, when are they coming with the vaccines, or whether it has come into existence or not, but if we don’t know we can’t give the people the proper information about the vaccines, so we want to know, so our people should know, but we don’t have any information about it”.

M: Is that all you want to know about Ebola?

*(The respondent and the moderator laugh)*

R: “The other thing I want to know, when this Ebola is going to end, this is a question always asked by people, because plenty of them do not believe that it is a sick from God, they believe is a manmade disease, but we believe that all sickness is from God, God always punished people in different ways, so they have to believe, because God spoke about sickness and War. So they people asked when this sickness is going to end, we always answer, by the grace of God through prayers and with the backup from the medical people it will come to an end”.

M: Thank you, do you have any other thing to say in line with this interview?

R: “Well I thank you for your time taken to interview me, it is a big interest and concern, it is my pleasure to thank you and your organisation for taking this venture”.

**ADDITIONAL PART OF INTERVIEW, OBTAINED BY COLLECTOR 2 AFTER CONSENT IN PERSON, March 2015:**

M: You said the two factors that were disturbing this Ebola issue, one people were not understanding the message properly, and two they doubt the sickness and were denying, so why were they denying the sickness?

R: “This sickness is above the understanding of the people and the entire country, and the sickness had more knowledge than the people”.

M: What knowledge does the sickness have?

R: “The sickness, as I said is above because people do not understand how to deal with it, because if they told you not to touch and also when someone had died, don’t wash or touch the person, but in our tradition when someone died, we wash and dress the dead body perfectly, and the sickness is above that, and this led to the spread of the sickness”

M: Why the people were not adhering to the instructions of the medical people?

R: “The people do not believe the medical practitioners”.

M: Why?

R: “As I said the people did not understand the sick and they doubted it, and there was a rumour that when you go to the medical people, you may get implicated, and people were having this fear and did not take the medical advices”.

M: Why the people do not believe the message, it is because the people do not give out the message well, or it is just there on thoughts or the channel of passing the message had a problem?

R: To me, it is the beliefs of the people”.

M: That was why the messages were not going down well?

R: “Yes, because the message do not have any problem”.

M: “The channel also was not having problems?

R: At all, not”.

M: So it was because of their own beliefs the message were not going on well?

R: “Yes”.

M: Ok, I knew later, they accept that this Ebola sickness exist, why the change of belief?

R: “They had saw many examples, that when you touched you will get the sickness and the sickness has killed a lot of people, plenty people died”.

M: Which month or date they got this belief?

R: “To me, it was September last year, this was the time they really believe and the belief was serious”

M: In your community, which time do you first hear about Ebola?

R: “We got our first Ebola case in August and then in October it got more serious and killed a lot of people”.

M: What language is awoouka?

R: “Awoouka is a Temne word”.

M: What is the meaning of awoouka?

R: “Awwouka is the sickness that disturbs our creatures or fowl in the villages, there is a special season for the sickness, it makes the fowl appear doll (=dull) and weak, in most cases unless we use capsule to cure them”.

M: Is similar to Ebola?

R:”Yes, they are similar with Ebola, when someone is infected with Ebola, the person doll (=dull) and weak and able to move, that why we call it awwouka”.

M: Ok, you said the washing of hands was not effective, why was it not effective?

R: “The washing of hands”?

M: Yes, why was it not important and why were people not doing it?

R: “Because they do not believe the sickness, as a result of that, they said if even you washed your hands, does not solve the problem”.

M: So it was just the belief of the people, that even if they wash their hands it will not go?

R: “Yes”.

M: The Ebola survivors, which problems are they experiencing now in the community?

R: “Well the survivors, people are actually ignoring them, some people are saying when someone survive from Ebola, there is a certain periods of time, you don’t go nearer to them, more male survivors you should have sexual intercourse with your wife, but now, even now if they found that you are Ebola survivor they will not go closer to you, because they are not sure. Some people may think the survivors had not gone through the time given to them”.

M: They have not fully accepted them?

R: “Well first they were not accepting them but now they had accepted them due to the messaging that is going on”.

M: So they have accepted them now?

R: “Yes”

M: What about the other communities are they doing the same or something different?

R: “Actually we had accepted in our community and that is happening all over the country and they had seen the trouble been caused by the male Ebola survivor, giving problem to wives”.

M: Giving problems to their wives, how?

R: “Fine, when they tell the Ebola survivors that after surviving from Ebola, you had ninety days before you get a sexual intercourse with your wife, but they kicked against that”.

M: So they are not abiding by that?

R: “Yes, they are not abiding by that, as a result the wife will die”.

M: Have you heard cases like that?

R: “Yes, we had heard that in the provinces, although it has not happen in our community,”

M: That Ebola survivors had done this?

R: “Yes”.

M: Hmmmmm.
