# Supplementary material for: Development of a set of community-informed Ebola messages for Sierra Leone
Source: PLoS Negl Trop Dis. 2017 Aug 7;11(8):e0005742. doi: 10.1371/journal.pntd.0005742 (PMC5560759; doi:10.1371/journal.pntd.0005742)
Supplement: S1 Appendix — (ZIP) [file pntd.0005742.s001.zip › Ebola messages - FGD and interview transcripts/R2HC Ebola Fieldwork 1/R2HC Ebola F1 FGD-FEOLD-Rural1 V3 CORR.docx]

| CODE | **R2HC Ebola F1 FGD-FEOLD-Rural1 V3 CORR (Rural focus group discussion)**  **V2 18feb2015 – Originally posted as rural2**  **V3 – 11^th^ March 2015 – Corrected personal data participants** |
| --- | --- |
| DATE | January 2015 |
| DURATION (minutes) | 79 |
| Collector nr | 4 |
| LANGUAGE INTERVIEW | Krio |
| TYPE FGD | Female Old |

PERSONAL DATA PARTICIPANTS

| Nr | Sex  (F/ M) | Age  (in years) | Education Level (e.g. none, Primary, secondary, tertiary) | Language (e.g. Mende, Temne, Krio) | Religion | Job / Employment (how they earn their living e.g. farmer, teacher, trader) | Role in community  (e.g. youth leader)  ANONYMIZED, ONLY AREA OF ROLE INDICATED |
| --- | --- | --- | --- | --- | --- | --- | --- |
| 1 | F | 56 | Tertiary | Temne/Krio | Christian | Teacher | None |
| 2 | F | 35 | Tertiary | Temne/Krio | Christian | Teacher | None |
| 3 | F | 60 | None | Temne/Krio | Muslim | Farmer | None |
| 4 | F | 47 | Secondary | Temne/Krio | Muslim | Trader | None |
| 5 | F | 45 | Tertiary | Temne/Krio | Muslim | Teacher | None |
| 6 | F | 35 | Primary | Temne/Krio | Muslim | Farmer | None |
| 7 | F | 35 | Primary | Temne/Krio | Christian | Business | None |
| 8 | F | 60 | None | Temne/Krio | Muslim | Farmer | None |

**TRANSCRIPT: (M = Moderator, R= respondent, R1= first person responding to a question, DOES NOT correspond to numbering used in Personal Data!)**

M: The first question I am going to ask you is; how has Ebola affected your community?

R1: “The way how Ebola has affected our community?

M: Yes Ma.

R1: “We were the first victims for Kailahun (first district in Sierra Leone with Ebola cases) to ( - - Name of interview village - -). This is a survivor elderly woman. Out of 31 Family group, 16 are gone

R: “17”.

R1: “17 away. So we have the bitterness of the Ebola in ( - - Name of interview village - - )”.

M: Yes Ma can you too add something to that?

R3: “Out of the 17 who died, what actually us was the too much spray. They used to spray too much Chlorine on people and after they finished they come again with the motor car and spray, without anybody dying- just spray, spray’.

M: What I mean, when this Ebola breakout in this community, how has it affected you really?

R4: “One area where this Ebola has affected us is that right now even the school children, most of them are pregnant. Then the book learning, many girl children have turn to zero. Like me sitting here I have one daughter, now she challenges me because she has involved into sexual intercourse. The way Ebola entered (- - Name of interview village - - ) town from Kailahun (first district with Ebola cases in Sierra Leone) to here, any way that pain is disturbing”.

M: Ok Ma, can you add little to that?

R5: “When Ebola breakout, even my own biological child was infected, he was cured in ( - - Name of district headquarter town of the interview district - -). The very day when he arrived, the very day we took him to the Centre. When his blood was tested, they said he has the sick so they took him to Kailahun (first district with Ebola cases in Sierra Leone) where he was cured and brought him back in good health; I bless God for that, now that the white man has intervened in this Ebola business”.

M: Yes the Mami, can you too add yours?

R6: “The thing which affected us more in (- - Name of interview village - -) here about this Ebola. People have medicines which they put into the water well. All this can make us fear. And number two, so when they carry our people they do not come again.

M: Ok, can the Mami too add something to that? How has Ebola affected you?

R7: “Well we say thanks to God now. Because at first we were “worried at heart”. When we lie down, we do not sleep. The next thing people will tell you to watch out for those people who come to spray. We do not sleep. The water wells where we fetch water to cook, drink, launder and do everything, were all sprayed. We too we never felt free”.

M: Yes Ma, how has Ebola affected your community?

R8: “This one is disturbing, because it is now in our own hands. All of my children have died now, I don’t have anything”.

M: Yes the Mami, you be the last to talk on this, “ya”?

R1: “Ok this Ebola has taken us back. The way it attacked us here in (- - Name of interview village - -), here we did not feel fine”.

M: How did it take you back?

R1: “Well, people with whom we were doing business, now business is not going through. If you are ready to go anywhere, there is no chance to talk about. At night you are worried, when day breaks you are worried. All those headaches have spoilt our chance”.

M: You said there is no chance to talk about, what do you mean by that?

R1: “Well when they said you have to stay in one place. Because when you leave here and go to Freetown (capital city), they will not accept you. They will tell you to stay in one place. That one is disturbing. People like us we keep moving up and down to find what we can eat. But this one has made us to sit in one place. When it finishes, we are going to be very happy”.

M: Yes Ma, have you ever seen or known anybody who has Ebola?

R2: “Here in (- - Name of interview village - -)”? Well we here when they come they just take them and carry them away.

M: Do you know the people?

R2: “Yes this now is one of them. When they took her away she came back, she is a survivor. So as you see her now she is my neighbour”.

M: Yes my sister, do you know of anybody who has Ebola?

R3: “Well, here when they came to take them away including my sister who died and her brother called (- - name of brother - -). When they took him they said he was positive. From the time they took him away, he didn’t come back; they said he is dead”.

M: Yes the Mami behind there, can you add anything to what your sisters have just said?

R4: “Except, for the child of this woman who came back alive but all the balance of (=rest of) the people nobody came back, all of them died there. We are all afraid, we are not feeling free on that”.

M: Yes, what about the Mami who is in the middle there, you too, talk small thing?

R5: “Well, we are all afraid”.

M: What I mean, have you seen a person with Ebola?

R6: “I have not seen a person affected by Ebola”.

M: But just now you told me that this guy is a survivor?

R6: “But I have seen when they come they can take people and carry them away and tell them that are positive or negative”.

M: Do you know those people who have survived Ebola?

R6: “Yes, I know them, but they are not coming again”.

M: Yes the Mami, can you too add something?

R7: “They said the symptom of Ebola is when some vomit”.

M: I am not talking about the symptoms, I said if you have seen or know a person who has Ebola?

R8: “Because of my child who came, he vomit, he went to toilet and his eyes were red. When we went and they looked at him they said he has the sickness”. (Ebola)

M: Ok, so the Mami at the last end there?

R12: “Well all is the same. Our children here in (- - Name of interview village - -), a lot of them who have come back pregnant, they said they have the sickness (Ebola), and we know that they have come because they have the sickness (Ebola), that is why they have come”.

M: Ok, so what I want to know is...

R: “Talk to me why do you want to jump me? You had jumped me once. I have spoken only once. I have seen somebody with Ebola, he vomits and blood was oozing out of his nostrils, he died of Ebola, So if you want to jump me, please do not jump me”.

M: Ok, I am sorry about that. Eh, what I want to know again is, why do you think Ebola has spread in Sierra Leone?

R1: “Ya (yes) It is the movement. It is the movement of the people, because they said it is a sickness that transfers. When you do not steady in one place maybe where I go I take it from there and bring it back to my family and spread it. They say it is a “Family Mondor” (=family share). So that is why the sickness (Ebola) spread”

M: Ok, yes Ma, can you tell me why Ebola got spread in Sierra Leone?

R2: “They said we should avoid body contact, but we do not agree. When a person dies we go ahead to wash it. They have told us to stop all that because the sickness gets spread”.

M: Yes the Mami at the back there?

R3: “They have told us that let everybody stay at home. When you move from your home to another place, maybe where you are going, you will meet the sickness (Ebola) there. When you return to your family, you spread it there”.

M: Yes, the Mami?

R4: Well like when a person,a person dies, if that person is a Muslim, they should not pray over the body. If the person is a Christian, they cannot take the body to the Church”.

M: What I mean, why do you think Ebola is spreading in Sierra Leone?

R5: “Well when they said it has germs, and that can be transferred to a person. That is why eh, people are afraid”.

M: Ok, the Mami, yes why do you think Ebola has spread in Sierra Leone?

R6: “They said you should not touch, body to body. Even if it is your child, when sick, keep off from him or her. Even if he or she dies do not wash it or pray for him”.

M: But what you have said, do people abide by it?

R6: “No they don’t do it”.

M: So De Mami, why do you think Ebola has spread in this country?

R7: “When we meet we should not greet each other, you keep to yourself. If we meet and rub skin to ourselves it can transfer. So instead of that let us keep to ourselves so that Ebola can finish. But if we are not doing that then Ebola will never finish”.

M: Ok, De Mami, you too can add small thing there, ya?

R8: “Ok Mama. When Ebola came they told us not to shake hands, then we should not sit together, even prayer, when we go for prayer at the Mosque, in the past we used to fill the Mosque and we touch each other, but now, sometimes we can be twenty (20) in the line, a big line because of Ebola. “They said don’t touch”. So if you do that, we will be affected by Ebola”. It is something that transfers, that is why we are taking the advice that they are giving”.

M: So, which word do you use to call Ebola in your language, you said you are Temne and the others also speak other languages?

R1: “We just say Ebola, because they said it is Ebola. So it is that Ebola, because they said it originated from a stream, a stream is named Ebola”.

M: Yes De Mami, you have not said anything yet?

R2: “Myself it is so. They said we should not touch anybody”

M: What I mean, how do you call it in your language?

R3: “We say the sickness is a rascal sickness. That is how we call it, we call it “RASHKAY”.

M: What do you mean by “RASHKAY”?

R3: “RASHKAY”?

M: Mhmm.

R3: “You should not greet your good person again; you should not shake hands with him again, even if you have taken centuries, because in the past after a long time not seeing this woman, I am going to wrap her and kiss kiss her. But now that is not possible again. When I see her now I take my time, as I am scared of her, so is she too scared of me. So we take that to be rascality, that is how we take it”.

M: Thank you very much ya. Yes Ma, we were about to say something?

R4: “Ee, it is the same thing I wanted to say. Because the other name, especially when your good person come and there is no way to do that is what I was about to say. That is why they call it that name”.

M: Which name?

R4: “Ee, that RASCALITY”.

M: Why do you call it RASCALITY?

R5: “Well for us in Temne, we say RASHKAY. RASKAY in the sense, you have been on terms with a person then when see him or her you do not appreciate him or her, you take different eye to look at him or her as if he or she is a stranger. Even myself it has happened to me. I went to the bank and I met ( - - name of a female - -), it has taken over a year without seeing her. She greeted me and said “Aaay sister,Aw u do” (= Sister, how do you do)?. I just looked at her and replied “Ekushe” (= you are welcome). So she felt it and I felt it too. Because I have been use to wrap wrap (= hug) with her, kiss, kiss her and she will tell me about her work. But no sooner we greeted in that silent way I did not feel fine in my heart. This is a sickness...is only God can, it can make a family (*interruption)* Mhmm! That brings changes, then you begin to recall”.

M :Ok, There are people who do not believe that Ebola is real. Do you know such people who do not believe that Ebola is real?

R1: “Well at first, many people did not believe that Ebola is real”.

M: Why do they think that way?

R1: “Now we believe that Ebola is real”.

M: Why do they think that way? Yes De Mami?

R1: “Well some of them who feel that Ebola is not real say the symptoms of Ebola and the other sicknesses, they said the sicknesses have been happening and they were cured. Gonorrhoea, this,that. But now all those sicknesses have been related to Ebola. So all of us have agreed, we who believe. Some say these sicknesses have been happening, and they are cured. Now they say is Ebola, a sickness without medicine. So, are still arguing, well it is this denial that has caused this sickness to spread”.

M: Yes De Mami, can you too add small thing?

R2: “When this sickness breakout, many were denying that when they said people should not eat beef, like a leftover of fruit eaten by bats like mango. If it happens that you have played anything you have to wash your hands. All that our people said in the past they were not washing their hands yet they have long life. But now even when they are saying that people should not eat mango leftover by bats, people say it is a lie, they deny about that. But the way they have seen the sickness is hitting us because of denial, people are beginning to be(come) aware”.

M: Yes De Mami?

R3: “Some were denying that Ebola is not real. But now they believe”.

M: Why do they deny?

R3: “Ee, because they were not seeing the symptoms that is why. Just like what that woman said, all those symptoms have been there, but they were still being cured. So if they are saying they are going to put together with Ebola, which is why some are denying. But the way we are seeing Ebola, the way it is killing those who do not take control, everybody now believes that Ebola is real”.

M: Yes Mami at the back there, you too can add small thing there ya?

R4: “Well some are denying that Ebola is not real. But me, my children, when they go to toilet I wash their hands, protect them for them not to get Ebola”.

M: Yes De Mami who is closer to her?

R5: “This sickness they again said is caused by water, that it is a germ that moves with it and breeze”.

M: Why do they deny that it is not real?

R5: “No, we are not denying. Because we now know that the sickness is real. But after showing you to wash hands those are what we are doing small, small”.

M: Yes De Mami, you too can add small thing there ya? Why do they deny that Ebola is not real??

R6: “Why do they deny, at first they said there is no medicine. Can there be a sickness without medicine? That is why many people do not believe”.

M: Yes De Mami?

R: (*giggles)*

R6: “Ee, a sickness without medicine? Any sickness should have a medicine. But if they say there is a sickness without medicine, how are you going to believe that it is true”? That is why a lot of people were denying at first”.

M: Ok, so what I want to know again is, those Ebola messages Mhmm? That you see and hear, I want you to give small, small example about them? Yes De Mami, give me some?

R7: “They said let us abstain(=have no sexual intercourse). Some of them, after being cured, they give them a law, like the men they should not have contact with the man until after three months. Some of them when they come out, they wouldn’t bear and that is how the Ebola transfer’.

M: Yes De Mami, can you add small thing there? The Ebola messages, give us some

R8: “The Ebola message, the said we should not wash dead body. That is a big message because it is a tradition, so that message has entered into us now, so by now we should not wash dead body”.

M: Yes De Mami, tell us the Ebola message that you have heard ya?

R1: “The Ebola message is you should not touch another person, person should stay where they are”.

M: Yes De Mami, do you have anything to add to that?

R2: “The Ebola message, if someone vomits nearer to you, you should not touch that vomit, if you have a plastic you put it on before you carry him or her to hospital”.

M: Yes De Mami, can you add small thing there? The Ebola message you heard?

R3: “All the Ebola messages I have heard, they said we should touch nobody, when somebody dies we should not touch it”.

M: What about De Mami at at your back?

R4: “The Ebola message say you should stay where you are and not to move one place to place”.

M: Yes De Mami, you too?

R5: “They have said when a person dies, even if it is your child, don’t touch it you have to cal 117”.

M: Yes De Mami right there in the corner, she is thinking about what to tell me, tell me at once?

R6: “Well Ebola they told us that we should clean ourselves every day, we should wash with warm water. They said it doesn’t like dirt because the “Tumbu” (=Germ) if you fail to wash it is going to enter into your body, well also need to wash with warm water”. The virus does not like hot”.

M: Yes my sister, what do you think about it?

R6: “It is fine”.

M: You have to talk louder ya?

R6: “It is fine; it is to prevent ourselves not to get Ebola”.

M: Yes De Mami, you were telling me about the Ebola messages?

R7: “The messages have trained us now. This “Wash Hand” has now become a habit, even the smallest “pikin dem” (= children) at home, he or she cannot just come from the play ground and you give him or her food and she begins to eat he or she will remember to say oh, I need to wash my hands”.

M: Yes Mami, what do you think about these messages?

R8: “They said people should not go walking about, that everybody has to stay in one place”.

M: So what do you think about that?

R8: “They said they should not wash any dead body (i*nterruption*)..

M: Let us leave her to say what she wants to say ( ………??.............) Yes De Mami?

R1: “Well as for us the message which they sent to us when we followed it we saw it, that is why we are a bit “Blo-blo” (=at peace) now

M: Ok, Yes?

R2: “Well the message is fine because it give us good health and long life”.

M: Yes the Woman at the back there?

R3: “Well, the message we are happy for that, thank God when the medicines have come”.

M: We are talking about the messages first.

R3: “The message is fine

M: Yes De Mami, you too?

R4: “The message is fine. Right now we are feeling fine and we thank God”.

M: Yes Mama?

R5: “Ee, is the same thing”.

M: Which one is the same?

R5: “In the past they told us that there is no medicine, but now medicine has come. When you are sick you can go to the Centre they can treat you. On that side we are feeling free”.

M: So, after giving me what you hear in the messages, what do you think about these messages? The way they disseminate the messages, what do you think about all the messages?

R6: “Well these message are safety, they safe guard us now because we have take it to be a habit, a good habit”.

M: Yes De Mami you too, how do you see the way the messages are coming to you?

R7: “The way the messages come to us, the way we see it is fine. It give us good health”.

M: Is it clear properly to you?

Rs: “Yes, it is clear to me, no cause to complain”.

M: Yes Ma, how do you see the messages?

R8: “It is the same thing, they did not come to tell us what will break our heart, but they come to tell us what will benefit us so that is why when they tell us and we follow it we feel fine”.

M: Ok, yes De Mami over there when they tell you the messages, what do you think of them?

R1: “We feel fine”.

M: So the Mami in the middle there?

R2: “Like we are saying now, everybody is well, we are feeling fine”.

M: Yes De Mami, you too?

R3: “Is the same thing like they have said”.

M: No, is your own I want to hear?

R3: *(“Laughs”)*

M: How do you really feel about the way the messages are coming to you? Is it clear to you? Do you accept it?

Rs: “Yes”.

R: “It is clear, we feel fine”.

M: Ok, what is the good message that you can give to people to encourage to send sick people to the hospital, particularly to the Treatment Centre for Ebola? What do you tell them to encourage them to tell the patient to go to the hospital? Yes De Mami we start with you

R4: “Like in our own Community here we have those Contact Tracers they move from house to house encouraging people telling them when your person is sick, do not keep him at home, and send him or her to the hospital. They tell them that there are Treatment Centre, people are there to cure, not that when you go there you are going to die. So we have been seen that. They have been bring sick people out and take them to the Centre and they get well and come back. So they encourage people to bring the sick out and not to keep them”.

M: Yes De Mami, what can we tell people to encourage them to take sick people to the Treatment Centres?

R5: “Is just giving them good advice that let them not be discouraged. Some, like yesterday that case happened. A second daughter of a woman got sick, mother for her daughter, but she had hidden her, where they went for treatment, there was no improvement. So when we told her to take the child to hospital she agreed and they treated her, the child had even stopped breast feeding, there was no milk in the breast”.

M: Ok, yes Mami, which kind of message we can give to people to encourage them to carry their people to hospital?

R6 “We thank God for like this (- - Name of interview village - - ) village we selected Committee to go house to house to search for sick people when found we bring them out talk to them”.

M: Which kind of Talk?

R6: “Tell them that there are medicines in the hospital now, if you go there you can get well and come. Do not lie down at home, if you lie down here and take “Pepe Doctor” (= Drug peddler) to treat you, you have to go to the hospital there medicines there let them treat for you to get good health”.

M: Yes De Mami closer to her?

R7: “Well all this is fine. All the good talks they have been giving are making us happy because in the past when they carry somebody he or she will not come back, but now when there are treatments, the coming of the white man if they carry you to hospital they can cure you and come back. So we know that the message is fine”.

M: So, De Mami, yes what can we tell people that would let them not be afraid to carry people to hospital?

R:8: “Well we will encourage them not to hide the sick. When a person is sick you have to carry him or her to hospital”,

M: Why should you not hide it?

R8: “Because some of them are afraid to carry him or her to hospital because they say if they carry him or her, he or she will not come back. But me when they carried my child they brought her back, you see”.

M: What about you?

R1: “Well at first, people were afraid, when a person is sick they won’t take that person out; they will hide the person, you will not see them again. But now that is not happening again. Now they have taught us, they have enlightened us. We tell thanks to God for this chance. So it is fine, the message is fine”.

M: Ok, so the Mami over there, you say small thing about what we should tell people to make them glad to carry their people to hospital or to the Treatment Centre?

R2: “What will make them happy to take their people to the Centre”?

M: What are we to tell them?

R2: “We only need to tell them that when you go there you can get a medicine then you will get your good health. If you stay at home and you do not have medicine you will not get well but when they take you to hospital they will give you medicine and you will get well and come back and join your family they can be glad for that”.

M: Mhmm De Mami you want to say something?

R3: “The other thing again, maybe it is not Ebola. Is just that they are afraid. Sometimes it cannot be Ebola, it can be chronic Malaria but when they carry and discover, now that the Doctors have come they can be able to treat a lot of sicknesses in the event; Gonorrhoea, syphilis, everything and any kind of sickness now they will cure you. Then when you are cured and you come back, it is a free treatment you don’t have to spend”.

M: Ok thank you. So I want to know, which channel can be best used to send those new messages about Ebola to reach to the people?

R4: “Use like how”?

M: What can you use to be able to send those new messages about Ebola for it to reach the people?

R4: “Well like for some of us you have got the idea now, we are preachers. So you can visit somebody who have still not got the idea, when you reach there you advice the person that it is not all sicknesses that are Ebola. The treatment is free, you don’t have to pay”.

M: Yes De Mami, do you have any other way that we can tell these new messages apart from when this Mami said to visit or any way we can do it, the channels we can use?

R5: “Well just like as come the way you have come. You come to enlighten us, we in turn go and explain to them what is the thing. That is the only thing makes them happy that if you tell them anything they will be glad to take their people to the hospital”.

M: Yes De Mami, can you think about any way we can these messages to people, the ways we can use?

R6: “Well the only way is like this when you come and transfer it to us. In turn we too have people who have not got the message yet. So if we have the chance to meet such people we can transfer the message to them”.

M: Ok, so is the De Mami, is there any way we can use for the message reach to the people?

R7: “Well just like what our sisters are saying. When a person is sick and you go to visit the person, you can explain to the person about the sickness and how they can treat it. Also that the person too can “take heart” to go to hospital”.

M: Yes De Mami in the middle there, which way can we try to get the message out there to the people?

R8: “Let the government try the more, especially for the children. The children are no longer learning, so if school was open, we can enlighten the children how to go about this sickness”

M: De Mami, can you tell us something

R1: “We also hear it when they say it on radio the things that they should not do”.

M: Why did you say to use radio?

R1: “The radio, because when your child goes to toilet, he should wash his hands. Even you the big one when you go to toilet, you should wash your hands, if you have Chlorine and Dettol when you heat the water you drop it inside”.

M: So you too can you tell me what you think about the visit?

R2: “Well there are some people who have not got the message; they are still in the dark. But, when, you meet such problems, you have to advice the person. And also the training, you train some people who disseminate the message to other people”.

M: Yes De Mami do you have any way we can tell the message to the people for them to understand?

R3: They need to train people to understand how they treat people”.

M: Yes De Mami, you are just thinking?

R4: “They have thought us, they have enlightened us. People are now clean. At first we did not have understanding but right now it is better”.

M: Yes De Mami in that corner?

R5: “Is the same thing all what they are saying. Because when you do not understand, it can be difficult but when you understand you can know how to go about it for you not to have problem”.

M: Ok, I also want to know, do you think if somebody has Ebola that person will. Rather go to the herbalist or to the hospital?

R6: “At first, when there were no hospital the first idea that would come to them, some would think that the vomiting is as a result of “Fangae” (= witch-gunshot). So they take him to a “Moray man” (=sorcerer). Maybe is out of “Sababu” (= influence). Bless God there are hospitals now. Blood, be it “Fangae” (= witch-gunshot) now or not when we go to the hospital the “Fangae” (=witch-gunshot) will finish because they will treat you there but when the Centres were not yet open, the first idea was to go to native medicine”.

M: Ok, what about those Health Centres that were there before?

R6: “Those hospitals, clinics”?

M: Yes, were people attending there? Or do they think of first going there?

R6: “Well, about that, I cannot tell you lie when this breakout many hospitals people do not go there. Even when you go there they look at you differently. Because of death toll there is why people do not go there and decide to go to the herbalists, even if the person did not get well, but it sustains him. When you go to the Government Hospitals and other outside Clinic they tell you there is no medicine. So that is the first idea that makes people go to the herbalists”.

M: So what about those Holding Centres, those Treatment Centre, do people remember to go there first?

R6: “People don’t go there”.

M: Why do you think so?

R6: “When you go there they hold on you and observe you, after which they call, at that time you can get “Alafia” (=relief). If the Holding Centres had not come, it would have be difficult” to have removed those people from various corners, they would not have come”.

M: So De Mami you too, so do you think if somebody has Ebola, the first thing he or she would do is to go to the herbalist?

R7: “That is it. Because they, this Ebola when it affects you, you are going to vomit blood and it oozes out of your nostrils and when the same symptoms is like when one has been “Fandae” (= fire one with witch gun) so they first go to the herbalist”.

M: So you think that they first decide to go the herbalist?

R7: “Yes because they are told that there is no medicine in the hospital. Even if you are sick and go to the hospital they will tell you there is no medicine. And we are also afraid. There some people when you go there the medicine they give you is to kill you once and for all”

M: What about those clinic that were there before this time? Do people think of going there first?

R8: “Well in the past they were giving out medicines. Because of this Ebola, when people are sick they said there are no medicine. So we are not thinking about that”.

M: What about the Holding Centres, like the Treatment Centres for Ebola, do people prefer to go there first?

R1: “Well now we go there because when you go there if they see that it is sickness or not, they can come and take you go and give you medicine and they cure you. We do go there”.

M: So De Mami you too, please add small thing to it, ya? Do you think when a person is sick of Ebola the person will first think to go to the Traditional Healer, herbalist or those medicine men?

R2: “At first, “lest” I forget that part. First they said that “na sweh fordom pan we” (= we have been cursed). ‘It is a curse, can’t you see one household everybody is dead, is a curse’. We stopped going to the hospital, we said it is not Ebola, it is a ‘sweh’ (= curse). But we thank God, when the message is coming. Some of them when they attend to meeting and they open (= put on) the radio, we will call our children to come and sit down to listen. Through the message that the radio brings to us all of us are now aware”.

M: What about those Health Centres that was there before this time, will the people remember to go there first before going to any other place?

R3: “Even before the Ebola outbreak when you go there they won’t look at you, except you go to the herbalist”.

M: So what about those Treatment Centres, those Holding Centres for Ebola, do they go there first?

R3: “Right now they go there. At first when they carry a person there, by the time you think of it, the person is dead that was why they were going to the herbalist. But now when they see that people go there and get well, they do go there. Now anyone who falls sick we call and they come and take the person”.

M: Yes De Mami who is closer there, do people remember to go first to a medicine man?

R4: “At first it used to happen. When this sickness came they said it was a “witch plane crash” that is why they are dying so much, that made us believe that except we go to medicine man. But now, we know that this sickness is real. So there is medicine now”.

M: What about those Clinics that was around, do people remember to go there first?

R4: “They say you have to go there for check up but at times when you go there they say there is no medicine. Then they also fear to treat you”.

M: So what about those Holding Centre, those Treatment Centres for Ebola, do people remember to rush there quickly?

R4: “Now they have seen that there are medicines, so if you see those symptoms you can go there for them to call and try to know if it is the sickness or not, then they can treat the person”.

M: Yes De Mami at the back there with that nice Yenki tie on your head, so you too can add small thing. When people have Ebola do they first go to the herbalist?

R5: “No, at first they used to do it but now they don’t do it. When somebody is sick you carry him straight to the Centre”.

M: Why do you think they are behaving this way so now?

R5: “Well there was no medicine, all the hospital were all closed. When you are sick and you go to the nurse, the nurse will drive you. But right now we thank God there is medicine and the white men are here; if you go there they will treat you. We tell God thanks.”

M: So De Mami, where do you think people will think of going first?

R6: “Well, at first they used to go to those herbalists because they were afraid, when they carry you away, you are going to die. So before they carry you..., but now there are plenty hospitals, now when they carry you, they cure you and there is encouragement now”.

M: So now we are coming to talk about those services attached to Ebola. The number one thing we are going to talk about is the Ambulance Service. I want you to tell me everything about it. There is the good side and the bad side of it, so which bad side of it do they talk about?

R1: “The good side?”

M: Mhmm, when it comes to collect people?

R1: “When they come to collect people and rescue them till they reach, that is the good side, the bad side is that when a person enters the Ambulance, the blasting of the siren alone and the spray that they spray, the break-neck speed at which they carry you until they reach with you in Kailahun (first district with Ebola cases in Sierra Leone), the person would have been hopeless”.

M: Ee, are they still carrying people to Kailahun (first district with Ebola cases in Sierra Leone)?

R1: “No they have stopped that one”.

M: Yes De Mami, what can you tell me about the Ambulance?

R2: “The good side of the Ambulance is when they come to take a person. But the bad side it affect all of us. Because when they come to take a person, that wayo! Wayo! Wayo, that voice (=siren), when you hear that now, is just like the gun boat during the war, when you hear the gun boat you have to go and hide. So also is the Ambulance. No sooner you hear the voice you know that they are coming to take another person or a person is dead. So we have that fear”.

M: Yes De Mami, what you want to add to that? When the Ambulance comes to take people the good things that they say about it?

R3: “It is fine, because when a person is sick they need to call for them to come and take the person. But blaring of siren everybody’s heart will get confused”.

M: That is the bad side?

R3: Yes the way it comes blaring, our hearts get “boxed up”.

M: Yes De Mami, do you have anything to say?

R4: “Yes the good thing, because it is the Ambulance makes when somebody is sick they can rush to come. But the bad side is the speed which they take, they shake you until you reach, by the time you reach you are no longer human being again. That is why many die on the road, which is the bad side”.

M: Yes can you tell me a little about the good side of the Ambulance?

R5: “The fine side is when they come everybody will be glad. But when they come with noise,that confuses the people. So everybody is afraid. Even my very self here, when they say they are coming, wherever I will be standing I begin to tremble”.

M: Yes De Mami, can you tell me small about the Ambulance?

R6: “When it comes to take a person we are glad about it or when the Patient enters the Ambulance, we can be there looking when they spray the person. But at this time they are not spraying again.

M: So De Mami too, what do they talk about the Holding Centre and the Treatment Centres?

R6: “The place is fine they clean there, but we have not had a patient there. You either carry him there or they come to take him. So the Centres are fine”

M: What about the bad side?

R6: “I don’t think there is a bad side”.

M: Ok, so De Mami, what they talk about the Holding Centre and the Treatment Centres?

R7: “They are holding them fine there. Because when a person is sick they can come and carry the person”.

M: So is there any bad thing that you hear people say about them?

R7: “No”.

M: Yes De Mami, you too?

R8: “As for me the ones I see they carry, they will be very worried for that patient until they see to it that he or she is ok. But if they notice that it is beyond their control they will call the Ambulance to come and take the person”.

M: Is there any bad thing people are saying about the Ambulance?

R1: “No, no,no”.

M: Yes De Mami over there, you too, what do they talk about the Treatment and Holding Centre?

R2: “The good thing is when you are sick you go there they will treat you and give you good medicine also. So that is what I know”.

M: What about the bad thing?

R2: “Aa, I have not heard any bad thing yet”

M: Yes De Mami, do they talk any bad thing about the Holding Centre and the Treatment Centre?

R3: “Yes, the last time when they carry me away, they encourage me. Because when they carry my child they encouraged me and told me not to feel bad. When I entered I washed my hands first and then enter inside. They have no bad thing that they did to me there”.

M: Yes De Mami in the middle, can you tell me?

R3: “The encouragement is fine, so people feel fine and the place is also clean”.

M: Yes the De Mami, can you tell me small thing about the Holding Centre?

R:4: “When a person is sick and they carry you there, if they see that they can take care they can give you medicine, if it is beyond their control, they call 117. And the people who are working there make people feel fine”.

M: So, I want us to talk small about the Burial Team.

*Rs: (grumbling that they have not eating anything from God morning)*

M: Please let us be patient, please. I want you to tell me about the Burial Team?

R5: “The Burial Team, at first when they come, they come with tension. Even ourselves before they reach us we could have run far away. They just go about spraying. When coming they protect themselves. Instead of going to spray where the person is laying, they go about spraying those who are not concerned, by the time you think of it, that person will die. That is one disturbing thing on the part of the Burial Team”.

M: What about the bad thing about them?

R5: “The bad thing is what I have explained”.

M: So you do not have any good thing about the Burial Team?

R5: Mhmm” (No).

R6: They come with confidence”.

R7: “The Burial Team, no sooner they come, they come for the work and nothing else, and when they come whether it concerns you or not, even if it is your person who has died, they will spray all of you. You have to run away from the place”.

M: So you too have no good thing?

R7: “Mhmm” (No)

M: What about you, do you have any good thing about the Burial Team?

R8: “I have no good thing about the Burial Team, When they come to take a dead person they .run with it, when they reach the cemetery, they just throw the body into the grave. I am not glad about it”.

M: What about you?

R1: “The Burial Team?

M: Mmm.

R1: “We are not feeling fine on that”.

M: What about you?

R2: “One, when they put you inside a plastic, hmm. At first, people were not buried in plastic but now when they go to burry they remove all their uniforms and boots and put it into the grave. If they do that to your own person, will you feel fine? You can never feel fine”.

M: Yes Ma, what do you think about this Burial Team business?

R3: “When they come, they come with happiness. They will dress in full view of all of us. They cover their faces and go about spraying. When you want to go nearer to the body they drive you and when they take it to the grave they just throw it inside and remove the entire thing they are wearing and put it inside the grave

Rs: *(“Laugh)*

R4: “Those Burial Team brought about “kus kas” (= conflict) here, if you ever heard that three sons from (- - Name of interview village - -)……?........ because of the reaction they took. Ok, our sister died but it was a quarantined home and we accepted that it was an Ebola case, but the treatment by this Burial Team made our children to react. Ok, that was my first time I see them dress, I cried. People blew off from crying. Then the son of the dead man was crying in the corner and they drove him away. Pa “comot” (= get out of here). But he is a human being! They went inside and sprayed. Even the clothes the dead woman was wearing they brought everything outside. That was too much! The Burial Team is not good at all, it is not good. Then one of the girl children of the late woman decided to confront the police who beat her with their gun until they went away with body. The girl pleaded with them to stop spraingy her mother since she has been sprayed inside. So they were not reacting good at all. God so good when the changes have come. Because this could have brought another war just like you heard that we were fighting each other here in (- - Name of interview village - -). She is a lady, very good lady; they treated her badly and that was the lady reacted to them”.

M: What about the 117 call line when they said you should call. What good thing can you tell me about the 117 phone line?

R5: “The 117’

M: Mhmm.

R6: “The 117, because when somebody is in that position when they come to take the person, hay, the blowing of the siren alone around the patient and what they do to the person before carrying the person...”.

M: Now is the phone line that I want to know about.

R6: “Oh the phone line”?

M: Mhmm.

R6: “Well at first they respond quickly. But now if your person is dead or a person is sick, like that of our brother right now he is vomiting blood, he is there.

M: Hmmm!

R6: From this 5 O’ Clock they started calling them up to now they have not turned up. They say they are now lying for them”.

M: Hm!

R6: “They don’t turn up again, even if you call them. For instance if a person die today and they begin to call them, the body will lie down until say this 5 O’ Clock coming, the person will be lying there”.

M: So you don’t have any good thing to say about them?

R6: “Mhmm” (No).

M:So let De Mami too say small thing about this 117 ya?

R7: “The only thing is, when you call them they haste to come. But now you call, one of these days an elderly woman died here it almost stink the house, people ran away from the house, you call them they won’t come. You see, and that is their duty. They have given the number then when you call they ask you question again. You see, even here yesterday when they call them they did not come, except one contact tracer who had to send(= given message to, brought message to) to the office. So they want to make a delay, let them don’t delay again the thing is now coming to an end”.

M: So you don’t have any good thing that you can say about them?

Rs: “Mhmm” (No)

R8: “No, somehow it is good because they come and in hast, but now I don’t know whether they are relaxing”.

M: Ok, let De Mami too say something about the phone which you call, that 117?

R1: “The 117, right now me as a civilian I don’t just call it like that, except they call at the old centre for them to come. That is the law they told us as Ward Committee. But the reason why they are always stubborn to come, they often fool them. In (- - Name of interview village - -) here I think they have fooled them three times. They can just call them and when they come they tell them that this is not the place where they called them. So now when they call them they waste time to come. So now they call the Holding Centre and the Centre will call like one of my brother, (- - name of brother - -) as he called they came and took him and they went. But if it is extra number (=……………) they will not come.

M:Yes De Mami, you too can “rub mouth into this” (=can you add to this) ?

R2: “Yes, as for me I don’t know much about those numbers but when I hear the Ambulance coming I know they have called it; that is why they come at first. Like now they are saying this because the foolishness they bring to them that is why they delay”.

M: Yes De Mami at the back there, can you tell me something about the 117 phone which they use to call?

R3: “Well in the past, when they call them they come quickly, but now when you call them they relax, they want the sickness to end now.

M: Yes De Mami, can talk small thing about the 117?

R4: “Just like this woman was talking, at first there was no delay but right now they delay”

M: Yes De Mami can add small?

R5: “Just like my sister have said”.

M: Please add your own small.

R5: “It is about the delay. Even if your person dies at night, the person will be lying until how much o’clock, not until they feel like coming before they come, that’s what they do”.

M: So you too have no good thing you can talk about them?

R5: “Mhmm” (No)

M: So what about those Health Facilities that they made, those Health Centres that were here before, do you have anything good to tell about them?

R6: “Like for us at our hospital, at first they were treating us. When you carry your sick child they can treat you. But since coming of Ebola, we can carry pregnant woman except those ABTs (= …………..). The nurses run away from pregnant women, just because of this problem, at first they were helping us but now”.

M: Yes Ma, you too what can you say about the 117, then the Health Centre which have nurses to care of them”

R7: “Anyway the nurses are now devoted. Some were afraid because their colleague nurses have died. So that is why, even us, we lost a very prominent lady here just out of this sympathy. She had many children, she went and treated a woman and she too contacted the sickness and she died. So the fear made plenty of the nurses had said that they want to resign, if Ebola did not end they will resign from and look for another work because they are seeing their colleagues dying. But now, they were working hard when there was free medical. They treat lactating mothers, they treat pregnant women, when you have a delicate sickness they refer you”.

M: Yes De Mami, can you tell me about the Treatment Centres and the Holding Centres? Can you tell me about the nurses, any good thing or the bad thing?

R8: “Now they are trying, we tell the government thanks. When your child is sick and you take her to the Centre they will treat the child. Even we the big ones when you are sick and you go there they can treat you. Pregnant woman when you reach there they are going to be worried about you”.

M: So you do not have any bad thing about them?

R8: “Mhmm” (No)

M: Ok let the De Mami too say something ya?

R1: “All the same they are treating people. At first, it was not like that but now when you go be you a child or lactating mother, they treat you fine, is just because of this Ebola that everybody is hesitating”.

M: Yes De Mami, you in the middle there. Those Health Centres that take care of patients, do you know anything about them?

R2: “The nurses”.

M: The nurses?

R2: “At first they were afraid but right now they are trying”,

M: So what I want to know is how do you react to people who have survived from Ebola? Yes De Mami, How do you treat them?

R3: “Well they do advice us that a person who has survived Ebola, you should draw him or her nearer and encourage him or her, console him or her and tell him or her what to do. You should not take him or her as an extra person, you should be nearer to him or her. He or she has been cured and will never get such sickness again so you should not push him aside”.

M: So are there not people who treat them badly?

R3: “They do not treat them badly”.

M: What about you Ma, what can you say those who have been cured from Ebola, how do they treat them here?

R4: “This is one of our sisters, look at her. We accepted her, because she took about one month..., two months in Kailahun (first district with Ebola cases in Sierra Leone), until they finished treating her. They first told us that she was dead, we all cried for her, later they brought her.

M: Hmm!

R3: “So we still accept her as our sister and we encourage her and she is in this community and at times she helps, though she has her trauma”.

M: Yes De Mami, they said you are a survivor, hoe do people treat you here? How do they react to you?

R4: (Ebola Survivor) “Well they are treating me fine, they are holding me fine”.

M: Nobody, is treating you badly not so?

R4: “, Mhmm” (No) nobody has treated me badly”.

M: Ok,yes, De Mami, can you small thing on how do you treat people who have survived Ebola”.

R5: “Mama, you can see them come and take someone and that person will not come back. If they carry you and bring you back , the gladness will not be small. You have to embrace them, draw them closer to you, do not provoke them. I know that our sister who went is back and has come, to meet us.”.

M: Yes De Mami, you too can add something to it?

R6: “They told us that when somebody is sick does not look at him or her side ways. We are glad for all this as it has brought us good health. We should be encouraging them”.

M: Yes

De Mami over there, can you too tell me something about how you treat somebody who has survived from Ebola?

R7: “If they come back with the person, I will be very glad. Because when they brought my child back on that night I slept long, I was glad I draw her closer again”.

M: Yes De Mami in the middle there, how do they treat people affected with Ebola?

R8: “Well, they are not threatening her badly, Is just that when they are treating her body gets white. Her body is not fine, but when we saw her we are glad. So we tell God thanks, the body has turned up now. She has weight now”

M: Yes Ma, do you have anything to say? When a person is cured from Ebola, what can you do for him? How do you treat him or her?

R1: “We do encourage him or her, be with him as before. We cannot make the person discourage”.

M: Have you heard about any treatment that is coming to cure Ebola?

R2: “Yes”.’

M: What have you heard?

R2: “They said people have come, the white man has come to help us cure this sick. And we are seeing that. In the past, many of our brothers when they go there they said there is no medicine, but now they have seen that when they carry a person, the person can be cured and return back”.

M: So what think about that when you said you have heard about the new treatment?

R3: “We are happy, people are glad”.

Ml You said bout the new treatment of Ebola, I want you to explain it to me?

R3: “I said yes, they said a new treatment has arrived, that white men have come to help us cure this sick”.

M: What would people think about that?

R3: “Well they will think that they have come with medicine to help them so they too can cooperate with them”.

M:So De Mami have you also heard about a new treatment to cure Ebola that will be coming soon?

R4: “We heard about it

M: What did you hear?

R4: “We heard that Doctors have come, and some more are coming to help us. Then they have directed us to First Aid, when a person is not well you mix ORS for the person to have plenty of water. Even these days, they gave us some which is a tablet”.

M: So do you think about the new treatment that has come, I want you to explain small about it please?

R4: “Well eh, we have not seen it yet”.

M: When they say if a person has Malaria he or she should take so, so tablet?

R4: “We heard about that

R5: “We have heard about that but we have not seen it yet, except this Malaria tablet that they gave us”.

M: So De Mami you too, you can tell me small thing about the new treatment?

R6: “Me, I have not heard about it yet”.

M: Yes, De Mami over there, have you about the new treatment?

R7: “I have not heard about it yet, only one we know about is to cure Malaria which they gave to us”.

M:De Mami you too, have heard about any new treatment?

R8: “I have not heard about it yet”.

M: What about you, haven’t you heard?

R1: “Me too, I have not heard”.

M: Yes you too have you heard?

R1: “I have not yet heard”.

M: Ok, thank you eh, have you heard about any new way to prevent Ebola apart from the ones you have talk about; to wash hands, don’t touch... have you heard about any new one?

R2: ”Me I have not heard it yet”.

M: What about you have you heard?

R3: “No”.

M: You too have heard about it?

R4: “No”.

M: Haven’t you heard anything about the new treatment?

R5: “Me is just her First Aid, ORS and Panadol just that”.

M What about you?

R6: “No, I have not heard about it yet”.

M: Yes you?

R7: “No I have not heard about it yet”.

M: You too, I want let all voices come into this recorder?

R8: “You have to drink plenty of water with ORS”.

M: What I mean, for you not to get it at all, to prevent?

R1: “No”.

M: What about the Marklate (=vaccine) , have you heard about any Marklate (=vaccine) for?

R2: “No”.

M: What about you, have heard anything about Ebola Marklate (=vaccine)?

R3: “Me, I heard about it over radio”.

M: What did they say?

R3: “They said they will be coming with a Marklate(=vaccine) for this Ebola, but I don’t know if they have started”.

M:So what do you think people will think those things?

R3: “Well, except if they educate us on this Marklate (=vaccine) because people are still afraid. Even the tablet which they gave us for Malaria (mass distribution malaria drugs, recent) a lot were afraid to take it. Some took it and the Malaria heavy they died. So before distribute the Marklate (=vaccine) let them educate us very well about the Marklate (=vaccine)”.

M: You too have you heard any Marklate (=vaccine) that is coming for Ebola?

R6: “No”

M: You too, have heard about any Marklate (=vaccine)?

R7: “I heard it over radio”.

M: What did they say?

R7: “They said they were going to bring Marklate (=vaccine) for us for your health care, I know many people were afraid, but this Malaria treatment that they gave us will make a lot of people not to be afraid again”.

M: So what do you think people will remember about those things?

R7: “They will just remember that if they come with those things is to cure us”.

M: What about you?

R8: “No”.

M: You over there, have you heard about any?

R1: “No I have not heard any”.

M: You in the middle there, have you heard?

R2: “No”.

M: What are the common things that people say about Ebola? I want to know.

R3: “They this is a bad sick, when it hold you, you will not live. That is what they talk and that is what we also talk, that what kind of sick is this when it holds a person the person will not live. Now that they are curing, they are living. We have all seen now that Ebola has medicine”.

M: Ok, so is there anything that people argue about? Which they do not agree upon about Ebola?

R4: “Yes, they argue because of the symptoms. When they said this dysentery has been there before, blood oozing out of the nostrils is natural it thus happens. So people do argue that these kinds of sickness have been there before and were cured”.

M: What about the prevention and the treatment, is there anything they argue about it?

R4: “Yes”.

M: Mhmm.

R4: “Some do argue that about the frequent hand washing. They said the wash hands is like where ever you go you come to wash your hands. Some argue that it is idealness; some will tell is because you do not have a work to do that is why you wash your hands all the time. You touch this you wash your hands”.

M: So what about the treatment, are they saying anything?

R4: “Well, the treatment, I am not too I have not seen too much argument”.

M: So, De Mami you too, do you hear about anything that they say about it?

R5: “Like this gun pointing that they are doing to test temperature (note: this means use of infrared no touch thermometer, looks a bit like a gun), saying that this is not the first time that they are taking their temperature”.

M: So is there any common thing people discuss, that they talk about Ebola?

R6: “Yes”.

M: Yes what do they say?

R6: “Some of them, still plenty do not want to believe that Ebola is real, so they put up those arguments.

M: So is there anything that they argue about the treatment?

R7: “This treatment”?

M: Yes, when they go to cure them?

R7: “Yes, they say the sickness is a combine sickness because people have been sick; dysentery, there is the Malaria, all that is a combination put together they say is Ebola”.

M: Yes De Mami, can you too say small thing?

R7: “The only argument which I have heard is those who have been discharged and given three months not to touch a woman, they say is a lie. And the moment they touch a woman the problem comes back. I have heard that argument. They say after treatment no sexual intercourse for three months. You have to prevent yourself for three months, you should not have sex. Some men do deny, they go ahead to have sex, and as they do that the sickness will attack the woman again”.

M: So is there anything which they discuss about the prevention of Ebola?

R8: “No they are no longer arguing about it again”.

M: What about you?

R1: “I plenty because the way how Ebola came and the way they are treating it people say all these sicknesses have been happening. That when you sick two or three times means it is Ebola. That they do not believe. They do discuss it but..”.

M: What about argument on the prevention side, what do they argue about?

R1: “No, I have not heard that”.

M: Ok, De Mami too can add small thing there ya. Is there anything that you hear people talking about Ebola?

R2: “The body contact, people do not believe it. Secondly again you have to prevent yourself to wash your hands”.

M: So what about the argument, what do they argue about?

R3: “Some compare HIV to Ebola. They say just like how HIV came it was powerful, but now Ebola is more than it. When they say HIV is a combination of illnesses that causes HIV. Now Ebola has come, and it is the combination sicknesses that causes Ebola. But this now has killed a lot more than HIV. That is the way they argue”.

M:Yes, De Mami, when passing by what do hear people discuss about Ebola?

R4: “Ee, some say when cholera came we didn’t have too much worry like this sickness, some have never seen the example of this thing. Vomiting, blood oozes out of your nostrils. When there was cholera people used to vomit, toilet, dysentery, you vomit blood, but it never had too much worry like this one’.

M:Yes Ma, what do you hear people say about Ebola?

R5:”I don’t hear anything”.

M:Is there anything that people need to understand about this Ebola?

R6: “Me the only thing I want all of us to understand is Ebola is sickness and it kills. That has been understood because it has been killing people”.

M: So which way do you think we can explain this people so that they can understand it better?

R6: “I will say it by mouth, I will make a postal and draw , even if it is in a classroom I will demonstrate to the children for them to understand well that from the bat to this, to this. And when you get it this is what you should do”.

M: So De Mami you too can add something ya? When passing, what do you think people need to understand about this Ebola?

R7: “Like the shake hand, you avoid the contact. Make a vanguard and poster you attach it to the wall and begin to teach the children. You teach them how to prevent it, hand washing. When you go to toilet and come back you wash your hands.

M: Ok, De Mami you can add small to that ya, what do you think people should understand “good good one” (=very well) about this thing?

R8: “Body contact, that one is a problem”.

M: How do we explain that to them?

R8: “How to explain it to them”?

M: Mhmm.

R8: “One thing if you have your husband, stay with your husband. If you have your boy friend with whom you have dated for long stay with him or you the man stay with your woman. Then you go and a body contact, you will take and carry it to your woman”.

M: Yes De Mami, you too, can you talk something?

R1: “Eh is the same thing. This “shake hand” is creating all these problems”.

M: Well, how do we tell them?

R1: “We should only tell them not to do what they say is not fine. What they ask them to do is what they should do”.

M: How do we tell them?

R1: “Well we have to tell them that body contact they have to avoid that”.

M: Yes De Mami can you tell us a little? What do you think people understand better about this Ebola?

R2: “They should not deny that the sickness is real. You should tell them the truth that if he or she does this, this will be the result”.

M: What can we do for thrm to understand what you tell them?

R2: “You should not touch, you should wash your hands”.

M: Ok, De Mami, you too can add small thing to that ya?

R3: “We the elderly people, we do not have a problem because we have seen the examples that they show us, but these small kids have the problem. Like some children, they go to toilet without carrying a soap or water, but when there is light at the end now we are trying for the others”.

M: So how do we present it to them so that they can understand?

R3: “We have to tell them to prevent”.

M: So De Mami you are the last person to talk on this, so what do you think people should understand better about this Ebola?

R4: “They should understand that Ebola is real, but still some people are denying, you see because they have seen people sick of Ebola and died. So they should understand that. Then they thought us also how to prevent it. You should not this, you should not make like this. So you should tell them all these things”.

M: How do we tell them?

R4: “Tell them that if you do this, it would not be fine, you will be held by Ebola. You have to clean yourself, you should prevent yourself”.

M: So, I thank you very much for patiently listening to the interview. Just like I told you, I don’t have any immediate something to give you now, but the information which we are gathering, we must take action on it ya. We will take it to the appropriate authorities ya. May God bless you ya?

Rs: ,Mhmmm.
