# Supplementary material for: Development of a set of community-informed Ebola messages for Sierra Leone
Source: PLoS Negl Trop Dis. 2017 Aug 7;11(8):e0005742. doi: 10.1371/journal.pntd.0005742 (PMC5560759; doi:10.1371/journal.pntd.0005742)
Supplement: S1 Appendix — (ZIP) [file pntd.0005742.s001.zip › Ebola messages - FGD and interview transcripts/R2HC Ebola Fieldwork 1/R2HC Ebola F1 FGD-FEOLD-Urban2 V2 ADD PROBE.docx]

| CODE | **R2HC Ebola F1 FGD-FEOLD-Urban2 (urban focus group discussion)**  **V2 – 11^th^ March 2015 – ADD PROBE and correction personal data** |
| --- | --- |
| DATE | February 2015 |
| DURATION (minutes) | 60 |
| Collector nr | 4 |
| LANGUAGE INTERVIEW | Krio |
| **TYPE FGD** | Older Females |

**PERSONAL DATA PARTICIPANTS**

| Nr | Sex  (*F/ M*) | Age  (*in years*) | Education Level (*e.g. none, Primary, secondary, tertiary*) | Language (*e.g. Mende, Temne, Krio)* | Religion | Job / Employment (*how they earn their living e.g. farmer, teacher, trader*) | Role in community  (*e.g. youth leader*)  ANONYMIZED, ONLY AREA OF ROLE INDICATED |
| --- | --- | --- | --- | --- | --- | --- | --- |
| 1 | F | 30 | Tertiary | Krio/Mende | Christian | Teacher | None |
| 2 | F | 47 | Tertiary | Krio/Madingo | Muslim | Trader | None |
| 3 | F | 29 | Tertiary | Krio/Limba | Muslim | Driver | None |
| 4 | F | 40 | Secondary | Krio | Christian | TBA | None |
| 5 | F | 50 | Secondary | Krio/Temne | Christian | Trader | None |
| 6 | F | 52 | None | Krio/Temne | Muslim | Trader | None |

**TRANSCRIPT: (M = Moderator, R= respondent, R1= first person responding to a question, DOES NOT correspond to numbering used in Personal Data!)**

M: How has Ebola affected this of your community in which you are?

R1: “It affected us greatly, it made some our good people...(*not clear*)

M: Yes my sister can you add something?

R2: “Well Ebola has really been difficult for us, like we lost most of the big, big people who are respectable people in this area, it made some people to even talk to each other that there is always a barrier between us that this person is Ebola and that has created so much enmity and we are not happy about that at all’.

M: Yes my sister can you add something there? How has Ebola affected this community?

R3: “Well the closing of schools, the 6 to 6, there some market women is around 6 to 8 that they do get sales but due to this Ebola they came up with this State of Emergency. So there is the barrier really, so like in our own area it reached a time when those who are down there will not be allowed to come up, even if you beg water to drink they will broke the cup. The person would not want to use the cup again because of the area the person is coming from”.

M: Ok, yes my sister can you give us your own view on how Ebola has affected this community?

R4: “Just like what this woman said, it has pushed the school business behind, no business”.

M: Yes my sister how has Ebola affected this community?

R5: “It has pushed school business behind and has killed some big people in this area”.

M: Yes my sister, how has Ebola affected this community?

R6: “It has affected us greatly because it is not only the community, one the closing of schools, two the hospitals people are afraid to go to hospitals or houses think that if they go this house and most of the houses which they quarantined they won’t allow us to go there. You can only stop at the street if you want to talk to them but some people after this quarantine when you pass by and did not greet them the next time when you pass by they would not even want to talk to you. That has even brought some enmity between people”.

M: Yes my friend, can you add small thing?

R1: “Yes, if can buttress what my friends have said this Ebola has affected us greatly. If I can recall I am also a victim, victim in the sense not that I am infected but the Ebola is now, they quarantined my people I am not glad about that, though nobody did not die but sister do get sick, that brings enmity and people were watching at that kind of way, I did not feel fine, and I know issues of Ebola and unto this moment I am feeling fine. Right now I am thinking of going to work or to graduate now, nothing like that, I am not feeling fine over this virus”.

M: Yes my sister can you too add to how this Ebola has affected your community?

R1: “Yes based on what my sisters has just said, where I am was a hot spot, the last house which we share boundary was affected, almost I can say my brother has dies, my good people died and it created a lot of enmity. Now certain people say we were mocking them that they have Ebola, certain people now do not talk to each other because of Ebola. This Ebola all we can do is to pray for this Ebola to end so that can move freely in the country again”.

M: Do you personal know anybody who has got Ebola?

R2: “Yes, my friend”.

M: Yes too, do you know anybody who has got Ebola?

R3: “Yes”.

M: Yes my friend, do you know anybody who has got Ebola?

R4: “Yes my sister, do you know anybody who has Ebola?

R5: “Yes”.

M: Yes my sister, do you know anybody who has it?

R6: “Yes my neighbour”.

M: Yes Mama?

1: “Yes, I know many people who have fallen victim of this Ebola”

R2: “Yes me too I know somebody who has got Ebola, my aunty”.

M: Yes my sister?

R3: “Well like we were saying our neighbours”.

M: Ok, what I want know again is why has Ebola spread in the country?

R4: “By touching”.

M: Ok, why has Ebola spread in Sierra Leone?

R5: “By washing dead bodies “.

M: Yes my sister why has Ebola spread in the Sierra Leone?

R6: “The too much spray which they spray”.

M: Yes, Ma?

R1: “Some fail to listen to instruction they gave them ways and means to prevent this Ebola sickness but people fail to according to the rules they don’t even believe that it is a virus that exist so with this stubbornness thing has spread’.

M: Yes, my sister can you give me reasons why Ebola is spreading in Sierra Leone?

R2: “Is because of the carelessness of the government, they failed to put things in place, what I am trying to say of cause we know that this sick started in Kailahun if they had used their initiative to quarantine them this sick would not have spread but they were late and now when they want to eradicate this sick is too late. So that is my view”.

M: Ok, yes my sister can you too give me reason why has Ebola spread in Sierra Leone?

R3: “Because people do not believe what they are saying. Even when they say the sick is real some people deny that it is not real”.

M” Yes my friend you too add something as to why Ebola is spreading in the country?

R4: “Anm, this directly to the government, just like what my sisters said, because in neighbouring countries like Guinea and Liberia has Ebola, they could have locked our boundary because as the thing Yahya Jammeh (= president of Gambia) closed his boundary unto date it is closed nobody cannot go inside Gambia freely. So I think this is one of the most reasons why we were affected”.

M: Ok, so among the local languages that you talk, do have any name you call Ebola in those languages?

R5: “I only hear Krio; I don’t hear my own language”.

M: Ok, do you have any local way you call Ebola in your language?

R6: “I have never heard them call Ebola in another language except Ebola in Krio”.

M: My sister you too?

R1: “No”.

M: Well my sister middle there?

R2: “Well I never heard except one way they call it in Temne (*speaking Temne*)(…………………………)

M: I want you to give me the meaning of what you have just said?

R2: “It means that it a sickness brought by God whether you do bad or not when you fall victim you just have to go”.

M: Ok, my sister, do you too have any way you call Ebola in your language?

R3: “Is just the same, Ebola”.

M: Yes my sister?

R4: “I don’t know any other way to call it except Ebola”.

M: Yes my sister?

R5: “Is just the same”.

M: There are people who do not believe that Ebola is real, do you know of such person who do not believe that Ebola is real?

R6: “Like before, even I thought that Ebola is not real I took it that they are just creating that the sickness is real except when it killed my friend and when I came to (- - name of interview community - -) the area that I saw where it killed and people died that was the time I knew that Ebola is real”.

M: So those who believe that Ebola is not real, why do they think that Ebola is not real?

R1: “Ok an example is myself; why I said Ebola is not real because by then it had not killed any of my family yet, and I have never seen the signs yet, I have never. But Guinea they said when a person has Ebola. You can see the signs the body get swollen and I have not seen that so I never believed’.

M: Ok, my sister do you know of any who has the belief that Ebola is not real?

R2: “Like the compound where I am my uncles were denying that Ebola is not real, I have one of my uncles who is always denying that Ebola is not real’.

M: Why does he deny?

R2: “Well he said the way they show people with Ebola on the television, the way they look he said he has never seen affected person that way, when their hands get swollen and they have sore on their body. The last time they came and played for us a band and they showed people with Ebola but the way they showed those people, I have never seen such thing with somebody whom they say has Ebola. So that is why he never believed that the sick is real”.

M: Yes my sister, can you add small thing to that?

R3: “At first. Because they said Ebola has no medicine later they came back and said when you go to Treatment Centre you can recover quickly that brought about the confusion people began to argue that Ebola is real, some say it is not real”.

M: Yes, my sister you wanted to say something?

R4: “Yes even myself I was denying that Ebola is not real because if anybody has Ebola he or she cannot walk or do anything so I did not get the believing that it is real”.

M: Yes Ma?

R5: “Yeah, really at first people did not believe that Ebola is real because what they are telling people about the signs and symptoms like when you bleed, you get frequent stool, frequent vomiting. All of those signs somebody can get them and then the person dies, because they are saying that when somebody shows those signs it is Ebola. So all those signs somebody can get them, the person can also sick and without showing all those symptoms so many people did not believe that it was Ebola maybe it is just another sickness that has come so people never believed. Even dead body when you tell somebody that when a person dies do not wash it, they still do not believe because maybe the person who died did not die with any of those signs and symptoms so they believed that they should go and wash the person so when they wash it they later come to know that it is Ebola”.

M: Yes my friend can you add small thing to that?

R6: “Yes Ma, it is based on the translation of the messages which they are giving because at the time when thing started in the neighbouring countries they told us that all opening on human being will ooze blood and we have been seen that and then at the end of the they came and translated that to vomiting, fever and those things and those things are normal sicknesses that we have been getting because I remember each and every year we used to do sensitization on for Cholera but this year there was no sensitization for Cholera only Ebola. So then most people were sick of Cholera at the end of the just like how they were sick cholera is just the same and when they go to the centre when they do test there they say it is Ebola and me till date I have never deny that Ebola is not real and I have never believed that it is real. I am confused, I am really confused. One if they can go to the radio and tell us what is Ebola that can be more preferable than this”.

M: Yes my sister you too want to add?

R1: “Yeah, although what I want to talk is just the same as what my sister has just said. We have some people in this community who are still not convinced that Ebola is real. Do you know why? This sick they are talking about because when somebody Has Malaria you do vomit, your body get warm, and can get high fever according to what the Doctors are saying, you get head ache and when they say when somebody is affected you bleed, when somebody has sinus, that chronic one blood will come out of the nose so if they take all those sicknesses and out them into this only Ebola virus hand, when somebody body is warm, has head ache, his nostrils running you know’.

R: “Any way, we have doubt over this sick only God can sorry for us”.

R1: “But why we do agree is that because the big ones are saying is the sick so we just have to abide by it’.

M: Yes my sister you too can add small thing to that. Do you know anybody who does not believe that Ebola is real?

R2: “Me I used to deny, and onto date I am denying that Ebola is not real”.

M: Why are you denying?

R2: “Because they way they explain the way the sick attack people I have never seen those signs that is why till date I am denying that Ebola does not exist”.

M: Yes my friend?

R3: “Like me, my uncle fell sick and I was holding him in the hospital until he died and the son of my uncle about five years when I came back after few days I fell sick but when I fell sick if I had gone to the hospital they could have forgotten about my life, because I having frequent stool and vomiting. Those were the only sick that I used to get but thank God my sister was close to me, everybody ran away from us, they were the only ones with us, we kept working secretly till I got well as I felt better then my husband fell sick again, he too it was the same thing he was treated at home and he got better just after him two of my children also fell sick, the other was not going to toilet and the other was vomiting, the other was just lying down without vomiting but cannot toilet and his body was warm. Like my sister who was taking care of me she did not even sick she was just colic, but she died in my hands. So I am confused about this talk about Ebola, I do believe but I do not believe again. So that is it”.

M: Ok, so what I want to know again is about those Ebola messages that you hear, I want you to give me small examples of them?

R4: “I don’t really understand”.

M: The Ebola messages which they have been giving you or which Ebola message has they ever told you?

R4: “They only told us that we should not touch our companion, when you touch your companion you will get Ebola, you should not wash dead body and when you when your person dies you should not go to greet because when you gather to gather even if your companion touches you, you are going to get Ebola, something like that”.

M: So what do you think about those messages which they gave you? What is your personally thinking about it?

R4: “Lie I told you I do not believe, so I used to touch. Like those who are close to me I used to touch them I have no fear about that”.

M: Ok, so my sister can you give me some of the Ebola messages that you have been hearing?

R5: “What I heard about this Ebola is that they said when you have high fever that means you have got Ebola, when your head aches you have got it. So like those things I don’t believe them at all because since the outbreak of Ebola cold is my sickness, my body gets warm which reached a point when I vomit the last time a boy was passing and he said ay (- - name of female person - -) you are vomiting and then I told him not shout about it. So like those things can take place, is just basic things it can happen. There is nobody who can tell say his body never get warm, or has not been caught by fever, so like I don’t believe that in the first place, it is a lie for me”.

M: Yes Mama can you too give the Ebola messages that you have heard and what you think about it?

R6: “Yeah, the messages are coming in and they are well understood but they are normal signs which have been happening “.

M: Give me some of the messages that they have given you?

R6: “Like they said don’t touch when somebody’s body is too warm is Ebola, severe head ache is Ebola’.

M: Ok, so what do you think about those ménages?

R6: “Well, I think that they are normal signs which has been happening but when they say they are signs of Ebola, we are abiding because there was a time when (- - name of interview community - -) was a hot zone, even when you want to come near me I will tell you not to touch me, do not talk near me o”.

M: Yes my sister, give us the Ebola messages that you have ever heard?

R1: “According to the messages which I have, they said this virus when somebody has it, if he or she hasn’t got fever yet, high fever even though I touched the somebody the virus can never transfer to the person unless when the fever is so high and then you begin to see that he or she is vomiting, toileting then you can avoid the person. So I just thought to fit that it is true because when the person vomit and you play with vomit you are going to get the virus, when you play with the stool you get the virus so I thought it fit that we should avoid so that this thing will stop. Because when it enters into a family all of you will go if you did not take time, if you did not abide to the rules obviously all of you go”.

M: Yes my sister who has that fine phone at hand, I want you to give me small Ebola messages that you have heard?

R2: “Well they have been advising us that we should not touch dead body, even if the person is your mother, just call 117 for them to come and take her to the Treatment Centre, that we should always wash our hands with chlorine, soap and water. About those things, I never believed, but I have to protect myself because I like my life I just abide to what they tell me and a day like today even though Ebola is not yet finished but I thank God”.

M: Yes my sister, gives me some of the Ebola messages that you have heard?

R3: “Yeah, the messages which I hear is that when somebody is sick do not touch the person, when the person has high fever and more likely when somebody is at the point of death and the worst when somebody is dead when you touch the person they say the virus will transfer because they said the virus lives on blood. If they ask me to name this sick it is vampire because is only vampire that sucks blood so they just name it Ebola but it is vampire. This virus just wants blood doesn’t want water, doesn’t want flesh just blood, so”.

M: Yes my sister, give me some Ebola messages that you have heard?

R4: “We only want this sickness end in our country”.

M: Ok, so which good talk can you tell somebody who has a sick person to encourage him or her to take the person to hospital?

R5: “I will tell the person because lately, people have been cured and discharge now. It is not like before when they go with your relative for you to see him or her it will not be easy but now when they go with a patient the possibility for him to survive, especially if the person goes there by himself has 50% chance to live”.

M: Ok,

R5: “When he goes by himself is something like 70% chance, so I think that I will advise anybody who has a sick person for now to be able to go with him”.

M: Ok, yes my sister can you tell me anything about this talk? If person is sick of Ebola what would you tell his or her relative for them to have the courage to take the person to go hospital?

R6: “Ay God, truly speaking, this is inside my heart because I have heard that when they carry people they kill them there at the hospital because some people can get better then he or she can call his or her relatives that he or she is feeling better in one or two days they called back to say he was dead. So like me what is in mind I wanted him to be treated at home”.

M: Yes my sister, which words of encouragement can you give to somebody to encourage him to take sick person to hospital or to the Ebola Treatment Centre?

R1: “Well if the person is at home we cannot ascertain that it is Ebola or not because the signs and symptom which he or she has he or she was having the same thing before the coming if Ebola so like if he or she sees that he or she is getting high fever, head ache so like you just have to convince him or her to go for treatment. You can tell the person that it doesn’t mean that when you have fever you have Ebola you can simply go to hospital for them to go and treat or for them to check you they will confirm whether you have Ebola or not. If they go there again feeling strong the next day they say they will inject them on their big toe before you think about it for some hours the person will die. Like me, the person is my own somebody, I have heard that, that is what he does at the hospital, when never wanted him to go there”.

M: Yes my sister can you add something to that, things that you will tell people to convince them to take sick patients to hospital or the Treatment Centre?

R2: “Before they said they used to inject people for the virus not to spread but when the expatriates came people are been discharged although in our community out of 100% who went only 45% returned 55 died so we live everything to God but all I can advise is if a person is sick carry him do not wait until it is worst at home”.

M: Yes my sister, you want to add small thing?

R3: “My own advice is that if there is somebody is around who is sick in the community when you sit at home and they begin to ‘pepeh doctor’ (= doing home treatment) you then you are adding to spread the virus, the earlier you go the better, you go to the hospital and try to sick medication because this does not only go for one person when it enters into one family it will make sure it kills ever member of that family so to avoid that the earlier the better the sooner you get infected you have to go straight to the hospital”.

M: My sister add small thing to that?

R4: “If it is me I will advise the person that he or she must courage the person”.

R5: “ As for me I will just advise the person and tell the doctors if there is a way let them carry him to (- - name of a treatment centre in interview district - - ) because there are white men there because according to the survivors who goes there say they take great care of patients there while the other Centres we get bad news about them that they do not take care of them and you survive they will give you money when in actual face even when you were not getting anything to eat but for that two or three months when you come back some people will be afraid that this person has got Ebola so I am not going to go near him, you will have something that can keep you going, while the other Centre they will not give you they hold on it”.

M: Yes sister please, can you add small thing to that?

R6: “I will tell the person that if the person is sick they have to carry him or her to the hospital and for you call 117 for the person and him make sure that he or she did not touch the person and make sure they give him jelly water until they come and take that person”.

M: Ok, so what can be the best channel for us to give people Ebola messages that they can get it quickly?

R1: “Radio”.

M: Why did you say radio?

R1: “Well, especially when it is 6 O’clock everybody wants to listen to the radio to know the update of the result, so radio is now part of people’.

M: Ok, very good. Yes Ma, apart from radio, is there any other channel that we can use?

R2: “Mouth, because when I say mouth because if you have a word here you will not know how it will get to (- - name of a central point in the interview district - -). So it is like not everybody that has access to radio, some people will prefer when somebody is standing and you tell the person than the person listen it on the radio”.

M: Yes Ma?

R3: “I just want to buttress to what this woman has just said, I just think fit that we who are in each of the communities, they should be able to use people in the community to talk to the people, because I am able to know this woman because we are used. I can be able to pass the message, she can understand it and accept it, even if she did not accept it at that moment but later she can say this as we are here together she would say let me just think about it, than you go and take somebody who would say please let me hear from a distance. But if you use somebody who is staying in the community I think he or she can pass on this message through to the people”.

M: Yes my sister?

R4: “Just like what my sister was saying, let them use us or we do it to ourselves, let us pass the message and the most important one is those social networks because now I know that even the least child has access to internet, them even through radio let them just find a way that the message can spread, which is the most important thing”,

M: Yes Mama, which channel can we use?

R5: “Like we believe those megaphones and we have youth here, like me I want a job, if they come here and take 20 or 30 youth men for us to spread the message, we can spread it even if you do not want to listen you will listen. Like you if I meet you I have a way to encourage you and how I come with the message to you but this now like I am telling you this government is careless. At overseas those who think they can pass the message if they come to this community and give us say Le 100,000 (21feb2015 about 23 USD) each we can spread the message even if it is two or three days. We are here we don’t have jobs and we are spreading the message which is going down well with our people because of some of our people are Temne and most of us speak it but on the radio maybe they only talk English or Krio, even if you have radio how are you going understand the message, but if go to a compound and talk Temne so you see how the message works and go down well with the people? But this government is not serious”.

M: Yes Mama, can you add small thing to that?

R6: “Like what my sister just said, for them to involve the youth for them to go and spread the message. That is just it”.

M: Ok, so if a person has Ebola, where would the person think of going first? Would the person think of going to the hospital or will go to a ‘meresin man’ (=traditional healer) or will go to the existing Treatment Centres or will go to the Ebola Treatment Centre? Where would the person think of going first?

R1: “Is to those Ebola Treatment Centres will be the first place the person thinks of going”.

M: Yes Mama, if a person has Ebola where would he or she think of going first?

R2: “He thinks of going to the hospital first”.

M: Ok, yes Mama of a person has Ebola, would he think of going to Pa (- - name for traditional healer - -) or to the Health Centre or to the Ebola Treatment Centre?

R3: The person will think of going to the Treatment Centres but at the same time the person will be afraid”.

M: Yes my sister where would the person think of going first?

R4: “Well like for some of them when they know that they have got Ebola some thinks that they have sent a sick on him so he or she goes first to the ‘Moray man’ (= Traditional Healer) for him to look what is going on with the person”.

M: If a person has Ebola, where would his or her heart run to go first, would he go to the ‘meresin men’ (= traditional healer) or to the Ebola Treatment Centres?

R5: “If you know that you have Ebola and you know that some people have been going in and coming out, the next thing you have to do is to go to hospital, but if you did not know that you have Ebola and that is just another sick, believe you me the person will find the native doctor first”.

M: Yes Aunty, you too add small thing to that?

R6: “Yeah, if I can add small thing to that, at first when this sick came some people when they get the signs and symptoms they go to ‘moray man’ (=sorcerer) and even in this of our area that is what that killed many people at the place where I told you that my father is living 90% of them are infected. The woman was a pregnant woman she runs up line so when she came she was infected with virus but what made it to kill many people the woman was not going to toilet or neither pass urine so they said “she been nailed” in our traditional Temne language so one pastor went and prayed on her and even the Sowe (= *key person in female secret society*) woman who was there died, so that is it many people died because a lot of people did not know because of the way they are passing the message, just lie I am telling you they said when you have Ebola you toilet and vomit. The woman was not going to toilet neither was she passing urine so they said it was not Ebola. So a lot of people, if you go down to us they will tell you that woman killed more than twenty people just because of this. So that is it”

M: Yes my sister, can you add small thing to that, if somebody has Ebola where does the person run to go first?

R1: “Well before you go to the Treatment Centre because even if you are feeling it you will not know that it is Ebola you just have to go for a test to the nearby Centre they can take you now to the Health Centre for the Ebola”.

M: So, I want us to talk about those services that are related to Ebola, like the Ambulance Service, what you can tell me about it, the good thing that people talk about them and the bad thing. I want you to tell me first about the good thing and then the bad things later. Yes my sister what can you tell me about the Ambulance Service, the good things that they do?

R2: “The good thing that they do is to come and collect people and carry them, then the bad thing which they do is some of them there can be no patient in the vehicle for them to make it fearful for everybody to get worried they will blow their horn win-won”.

M: Yes my sister what do you hear them talk about the Ambulance?

R3: “The good thing is that the Ambulance is fast in terms of speed, you understand? And the bad thing is that chlorine because if somebody is not well when they spray that chlorine maybe the person do not have Ebola and some people have high blood and then you try to suffocate him or her and you squeeze them into the Ambulance before they reach with them some die on the way. So I do get that complain from different community”.

M: Yes sister can you too add small thing to that? The Ambulance Service, what do you think they talk about them, the good side and the bad side?

R4: “Well the Ambulance, because there are enough Ambulance now in the country. I remember when Ebola started they used to say the people do not come on time and that is why many people died because there was no good treatment but now they come on time. But the bad thing which they are doing to people is that at least when somebody is sick the person needs care, needs attention. Some times when they are sending people into the Ambulance they just lift the person like when you are lifting a dog which you do not want into your house because some dogs have respect at their house, they will just take it send it. One woman they who died, the woman was sick and cannot walk in the community when they came instead of them helping her, because they are already protected, they are paying you for the job - you need to do the job well. Instead of helping the woman they did not do it, the woman’s husband came to help up the woman into the Ambulance. So you see, I wonder if they had taken that woman and take her direct if God did not destined her to die she could have saved. But they went along taking people and I think the best thing they should be doing when you take one sick person go and leave the person, there are many Ambulances but they would not do it they go along taking people, it is bad”.

M: Ok, eh, what about the Holding Centres and the Treatment Centres where they carry the patients, what do people say about them?

R5: “Well, before they were not treating people too well because they too were afraid of people. When a sick person goes, some [people just go because they need the money so they were not treating people well. But now we are hearing good information that they are treating people well, they are not injecting people on their toes again; people are now discharging”.

M: When you said just now that some people go because they need the money are you talking about the Staff?

R5: “The Staff, the nurses”.

M: Yes sister, you met us talking about the Treatment Centre and the Holding Centre what do people talk about them?

R6: “Yeah, I can talk about the bad things that they do. They used to say the first thing which they say is that when you go they are going” to inject you on your last toe” so that you can die. But at this later time when I went there I heard the when you go they will take you to one area call Kerry town and there they treat people fine and feed them three times a day and when you are to discharge they give you small, small things, they will give you certain things that makes you happy”.

M: Yes sister?

R1: “Yeah, the bad things I am hearing about the staff, when they admit somebody the way of their dress, the code of their dress can be fearful and some people have heart problem when they see that they become afraid and get panic, even if you do feel and the way in which they pass the food, pass the food in the sense when they come with the food they way they dress you cannot see the individual’s face to say he or she will be able to courage the person to take heart, you cannot bear it, it will look like you are in hell, your chances of survival is less because if they dress in that fearful way, how would you communicate, to even send a message to someone who is taking care of your child for her to take care is not possible. Because of cause in the past they used to do it but now when you see the face you can be able to talk to him. As you see them, some people even comment on them that they as you see them your heart will jump and you will be discourage. So that can make people die by that”.

M: Ok, so let us talk small about the Burial Team, I want you to tell me small thing about them, the good thing and the bad thing?

R2: “Well the Burial Team I believe that they never educated them. When they come to come and take dead body, in the first place some people died by stress when they come to take somebody they spray all over the house whether there is food or not, and in the past they were not changing people, when they come they spray the whole house, they would not even advise that when they finish spraying the house you stay for some time and do not enter there. They take body, human body, certain people do not have respect for the body; they take it and fling it, if you hear the sound – “VAAP!” (= imitating the sound) So just like one of my sister died when they went to take her, the Red Cross have small regard to bury people, they dress it, pray over it they the people who came to take it. They put it down quietly and took it away. Then, when they spray the house they advised the people not to enter there until after three days. Then the Red Cross came in to help the Burial Team it looks better, the death cases reduced”.

M: So the Burial Team is divided into two?

R2: “Well I don’t know whether it is two or even three, the Red Cross are there who can assist people. When they spray peoples place they will advise them not to enter there. That spray used to disturb people”

M: Ok, can you tell me anything about the Burial Team?

R3: “Well like for me that of my sister who died I was not able to see her or even where they took the body because I was not in good condition so I had to pull out, but like this they were there, as to how she explained to me that she was treated fine and they advised her husband to go and see how they were going to bury her but him too was not feeling bright so he was unable to go”.

M: So what about that 117 which they said you should call, I want you tell me small thing about them, the good thing and the bad thing about it?

R4: “I remember at one time I was at (- - another community in the interview district - -) and somebody died in (- - another bigger town in the area - -) because they had said when somebody dies let no one touch the body until they call 117. They called them and the body has been there for three days and had swollen to get burst nobody did not go there, his wife even went on air and said it that they have called them to go and take the body and till that time the body was lying there it was almost stinking there and they could not go there to collect it but they had said when somebody die they should not touch it they should call 117 and when they call them they don’t go there and at times they answer the call and they will tell you they are coming but they would not come there. So you see all that is not good and that even makes that some people when their relative they will go and wash it and bury it because you cannot leave it there to stink and a dead body is not suppose to be at home when the person is dead they should take him to go and bury him, so tomorrow even if somebody in the vicinity where you area you can take it to go and bury it”.

M: So you have not heard any good thing about them?

R4: “No”.

M: Yes my sister you too can add your own view, one by one, what can you say about the 117 Line?

R5: “All what they are saying is true, they do not have one regard for people. This sickness is spreading, we need to take the challenge now that is what they have said although yes we were hearing that when they call them to certain area like (- - name of interview community - -) if you call them to say somebody is dead when they are coming the particular number which you used to call them can be off they will come search and they would not find any dead body so that is why when they call them in certain areas they would not go there. But I know that even a teacher, they student can make fool of him in class, so if you are employed for the job, do it”.

M: Yes my sister, the 117 Line

R6: “Well like I can say much there because like I am not dealing with that area, because maybe is the way the message does pass. Before they do not come on time but at this later part to mu own observation some time they come on time same time they don’t But there are some areas I believe that it is the passing of the message that is crating the problem or the way the person deliver the message maybe the person forgot because anything that eat salt can forget but one thing I would advise them is that let them know that they are dealing with life and they too have life. So if you value your life then you have to value the lives of your brothers and sisters’ life”.

M: So those people who are Ebola survivors, how do people react to them?

R1: “They used to tell us that we should not push them, let us encourage them nut now they said they are the ones spreading Ebola in the community. So everything is just baffled, everything. At first they said we should not push them we should encourage them and now they said they are spreading the Ebola’.

M: Yes can you explain small about how they do spread it

R1: “Well more especially that sexual intercourse, like when your boy friend…..??...”.

M: My sister with this fine hair?

R1: “And they told us that survivor cannot get Ebola again but now...”

M: I want you to tell me small about how you treat Ebola survivors here?

R2: “Some people if they see that a person has had Ebola they took him and has come back we can still be afraid to go near him or her we think that maybe the person is not properly well yet”.

M: Yes my sister I want you to add small thing to that, how do you treat Ebola patient here?

3: “Actually when they just return people can be afraid of them because they do not understand how they are seen the person. We can be convinced when they say the person do not have Ebola again, it gives you encouragement to talk to the person and play with the person”.

M: Yes Ma?

R4: “Like us who are at the hot spot because if you have a house here and there and then the whole area around you is quarantined if you survive you survive if you die you die, so when we see them you go to them and talk to them”.

M: So have you heard of any new medicine to cure Ebola?

R5: “I have not seen any medicine except the one which they said it is for Malaria”.

M: So what do you thing about the medicine that you are about?

R1: “Well they said that there is the medicine is there but it is not yet time for them to start using it, so I think that they must I have tried it on animals or people but they think it is not for people”.

M: Yes my sister?

R2: “Just like what my sister said, well for me I have just heard that they are coming with vaccine but it is going to be in March but they said it is first going to be tested on the nurses when they take it and see that they are ok, then they will bring it to us now, because they are eating Ebola money”.

M: What do you think about the vaccine?

R2: “For me the decision which they took is fine for me when they said they are going to give it to nurses first and of cause Liberia have started taking it that why they eat to see how the medicine is going to affect them, the reaction of the medicines before they bring it here in Sierra Leone, that is what I understand”.

M: Yes my sister what do you think about that vaccine?

R3: “Well to me, they last gave people medicine which they said is a Malaria medicine so from then I swore that any treatment which comes from government I will be afraid to take it. They way that Malaria affected people, I am afraid especially this Ebola, for this Ebola treatment, I will protect myself and pray to God, I am afraid to take vaccine for that”.

M: Yes, Mama what about you?

R4: “Just like what my sister said, but I don’t have any reliable source, but anyway even this which they say they are going to give people took it and there was no problem

R5: ”Even myself I took it, I am waiting for the first person who is going to take it”.

M: Yes Mama? :.

R6: “To me personally, it has taken 10 years without me taking injection, then this Malaria medicine why was it killing people I think it was through the prescription because it was not thoroughly prescribed the people were not going for training just because influence or personal usage they call then to go and distribute medicine, so they will just go and dive you the medicine, mark your house and leave. Some time when I come home they just come and showed me medicine but I have taken Malaria medicine before I know how it worked me and now the medicine is working [people they won’t give them chance to recover, and there are some medicine if it does not fit you and you take it, it will work on you for nearly five days, but as it starts working on people they come and take them to the Treatment Centre”.

M: When you said it will work on you, what do you mean?

R6: “It makes you vomit, you just feel like a young pregnant woman that is how it happens to you, it weakens and then that shows you that it is one of the symptoms of Ebola so you see and they do not ask without confirmation, as they say there is a sick person they will just come and carry you so you see that is why people are afraid to take the treatment, I am going to tell lie on anybody, the tablet they are giving now there is nobody who can take the three, if the government is listening, it not everybody that knows the prescription”.

M: Yes my sister, have you heard of any new treatment for Ebola? Ok, so have you also heard about any new way to prevent Ebola?

R1: “No”.

M: What about you?

R2: “No”.

M: Yes my sister?

R3: “Not at all”

M: Yes Mama?

R4: “I have never heard, what they have told me is just what I know”.

M: Yes have you too heard about any new to prevent Ebola?

R5: “Yeah, because that is due to lack of communication from our people, even if it is there but we have not heard about it yet”.

M:Yes Mama?

R6: “I have not got any information; I don’t know any other way yet”.

M: What about the Marklate (=vaccine), have you heard about any Marklate (=vaccine) that will be coming for Ebola?

R1: “No”

R2: “No”.

M:Ok, yes Mama?

R3: “I have one of my relative who called me but he not in town here, he said they will be giving Marklate (=vaccine) and he told me that let me talk to people and those around me that let nobody takes the Marklate (=vaccine)”.

M: Did he explain to you the reason why he said people should not take the Marklate (=vaccine)?

R3: “Just because of this sick that is killing people so he pleaded with me not to take the Marklate (=vaccine). So it is now fixed in our minds that any Marklate (=vaccine) that they come with we will not take it”.

M: Yes Mama you too can you tell me about any Marklate (=vaccine) that is coming up?

R4: “Well I have heard about it but if it comes because people have different system there are some people think that if they take Ebola treatment they will die. There is somebody who if you give the person Malaria medicine it will react because maybe the person has another sick so when you give the person Malaria medicine it will react and then you give somebody a particular treatment is bad. So they should not come with medicine to treat Ebola”.

M: Yes Mama”.

R5: “No, I have never heard anything about Marklate (=vaccine)”.

M: Yes Mama have you heard about the Marklate (=vaccine)?

R6: “No”.

M: Yes?

R1: Yes I have about it but what our sister is say is very important. Just like I am telling you now, the government is so careless, even this medicine, it killed a lot of people because some people take three together some four and that is not the way. And let me tell you something what we are hearing, maybe you do not have malaria in your system and then you drink that medicine it is likely that it will kill you. So if it is a test that they did, maybe it will not happen they are just giving medicine whether you have the sick or not. It is not telling well”.

M: Ok, so what is the most common point of discussion when people gather to talk about this Ebola?

R2: “Well the rise and fall in the Ebola result, today it can be 12, tomorrow 1, next tomorrow, will be 30 and above, tomorrow 2. Theresult does not even go accordingly. Then I got it on the radio at one time that the Treatment Centre at (- - name of treatment centre location in the area - - ) only three patients are there, but the other day I heard that they are 26 or 24 I don’t know for the other Treatment Centres”.

M: So, what about the prevention and the treatment, is there anything that people talk about them?

R3: “Preventive measures are going on, people are preventing themselves, especially here in (- - name of interview community - -) here because of what we have under gone, we lost our loved once, friends and family member so now even if they tell you to go and wash in the Atlantic for you not to get Ebola (- - name of interview community - -) will do it”.

M: Ok, so the last thing I want to ask is, is there anything specific about Ebola which you think people need to know so that they can understand it better?

R4: “People need to understand that it is not just Ebola that has come to this country. From the time when I have sense, this is this only year I heard that there is no Cholera, choleras is no more, any sick is Ebola. Stomach ache is Ebola, head ache is Ebola, and Malaria is Ebola”.

M: So Mama add small thing to that?

R5: “Well all that I have to say is that because thing has been affecting us, is this only Ebola because during delivery if bleeding becomes worst the nurses will run away. It is true that we should prevent ourselves, but it also a risk, but let’s just take this risk, the risk we are taking is to end Ebola for us to have freedom and peace”.

M: Ok, yes Mama, please tell me, is there anything people need to understand about this Ebola?

R6: “Well people should understand that the sick is real and we don’t have to relent until they announce that the country is Ebola free”.

M: Yes Mama what do you think people need to understand?

R1: “According to what they say if your headache they say is Ebola”.

M: What do people need to understand about this Ebola?

R2: “There are compound that was quarantined or maybe somebody died there of Ebola they would like you to go and sympathise with them at the burial and which of cause it is wrong because some people when you did not go there they turn you to their enemy because when the person was bereaved you did not go to visit them. Really it is not supposed to be so because maybe the virus is at the [place and when you go there you might get and spread it to your family at home. So really people have to stop that”.

M: Can you suggest any way how we can get this message to them?

R2: “Well they can tell them in any way because those who use that megaphone they can meet them and tell them my people do you have anybody here who is Ebola. One if somebody did not go to visit you do not feel bad because maybe the virus is scattered at the house so if the person did not come to visit you do not feel bad about it after Ebola we can come back together and make peace

“.

M: I like that. Yes Mama, you can add small thing to that. What do you think specifically that people need understand about this Ebola>

R3: “Well they need to tell people about the thing that is killing people and according to my own understanding when somebody dies, you should not touch the body or wash it, that is what that is killing people. Any who washes dead body has had less chances of survival”.

M: So which way can you suggest for us to take this message to the people so that they can get it?

R4: “You have to find people who have the understanding and who knows how to pass the message so that they can explain to people from house to house in a way they can understand if they do that you can see the way they will eradicate the virus in Africa”.

M: Ok, Mummy you too can you add something to that?

R5: “Just like what they are saying, when the person is alive and you have the virus the possibility for the person to survive is slim according to we are hearing from medical people when the person is dead, the possibility is open. So we just have to be careful”.

M: So which good way do you think we can tell people about this good things which you have said?

R6: “If you are sympathising with a person call the person on the phone and sympathise with the person or if you are not able to get communication leave the person after the 21 days if you see that there is nobody in the compound that is affected, you can go and visit that person and sympathise with the person”.

M: Is there any other person who wants to say anything concerning Ebola?

R1: “Concerning this Ebola?”

M: Yes, what I mean is there anything specific that people should understand about Ebola?

R2: “Is just that Ebola is real, all of us has to be careful, until we zero for forty one days”.

M: Ok, I thank you all very much.

**ADDITIONAL PART OF INTERVIEW, OBTAINED BY COLLECTOR 5 AFTER CONSENT IN PERSON with two (2) of the original participants, March 2015:**

M: In our last interviewed, you spoke of a barrier between you and the quarantined people, even when you are passing by and greets, they will not answer, and this has created enmity between the quarantined people and non- quarantined people, so I may like to know what led to the barriers and the enmity between the quarantined people and the non-quarantined people and why are they not happy with the situation, and why the people do not greet the quarantined people and even when they are passing by, they gave comments?

R1: “Ok, the enmity that exist between us, because it has not happen with us here, so the enmity that exist between them is that, they don’t have dealings with them, because they are thinking when they had dealings together, the sickness will transfer to them, they do not talked to each other, everybody minds his or her own business, so the sickness will not transfer to each other”.

M: So that came about enmity, because one person do not greet to other thinking that when they greet them, the sickness will transfer?

R1: “Yes, this a bad sickness and it transfers easily, even when they had finished the quarantined period, when they saw them in the street, they will skip off from, thinking that when they greet and touch, the sickness will transfer and the even the people in the quarantined home are ashamed and hide, for people not to point hands on them, that this the person that had that sickness”.

M: If you were an Ebola survivor, which problems you think, you will experience, when you returned to your community?

R1: “The problem you will experienced is isolation, people will not be happy to be with you and you will not happy within your inner self, at time you may want to eat together, there will that difference among you and others and that playing with each other will stopped”.

M: How this community treats Ebola survivors when the returned?

R1: “Just as that woman said, they will think that when you had got the Ebola sick, when they mingled with you, they will got Ebola sick, because a sick person is a sick person, maybe the person is not properly cured if I touched him or her I will contact the sick”.

M: Do you think, this thing is happening in the other communities?

R1: “Yes of course”.

M: Ok, Some people do not believe the sickness exists, it took a long time before they adjust to it, these people do not believe because they said, people created the sickness, I want to know, who are people that created this sickness and why they said the sickness was created?

R1: “At first when Ebola broke out I denied that the Ebola sick do not exist but presently I am not denying again, because I had saw with the sickness had done, the way it is killing people, and the way it is treating people, although there are other people that are still denying, I knew Sierra Leoneans will are very stubborn, just like how you said, plenty people are saying they do not believe the sickness exist, they knew who they are”

M: But why do you think Ebola do not exists, what really happened which made you to deny that the sickness do not exist?

R1: “Someone died here, they said it is Ebola, before Ebola broke out people got sick and die and the symptoms for Ebola are the similar to malaria, that why I was denying that Ebola do not exist, but I had believed that Ebola is real and exists”.

M: So what do you think, people were saying Ebola do not exists and it was created?

R1: “Because World Health organisation (WHO) showed the estimated amount of people that are going to die by this Ebola sick so everyone were wondering why they should say so, so the people said this is a manmade disease if not they should had not estimated the number of deaths, that was the reason. The people thoughts this is a chemical, but when it begun killing people, if even it is a manmade disease everyone had got the believed now that Ebola is real and exist because if it entered into a family if you do not the right thing, it will wipe-out the whole family”.

M: You also said, some people were secretly given treatment to sick people at home, so what was the type of treatment were they given to the sick people at home, and why they do not go to the treatment centre or call 117?

R1: Well really we had not gone through that, but people are doing that thinking the sick patient had malaria, some other people thinks it is cholera because they had the similar signs and signs and symptoms”.

R2: “The reason why some people were treating their sick people at home, because it has never happened to us, I thank for that, some people were saying the sickness is malaria or cholera, there is no differences between the signs and symptoms of malaria or cholera to that of Ebola, this was the reason the people were not worry and they were treating their sick patient at home having the fear that if they go to the hospital one or two things will happened, fearing that they will kill their relatives or given them chemical. So people think”.

M: You also said, when they go and collect sick patients at their homes, when they take them to the hospital, they killed them, who are they referring to and why do they think this?

R1: “Some people say when you are taken to the hospital, the chemical alone they are using kills you, and even the environment they come and collect people, the chemical is really harmful, when it is sprayed, it disturbed plenty people, even those that are not sick and that is the reason some people thinks that when they take you to the treatment centre, they will inject you and you will die, even though it has not happened to me but so I heard”.

R2: “Just like how my sister said, first they did not know how to mixed the chlorine, because you may see some people walked strongly into the ambulance, but since there is no air space inside the ambulance, they will spray enough chlorine and people were strongly with the idea that chlorine killed the people, because at one time they came to collect a dead body around our area, when they opened the ambulance though we were at a distance, we smell the chlorine. The way they were using it, ether they had idea or not, it was too much, it was too concentrated”.

M: You said when they come to collect dead bodies, they were given them money and also when people go to the treatment and come, they are afraid of that person not to give them the sickness, so who are this people that were receiving this money and who are the people that are given the money and why do you think they were given the people to you?

R1: “In the area of the money, I do not have any idea, what they were saying, when they come and collect a body if they person had died, if you need this dead body after three days you will go and give them something”.

M: What is the something?

R1: “Money”.

M: How much?

R1: Well I heard some people saying five hundred thousand (about 113 USD 11mar15)”.

M: Leones or dollars?

R1: Five hundred thousand Leones, they will give them and they will hand over the dead body to them for them to bury”.

M: Who are they giving the money?

R1: “where they will take the body for observations”.

M: Where?

R1: I don’t have idea”.

M: Another person said the government is careless and not serious, I want you to tell me why are the government not serious and what they are careless about?

R1: “You the government the as head, anything good or bad that happens, they will cast blames on them, because they are the head, people were saying, when the sickness broke out in Kailahun, they should had put the whole Kailahun District under siege but since we do not knew the sickness and we don’t had the any experience of this kind sick even though it has been in another countries or the way it operates but we did not know until after it killed a lot of people, that was the time everyone got the awareness”.

R2: “Just like how that woman said, when this sick started in Kailahun, if they should have stopped it, it would have been better, it should have not even come here, the put some implementation in place, taxi’s taken two passengers at back, and closes the roads to the rural areas, and on Saturdays all markets or businesses should closed after twelve o’ clock and they stopped the choking of people in vehicles, it was really helping us, and even made the daily Ebola result went to two cases per day, but when they withdrew all this laws, we had started getting cases that is more two”.

M: Some people said, they were injecting people on their toes, why were they do that and who were people doing that?

R1: “I do not have idea on that”.

R2: “Well I heard but I do not see”.

M: They were injecting them on their foot?

R2: “Yes, when the Ebola broke out newly, some were saying, the toes and some says the span cord “.

M: why do you think they were doing this?

R2: “I don’t have idea on that”.

M: Ok, you just heard by rumour?

R2: “Yes”.

M: Another person said, people are eating Ebola’s money, who are the people eating this money?

R2: “They always say people ate Ebola’s Money”.

M: Who are these people?

R2: “They said the big ones”.

M: Who are these big ones?

R2: “The heads, those that are in charge”.

M: heads of hospital or the people in charge of the Ebola?

R2: “The people that are in charge of, I don’t their names”.

M: Is it the Emergency Operation Centre (EOC)?

R2: “We just heard rumour”.

R1: “What I heard, they said the government”.

M: The government officials?

R1: “Yes, and the heads of the centres, nurses, and doctors, all of them ate the money”.

M: So why are they eating this money?

R1: “Because they said every day the number of Ebola cases are increasing, so for this reason, they said it is money finding”.

M: Ok, in this community, have you ever heard of any secret burial or secret washing of dead bodies, can you tell me why were they doing it, and what kind of people were doing it?

R1: “Since Ebola broke out, I had never heard of that in this community, because in I heard it I will call to help the person and also help myself, because if a person it infected, it will scattered all over”.

R1: I did not hear of any secret burial in this community”.

M: Ok, thank you
