# Supplementary material for: Development of a set of community-informed Ebola messages for Sierra Leone
Source: PLoS Negl Trop Dis. 2017 Aug 7;11(8):e0005742. doi: 10.1371/journal.pntd.0005742 (PMC5560759; doi:10.1371/journal.pntd.0005742)
Supplement: S1 Appendix — (ZIP) [file pntd.0005742.s001.zip › Ebola messages - FGD and interview transcripts/R2HC Ebola Fieldwork 1/R2HC Ebola F1 FGD-FEYOU-Urban1 V3 CORR.docx]

| CODE | **R2HC Ebola F1 FGD-FEYOU-Urban1 V3 CORR (urban focus group discussion)**  **V2 – 10^th^ March 2015 – ADD PROBE**  **V3 – 11^th^ March 2015 – correction personal data participants** |
| --- | --- |
| DATE | February 2015 |
| DURATION (minutes) | 44 |
| Collector nr | 2 |
| LANGUAGE INTERVIEW | Krio |
| **TYPE FGD** | Younger Females |

**PERSONAL DATA PARTICIPANTS**

| Nr | Sex  (*F/ M*) | Age  (*in years*) | Education Level (*e.g. none, Primary, secondary, tertiary*) | Language (*e.g. Mende, Temne, Krio)* | Religion | Job / Employment (*how they earn their living e.g. farmer, teacher, trader*) | Role in community  (*e.g. youth leader*)  ANONYMIZED, ONLY AREA OF ROLE INDICATED |
| --- | --- | --- | --- | --- | --- | --- | --- |
| 1 | F | 18 | Secondary | Krio | Christian | Student | None |
| 2 | F | 15 | Secondary | Krio | Christian | Student | None |
| 3 | F | 21 | Tertiary | Mende/Krio | Christian | Trader | None |
| 4 | F | 22 | Secondary | Mende/Krio | Christian | Trader | None |
| 5 | F | 16 | Secondary | Krio | Christian | Trader | None |
| 6 | F | 14 | Secondary | Krio | Christian | Student | None |

**TRANSCRIPT: (M = Moderator, R= respondent, R1= first person responding to a question, DOES NOT correspond to numbering used in Personal Data!)**

M: How has Ebola affected this community?

R1: “How has this Ebola affected this community is that it made us lost our brothers and sisters it has made us lonely, we have lost our parents and loved ones, like for me I have lost my daddy”.

M: Through Ebola?

R1: “Yes, so it has made us lost most of our brothers and sister so some of us it has put us to zero, so that is how this Ebola has affected us”.

M: Yes my sister with the black, do you want to add anything to that?

R2: “Well to me this Ebola has affected this community when we can no longer gather together again like for example where [we] used to go and fetch water they have banned us not to go there again to fetch water because of this Ebola they said they do not want us to carry Ebola there. So it has affected us greatly because we were straining for water here, so where we used to go and get water the man who owns the place said we should not go there again because of the Ebola”.

M: Yes my sister, how has it affected this community?

R3: “Well Ebola has affected this community greatly, it has restricted our movement like me I used to go and find my Aunties once in a while when I am ?ideal? I go to them on holiday but now I do not go because I am afraid I don’t know at their home whether a person might have carried the sickness then I will not go there to bring it to our house. Then it has made me lost trust in many people. Like for some of my Aunties who come around I will allow anybody to touch me; even me I don’t touch anybody because I don’t want to get this Ebola”.

M: Yes my own sister over there?

R4: “Well just like what my sister said the Ebola has really affected us, it has restricted our movement. Like for the water side which she said, that has made us to go very, very far us and that has made us to strain, it has made us not to make friends again, we do not meet with friends, we do not joke with. It can make you be afraid to touch your companion. You can really know that this person does not have but within yourself you will trust the person so you don’t just have to touch the person. So it has really affected us, it has made us not to move freely we are afraid to touch it has made everybody to sit at your house now”.

M: Yes”.

R5: “Actually Ebola has affected our communities, you can see families die, we the young ones we are not listening, we the young ones they are telling us not to touch but still people are going ahead to touch. People are dying so it has even affected some families because two to three people have died and we cannot even be able to go and say sympathy and condolence for burial so Ebola has affected us too much that I cannot explain all”.

M; Yes my sister?

R6: “It has affected us so much because we used to pass in certain areas to go to market but now we cannot because the area has been quarantine”.

M: Ok, I thank you. Have ever seen anybody who has Ebola?

R7: “No”.

M: What about you, have you heard about somebody who has Ebola?

R8: “I have never seen somebody who has Ebola”.

M: Why do you think this Ebola has spread in Sierra Leone?

R1: “It has spread because one, when a person has Ebola even if the person is your person (=family, close to you) you should call 117; you should not touch the person to come and take the person to the hospital the earlier the better. If you fail to do that and you go and touch the person. Then they also said that when a person dies you should not wash the body once it is suspected Ebola case you should not wash it or touch it but some people are still not listening, they are still washing and touching people that is why Ebola is now spreading it is not ending”.

M: Yes my sister with the black?

R2: “Like my sister has talked, the reason why this Ebola has spread is that some people do not believe that Ebola is real, some men are taking it as a political reason. But truly Ebola is real. As my colleague have said that when the worker advise that you should not wash dead body, you should not touch your companion you should not be close, something like that but still some people do not believe that Ebola is real’.

M: That is why thing is spreading because people do not believe that Ebola is real?

R2: “Do not believe that Ebola is real”.

M: Yes my sister in the corner?

R3: “Just like what sister have just said when they are telling us to wash our hands, we should not touch, you should not bury the dead but are still washing dead body so that is why Ebola is spreading”.

M: Yes my sister in this corner?

R4: “Some people when their person is sick they are now afraid to call 117 that why is Ebola is spreading”.

M: Yes?

R5: “According to my sister, why this Ebola is spreading actually is because they have told us ‘ABC’- Avoid Body Contact but some people still even when you are trying to push they will still come to touch you. Some people when they are sick they say it is not Ebola even if they go to quarantine them they fought the police. So because they do not believe that is Ebola is real they can get the virus and they go about touching people that is how the virus spread”.

M: Yes my own sister?

R6: “Well in addition to what they have said, they all have right, then one of the main precautionary thing which people are not putting into practice, because if they say don’t touch and you touch, for instance that Nurse, they quarantined one place down here the Nurse went and washed a patient who died of Ebola, that was they took her and her daughter they took them as suspect case and they quarantined the whole compound. They do not agree to put the precautionary measures in place, they do not wash their hands, they do touch, wherever they meet the are glad, the people are not putting into practice what the medical personnel have asked them to do and all that is coming about because they do not believe that the sickness is real because at first if they had believed that the sickness is real that sickness would not have spread to this extent”.

M: This other question says, do you have any local term to call Ebola?

R7: “No”.

M: You haven’t heard any name?

R8: “We have not heard any”.

M: You have not heard any name?

Rs: “No”.

M: Some people think that Ebola is not real, do you have such people in this community?

1: “They cannot be there, because they have seen what has happened in the community”.

R2: “They are there”.

M: Who do not believe that Ebola is real?

R2: “They are there, some people just take it to be politics; how Ebola is not real, look is just about money business, this or that, and we have a lot of talk coming through this Ebola”.

M: Some Say it is because of politics that Ebola has not ended?

R2: “Yes”.

M: Ok, can you give me an example Ebola messages that you have heard?

R3: “I have heard a lot of Ebola messages”.

M: Like which one have you heard?

R3: “Avoid body contact”.

M: Which one again?

R3: “No”.

M: What do you think about that message? The one which they said avoid body contact, do you think that is a fine message?

R3: “Yes it is fine message”.

M: Which other messages have you been hearing?

R4: “They have said that we should not wrestle, even when you go out for a walk say about one mile when you come back you wash your hand with soap and water then you should not go to your neighbour’s house because you do not know whether somebody has it or you who is paying visit you might have carried with you”.

M: My sister with the black?

R5: “Well the Ebola message that I have heard is like that which they Say the cases are reducing but still the cases are going on and on, even the deaths are just increasing daily. That is one message I have heard”.

M: Yes my sister with the pink?

R6: “I have heard that they have come with some foreign Health Workers to fight against this Ebola they have opened a lot of lab (=laboratories) and have brought a lot of people to help with this Ebola”.

M: If a person is sick of Ebola, which message can you give the person to encourage that person to go to hospital?

R7: “Well I will talk to the person that this sickness you are not the only one at risk if you say you are not going to hospital, that if the person stays at home your whole life and the community as a whole will be at risk because if anybody touches you he or she is going to come in contact with the body. If you remain to lying down there and the sick gets worst then you die, then your family will lose you but the is possibility that if you go to hospital the earlier the better it will be well they will be able to cure you and you get well and come back to meet your loved ones so instead of you put the whole family and for you to save your loved ones you have to go hospital quietly so that they can cure you and you will come back to be with your loved ones peacefully”.

M: You too want to say something, about the messages. Which message can you give?

R8: “Well, just like my sister said I will not go closer to the person; I will give her space and say to her my sister, I beg you let this sickness do not overcome you, go to hospital; do not allow motor car to come and take you because we are hearing that the motor car in which you are going to sit when they spray inside that one can kill. So I will advise her to walk by herself to go to hospital and when she goes to hospital then she will come back home to come and share love with her family. But you stay at home you will not know that it is the sickness, you decide to go give her ORS and then you exchange and when you exchange then you are going to get it. If she takes it fine if doesn’t take it the earlier the better a person will go and call 117, and that is where enmity starts and when you advise her you will hear from her whether she will like you as a friend or whatever”.

M: Yes, my Sister, what messages can you gives somebody to encourage the person family to carry her to hospital? Who again wants to add to that?

R1: “When once you have the sickness some people will be pushing you, where ever you go they will say don’t come here and the person too will be afraid to go hospital, but you should encourage the person you should not neglect the person that my sister go to hospital so when you encourage her the earlier the better that this sickness will not spread in the community where you are when you would have gone to the hospital you will get healing. You should encourage her; you should not neglect her in your community but you should encourage her and tell her to go to hospital when she gets to the hospital she will get healing”.

M: Yes who wants to add to that? Which message can you give somebody to encourage that person to go to hospital?

R: “I will tell the person that he or she is not the first to have the sick I will tell the person that the earlier he or she goes to hospital the better his or her chances of survival hat is the talk will tell the person”.

M: Yes, you too want to add to that?

R 2: “Yes, like what my sister just said, because like when some people gets this sick he or she just feels that it is the end of his or her life but what I believe is that once you are not destined to die by the sickness you will not die so if I see anybody who is sick of this Ebola I will talk to the person and encourage him or her to go for early treatment so that it will help you to survive”.

M: Alright, ok, em, if I want to send a message, which channel is the best to send Ebola message?

R3: “Through television”.

M: Who again?

R4: “Radio Station”.

M: Radio Station, who again?

(*Respondents arguing that some village do not have Television and radio stations*)

M: So which channel radio?

R5: “Yes, some villages do not have Television”.

M: For instance, in this community if somebody suspects that he or she has Ebola what do you think the person will do first? Will he or she goes to a ‘meresin man’ (=traditional healer) for cure or will go to the hospital to be cured?

Rs: “To the hospital”.

M: Why do you think so?

R6: “I think so because they have told you about Ebola and then you have you have seen signs and symptoms if you like your life and you like your family, you will tell your child not to come closer to you if you don’t want your child to know then you can go to hospital and then they can call your family and tell them that so, so person is admitted at the (- - name of a treatment centre - -) or any other hospital so that at that time you can get better treatment then if you go to the ‘meresin man’ (herbalist, traditional healer), herbalist has no hands in this Ebola”.

M: Yes, you too what add to that? Where do you think the person will go first to a ‘meresin man’ or to the Holding Centre or Treatment Centre?

R7: “Like in some cases some people who do not believe they are afraid to go to hospital thinking that they are going to die there. Some will think that they should go to the ‘meresin man’ (=Traditional Healer) thinking the ‘meresin man will not kill them but will help them to get well so some people will not go to hospital. But in some cases because when people will go to hospital believing that when they go to hospital they will not kill them they cure them, but some people will go to the ‘meresin man thinking that if they go to hospital they will die so they prefer to go to the ‘meresin man’ because the ‘meresin man’ knows their problem that is what I think”.

M: Yes?

R8: “Like what my sister just said if you have seen the signs and symptoms and you really know that it is Ebola you should go to the hospital instead of going to the ‘meresin man’ (=traditional healer) should go to hospital so that can be able to protect your family”.

M: You too want to add to what she has said?

R1: “No”.

M: This other question here is about the Ambulance. I want to know if you have been hearing good or bad thing about this Ambulance.

R2: “I have not heard any bad thing about them”.

M: So which good thing have you been hearing about them?

R2: “When they come to take dead bodies if a person who is an Ebola patient they come and take it and there are some men when they come to take a dead body they do not allow anybody to go near to the motor car because some people when they go near they go there to thief. Like one time when one died here and when the Ebola Ambulance came to take the body they did not allow those people who were passing around they were afraid and some people talk a lot”.

M: Yes, you too want to add something about the Ambulance Service?

R3: “Well I have heard I have heard good thing and bad thing about the Ambulance.”.

M: I want to hear all the good things that you have heard.

R3: “Well the good thing is when they call the 117 Line the Ambulance will come immediately and assist the people but in some areas when they call the 117 they will not come there they will even make the attempt they have no remorse to come. That is the bad thing I have heard about this Ambulance. They will call then and call them they will not come and they have said people should not wash dead body, they should not touch dead body but when they call them they won’t come they do not show that remorse. That is the bad thing I have heard about them”.

M: Yes, have you too heard any good or bad thing about the Ambulance?

R4: “The same thing like my sister has said, when somebody dies of Ebola they will call 117 they call and call...they will not come on time, they won’t do it. That was what they did to one boy until the boy died. So if like a mother’s child dies and then the mother touches her child because of feeling for her child because they have not come to collect the child she too will contact the sick”.

M: So you are they always delay en?

R4: “Yes”.

M: You too want to add to that?

R5: “Yes, I have the good I have the bad. The good, I have heard that when some people call the Ambulance it will come immediately and will assist them but the bad is some people are saying that when the Ambulance comes where the patient is supposed to sit is where they spray, some people in the Ambulance some people because they have power they will try to kill you inside the Ambulance. I have heard much about the Ambulance which I cannot understand”.

M: What do you mean?

R5: “The things I have heard is that, some times last week a friend asked me whether I have heard the news, then I said which news and then she asked me if I know that driver put ‘plasas’ (uncooked leaves used for food) into the Ambulance and the Ambulance is not for any other thing but for someone who is sick, someone who has died, or a person who has Ebola so when some people die inside the Ambulance we black men take it that it is the chlorine that has killed the person so I want to get much idea about this chlorine because some people are saying that when you go by yourself to the hospital you can overcome it but when you go inside the Ambulance they will kill you”.

M: You too want to add something there?

R6: “Mhmm, yes. The good side about the Ambulance is that at times when they call them if it is a sick case the earlier they come they would be able to save that life but at times the dead body will be lying down even if the call them, people can even go over air and say a dead body has been laying in a house for over three days and they have called the Ambulance and nobody has shown up. They would call and they won’t come they won’t even attempt to come, they cannot even answer. At times that is what makes the sickness spread. Sometime some people would say since they have not come for the person the family will say let us wash it and hide it and bury it quietly and that is happening, people are washing body secretly undercover apart from the delay on the part of the Ambulance”.

M: Yes, you want to say something?

R7: “We have heard that case here before. The child died and they call 117 and they did not come, it was later in the evening that they came to collect it”.

R8: “So added to that I understand that sometimes the Ambulance transport people, for instance when they are going if they reach a check point they put the emergency on (=siren/ light, alarms on the ambulance) as there is a patient on board or there is a dead body there and, that is the bad side about them”.

M: What about the Holding Centre and the Treatment Centre, have you heard anything good or bad about them?

R1: “Me the bad thing I have heard about those Centres they said when the patients go there they do not treat them well they treat them just like slaves, like prisoners they do not give them water, good food they do not treat the patients like, those who come there came and explain to us, they said when they go to the Holding Centre they said when they are there they do not treat them well. The Medical people do not treat them well and this sick you should not neglect the patient, you have to encourage the patient when you encourage the patient you help to improve his or her life. So the person cannot say because he or she has Ebola the family has neglected him or her, the Health Workers neglect him or her then the family too neglected him or her. They said when they go there they do not treat them fine, they treat them as if they are slaves, they do not give them good food”.

M: Have you heard any good thing about them?

R2: “The bad thing I have heard about them is that some say when they go with them they have one injection which they give to them so that they can die quick. Then, just like my sister said, they do not treat them fine they are treating them badly that is what I heard”.

M: Has any heard anything good or bad about them? You too want to add something?

R3: “Yes, like I understand that when a patient goes there when they mix ORS and give it to that patient, the person will be lying there for the whole day, they said the patient do not get any encouragement and some die of broken heart”.

M: Yes my Sister, have heard anything good or bad about the Treatment Centre or the Holding Centre?

R4: “Most of the people who come from there said when they are there they do not even give them food or water that has made a lot of people to die of this Ebola”.

R5: “As for me where I was 24 hours they give them food but the only thing when you go there very week they have one injection which they give you if you are not strong enough you will die. But the ones I have seen at the Holding Centre, that (- - name of treatment centre - -) Centre they give them food 24 hours even the orphanage they have one man there who takes care of them and those Nurses too who take care of them”.

M: Are they affected by Ebola?

R5: “No their parents died of Ebola but they care of them, they feed them and you see them playing around. I am not saying about other Centre, but (- - name of treatment centre - -) they do help. I have been close to them I have lived with them. I can be in my compound and I can see inside the (-acronym of centre --). So I know most or majority there”.

M: You too want to add to that?

R6: “Yes, I have heard a lot of good things about them because if they are not doing well there nobody will go there and they discharge that person alive. They feed them, they give them medicine. Just like what this one said the medicine, the injection which they give to them when you are week is too powerful when you are week if you are not strong enough you cannot make it up. Then at times by the time they get there they have already given up. Most times they always give up on themselves. The Nurses too are trying because it is not easy for you to risk your life to leave your home to come and stay there to treat people when you do not know maybe the person has it or not. They are just risking their lives for the health of those people get well”.

M: Emm, this Burial Team, Have you heard anything good or bad about the Burial Team?

R7: “I have heard good thing and I have heard bad thing”.

M: I want to hear all that you have heard.

R: “The good thing is some people even [give] them money to bury their person properly because even if where we are we see how the Burial Team dresses, they will say please when you are going to bury that person please hold him fine. Like when they come for the dead body they do not just fling it like that they will pick it up quietly and put it into the grave. The bad thing which I have heard about them is that they said when they are taking the bodies from the Centres, and I have seen it myself they swing it thrice and fling it they do not have any regard, no respect for...”.

M: Inside the grave?

R7: “No inside the motor car, when they come to collect it, then inside the motor car when they are going, they will just fling it the way they like and that does not tell well, when a person is dead you have to give the person last respect so he or she can get...”.

M: Who again wants to add to that?

R8: “Well for me I have heard a lot of good things about them. If it is not for their sake bodies will get rotten in the house like this sickness would have been worse than this. But the bad thing which I have heard about them is that when they go to bury, you who have money they will take good care of the body because you are going to give them something and tell them to take good care of the body that the body is a special body, but if you do not have money, oh no, they will just fling you, they will bury you just like an outcast, like somebody who has no owner because you do not have money your people do not have money to bribe them. That I have heard but I have [not] seen”.

M: Who again wants to add to that? Yes?

R1: “Well I want to add something to that about these men. Actually they are trying for us, you see some people when they come they rush because they like Sierra Leone people when they come they rush to take the body to go bury it. But some people even if you call persistently, I don’t know, but is like the Burial Team, there is the good one there is the bad one. You will call them persistently more especially where I was (- - name of community - -), the last time it took up to one week even though they are close to the Centre. So the mother walks to the Centre and ask them if they want the body to get rotten at home or they want all of them to get the disease. They left a whole human being inside a room and pallor just for them to avoid the sick. Not until when the mother went and lodge complaint at the Centre before they came and took the body except they had to spray the place before the people were able to settle”.

M: Who again want to add? What about the 117 Line, have you heard any good or bad thing about them?

R2: “I have heard the good and the bad.”

M: I want to hear them.

R2: “Some of the good is that when you call they come quickly to come and take the body but some of them when you call it takes long some time people call from the morning, at times also they murk the people when they call they give the people the wrong address when they go there they will not meet any sick person or no dead body, nothing no signs and symptoms of Ebola some just call, I think it is because it is free. Well at times some can call to show concern. Some times when they call 117 they do not try to come because when serious cases come up even if they call them they will not come they will just think they want to disturb them so that is why even if they call them they will delay to come. Sometimes they come quick”.

M: Yes, you too want to add to that?

R3: “Yes, I have heard good thing about them because if they had not made themselves available like the sick would have been worse than this. Because of the transportation means they use to come that alone has helped greatly in the communities in the country as whole even though at times when they call them to come is a trouble it takes them long before they come but even that is not their fault just like this said people can use it to make fun, they will call the 117 to come to a location when they come they will see nothing there. That alone is a reason for them not to come on time when you call them at times they too can delay to come because they do not take them seriously. Even if the person called you for nothing it is their duty because they will be coming to save life. That is what they should get at the back of their mind that they are coming to save life and that is what they [are] paid for”.

M: Who again wants to add to that?

R4: “So like my sister said, at times people can just call especially those boys they can just call them for unnecessary reason without any sick person or dead body so it can reach a time when they call them they will delay to come that is why some times they begin to abuse them”.

M: So the hospitals that were there before and the ones they have built now, have you heard any good thing about them?

R5: “Well the bad thing I have heard about them is that when patients go to the hospital the nurses there do not treat them seriously because they take it that when they attend to them they too will get the sickness. So the Doctors too are afraid to treat the patients. In other cases the Doctors too can show that remorse to treat the patients but in some other cases they do not treat them, well they take it that if they treat the patient they too will get the sick and they do not want to get it so they leave the patient to die at the hospital. That is want I have heard about them”.

M: Do you want to add to that?

R6: “I have heard a lot of good things about them as well as the bad ones. Some of them when they come and treat and that is their job, they vowed to do that. Then at times some of them when they call them they do not treat, Doctors and Nurses run away from patients and leave them their and maybe the treat which he or she could have given to the patient will go a long way to save the patient but because he or she runs away, trying to save him or herself they would have killed the patient already. So that is the bad aspect I have heard about, Doctors can even deny that they are not Doctors, the Nurse too will say Doctor you have to go there, Doctor too will tell the Nurses no, you have to go there, they keep denying themselves”.

M: Do you want to add to that?

R7: “Yes well actually, you see I think (- - name of a big hospital - -), yes at the (- - name of big hospital - - ), well when this Ebola started new we used to hear bad things about them but even we the mortal men have the problem. Just a small incident happened but I don’t think, they said she has Ebola she went and treat an Ebola patient but she too has caught the virus instead of telling her friends not to touch her because of so, so, so thing, she went to her family and they ran away and went to (- - one end of the interview district- -) when she went to (- - one end of the interview district - -) they were able to trace them until they caught them by the time they want to think about it the lady died. So we should not only put the blame on the Doctors, we ourselves have the problem, some people are very stubborn because even when we have seen it killing people. Somebody was sick close to us by the time we went to the hospital the Doctor was not able to do anything. So if the patient is able to go quickly and if the Doctor is a Godly person and knows that he has a person he will be able to support you”.

M: This other question says, have you ever seen somebody with Ebola in this community?

R8: “Mhmm, like this quarantined house the woman who they said went and treated somebody and they got infected together with her daughter but they survived and came back yesterday”.

M: So when they came back how you did treat and reacted towards them?

R8: “Like when they said we should not go far away from them, when they came people went and welcome them, talk to them”.

M: So like everybody welcomed them in the community.

Rs: “No”.

M: Have you heard any new treatment for Ebola?

R1: Me I have heard that a medicine has come but I don’t know whether they have started but is just the same thing, the ORS, Malaria treatment”.

M: Who again want to add something? Have you heard about any new treatment for Ebola?

R2: “Like what they say that even Bitter Cola (*note: Bitter Cola - officially “Garcinia Kola” is a tree of which the nuts are chewed /used for medicinal/stimulating purposes* ) can cure Ebola’.

*(Respondents talking in chorus)*

R3: “I have not heard about any new one yet”.

M: Have you heard any new way to prevent Ebola?

Rs: “Except this wash hand only, wash hand, wash hand, Avoid Body Contact, Avoid Peoples Compound is just the same”.

M: Have you heard of any vaccine or marklate (=vaccine) for Ebola?

R4: “No, at this later part, no”.

M: In this community, what do people say about Ebola? What are their concerns?

R5: “Some people can discuss that Ebola is not real and at the Ataya Base (*note: preferred meeting place of young people, where they drink a strong highly sugared green tea that comes in a box labelled “Gunpowder.”, but is also called Ataya*) where they usually sit some do bet that Ebola is not real they just want to make money, all kinds of talk, words which people are not supposed to use they use it and some people even use word that can affect them later, their family can catch up with the sick and come and spread it in the family. You can never believe that this Ebola is real until it affects you and when you get affected you just have to die”.

M: What do you want to add to that?

R6: “Like some boys who are idle they won’t sit down and talk anything serious about this sickness. Some say the government is finding money, some talk the good side, some talk the bad side and some say the negative side everybody is saying his own they go into bitter argument. Some say this Ebola is not real that the government only wants to make money, is just money finding. Some who have seen and experienced it will argue that Ebola is real but some who have seen and do not believe say government is just doing money finding”.

M: Do you want to add to that?

R7: Like some men around this area when they drunk they stand out there and say bitter cola is a medicine for Ebola, they nearly fight over it”.

M: Is there anything specific about Ebola that people need to know for clarification purpose?

R8: “Except if for those who do not understand you try to bring it to the lowest level for him or her to understand”.

M: Is there not anything specific about Ebola that people need to know about?

R8: “Most of the calendars (=posters), they way how the symptoms are portrayed, I have never witnessed it the way we see it”.

R1: “I have seen it’.

M: So like the symptoms that they put there you have never seen it, isn’t it?

R1: “No”.

M: So like you want to know more about the signs and symptoms of Ebola, isn’t it?

Rs: “Yes”.

M: Yes, you too want to add to that?

R1: “Yes, I have seen it but like I did not go close to the person, this lady ran away and came there I don’t know where he came from so in the morning I saw the lady but like the rashes on her body so like I had to meet one of my cousins and asked her what was wrong with the lady so she said this woman we need to meet Councillor..”.

M: So like you can really see the signs and symptoms on her?

R1: “Yes, she and then she went and found a sweater trying to cover herself, she too I don’t know whether she was trying to hide herself, the same night, she came in the evening and I saw her and she died at that night so they had to quarantine that house at (- - another community in same interview district - -)”.

M: Is there anything specific about Ebola that you want to know for better understanding?

R2: “Like the way people are using this chlorine, because more people are stressing on that because some if they move from here to there they will wash their hands as he or she finishes and go to another place he or she will wash his or her hands without the understanding as to how to use the chlorine’.

M: Like the need to know the mixture because you cannot use chlorine just like that and it has to be the correct mixture.

R2: “Even if it is not the correct mixture, wherever they go they wash their hands and this chlorine is a bit bad. That is why those offices now do not use chlorine they use soap. We want to know the correct mixture then they said this chlorine can cause skin cancer, how true is it, whether it is true or it is a lie because that has made people to be afraid to wash their hands with chlorine. Some people no sooner he or she smells chlorine he will find somewhere to wash his or her hands with empty water as long as he or she has smelt the chlorine saying because they said it can cause cancer so we really want to know if it is true or it is a lie so that they can teach people, let them sensitize people hoe to mix the chlorine, the measurement let them know, the quantity and the amount of water let them know how to go about it so that they will be able to do the correct thing instead of you continue to cure Ebola and when it is finished you begin to cure another thing. They said it causes skin cancer, we want to know how true is it because that has made people to be afraid to wash their hands with chlorine, some people when they smell chlorine they will not wash their hands there he or she prefer to go and wash hands somewhere with empty water because they say it causes skin cancer so we really want to know if it is true or not and let them teach people how to mix this chlorine, the measurement let them know and the amount of water and everything let them know so that they can do the correct thing instead of you continue to cure Ebola when it finishes then you cure another thing. They say it cures skin cancer

M: Well.

END

**ADDITIONAL PART OF INTERVIEW, OBTAINED BY COLLECTOR 2 AFTER CONSENT IN PERSON with two (2) of the original participants, March 2015:**

M: Good afternoon, in our last discussions, you said that, we the younger people do no listen, what do you mean by this, what are they not listening to, and you also said, they are still washing dead people, why?

R1: “What do we mean that we the younger ones do not listened, because we the younger one do not take instructions? For instance they had said we should not touch a sick person neither the dead, some people do not listen to that, some people once it are their relatives, they will touch, and take care of their sick relatives, maybe the person is infected, you also will be infected”

M: That was the reason you said they are not listening and they are still touching people?

R1: “Yes”.

M: Yes sister?

R2: “We the younger ones are very stubborn these days, as they are telling, some children like walking about, visiting people’s house, they don’t know who is sick of Ebola, you will visit people’s houses, you may not know who has left the Ebola sickness there , they will also contact the sickness, so it was happening”.

M: Some people still do not believe Ebola exists and whilst some of them are still taking it on political lines, and you said again government is using this Ebola situation to find money. I want you to give me reasons for all of these statements”.

R1: “We did not ever say so, but we knew some people do not believe, Ebola exist but we don’t think government to make money out of this, or we to turn it into politics, because this is a sickness, plenty people are dying and we also had sympathy and we are empathizing with, we can’t say the sickness is about politics, we can’t do that, but we believe that there are people that do not believe this Ebola sickness exist, and we are taking the precautionary measures”.

M: Which events that took place in this area, which made the people to believe that Ebola is real? *(Noisy background)*

R1: Well, we had cases down here, at (- -name of the place - -), that particular place was quarantined, that shows the Ebola sickness is real, that made the people to be afraid and cautioned”.

M: Can you tell me the specific day or date that they find out that that person had Ebola and the time the quarantined the house?

R1: “It was in December

M: December, which day, around the 20^th^ or early?

R1: “No”.

M: Ok, from what you told me, you said people were calling 117 before but later became afraid to call, can you tell me why were they afraid and why the changes?

R1: Why were people afraid to call 117, because some people after calling 117, the ambulance will come, took their persons, so at the time of visitation, they will tell them, their sick person has died, so when they had sick person or they themselves are sick, they will call neither go to the hospital, some people will not call 117”.

R2: “Some people may want to bury their loved ones, secretly”.

M: Ok, Some people washed patients that had died of Ebola, so can you please tell me why they are doing that and why they had the thoughts that, they should washed their loved ones that had died of Ebola?

R1: “They do not believe that the Ebola sickness exist, and some are doing it out of love”.

M: So they did it openly or secretly?

R1: “secretly, I saw them closing the door”.

M: Have you notice any secret burial taken place?

R1: “No, the only thing we knew about it” (*Noisy background*)

M: You told me this Ebola business is about money making?

R: “We did not mention any money business”

M: You mentioned again that, when they spray the chlorine in the ambulance lead to the death of people?

R1: “The spray of chlorine suffocated the people, because the ambulance do not have airways, all the part of the ambulance is closed”.

M: Where these beliefs came from?

R1: “We heard from the people, but we always see the ambulances, they are totally sealed”.

M: Have you seen anybody with Ebola instead of going to hospital, they will prefer go to the traditional healers?

R1: “No”.

M: “You said again, the ambulance services, when they go to collect dead bodies, instead of collecting the dead body and leave, instead they will end up thieving, who are this thieves and what they steal?

R1: “I did not said that”

R2: Well some people, they are kleptomaniac, they do not heard a steady hands, we they go to collect dead bodies, these people may see things that they like and take it away, without the consent of the people”.

M: who are the people doing this?

R1: “It may the workers or other people that may assist in taking the dead body into the ambulance”.

M: You said again, these people have powers that is why when they put you into the vehicle, you will be killed, who are these people, and why do people think this is?

R1: “The people that went to collect the bodies”.

M: Are they the people that have power, the people that collect the bodies?

R1: “Yes”.

M: What kind of powers they have to kill people?

R1: “Hence you are in the ambulance, they had all the powers to do anything to you, like one incident that happened, they went to collect man at (- -name of community- -), when they load this man into the ambulance, they later put the man in a plastic bag, and they said the plastic was suffocating the man”.

R2: “I don’t have any idea about that”

M: But do you know why are they killing people?

R1: “At times they will do it out of grudge”.

M: what are reasons for the grudge?

R1: “Like for instance this people have lived in the same area, one will be in need of something that the other has, but will not give it to him or her, because they had been rivals”.

M: You also mentioned about an injection that is given to people that leads to their a quick death, what do you mean?

R1: “We did not talk about that, we only spoke of what causes early death is when they took a person to the hospital that is totally and entirely weak, some in the process of taking them to the hospital, they have almost given up, the injections that they gave, killed them because the injection is powerful, because they are totally weak, they may not withstands the powers of the injection”.

M: Who administer these injections?

R1: “The Nurse, the health workers, the people that worked at the centre”.

M: Who told you this?

R1: So they also said?

M: Who are they?

R1: “Some of these survivors that come back, they explained to us?

M: The Ebola patient that have survived, the Survivors?

R1: “Yes”.

M: Ok,

R2: “We were having a nurse that went there willingly and healthy, so she started calling parents the way they are treating her, she is really doubtful if she will survived it, because the injection they are giving her is too powerful, she died, she went there healthy but she died”.

M: You also mentioned of giving someone last respect, what do you mean by that?

R1: “Like the way they bury people, they do not do it that respectful way, they will jam-pack the body together and when they go, they will fling it as how they wanted to do and gathered a trip of soil and spread it over the corpse, if they are burying people differently in own graves it would be better, but mass grave, burying one and fifty people in one grave, do you think, that is a respect? That is no respect, they will just bodies and do not take a proper care, but if it is their parents, they will take proper care of them, and they will even prepare a special place where to keep them”.

R2: “They put them into plastic bags”.

M: Another person said, you have to give the person the last respect so that, what do you mean of this?

R1: “This means the person will rest in peace in his or her grave”.

M: Someone said again, if the doctor is a Godly person, what do you mean by this? (*Dog barking*)

R1: “It means the doctor is a God fearing person and he has respect for human being, like in the process of burial, they will do it fine, thinking that he or she will die one day”.

R2: “In that case if the doctor is a Godly person, because in some certain cases in hospitals nowadays some doctors deny their profession, some will say no I am not a doctor, because they are afraid, but as a doctor if even you enemy is sick, you have to take care of the person, because you have taken oath to save life, you are also paid for the job. So if you are a Godly person, you will attend to the sick person”.

M: Do you have Ebola survivors in this area?

R1: “Yes”.

M: When the Ebola survivors returned, do you welcome them?

R1: “yes”

M: Do you meet them?

R1: “Yes, we even mingled with them”.

M: “If you were an Ebola survivor, when you returned to your community what are the problems you expect from your community?

R1: “It is about two things, if the community, had the knowledge, when had come, I do not have the tendency to transfer the sick, they will welcome me again, but in some communities, that the Ebola survivor do not have the tendency to transfer the Ebola sick, and they do not have the awareness that we have to welcome this person with open hand, they push far away from me and they will stigmatize me. If the community is not well sensitized that an Ebola survivor does not transfer the Ebola sick, they will reject the person, but if they are sensitized and aware they will welcome you with open hand”.

M: “If you were an Ebola survivor, when you returned to your community what do you expect from your community?

R1: “Well I will be welcomed with opened hand, and I will tell that I have cured from this Ebola sick, I will not infect and I will not get the sickness again and I will tell again this sickness is real”.

M: How do you think other communities treat Ebola Survivors?

R1: “Some communities treat them good, but some will push far away from them, thinking the survivors still have the Ebola, and after they have survived they said you should sex until after ninety days”

M: Thank you very much for talking to me.
