# Supplementary material for: Development of a set of community-informed Ebola messages for Sierra Leone
Source: PLoS Negl Trop Dis. 2017 Aug 7;11(8):e0005742. doi: 10.1371/journal.pntd.0005742 (PMC5560759; doi:10.1371/journal.pntd.0005742)
Supplement: S1 Appendix — (ZIP) [file pntd.0005742.s001.zip › Ebola messages - FGD and interview transcripts/R2HC Ebola Fieldwork 1/R2HC Ebola F1 FGD-FEYOU-Urban2.docx]

| CODE | **R2HC Ebola F1 FGD-FEYOU-Urban2 (urban focus group discussion)** |
| --- | --- |
| DATE | February 2015 |
| DURATION (minutes) | 85 |
| Collector nr | 4 |
| LANGUAGE INTERVIEW | Krio |
| **TYPE FGD** | Young female |

**PERSONAL DATA PARTICIPANTS**

| Nr | Sex  (*F/ M*) | Age  (*in years*) | Education Level (*e.g. none, Primary, secondary, tertiary*) | Language (*e.g. Mende, Temne, Krio)* | Religion | Job / Employment (*how they earn their living e.g. farmer, teacher, trader*) | Role in community  (*e.g. youth leader*) |
| --- | --- | --- | --- | --- | --- | --- | --- |
| 1 | F | 25 | Tertiary | Krio/Temne | Muslim | artisan | none |
| 2 | F | 22 | Secondary | Krio | Muslim | student | none |
| 3 | F | 25 | Tertiary | Krio | Christian | student | none |
| 4 | F | 24 | Secondary | Krio | Muslim | trader | none |
| 5 | F | 23 | Tertiary | Mende/Krio | Muslim | trader | none |
| 6 | F | 27 | Secondary | Krio/Temne | Christian | student | none |
| 7 | F | 25 | Tertiary | Temne/Krio | Muslim | teacher | none |
| 8 | F | 30 | Tertiary | Krio | Christian | farmer | none |

**TRANSCRIPT:**

M: How has Ebola affected this community in which you are?

R1: “In this community?”

M: Yes, how has Ebola affected this community?

R1: “Well, it has affected us in various ways; it killed people and our loved ones”.

M: Yes can too add something?

R2: “Well Ebola has really, really affected us in this community. Like, we lost most of the big people who have respect in this area, it made people talk to their companions fine because of this Ebola, that barrier; people will say you have Ebola, so they will not go near to them. So like it has created enmity between us and many people and we are not happy about it”.

M: Yes my sister can you too add small thing to that?

R3: “Well the closing of school, the 6 to 6, some market women it is around 6,7 or 8 that they are able to sell but like with this Ebola the State of Emergency, is like there is the barrier. Like in our own area, there was a time those who are down there should not come up here and those up there should not come down here, even if you beg water to drink, they will destroy the cup so nobody would want to use that cup again because of the area the person is coming from”.

M: Yes my sister do you have anything to add to that?

R4: “Yes, just like my sister has just said, it has brought backwardness in schooling, no business”.

M: “Yes my sister, add small thing to that?

R5: “It has brought schooling backward and has killed big people”.

M: Yes my sister you too how has Ebola affected this community?

R6: “This Ebola has affected this country greatly not only the community, one - the closure of schools and then two - the hesitation of people that if they go to another person’s house they will get the sick because most of the houses which they quarantine they do not allow us to go there even if the person is your friend, you can only stop at the street if you want to talk to them. There are some people after this quarantine because you did not go to visit them, when you pass by and greet them they will not answer you and that have created enmity among people”.

M: Yes my sister can you add small thing to that?

R7: “Well if I can buttress what my sister has just said, Ebola has affected us greatly, if I can talk, I was a victim myself, victim in the sense not that I was infected, but like where the Ebola was they had quarantined the people, they quarantined my people and I am not happy about that, although nobody died but one or two of my sisters were sick and that brought enmity into the house, so people were looking at me with a scorn and I did not feel fine. I know it is because of Ebola and I am still not feeling fine because even like me I should be thinking of doing something to ensure that I graduate so I did not feel fine about that”.

M: Ok, thank you very much. Yes my sister you too can add something to that? What is your own view, how has Ebola affected this community?

R8: “Yes based on what my sisters have just said, where I am is one of the hot spots for Ebola, that last house close to us which we share boundary with, they had it, also I can say the mother-in-law of my brother died, my good people died because of this Ebola and it created a lot of enmity. Now certain people are saying we were mocking them because they had Ebola, certain people now do not talk to themselves because of Ebola. And this Ebola the only thing we can do is to pray for it to end so that we can move freely in the country”.

M: Yes my sister, what I want to know again is that, do you know of anybody who has had Ebola?

R1: “Yeah, my friend”.

M: Ok, you too do you know of anybody who has had Ebola?

R2: “Yes”.

M: Yes my friend, do you know of anybody who has had Ebola?

R3: “Yes”.

M: Yes my sister, do you know of anybody who has had Ebola?

R4: “Yes, my neighbour”.

M: Ok, yes Ma?

R5: “Yes I know many people who have fallen victims of this Ebola”.

M: Yes, you in the middle?

R6: “Yes I also know somebody who is my Aunty”.

M: Ok, yes my sister?

R7: “Well, like I was saying, my neighbours”.

M: Ok, so what I want know again is, I want you to give me reasons why has Ebola spread in Sierra Leone?

R8: “By touching”.

M: Ok, why has Ebola spread in Sierra Leone?

R1: “The washing of dead bodies”.

M: Ok, yes my sister, why has Ebola spread in this country?

R2:”By washing dead bodies”.

M: Yes, is there any other reason?

R3: “The too much spray which they spray the houses”.

M: Yes Ma?

R4: “Because they failed to listen to instructions, that is why, they gave them ways and mean to prevent Ebola, this sickness but people failed to go by the rules. Some do not even believe that it is a virus that exists, so this stubbornness made the thing to spread.”

M: Yes my sister can you too give me your own reason why Ebola has spread in this country?

R5: “Well my own reason is that it is because of the carelessness of the government, they failed to put things in place, what I am trying to say is that, of cause we know that the sickness started in Kailahun (=first district in Sierra Leone with Ebola cases), if they used their initiative to quarantine them this sickness would not have spread but they were late, now when they want to irradicate this sick, is late. So that is my view”.

M: Yes my sister, can you too give me one reason why has Ebola spread in this country?

R6: “Because people do not believe that the sick is real, even when they say there is the sickness people will deny that the sick is not real, so that is one reason”.

M: Yes my friend, add one reason to that, why has Ebola spread into the country?

R7: “This is directly to the government, as my sister said, because like the neighbouring countries, Guinea, Liberia they have Ebola, when this happened they could have closed the border to prevent this country, because like Yayah Jameh (=president of The Gambia), he closed his boundary and up till date nobody can enter Gambia freely, so I think this is one of the major reasons why Ebola spread”.

M: Ok, so do you have any way that you call Ebola in your local language?

R8: “As for me I only understand Krio, I do not understand my own language”.

M: Ok, Do you have any local way you refer to Ebola in your language?

R1: “I have never heard them call Ebola in another language except Ebola in Krio”.

M: Ok, my sister you too?

R2: “No”.

M: My sister you too, don’t you have any name by which they call Ebola? Yes my sister in the middle there?

R3: “I have never heard, except one time when they were saying that this is a sickness which they call in Temne “Anmisali Kantoff”, so I don’t know if that is how they call it”.

M: I want you to tell us the meaning of what you have just said, it is very interesting, you are the only one who has said this talk?

R3: “Well, that is what I heard them say; “Anmisali”, that means it is a sickness which God has brought to the world that whether you bad or good when you fall victim you just have to go”.

M: Yes my sister do you have any other way that you refer to Ebola in your language?

R4: “Is just Ebola”.

M: Yes my sister do you have any way you refer, to Ebola in your local language?

R5: “As for me I don’t know any other way to call it except Ebola”.

M: Ok, yes my sister?

R6: “Is just the same thing”.

M: Ok, there are some people who do not believe that Ebola is real, my sister do you know such people who do not believe that Ebola is real?

R7: Mhmm, like before, I myself thought that Ebola was not real. I took it that they just wanted to create the sickness, not until when it killed my friend and when I came to (- - name of the interview community - -), the way I saw how it killed people, people died and that was the time I knew that Ebola was real, so”.

M: Those who hold the belief that Ebola is not real, why do they hold such belief?

R8: “Ok, let me say me as an example why I did not believe that Ebola is real because it had not killed any of my people at the time and then the area where I am it has not killed anybody yet and I have not seen the sign yet. Because they said in Guinea when you have Ebola the signs will show when the person’s body gets swollen and I have not seen that yet, so I did not believe”.

M: Yes my sister do you know of anybody who believes that Ebola is not real?

R1: “Like the compound where I am people were denying, my uncles were denying that Ebola is not real, I have one of my uncles who used to deny that Ebola is not real”.

M: Why does he deny that Ebola is not real?

R1: “Well he said the way they show people who have Ebola over television, the way they look, he said he has never seen an affected person with such signs and symptoms; that when somebody has Ebola their body gets swollen, at one time a band (=video band) came to play here and they showed us people affected by Ebola, but the way they showed the people I have never seen those signs so that is why he did not believe that the sick is real”.

M: Yes my sister can you add small thing? Do you know of anybody who does not believe that Ebola is real?

R2: “At first. Because they had said that Ebola has no medicine and then later they came back and when you go to the Treatment Centre you will recover quickly. That brought about the confusion, people started arguing that Ebola is not real and others say it real’

M: Yes my sister you wanted to say something, do you know anybody who has been denying that Ebola is not real?

R3: “Yes a lot of people were denying that Ebola is not real because they said if anybody has Ebola he or she will not be able to walk or neither do anything, so I did not believe that Ebola is real”.

M: Ok, yes Ma?

R4: “Yeah, at first people did not believe that Ebola virus is real because according to what they tell people about the signs and symptoms such as bleeding, frequent stool, frequent vomiting. Somebody can get all of those signs then you still die. They always say that when a person has those signs is Ebola, so somebody can be sick and die without showing all those symptoms so a lot of people did not believe that it is Ebola, maybe it is another sickness so that is why many people did not believe. Even dead body, they have told us not to wash dead body. People do not believe because maybe the person who died did not show any of the signs and symptoms so they believe that they are going to wash the person so when they wash the body they will later come to know that it is Ebola ‘.

M: Yes my friend, you can add small thing to that?

R5: “Yes Ma, it is based on the transmission of the messages that they give. Because when they started in the neighbouring countries they told us that when one is affected blood oozes out from every part of the body and at the end of the day they translated everything into Ebola, vomiting, fever, and those thing are normal sicknesses that has been affecting us because I remember each and every year we used to do sensitization on Cholera but this year there is no sensitization on Cholera only Ebola. So then most people were sick of Cholera, so like people get sick the same like Ebola, when they are sick they go with them at the Ebola Centre when they do the test is Ebola, so like me till date I am not denying that Ebola is real but I am really confused, I really want them to go to the radio and tell us about this Ebola that can be more preferable than”.

M: Yes my sister, you too add small thing to that?

R6: “Yeah, what I want to say is just the same like what my sisters have said. I have some people in this community who do not believe that Ebola is real because this Ebola they are talking about, when somebody has Malaria the person can feel fever, vomits, and can get head ache and the person gets high fever according to what the Doctors said, and when they also said that if you have Ebola you can bleed, but when somebody has sinus, that chronic sinus you can have blood coming out of the nostrils, if they take all those sicknesses and put it into Ebola; when somebody is warm, has head ache, and the nostrils running, you know, anyway we have doubt over this sick. May God be sorry for us. But we do agree because the elders said it is the sickness and we do not know so we just have to abide to what they say”.

M: Yes my sister, you too can add small thing that, do you know of anybody who do not believe that Ebola is real?

R7: “Well I used to denying, and till date I am denying”.

M: Why do you deny?

R7: “Because according to what they told us about how the sickness affects people that is not what we are seen those signs that is why I am denying up to date that Ebola is not real”.

M: Yes my friend add small thing to that”.

R8: “My uncle fell sick in our hands; I took care of him at the hospital until he died. My uncle, my uncle’s child about five years but we do not know what happened when we came back home, after the three days we came back home I feel sick, but when I fell sick if I was taken to the hospital like they are saying different thing by now because I vomiting and toileting, that was the only sickness that was affecting me, I could not walk. But as God would have it, my sister was close to me, everybody ran away from me, they were the only people moving around so I was treated secretly until I felt better. No sooner I felt better, my husband too fell sick. Him too he was treated at home, as he was getting better my children too fell sick both of them, the one was not going to toilet and the other was just vomiting, but the other could not vomit or toilet he was just getting fever. Like my sister who was taking care of me at home she did not even get sick she only got colic but she died in my hands. So I am confused about this talk about this Ebola, I do believe, but on the other hand I do not believe again”.

M: So what I want to know again is about those Ebola. Messages, I want you to give me some examples of them? Which Ebola messages have you heard?

R1: “I do not understand”.

M: The Ebola messages that they have been giving you, I want you to tell me small thing about them?

R1: “They only told us that you should not touch your companion, when you touch your companion you are going to get Ebola, you should not wash dead body, then when a person dies you should not go to the burial because when you gather you are going to get Ebola, something like that”.

M: So, what do you think about the messages that they have given you? What is your personal opinion about them?

R1: “Like me, like I told you, I don’t believe so I used to touch, like those who are nearer to me I do touch them”.

M: So my sister, can you give me some of the Ebola messages that you have hear?

R2: “What I heard about this Ebola is that they said when you have high fever that means you have got Ebola, that high one, when you vomit, you have got it, so I don’t believe those things. For me, from the time when Ebola broke out cold is my own sickness, my body gets warm, it reached a point they started saying ay... Like the last time I was vomiting and a boy was passing and he said ay, (-- first name of this respondent - -) you are vomiting, isn’t it? Then I told him please do not alarm it. So but the things that are happening nobody can’t say things cannot happen, my body never got warm, so like I do not believe it is a lie, for me”.

M: Yes Mama, can you too give some Ebola messages that you have heard and what you think about them?

R3: “Well, the messages that are coming in are well understood but they are normal signs that have been happening”.

M: The messages that you have been hearing, give me some?

R3: “Well when you touch somebody, then when somebody’s temperature is too warm, severe headache is Ebola”.

M: So what do you think about those messages?

R3: “Well think they are normal signs which have been happening but since they say they are signs of Ebola, we abide, like the area where I am, (- - name of interview community - -) is a hot zone, so even when you want to come near me I will say don’t touch me, don’t talk near me”.

M: Yes my sister, give me the Ebola messages that you have heard?

R4: “Well according to the messages which I have, at first when somebody is infected if the person has not got fever yet, that high fever even when you touch somebody the sickness will transfer except when the fever is very high fever and you see the person vomiting, toileting; then you can avoid that person. So I just took it that when the person vomits and you play with it then you will get the virus, when you play with the stool you will get the virus, so I thought it fit that this thing is true so let us avoid, because when it enters one family, all of you will go if you did not take your time. If you did not abide to the instruction which they give obviously the whole family will go”.

M: Yes my sister who is holding that fine phone in hand? I want you to give me small Ebola messages which you have heard.

R5: “Well they have been advising us not touch a sick person even if the person is your mother just call 117 for them to come and take her, they also told us that we should wash our hands with chlorine and soap and water. We did not believe before, but to protect myself and because I like my life I just abide to what they tell and today even though I am not yet safe because Ebola has not yet ended but I thank God”.

M: Yes my sister, give me some Ebola messages that you have heard?

R6: “Well the messages that I have heard is that they said when you see any corpse, do not touch it, and when somebody has high fever, and more likely than not when somebody is at the point of death and worst of it when somebody is dead, they said when you touch it the virus will transfer, because they said the virus just needs blood, if I were to name this sickness, I will call it vampire because is only vampire that sucks blood. So they just name it Ebola but it is vampire because if there is a virus which does not need flesh only blood”.

M: Yes my sister, give me some Ebola messages that you have heard?

R7: “We only want this sickness to end in our country”.

M: So, what I want know again is that, which good talk can you tell somebody who has a sick or Ebola patient at home to encourage him or her to take the person to the hospital?

R8: “I will tell the person, because lately people are getting admitted and discharged now. It is not like before when they go with your person it will be difficult for you to see the person. But now when they go with a patient he or she has great chances of survival, especially when he or she go by him or herself he or she will have ninety percent chance to live. When they go with him or her is about seventy percent chance, which is quite different from when they come for the person. So I am advising anybody for now, I am not talking about in the past, but for now if anybody has sick person to go with the person”.

M: Ok, my sister you too, can you tell me something, if a person has sick of Ebola, what would you tell the person to encourage him or her to take the sick person to the hospital?

R1: “Aaa, God, because truly speaking, this is from inside my heart, when they carry people they kill them there. Some people can get better and even call their relatives to say they are feeling better, in one or two days they will call again to say that person is dead, so like me, what is in mind when my person is sick let them treat him at home, that is what I think”.

M: Yes ma, which words of encouragement can you give to somebody who relative is sick of Ebola to take the person to the hospital or the Ebola treatment?

R2: “Well since the person has been infected, and we cannot confirm when the person is at home whether it is Ebola or not, maybe the signs and symptoms which the person shows may be thing is not Ebola the person used to get those signs before the coming of Ebola. So like if he or she sees that the person is getting fever, feeling head ache, so they only need to convince the person for him or her to go for treatment. You can also tell him or her that it doesn’t mean that when your body gets warm means you have Ebola, you can tell him or her to go to hospital so that they can treat the person, they will confirm whether you have Ebola of not. But like if they go there again the next thing they said they do inject people on their big toe by the time you think of it for some hours the person is dead. So like me, my own somebody, when I heard that that is what they do people in the hospital I would not like my person to go there”.

M: Yes my sister, can you add small thing to that? What do we tell people to convince them to take sick patients to Treatment Centre?

R3: “They said they were injecting people for the virus not to spread, but since the arrival of the expatriates, people have started discharging. Although in our community out of hundred percent who went only forty five percent returned, fifty five died. Some of them, I don’t know, that is all I can advise, when a person is sick do not wait until it gets worst ah home”.

M: Yes my sister, can you too add something to that?

R4: “My own advice is that if there is somebody in the community who is sick, if the person sit at home, they will ‘Pepe Doctor’ (= treat by unqualified person, quack) you and that is how the person will spread the virus, the earlier is the better if you go to the hospital to seek medication because this sick is not for one person, when it enters into one family it will make sure to eradicate everybody in the family. So to avoid that - the earlier is the better, the sooner you get infected you have to go to hospital straight off”.

M: Yes my sister add small thing there, isn’t it?

R5: “If it were me, I will advice the person to take the sick to the hospital, he should not touch the person, let him just encourage the person and then advice the person strictly to tell the Doctors if there is a way to carry the patient to (- - name of a treatment centre in the same district - -) because there they have white men there. Those who are going there, the survivors say they take are taking great care of patient there while the other Centres are complaining that they are not taking care of them, and when you survive they give you money and they will give you something that you could feed on for the next three months because when you come back people will be afraid of you saying that you have had Ebola once so they will not come closer to you, but they will give about seven hundred and fifty for you to hold on to it”.

M: Yes my sister, you can add small thing to that?

R6: “I will tell the person that if someone is sick and the condition is rough they have to call 117 for him, they should not touch the person and should be giving him or her jelly water until they come to take the person”.

M: So, which channel can be the best to give out Ebola messages so that people can get it quickly?

R7: “Radio”.

M: Why do say we use radio?

R7: “Well especially when it is 6 O’clock, everybody wants to listen to the radio to know what they are saying on update and results, so radio is now part of people now”.

M: Ok, very good, Yes Ma, apart from radio, is there any other channel that we can use so that people can get it?

R8: “That mouth radio, I said “mouth radio” (=word of mouth) because if you have said something here, you will not know how it gets to (- - name of central business point in the interview district - -). So is like it is not everybody that has radio, some do not even listen, some prefer when somebody is standing then you tell the person”.

M: Yes Ma?

R1: “I just want to buttress what my sister has said just now. We who are in the community, should be used to talk to the people, I can talk to this woman when I come he can listen to me because we are used to each other, I can pass the message to him and he can understand and accept it, even if the person did not accept it at that moment but later the person will think to him or herself that since we are all together whatever I have told him or her, he or her should take it seriously, so he or she will consider than you go and take somebody whom they can just push aside and pass without listening to him or her. If we use this method to pass the message it will go down well”.

M: Yes my sister?

R2: “Just like what my sister is saying, let them use us, we do it to ourselves and tell us to be passing the message. And the most important one is those social networks because now even the least child has access to internet so I believe that. Then even through radio, they must find a way how to spread the message, that is it”.

M: Yes my sister?

R3: “Just like what my sister is saying, we can use the megaphones. We have youths here, like me I want a job, if they come here and take young people in this community, you will see how they will spread it, even if someone do not want to listen, he will listen. Like you if I meet you I have a way to encourage you and talk to you before bringing the message to you. But this government is careless. At overseas those who are able to pass the message, if they come to this community and give us hundred thousand (= Leones, a bit over 20 USD) about each we will go about spreading the message, o yes, even if it is for two or three days. We are here we do not have job, if they come we will spread the message and it will go down well to the people, because our people is Temne that they hear and most of us speak it, but the radio maybe is just English that they speak so how can the person understand even if the person has radio. Here is a Temne compound, they talk Temne so you see how the message can go? But this government, don’t bother”.

M: Yes sister?

R4: “Just like what my sisters said, let them use the youth in the area for them to spread the message”.

M: So if a person has Ebola, where would the person go first? Would he or she go to a ‘Merasin man’ (=traditional healer), or to the hospital or to the existing Health Centres or to the Ebola Treatment Centres?

R5: “The person will first think of going to the Ebola Treatment Centre”.

M: Yes Mama, if person has Ebola where would he or she think of going first?

R6: “He or she will first go to the hospital”.

M: Yes Mama?

R6: “The person would think of going to the Ebola Treatment Centres but at the same time he or she would be afraid”.

M: Yes my sister, where would the person think of going?

R7: “Well some them when they get Ebola, some take it that someone has sent the sickness on them, so they would first go to the ‘Meresin man’ (=traditional healer) first to heal him or to check what is wrong with them”.

R8: “Excuse me Ma?”

M: Come and talk this first before leaving. If a person has Ebola where does he go first?

R8: “Well that is if the person knows that he or she has Ebola and he or she has seen that people have been there and coming back, the first thing that would come to his or her mind is to go to hospital but if he or she doesn’t know that he or she has Ebola he or she will think that is just any other sickness so he or she will first find a native Doctor (=traditional healer)”.

M: Yes Aunty, you too add small thing to that?

R1: “If can add my own understanding, at first when this sick came, some people when they get the signs and symptoms they go to the ‘Moray man’ (=sorcerer), and even in this area, that is what that killed many people, where I told you my father stays, where about ninety percent of the people are infected. He runs up country, so when he came not knowing that she had been infected by Ebola, what made the thing to kill many people is that when the woman came she was a pregnant woman but she could not toilet or urinate so they said oh she has been ‘Gbagba’, she has been ‘Gbagba’ (= the body of the person is ‘held’ by a witch as punishment, resulting in no faeces/urine and weight loss) so a Pastor went and prayed on her, a ‘Sowae’ woman (= important member of Bondo society, involved in initiation of girls) went there but she too died because they felt it was not Ebola because of the way they are passing the message, like I was telling you, they said when you have Ebola you toilet and vomit, but that woman cannot go to toilet, cannot urinate so they took it that it was not Ebola so many people down there to us if you ask them they will tell you that woman killed more than twenty people in the area just because of this. So that is it”.

M: Yes my sister, can you add small thing to that? If a person has Ebola, where would his heart run to go first?

R2: “Well before he or she goes to the Treatment Centre, I believe that if you begin to feel certain ways because there are certain ways that you feel, just go for test to the nearby Centre, they can take you to the Centre for Ebola”

M: Ok, so I want us to talk small about those Ebola related Services. Like the Ambulance Service, what can you tell me about it, the good and bad things that people talk about them, I want you to tell me first about the good thing. Yes my sister, I want you to answer this, the good thing that they do?

R3: “The good thing that they do is that when they come to collect sick people and carry them away. Then the bad thing that they do is some of them there can be no patient in the Motor car but for them to be just fearful for peoples’ to troubled they go along blowing the horn ‘Weee!worr! weee!worr! (=imitating sound of siren), ‘wae dem wae dem’ asking where are they? Where are they?”.

M: Yes my sister what do you hear them say about the Ambulance?

R4: “The good thing that they say is that the Ambulance is fast in terms of speed, and the bad is about the chlorine, because if somebody is not well and they spray that chlorine on the person, maybe that person does not have Ebola, there are people who have high blood (…?...) and then you suffocate the person again with chlorine and you know that they need breeze, and you squeeze them into the Ambulance before they reach with them some would have died. We are hearing that complain from different communities”.

M: Yes sister you too can add small thing to that, the Ambulance Service, what do you hear people say about them, the good thing?

R5: “Well the Ambulance Service, yes because there are enough Ambulances in the country now, because I remember when Ebola started, I am somebody who moves about and I hear a lot, they said the people were not coming around that is why more people died because there was no good treatment but now they come on time. But the bad thing that they do is, at least when somebody is sick he or she needs care, needs attention. There are some people when they lift them to put them into the Motor Car (=Ambulance) they just do it like when you are lifting a dog that you do not want into your house, because some dogs have respect in their house, but they will just take the person and send him or her. One woman died, the woman was sick and could not walk in our community because when they come they need to protect themselves, and they are paying them for the job, when they came instead of helping the woman they did do that, it was the woman’s husband who wore the gloves and helped the woman into the Ambulance. So you see, I wonder if they had taken that woman directly maybe the woman could have saved, but all those they took along the way, because they went on collecting people on the way and I think the best thing they should be doing is when they take one patient they should go and leave the person first because there are many Ambulances now but they don’t do it, they go along picking other people. It is bad”.

M: Ok, what about the Holding Centres, the Treatment Centres where they carry the patients, what do you hear people say about them?

R6: “Well before they were not treating people well because they too were afraid of the people, when a sick person goes, some people will just go because they need the money so they were not treating people well. But now we are getting good information about them that they are now treating people well, they are no longer injecting people on their toes, people are now discharging from hospital”.

M: When you said just now that people just go there because of the money were you talking about the Staff?

R6: “The Staff, the nurses”.

M: Yes sister, you met us talking about the Treatment Centre where they carry patients, what do hear people talk about them, the good things and bad things which people talk about them. Do you remember anything?

R6: “Yeah, especially the bad things which they were saying about them. Some were saying that when you go there they will inject you on your big toe so that you can die for you not spread the virus to another person. Then at the same time at this later part when you go they take you to a place call (- - name of a treatment centre in the interview district - - ), there they hold people fine, they feed them three times a day and when you are about to discharge they give you some things and money, they will do something for you that you can start life with”.

M: Yes sister?

R7: “Yeah, the bad things which I hear about the Staff, is that they said when they admit somebody the way of dress, they dress fearful and some people have hypertension when they see them they become afraid and get panic, even if they are feeling hungry the way they pass the food to them, pass the food in the sense, when they go with the food, they will not see the particular person who is giving the food, you cannot even see the face of the individual, to say he can encourage the patient and tell he or her to say bear it, person just [take] it that he or she is in hell, the chances of survival is less because if they dress fearfully how cannot the person communicate even if the person wants to send a message to someone to take care of his or her child, in the past they were doing it, the person can pass message but now, if you are able to see the person’s then you will be able to send a message. Some patient can tell you that the sooner they see them their heart jumps, they feel discouraged, so some people die by that”.

M: So, let us talk small about the Burial Team. I want you to tell me small thing about them, the good side and the bad side?

R8: “Well the Burial Team I believe that they never educated them, when they come to take dead body, some people died by stress, the stress, when they come to take the dead person they spray all over the house whether there is food stuff or anything; in the past they were not changing the clothes, when they come they spray the whole house. They would not even advice the people not to enter the house after spraying, they will take certain people who have respect in the area, they will just take the body and fling it, you will hear the sound, ‘veep’. Just like when one of my sisters died and they went to take her. The Red Cross have some regard to bury people. They dressed her, prayed over her and they took her quietly and took her away. Then when they sprayed the house they told the people not to enter there for three days. When the Red Cross came in to help the Burial Team, it looks better, the death cases reduced”.

M: So the Burial Team was divided into two?

R8: Yeah, well I don’t know if there are two or three sectors, there is the Red Cross which assist people. They, when they spray people’s house they advice them, that spray was disturbing people”.

M: Yes my sister can you tell me about the Burial Team? Those whom government has appointed to bury dead body?

R1: “Like for me sitting here, like my sister who died, I did not even see where they took her body because I was not in good condition, I pulled out, I went and find somewhere to sleep. But like this I was there, they took the body fine, they even advice her husband to go and see how they were going to bury her but him too was not feeling bright so he couldn’t go. So that is it”.

M: So what about the 117, that phone line which they say you should call, I want you to tell me small thing about it, the good thing and the bad thing that people talk about them .so that we can gather all these information. Yes Mama, the 117?

R2: “Yes, I remember at one time I was in (-- another area in the interview district --), somebody died in (-- another big town in the interview district - -) so they call them and then they said any time somebody dies they should not touch the body until they call 117, they call them and the body had been there for three days and had swollen about to get burst, nobody went there, the woman even went on air and talked about it that there was a dead body there for over three days about to get burst and till then they could not come, but they had said when somebody dies we should not touch it but to call 117 but when they call them they do not come. Then at times you can call and they will answer that they are coming but they will not come there and that is not good. That can even make some people have the mind to take the body and wash it and bury it because they cannot leave it in the house to smell and a dead body should not be in the house, when the person is dead they have to take it and go and bury it, so if they call them and did not come, even if somebody die in the vicinity they will take it and bury it”.

M: So, you don’t have any good thing which you hear about them?

R2: “No”.

M: Yes my sister you too can add your own view?

R3: “Just like what my sister said, there is no lie there, it is true; they do not have any regard for people. We now believe that this sickness is real, we are not taking that challenge, although they too they used to call them in some area in (- - name of interview community - -) to say there is a sick or dead person in the area when they come and search they will not see any dead body so that is why when they call them in certain area they will not go there again. But I know that even the teacher, the pupil can make fool of him in class, when they say that is their job, let them do it. That is what I have”.

M: Yes my sister the 117, can you tell me something about them?

R4: “Well, like I am not there, I am not the one sitting behind the machine but because maybe it is the way the message is passing. Before now they would not come until say in one week, but now to my own observation they do come sometimes. But what I believe is leading to those things is the passing of the massage because there is a way a person delivers the message, because any human being that eats salt must forget, maybe they forgot. But one thing I am advising them is that one thing they should know is that they are dealing with and they themselves are lives. If you value your own life them you value the lives of your brothers and sisters’.

M: So, those who survives from Ebola who we call Ebola Survivors, how do people treat them? Yes my sister?

R5: “Hmm, in the past they used to tell us not to push them, we should encourage them, but now they say they are the ones spreading the Ebola in the community so everything is just baffle everybody. At first they said we should not push them, let us encourage them, now they said they are the ones spreading Ebola”.

M: Then explain small about how they spread it?

R5: “Well more especially through that sexual intercourse, like when your boy friend is discharged”.

M: Yes my sister

R5: “And they were also telling us that survivors do not have Ebola again, and now they say”.

M: Yes my sister, I want you to tell me small about how you treat Ebola survivors here?

R6: “Like some people, if we see that he or she has had Ebola and he or she is back, we can still be afraid of going nearer to him or her, we say maybe the person is not yet well so let’s not go closer to him or her, that he or she is just coming from Ebola hospital so let’s not go nearer to him or her”.

M: Yes my sister, how do you treat Ebola survivors here”.

R7: “Hmm, actually when they come people can be afraid of them, but with time the way you see the person you can be convinced that the person is well and no longer has Ebola, so that can give you courage to talk to the person, play with him or her”.

M: Yes Ma?

R7: “Like for us who are in the hot zones, you have a house on this side and on this other side who have been affected by Ebola so when the survivors come back you can just hear that this person is dead, it cannot even take a minute then you hear again that the person is dead but like some of them it turned out that they did not die, so when the person comes back, we do shout as if the person has just been born, and can go near to the person, because we in the hot zones had already resolved that if the person survives is fine, if he or she dies then he or she dies. So when we see them we go near to them, we become glad and we interview them about what they were going through from the Centre”.

M: Ok, yes my friend, can you add small thing to that?

R8: Well my own view we have hesitation over them because when they come government has said we should encourage them, as long as the person is not your boy friend or girl friend because they said it is through that sperm, that sexual intercourse that can gets the thing, so apart from that one cannot get the sick. So because of that I do not hesitate to go near to them or to talk to them because we even have some of them who come into this compound, we talk to them and we discuss with them and they go, we have no segregation between them”.

M: “Yes my sister, can you add small thing there?

R1: “Just like my sister, they do come here, one of them do come here but is just that he does explain how people are treating him, they are not treating him fine because government said when somebody survives even if he does not wear gloves and touches somebody who has Ebola he cannot be infected anymore according to how we got the message but is just that this I have one of my brothers where he was working the white men did not accept him again because they said once he had had Ebola and has survived, he is still infected, according to what he told us now. But we are still going close to him and encourage him so”.

M: Please, have you heard about any new treatment that is coming for Ebola?

R2: “Yes”.

M: Please tell me small about the treatment which they say is coming for Ebola?

R2: “Well they told us that it is going to come and they are going to start to give it to people in January”.

M: What are they going to give them?

R2: “The medicine, but since January, I have not seen any medicine except Malaria medicine”.

M: So, what do think in your mind about the medicine you are talking about?

R2: “Well they said the medicine is there but it is not yet time for them to start using it so I still feel that they have tested on people and animals and they have seen that the medicine is still not ok for people”.

M: Yes my sister, have you heard anything about medicine?

R3: “Well, just like my sister said, according to what I am hearing they said they are coming with a vaccine but it is going to be in March, they said they are coming with the vaccine but it is the nurses that will start taking it first, when they take it and they see that they are ok, then they will release it to us because they are eating Ebola money”.

M: What again I want to ask is what do you think in your heart or your mind about that Marklate (=vaccine)?

R4: No. I, like for me, the decision which they took when they said they are going to give it to the nurses first. Right now I have heard that they after taking it and there is no problem there, that is why they want to see how is the medicine going to react and the reaction of the people to the medicine before they bring it to Sierra Leone. That is what I understand”.

M: Yes Mama?

R5: “Well they last gave people Malaria medicines so since then I had sworn that any treatment that comes from government, I will be afraid to take it. The way that Malaria affected people, I am afraid to take it. Especially this Ebola treatment, I do pray and prevent myself but I am afraid to take vaccine for that”.

M: Yes Mama, have you heard any rumour about?

R6: “Just like what my sister said, but I have not got it from any reliable source so I cannot say anything now and I don’t want anybody to ask me where I got my information”.

M: Nobody is going to ask you Ma.

R6: “So like I was saying, but anyway if they say the nurses are the first to take it, if nothings happen to them, me too will take it but that first one, when they take it is what I am waiting for”

M: Yes Ma, add something to that?

R7: “Well to me personally it has taken ten years without taking injection, so like this medicine which they say is killing people is through the prescriptions, because they were not prescribing it properly, some people were not going for treatment, just because of personal influence or personal gains they call them to come and distribute medicine. Some people can just go and give you medicine, mark your house and leave. Most times I cannot be around when I come they tell me about medicines, but have taken that Malaria medicine and I know how it worked on me and when it is working on people they do not give them chance to recover, because it is a medicine if it does not fit the system of the people they can be up to five days and they will just come and take people to the Treatment Centre”.

M: When you say it will work you, what do you mean by that?

R7: “It will makes you feel like vomiting, it makes you feel like a young pregnant woman, that is how it makes you feel, it weakens you and that shows you that it is one of the symptoms so you see, then they do not ask and they confirm, as they say there is a sick person here they will just come and take you away, so you see this have made people to be afraid to take the treatment. Thus is not a lie, the tablet is not everybody who takes it. If the government is listening, let them hear, it is not everybody that is taking the medicine according to prescription”.

M: Yes my sister, have you heard about any new treatment that is coming to cure Ebola?

R8: “No”.

M: So have you also heard about any new way to prevent Ebola?

R8: “No”.

M: What about you??

R1: “Not at all”.

M: Yes my Mama?

R2: “I have never heard, what I have only heard what they have told me, that is just what I know”.

M: Yes Mama has you too heard any new way to prevent Ebola?

R3: “Hmm is just that is lack of communication but even so it is there but we have not heard about it yet”.

M: Yes Mama?

R4: “We have not heard any other way yet”.

M: What about the Marklate (=vaccine)? Have you heard any of any Marklate (=vaccine) that is coming for Ebola?

R4: “No, we have not heard anything yet”.

M: Yes Mama?

R5: “I have one of my people who called me but he is not in town here, he called me and told me that they are about giving Marklate (=vaccine), he pleaded with me to tell my people not to take it”.

M: Did he explain to you why people should not take the Marklate (=vaccine)?

R5: “Just because of this sick that is going about killing people, so he pleaded with me not to take it, he told us that we should not take any Marklate (vaccine) which they bring and we are not taking it”.

M: Yes Mama, can you tell us small about the marklate (vaccine), have you heard ant about any marklate (vaccine) that is coming?

R6: “Well I have not heard it yet but if it comes, because human being have different system, there some people if they take Ebola treatment they will die, there are some people if you give them ordinary Malaria medicine will affect the person and if you did not do test on somebody and then you give the person particular treatment it is bad. So they should not just come with treatment for Malaria, eh I mean Ebola”.

M: Yes Mama, the marklate (=vaccine)?

R7: “No, I have never heard about that”.

M: Yes Mama, have you heard about the Marklate (=vaccine)?

R8: “No I have not heard about it, but like what my sister is saying, the government is so careless just like I am telling you, even these medicine killed many people because some people take it three at a stretch, some take four and that is not the way, and let me tell you one thing according to what we are hearing, maybe you do not even have Malaria in your system and then you drink that medicine it is liable to kill you. So if they do a test maybe it would not happen. But they are giving the medicine that is not telling well”.

M: So, what is the most common point of discussion when people meet to discuss about Ebola?

R1: “Well the rise and fall of the Ebola results, today it will be zero, tomorrow one and the other day is thirty and above and then the other time two. The result does not go according to. Then I also listen to the radio at one time that the Treatment Centre at (- - name of treatment centre in the interview district - -) is only two patients there but the other day I heard that it was twenty six or twenty four, I don’t know if it was the other Treatment Centres”.

M: So what about the treatment and the prevention is there anything that people talk about?

R2: “Preventive measures are going on especially at (- - name of interview community - -) here because of what we have under gone, we have lost relatives, loved ones, so now anything, even if they say to go and wash in the ocean for you not to get Ebola (- - name of interview community - -) will do it”.

M: Ok, so the last thing I would like to ask you is, is there anything specific about Ebola that you think people need to understand it better?

R3: “That can prevent them?”

M: Even if it is prevention, treatment, anything about Ebola that people need to understand better?

R3: “People need to understand that it is not only Ebola that has come to this country. Since I got my senses, this is the only year I have heard that people do not have cholera, there is no cholera is not affecting anybody, any sickness now is Ebola; stomach ache is Ebola, head ache is Ebola, Malaria is Ebola “.

M: So Mama, you too can add small thing to that. What do you think people need to understand better about Ebola?

R4: “Well all I have to say is this, you know that this Ebola has caused pregnant woman to die. Because during the labour, if the person begins to bleed, the nurses will run away because they want to prevent their own life, even though they are preventing their own life but everything in this world is a risk, let us just take the risk, the risk we are taking will end Ebola, let us have peace, let us have freedom”.

M: Ok, yes Mama, please tell me any specific thing that people need to know about this Ebola?

R5: “This Ebola business”.

M: What do people need to understand better about it?

R5: “Well people should understand that the sick is real and is serious and we don’t need to relent until they declare that the country is Ebola free”.

M: Yes Mama, what do you think people need to understand?

R6: “According to what they say when your body is warm and your head ache, it is Ebola. So what I will advice is that when your head aches it is Ebola you go to the hospital”.

M: Yes Mama, what do people need to understand?

R7: “One thing I want people to understand about this Ebola is that, there are some compounds when they quarantine the place or maybe somebody has died there out of Ebola you need to go there to sympathise with them and which of because it is wrong, you should not go there. Because some people when you did not go there he or she makes you the enemy saying that when he or she was bereaved he or she did not come to greet or did not even come to sympathise. But really it is not supposed to be so. Because they don’t want public gathering because maybe when you go there the virus is there and when you go there you pick it up and come and spread it to your family. So really people have to stop that”.

M: So, this good point which you have told us, can you suggest any way which we can tell people?

R7: “Well they can tell them in any way because those who are using the megaphone, they can meet them and tell them that if they have somebody who is sick of Ebola, if somebody did not come to greet you, maybe somebody has died there and when you go to greet you can get the virus there. So please if the person did not come do not vex, do not take it that it is a bad thing after Ebola we can come back as one and make peace among ourselves”.

M: I like that one. Yes Mama, you too add something to that. What do you think specifically people should know about Ebola?

R8: “Well to me what I think people need to understand is that when somebody dies and you touch when you go to wash the body, that is what that killing people more, anybody who washes somebody who has died the chances of survival is less so let”.

M” So, which good way can you suggest to us to tell people about this point you have raised?

R8: Well my own understanding is you have to find people who understand how to pass the message so that they can explain to people house to house so that they can understand. That is just what I am saying. If they do that you can see how they are going to eradicate from Africa”.

M: Mum you too can you tell us small thing?

R1: “Yeah, just like what they said when you are alive if you have the virus the possibility for it to transfer is slim according what we hear from medical people but when the person is dead the possibility ids open so we just have to be careful”.

M: So which good way do you think we should tell people about this point you have raised?

R1: “Well, do not sympathise, if you want to sympathise you call the person on the phone and sympathise with the person or if you are not able to do any communication leave the person after everything, after that twenty one days and you see that nobody is affected there then you can go and visit that person and sympathise with him or her”

M: Ok, any other person has any other point because this is the last question concerning this question?

R2: “Concerning the Ebola?

M: What I am saying, is there anything specific that people should understand about Ebola?

R2: “They should know that Ebola is real so we all have to be careful until we zero for forty two or forty one days”.

M: Ok, I thank you all for patiently going through this interview

Rs: “Ok”.

.
