# Supplementary material for: Development of a set of community-informed Ebola messages for Sierra Leone
Source: PLoS Negl Trop Dis. 2017 Aug 7;11(8):e0005742. doi: 10.1371/journal.pntd.0005742 (PMC5560759; doi:10.1371/journal.pntd.0005742)
Supplement: S1 Appendix — (ZIP) [file pntd.0005742.s001.zip › Ebola messages - FGD and interview transcripts/R2HC Ebola Fieldwork 1/R2HC Ebola F1 FGD-MAOLD-Rural1 V3 CORR.docx]

| CODE | **R2HC Ebola F1 FGD-MAOLD-Rural1 V3 CORR (rural focus group discussion)**  **V2 – 1^st^ March 2015 – Probing added**  **V3 – 11^th^ March 2015 – Correction personal data participants** |
| --- | --- |
| DATE | January 2015 |
| DURATION (minutes) | 70 |
| Collector nr | 6 |
| LANGUAGE INTERVIEW | Krio |
| **TYPE FGD** | Male old |

**PERSONAL DATA PARTICIPANTS**

| Nr | Sex  (*F/ M*) | Age  (*in years*) | Education Level (*e.g. none, Primary, secondary, tertiary*) | Language (*e.g. Mende, Temne, Krio)* | Religion | Job / Employment (*how they earn their living e.g. farmer, teacher, trader*) | Role in community  (*e.g. youth leader*)  ANONYMIZED, ONLY AREA OF ROLE INDICATED |
| --- | --- | --- | --- | --- | --- | --- | --- |
| 1 | M | 57 | Primary | Temne | Muslim | Farmer | *local government* |
| 2 | M | 35 | Tertiary | Temne | Christian | Teacher | ***Community based organisation*** |
| 3 | M | 36 | Tertiary | Temne | Christian | Teacher | ***Community based organisation*** |
| 4 | M | 27 | Secondary | Temne | Muslim | Carpenter | *none* |
| 5 | M | 45 | Tertiary | Temne | Muslim | Teacher | *mosque* |
| 6 | M | 38 | Tertiary | Temne | Muslim | Teacher | *health* |
| 7 | M | 28 | Secondary | Temne | Muslim | Farmer | *youth group* |
| 8 | M | 38 | Tertiary | Temne | Muslim | Teacher | *none* |

**TRANSCRIPT: (M = Moderator, R= respondent, R1= first person responding to a question, DOES NOT correspond to numbering used in Personal Data!)**

M: As a community in what ways Ebola has affected you?

R1:”It has affected us greatly, because one, even the schools which are not open are affected, and most of the problem that is affecting us more, we the people don’t believe the medics again, that has brought up most of the problems, a person will become sick when he or she goes to the hospital is afraid, until now when everybody has developed freedom to go the hospital when they are sick. That is one of the areas, if we the people don’t believe the Doctors the problem will not be solved, but if we the people believed the Doctors the problem will be solved, because when you are sick you will not be afraid to go to the hospital, that is the first problem”.

M: You said you don’t believe the Doctors again, what really happened for loss of confidence?

R1: “Fine, in the first place when the, this sickness (Ebola) broke out, the Doctors, when they come and take anybody alone, they will not come with the person back, you understand? and the spray they sprayed the people (the spraying of chlorine), we discouraged*( a sound mobile phone* ), so that makes that a lot of people here are afraid to go to hospital, but we thank God a lot of people come and they are well sensitized, so when anybody is sick, they will call them and they come and take the people to hospital”.

M: Any other person?

R2: “Yes sir, to my own side, the way the Ebola has affected us, one is through the breakdown of schools, and right now as for me, my uncle has died, who has been taking care of me, through Ebola, and secondly (*a motor bike horn sound*) by now last year schools have opened but till now schools have not yet opened and results are not yet released and then three, there are people who are sick and go to hospital which of course they are not treated but right now they say when someone is sick you should pull and take them to hospital, for me I have become sick and went to the hospital, I sat there for long and ask for medicine, they did not give me, I walked there three times, they did not give me medicine, they said the medicines are in short supply, unless the nurse that begged me, she will look if she has medicine and she will give it to me, this is part that affects more”.

M: yes?

R3: “As for me the way it, Ebola has affected our community, one is education, it makes education lacking, the exams council will not able, because when you check the girl children that are class six, form one and form two has become pregnant, so how long will they give birth and go back to school, so this sick really affected us, secondly some of we the teachers are community teacher, it is from those pupils we are paid, so now if they are all pregnant, how will the teacher live or take care of the home then secondly back most of the people that paid for these pupils get money from the “lumas” (a special market day), but now government have closed transportation, not everybody has a mind to travel, so that all has been affecting this community, let me stop”.

M: Yes any other person?

R4:” Ebola! I always come behind, first place we, the local farmers, we are at the local produce, during the “lumas” (special market day) where they sell the local produce, but now you find out when you want to go to Freetown, they say you have to take pass (permit to travel out of the area/district) and not everybody has right to talk and that patience to go and sit where they give those passes it is happening, then two the movement; they have stopped people from moving, I need to move and get “ lili and natin” (money) to sustain myself, now there is no way to go, now even to send, there is no way to send, now you find out that there is no control in homes, because what I need to provide is not enough, so the children will get out of hands, some turn gamblers, some turn stone crackers, some “okada” (commercial motor bike) riders and some of the girl children get pregnant, because if they go to school you will find out that , if she sees her companion that is not pregnant, she will say no, why will get pregnant at this time when my friend is not, but since the Ebola outbreak they are going here and there as and how they want, they are just getting pregnant”. As my brother just said, we the heads of schools, we the heads of schools are going to get the suffering in the next academic year, because we have lost a lot of teachers and a lot of school pupils, because the number of school pupils in school are thirty and the rest is for school fees, if they don’t have money to pay, is a problem”.

M: Okay, yes sir?

R5: “The other thing is, Ebola has come and has totally changed our culture, because we as Sierra Leoneans we used to visit, when our brothers and parents are sick, we used to go and greet them, but now they say no visiting, we should not visit a sick person, secondly when a person dies, we were used to celebrate - to do the memorial service this and that, but now they say we should touch dead bodies, then the way of burial, we were used when a neighbour or brother dies, we get white “sreatin” (special cloth to cover the corpse) and cover corpse then pray (for) the person, but they have removed that totally in our country, then secondly we were used to the old system, there was no medical, when you get headache the traditional healers are there, they will heal you, when malaria affects you, they will just cut leaves and heal you, but now they say no traditional healers, when you are sick, even if it is malaria, they say you have to report, so they totally change our culture and the way of healing ourselves. Then thirdly, teenage pregnancy is on the increase, our sisters have all got pregnant, about 75%, especially the ones coming sit to the BECE (type of exam), so totally education has begun to drop in our country”.

M: Yes any other person?

R6:”Me, really what brought about the problem in my community is Ebola, and the Ebola was able to wreck in community due to the activities of some Health personnel that come, during the time when the Burial Team comes. When a person is sick they come, we have one house with almost about twenty three people within the same compound that lost their lives in this Ebola outbreak, it happened in a way that all of us were not satisfied with the health personnel, when they came to spray (chlorinate) the people, you know according to health advice given to them that if they have sprayed a particular place, three days, that will be the time when the medicine will work well, before another human being enters, but we now, when they came, they did not advice the people so,( *sound of an iron)* they will just spray you, spray the room and tell you to enter, and so what, I have never seen that sick in this country when within one week or two to three days people will not get massive deaths like this, it is very seldom here in our country. They are doing so, because the advice given to them in the of office, they will not do it, that have made we lost a lot of relationships in this community, because the advice given to them to come and manifest to people they will not advised so, they will just leave the people like that, and the thing is disturbing the people in a way that they will not be able to withstand, we have survivors here, that are within that same house, there was about two able to survive.

The second point again, most of the activities of this Ebola has created grudges in this community, there are grudges right now, the friends of this sick, like the contact tracers has become ?envious?, they have become ?envious?. Now, no sooner you see someone is sick, you raise an issue of a sickness and complain, that particular family will take you as an enemy to them, and in fact most of these contact tracers when they are passing in a particular area, they will abuse them, they say these are the people that “Kongosa” (gossip) are our people to go and kill them, this because there is no credibility in Health practitioners. When they take your person, they will not come with him/her again, we have plenty of cases here until now. We do not have message from the treatment centre that so and so person is alive or has die we don’t have that information, so this has made we have loss great confidence in the health practitioners, we are taking it now that they do not want it done, because if they want it done, any advice you have for the this sickness not to kill a lot of people, give them, they are trying and sometimes they are not trying, because they benefit from it, I stop here for now”.

M: Ok Sir, go ahead

R7: “The other thing again that is affecting about this Ebola, the last time they went with my brother (name of brother), from when they took him along, right now everybody is in doubt whether he is alive or not, we did not get any result from *(voice of the moderator*) the treatment centre where he was taken. The burial teams; the way they handle corpses, it is not really satisfactory, first time they were able to put things in place, when the sickness was fearful, but now they say when someone has died they “lappa” (………..) and trouser, when they come, they will take care”.

M: Yes will come to that, we will discuss it. Yes sir?

R8: ”The other one that is affecting us here, we were having one old woman here, she was the first person that got sick and was taken to Kailahun (first district in Sierra Leone with Ebola cases), they came with her back as survivor but when they checked the certificate, it was fake, a fake certificate because these days when the” EU” (European Union) came to check for survivors, when she took her certificate they checked it, they said it is a fake certificate, that has given cause for all the people to lose confidence in the medics”.

M: The other question is, have any of you personally seen an Ebola patient, I mean the sick person?

R1: “I have not one day seen the person, because they say when a person gets Ebola , the signs and symptoms, frequent stooling, rashes all over the body, red eyes (*someone talking*), hosing (running) blood from the nose, I have not one day/not ever seen it, when I see that, I only remember about death”.

M: Yes sir?

R2: “I have seen it in films, the sick in Congo, the other one is my girl child, and she is a survivor, one of these days they discharged her from the hospital, they came and took her here to (- - name of the main government hospital in the interview district - -) for two weeks, so that, I have personally seen an Ebola patient”.

M: Any other person?

R3: “Yes, I have seen a patient which they said he/she has Ebola and I believe that the sickness exists and is the sickness, because of the symptoms which they always see of somebody that has Ebola, I have seen one, two times”.

R4: “Yes sir, myself I have seen it once, is one boy that we live in the same area, I saw him vomiting frequently when they came to collect him and he was also having frequent stooling, that was my first time to see an Ebola patient”.

R5: “Yes I have seen it, but I don’t have the conclusion that it is Ebola, because technically some of the other sick we have are similar in symptoms to Ebola, like cholera, malaria and dysentery, these are common, so if that includes the symptoms of Ebola, automatically though some symptoms are not complete, you will vomit sometimes - it may be cholera, you will get high body temperature - sometimes it will be malaria, you will vomit and stool blood - it will be dysentery, so we are really confused, so to me I will not conclude if it’s Ebola or another sickness”.

M: Yes brother I saw your hand up?

R6:”Yes, one of my friends, who first died at (- - name of interview village - -) here, he was chewing his tongue, blood was hosing (running as from a tap) from his nostrils, mouth and all parts of his body and ear. I have seen an Ebola patient”.

M: Any other person?

R7: “Yes, I have seen them a lot because I usually visit the hospital (PHU), at (- - Name of an MCHP – mother and child health post, lowest level of PHU, at a village/small town in one of the neighbouring chiefdoms), when they had patient there, have seen a lot, I will do follow ups until they confirm the patient positive. Like in this community we have ever had an Ebola case, like the case my brother highlighted, they took the person, without coming back with the result; if it is positive or negative.

M: What has brought about the spread of Ebola throughout our country, which we are not able to control?

R1:”One, we have to believe that Ebola exists, we are denying”. Then most of these symptoms of Ebola that is showing up are common to other sicknesses here, I want everybody to know that not when ?? somebody are shaving is Ebola, it might be malaria??”.

R2: “Am also throwing again to the medical personnel, neglect and we don’t have any strong medical Doctors in this country, they are not empowering the Doctors in this country, so because of that when sick comes there is no, know how for it, definitely to the time they will get an idea of combating the disease is too late, our medical sector in this country is too dormant, there is no empowerment to the Doctors and Nurses , so that makes when blunders comes up today , that leads to the thousands of people are dying because of lack of proper medical”.

M: Yes sir?

R3: “Myself, if I may start, the government has hands in that a little and also the medical, why I said so, because this sickness came far away from Guinea according to rumour, until it stops at Kailahun (the first district in Sierra Leone with Ebola cases), if Government should have applied this measure of quarantining districts before this time, this sickness shouldn’t have spread. But they were timetabling it, this has led to it spread”.

M: Yes any other person?

R4: “Yes, is lack of sensitization, the messages of Ebola are not meeting the poor people, them the bigger ones are just centralizing it, they are not decentralizing it to we the common people, that has led to the spread of Ebola. Anything that is for the community, either good or bad, let it reach to the poor person/people. But if you people take as you are the bigger ones, when the money comes you people will eat it without giving the poor ones, the poor ones will suffer. The plenty money that was sent to the central people, they was decentralized it, so we don’t had any sensitization because we don’t know, they say don’t touch if the message do not reach us, we will continue to touch because we don’t know, so that makes Ebola to spread”.

R5:” Yes sir, what led to the spread, first time when Ebola came to Sierra Leone like right now, there were not enough treatment centres, that was the number one thing that led to a lot of death of people. Like for us in (- - name of interview district - -) we were not having treatment centre unless you are taken to Kailahun, some of them die on the way to Kailahun (the first district in Sierra Leone with Ebola cases) when been collected from ( - - name of interview district - -) here some of them after they have been taken away, they will never come back with the result either positive or negative. Then laboratories were not available to quickly test and get the result of a person so that they will rush up for treatment. So that is my contribution”.

R6: “May God help us all, this sick, some people take as a way to enrich themselves, while I said so, an incident occurred this behind time (last time, recently) at (- - name of section of the district headquarter town of the interview district - -), when a member of the Burial team , he was having a chemical, only him knows where he got that chemical from, he went to a nearby stream named the “stream by the swamp”, where he poisoned the water well which (water) people use to cook, you know Sierra Leoneans we are too selfish, we only take advantage of situations when our companions are in tears. He tried to put this chemical in the water well, but some guys were in hiding watching, he was having the bag with chemical about to put it in the water well, the guys watching went and asked him, what he is about to do with the chemical, he said, he has been sent to help in putting medicines in water wells, so they said drink the water as we don’t believe you, later he did, so he went home and started grumbling that he is sick, the guy end up to dying, when tested he was Ebola negative, so unless and until we get credible medical personnel people (and) will have the free will to accept any Ebola messages and also take their people to the hospital”.

R7: “Again, when Ebola came to Sierra Leone, the Government and partners say Ebola does not have Medicine, so the people viewed it that a sickness that is without medicines, what is the need to go to the hospital, this is one of the key problems. They will say that they have cured this person, but why the ninety days, they said you should not be with your wife neither your husband, so people will say this is not Ebola but another thing, they will say this sickness is not real, they are just lying. And also politics, when it started in Kailahun, they said APC wants to play games, is lie there is no Ebola, at the end everywhere in Sierra Leone have got Ebola. I will stop here first”.

M: Are there any local terms that people use to describe Ebola?

R1:”Krobola”.

M: What do you mean by that?

R1: “When they “krobola” means “kasara”, “filtina” (trouble, swearword).(l*aughing*) “wooca”.

M: what is “Wooca”?

R1: “Fowl sick (coccidiosis)”.

M: Some people do not believe Ebola exists. Do you know the people in your community? (*Noise by respondent*). Yes

R1: “Now we believe that Ebola exists, but first there was no believe, because the way the medical people were treating us, we were not satisfied. Right now 98% people have believe Ebola exists, because first when Ebola came, Doctors were running away from patients saying don’t touch me, don’t touch me, so the people were afraid, they will say you man leave me alone, there is no Ebola they start telling their companion. But right now 100% because they have seen the way Ebola has killed people”.

M: Yes sir?

R2:”Yes we believe that Ebola exists, especially we in this town, some people have died of Ebola, they have taken away some of our brothers and sisters that they say are positive, they took them for cure and they came back as survivors, so we are totally convince that Ebola is real”.

M: Yes any other person?

R3: “I want to say that Ebola exists and it’s real, first when you meet my man , I will say: “hey guy en kpaii”, you shake hands and embrace. But now, you hardly see that, so even become furious if someone attempts to touch.

M: When Ebola came newly, they were giving out messages to us of what to do, and not to, do you know of anyone or you heard of not so?

R: “yes sir”.

M: Yes we know you heard of it, so we want to know how really do you understand those messages, which one of them you understand better?

R1:”To me the touching of the sick and the dead, Which is of the key things, that when you touch someone that has died and has Ebola, you will also get Ebola”.

M: so that is clear to you?

R1: “Yes sir”.

M: Any other person?

R2; “For me I support my brother 100%, because touching of the dead and washing, when someone has died, you don’t know the cause of death, then start playing and touching, the sickness will transfer to you”.(*noise at the background*).

R3: Yes

M: yes sir?

R3: “What makes us to believe, a lot of family have died of the washing business, when person dies you hide and wash the body and later bury, in the end a lot of people die behind that, that shows that playing with the sick is not fine, so when a person dies avoid the body”.

R4: “Yes sir, I was listening to one radio programme, when they said one Imam died, this Imam learned better Arabic, and some of his followers washed his body and some even rubbed the water that was used on their bodies, the after effect was, a lot of people died, More the ones that used that water”.

M: Which of the messages that will be suitable to tell people, so when they are sick, they will go to treatment centre? (*a long silence*)

R1:”Yes”.

M: Yes sir?

R1:”One, we should be telling them, when a person is sick, you should not keep the person for one to two hours , take the person to holding centre, because the more you keep the person, you don’t know the main problem of that person, maybe it’s Ebola, but if you people are able to “cajole” (talk to convince) the people that when a person is sick, don’t touch, at least it is very difficult to see people that don’t touch a sick person, so you should have trained personnel that will use the protective gear, to be giving first aid treatment to sick person before taking to hospital, maybe it is a common sickness, you should not be watching that person until the person falls and “gbop” (die)”.

M: Yes any other person?

R2: “To me is isolation and early reporting to the hospital. When someone says (he) is not well, you isolate the person immediately, if it is in a family, allow one person to be attending to person, giving ORS, even if is plastic let the person use as protective, until you call the medical team to come and take the person. Then thirdly this issue of washing hands, we should not forget it. This hand washing with soap and water, tell everybody in family that they should wash their hands when they come or go out”.

M: What do you think would be the best channel to get your Ebola messages to the people as how you have suggested to be washing hands?

R1:”Christian and Muslims, they should be passing on this messages in our local dialect in mosques and churches that is one, then in schools, the general Assemblies, God will March when schools reopened we will do it in assemblies, and we will put measures in place at least every thirty minutes the classes would have talk on Ebola, every school will have a fixed timetable with a particular period in their school, every day on Ebola talk”.

M: Yes any other person?

R2: “Then secondly on media, let them pass the message on micro (=……), thank God everybody is using Radio, so you will get messages clear in our local languages not only in English. Let them tell the aired men, let them talk in the local language used by people in a certain community, so that the messages will reach the poor people that do not understand even Krio”.

M: Any other, I saw your hands up?

R3: “The other area again, which I see we will try to fight this thing here is by prostitution, prostitution is another area that “scatter” (=spread) this sick and, I believe if we all can advise ourselves to abstain first and stick to a partner that you trust, I think say, that alone will help us a long way, like most of the time big people, especially we the men take chances, because of this woman is coming closer to me. And we have series of complaints to (=about) that. It happens you will fall in love with somebody which has that sickness, because when the person have the sickness, you don’t, you know, all your aim is just is to score the goal, at the end of the day you continue the sickness, with your family, “mourdor” (=…..) to your family”.

M: family ” mourdor”, is it the other name for Ebola?

R3:” Yes it family ”mourdor”, because when you come with it, not you alone suffers, other people suffer the consequences , so that the prostitution and the affiliations of other relationship, you have to learn that, that again will contain this sickness”.

R4: “Yes sir”.

M: Yes sir?

R4:”To my own side, they say the friend of Ebola is dirty, when sometimes from workshops and meeting, I will go and tell people that Ebola’s “paddy” (=friend) friend is dirty, so everybody should keep their place clean, then secondly they gave us rubber to get, which is veronica’s rubber (= veronica bucket, a bucket with a lid and a tap attached, to get running water) to get water, this is bucket we use to wash our hands, every day I clean, fill it with water and put on the street”.

R5:” We love to tell our people to do it in school or the media, but not everybody are school goers (==not everyone is literate/can understand), so I prefer we dramatize it, so most of the children that are not going to school will watch as they pass by. Because most of the people do not go to school, or mosque neither church but when they see it in drama form they will able to translate that to the understanding of others”.

R6: “The other one, the bye-laws that were put in place must be enforced, because those bye-laws are the only ones that will be able to help us, because anywhere there is law without enforcement, problems will never done (=stop) there, that is the same problem that is (*voices of other respondents*) disturbing ( - - name of a village - -), this village (- - name of the same village - -) unless they quarantine the whole town because of their deniers. These bye-laws are very important, so tell all the authorities to press on those laws that make us to succeed”.

M: In the event of infection, where do you think that the person will go, either to a medicine man (=traditional healer) or Hospital?

*(Respondents speaking in chorus)*

R1:”Hospital, because it is not a medicine man’s sickness, in fact that is why the medical people say when you are sick report, because if the persons goes to traditional healer, the person must be touched”.

M: Okay?

R2: “In hospitals there is care, this was the same thing that happened in ( - - name of the district headquarter town of the interview district - - ), the medicine woman was quarantined because of rubbing medicines. So when you are sick go to the hospital”.

M: Have you ever heard people talking, in either good or bad way, about the Ambulance services?

*(Respondents talking in chorus)*

M: Yes let’s talk one after the other?

R1:”there are a lot of bad ones”.

M: like which ones?

R1:” First when they come to collect people, they make all the people panic, because they will spray the people with chlorine inside the Ambulance and sometimes the person dies before they reach at the hospital, even shelltox (a brand of aerosol insecticide used to spray rooms) when sprayed in your room will disturb you. So if they do not change that system it is a problem for the ambulances”.

R2: “Am talking for the bad side of the ambulance, one is the blowing of siren (“Wae am, Wae am”) where is the person, it will make the people to panic. Secondly if Joe and Brima report sick, sometime Joe has the sick and Brima do not, both of them will be put in the same Ambulance, and this ambulance has been collecting sick people from different places, and they do not wash the ambulance, so if Joe is having Ebola, he will pass it on to Brima. Three, the speed of the ambulance drivers are beyond limit, somebody is sick then run the ambulance at higher speed. They do want know, there concern is to run with high speed”.

M: Any other person?

R3: “Yes my own suggestion is the burial team, the way of handling the corpse”.

M: Okay don’t worry, we will come there I have a whole lot of them let’s do it one after the, the Ambulance first?

R3: “When someone is sick they will call them, they will not come earlier, sometime after two to three days”.

R4: “The ambulance driver do have the respect to treat people, because automatically take you as somebody dead, they do not talk to you, they do not care about, they run will run with you with high speed, before they reach where they are Admitting you, you should have collapsed and die, so they should having respect for people”.

R5: “ Respect, those people they do not have respect for the sick people, their target is that you are done, the last time Ambulance took people from (- - district headquarter town of the interview district - -) to Kailahun (first district in Sierra Leone with Ebola cases), they passed by the highway as they reach at Masiaka (a main road junction) they park and went to the bar, they drink enough, even one of the patient died inside the ambulance, they do not value the lives of people at all, they do not even check for gallops, they just like how they want, they don’t have respect for the sick person at all”.

M: Any other person?

R6: “Yes the good thing back, they are really take care of themselves, because they are the people that easy get the Ebola sick, then the bad thing this ambulance that carry sick people, the driver will take to a stream were a lot of people gather and wash it, so you find out that the problem will transfer back to the people that use the water and it will after them”.

M: so you said the water side were people wash, is the same water the drivers use to wash the ambulance?

R6: “Yes is the same water side they launder”.

M: Any other person? Yes you were talking of the goods side

*(Noise from the respondent)*

R7:”Hmm actually they are preventing themselves not to be affected by this sickness, they dress neat, and actually they prevent themselves from not getting the sick”.

M: Any other good?

R8: Well to me, them the ambulance drivers and any other person with them, I see it like they only cater for themselves only, not the sick persons, because if you are protecting yourself, with all the protective gears at least to safe guard your life , what about the person you are putting inside, at time they load two or three in the ambulance, and among the three there will be one most likely maybe having the sickness, because not all of them will be having the sickness, while they should not also give one or two or in fact all of them the protective gears, so that you also will be protected to touch them, but in case none of them are having it, they will be touching and even communicating in ambulance, maybe this man has Ebola, may God forbid, when I get in contact with him I will get Ebola, so maybe before they reach the others have already contracted the Ebola, so if the person is having PPE as a sick person. The ambulance may be thinking to protect the sick not only the chlorine, because the way am seeing the chlorine how it is destroying people because when it is used in excess inside a room the more you damage the person, that is really making people not to survive. So I believe that not the chlorine that is falling down on people, let them be giving also the protective gears, so it will be uniform, so if I touched you, it will not transfer to me neither you and among the sick again, the virus will not be transfer because you are all protected”.

M: Any other person?

R1:”Then again, I want to commend the military for taking over the ambulance driving, these are the people I want to commend because they are good drivers the military”.

R2: “Yes sir, me to my own side, one upon a time I was going to ( - - name of district headquarter town of the interview district - - ), were I met one ambulance driver, he went carefully and park the at a corner, he was loading a lot of people. They said they were survivors, at that time I meet people insisting to go with them, they said they are from Kailahun (first district with Ebola cases in Sierra Leone, with one of the first treatment centres) going to Kabala (district headquarter town of a district in the North, where there was no treatment centre at that time), he parked the vehicle and went to the market to buy something, at that moment the people were inpatient insisting that they want to go, so carefully went to the driver and talk to him, other people were crowded on the vehicle watching the survivors, some of this survivors were ashamed, so they advise the driver to move the ambulance”.

M: What about the treatment/holding or care centres is there anything bad?

R1: “Those ones are very important, we know, those care/holding centres they helped us. You will find out that not all the sickness is Ebola, when you go there and see your family member you will not be afraid, so if you are there they will test you, if it is not Ebola they will treat you and you will return back home”.

R2: “Yes sir, when they brought these treatment centres, it helps us greatly, because at first there was no treatment centre, when people get sick unless they “pack” them here or take them to Kailahun (first district with Ebola cases in Sierra Leone, with one of the first treatment centres), because there was no treatment centre, so they have said this treatment centre, when you are sick you should go there if even it is malaria or fever sickness, so when you go there, they will talk to you fine, ”bayo bayo”( pamper) you fine, treat you and welcome you come back to your place, so this treatment centres are very important”.

M:”Yes any other person about the good things of treatment centre?

R3:”The good things but the treatment centres as they like say, the distant treatment they are giving people, they will take you far away to Kailahun (first district with Ebola cases in Sierra Leone, with one of the first treatment centres) for treatment, and your family will not see or hear about you. They are telling the people in the community to report and somebody will report earlier at the CDC treatment centre, so they will check you properly, if you have Ebola or not, that helped us”.

M: Yes sir?

R4:”I don’t think that, the establishment of these treatment centre contributed to the lessening of this Ebola, because when a person is sick for example, because when the treatment centre is near that person, they will just take you there and treat you quickly, than when you are sick here, they take you to Kenema (second district in Sierra Leone with Ebola cases) or Kailahun(first district in Sierra Leone with Ebola cases) *( sound of a motor bike*), the distance, maybe the disease is not going to kill that person, the measures, the distance covered from here to Kenema, before they arrive the person is weak, because of the drivers running with high speed, before they reach the person has collapsed; even to take medicine is problem, but the if treatment centre is in my “nostrils” (nearby), nobody will tell me to go the treatment centre, I will go there myself that am not well, when somebody goes there, especially we the contact tracers that are sending people, we always find out how are they handle treatment, when we go there and see, they are really taking great care of the patient, they give them food, they eat three times per day. We were having one patient (name of the patient) we sent her there, she was there and even refuse to leave (the centre) because she said the treatment is good. Somebody people at their home they eat once per day, but the treatment centre three times per day. They talk to her fine”.

M: Yes sir?

R5: “Although I don’t hear the question”.

M: the treatment centre.

R5: “I have caught up, one of these days I heard of a friend, he is over that side, I heard that, they have taken him to the treatment centre, so I met him yesterday and ask “orman”(guy) your body looks good, he said “orman”(guy) I swear to God, is the way they have been taken care of me at the treatment centre, I ate three times a day, motorcar will go out and come in with food, so if you know anybody with that sickness, tell the person to go to the treatment, I said okay; you have done well”.

M: But what about, yes sir..

R6:”Me I will also continue to emphasize on the Government and other medical practitioners, mmm, they contributed immensely to the massive death of people, why I say so, they said prevention is better than cure, if only they would have strategized on this issue from day one when this thing started, because they will not tell us that there is no way to bring treatment centres here, I don’t know if politics is playing or any other thing playing on this game. Where the things started there was treatment centre but where the thing got worst and famous there is no treatment centre, some people bleeding, unless the amount of people they wanted to die has died, that is the time they started coming with treatment centres, they contribute, what you are supposed to do first don’t do it last, it leads to the unexpected”.

M: What about the burial team?

R1:”Mm, the burial team, I must commend this recent once now. Red Cross especially, their interventions help us a lot, if I take a case scenario here in ( - - Name of the interview town - -), we were stoning the burial team, when they come to bury dead body, they will treat it like any dog. The first burial team they prefer let somebody die and their family so that they will be buried, because you will not see Joe buried like a bush animal, I will prefer I do the burial myself, if it is Ebola now then all of us die, if it is not Ebola okay. But thank God now red cross burial team, when they come if it is Muslim death they call the Imam aside and call the family also to give “casangey”(white satin) and they will pray the dead, the family will also escort the burial team to the “bury gron”(cemetery) to bury”.

R2: “the burial team, the first ones, that is why we bless them, first ones the burial team, even this town, that is why there are three boys in prison, there was heavy fight here, because the way they treated a woman, her family was not satisfied, there was fight, between the people, burial team, and the police, they even brought deltas (=…….) here in this village”.

R3:”Yes sir, my own contribution towards the burial team, when my Aunty died here (name of the aunt) , when they came they met they have already dug the grave to bury, when they moved from ( - - Name of district headquarter town of the interview district - -), as they reach, enter in the room directly and undress the woman because she was dress up, they removed her dresses and put her in the plastic, they took her like animal, they did not treat her like mortal man, they begged them for the woman to be buried here, but they put her in the ambulance and took her to ( - - Name of district headquarter town of the interview district - -). Then secondly, first time when they come here, they said a person should not follow them where they bury, so it happens some boys fall victim in this type of programme, when they went at the burial place, one police/soldier man was highly drunk, so he took the gun and hit the boy, the boy also resist, so the boy take back and get out of the cemetery, when they return they start quarrelling. Because when they go for burial, they will not even cover the grave with soil properly, they will pull the PPE and leave them exposed without even covering them, so it will remain and they will return”.

M: Where they not covering?

R3: “Uhmm, they were not covering the dirt, they just pull the PPEs and put them with the dead person so with all that”.

R4: “When this sickness came newly, actually the burial team was not having respect for the dead, because when someone dies, it happens this sickness, when a person dies is like a magnet, it will attract, even when they put them in the body bags, if you don’t want to cry you will cry, because incident like this will create confusion between burial team and the community people, because when a person has died, though gone, but the person receives respect, but the way of handling is like throwing the dog in a dust bin, so actually, I don’t feel happy. Except this last one that was introduced by Red Cross, when you call them, they will call the Imam, he will stand far off and pray the person or sometimes the Imam will be at the mosque and the dead body at the house and then pray for the person. And they will also ask for “lappa”(cloth), first time when a person dies they will bury you naked. Actually one thing happened in ( - - name of interview village - -) , which I was not happy for them, woman, we have to respect woman as God says, but that day the woman die with cloth, but she was off loaded that day naked as how she was born and was pushed into the plastic. If you are saying, this sickness transfer, the cloth of that women was removed, you will not get the sickness? You know it is very disadvantageous, so we don’t feel happy about them”.

M: Yes sir

R5: “Me to me, is like some guys that employ in this burial teams, I will always reflect my mind (=think back to) during the war, is like some of them maybe they are there before, if they respect somebody that is alive or who has love for their own, I believe despite the money they are finding, but they should have great love ,care and respect for the person that have die, but is like they are taking advantage or they don’t have sympathy, so I see them, because not only the advice they have given them I don’t think it is appropriate, that when you come take dead bodies swing it in vehicle like goat. So for me, the advice I want them to give the burial team, the ones enrolling them, should know the type of people they should put in the burial team, because if you go and take someone that has been used to killing mortal man or having no respect, “to God”, this thing will continue more. The other incidence that was happening here, people were poisoning water wells, most of this people were the people we see wearing PPEs, at night now people guard their water wells and houses, honestly people were going round to poison water well so when someone drink the poisoned water, they will get frequent stooling, vomits and die, at the end of the day they will go and lie on the people that is Ebola, for this thing not to end. And most of this act is done by some of the members of the burial team, there is an evidence at (- - Name of section of the district headquarter town of the interview district), they caught him poisoning the water, when they ask him, he said he was sent. So they ask him which burial team he worked in. He said he worked for so and so burial team. So definitely, these are people they don’t have sympathy and they don’t want this thing to end, so okay”.

M: Okay do you have any survivors?

*(noise from the respondent)*

R1:” Yes we have, right now I have one with me at home”.

M: Yes what about you sir?

R2: “I have two”.

M: Okay?

R3:” yes they are there, but according how we started this discussion, one woman was sick, they took her to Kailahun (first district in Sierra Leone with Ebola cases), later when she return with her certificate has survivor later they found out it was a fake certificate”.

M: How do people treat these survivors in your community?

R1: “We, here we don’t have any problem like my child is free to walk up and down in the town”.

R2; “According to the survivors here, we welcome them fine and some are working in the holding and treatment centre and they are treating them fine”.

M: Have you heard about treatment for this Ebola?

R1: “Yes I was listening to BBC (radio) last week that flight just landed in Liberia that they have prepare one vaccine, so the whole of January they are testing it in Liberia, February and March they will go to Guinea and test the vaccines”.

M: Yes sir?

R2: “Am hearing of Zmapp (experimental medicine for Ebola) which cures Ebola, but we have not yet seen it in our country”.

R3: “Yes sir, like for me, although I have not seen, but the other medicine they say that cures Ebola is chlorine, they say it is the only medicine that cures Ebola”.

R4: “I have also heard of it in America, it is used on most of the patient taken out of the country, they said if it is properly taking care of it (=Ebola), it will reach us here in Sierra Leone”.

R5:”That medicine even the nurse who was sick of Ebola, has been used, and the nurse has discharged”.

M: Okay, have you heard or find out of any new ways to prevent Ebola?

R1: yes sir, according to the medical personnel, the prevention of this Ebola…………??………………, secondly soap and water, number three be ready to clean your environment you live always”.

R3”(:………………………??………………………………………………)when you are travelling at the extreme end of Freetown (capital city) use long sleeve to avoid the touch and touch, then always wash your …hands?…., because this Ebola is from dirt.”

R4:” I want the government to train more doctors, because if we can find out, a lot of damage that happened was with this medical people, because they were not having the know-how, if they train now , thank God we have Labs (=laboratories), but if it is from the time this thing occurred in Congo, it was the time they produced the medicine, we should have not got it here, unless it came to West Africa and caused problem, now it is in Sierra Leone. We are praying to God to get the medicines, that is what I have said to train more doctors for this sickness.

R5:” What I see to teach people more about hygiene and personal sanitation, because if you look at the symptoms of this Ebola, it is about personal hygiene and sanitation, but if for example, there is a town without toilet, all us go to bushes and use them as toilet, it will be very easy for us to get the symptoms of Ebola, which is (actually) cholera. And it happens again there is no water well, all of us will go to the bush and get a water that is not pure and not covered. So we are recommending to the government, for the building of more water wells in the villages, at that time, it will be little safe from Ebola, then also the building of toilets, let them build toilet for us, especially in the rural sector, those villages. I travelled to one village where I do teach (- - name of the village - -), most of the people, when they wake in the morning, they don’t have toilet at their back yards, they go to the bush, one day I ask one guy where is the toilet, he said to me, just stand and wait until you shit on yourself, just go up that hill, and I went there. In that village when cholera breakout eight people died, so just imagine if it was the time for Ebola, it would have been a big problem. So let government focus on that”.

R6:” like one of the new way to combat Ebola, is about the commercial vehicle, let them give the disinfectants to the drivers, so when they travelled to Freetown (capital city) or Kenema (district and district headquarter town in the east of Sierra Leone), when they return, let them disinfect the vehicle before it is loaded again for another trip, because within this cause, people may be sweating, you don’t know if that person is Ebola positive or negative, so government to find ways to be spraying this vehicle with chlorinated water, that is my suggestion”.

M: what are the most common point of discussion about Ebola?

(*voice of the respondent)*

R1”One is planning, how you plan with your family to avoid Ebola, the way of planning, you wash your hands before you enter inside the house, you want to eat - you wash your hands with soap and water, you want to go the market after you wash your hands. You have to plan, when a person is sick, move the sick person to treatment. You have to plan with your family”.

M:Any other person?

R2: “That man has touched a lot of the points”.

M: This is our last question, is there anything specific about Ebola that you think people may like to know?

*(Long silence)*

R1: “yeah, the particular I think people should know about this Ebola, one you have to be aware, we will able to help or not able to help

R1:” they need to talk about it every day in radio’s, as my brother just said, let them be sensitizing people always in our local languages “what is Ebola” which my grandmother in the last village will understand, they will not just sit down and big English, so the poor person in the village can understand. That is most of the reason people in the village do not believe, because the message is not reaching them”.

M: Yes my man?

R2:” if we were living in an advanced country, I don’t think this sick will continue to wreck, yes it fine, the media has played a great role in this Ebola fight, is just because our country is poor, it will be fine when somebody sees someone if not physically but through a television, at least they will know, like the drama my brother was just talking about, in a drama, to see somebody that is well contact the disease, how the disease affected the person, and after which, what are the ways they took to cure that person, with this messages, at least we have actor and actresses in this country, not only the singing, everybody is singing “Ebola go”, these are not necessary, it is necessary in some other quarters but not all the time. Let’s take another step again, like in the Christianity, people were playing film for people to know the activities of Jesus Christ, telling people how they kill Christ that is why I said our country is poor, because countries that are advanced they will just broadcast through television, they show it there; everybody will see, but look here, how many of us have television, let’s say now Electricity light. It will be good for us to have light, but if there is no light, it’s not good for people in the village. I believe that if we all get all the basic necessities of life, this problem will have reduced, look at advanced countries they had Ebola, but it did not take long, like Nigeria, within two weeks’ time everything was finished. They live to that standard way that they may be able to take care of certain situation. Our leaders are selfish”.

M: Yes any other person?

R3:”Mm, we Sierra Leoneans we have to change our attitude, that will help us, we don’t love ourselves, that is all what created this problem. Because even this man that is talking of television, you can’t play television like that. That is just telling you some people take this sickness as business, like how you were talking, not so? They are taking money more important than our lives. We don’t need to make difference, but if we all have said “we are one”, this sick will “done” (=stop), but other man is working so and the other one is working over there. The burial teams, the way they spray people, am afraid, you want to spray a person you don’t protect yourself, is that person will be protected”.

R4: “so what I need, what the man was saying, this money, money they have put in this business, some when they come with the money, they themselves know where they are taking the money to. They will not come to the people and say so and so we want to do to cure this sick. They are just sitting in one place eating the money that is the reason we still have Ebola here”.

M: Any other person?

R5:”for me the plenty of worker they take, the contact tracers now, nurse and doctors now, if they don’t try to reduce their allowance or to reduce them (= reduce their number), Ebola will not end, because, if they pay me five hundred thousand Leones (= Feb 2015: 114 USD) per week then I may not want Ebola to end no.”

M: that government is looking into it now, thank you very much

**ADDITIONAL PART OF INTERVIEW, OBTAINED BY COLLECTOR 1 AFTER CONSENT IN PERSON, February 2015, with 2 of the FGD participants:**

M: Why the spraying of chlorine discourages the people?

R1: “First of all the during the outbreak of this Ebola virus disease in this country, especially in this community, according to my observation, I observed that the content of the chlorine was hazardous, so it was affecting people very seriously, that was why people did bought the idea of spraying the chlorine because its choked and according to their own understanding it disturbs their lungs, that was the reason people were not allowing again for the chlorine to be sprayed when they have a confirmed or suspected cases in their houses”.

M: Why did they use the word Panic?

R1: “An incident occurred here, in a home when eighteen to twenty-three died in a same household, at first when people got sick, when they came to spray, they will not spray the dwelling place alone, but the entire house and they were not sensitizing people not to enter into the sprayed house. They were telling the people to use that house at the very moment and the chlorine was distracting the lungs of the people because it was very hazardous and was causing threat to the people and it was later noticed that the chlorine has an effect to the death of the other family members that was the reason they were not allowing to spraying of chorine again”.

M: Ok, that was what you referred to that it gives panic?

R1: “yes”.

M: How has the Ebola led to more pregnancy?

R1: “Well eh”.

M: What about no touching?

R1: “Well specifically, they told us not to touch, but the outbreak of this Ebola led to shutdowns of schools and schools are one of the areas we sensitized especially the young girls to understand that pregnancy at this stage is not good for them but with outbreak of this Ebola, there is no schooling, no other activity, no marketing like regular time is not available. All of these led the children to go astray, they told them not touch, but they did not listen and adhere to advice of the medical practitioners that led a lot of girl child to take up pregnancy”.

M: Ok, have you heard of a person who had gone to the traditional healer to be cured for Ebola?

R1: “I have not got that yet with my ear, but I made to understood that people are still going to the traditional healer for healing from other diseases”.

M: Ok, disease like what?

R1: “Like how a person is been “fankay”.

M: What is the meaning of Fankay?

R1: “Well it is a traditional witch gun which is used to destroy a person’s life, they knew the means how a person is being affected and how the person is also cured, but I have understood that people are still going to the traditional healers for healing. I had a cousin who dreamt people running behind him with sticks and they throw sand on his back, when he woke up, he started scratching his back, so they took him to the traditional healer later he gained normalcy”.

M: He got cured?

R: “Yes he got cured, people still believed the traditional healers and they are still going there”.

M: The activities of health workers are racked by Ebola, why do you think this is?

R1: “Well this happened at times, most times some health practitioners that works in the community, they do not sensitized the people understand the effects of Ebola rather when they come, they just do it in a haphazard way, which at the end of the day, people can’t able understand, and all the people are not educated. For somebody to understand, you need to explained to the person in way that the person gets the understanding of what you are saying, like before now when HIV broke out, people got to believe that HIV exist because they developed some movies and played in the communities, because they have thoughts, seeing is believing, if they don’t see what is happening they wouldn’t believe. Even in the issue of Ebola, people got to believe at this recent time because when they saw the death of many people that is the time they accepted that Ebola exist, so these are some of the things”.

M: What do you think are some of the activities of health workers that racked the community?

R2: “One, they are not giving good advice to people, then the other health workers in the community are afraid to contact the people like in the area of the PHU during the break out of this Ebola, when a person goes there for treatment, they will not treat you they were afraid, they said they were afraid of their lives, because they do not know the protective measures to protect themselves and even the patients that has reported ,so because of the threat and when Ebola broke out newly, there were no better idea to protect the disease, so this gave the continuity of the sick to spread in the community, so the these are some of the things that led to the racked of certain community”

M: How are assessing the health workers now?

R: “We thought they have acquainted with the system, now they can talk to the understanding of the people, the contact tracers they are another community stakeholders which has the knowledge of Ebola, they are helping them now to talk to people that Ebola is real, I thoughts these has been the situation”.

M: They said the health personnel are not working well, because they are benefiting from it, what is the meaning of that, what is the benefits they are getting?

R: “The benefits that we tagged to them, is the payment that is given to them and you know at the end of the day they are receiving salaries for their work, like before we were arguing with them, because when a person is dead, they did not treat corpse in any good way, so there was that argument, we were never in good terms with them, really it was not easy for them, some of them we used abusive languages on them. It was only in these recent times we made to understood that they were not having the knowledge before, only now, they have the technics to handle it”.

M: What is the benefits that people thinks the health workers received?

R: “Like Their salaries”.

M: the salaries?

R: “Yes, the salaries they paid them”.

M: So they feel that they are well paid?

R: “Yes they are well paid, that is the major issues, sometimes people usually say, because they are paying well the is why you people are treating us like how you feel that is why you don’t want Ebola to done (=to finish), these are some of the things”.

M: People were raising concerns of given the survivors fake certificate, can you throw light on that?

R: “I heard that through by rumours but I have not seen the fake certificate, because the two survivors that I have seen in this community, their certificates that I have seen, I believed they are authentic, but I heard it through by rumours that there are fake certificates but I have not seen them”.

M: Ok, they said the bigger ones are centralizing everything, the Ebola messages are not reaching the poor people. What are they really trying to talk about?

R: “Yeah, that is true, most of the time when Ebola meetings are called in a community, most of the times, they only called stakeholders, the youths and other class of people in the community are left out, and when they come, they did not have the time to pass it on to others, but if the youths were involved. They will move around to pass the information to other people, only recently when they have trained contact tracers, they helped now in passing on the information”.

M: “What is the meaning of these words “kasarah, fitna, wouka” Kasarah what do they mean?

R: “Kasarah means it is finally done, it is done, when a disease broke out and kill almost everybody that is what we referred to in Temne. That is has come to “karasah” (finished) us”.

M: What do you mean by Karasah?

R: “It means to come and kill”.

M: To come and kill?

R: “Yes”.

M: What do you mean by “Fitna”?

R: “It is a outbreak of disease like Ebola, it may just come, this is because we did not expect it and we did not learned it from our four fathers, a disease that just broke out in a community that is what we called “Fitna”.

M: What about “kawouka”?

R: “Wouka”, it was there before, it is just like when a fowl is sick of, I don’t know how they called it, this is how our fore parents (=fore fathers) got sick, they will just sit down, starts hosing out water from the mouth and vomiting with blood, those petty things but now it is not happening, so as the outbreak of this Ebola, they want to related it to the “wouka” that has been happening before, because it has some similar characters like the vomiting, hosing out water from the mouth, all of these things, so they “wouka” means a disease that affects human being”.

M: What about “mordor”?

R: “This “Mordor” means when something is being bundled, like for instance, when a whole community is bundled and put into a single place, they gave the name(name of the community) is like they have “mordor”(bundled) us, they have given us one “mordor” (bundle). So if they said we are all bad in (name of community), it means they have “mordor” (bundled) us. So in the case of this Ebola, they have taken it has they came to “mordor” (bundled us), this is a “mordor” (bundled) that came and that has touched everybody and until God prevents you or you prevent yourself”.

M: What do they mean by delta?

R: “The deltas people that may not understands, they are the police officer”.

M: Ok?

R: “They are the police”.

M: What about the army men”

R: “They are the soldiers”.

M: What is the linked between the guys that are employed in the burial teams and the war? (*Motor bike passing*)

R: “Well to me, the link they have is with the government, the medical area of the government, they have the mandate to pass on information to other people”.

M: What is the linked between the guys that are employed in the burial teams and the war?

R: “What we were trying to say, is that when the burial teams do comes for corpse, they don’t talk to anybody, they will just collect the corpse and buried without the approval of the deceased family, so that is what we linked with during the rebel war, when the rebels entered into any village, they don’t want to know the consent of people, they just do what they feel like, so that is the linkage”.

M: How do you see the guys they are employed in the burial teams?

R: “What I observed, is like it is featuring “.

M: What do you mean by featuring?

R: “Featuring is just like a connection that is exist there, you can called a person who know and trained the person to be part of the burial teams, if could observed, most of the communities I have not seen a person that is part of the burial teams. In the big towns they will just picked themselves, their relatives, then trained them to be part of the burial team”. (*A vehicle passing*)

M: Have you heard of any secret burial?

R: “Yes, I have heard about it”.

M: Can you give me some examples?

R: “One example happened in (- - name of town - -) when a child died in one quarantined home, they kept the child, when they took the child for burials, the secret came out that they had the dead child and kept for three days at the quarantined home, later when this happened, on their way rushing to bury the child, they were captured, so they I understood that secret burials are going on”.

M: What is the reason of getting secret burials?

R: “They are just viewing as, the way they would want to administer the last blessing to their relatives that died, it is not the way the burial teams gives to their relatives, like for me, they will washed the person, dressed the person up before burials, they will used spray (=deodorant) and bathing soap. But now when a person dies the burial teas has the mandate to bury, they will not wash, at times they will spray, the corpse is not even wrapped, these are some of the things that led the people to carry on secret burials and they are not adhering to the medical laws, this was the problem”.

M: If you are an Ebola survivor, what do you think is the problem you encountered?

R: “A lots of problems”.

M: Like what?

R: “Number one, I have to be side lined in the community, they would not come nearer to me, they will touched me, people will go away from me, even to eat together they will not allowed, at times they will gives me a name, at times they will say “taabunnekor yaa”.

M: What do you mean “Taabunnekor”?

R: “Don’t touch, they will still have in mind that you have the disease, at times, your wife and families will go away from you, they wouldn’t the believed that you have cured” .

M: What do you think of the way people react to Ebola survivors when they returned to the community?

R: “Welll…, they are not treating them well, like how I just said, people go away from them, even to talk to some of them, there will no common talks and interaction again, people will be afraid of you, is like they stigmatized you, they will oooh this person is an Ebola survivor, they will still think you have the disease. Even though you have issue the certificate of survival, they will not believe you are freed from Ebola”.

M: Ok, I thank you very much for answering these questions, I thank you sir

R: “Yeah, thank you.
