# Supplementary material for: Development of a set of community-informed Ebola messages for Sierra Leone
Source: PLoS Negl Trop Dis. 2017 Aug 7;11(8):e0005742. doi: 10.1371/journal.pntd.0005742 (PMC5560759; doi:10.1371/journal.pntd.0005742)
Supplement: S1 Appendix — (ZIP) [file pntd.0005742.s001.zip › Ebola messages - FGD and interview transcripts/R2HC Ebola Fieldwork 1/R2HC Ebola F1 FGD-MAOLD-Urban2 V2 ADD PROBE.docx]

| CODE | **R2HC Ebola F1 FGD-MAOLD-Urban2 V2 ADD PROBE (urban focus group discussion)**  **V2 – 11^th^ March 2015 – ADD PROBE and correction personal data** |
| --- | --- |
| DATE | February 2015 |
| DURATION (minutes) | 70 |
| Collector nr | 6 |
| LANGUAGE INTERVIEW | Krio |
| **TYPE FGD** | Older Males |

**PERSONAL DATA PARTICIPANTS**

| Nr | Sex  (*F/ M*) | Age  (*in years*) | Education Level (*e.g. none, Primary, secondary, tertiary*) | Language (*e.g. Mende, Temne, Krio)* | Religion | Job / Employment (*how they earn their living e.g. farmer, teacher, trader*) | Role in community  (*e.g. youth leader*)  ANONYMIZED, ONLY AREA OF ROLE INDICATED |
| --- | --- | --- | --- | --- | --- | --- | --- |
| 1 | M | 35 | Tertiary | Temne | Muslim | Teacher | Local government |
| 2 | M | 26 | Tertiary | Temne | Muslim | Teacher | None |
| 3 | M | 35 | Tertiary | Temne | Muslim | Teacher | Youth |
| 4 | M | 39 | Tertiary | Temne | Muslim | Teacher | None |
| 5 | M | 40 | None | Limba | Christian | Trader | None |
| 6 | M | 36 | Primary | Soso | Muslim | Welder | None |

**TRANSCRIPT: (M = Moderator, R= respondent, R1= first person responding to a question, DOES NOT correspond to numbering used in Personal Data!)**

M: As a community, how has this Ebola affected you?

R1: “It has affected us a lot, as a community because of this Ebola we don’t have anything to do again, we are idle. Even our children no longer go to school and even the income, some of the things we were doing has reduced drastically so those are few of the effects I can remember for now”.

M: Any other person?

R2: “Yes, Ebola has affected us to a great degree as a community to an extent that we have huge number of deaths in the community who has served as respectable people in the society and till date there is no school and the Radio Emergency Teaching and not everybody that has access to this radio which cannot compare our educational system to that of the external exam which we have stopped now education is going the drains, that is the second factor, the third factor is that we have observed even in this community that there are a lot of things which we are unable to put in place, is not everything that we have in this community, even message about the sensitization not everybody has access to that and that has also affected us. So the last but not the least at the time when schools will be reopening, we are going to find it difficult because our children are no longer attending and a lot of teenage pregnancy has affected the community, as it is now we have up to fifteen children that has got pregnant and these school going children who have become pregnant and up till now we don’t even know their faith and destination or where to start their education. So, as a result that is why we are saying that Ebola came to fight us and up till now we are not able to get up to 0% and this has traumatize the entire community and we are also conscious about the zero percent, when we get zero percent now.”

M: Yes Sir?

R3: “Yeah, well if I could add to what my brother has just said, the disturbance which has caused in this community and it is rising Nationally.”.

M: No, you have to come step by step.

R3: “Oh from the community?”

M: Yes from the community.

R3: “Even the market women they can cease their own time of business, they can arrest them at night, they can ask for bribe and can even we the costumers who goes to buy do strain at times, if you want to buy you go as if you going to thief, and they open a small hole where you can buy from, all this is how Ebola has affected us”.

M: Yes Sir?

R4: “One thing which Ebola has done in this community is that it made us loose some respectable people in the community who were developing this community and this Sunday again some people are it normal that they go to the street to buy food but on Sundays they do not sell and that is a problem. Again the teaching, the teachers are just sitting down idly, there is no work; there is nothing to do. In the morning they will just sit down, even the Ataya Base (=(*note: preferred meeting place of young people, where they drink a strong highly sugared green tea that comes in a box labelled “Gunpowder.”, but is also called Ataya*) does not function; it is a great effect to the community where we are now. Even the reopening of schools, even if schools have to reopen the school materials have all gone. Even the children, whom they would come teach - many are pregnant and have problems, some have died, some do not have mothers; some do not have fathers in the community because the people who used to pay for them have died. Like one community, there is one house around here only one child remained all the others have died. It is a great problem to the community”.

M: Why do you think Ebola has spread all over Sierra Leone?

R5: “I think Ebola spread all over Sierra Leone because of this movement of people, some people might have got Ebola the person will move for him or her to be cured in another area to be cured traditionally, it is spreading traditionally more. And even the vehicles when they are moving they take passengers whose status they do not know and bring them into the other community when they bring them into the other community you cannot deny them and that is how it spread more and more. Even one of our brothers he moved from (- - name of headquarter town of another district - -), he brought it and everybody in the community got it because all the family died that is why Ebola has spread all over the country. Also the traditional healers and even the medical advise people are not taking it, when they say don’t touch, some people go about to touch and they get it by the time they go to hospital they are almost dead”.

M: Yes Sir?

R6: “Really the ..?.. if Ebola is caused by a lot of factors”.

M: Like?

R6: “Like, the first one is denial, denial in the sense when the sickness first came people had little or no knowledge about the sickness so at the end of the day there was denial people were saying that how can there be a sickness without medicine? When you get it you are going to die, so at the end the day people were denying the existence of the sickness. Then two in addition to what my brother said, the movement, that migration, the movement of people from one place to another is also a cause for Ebola to spread. Then again it spread out of ignorance some people even when they have told them that if you touch somebody you will get the sick they still take it that because of tradition and other cultural belief, they say we have been greeting for so long we have never got this kind of sickness here we don’t know where this kind of thing has come from. That again can help the spread of Ebola. And again if you look at these survivors who are coming out, they are the other threat”.

M: Eh, we are coming to that, any other person?

R1: “What I can say about the spread of Ebola, the fact that one, it is the negative attitude of both individuals citizens as a result of traditional healers, the negative attitude of the individual Sierra Leoneans too has caused the soar of Ebola. One the medical practitioners have told us “Don’t Touch”, “Avoid Body Contact” and don’t wash dead bodies and this sensitization has gone right across the country but there are still some people who are going to bury people and they will lie to us that the person has just taken bath and was about to go to church then dropped dead so we have washed and buried it. All these are causing the spread of Ebola. This negative attitude has caused the disease to spread all over. Now you know that thing migrated from Kailahun (=first district in Sierra Leone with Ebola cases) and now it has come here to (- - name of interview district - -), it is due to this transmission. When one is sick instead of going to the hospital, they will take it that their uncle is a herbalist let us go to him and he will take there too, by the time they notice it, it has caused damage it could have reached an alarming rate it might have killed four or five people. So the denial and the negative attitude of people is what that has led to the spread of this thing. To do what they tell them they won’t adhere to the preventive measures and if we cannot take this we will be not be able to contain it”.

M: Ant other person?

R2: “Just like what my brothers have just said, we have what we call denial, I mean trials because denial and trials is different thing. The trails, like me now if I am a Traditional Healer and somebody is sick, I will tell the person that I have a medicine and will go and get the medicine all that is trial, and then these trails have turned into failures. We in this country we never knew what is Ebola and we never know what is the treatment. The person who discovered, the first scientist who discovered this Ebola is Claracks James Peters who we never know about. Until the sickness reached here. But people when the sickness came everybody brought out their tentacles say they have medicine, me I know leaf, so those trials which they did they have failed so it continued to spread this disease and it moved drastically because when this one tried and failed. These trials have failed and the sick is just spreading and spread until it has reached a peak and spread all over and became uncontrollable”.

M: Yes my brother? Well in this community, do you have any local name for Ebola?

R3: “I don’t know of any one”.

M: ok,

R4: “People usually say “Ancholera” that it is Cholera that is why they are getting frequent stool and that it is not any other sicknesses.

M: That it is not Ebola it is cholera not so?

R5: “Yes that is their perception; a sickness without medicine now is all about Ebola”.

M: Are there people in this community who still do not believe that Ebola is real?

R6: “Yes there are, though we cannot just call names “.

Rs: “There are many of them, there are,

R: “Ebola”.

M: Continue Sir?

R5: “Even the Ebola some people are still denying, when you tell them don’t touch one of my brother who is denying that Ebola is not real; said that if anybody comes to him he will stab the person when he said he will stab anybody who comes to him his wife was found wanting of Ebola, not too long he too fell sick and went and there are people who are still denying. In this community even though they have seen the fatal way Ebola has spread in the area”.

M: Any other person? Ok, you have been hearing different messages on Ebola, which of the messages that you really understand that is really clear and which you believe that people should abide to?

R6: “Well as far as this preventive measure is concerned the first messages that came to us did not come down well”.

M: Ok.

R6: “Well, the first information, even the sensitization did not go down well we didn’t know the right precautionary measures that we should put in place. It was there and then that we got the information that Ebola can kill, but at the initial stage they said it is a killer but now you go late or you go early you are going to die. There is also what we call AWOLE, or let us say escapees to see whether (….not clear…..), so that is what is making people fear because they thought they were going to die. But when now we are following the medical precautionary measures we are now getting encouraging results as compared to before”.

M: Any other person?

R1: “As for me I will only educate the person that Ebola is real”.

M: Among all the messages which one that is clear to you?

R2: “Really, a lot of messages were coming some of which we were getting on the radio like on that popular program called ‘Monologue’ and we have one other one which the political artists sang. But the one which really touch me, the one which Obama sent, when he said it is not when you sit in a motor car with a person who has Ebola then you are going to transfer to you, then he again said that is not when you exchange hands with Ebola that means that you will get Ebola, he we should not wash dead body and we should not touch the sick, if you abide to those two key things the possibility for you to get Ebola is very slim, is not that you cannot get Ebola but you will be very safe. So there was where I got the whole thing”.

M: Yes Sir?

R3: “Well, is almost the same like, what my brother has just said the messages that were coming in we accepted it because it was real. and even the sexual intercourse, if you were doing it you have to stop the sexual intercourse and even the contact, the naked body so we have to stop those things then even to accept somebody who comes from where you don’t know, you have to ignore that person and shorn him because you don’t know where he or she is coming from”.

M: Yes Sir?

R4: “Well to accept somebody just like that, but some people will say the person is his relative and that they have nowhere to throw the person if they are going to die let both of them die.”

M: Then among all these messages which one, do you think, is best for people to go for early treatment?

R5: “Well for me is to just tell people that a lot of people has got this Ebola and they died. But those who went to hospital quickly they got the correct treatment they were able to survive. So we can tell people like that that people have got the sick they went and survived so let them too get the belief that they too can make it”.

M: I said among the messages which one do you think will be the good one which will encourage people with Ebola to go for treatment?

R6: “Ok, well we have got the message on hand washing, that we should wash our hands with soap and water or with chlorine, water or with Dettol, after. Then we also look at the one which says ‘Report all early cases, early symptoms, anything that you feel, your head, or stomach ache you have to report earlier so that treatment can diagnosed and cure it before it escalated to a point. We know that the sick people who went early were well and became survivor. Then the touching of dead body, you have to abstain from washing dead body, you that and do not touch the sick if we abide by that I know is a good message for all that. Then, especially in respect of the Holding Centre, the Treatment Centre, Laboratory equipments and the economic boost”.

M: Any other person?

R6: “Yes. I just want to add to what my brother said. This message of, my own whole concept about this is that, the ..?wrote?... man said “Prevention is better than cure”, before I contact the disease, I prefer to do all what I should do for me not to get it, except otherwise, if I get it otherwise then I will go for early treatment, that is what you should do, any symptoms you get, I think that can help you”.

M: Yes?

R1: “Even this message about this early treatment. We are in community with one man when they told him to, wash hands with soap and chlorine, the man did not know better about the chlorine he just came the chlorine inside the water and washed the entire body because he didn’t know. Later before going to the hospital his entire body had peeled off and later he got well and came back, so he is now an example, even if his child part here he will put the child into the motor car and take you to hospital because he has seen an old person who has died of this Ebola because if this saga which fell in our community. Even in the community he is now ready, anywhere to act”

M: Then, all these messages we are talking about, which channel do you think is the best that we can use to get on to the community members?

R2: “Through sensitization, the radio, we have heard about this IRN, and the sensitization which has gone all across the country. And what I see as at now at community level cannot see a man sick of Ebola but I believe that with the help of the thermometer has a great role to play it can detect a high temperature even though the person might not have shown signs and symptoms which can tell what is wrong. So that is one of thing that should be there even after post Ebola in abundance for everyone to know his normal temperature then the person is sick and goes to hospital they have the big machine which they use to test your temperature to give you the right treatment, likewise we are also suggesting that even the schools need to get them”.

M: Any other person?

R3: Yes, if I can recap, I just want to say this one is the most appropriate channel you are using. Really we had a lot which we have gone through like seminaries, think about music, the radio, and make jingle, and a lot more. But for me the most appropriate that I have seen is let us look at a particular artist who is popular and being him into the community whatever he comes for, he should first talk about Ebola, during the process when he is performing he can talk about Ebola, I think that is most effective way that. But the radio, is not everybody that has radio and as for the Television it is the worst”.

M: Why do you say the Television is worst?

R3: “Is because it is not everybody that can afford it, just very few people have access to the Television and the purpose of the thing is that they want the message to reach to the least household”.

M: Yes Sir?

R4: “The most appropriate thing is that because people in Sierra Leone some are Muslims and Christians. The government should empower the Imams and Pastors and the stakeholders in the community but government first used some boys as they see them they go about dancing in the street and people will not take them seriously and they would say what kind of sickness it this that has come to this country that this raray boys are going about dancing in the street. Now when the government has come to use these boys, people are in the community who has respect in the community, if you use such people is better than when you use small boys who are just roaming the street. Wren the radio, some elderly women are in the community who does not even know how to talk Krio but she goes to the Mosque or church her Imam or Pastor will tell her all those things but they force the boys to play music, even if they play the Music those women would say which kind of thing is this in this country that people has to dance in the street, they say is not a sickness they are only lying for them. So we have to make use of the Pastors and Imams, in some community they use teachers but the children listen to their teachers, they can go and say this is their teacher said but now it is not happening, it was only later that government. But the Head of Health was the first to destroy communication; he went on radio and said this sickness has no medicine so people began to ask what this sickness that has no medicine is? It has all the symptoms of cholera, and all other symptoms that we seen before so just want to come and kill. They could have the Imam for them to avert the problem”.

M: Yea Sir?

R5: “Well as far as what we have been talking, it is very nice that after post Ebola they should make Ebola a curriculum in schools to become a subject to every child for even our fourth generation so that they can fear it. If they make Ebola a curriculum in school it will be fine”.

M: Yes my brother you want to add something?

R6: “Yes just like what my brother said, they should make Ebola a curriculum in school from class two levels to tertiary level so that it can get an aspect of itself that they only talk about Ebola like how they were talking about HIV AIDS, and put it into a book which is plenty. This is a way for children who are not yet born so that they can meet it in the books for them to know that such thing has happened in this country. By so doing the coming generation will be alert so that they can be in readiness to attack this kind of sickness any time it broke out”.

M: Ok, In case some people have Ebola, do you think they can go to the Traditional herbalist first or they go to hospital first? Are there people in the community who still hold on to such belief?

R1: “Well in this community people now [refer to go to hospital because of the kind of sickness in our community when they see their companion trembling and die, stomach ache, vomiting and die. When they see some of them went early and they came back, so when anything happen they go straight to the hospital or they will be the first to tell people to call 117 to carry them with the Ambulance and that they will come back. That is the message that the Imams are passing that people now believe to go to hospital than to go to the Traditional Healer. Even the Traditional Healers are now afraid because when they touch they will die, Pastors touch they will die. Even when they use to go and pray for people now when you call them they will tell you to go straight to the hospital”.

M: Yes Sur?

R2: “When it comes to this particular issue of Traditional Healer, why do people go to the people prefer to go to this Traditional Healer then to go to hospital or the Heath Care Centres is one the sensitization about this particular disease which the Traditional Healers because where you go to heal somebody and in the process you too die, the person who go to get the medicine dies, the one who rub the medicine on the sick person dies, and we are glad about when government made it law that no Traditional Healer should operate and that they should close because they are ones who help with the spread of this thing so we are glad when government has intervened and made it a policy that no “Pepeh Doctor” (= a quack) should operate now people will prefer to go to the Centre for early treatment. Though it is not everybody that knows about this policy, because they are hearing about Swap Team, Surveillance Team , they are hearing about Burial Team so all these ones people never knew about them but now with the intervention of government, we now know who are the Swap Team, who is the Surveillance Team , who is the Contact Tracer, so now we have understood the one whole thing. At least it is good”.

M: Ok, any other person?

R3: “According what my brother was talking here, a typical example has happened around here, down here. The person was a Traditional Healer he tried to heal somebody then he dies, and even his brother in law he too died, they were all playing with each other so they all of them went. That is a typical example which has happened to them and must have sent a message to all Traditional Healer across the country”.

M: When Ebola broke out government did set some facilities to help contain this Ebola, for instance it established the Ambulance Service, it established the Holding and Treatment Centres, it established the Burial Team, it established the Ebola Phone Line the 117, along the line have you been hearing any good or bad thing about any of them, let us start with the Ambulance Service, do you have anything negative about the Ambulance Service?

R4: “The Ambulance, yes Sir,

M: Yes?

R4: “People used to get negative perception about it,”

M: How?

R4: “One, people were looking at it that at time there will be nobody in the Ambulance, they only go about causing unnecessary noise so people always feel frightened, even myself at time when I come to the street and see the Ambulance, I will return to my house because of the feeling that I have there is where they hold Ebola patients. Then two, they have this overspeeding, when they are passing wherever they are passing they broke traffic, they make a lot of accidents to happen when it is not supposed to be so. So those are the two I can remember for the Ambulance Service”.

M: But do you remember any positive thing?

R4: “Well the positive aspect of it is that when a person has Ebola nobody would want to load the person in his own vehicle so when they call them they will come and take the person so that is a blessing to us, that is the only thing that see as positive”.

M: Yes Sir?

R5: “My own regard toward the Ambulance. The Ambulance issue there were two problems which people were critical about, you know that initially we did not have enough Ambulance there were only few, at first we observed that we had suspected case, and then we have confirm case. Before the Ambulance comes to take the victim they would not know whether it is a suspected or confirm case they will just load the person into the Ambulance, when they meet the Ebola positive person they load them in the same Ambulance so that was the first thing that you should not load two patients in the same Ambulance when you do not know their status, there was another problem in that case, so as a result we knew that the thing was going to spread because that person who is in that Ambulance is sweating, if the first one did not get it the second one will get it because the first one would leave his or her sweat there so that is one of the factors. Then two, again is the blowing of siren, blowing which signals that we are under an epidemic which is Ebola, though at times the Ambulance can pass by with no victim inside it. But later they sensitized us that it fine for the thing to be on so that we can know that we are under State of Emergency , and now we are hearing about the Red Ambulance which they say is responsible for Ebola survivor which the good of the Ambulance, we are happy for the increase in the Ambulances which has made the Burial Team to go fast because it is chain distribution, when the Ambulance is available the Burial Team can move fast because they will be able to get to the spot on time, get to the body take swap thing and go. So it comes ti the increase in Ambulances it also combat the situation. Initially we used to face a lot of problems but as we go along we have been able to combat that case. Well the positive side of the Ambulance is that it is the only one prescribed to carry somebody suspected of having Ebola, two it is faster to take any body to wherever they want to or to the destination for treatment. That is the positive side. Time there was here when they lined all of the Ambulances up and they went on blowing their horns and everybody was like hay! Hay! Everybody was afraid, as they see of the Ambulances that make them to be afraid”.

M: Yes Sir?

R6: “The Ambulance, the negative side of it so that it is not ever where in Sierra Leone in the community that it enters, in some areas it cannot enter there even in our own area in places like (- - names of other districts - -) some areas are swampy areas, is all over swamps and small, small Islands and government did not provide boats and the Ambulance cannot enter there. Even down here at this (- - name of interview community - -) here an Ambulance cannot enter, it will just come and park in people’s compound and some people who are in that compound can be afraid that the spray is going to kill them. So that is why they always meet people who throw stone at them not park around their compound. The positive of the Ambulance is that it has rescue a lot of people, when it comes it takes people for early treatment. But the negative side of the Ambulance is about the swampy area”.

M: What about the Treatment Centres and the Holding Centres, do you have anything negative or positive about?

R1: “The negative part about the Holding Centre is that they always waste time at the Holding Centre. If they have told you that the person has vomited, has toileted and you suspect that it is Ebola and you keep the person at the Holding Centre for 1,2,3, 4,5 or 6 days, even here in (- - name of interview community - -) at the Holding Centres the Nurses do not have the mind to touch a patient until the team come and take a Swipe (swap) and go, not until two or three day before result are out when the result is out the [person will be waiting. The positive side of the Holding Centre is that when they put then at the Holding Centre, it makes the sick to spread more and more that is it about the Holding Centre”.

M: Ok, yes any other person?

R2: “Yes, with regards to the Holding Centres, and the Treatment Centres, we know that the Holding Centres initially when they take you there they draw you sample and after that treat you but initially when we had only one Centre in Kailahun (=first district in Sierra Leone with Ebola cases) it was difficult but with the help of the International Community, the British and others we observed they even the patients at the Treatment and Holding Centres has been reducing because of the fastest laboratory tests, that is the good side of it. But the other bad side of it is that initially when the thing was slow and it was only one Centre that we had, the Holding Centre get filled and people will be lying down outside because there is no place inside but now the Holding Centre can even hold four or five cases, now there people who thinks that the number of times they hear the searing is the number of cases that we have, and people are using that assumption that when you hear five searing then that is five patients when it is twenty time they assume is twenty, people are using that assumption now. So about the Holding Centre that is the only bad thing when we did not the apparatus to do they test but now that we have them, so with help of the International Community is better”.

M: Ok, any other parson? What about the Burial Team?

R3: “The Burial Team, at first when they come, people have negative feeling about them based on their attitude towards the dead. People were looking it them that they do not respect the dead because it is not their relative. Like me, I witnessed one when they came for the man, when they look the man they just send him into the Motor car, so that even used to give cause for some fracas to happen between the relatives of the person who died and the Team. So that is one negative side. The negative side is when they come they don’t want to touch the person because the Ebola is more infectious when they person is dead because when a person is dead, because they are trained to do the job, they can do it better than the untrained ones like us. So that is the positive one. So if they come they can reduce the infection rate bur if it is us, we will only add it because we do not have the technique and the knowhow to handle the dead person. So that is what I can say for now”.

M: Yes Sir?

R4: “Well with regards to the Burial Team, initially when Ebola came we heard about a Burial Team which was a small Team, but we observed that when they come they would say they are going to take the Swipe (=swab) first if they see that it is negative they will come back with the body but initially when they come they take small envelope so that they can give you the body, initially it was happening, so is like government made it a policy that nobody should touch the dead, the good side of it now is the when the increase the Burial Team to Le 500,000 (1march15 = about 114 USD) though people are now saying that it is they who do not want the Ebola to end but the Burial Team have done a lot of job”.

M: Ok, other person?

R5: “Well em, my own idea I have about the Burial Team is that the fracas that they do have when you look at the ages in a traditional societies. Like the Muslim, they would want to perform like a Muslim is dead but they prevent him or her from doing that even to go closer they prevent them, so it a woman they would want to perform as a Sowe. But all of these they said no, even to go closer they would not agree. So those society people or religious people do feel fine about those kind of things which goes against traditional rites for the dead. The Burial Team, they too do retaliate and stand against ??thit=se?? people to make sure they are the only people to do it, so those fracas used to happen which was another obstacle for the Burial Team “.

M: What about the 117 Phone Line”.

R6: “The 117 Phone Line, it is very difficult to call the 117 Phone Line, but there is delay about the channel. When you call then they delay to come it takes too long for them to locate the person except when the government used the other team to be able to locate the area where there is a sick person. people are using, and I met a scene at the (- - name of a road in the interview district - -), a dog died and they covered it like a human being and they called 117 when they uncover it, it was not a human being it was a dog they still loaded it and when they did not get vexed. The only thing is the used to delay because if the person has started to vomit, toilet, if they call 117 until the rest of the day or maybe after two days before they come there, that was affecting people in their communities because if two or three people are sleeping in a room and one dies the two will still continue to enter the room and if they call 117 they will not come, that is the problem they are having with 117”.

M: Yes Sir?

R1: “The issue of 117, initially there was a high hickup, they are slow to respond especially the Burial Team. they also observed that when they call them to a destination when they go there they will not the person, some people are using the 117 as a toy to play with, they will just call 117 and give them April fool. It came to a time when ever they want to go to some areas they will send their surveillance team to confirm to them that it is true that a person is dead before they come there but with the increase in the Ambulance and the Burial Team they are trying when you call them they come even though during the look down they changed the number and not everybody knew the number it was 442 but people were not familiar with the number and then they are continue with the 117, the 117, the code is fine to call is simple but there were people who were misusing the line they would just call them and make fool of them that is where we blame them for their slowness, that was the only problem but they did a very good job”.

M: Do your community have Ebola survivors?

Rs: “Plenty of them”.

M: Then how do the community react to them? Do they stigmatize then?

R2: “Ok, if I come in, being that they are people, we like them, we have been here for years if we hear this kind of sickness for them we would not feel good about them, we would like to see them come back and when they come we embrace them but we will still have the perception that the sick is still in their system but we can still encourage them , like me I cannot come out it to them that they have it but I will just distance myself from them”.

R3: “As for me I have two here, but I have never gone to meet them except we meet on the street and I make the hypocrite but anybody who has got something to do with Ebola I will not have anything to do with him or her, because I just think that the virus is still with and they have the tendency to transfer it to another person”.

M: But do you remember you point on their sexual behaviour; can you dilate on that a little?

R3: “Well their sexual behaviour, they told me that they should not have sex for ninety days which is about three month and we too took it from them, and we are especially the women, if you see a woman making all sort of things you need to know that she wants to begin those things again but we know them we do not get closer to them is only the men that we are fearful of the they can go about spreading it, but from the timer when the released them we have not had any other case yet., so this means that they are still abiding to what they told them”.

M: Any other person?

R4: “Yes, the issue of this Ebola survivors is that at least for everybody to accept them, that when we see them we have to embrace them, they are our brothers, they are our heroes yes, but the problem now is that is good to embrace them but the issue of sexual intercourse (Mami en Daddy business), you who is an Ebola patient the virus can be in your system for three months and those who are coming from these Ebola Centres, they came with logistics, they came with money especially when they come with the money and our sisters do not have money there is this hardship some will just say please come and give me this thing I will give you something as a result we see that our community will have a treat though we have not got such cases yet but when you look at it, it could have been better for government to put these people somewhere where they can be under close supervision until after the three month them they can be released into the communities, they should not give them freedom of movement and all the facilities. You give them 100 or 200 to enable them play around the Centre that would make them safe. It is not only our community only but the whole country, it is a threat and they need to confine them somewhere until after the three months”.

M: Do you have anything to say Sir?

R5: “Well, eh, I just want to support what my brother has just said, that ninety day which have given to the women and the men is a threat to the spread of this Ebola because that man who has come from the Centre, they come with things which sweetens the heart so when he meets his or girl friend the girl friend will say so I am blessed since he has come back this is my own time, so she will have that tendency to go and sleep to her boy friend and the boy friend will not be afraid and he will go into the woman. So like my brother said we should isolate them. All of them nationwide should be isolated so that they can be released sequentially, that is how they should release them”.

M: Up to this moment, have you heard about any medicine that will be coming for the treatment of Ebola?

R6: “Well we receive message by phone that Liberia has got a medicine for Ebola for ten thousand people, a kind of vaccine but Sierra Leone we have not heard of any medicine yet we just heard about treatment. When they were going to the Centre we never knew which medicine they were using we only see them come back as survivor, we do not know which tablet or vaccine they are using. We only heard that it has arrived in Liberia”.

M: As a community, what is the most common point of discussion that people do ask about this Ebola? What is the doubt that people have on their minds?

R1: “Well the doubt that we have on our minds is this falling and rising of figures. Today you will hear it low, tomorrow you hear it high, some people begin to ask why is it that the figure is low at one moment and the other moment it goes high. So to us we are not going to leave them until we hear that everything is ended. So those who are announcing for schools to reopen some of them their children are not here they are outside of the country, so they are now coming to cajole us again to send our children when the number keep keeps rising and falling, that is making a lot of people panic. Number two, they do not have confidence that even they can cure they sick. We do not have that confidence at all! This also brings another doubt that if they are saying the children should go to school and the sickness is against group gathering, the school is gathering system there every tendency that there is a child who have been under quarantine and no knows about him or her and can join the school and begin to spread the thing all that brings doubt”.

M: Is there anything specific that this community needs to know about Ebola that enable them to understand the disease properly?

R2: “Yes there is, some people are still denying that Ebola is not real because of things they have not seen yet. Like when they come with projector for people to see the reality of the sickness so they see how the sickness affects then they too can believe, but is like people think that is just those common sicknesses that we get like Malaria, typhoid because they have the same symptoms. I have somebody down here who is denying up to date that his wife did not die of Ebola because he said him and his were sleeping together and even the child did not get it and up till now they are all living even when we were talking here the child passed here running and it is forty nine days today”.

R3: “Yeah, with regard to what my brother said I just want to add, this issue of Audio visual recording of targeted areas where we can shoot it and exhibit for people to see, even those who will pass by will have a pre knowledge even when people are denying they are still asking why the high rate of Doctors dying? some are saying maybe the Doctor forget themselves , some are saying that the Doctor would have detected early signs, and that they did not go early so this was the discrepancy among people that why are Doctors dying when they are supposed to detect the signs early? “

R4: “Some were saying Doctors do not know the measurement of the chlorine they are using”.

R5” So even the use of this chlorine they say they use to mix it with other and this and that. You can see the Swipe Team (=Swab team) comes, the Surveillance Team and all the rest, we have done those entire things and yet”.

R6: “And also people are doubtful that government has never announced that a member of the Burial Team has died but has announced the death of Doctors but Burial Team are so much at the front then the Doctors because they take the body, they dig the grave and bury but they have never died only Doctors die. They say government is only lying for them, that they are killing their own Doctors.”

R1: “I would also like to add something, although I did not respond on the question on the vaccine, based on the information that we are getting that we don’t understand the ??repacaution?? of the drug so like we in Sierra Leone here we are look at what is happening then we can follow”.

R2: “Even like this Malaria tablet which they gave out people say the medicine and the old box is the same but the tablet is different, even my mother at home she took one and two days she couldn’t get up and then people began to say they have put the Ebola in their to spread it. Now she said she will not take it again. She said the white men have come to kill us”

M: Ok, I thank you very much.

**ADDITIONAL PART OF INTERVIEW, OBTAINED BY COLLECTOR 5 AFTER CONSENT IN PERSON with two (2) of the original participants, March 2015:**

M: From our last discussions, someone raised a concerned that the market women are harassed, arrested at night and the people that do this arrest also take bribe from the market women, I want to know, the people that carryout the arrest, and why they took bribe from the market?

R1: “The night arrest is done by the Sierra Leone police, two or three may come and arrest, after two to three seconds they will stood and talk then, they released, so we thought that it is a bribe, because when you arrest someone, within two to three seconds, then you released the person, it is bribing”.

M: Have you seen where it happened?

R1: “Yes, at that junction, where we usually sit, there is one woman that sells cake, the other sells slippers, at that particular time, they have packed up and covered their business already, so they came and arrested them, they said it has past time, so they stood there for a while, all of the sudden they released them”.

M: So when they were released, did you see any other thing that happened?

R1: “Well they were far away from me”.

M: Ok, so you can’t tell what happened if there was bribing or not?

R1: “Yes”

M: OK

R2: “So if I could add to what my brother said, on several occasions I had witness this arrest, some of this police officers come around to arrest even before the stipulated time for the closure of business per day which is six o’clock (6pm), like for example, a lady who sells “acheckeh” (= steam cassava) at (- - name of the garage - -), the police officers came and arrested the “archeckeh”(= steam cassava, before she could reached the police, she met the police officer eating the “archeckeh” at (- -name of police barrack- -) and I had also witness another occasion at (name of place), where the police officers came and arrested different business items from people which includes breads and put in the vehicle and took them along, some us were expecting that since it is a law that is put forward by the president that six o’clock everybody should close up sales for a day, we are expecting that the people should just come around and tell the people to pack up their business, ensure that everybody as pack up and gone home, but some of these police officer and military personnel took advantage of this situation, within the process of arrest , they will take anything the meet even money, which they may not refunded to the people, this is not anything hidden, I had witness once or twice, even in another incidence again, they met one woman having an empty Coolman, they arrested her, then I had witness again another boy in his shop, nothing was even in shop, at that time he has closed the shop for that day but he forgot his shirt, when he came back to collect the shirt, the police officers arrested him, then he said to them, there is nothing in the shop, I only came to pick up my shirt, so the argument continue, so they later arrested the boy and took him along, the boy end up given them two hundred thousand Leones for him to be released, this things are happening even now”.

M: some people said in our last discussion, people move from one place to another to seek treatment when they are infected with Ebola, have you seen cases like that?

R1: “You mean the survivors”?

M: No, the newly infected patients with the Ebola virus disease?

R1: “Ok, if could throw light, even in this community (- -name of interview community - -), Ebola was brought here by someone, this person travelled from (- -name of town- -) to here, on a business trip, this person came and lodge to a business partner in this community, overnight, she got an attack, they said she pronounced that she had Ebola, so in the morning, they manipulated again and returned the person to where she came from, not knowing that she had already left the virus. So the business partner got sick, so people do not the story, so anytime they saw the women, they say, they had “Gbhaha” (= a Temne word for an artificial sickness), so she came with it and allowed her community to suffer, because she scattered it all over, a lots of people suffered”

R2: “if I could make an addition on that, this Ebola, only a laboratory test can proof someone positive or negative, but will saw instances when people fell sick, some prefer going to hospital, especially we that are down (- -name of community - -) considering what happened to people at the beginning of this outbreak, a lot of them survived after visiting the hospital, so when they returned, they were telling their other people, if they are sick, let them go to the hospital, at least there is that confidence now, whilst some of them that have not contacted the virus, do not believe, they are just thinking, if they go to the hospital, they will kill them”.

M: Yes sir, what I am saying, have you seen or heard any instance when an Ebola infected person moves from one place to the other to seek treatment from a traditional healer or a traditional way in this community?

R1: “No, we don’t have it here, but we have heard of it some people moves from (- -name of town- -) to another town, but for us here, they brought it for us”.

M: In our last discussions again, someone spoke of ignorance, traditional and cultural beliefs and also the denial and negative attitudes of peoples has led to the spread of this virus, so I may like to know, what is the ignorance, traditional and cultural beliefs that is leading to the spread of the virus, can you give some examples?

R1: “Yes, let me come in, our own case in (- -name of community- -) is a case study, ignorance in the sense not that the people do not know the cause of the sickness, how it affects, they only knew just a little about this sick, like the woman who spreads the sickness here is a “sowei” (chief priest of the female secret society), she was not knowing that this Ebola sick will stopped a pregnant woman from giving birth to her baby, when the pregnant woman is infected with the virus, she was not knowing that at all, it was just after when the woman was invited to a workshop, were she asked this question about the pregnant woman having Ebola sickness, so they answer her question, that when a woman is pregnant and has Ebola sick can’t be able to give birth, so straight away she put her hands on her head and said O my God, I am in trouble”.

M: So what are the traditional and cultural beliefs you said that led to the spread of Ebola?

R1: “When a pregnant woman died and do not gave birth to her child, our people has the tradition that, they have3 a medicine that they will rubb on the stomach, so she can deliver the baby, that is the tradition, and the tradition again, this native way caused a lots of problems, as the “sowie” (chief priest of the female secret society) has said they had “Gbahagbha”(artificially stopped the woman not give birth) so they has that mind that the sickness of the pregnant woman was not Ebola but “Gbahagbha”, so that touched the woman”

M: Who are the people that has this negative attitude and denial, you were talking about?

R1: “Well even the doctors, when this sickness started, they pronounced it that, this Ebola sickness, does not have medicine, so the people had it mind, if there is no medicine, why should go to the hospital, that was where the denial first started and some people gave money to another people like young boys, going into the street, playing music, shouting there is a sick, boys that you can even give birth to. So with these things, the people took up the denial that they do not believe Ebola is real, at that point government should have gathered all imams, teachers and stallholders in the community and tell them that, this Ebola sickness so it happens, they said you will vomiting, but all these signs and symptoms given by the government through the medical expert, that has been given to us, these signs and signs and symptoms had happened before, so now people are not afraid, some of them had the feeling that, when their brother died, they will touched, when their relatives sick and vomits they will not them to the hospital and people were afraid again, when they talk about ambulance, before, they do not know what they called ambulance, that was the reasons of their denial, even the young guys, they don’t know. Even the young guys, they do not know, what is Ebola, some were singing that Ebola does not exist, and even the politician also says different things about Ebola at different places, these are all the facts that brought up the denial, even in Sierra Leone and beyond Sierra Leone, they don’t still believe Ebola Exist because another scenario also happened in our community, a wife of a man died they said it Ebola, and after her death, the child and man who was touching and taken care, of the woman, do not proved positive and up to now, the children are still going strong, nothing her happen with them”.

M: Another person in our last discussion, said the Ebola survivors are threat to the people, what do you mean?

R1: “The survivors”?

M: Yes?

R1: “We saw them as threat, because, when they are discharged from the treatments, they only give them condoms then tell them not to have “mami and daddy biznes”(sex) for ninety days, human being whatever will be the case, we are all selfish, being that the person is a survivor, hence he or she had got the sick, he or she may not be infected again, they may do something different and do not consider it serious, since the thing is not affecting the person directly, we even a case of one survivor, someone met him kissing with a lady, so we raised up that alarm, then people says no it wouldn’t be so, the Ebola survivor was also denying that he did not sick of Ebola but malaria, so a person of that nature, arguing us publicly about kissing his girlfriend, lets me assumed now he was in a place where there is no person, what would he say he did, so is a threat to society”.

M: How would traditional healers contribute to the negative attitude of the people, can you give examples?

R1: “the short example I am given, even that “sowie”(chief priest) is a traditional healer, if they had told her that, the pregnant woman was having blockage and she produced medicine for her, so the “sowie” woman touch the Ebola infected pregnant woman and also she got infected, and the “sowie” woman spread the Ebola sick to all his family that was living with her including children and all the people in the area, so that is the negative attitude, because she do not know, unless the china medical came and trained them for two days, and they pronounced to her what she did, then she said ooo I am in trouble and as she came back, the sickness started spreading, it was during the that western area surge, everybody got the sick now at the house”.

R2: “Yes sir, if I may add to what my brother said, the negative attitude of the medicine men, the medicine men are having it in mind that, they are competing with the traditional doctors, because some illness which a traditional doctors cannot handle, they can handle it better, we have seen instances of these nature when they meet”.

M: What do you mean by traditional doctors?

R1: “The trained and qualified doctors, so they are taking it that there are some sickness which the trained doctors cannot cure, but they can cured, we have seen an instance , one women was sick, they said, the trained doctors have treated the women, but the woman could not cured, so a traditional healer said, he can cure the sickness of the woman, we heard that in our home (--name of town--), there is a man, which is a traditional healer, they had warned him to stop healing people but he is still doing it, he has moved to the middle of the bush, when there is a case to heal, they will called him to come and heal, these are all negative attitude towards the sickness, people are not still taking the precautions of this sickness, so these are one the negative things the traditional healers are doing”.

M: How do the community people behave to other people that attend burial ceremonies?

R1: “This is where the denial came up, some communities are still doing this, they buried at night, particular (--name of district--), they still buried at night, it happens a dead man was buried at night, so when they suspected that, they have burial at night, the people denial it, when the ambulance arrived they had already buried the man, the person that did the night burial also died, he died far off, even some churches they usually say they have done a test on someone that had died, when they are about to church the person, they will say the government had tested the person, another incident happened, when I was travelling from (-- name of the town--) to (--name of the town--) we met a vehicle with a dead corpse, they said, the government had done the test , but the police officers were not having any testing equipment to test at the moment, so they released the dead body. They moved with the dead body and entered the city and they churched dead body. This is where the denial started. When the Ebola broke out, the education department do not educate the people welled, they blunder totally, they would not have tell the people that the sickness do not have medicine. The signs and symptoms of Ebola which is vomiting and frequent stooling had been occurring to people in Sierra Leone, they should not utter statements like that, and they would just take the people to the treatment centre, so people still buried their love ones”.

R2: “If could add, this issue of burial, in our own community, we do not burial here at all, when someone died, we called the burial team, above all, the contact tracers are doing welled, they are doing a very good job, because I have witness one incident, it was even a rumour, when they said one man was sick, they called 117, the ambulance came and took the person to the holding, tested the person, after two days they brought the person, look at the person sitting over there, so it is not easy for someone to die, which his or her neighbours do not aware of the death, so I have not seen that at all”.

M: Someone used the word “Ancholera” which language is that?

R1: “Ancholera” I am the one that used the word, it is a Temne word “ancholera” meaning, diarrhoea, frequent stooling, vomiting, abnormality. So frequent diarrhoea is called “Ancholera” in Temne”.

R2: “So the people were denying the sickness that, it is not Ebola but Cholera, so instead of calling it cholera, they called it that in Temne “ancholera”.

M: In our last discussions, someone spoke of people denying that fact of Ebola existence and they continuous touching, which even led to stabbing of a person, can you please tell what brought about the stabbing and who did the stabbing, and what object did the person use to stab?

R1: “I do not understand the question sir?

M: In our last discussions, someone spoke of people denying that fact of Ebola existence and they continuous touching, which even led to stabbing of a person, can you please tell what brought about the stabbing and who did the stabbing, and what object did the person use to stab?

R1: “Well if could stepped in, the stabbing happened, when a man touched his friend, so the friend told him, don’t touch me again because you said Ebola do not exist, so they were having that indifferences, so he came again and touched the his friend, the friend told him, if you touched me again, I will stabbed you and the other day he came again and touched his friend, his friend end up stabbing him”.

M: What object does he use to stab?

R1: “He used razor blade”

M: So the reason was when the friend touched him?

R1: “Yes, when the friend touched, the person that was stabbed do not believe Ebola exist, rather the person that did the stabbing believes Ebola exist, some his man said don’t touched me I also don’t touched you, let wait after Ebola”.

M: So who actually did the stabbing?

R1: “The one that believes Ebola exist, is the person that did the stabbing”

M: Another thing came up again in our discussions that, they did not explained the precautionary measures of Ebola well to the people, what do you mean by this, or you state explains of what needs to be properly explained?

R1: “At first when the sick came, it is a new sick, we did not have any understanding about the sick, the first message that was disseminated, this sickness do not have medicine and people were having that concept, but after sometime, they came again and said when you are sick and go early to the treatment centre, you will cured, so this popup confusions in the minds of people, how can you say the sickness do not have a medicine, people were having that confusions, but during the sensitization, there are key stallholders that they should have employed to do the work, but you can’t employ any irresponsible person to do the work or to go out there and talk to people, some people will not listened to them and they will not even take to be serious people or serious message they are passing to people. All the boys we saw in this area sensitizing, in fact they were just sharing soap, they don’t have nothing to tell us and we listen, because we knew more than them. So the boys they used the last time, do not disseminate the message well, we were expecting them to used key stall holders in the community like pastors, teachers, imams and landlords, these are the best people we were expecting them to take for the sensitization because if you used landlord, the landlords will talked to their tenants and they listened, or if they used teachers, the teachers will talk to his or her pupils parents and they will listened and put into practices and they used imams to talked to the congregation in the mosque, these key stakeholders will disseminate the message better than those small boys”.

M: Somebody used the word “awol”, what do you mean by this?

R1: Well, “awol” is a krio word meaning, a person that left his or her house and stays outside, so when they said “awol” they were using the “awol”(street boys) boys to do the sensitization for them, so when they were moving along, they referred to them as “awol” boys, these are the boys, that do not have respect for people in the community, so this type of boys, when they sensitizing, people will not take them as seriously”.

M: What language is that “Awol”?

R1: “it is a krio language “awaol” is a military terminology which means absent without leave”.

M: Ok?

R1: “But our own meaning, is a person that is in the street without controll”

.

M: Someone spoke again of (- - name of radio program - -), I want to know if this monolog is a radio programme or a television, and which name radio station broadcast this programme and the its role in disseminating the Ebola message?

R1: “Well (-- name of program --) is a radio programme, broadcasted by citizen radio”.

M: What is the frequency?

Rs: (--frequency and name of local radio station--), they are the first radio stations that started doing the sensitization about Ebola, as the outbreak started, they begin the sensitization, so of us were taking it as a joke and the presenter of the monolog programme (name of person) said that anything that begins in (--name of town--) will end in (--name of town--) so let don’t sit down but let’s be conscious and work harder, but you know when something occurred newly, some people will not take it too serious until finally it reaches here, so that is what I know”.

M: In your last discussions, some people spoke of an artist singing a political song, I may like to know, the title of the song, name of the artist and the message the music is about?

R1: “Well our artist in Sierra Leone, had sang a lot of music at the start of the Ebola outbreak, all the music is about Ebola, Ebola, some say Ebola must go”.

M: This Ebola must go music who sang it?

R1: “There is one that was sang by M.B Atila, the deputy minister of social welfare, this is one of the music that the government is using”.

R2: “There is another music again, that was sang by big Joe”.

M: Do you tell me the title of the music that big Joe did?

R1: The title of the music is “Ebola is a killer disease”.

M: What is the title of the music that was sang by Atila?

R1: the title of the music is Ebola, Ebola. He sang a whole album”.

M: The one he did for the for UN women?

R1: “Yes”.

M; Ok, I had also heard it, titled, yes Ebola must go?

R2: “Yes, Ebola, only Ebola”.

M: someone said President Obama sent a message, what message did president Obama sent?

R1: “Yes, I was the one that said that”.

M: Which was the message that president Obama sent, can you talk on that?

R1: “Yes, for me, I took into granted the sensitization Obama did, at one time president Obama said Ebola is a threat to human survival, he said but only that, if we rely on them, they will controlled the disease, he further stated that, if you even exchanged money what a person that has Ebola, does not mean you will catch Ebola, he said it does mean if you sat by a person with Ebola in a vehicle, that you may contact Ebola, he said what you people had to reframe from is washing of dead bodies and the touching sick person(s) and I believe in that principles and I am following the principles”.( *the sound of a siren)*

M: So that was the message?

R1: “Yes, and indeed all the people that had contacted the virus, they went against the principles and they were touching, washing dead bodies”.

M: Ok, someone spoke of saga in their community, what saga was the person was referring to?

R2: “The saga we were talking about, is this Ebola saga, Ebola saga came in our community especially our community (--Name of community--), it put everybody off totally, they quarantined forty-seven houses, more than three hundred people in quarantined houses where totally off normality. That was why the used the word saga, it this saga is still continuing, like for instance, the house the quarantined down here, the mother of that Ebola infected pregnant woman, did not get infected with the Ebola virus, she is survived and she is welled but all the people that was infected with virus, who went to the treatment centre, those that survived and came back, there is a big problem now between them, every morning they used abusive languages on the mother of that woman, telling her, she brought the sick in their community that has killed them ”

R2: “They said, the woman did not touch her daughter, they said she suspected that her daughter was having Ebola, that was when she was helping her, she used a plastic as glove, so was saying, please touched my daughter for me, all the people that touched, where infected with Ebola, this is the saga that is in this community, even right now, they were using abusive language on her, that if the mother of the woman past again, they will abusive her mother. So with this people had advice the woman to leave the community for now, because some people may attacked her physically,”

M: Ok, they were talking again about “raray boys”, who are these “raray boys” and what is their role in disseminating Ebola messages, where they paid or hired or they did it voluntarily?

R1: “The “raray” they were using”.

M Who are they?

R1: “They are idlers, people that are in streets and do not have any strong abode, these idlers were used to sensitize on Ebola, during the three days lockdown, they were passing and shouting Ebola exist, Ebola exist, some of this boys, their trouser were down their waist, some with appearance, the people won’t accept the message that the sickness is real,”.

M: So were they doing this voluntarily or they were hired to disseminate the Ebola messages?

R1: “Well we do not ask them, but they will not do it voluntarily, they paid them”.

R2: “They give money, but these boys that they employed, they did not do the correct Job”.

M: Another person mentioned that the head of the health ministry, destroyed the Ebola communication, who is this head?

R1: “When they said the head of head, the ministry had different department, they have the education department, the education department destroyed the communication, between the community and the hospital, they said this sickness does not have medicines, they saw people wearing PPE (personal protective equipment), the people were afraid, they said let don’t go the hospital again, so the education department of the health ministry, destroyed the communication, between the community and the hospital, the first message they said there is no medicine for Ebola, that created even pregnant women are afraid to hospital, because they said, when they go, they will die, so they will end up staying and touch at home, the communication between the community and the hospital cut off totally, when you go to the hospital, they said unless you sat down for three days at the hospital without treatments, waiting for reports at the hospital. Even the health practitioner, do not have the know-how, they are afraid to touched the patients for them not to get infected, that was the reason they cut-off communication between the hospital and the people”.

M: So who is head of the health ministry?

R1: The head of the ministry at that time was Miatta Kargbo (= previous minister of health)”.

R2: “Doctor Brima Kargbo (= current Chief Medical Officer) and Sedi Yayha Tunis (= department head in the Ministry) were always on the radio talking on Ebola issues.

R1: “Where I want to blame the ministry of health, because I made mentioned of that, before that sickness spread widely, the health workers do not do the actual work, because this sick started in Guinea, it was in Guinea within few months, it came in Liberia, all of these countries we shared boarders with Sierra Leone, if we were expecting he sickness, we should have prepared before it comes, they should have built a lot of holding centres, treatment centres on the alert, they will be on the standby for the first Ebola case they will hear, but they did not do that, they waited after the sick broke out in this country, at that time, we were having just one treatment centre, it was in (Kenema), so when someone fell sick, it will took four hours to take the person from here to the treatment centre, so people, before they arrived, they had died”.

R2: “An accident even occurred at a time when the ambulance was moving patients to the treatment centre, five of the patients including the driver died, only now they have removed the ambulance from the accident point, so we were not ready for the sickness at all”.

M: So is that blame you are talking about that destroyed the communication between the hospital and the community?

R1: “Yes”

M: somebody mentioned, about a red ambulance, what was the purpose, which organization had it, and what inscription was on the ambulance?

R1: “All the ambulance that came, was red, and they used red colour to write on them, every morning, when they are passing, if even they had patients or not they will just be sounding their siren “woooov waaaaav”, some people are afraid now, when they saw the ambulance, because even your head is aching, your controlled it, because when the red ambulance is passing, in fact it was one ambulance, there were red ambulances. The last time thirty- six ambulance was passing, people came out watching the ambulances, the ambulance were just sounding their siren, “waaaawaaa”, they were brand new, no person was inside the ambulances, unless the drivers, they took them from water queue, so people were saying O man God, government has killed us again, we will not have that

Zeal to go there, so this destroyed the communication”.

M: so the ambulance don’t have words written on them?

R1: “There is a one that move with survivors, and it is a long one”.

R2: “Japan ambulance, it has moon and star, it is a red ambulance”

M: So there is no name?

R1: “In fact all the ambulances don’t has number plate, there is no tagged that identify, if it is this type of ambulance or not”.

M: Another people mentioned again, a set of people don’t wants Ebola to end, who are this sets of people and why they don’t want this Ebola to come to an End?

R1: “I am the one that utter that statement, because the government, when this Ebola started, there are people in the ministry of health, which government employed and they are paying them, my brother, for instance if a person is paid five hundred thousand Leones for just working on foot and sensitizing per week, so within a month, that person will be having two millions Leones per month, will these sets of people may like Ebola to come to an End? No, the person will make it more worst, for a continual employment, because Sierra Leone we don’t have job facilities at the ministry of health, another man said, if they should had use teachers, that are sitting down presently without job and paid them on hundred thousand Leones per week plus their salaries they are receiving, this sickness, should have end long ago, Most of the boys they employed, some can’t even writes properly, when they writing line list, they don’t do it properly, which is now creating confusions. At a particular place, they will supply more one, it is just because of the incompetency of this boys that they had employ”.

R2: “Yes sir, if may add, what was the question again?

M: another people mentioned again, a set of people don’t wants Ebola to end, who are this sets of people and why they don’t want this Ebola to come to an End, your colleague made measured of the sensitization team, so can you give example of people of that nature?

R1: “I will divide that into two sectors, because in this country, we had high unemployment rate, some people have secured a job, during this outbreak, plenty people that was not having job, had secured a job now like the drivers, contact tracers, medical people, and even other people that do not knew anything about medical, they are all employed now, so they had the feeling that, they had a secure a permanent job, failing to know that, this is just a crisis management after the crisis, they are going to lied them off the job, they should do the correct thing to reduce the sick, they are not doing it, they will do the wrong thing, at the end of the day, the thing will be getting worsted, for me I am seriously blaming the white people that came for this Ebola fight, we knew that when most of this guys are coming, the pay them in thousand and dollars either on monthly or weekly, but I can imagined you had cured someone and this person end up posing threat and after they had cured the person, they released the person and they only advice they will give person is do not have sex, then you gave the person condoms, so there is a possibility for the person to spread the sickness, with these thoughts I said, this sick will not end quickly, because these survivors they are releasing, will spread the sickness the more and those that had a job, are still continuing doing their job, and everything is going on”.

M: Well they said there was a fracas between the people and the burial teams in relation to age, they said the age of some members of the burial teams is not commensurate to what the society expect. So can you please tell me why are societal people are not taken the members of the burial team, very well?

R1: Well in our community settings or tradition, some people may like to give last respect to the dead, the believe that it is the last respect they given to loved one after death, it has been happening in several occasions, most of the people who handle the dead for burials are around age limit of 25years, 30years, because this age category has respect for the dead and knew how to handle the dead, in the case of this burial teams, some of the members are small boys, and some of these boys before they come burials, they had drink and drunk, because they said, it is a job that is risky, and maybe when they are doing the job they may die, so they would be under the influence of alcohol, that empower them to do the job, so in the process of executing their jobs, they don’t treat the dead bodies of other people properly, there was an instances, one of the burial teams went to collect one man, after taking the man from inside the house, they just thrown the man into the ambulance, so confrontations raised”.

M: So that was the fracas?

R1: “Yes, so they started throwing stones, they even broke up the driving glass of the ambulance but now we are seeing a little improve, I don’t, if it was because of the confrontations”

M: Ok, some people said again they were given money, so who are the people giving this money, and to who they were given this money and for what purpose”.

R2: “This fracas that was happening, some people were not satisfied with the treated given to dead bodies of their loved ones, so this burial teams were given money, for them not to take along the dead bodies for burials, so people bribe the burial teams, so they government announced that, they should not give any money to the burial teams, because before, first when you do not want them to bury your relative that died, you give them money. So that led to the spread of the sickness”.

M: Some one measure that they were given hundred or two hundred, is it Leones or dollars?

Rs: “Leones”.

R1: “Some people were giving the burial teams, one hundred, two hundred Leones, for them not to take their beloved ones for burials, so when they leave the corpse to them, they will care and performed the burial rights, this led to the wide spread of the sick, for instance, if I had and give them money, they would not take my dead relative for burials, they will just leave it with me”.

R2: “They were exhorting money from people, on a grounds that, they will treat your dead family member, the way you wants, and there was problem with 117, they called them, two to three days, they will not response, so if you want your process to go faster, some will go out and look for them where ever they are, and give them money, for them to respond quickly. For example, a dead body was lying around here for two days, so the parents of the dead body said, go and wait the ambulance at the junction, when they are passing, then called them, so when they were passing they stopped them, but they said, they are not going to take the body, because they do not command them to take it, so after, that they spoke, I do not knew what happen between them, they later, came back and picked up the dead body”.

M: Ok, another people, says, they come with something to sweeten the heart, who are they and what would they come with?

R1: “They come with the Leones, the money is one they come with that sweeten the hearts that is given to the burial teams, for their dead ones to be treated properly”.

M: Who are the people receiving the money?

R1: “The burial teams”.

M: Ok, the burial teams received the money, but who gave the money?

R1: “The parents of the victims or dead person”

M: Somebody mentioned that, they will kill their own Doctor, what do they mean?

R1: “killed their own doctor? I don’t understood the question”.

M: Somebody mentioned that, they will killed their own Doctor, what do they mean?

R1: “Ok, they were referring to the sick”.

M: Ok, that was what “they” they referring to?

R1: “Yes, because the sick killed the people that cured it, the sickness killed the medical practitioners, we had seen in many cases the virus had killed a lots of doctors that was in the fight, So that was the meaning, killing their own doctors, specific doctors had died”.

M: There was a story, which they said, a woman made a statement that the white men had come to killed us, what do they mean?

R1: “The saying that the white men have come to killed us, when the sick broke out, our people do not have the know-how about the sick, so all the signs and symptoms they gave on paper has been there before, we were touching ourselves before now, now they said we should not touched ourselves, they said if you touched, you will die, so with this, the people in the community said, the white men had come with chemical to killed our us in the community, they had come to killed we the poor ones, those that has money do not die, that was the reason they said, the white people had come to killed us, because they are the same people that produced the medicine”.

R2: If I could add on that, there was an instances that occurred in (name of town), wherein a whole medical nurse took up megaphone, parading in the street of (name of town), saying, that they the white men had come to killed us, they need our bloods. So she made all these assertions that the sickness do not exist, they are just here to take our blood, people took this statements granted, thinking that, the woman works at the hospital and she is making these types of assertions, maybe she is saying the truth. So all these news were coming as rumours”.

M: Ok. I thank you very much.

Rs: “ooo”
