# Supplementary material for: Development of a set of community-informed Ebola messages for Sierra Leone
Source: PLoS Negl Trop Dis. 2017 Aug 7;11(8):e0005742. doi: 10.1371/journal.pntd.0005742 (PMC5560759; doi:10.1371/journal.pntd.0005742)
Supplement: S1 Appendix — (ZIP) [file pntd.0005742.s001.zip › Ebola messages - FGD and interview transcripts/R2HC Ebola Fieldwork 1/R2HC Ebola F1 FGD-MAYOU-Urban2 V2 ADD PROBE.docx]

| CODE | **R2HC Ebola F1 FGD-MAYOU-Urban2 (urban focus group discussion)**  **V2 – 11^th^ March 2015 – ADD PROBE and corrected personal data** |
| --- | --- |
| DATE | February 2015 |
| DURATION (minutes) | 47 |
| Collector nr | 5 |
| LANGUAGE INTERVIEW | Krio |
| **TYPE FGD** | Younger males |

**PERSONAL DATA PARTICIPANTS**

| Nr | Sex  (*F/ M*) | Age  (*in years*) | Education Level (*e.g. none, Primary, secondary, tertiary*) | Language (*e.g. Mende, Temne, Krio)* | Religion | Job / Employment (*how they earn their living e.g. farmer, teacher, trader*) | Role in community  (*e.g. youth leader*)  ANONYMIZED, ONLY AREA OF ROLE INDICATED |
| --- | --- | --- | --- | --- | --- | --- | --- |
| 1 | M | 25 | None | Temne | Muslim | Carpenter | None |
| 2 | M | 23 | Primary | Limba | Christian | Welder | None |
| 3 | M | 21 | Tertiary | Fullah | Muslim | Student | None |
| 4 | M | 25 | Secondary | Temne | Muslim | Trader | None |
| 5 | M | 24 | None | Koranko | Muslim | Barber | None |
| 6 | M | 22 | Secondary | Mende | Christian | Student | None |

**TRANSCRIPT: (M = Moderator, R= respondent, R1= first person responding to a question, DOES NOT correspond to numbering used in Personal Data!)**

M: My first question for you is how has Ebola affected you community?

R1: “Well Ebola has affected us too much, so much really. Because everybody in the country knows that ( - - name of interview community - -) has been a red spot (= hotspot) even when people were pointing fingers at us that we are the owners of Ebola with many cases, but we thank God when the youth in the community took up the responsibility so we were able to get up to 30 to 50 people who were sick and 80% of them who came out sick so that gave us so much worries and it really makes us think that we are not safe but we thank God but it was really, really difficult”.

M: Has anybody anything to say? Ok, have you ever seen or know anybody who has Ebola?

R2: “We have seen plenty of them”.

M: Why do you think Ebola spread in Sierra Leone?

R3: “First of all, it is due to our carelessness, we have not listened to what the professionals are telling us, that is why the sick is spreading. Again is because of the Temne Line, the Temne people are very stubborn, even when they tell them the right thing, they will not take it and onto this time the thing is not still in the Temne Line, up to now cases are still coming up”.

M: Yes?

R4: “Well on my own part I want to start with what made the sick to spread. I start with the government, the sickness was in a small area in Kailahun (= first district in Sierra Leone with Ebola cases) and I feel that as a higher authority in the medical business they would have been able to got an idea on it and try to call and know about the sickness, but they allow the sickness to spread into Kailahun, from Kailahun it came to Kenema and onto date the sickness is affecting us. If during that time when it breakout in Kailahun the government could have taken strict measures to put it under control, if they had done we could not have had Ebola spreading in the country”.

M: Yes?

R5: “I believe that it is lack of understanding that made the sickness to spread. First of all we never had knowledge over Ebola. The way we are hearing about Ebola in Congo is quite different here so a lot of people took it to be a lie when they said this is how Ebola affects that when you get it you ooze blood. They will say it is Ebola while all the symptoms of it is similar to Malaria, at the first stage is like Malaria so initially it is like Malaria. Most of our Doctors and Nurses coming right to the community”.

M: Yes brother?

R6: “Eh, just like I have said, it is the government because it started in a small area then they were not able to take measures to stop it until date when it has spread in this way”.

R1: “I also want to add that the government, we are looking at it that, because we are now tired of this Ebola the government has to take radical measure but they want to put politicise it. You know when they know that somebody is going against the rules for Ebola not end, instead of they treat the person radically they go about encourage people and that is the more the thing gets worst so the government needs to do something now. Now let us say they had done the mistake so to fight it radically is the best way now to fight it. If they tell you to this when you did not do it if they have to keep you, they keep you”.

M: Emm, do you have any local term that you call Ebola, like in Temne do you have any local term that you call it?

R2: “No”.

M: What about you who is a Fullah?

R3: “No, we do not have”.

M: Some people still believe that Ebola id not real, do you still have such people who do not believe that Ebola is real and do you know why he thinks that Ebola is not real?

R4: “It was in the past. But now I don’t think there is anybody in ( - - name of interview community - -) who does not believe that Ebola is real because when Ebola enter in ( - - name of interview community - -) about 45 people that I know were attacked and 80% of them were infected, so let us say if we see 2, 3, 4 people died of Ebola so I don’t if there is anybody in ( - - name of interview community - -) who does not believe that Ebola is not real”.

M: So like those messages that you have seen, you see those postal, can you tell me some of the messages that they were telling you, you see it in TV, and you hear it on the radio, even Airtel too do send texts. Can you tell me some of those messages that they give you?

R5: “One they said we should not touch the sick then two they said we should stop washing dead body then they said to avoid body contact and to wash hands regularly with soap at all times“.

R6: “The first message which was ‘EBOLA IS REAL’ that was the first message that came saying ‘Ebola is real! Ebola is real! You should not gather in one place because through gathering can make some contact the disease and all sort of things’.

M: So what do you think would be the best message, for instance, suppose the government comes to you and ask you to go and talk to somebody who has a sick person at home, how can you go about, which kind of message can you give to the person to encourage that person to take the patient to the hospital?

R1: “Well that is like a common thing now especially here in ( - - name of interview community - -) because we have seen the example if they say a person is sick in this house although let me say if you are a distance say about 100 yards even if I know you but I don’t know where you are coming from I will tell you that before you keep this sick person at home please take the person to the hospital, or you do not know what kind of sickness is affecting the person, it might be that it is Ebola you might get affected you can call 117 they will come and take the person”.

R2: “Just like what my brother is saying if anybody is sick at home for the love of your family you have to tell the person to go to hospital because if it happens that the person has Ebola then you will infect the whole family, everybody will contact the disease so with that if you tell the person that he or she will say because he or she loves his or her family he or she will report him or herself for them to come and take the person to hospital”.

M: So from all those channels that you get information, like the radio, television, newspaper, which one do you think is the best way for these messages to reach the people?

R3: “Well like the radio, yes it goes but there are some communities there are people who are not conversant to radio you know, I believe that the door to door sensitization goes more because even if I do not have radio, even if the meet me at the market area they will bring the message to me, so the door to door is more important, the message goes more than any other medium”.

M: Ok, yes Sir?

R4: “Well just like what my brothers said, because normally most of us in our community we found out that yes, the radio and the media are doing a lot but some time, the people, not everybody is educated or listen to radio, in the morning they go out to find their living but I believe that through the organization of USEM, who give people megaphone to go house to house, that door to door it will, be beneficiary for us all especially here in ( - - name of interview community - -) some of our people were denying that this sick is not real not until when then President announced this scourge, the government was able to go house to house and begin to notice that there were sick people who kept in houses, so we talked to them and carried them to hospital about 17 of them and we met about 50 people who were positive, so here was a death zone. If they continue I think it will be more beneficiary to us”.

M: ok, now let us say somebody has Ebola, where do you think the person will go first? Do you think he will go to ‘meresin man’ (= traditional healer), will he go to the existing Health Centres or the person will run to the Centres they have just built?

R5: “Well for now we have people in the community through sensitization and through the messages that we have passed, there are some parents now even if their child is sick is either they call 117 or they make him go to hospital or to the Holding Centre. The person will be there now until they take sample if he is positive now then they take the person to the Treatment Centre. I think that is the best way that when a person is sick you take the person to the Holding Centre”

M: Ok, can you tell me about the good and bad things you hear about the Ambulance Services?

R6: “Well at first we were hearing a lot of bad things that made people to hide instead of reporting themselves to the hospital because people say the chemical which is inside there, if they load you there because when they load you there they have to close it and no air comes in, so the chemical will affect you, that is it even when they were taking patient from here to Kenema (= second district in Sierra Leone with Ebola cases) they tell us. If you look at the distance now if you load a patient here and load him into the Ambulance to Kailahun (=first district in Sierra Leone with Ebola cases) the person will die, he will be weak, but now we are getting better stories. I don’t know is because we have Holding Centre close in town (= close to / in our community) because now we see, we can even call, we can call for them to come and take a patient. Then in less than 30 minutes or one hour he will call now to tell you that he has reached, that is better now, yes”.

M: Ok, as you have heard now the Ambulance is passing, but the kind of stories we get about the Ambulance, one is the noise which they make when they are passing, some would not even carrying a patient they only go along causing noise, for those old mother and old father who can be around can feel bad about that when we are sitting close to them they will think that they are carrying a dead person. so that psychology in them will make then worried about this sick but like I think now the Ambulance system is correct because in the past when you call them except the following day before they come to take the body but now when you call they come instantly, because is like we have surveillance teams in the area you just go and meet them and they will call for them to come and take the person. So that is it”.

M: So, what about the Treatment Centres, the Holding Centres or the CCC (Community Care Centre, what are some of the good things and bad things that you hear about them?

R1: “Well, because we had a our friend who fell sick though it was not Ebola but we never knew but you forced him, willingly he went to the Holding Centre but unfortunately there was no bed and space. So what the Nurses did was they told him to wait outside and the bad which was available a patient had died there, the bad where he was supposed to be admitted so that gave cause to run away and they left him outside, so he had to run and he later explained to us. so that one thing, the Nurses did not behave well because as a Nurse you should know that you have to treat people, even if someone had died there you should not tell the person that a patient had died there, even if it were me I will run, that was one thing that brought so much in the community, for instance if they carry a patient and the place is so overcrowded at the Holding Centre and where the people sit is so small but it is better now because now even if they take you there right now you will have your own bed and everything but at first like the Ambulance you are talking about we were having a lot of stories. Like some people when they go to collect a sick person they pump the chlorine on so when such things happen, like most of the patient who came back they told us that it is not like that but when they reach with you and they put you down they will spray the place is not like when you are in the Ambulance because all those suffocations used to kill people but now it is better. Then even the Holding Centre now we are getting report now that they are doing fine is not like in the past”.

M: So what about the Ebola Burial Team, what are some of the good things and bad things that you hear about them?

R2: “First of all let God help us once more. Even to our own eye seeing, bad the way we see the care they take concerning the patients it was bad if not awful. We have seen when they come for a corpse, they will just fling it if the relative of that person is around at the moment you fling his or her relative’s body he or she will not feel good. So that is one of things which is not goo in our community. But the good thing is that now they take care. I have seen where somebody dies the Burial Team will tell you not touch it they will wrapped it for and take it inside, not like in the past when it was very awful”.

M: Ok, yes brother?

R3: “Aaam, just like my brother have said I think the Burial Team was not afraid because to send a dead person that way if the relative is nearer, it does not look fine. But now they take care of the body, they can even call family members to go and bury, they can send about ten member who can stay far away from the grave to see how they ate going to bury their people. Now it is nice but at first it came they were all afraid because they way they were handling the bodies or they would not want to take care.

M: So like this Ebola Burial Team, are you not hearing anything about them apart from the way they handle the body?

R4: “Normally a lot of them come around but we have not witness it yet. Just recently we saw somebody died at (- - name of an area in the same interview district - -) when the Burial Team went there they said they gave them about Le 200,000 (*22feb2015 about 46 USD*) and they allowed the people to go and bury the person. So now there is rumours going around that even the gathering people are involving themselves so much gathering. So if we are all fighting for this to leave our nation if some of these things are taking place it is not nice actually. In the past they were blaming them because of delay, when they call them it will take two to three days before they come the corpse will be there stinking. But now it is better, they respond quickly when you call them come time in two to three hours time they will come and take the person. but because of the rumours that we hear that they do take bribe to allow their people to go and bury them, we should be allowing those things, I think government needs to look into those issues and investigate because if thing like that is going then I think all the effort government and we the people are doing is just in vane, I don’t think the sickness will end”.

Ml Yes brother, have you heard anything about that?

R5: “Yes, I Have heard because I have one of my sisters who works at (- - name of a big hospital in the interview district - -) she told me that they do bribe some people like Le 500,000 (*22feb2015 about 114 USD*) when they give the Le 500,000 (*22feb2015 about 114 USD*) the team will hand over the body to the people to go and bury for themselves, I have heard about that”.

M: What about the 117 Line, what are some of the good things and bad things that you hear about them?

R6: “Well 117, at first the line was slow, it delays, like when you call they respond and take all the details that you are calling for but it will take some days before they could come but now it is better, now you if you call they will send or call their Contact Tracers in the community to come, now it is better”.

M: Yes brother?

R1: “I just want buttress what my brother has just said, now 117 is trying, I say excellent to them. At the time when the thing started the argument was plenty people did not take it seriously but now we praise them and thanks to them for them and let them continue the good job”.

M: What about the workers, those who are working at the hospitals that were there before this time?

R2: “Well initially, when the sick came the way they were treating people was not good to see, if you take a patient to the hospital they won’t attend to the patient at all, the patient will be there for the whole day, some people except will return home to seek medication to ‘Pepeh Doctors’. But maybe is because they did not have any know-how to handle the sick or is because of negligent of their duty brings that about. We have so many people who go there at times except us, the youth in the community, we have been to the hospital on several occasions to set confusion. In fact when you go there they will not even allow you they will tell you if you have a patient, just put him or her there and is not nice. Having cajole somebody to take him or her to hospital, then the person goes there they will not give the patient any attention they will just tell you to put the patient outside, then how do you talk to such person, the next time you will not be able to convince the patient to leave the house and go to hospital because the patient will take it that the same treatment had given him or her will repeated so those were some of the challenges people were facing in the community which they found very difficult for you to be able to persuade somebody, but thank God now with the effort of the media, television and all the rest, in fact some people will now call to tell you that they have a sick person at home come and let us take the person to hospital. Now the tension is less so we are now handling them with care, we tell God thanks for that”.

M: You want to say something?

R3: “The other reason why this sick spread is because of that it was a practical thing which happened and they phoned us we went there. I was going to town at one time, at the junction a woman was lying right on the road and they said let them take her to the (-- name of hospital in the interview district - - ) she was breathing bit by bit. So they talk to the woman and she got up, but like she was hungry. So the woman got up and we decided to go to hospital we forced her to go to hospital, when we reached the hospital gate we reported to the nurses, it looked like they Nurses only valued the Ambulance, when the Ambulance brings you they will take you with care but if you go there by yourself they will ask a soldier man to take you outside. The soldier we met at the gate was the first to tell us not to pass; if we do it was going to be a problem for us. So like I told my friend with whom I was standing that we should leave the area before involve into a problem. Because we under State of Emergency they will never know that we were about to help, they will say is because of lawlessness. So I told my friend to leave the area. So I told my friend that we are lucky not to go into the place there was no space inside even. The woman was there for the whole day when I returned from town and went to the place they said was dead, some say they came and took her. So, them, except a person dies before they take care of them but now that the pressure less you can go to hospital they will take care of you. In November we were getting high cases at that time they don’t even want to know when you mistake - they call a soldier for you”.

R4: “Well like the community but the Doctors who were in this hospital like the Doctor who is here now even when the sick had not come he will not come to the hospital until 11:30 to 12:00 which is very wrong they are public servants; you can hold on to patient until 6 O’clock and you the Doctor could not come to see the person. Supposing that person do not have enough time again? Then you also contributed to kill the person and you are there to serve the community, we have reported those thing several times, we have even gone to (- - name of local radio station - -), we went and told the manager about some of those concerns, the manager even went there to interview him he did not even encourage him, saying that the manager is not the one who him job, he said him and the president went to school. You are there to serve the community somebody goes to see you and you could not come until 11. Government need to improve on those things”.

M: Ok, so do you have any Ebola survivor in the community?

R5: “Yes we have them”.

M: How do people react to those survivors in the community?

R5: “Well in our community, we had accepted them that is why anyone who they release our Councillor will be the first person to go and hug the person for everybody to see in the community we were glad for them and we respect them because we do not want push them so our community do not have any case with that say we are pushing them far, we ignore them no. Right now we used to be together with them, is just that this is not the evening hours you see them coming to mingle with us”.

M: Yes, how does the community react to those Ebola survivors?

R6: “Well like we also have one up there with us, we can be together with him for the whole day we talk, of cause he do explain his experience with the Ebola, he explains to us”.

M: So like which message would you like to give to other communities who are treating those Ebola survivors harshly?

R1: “Well actually we will talk to those people, if anybody who has survived from Ebola and return to the community, if anybody stigmatizes the person let government take action against the person and put them into prison for six months maybe when they come out they will know what to do, they will not talk against them again”.

R2: “What I am saying is this if your blood brother or sister was affected by Ebola and he or she comes back is a big progress, because you got sick you went and came back is a big progress. People have gone, thousands have gone in the three countries, Liberia, Guinea, Sierra Leone and when you come they maltreat you I don’t think that is a fine thing. As for me the only thing I tell anybody is if your brother is sick, he goes and come back what would you do? Are you going to stay away from him or her? You can never say you are going to stay away from him or her. Let us take the Ebola survivors lie our blood brothers and sisters”.

M: Yes my brother?

R3: “I think that is one of the battles. I think that when somebody, let us take it that this is war front if 100 of them goes to war and 50 remain there and your own brother survived and come back I don’t think you will move away from him or her you have to be nearer to him or her to gain experiment from him or her”.

M: So like do you have anything specific that you think peace would like to understand better?

R4: “Well what I think people really need to understand is that about the information. You know that in the past we used to have scattered information but we have concrete one which says “Avoid Body Contact”, do not touch the sick, do not bury the dead that is what people need to know if people did not comply let them find police men to beat them up thoroughly this sick will en”.

M” Yes my brother?

R5: “What I want the government to do is that let them visit the Labs and check them thoroughly or interchange them especially those who are in (- - name of a neighbouring district - -) let them bring them here but let them make sure that the people understand theory job. Maybe they have put somebody there who do not like ( - - name of a neighbouring district - - ) who keeps giving those figures let us know. Just like what my brother was saying”.

M: Yes?

R6” This half baked book learning is creating problem people do not have understanding. Look like this Malaria Campaign, I was part of this Malaria Campaign; we were sharing the medicines. Before going to distribute the medicines, we had cause to sensitize people, met some ladies talking that the medicines which they have brought when you drink it will eat up your gut little by little so in one week you will die. So all these thing were going and then I said to them that it is not true that the medicine had been here before then I told them that if anybody has been sick Malaria if you see this medicine you will know it. But like the information had gone round the compound so people were afraid, even in my own village I called one of mothers and asked her about the medicines, she told me that those medicines they said it is killing people then I told her that it is not true but she told me they were not going to take it. So I think sometimes you can be able convince somebody when the two of you see each other. I think government has to put some of these things in place. Like this public flogging I think it is better. Sierra Leone man, if they beat his brother here all the others will take caution”.

R1: “Look at when the soldiers have taken over, before you say is 6 O’clock everybody will close down shops by the time they say 5:30 everybody will close because they are applying the beating. When soldier come and they meet you before time they will lay you down and beat them and leave them. So I am sure that if they apply that it will be very good”.

M: Ok, I want to tell you all thanks for taking your time to talk to me and I believe that this thing will end in our country.

Rs: “You are welcome.

**ADDITIONAL PART OF INTERVIEW, OBTAINED BY COLLECTOR 5 AFTER CONSENT IN PERSON with two (2) of the original participants, March 2015:**

M: Good afternoon again, in our last discussions, someone said, we the Temne people are very stubborn compare to other tribes, what do you mean?

R1: “Well, at first the Mende people viewed the Ebola sickness as the government want to play politics, but when the Ebola sick really affected them, when they regained their senses and believe that this Ebola thing is a real stuffs, so they put correct measures in place, before when they told them not to wash a person that had died, they did not do it in that strict way, if you looked at the trend of the Ebola confirmed cases we are getting now, the Temne line(=communities) are dominating, that is getting a higher number of confirmed cases and the Mende line (=communities), ninety percent(90%) of the areas are getting lesser number of Ebola confirmed cases, and the Temne line (=communities) have also aware of the sick, and they knew the precautions, but they are still going against the precautions that prevents a person(s) from getting Ebola, we don’t want to just explain more about tribes, but in a strict sense, the Temne tribes are very stubborn, If they have pronounced that , they are going to do particular thing, they will do it, despite anything that may happen”.

M: what about the urban and rural in terms of stubbornness, because some are in the rural areas and some are in the urban areas, there may that difference in terms of stubborn, what do you think?

R1: “Well, it is all about the misinformation people were receiving, if I could tell you, when I talked to any of my family members in village, they will give me different messages, about the many of the things that comes, for example the medicine supplied by medicin san frontier (MSF), our people in the villages thinks that, they are supplying this medicines to kill the people, so they are not taking the medicines because it kills. They are really getting the messages wrongly, we don’t know the group of people that is giving them that wrong messages and they have accepted that wrong information and it is part of them, that is the reason up to now they don’t have 100% belief, is like there are people going around them and passing the wrong information, telling, that when their relative died, let’s touch and wash the dead body, nothing will happen with them, and you know that the education system in the villages are not too strong, so the people are very stubborn”.

R2: “To add to what my brother had just said, we are not discrediting the Temne people, because it is our language, but if you watched closely at the south east, when people going by the measures given to them by the health workers, we have seen that, the cases are automatically dropping, but if we can take a look at the north and western rural, where we normally found the Temne, they got denial, so anything that is happening now, they will say they are playing with the Ebola result, they always say the Ebola thing is not real again, and these are some of the things, when they tell them to do this, they will go on a different way, and do what is not right towards the eradication of this disease, that was why we say the Temne people are stubborn”.

M: In our discussions again, they said “them” wants to politicized this Ebola sick, who are they referring to as “them”, How they want to wants to politicised it, and why they to politicized it?

R1: “May God help us all”.

M: Amen.

R1: “I will not point at any party or tribe, what I am trying to say here, at first, when this things started, they were talking”

M: Well, what I want to know. Who are the specific people?

R1: “If I could looked to the towns where this Ebola thing started, like the Kenema, Kailahun, I took it the main opposition Sierra Leone people party (SLPP) at first took it on political basis, that the ruling All Peoples Congress, party want to reduced their voting populations in their stronghold, where this Ebola thing started, is like they are not realising that the sick is real and up till now we have people that associating Ebola to chemical, that they had brought chemical to kill the people, they don’t still believe that Ebola is real, it is a made virus, but after the whole secret behind Ebola sickness will be exposed later”.

R2: “The thing my brother has just talked, this is not a secret again, when the sick started, it was May 25^th^, when we got the first Ebola confirmed in Kailahun, they said the government wants to take a stance towards reducing the population in the main opposition stronghold as the time for the national census is drawing closer and up till now, we still have the Sierra Leone Peoples party guys, which I normally sat with, who is casting blames on the government that they are politicising the Ebola sickness, even the late Honourable (- -name of person- -) of the Sierra Leone Peoples party (SLPP) that just died, once or twice, when he was on the radio, he was also capitalizing on that, blaming the government, all these instances show that the opposition is very keen on politicising this Ebola thing”.

R2: To make an addendum to what my brother said. I had a friend, the last time we argued bitterly, about the laboratory in Kenema, he said what really they don’t know? They knew everything, the creating of that laboratory (Tulane University),it was an agreement between the people and our president (- -name of president- -), that after the All people Congress had won the election, they will build the laboratory, I said to him, it is a lie, this laboratory had been in Kenema before the All people congress party taken over powers, but yet still people are saying that they came to test chemical in this country, so few people are politicising this Ebola stuffs”.

M: When I asked last, you told me that, there are people that do not still believe Ebola exist, in some particular community. Can you please tell a named community that does not still believe that Ebola exist?

R1: “Yes, this community, the (- - name of interview community - -) community, there are still in this community, that do not believe Ebola exist, even now, they get the high number of Ebola confirmed result these days, people are arguing us bitterly the Ebola has ended, all the results that is coming out now are results of the past, all the result that is coming out now, it is a result given out by the government, to enable get money, that is why they increase the number every day, this community, I am living now, there are still people that do not believe Ebola exists”.

M: Well you told me last that people do not believe Ebola exist, later, they changed their perceptions from not believe to believe, I want you to tell me what happened which led to the changed of perceptions of people and which particular time this happened?

R1: “May God help us all, this happened the time they brought the first surge in the (- - name of interview community - -) community, during the time when President Ernest Bai Koroma said they should carry on the first house to house search, I could not remember the date again, I was among the people that was doing the campaign,”.

R2: “It was in January to late December”.

R1: “That was the time we were moving house to house, to search for sick people, because just after the first campaign of the Medicin San Frontier for distribution of the medicine Artesunate+ Amodiaquine in November, just this campaign, the Western area surge took place, the confirmed cases we got in (- - name of interview community - -) community was not easy and Rukupa community was a hotspot that particular. so that convinced the people, and almost all the people in (- - name of interview community - -) community got to believe that Ebola is real, they are seeing it now visibly, now they are taking person for Ebola test. We even met a person on the height vomiting, the person was saying, please take me to the medical people, so they later tested the person for Ebola, so most them that have survivor are coming around us now, saying Ebola is real, that convince the people that Ebola is real”.

M: Can you remember the exact date this particular changed of perceptions took place?

R2: “Yes, just like what my brother has just said for us to remember the exact date is not easy, but I was made to understand that, it was the time for the first surge that was undertaken, because we the youth in (- - name of interview community - -) community always carried out sensitization programmes, moving door to door, telling people about the Ebola sickness, with the effort of the councillor that funded the programme bit by bit, when the President announced the first surge”.

M: Which type of surge are you talking of that, the three days lockdown or?

Rs: “The (- - name of interview area - -) surge”.

R2: “It was the time when the president declared the Operation (- - name of interview area - -) surge that was the time it happened, we were among the people that was going house to house to bring the sick people outside and escort the sick people to the hospital, there is one woman by the name of ( - - name of the woman - -) she was infected with the Ebola sickness, but she did not survive the sickness, until we started bringing out the sick people, after seen this evidence, they got to believe that Ebola sick is real and if they don’t take their time, it will wiped all of them in the community, they came with the awareness to us, they were thinking that when they are taken to the treatment, they will not come again, either they inject them to die, so when we started seeing the Ebola survivors that again created the awareness”.

M: With these explanation, can you tell me the exact date or if not exact but a range within this thing happened?

R1: “November ending, within that range, around the 20^th^ and above”.

M: Someone measured an organisation by the name of (- - name of organisation - -), I want to know the meaning and what do they did they do?

R1: “I don’t know about that organization”.

M: You said the medicine that they supplied killed people”.

R1: “Killed which medicines”?

M: That artesunate + amodiaquine , some people said this medicine kills them, and they were telling their family members not to take the medicine, why do you think the medicine was killing the people or what is begin the killing?

R1: “I want to believe, it was misinformation of the people, the people that had the know-how of the medicine, misinformed the ones that do not has knowledge in using of the medicine, that led to that saga, we were using the artesunate + amodiaquine for long now, what caused these things, were the things, our people were hearing about the medicine, some people did not learn, so they read and know more about the medicines, in this case, a person will come and talk to the wrongly about this medicines and poison their minds. What led to the death of people, were the people that distributed the medicine, they do not tell the people the actual dosage to be taken, so we found out that, there was over dosage in using of the medicine that caused the loss of many lives”.

M: Ok sir?

R2: “There was a question you asked about the western area urban and the western area rural, let me out rightly, the half-educated people in the villages always create the problem, they always stupid the people in the villages”

M: What do you mean stupid our people in the village?

R2: “Misinformation, if I assumed what I don’t knew better, but pretends that I know, and the people in the village had in mind this man is a learned man, I will take into consideration anytime he or she tells because he or she is educated, these are the same people that have not seen the medicines even before, before the arrival of the medicine they call us on a meeting and told us that there is a medicine that is coming artesunate + amodiaquine, after this, a lady get up shouting, this medicine, destroys the guts of a human being, so I asked her, who gave this information, she said it is like this like that, so these are some of the things, and the person that was narrating the story, she do not had the medicines and I assume a person that knew about the medicine, has told her the about it”.

M: Did she mention anybody that told her this?

R2: “No, she do not mention anyone, she said they told her”.

R1: “They will not mention anyone”.

R1: “I asked her if that was the exact thing she explained that happened , I said no, I knew about artesunate amodiaquine, well the medicine is not strange, as how my brother said what causes the death, was over dosage, it do mean that I had seen someone in our area that had died because of the dosage of the medicine, but we heard rumours that people had died because of over dosage, some people are taken, four tablets at a go, some people think it is Panadol, they took two in morning, two in the afternoon and two at night all of these things led to the loss of lives of people ”.

M: Have you heard of any secret burial or secret washing of dead bodies in this community?

R1: “Well in this community, we the youths are very vigilant, I had never got any information that they had washed dead bodies or burial secretly”.

M: If you were an Ebola survivor, what are the problem do you think you experienced?

R1: “Are you telling about the Ebola survivors”?

M: Well yes, but you are acting in that role, if you were an Ebola survivor, which treatment will expect from your people?

R1: First, you will experienced stigmatization, well frankly speaking, if a person had that understanding, and knew that I am a brother and I had survive from a war and I am back, I thinks they should handle me with both hand and with proper care and encouragement, in community they are not treating them badly, but I heard in other communities that they are stigmatizing the survivors, saying they had the sick and they are survive, but I thank God now everybody had the awareness that they should not treat survivors badly, they should encouraged them”.

M: Putting yourself in that position, after surviving from the Ebola sick, what is the treatment do you expect from your people?

R1: “I will think plenty, I may think that people will dissociate themselves from me, the love and embracement I was getting from friends, the friendly company we were getting together and other family members, I will not get that type of love again, people will be afraid of me, these are my expectations”.

M: Ok, have you heard, seen a person that had sick of Ebola, before he or she go to treatment centre, they may prefer going to the traditional healer, can you why they go there and for what reasons?

R1: “Here we don’t have cases like that, because we were moving house to house to search for sick people, when they discovered sick person, they will take the person to the hospital, that gave the conviction to people that, this sickness is real, because within search, when they tested more than fifteen people proved positive after the twenty –six cases they had, so with this when people are sick, they will voluntarily go to the hospital, because they knew that, the earlier they go to the hospital, the greater chance they had to survived, we had ever experienced issue like that in our community”.

M: So nobody had done that?

R1: the only thing was, from the story how the thing started, somebody that was ever thinking she had ever Ebola went to deliver, she went to the traditional birth attendant (TBA)”.

M: What is the meaning of that?

R1: “Basically I don’t know the meaning of the term, but I knew they are the sowies (= important member of the bondo secret society, involved in initiation), these are people that heal other people, and help in the local delivery of pregnant women, I guess there is a meaning for that, I don’t know exactly, but they are also healers, and act as Traditional birth attendant, they pregnant women went to her for delivering, the pregnant woman was an Ebola victim, she had already died, these are just built stories, we don’t issues on that here, in fact after that incident, we youths were moving up and down, to ensure it reoccurrence and the sowie mammies joined us in the drive, they said they will not do it again, so they totally desist from the act”.

M: Anybody may want to add anything?

Rs: “No”

M: I thank you very much.
