# Supplementary material for: Development of a set of community-informed Ebola messages for Sierra Leone
Source: PLoS Negl Trop Dis. 2017 Aug 7;11(8):e0005742. doi: 10.1371/journal.pntd.0005742 (PMC5560759; doi:10.1371/journal.pntd.0005742)
Supplement: S1 Appendix — (ZIP) [file pntd.0005742.s001.zip › Ebola messages - FGD and interview transcripts/R2HC Ebola Fieldwork 1/R2HC Ebola F1 HW-Rural1 V3 CORR.docx]

| CODE | **R2HC Ebola F1 HW-Rural1 (rural semi-structured interview with health staff and health volunteers)**  **V2 – 2^nd^ March 2015 – Probing added**  **V3 – 11^th^ March 2015 – Correction personal data correspondent** |
| --- | --- |
| DATE | January 2015 |
| DURATION (minutes) | 38 |
| Collector nr | 5 |
| LANGUAGE INTERVIEW | Krio |

**PERSONAL DATA RESPONDENT**

| Age *(in whole years)* | 42 |
| --- | --- |
| Sex (Female = F, Male = M) - circle | Female |
| Religion | Muslim |
| How much time does it take you to walk from your house to the nearest PHU? (minutes) | 30 |
| Mother tongue: | Temne |
| Role in the health facility / health: | XXXXXXXXXX |
| Education level (circle) | Secondary |
| Do you know anybody who had Ebola? | yes |
| If Yes, what is your relation to that person? | family |

**TRANSCRIPT:**

M: Can you tell me your first time you hear about Ebola?

R:”First I heard it from my child, when my child came back from school, my child said Mama, my teacher is telling me every day that there is a sick called Ebola which has come, and they said when a person contact this sick, it will kill the person. They said we have to wash our hands every day, anything we do we have to wash your hands. They said we have to come and tell our parents, we should be washing our hands, anything we do, anywhere we touch, that we are not satisfied, let us wash your hands. Then I told my child get out from my face, every day you are telling me about this sick, which kind of sickness I have not seen or heard about, when you are just telling me about. But everyday telling me about it. So the 23^rd^ of July we finally got the sickness at (--Name of interview village--), one (name of the person) that brought this sick in our community”.

M: As you were told by your child and the way you have heard of it, can you described how Ebola does show itself?

R:”My daughter told me that when a person contracts Ebola, blood will hose out from the nose, mouth, ear and anus and you will get rashes all over your body, when you scratch the rash blood start hosing from it.my child said you will experience headache, “warmbodi”(high body temperature, fever), you will get “runbele”(frequent stooling), vomiting. I said get out in front of me every day, your stories that will discourage someone, but my teacher said I must be reminding you about it when I come home, I will not get tired of saying it to you”.

M: so that is way you were thinking, that all about Ebola is nonsense?

R:”Yes”

M: can you tell me how Ebola has affected the –Name of interview village-- community?

R:”uhmmuh”(a deep breath) Yes, “Boku”(plenty)

M: Have you seen or known of people that have Ebola in this community?

R:”Yes, because the first person which I told you about (name of the Person), he was bleeding through the gum, they took him to one herbalist at (--another village in the area), they were not able to cure, they carry him to (--a bigger village in the area--) also they were not able, he end up to die, they buried him. I was in a workshop with our CHO (community health officer) in one primary school, when they pass with his dead body, then I told him (name of the person) the dead body that they are passing with, the person was sick, bleeding from the mouth and gums, they said child was taken to the herbalist but could not survive. So I did not know who call at the primary health care, after have bury two to three, so they came and said they have to quarantine that particular house, people started to “Baranta”(out of control) in this village, so the section chief try his best to talk for them to be quarantined. So when the medical people were asking who washed the dead body, who was the sick, they were afraid, nobody answered. Everybody was saying it is a lie, it is not Ebola”.

M: Since the outbreak of Ebola, what has really happened towards development in this community?

R:”The development has moved back, as they have quarantine XXXXXXXXXXX (the district), the movement of people has been resisted, everybody should stay where they are. Even the market woman were grumbling that no flow of buyers in the market, commodities are just increasing, for instance an item that they sell at five thousand Leones, now is ten thousand Leones, when you ask they said they are travelling again to buy at whole sale, so the once risking their lives to go and buy in bulk sell it at higher price”.

M: so will you tell me why you think Ebola had spread all over Sierra Leone?

R:”Yes, there was no believe that it Ebola. A sickness that destroys family, spoils friendship, we were denying, there was no believe, instead we were believing the native saying that it was “witchgun” (a gun of a witch), and it was so and so, that person was “fankay”( fire with the witch gun), so when have seen people dying in our community and they quarantine, that is the time we came to believe that Ebola is real and it exist”.

M: What do you think is the best way to prevent Ebola from spreading?

R:”First, the way the president has done, putting stop to movement, you should not go to another person’s house, you should not touch, if you visit a person, stay out and talk to the person, when you place your hands at a place you don’t satisfy, wash it with water and soap or chlorine”.

M: what do you think is the best way to treat a person with Ebola?

R:”The best way is earlier treatment, because hiding and being afraid will not cure you, instead it kills but when a person is sick, report at once that you are sick, so they will take you for an earlier treatment, when you walk to go, you will also walk to come back but if you wait and allow ”wata pas gari”( the sickness to worsen), then ambulance will come and take, you may not know, if you will come back, maybe the ambulance will take back for burial”.

M: Do you have any name for Ebola in your local language?

R:” some says “unkrobola, unbola”.

M: What do you mean by that?

R:” it means Ebola

M: Some people believe that Ebola do not exist, do you know those people in this your community, and why do they think so?

*(Sound of a motorbike)*

R:”As God could do it, most of the people that were having this belief, were the people that were affected by Ebola, some die, some are still alive, denying, that was what we were doing, when there is Ebola meeting, they will say make them leave us alone, they want to just eat money, there is no Ebola, it was a crash of “witch plane”, six hundred thousand people were in the plane, so these are the people that are dying. This plane was travelling to a destination and was loaded with people all over Sierra Leone so on its way it get crashed. So there is no Ebola, they are just lying”.

M: Can you give some examples of Ebola messages that you have heard, seen or read, what do they mean?

R:” Yes, they told us that we should not eat bush meat like bat and monkey, and remaining food of other animals, but some people are still denying, like a man that died here, he was staying in this community, he went for work at (--another village in the area --), they said a baboon was killed and they were selling at two thousand Leones, so he purchases and eat, he then came to the (--Name of the interview village--) centre sick, we did not allow him to enter inside, they started saying that he came yesterday, not knowing that he was here two days back, within this two days period, he fights the sickness but no way, so he decided to come to the centre. He was showing the symptoms on his face and body, his eyes were red, having “warmbody” (high body temperature, fever), hiccupping, he was not able to walk, so he returned back home, so there was a boy who lost his sister through Ebola, he was visiting the quarantine house, later he went and took the beddings of his sister and lay on it”.

M: So one of the message you heard is avoid eating bush meat?

R:”Yes”.

M: Have you heard of anyone again?

R:”No, the one in this community is the one I knew of, the man from (-- the name of the other village mentioned above --)”.

M: What I mean here the messages you have been hearing, like you just said, to avoid eating bats, is there any other message to prevents you from getting Ebola?

R:”Yes they are “boku” (plenty)”.

M: Like which one?

R:”You have to wash your body every day and clean your environment where you live, clean the toilet with soap and water or you use chlorine”.

M: Among those messages, which one you think is the best to practice, to prevent people from getting Ebola?

R:” the best way is hand washing, don’t touch, when you visit a person’s house don’t enter and don’t sit down”.

M: So which of these messages you think have not worked so well to the understanding of the people?

R:”like some people up to now, when they visits someone’s house, they must sit down and some people when you ask them to wash their hands, they will ask you, are you giving me food to eat, so I will tell them that please go and wash your hands, Ebola sick is not a joke have you seen the way it has killed people, hand washing is the best, so we will crack jokes then they will later wash their hands”.

M: So what you think would be the good message to encourage people bring their patients to the hospital for treatment, which message will you send to them?

R:”So we will tell them not to be afraid, like in (--Name of the interview village --) Village, we have the CC (Community Care) centre so let them be afraid”.

M:”So what is the meaning of the CC centre?

R:”Community Caring Centre, you will go first to another Centre before you are refer to the Community Caring centre, after screening you at (--Name of interview village --) community centre, with the symptoms you are showing they will transfer you to the community caring centre, we will also say to them don’t be afraid we are all the same people here, that is why they did not come with another extract person so don’t be afraid to come out if you are sick, we will give you treatment, but if you did not improve after the treatment, we will call for ambulance to take you to Makeni, they will also check you, if you are having malaria they will treat it for malaria, if you also have Ebola it will be treated also, so come earlier for treatment”.

M: In the event of Ebola infection, do you think the person will prefer to first to go a traditional healer, to come to (--Name of interview village--) health Centre, where you think the person will first go?

R:”In my own sense I prefer the person to come to the hospital, because only the medical people can cure Ebola”.

M:”Yes, what I am saying, in event of a person getting Ebola infected, they will think otherwise, either to visit a traditional healer or the Hospital?

R:” Some before now they first visit the “Medicine man” (traditional healer), when the traditional healer did not able to cure, that time they will come to the treatment centre”.

M:”Some people decide to stay home when they think they have Ebola, why do you think they are doing this, what is the message to encourage them to come to treatment centre?

R:”I usually tell them that you should not keep sick until you die, I will tell them that if you were taking paracetamol for headache or “warmbodi”(high body temperature, fever) then you get better, if you take it today and tomorrow again there is still no better, go to the hospital, the will go and describe the type of sick you are having, so staying at home sick is not good, get up and go the hospital”.

M:”What do you think is best way to pass on Ebola messages, because you are hearing them through radio, Airtel sent you text messages, on newspapers on Ebola, so which of these channel is best, which takes the message to the people?

*(Voice at the background)*

R:”Because some people are really difficult to deal with, you go and advise them, but they will not listen, so if you know people like this, you talk to them once twice, the only way is to call 117, that this so and so person is sick, he lives at this particular street in a this particular house, he is sick but don’t want to go to the hospital”.

M: Like what am saying, you are having your phone and you usually received message or text on Ebola, the Radios are talking about Ebola, they even have song, they have produced, and the newspapers are also talking of Ebola, of all this channels which one prefer to be the best if used, from the message to reach the last man in the village will hear and get the Ebola message?

R:” You know the best way I usually it when we had meetings, like our people here they will see this Ebola killing all of them, for instance the other countries, where Ebola had being affected before, like the hospitals also, let them play the films (documentaries) in the communities, people will see what have been happening and get more beliefs, because they have talked on radio, bring different messages everyday but I said if they watch this through films they will believe and know that Ebola is real”.

M: Okay, What is the good or either bad way you have been hearing of the Ambulance service?

R:” Before now, someone would have agreed to go to the treatment centre, but when they hear the “noise” (sound of the siren) of the Ambulance when it comes, they change their mind that I am not going. They said they don’t like the noise of the ambulance, has they hear it, they become afraid and if somebody is suspected of Ebola and refuses not to enter the ambulance, you don’t have the right to touch the person. The noise of the ambulance is the problem, but now when there is no “noise” (sound of the siren) they will willingly enter the ambulance compared to before when there was “noise” (sound of the siren). Some people said don’t want that sound blowing on them”.

M: So what about the Ebola treatment centre/community centres, what are the good things about them?

R:”Really the Ebola treatment centres are trying, if you have those symptoms and go earlier, they will must treat you, but if you waste time for the “wata to pas gari” (the sickness to get worst), maybe only by the grace of God that you survive, because he is the owner of our lives, some will survive and some will not survive at the Ebola treatment centre”.

M: What about the bad thing about the treatment centre have you heard anything bad?

R:” Well “boku” (plenty) of the survivors explained to us when you are sick and taken to the centre, doctors and nurses will not touch you either talk to you, instead when they have medicine to give you, they will just throw it on you, if you are able to get up is okay, but if you are not is your own business. Then food again, they said there was no frequent food given. Unless the ones taken to Kailahun, there they eat three times a day, but at the holding centre they were not treating them good, they don’t give them enough water, and they just spray them and they said they don’t want the spraying, even the ambulance, when they about to go, they spray and the ambulance starts to “hala” (sounding the siren). So the spraying, the sounding of the siren of the ambulance, the holding centres, these they were afraid of, they said when you went, there is no body taking care of them, when they want give you medicine, they will just stand at the door and send the medicine and water to you, if you are not able to get up, you will remain there and die”.

*(A voice of a fowl)*

M: What is the good thing you have been hearing about the Ebola burial team?

R:”first before this time, they were not treating people well, but now when they come, they will only spray where the dead body is laying inside the house, and they will ask if the person is a Christian or a Muslim, and they will show the religion they belong, the people will also give them “casankay”(white sartorial used to wrap the dead) to wrap and put the body in the plastic, they will also give the people chance again to pray for the dead person but they will stand at a distance in the process of praying, they will also allow some family members about two to four to follow them at the cemetery to see how they are going to bury their family member. First they do not allow this but now they allow”.

M: What about the Ebola 117 phone line, what are the good and bad things people talk about them?

R:”Haaa”first before this time, it was a problem, they have abuse, swear me in this town, saying when Ebola finishes I will know them, they said they will do me this, they will do me that, I did not even care about them, because I know I am doing the right thing. If I see that you want to bring “Nabara” (trouble) in this community I will call them and I was not the only person calling, so first they swearing the callers, when so and person sick person, and they hide person. So they will say the person that call to come and collect this person, they are going to swear, because when they are going with sick person, they are going to kill person, the person is not coming again, so they were afraid and they will “baranta” (be out of control) in this town”.

M: Like when you call the 117 phone line what are the good things they do, and the bad things people talk about them?

R:”( *voice of a fowl*) Because before when “boku”(plenty) people die, and there was not enough ambulance, so when they call them, to come and collect the sick or dead person, they will not come until after two days or when a person get sick, so people were grumbling about this act, unless now when there is enough ambulance provided by government, so when you call them now they will respond at once to collect either the sick person or dead body”.

M :( *sound of a vehicle) (voice of the fowl*) How do people treat Ebola survivors in this community, do you know any person that have survive from Ebola in this community?

R:”Yes sir, we have three of them now in this community”.

M: How do people treat them?

R:”Well the one that went to Kailahun, whom is (name of the person), when she came back, she explain that she really suffers when they carry them, she said they were eighteen in number, and among them she was very hopeless, she said the others were stepping and kicking her, and on their way they experience a lots of “gallups” (dykes). But thank God, they were treated fine”.

M: Okay, they were treating them fine, but I want to know how you people in this community treat them, when they have gone and come back as survivors?

R:”We will take them as our brothers, sisters, our mothers and fathers, we will encourage them back, because we have been told that they do not transfer the sick if even hold or touch you , we will encourage them as our brothers and sisters because they do not transfer the sick”.

M: Which message may like to give the other communities that do not encourage survivors, to be talking and encouraging the Ebola survivors, which message you may give them?

R:”We will give them message that please those Ebola survivors that have come back, you should take them as how you have been together before, and you don’t need to point your fingers on them, like when they are passing, then you say, “haa”, look at that person going he/she was having Ebola, the person was taking to the treatment centre now the person has been healed and the person is back, these survivors don’t like that type of behaviour, because they know the way they have suffered. I said there are bye- laws she has been put in place that we should not do this type of behaviour, by not encourages and stigmatizing/pointing hands on this survivors, if you persist doing it, until the person go and report that this person living at this particular address, is pointing hands on, provoking my situations as survivors, well you will be in big trouble with the government. As they have sick and be cured and they have come back, they can still help us in our community. Like for instance when a person is sick in community, and they said we should touch sick people, you can call the survivor to help you. So the sick person may not feel bad that when he/she is sick no person did not come near. This Ebola survivors are our soldier in this Ebola sick, even in hospitals they may go there to be giving helping hands to the community people that have become sick, as they say we should not touch sick person we have to be far away, so the survivor will be there to help in the community”.

M: Have you heard of any new treatments for Ebola that may become available soon?

R:”Well they have been saying that they have “mek” (produced) vaccines for Ebola, but up to now we have not yet seen it, but we have been hearing about it, they said it is going to be tested first in Liberia, I think it has not yet gone through, but we heard that when they finish making it they will come with it”.

M: What you’re thinking about the medicine you just talked about, that it is coming soon, it will be fine?

R:(*somebody shouting at the background*)”Yes it will be fine, because any medical person that has said he/she is making this, the person has tested it first before giving it out to any other person, so I think that when they medicine comes it will be fine, like how they have come with the malaria treatment, many people are not getting sick again, because “boku”(plenty) sickness before now was malaria and typhoid that was “Hambug” (disturbing) most of our people, which they were not going to the hospital to know the type of sick that they have, that is why they were just dying”.

M: Ok, so what your concern to your people about this treatment/medicine?

R:” I will be glad the time that medicine will come, because like the under-fives, when they described “marklate” (vaccines) for them, I believe that you the “kombra” (suckling mother, breastfeeding woman) begins to take this vaccines after given birth newly from one month to nine month, as it is happen to the children now, they are not getting whooping cough again, measles again and other sickness will not come their way anymore, but when you decide not take “marklate”(vaccines), like some children when you look into their vaccines card, they will not complete taking their “Marklate”(vaccines), that is why they are getting different sickness”.

M: Have you heard of any new ways to prevent Ebola?

R:”Well the only way, they said when someone had survive from Ebola and have come, if that is even your husband, wife, boyfriend or girlfriend, you should not get sexual intercourse with that person for three months, you have to prevent yourself for that three month, you don’t need to sex, you have preserve yourself”.

M: Assuming as a health volunteer what do you know about Ebola in your community, what you feel you need to do, so that your people, the once that misinterpret the Ebola message, will get the message very well, what you think you have to do has health worker?

R:”As how I was just talking, if you have volunteer to do something, if even they “cuss” (abusive language) you, swear you, whatever they told you have to just accept/bear, when you accept/bear you must do something your community. So still as they are talking plenty, abusing me, saying that they will swear me, even some called me “black catchon ,longsleeve, blackplastic” when am passing, and even “cuss”(abusive language),swear me, but I have volunteer that am going to me a slave for my community, I was still doing my work, going for house to house, telling that what they are telling us about Ebola, let please take it, for our lives to be become longer, if notice that your child is sick not hide, a family member is sick don’t hide, take the person to our centre, we are not able to treat the person we will call other people to help. I said you that woman (name of the person) when they were taking her to the centre, you people were saying is going to die, but she return back alive, so please we will not take you here to go and kill you people, if you have the hope that when they are carrying me, I will cure and come back, that is the hope, we should be having, don’t hope when you are sick and they take you, you going to die. You should hope to God that am going to get medicine and be cure, don’t be afraid, if you are sick just call me I will make you to come to the centre, don’t be afraid, if you are ashamed to talk, just call me, that is why you choose me in this community, you will explain your problem to me, then I will further explain to the Community health officer or nurse. So please let don’t be afraid of that clinic is ours, and they are complying with us, because pregnant woman were not coming to the clinic before, but now due to house to house talk, they are coming”.

M: About this Ebola, what you think people need to understand, how would you explain for them, to understand to better?

R:”When we were doing the “health tok” (health talk), we will ask, who is here that speak krio and understand, some will say let talk in Temne, is the language we hear and understand. Sometime we talk krio if they don’t understand we talk in Temne about Ebola and we will tell them, let the message don’t remain here, anyone of must go to house or village to spread the message”.

M:” So the best way is when you talk in language they understand?

R:”Yes”.

M: Thank you very much for giving me your time for this interview

R:”Thank you”.

**ADDITIONAL PART OF INTERVIEW, OBTAINED BY COLLECTOR 1 AFTER CONSENT IN PERSON, February 2015:**

M: Initially people were thinking that Ebola is nonsense, why do they think that Ebola is nonsense?

R: “Because we black men if do not see something we will but believe”.

M: What do you mean if they not see they will not believe, what is it if they did not see?

R: “Because when they were just shouting Ebola, Ebola, when they were taking people to Kailahun (=first district in Sierra Leone with Ebola cases) they vomiting, toileting blood with rashes on their bodies and feeling fever, because for us here because some of them did not see those signs they do not believe”.

M: Why is it that other people do not believe that Ebola is real?

R: “Because, I take it to be lack of understanding”.

M: Lack of understanding?

R: “Yes, because those who understands and are supposed to teach the people they supporting those who are illiterate. They tell them that it is a witch plane that crashed over there or they did it to him or they ‘Fankay’ (fire him with witch gun), that was the belief that had, they said because if they fire a person with a witch gun the person can vomit blood, toilet blood and have running stomach, that is what they believe they say it is a witch”.

M: But is this still existing right now?

R: “No”.

M: It has changed now?

R: “What do you think is the reason for this sudden change?

R: “Because they are teaching them regularly, when this one comes he talks about Ebola and go and then the other comes and talk and they are now seeing, like for us here we have seen many who have died. So even some who were in the quarantined house were denying but when they saw the way people were dying then they believed, when they died up to five, when this one sick today and them the other one died then they believed”.

M: What is the total number of people who died here?

R: “Well around eighteen people”.

M: Ok,

R: “It was thirty two people”.

,M: Ok,

R: “Only two survived”.

M: Ok, you told me about an Ebola patient who they took to the herbalist; I just want to know who carried him to the herbalist?

R: “”Well, because me I did not see, oh no yes the first case”.

M: Yes the first case.

R: “I remembered the first case when they were denying here”.

M: Yes.

R: “So they first took him to (- - another community in the same interview chiefdom- -)”.

M: Ok,

R: “Into the Poro society (= men’s secret society), they took him from there saying that it does not belong there, then they brought him here to one herbalist where he died and they came and buried him it was the time they suspected that it was Ebola, because they first took him to government hospital”.

M: Ok, who was this kind of person?

R: “Yes, he joined the Poro society (=men’s secret society) ”.

M: Ok, so why did they carry him to the Poro society (=men’s secret society) bush?

R: “Because they thought he had fine to pay and that is was affecting him”.

M: But what do they mean by this ‘Kassi’?

R: “Because if you have ‘Kassi’ somewhere, if you don’t have the money they will punish you, because we see how they fine them, they take them to the Poro (=men’s secret society) bush so we do not know what they do to him”.

M: Ok,

R: “But we can see in the town when they tie them”.

M: Ok,

R: “So they felt there is where he might have involved and got ‘Kassi’ somewhere. They took him there they said that was not it after they removed him from government hospital they carried him to (- - name of another community - -)”

M: Ok,

R: “They carried him today and he died in the evening”.

M: When he died what did they do to him?

R: “They buried him, later,..”.

M: What happened to the herbalist?

R: “Till now we have not heard anything from (- - name of the last community they took the person to - -) that anything has happened to the herbalist, or he is dead or had been sick, we have not heard that who were the people that took the decision to carry that man to the herbalist?

R: “His father but he too is dead”.

M: He too is dead now?

R: “Yes, only his mother survived when they carried her to Kailahun (=district in Sierra Leone with first Ebola cases)”.

M: Ok,

R: “Because when that boy died they buried him ‘sumumu’ (Secretly)”.

M: Secretly?

R: “Yes, they buried him because at that time we were hearing about Ebola but then we had not seen it”.

M: Ok,

R: “Because they said he was bleeding through his gum and his mouth”.

M: Who is that?

R: “The boy who died was the one who started Ebola in this (- - name of the interview community - -), so the grandson of the father also died and they buried him quickly and many people did not know, so when the father died then they decided to report. And call 117 to say that the old man who was denying that he does not have Ebola and his son does not have Ebola has died and in the same place he is the number four person. Because the two grandsons died, then the boy who brought the Ebola there they came to take note that the thing is Ebola that was the time they quarantined the place”.

M: But why were they burying people secretly?

R: “Because they were afraid of the quarantine because they said when they quarantine you the food which they bring for you Ebola is among and that when you eat it you will all die, that is what they are afraid of, even when they take a person to Kailahun (= district with first Ebola cases in Sierra Leone) they will say they are going to kill the person. Because when they go with people they will just pump the chlorine on them and it is that chlorine that makes people die. And they also think that when they take you they are going drain all your blood from your body.

M: But who are the people who are spreading this?

R: “Only they know because they there was one man who was in the Lab in Kenema (=second district in Sierra Leone with Ebola cases) when they take the person there he would say give me Le 30,000 (2mar2015 about 7 USD) for me to make you Ebola nonnegative but he was caught and jailed. That was why people were afraid, that when they carry a person the person will not come again, they will not know the whereabouts of the person, they will not tell you whether the person is dead or alive, that was what they were afraid of”.

N: Ok, Why were people afraid of the spray?

R: “Because they said they spray kills, it is killing them in the quarantine home”.

M: This spray, which type of spray is it?

R: “They said it is chlorine is too much, they way they mix it is too much now, so that used to affect them when they die in quarantine homes. That was what they were saying, those who were in the quarantined home to those who are out”.

M: Ok, if somebody is an Ebola survivor, what are the problems that the person encounters?

R: “Well the woman who survived and came back, she was saying that when she came back newly she was not seeing properly and do not hear fine, when I used to ask her that is what she used to tell me but now she says it is better is only the ears have some problem”.

M: But how does the community respond to those Ebola survivors?

R: “Because right now when the Community Care Centre is here the two survivors are there helping”.

M: They are there to do what?

R: “They are there to help and they give them small thing”.

M: Do they pay them?

R: “Yes, because there are some cases that come, they help treat the patients”. ”.

M: But how does the community respond to those Ebola survivors, do they stay away from them?

R: ”No, no, no”.

M: They do not stay away from them?

R: “No, they also encourage them, they do not run away from them’.

M: But when you share boundary with (- - name of neighbouring chiefdom - -), how do they look at your survivors?

R: “Because when they brought them they made laws that nobody should laugh at them and they catch the person he will go to jail so they do not laugh at them. Except that woman who told me that they are disturbing her at the house where she is, because they just go and lodge them there. Sometime they always tell her that it was her son that brought the problem here and now all the families are dead”.

M: So they tell him those words?

R: “Yes”.

M: Who is that woman to the boy?

R: “She was just lodged there because they share the same boundary, their own house collapsed and they decided to lodge them”.

M: Ok, so those who lodged her are the ones saying that it was her son who brought the problem?

R: “Yes”.

M: So who did she say they look at her now? How does she feel?

R: “Well we do courage her but she says they always discourage her, we tell her not to look to them because all things they are saying if you go to any police station it will be problem for them, so we tell her to bear. Sometimes she goes to the town chief and complains, even some of the food which they bring for them they would want to eat it without giving her sometimes they only give her small except she had to call the town chief to come between them and share the food. They had almost driven her from the house”

M: Drove her?

R: “Yes and the same boy in the quarantined home who used to tell her those words was the same who chased her with cutlass saying that he was going to kill her if she stays in the house. It was that night she left the quarantined home and went to complain to the town Chief and they came and talk to them to leave the boy alone. Some journalists came around and took statement to say if anything happens to that boy they will not take easy with them and that when this Ebola finishes, they were going to appear court.

M: So, this situation do you think is the same in other places?

R: “Well because the other child which they say his friend return after she was cured they advise him to wait for three months when she came, her people use to get vexed but when they brought them from (- - name of community - -) they came and showed the laws to her people that once somebody had come that, they were heroes, they would not like to remind her about what has passed, they should not drive her, they should not shout at her, they should take it that she is still your daughter and you should accept her, so onto now we have not heard anything from that and they are encouraging her. Except her mother who had told some words, because when she was sick they took her to the mother side. So till now the woman is there but she said the words which they have told her she is not going to return again to her husband and that let her people find another man and that she is going to rest to her people”.

M: The woman said she was not going to return to her husband again?

R: “Yes, she said she is going to reach to her people”.

M: That is a quarrel now?

R: “Yes because at the time when they quarantined the two homes both the mother and the Father side, when the mother left the mother side after treating her son she went back to the father side, so they said because she left the other side and went over to the other side, when she left here they gave her Dettol, chlorine and everything she even laundered her clothes before entering the house when she entered the husband said she should not enter the room she slept at the parlour until the following day when they came and quarantined the place, so they said there is no need to quarantine the place, they should quarantine where she was staying when she used to toilet and vomit there is where they should quarantine”.

M: Ok, that was just wanted to know. Thank you very much Ma.

R: “Ok”.
