# Supplementary material for: Development of a set of community-informed Ebola messages for Sierra Leone
Source: PLoS Negl Trop Dis. 2017 Aug 7;11(8):e0005742. doi: 10.1371/journal.pntd.0005742 (PMC5560759; doi:10.1371/journal.pntd.0005742)
Supplement: S1 Appendix — (ZIP) [file pntd.0005742.s001.zip › Ebola messages - FGD and interview transcripts/R2HC Ebola Fieldwork 1/R2HC Ebola F1 HW-Rural2.docx]

| CODE | **R2HC Ebola F1 HW-Rural2 (rural semi-structured interview with health staff and health volunteers)** |
| --- | --- |
| DATE | January 2015 |
| DURATION (minutes) | 16 |
| Collector nr | 2 |
| LANGUAGE INTERVIEW | Krio |

**PERSONAL DATA RESPONDENT**

| Age *(in whole years)* | 25 |
| --- | --- |
| Sex (Female = F, Male = M) - circle | Female |
| Religion | Muslim |
| How much time does it take you to walk from your house to the nearest PHU? (minutes) | 2 |
| Mother tongue: | Temne |
| Role in the health facility / health: | XXXXXXXXXX |
| Education level (circle) | Tertiary |
| Do you know anybody who had Ebola? | Yes |
| If Yes, what is your relation to that person? | Patient |

**TRANSCRIPT: (M= Moderator, R=Respondent)**

M: When did you first hear about Ebola?

R:”At first we heard of Ebola the 25th of May”.

M: How did they tell you about this disease?

R:”They said it is a dangerous deadly disease which kills and does not “pic” (select) anybody, it is very dangerous and it kills”.

M: What were your first thoughts about this sick after you have been told, what came into your mind”

R:”We as health worker, at first we did not believe now we believe that sick is real, some people were saying it is witchcraft, others were saying it is a “witch gun”, so and so, that was our first thought but of this Ebola, deadly and dangerous”.

M: In which way Ebola has affected this community?

R:”It has really affected this community, because here is the place we got the first positive Ebola case, at the (--Name of interview village --) community, it really affected us here, it kills a lot of people, it even kills nineteen people in one house, first house that was quarantined, and the next house it kills two people. And it came again this last time but did not kill, because the person that was affected and was positive, went to the treatment centre and came back survived from the sick”.( *voice of the fowl*)

M: Have you personally seen or known a person who had Ebola?

R:”But really when the result were not out, but I have seen a person personally that has Ebola, though we were not in the knowing, but the way the person explains the signs and symptoms that shows coincide with the signs and symptoms that was said to us by the “masta sabi people”(experts). Later when the result were out, it was positive, so I have seen an Ebola patient”.

M: Why do you think that Ebola has spread throughout Sierra Leone?

R:”Because people are not taking what the “masta sabi”( medical experts) the rules and regulations they told us to do and the denial has caused it to spread, if people were not denying, they accept and at least they will take to what the “masta sabi”(medical experts) has told us to do, don’t touch people, don’t go to people’s house, bury dead bodies, that alone they don’t accept and believe and they were not taking the rules and regulation, that is why Ebola has spread”.

M: What is the best way you think to prevent Ebola?

R:”The best I think is, I should be washing my hands frequently, I have to avoid body contact, I should not touch people, I don’t need to go to people’s house, I have to avoid treating sick people as a medical personnel, if ever I am treating people, I have to be careful always use my PPEs (Personal Protective Equipment’s), I don’t need to bury, don’t touch dead corpse”.

M: What do you think is the best way to treat a person with Ebola?

R:”Well the best way to treat a person with Ebola, for we the medical personnel, though some have gone for the training, some of them are trained to treat, some of us were trained not to treat but to give at least a first aid treatment which is ORS(Oral rehydration salt) then later call at the treatment centre or we refer to the treatment, they will come and collect , but really we deal with this ORS(Oral rehydration salt) and give them”.

M: In this community do they have any local term to call Ebola?

R:”No, it’s just Ebola, no local term”.

M: Are there people in this community that do not believe that Ebola exists?

R:”Right now to what has happened in the past, I believe that everybody believes Ebola exists and it is real”.

M:”The Ebola Messages you have heard or seen, what do you think of them?

R:”Well it is a very good message, it makes us to learn, at least to do, what we are supposed to do, and don’t do what we are not supposed to do, it helps a lots”.

M: Which ones you have been hearing, or which one you are hearing?

R:”To wash our hands, to go to a person’s house, don’t wash dead body, don’t travel for burials in other towns, they are really plenty”.

M: What are the best Ebola message you have seen or heard and why do you think is the best?

R: ”The best Ebola messages have seen or heard are, hand washing, avoid body contact, avoid washing dead corpse, and make sure your environment is clean always, it is best, really it is best because at least it help us, it makes plenty of us to practice personal hygiene now and our people are practicing personal hygiene, even we the health workers, the way of handling gloves, we were using one glove on many patients, but it has really helped, now is one glove to one patient after which we wash our hands, so we practice hygiene”.

M: Which of the message do not go down well with the people or is not the best, or have not worked so well?

R: ”Well all the message are the best, I take them as the best, because they went down well, people are going according to the message and are working towards the message.

M: So there is none of them that do not worked?

R:”There is none of them, all are the best”.

M: What you think is the best message to encourage people to bring patients that are infected with Ebola?

R:” Well we will talk to them, we will go and sensitize them not to be afraid, though the sickness exists and it is real, if you have a sick person don’t hide the person, if you hide the sick person, it will create more problem and continue to spread, but if you report earlier, the earlier the person receives treatment and will survive”.

M: Ok, in the event of Ebola infection, where do you think people will prefer to go, to a traditional healer, hospital or Ebola treatment centre?

R:”At least let the person come to the hospital first, to seek medical people first”.

M:”So you think, they will go to hospital first when they get Ebola infection?

R:”Yes, than they prefer to go “morayman” (sorcerer), let them come to the hospital first”.

M: Some people stay at home when they think they may have Ebola, why do think they stay at home?

R:”Well, some are still afraid, they have fear, and they are still getting the fear towards people’s perception that when we go to the hospital, they are going to kill us, the medical people will kill us, but thanks to God now people are not afraid, some are coming, plenty of them are coming also to the hospital, they were not coming because they were afraid”.

M: So what do you think you have to them to encourage them to come to the hospital?

R:”Well still continue to sensitize them, tell them about the Ebola message, at least talk to them to encourage them to come to the hospital to come and seek medical attention”.

M: Is there any way to do to bring them to the hospital?

R: ”To bring the people”?

M: Yes, to bring the sick people to the hospital?

R:”Well, we were doing the house to house checking, we were going from house to house to check for the ones that are sick, then through the sensitization and communication given to them, it really pulls them out to come to the hospital”.

M: Ok, Like for Ebola messages, what you think is the best channel to send out Ebola Messages in this community?

R:”Well the best channel is through sensitization, this is the one best of the channel we do for them to be coming”.

M: Have you heard any good or bad thing about the ambulance service?

R:”Well the ambulance service, at least we are hearing good things, at least when we refer a case and call for them to come, they will come immediately to come and collect the patient, so this is a good thing we are hearing about them, they are really doing well”.

M: Have you not heared any bad thing?

R:”uhmmnh” (no), we have not heard any bad thing about them”.

M: What about the treatment centres/holding centres have you heard any good or bad things about them?

R:”No, they are really trying, they are really doing their level best, because no sooner we refer, like the one we have here, the CCC (Community care centre) as sooner we refer a case, they will communicate and call, then ambulance will come and collect the patient, we have not heard any bad thing”.

M:”What about the burial team?

R:”The burial team are also trying because the moment they call them, they will come and “ceebut” (prepare) the dead body and take it for burial”.

M:”You have not heard any bad thing about them?

R:”No, they are really doing well”.

M: What about the 117 phone line?

R:” Well the 117 at first when the Ebola just starts, people were giving out complains that when they call them, it will take a long time before they come, but now at least they themselves are trying, they are really doing well, because as sooner they call them, they will come”.

M: Ok, the health centres that have been there before and their staffs and the new treatment centres now, have you heard any good or bad thing about them?

R:”No, but when the centres are permanent and the Ebola treatment centres are temporary for now soon as the Ebola is done, we have not heard any bad, we are working together as one, when we have a case they will come to our aid”.

M: In this community do you have anybody that have cure from Ebola?

R:”Yes, yes, we have?

M: How are they reacting to those kinds of people?

R:”Well they are really coming closer to the people. They are not like shunning or disown them, push them far away, they are really encouraging the patient, meet and try to communicate with the patient, they are really close to the patient”.

M: Have you heard of any new treatment for Ebola?

R:”Well, they said, they have made a vaccines, but which we have not yet seen, but we heard they have make a vaccine from the bats itself”,

M:”What do you think of that medicine?

R:”We have not yet seen it, we have just heard of it, and we don’t know anything about it, so I don’t have any thought of it”.

M: What do you think will be the concerns of people about the treatment if it comes?

R:”Well, I don’t feel that some, maybe if you accept the treatment, some will not accept, they will feel that this medicine that have come, because they are thinking when the medicine comes they are going to give we the health workers first after which they will give to another people but it has it rules and regulations of what to do and what not to do, some people will think they have come with the medicine to kill us or damage a part from our body”.

M: Have you heard of any new way to prevent Ebola?

R:”No, the only way to prevent Ebola is to always wash your hands frequently”.

M:” But you have not heard of any new method to prevent Ebola?

R:”No, I have not heard of any new method yet”.

M:”What about the vaccines?

R:”the vaccines is one”.

M:”So you have heard?

R:”I have heard of it but not yet seen, so we don’t know much about it”.

M: So as a health worker, what do you think is the perspective of Ebola to people in this Community?

R: “Well at first the perspective was very low but now we thank God people are aware of this Ebola, they are looking towards preventive precautions, the Ebola they gave them, which at least we are getting now”.

M: What do you think you have to know about Ebola, to enable you answer more question or allay their fears about Ebola?

R:”To continue doing sensitization frequently, may be it will allay their fears”.

M: is there anything specific about Ebola you think people should know more?

R:” At least people have known everything about Ebola, I don’t think is there anything specific”.

M: so there is nothing specific about Ebola?

R:” there is nothing specific, because they are getting the message, is the matter of just continuing the sensitization lay emphasises on this sensitization”.

End of interview.
