# Supplementary material for: Development of a set of community-informed Ebola messages for Sierra Leone
Source: PLoS Negl Trop Dis. 2017 Aug 7;11(8):e0005742. doi: 10.1371/journal.pntd.0005742 (PMC5560759; doi:10.1371/journal.pntd.0005742)
Supplement: S1 Appendix — (ZIP) [file pntd.0005742.s001.zip › Ebola messages - FGD and interview transcripts/R2HC Ebola Fieldwork 1/R2HC Ebola F1 HW-Rural3.docx]

| CODE | **R2HC Ebola F1 HW-Rural3 (rural semi-structured interview with health staff and health volunteers)** |
| --- | --- |
| DATE | January 2015 |
| DURATION (minutes) | 45 |
| Collector nr | 3 |
| LANGUAGE INTERVIEW | Krio |

**PERSONAL DATA RESPONDENT**

| Age *(in whole years)* | 45 |
| --- | --- |
| Sex (Female = F, Male = M) - circle | female |
| Religion | Christian |
| How much time does it take you to walk from your house to the nearest PHU? (minutes) | 180 |
| Mother tongue: | Limba |
| Role in the health facility / health: | XXXXXXXXXXXX |
| Education level | none |
| Do you know anybody who had Ebola? | yes |
| If Yes, what is your relation to that person? | neighbour |

**TRANSCRIPT: (M= moderator, R= respondent)**

M: When did you first hear about Ebola?

R:”I heard of Ebola in the year that I past, since I did not go to school so I don’t know the day, date and month, but it is in that year that have past”.

M: Towards the ending or the starting when is begins?

R:”I will say the beginning, because the month has not gone far yet at that time”.

M: So how was Ebola described to you?

R:”They said Ebola is a bad sick, it is a sick that kills, you can’t see it with your naked eyes. Anybody that has this sick in your compound, you should not keep the person in your house, it will start with fever and the fever you get “belerun” (frequent stooling) and vomiting”.

M: When they told about the sickness, what were your first thought about this sickness?

R:”Well it’s a bad sickness, so I thought of it, it is a bad sickness, and this sickness don’t have medicine, and when you started seeing the signs the vomiting and fever, run to the hospital”.

M: How Ebola has affected your community?

R:”it’s affected us greatly, though the sick do not pressed us here, but it affected us, because one, when people are going to work, when they go to town, you will be thinking of the sick, you may not know the person you will meet, you must be thinking, when you go to (--Name of district headquarter town--) you will be thinking of this sick, then you will be thinking of your little children for this sick, because we have heard that, this sickness does not have medicine”.

M:”Have you seen a person with this sickness?

R:” Yes they came and collected one aged man here, down there, they said it is the sickness that has infected him, so they took him along, till now we have not seen him”.

M: So they took him along, when did they take him?

R:”Around the time when people are harvesting rice, it was October, I can just remember the month, but it was in October, like how I just said, when you have a book, if something happen which is to write, you will write like the date and the month”.

M:” What do you think has caused the spread of Ebola throughout Sierra Leone?

R:”Ebola spreads, when we “open”(turn on) our radios, as how we heard, that it came from one woman that went to Liberia or Guinea, they came and took her, she a native a “sowei”(a priest in a female traditional society) or within, so the woman went there, she was there when the sick person that she went to cure died, then she returned back to Sierra Leone, until we heard that is the woman that brought Ebola to Sierra Leone”.

M: When she came with it, why is it spreading now?

R:”Why is it spreading, because anybody with Ebola, as sooner the person sees the symptoms, the person must go to the hospital, you don’t have to keep the person at home, you don’t need to go near the person, you don’t need to drink in the same cup, so you have to send the person to the hospital at once, when you keep that person, the Ebola will spread in the compound, spread in the house and spread all over the town, because we don’t need to touch, always when you touch something that you don’t satisfy wash your hands with soap, don’t touch your companion”.

M: What is the best way to prevent Ebola from spreading?

R:” The best way, it is this touching, if only you sit down you don’t touch each other, you don’t wash the dead, you don’t go and greet a person that is sick if you come together and do this method, that will prevent this sickness and you will able to handle the sickness and it will get finished, anybody that is sick, take the person to the hospital”.

M: What do you think is the best way to treat somebody with Ebola?

R:”The best way to treat somebody with Ebola?

M: Yes how?

R:”Is to send the person to hospital, when you take the person to the hospital, the “well body worker” (medical person/staff) will treat the person with Ebola. When you get the Ebola don’t sit-down, as sooner you start seeing the symptoms, if go earlier you will succeed, but if don’t go earlier you will not succeed, because we are hearing when we lay down, though we did not go school “open” (turn on) your radio, some talks will be “catch-up”(hear), they said don’t allow ambulance to come for you, walk on your foot and go to hospital, if you walk from your house to the hospital, may be you will able to succeed, but if you allow ambulance to take you, is the same ambulance take you to the burial ground”.

M:”Before the person reaches at that stage, when ambulance will come and collect and have showing the symptoms of Ebola, do they have any treatment for the person?

R:”there is no way to the treat the person, the “herbalist” (traditional healer) will not cure Ebola, “sowei” (society priest) man will not cure Ebola, a pepper doctor (unqualified doctor) will not cure Ebola, until you call 117 to come and take then go with the person if you have seen it as Ebola, when the person have got the symptoms call 117 to come and take the sick person”.

M: Do you have any way you call Ebola in your Language?

R :”( with a doubtful mood) you don’t …have…, yes, in our own language?

M: Yes?

R:”Yes”.

M: How do you call it?

R:” E tondanaa keykuri arnn Ebola”.

M: “E tondanaa keykuri arnn Ebola” what do you mean by this?

R:”This means Ebola is a sickness that kills”.

M: E tondanaa keykuri arnn Ebola?

R:” tondanaa keykuri arnn keycany emu Ebola” (Ebola is a sickness that kills)

*(A child shouting)*

M: some people in this community believe Ebola don’t exist, why do they think so?

R:”they alone know why they don’t believe, this people that do not believe, they alone know, why they don’t believe, you see the “Well bodi” (medical) people have come and say there is a sickness coming on the way, you don’t have to sit and careless the sickness and they have known that this sickness is not the one that will just cure from native or all thing, so if they don’t believe, they are denying and people are still dying is their business”.

M: Can you tell me the type of people that don’t believe, either big or small people can you tell me?

R:”Well, us here, I have ever heard of it, because we are seeing they are taking our people and they go and die, we are also hearing about it from another village. We are hearing that so and so amount of people have died, without nothing but just from sickness and this sickness they said you can’t see it with your naked eyes, its “tumbo” (virus), once it enters you it circulates, you will walking you don’t know unless it attacks you, so you see, we here we believe”.

M: Will you tell me some examples of the Ebola messages you have seen and heard or know?

R:”No, I can’t remember their big talks”.

M: The talk that they talk at the hospital for Ebola or the advise they give?

R:”Ok, the advice, the talk about Ebola”?

M: Yes?

R:”These are the advices, they say sickness has come that do not have medicine, because they call us on workshops, and tell us that sickness has come which we don’t see with our naked eyes but it kills, lets us don’t sit down, this a “Family moudor” (a sickness that will kill a whole family), lets us don’t sit down on that sickness, if that is at your house, hold it there, if it is in your village you have to report, if you don’t do so it will kill the your whole family so let us take our time, when someone get sick, take the person to the hospital, if it is Ebola or not Ebola, you have to go, they will do the test on you, if it is Ebola maybe you will be cured and if it not Ebola they will also treat you for that sick. But this time we tell God thanks before when the sick came newly we were that people were dying, so if your body is warm go to the hospital, if your child vomit go to the hospital. First time people were going and do not come, there comes fear in people, so they were not going again ”.

M: With all these Ebola messages you have been told, which ones have worked in here?

R:” Here in our own village, since when Ebola came, they told us that there is a sick that do not they medicines, everybody should put water in bucket and place it in there veranda and put soap, any turn you turned, wash your hands, when you come from the toilet you wash your hands, you go for walk, when you come back, wash your hands, you have to avoid your neighbour’s house, sit down at you house, control you children, we have heard all those messages and we believe it and we are doing it”.

M: Which of the messages have not worked here?

R:”The messages we are hearing, the wash hands, then when someone is sick they will take the person to the hospital, even this delivery people were afraid before this time, I gave the zeal that let them go, and nothing is going to happen with you, we will not go they say it is Ebola, it will happen so, there will be symptoms”.

M:” With all the message you are telling me, which ones you like?

R:”I like them all, I like this wash hands, I like don’t touch, and I don’t hate any one and I like this one when some is sick go to the Hospital.”

M: Thank you, what is the Ebola message that will encourage people to bring/send their patient to the hospital?

R:”Like the other message now, like how I was telling just now, first before this time people were afraid, not so, but by now when we have started seeing, when they say someone is sick of Ebola, then the person is be taken and come back, so we are telling the people that when someone has laid down, when your body get warm and your head aches go to the hospital, don’t be afraid, go the hospital, if you sit down at your house, it might not be Ebola but another sickness, so when you die they will bury you as Ebola death so go to the hospital”.(*Voice of children at the background*)

M: In the event of Ebola infection, where do you think people will prefer to go, will they prefer to go to the hospital or another place?

R:”Like how I was just telling. First people were afraid to go, but by now people will go to the hospital, because everybody want good health. A person will not sit down and say let me go to an Herbalist (traditional healer), no because Ebola is not cured by herbalist. When you go to the hospital, they will go and check if you have Ebola or you don’t have Ebola”.

M: So they will stay at home again in the event of Ebola infection?

R:” Well for us here, the Ebola just hits us small, and it went back, it did not stay here”.

M:” Why it did not stay here?

R:”Because of the message they came and told us and we did what they said. But if we should decide not to work by this messages, and work by ours, the Sickness will spread all over this town. Don’t keep sick person, don’t wash dead bodies, and don’t go to burial house, don’t go to neighbouring house, they taught us this law before even the Ebola reach in (-- name of district head quarter town), but people first came at the village Barry in our village and tell us about this sickness, they even came with comedians, they ask us how did they catch the sick. So if we have heard the sickness has come we will take time”.

M: What do you think is the best channel to pass on Ebola message to the people?

R:”You have just talk to them”.

M:”How they should be talking to them?

R:”Like they call the people at the town Barry and talk to them, when you are call to workshops because not everybody is going there, so we when you come back, tell the people what you hear, so at that time you succeed in pulling this sick out of this country. But if you go to workshop on, what they told you about Ebola, when you come back and decide not to tell anybody, the people will not understand. What they told you where you are called to come and tell the people what you were told. If a person want to do it now, the person we say this woman went on a workshop when she return back, this is watch the told him about this bad sick, so am not going anyway, let me don’t go take a sickness and bring it to village and the sickness is a “family moundor” (a sickness that will kill a whole family)”.

M:” How you ever heard people talking of any good or bad about the ambulance service?

*(A voice of the goat)*

R:” Some people become afraid of the sound (siren) of the ambulance as it passes by, some says the speed when they are going for a sick person”.

M: (*let please talk louder*) yes were talking of the speed?

R:”Even if you were to be in the ambulance, you will be afraid because of the speed”.

M: Is there any good people talk about the ambulance?

R:”No, I just heard that they have come with a lot of ambulances to help fight this sickness, if they should have not done, Sierra Leone we are dead. If they say we don’t have to put the patient on the bike, the person will affected unless Ambulance. We tell the government thank you for the ambulance”.

M: What about the treatment centres?

R:”The treatment Centre, though we have not gone there, but those who have gone there when they come we will hear what say when they are interviewing them on the radio, they will ask them how was the place they took them, they will be advising their companion that anybody that is sick with Ebola, as the person sees the symptoms, don’t sit down call 117 let them come take you to the treatment centre. If the sick have not overcome you yet, when you go you will succeed and your life will be saved, but if you are late, you may not survive. They said when you go, they will treat you fine. The person that talked, person said the way hold them and “bayo bayo” (pamper), they eat, they wash them and dress them, the person said there is no problem. So I have heard them”.

(*Someone talking on the background*)

M: What about the burial team, do you hear any good or bad?

R: “I have not heard any bad talk about them”.

M: But what is the good talk you heard about them?

R:”Anyway let me don’t lie to you, one time when I was lying down, I heard it from (-- Name of another chiefdom in the same district --) chiefdom, they went there for burial, after the burial, they took off their dresses and drop them in the town, they gave that report that they are doing bad, they I said whenever they bury, they will remove the cloths they were putting on and place them in the street. When you bury an Ebola person will you drop the dresses that you are wearing? Exercise from bad language, when you step on “kaka”(skirt) where do you leave the shoes you step on that skirt with, are you going to leave it there, are you going to leave the shoe from everyone to come and see. Hand gloves and their dresses after burial they just put it on the line, I have one time heard of it”.

M:”What about now?

R:” Now, we are not hearing that, but because is the time when the sickness came up newly, that is why they were doing that, but for now no. First when they come and take your person, you will see that person again. But now if someone dies at the hospital, they will bury that person fine or someone dies like for instance in this village but not from Ebola, the person just dies that way, falls down and just die, they will call the team to come and they will come and take the blood, and bury the dead body, if it is the sickness, they will come and say it, that this man dies from this sickness. If it is not the sickness the leave you people, but if it is the sickness they will put you in a fence, they will quarantine you people for twenty-one days”.

M: What about the 117 phone line, have you heard any good or bad about them?

R:”No”.

M: What is No, now is it bad or good?

R:”It is not bad”

M:”Ok what is the good now?

R:”When a person is sick and the person does not have the number of the “Well bodi” (medical) people’ number, how would you call them, unless they call on that line for the people to come and collect you, not so? So it is not bad”.

M: So what about any existing treatment facilities/ staff that is now working there?

R:”Treatment centres, apart from the time they were going to Kenema (*district where initially all Ebola victims were sent to*)”?

M: Yes?

R:”I heard that is (--name of hospital in the same district--), is not where the treatment centre is”.

M: The one that you know?

R:”Yes, is there (--same name as above--) the treatment centre not so”.

M: What is the good or bad have you heard?

R:”Well is nothing bad about them, (--same name as above --) from the morning is a fine place”.

M: Apart from (--same name as above, hospital in same district--), is there any other place?

R:”Have not heard of any other place but I have hear of (--same name as above, hospital in same district--)”.

(*Motor bike passing*)

M” You said you know someone that have survive from Ebola, how are people in this Community treating that person?

R:”They are acting to the person well, they are playing with the person, and they do not drive the person away from them and they talk fine to the person and welcome the person”.

M:” When the person came at first, how was the person treated?

R:”They do take it as nothing, they just take the person as how we have been before. But we are hearing that when a person is sick of Ebola, we don’t need to be afraid of the person, you don’t laugh and provoke that person, so all of these we have heard because the person has been cured, we have to hold that person with our two hands. You that is not been sick with Ebola, you should be afraid, is you that should be having that fear, because the person has been cured from Ebola, so if it is going to be any Ebola in your village it will not catch the person again”.

M:” have you heard of any treatment for Ebola that will be coming soon in the country?

R:”No I have not heard of it, that side I have not heard of it, sometime I did not open radio, but they will call us on workshop, and tell us anything that has come that is new. But even when the Ebola came, they came and told us about, tell us the animals that we should not eat and we have followed that method”.

M: Have you heard of any new way to prevent Ebola?

R:”You mean the person have not got it”?

M: I have you heard of any new way to prevent Ebola?

R:”I have not heard of it”. (*Children playing*)

M: I have you heard of any vaccines for Ebola that may be coming soon into the country?

R:”Yes, I have heard of it”.

M: What did you hear?

(*Children talking*)

R:”They said they are trying, the white people are trying to bring the “marklate” (vaccines) for Ebola, but it has not yet reached here”.

M: What do you think of the vaccines when you heard of it?

R:” Well I felt happy, you know what? For me to happy for to hear they are coming with “marklate” (vaccines) for Ebola, because first they said there is no medicine for it. But if it is here now, when the sickness comes again it will not catch anybody, we will attack it quickly”.

M: So as a traditional birth attendant, what do think you may want to know about Ebola, which you don’t know, so that you may able to respond to question ask by people?

R:”What I want to know about Ebola?

M: Yes what do you need to know really when people may ask you as a traditional birth attendant so you may able to answer more?

R :”( *doubtfully)* what I may want to know”?

M: Yes what do you need to really know when people may ask you ask a tradition birth attendant so you may able to answer more, either young man and young woman may ask?

(*Voice of children*)

R :”( *still doubtfully*), when did I hear about the sick or how the sick came or what they told me about the sick”.

M: Yes that is what I want to know?

R:”That, we were just hearing sickness as, sickness has come, you don’t where this sickness came from. So I want to how this sickness came, so when they ask me that question I will answer not so?

M: That you may like to you know?

R:”Yes”.

M: Is there any other thing you may like to know more about this sick?

R:”Uhhmm, I want know, as this sick as come, if this sick is not going to done again or is going to done, if this sickness will be coming every year ”.

M: uhm, those are the thing you want to know?

R:”Yes”.

M: Is there anything specific about Ebola you may like to know better, so you can explain to you?

R :”( *long silence) (* ) I said I want to know when this will finish, or it is not going to finish, I want to know not so. Ok”.

M:Is that the only thing you may like to know, there is no other thing?

R:”uhhhumm”.

M: ok thank you very much, this is the end of the interview.
