# Supplementary material for: Development of a set of community-informed Ebola messages for Sierra Leone
Source: PLoS Negl Trop Dis. 2017 Aug 7;11(8):e0005742. doi: 10.1371/journal.pntd.0005742 (PMC5560759; doi:10.1371/journal.pntd.0005742)
Supplement: S1 Appendix — (ZIP) [file pntd.0005742.s001.zip › Ebola messages - FGD and interview transcripts/R2HC Ebola Fieldwork 1/R2HC Ebola F1 HW-Rural4 V2 CORR.docx]

| CODE | **R2HC Ebola F1 HW-Rural4 V2 CORR(rural semi-structured interview with health staff and health volunteers)**  **V2 – 11^th^ March 2015 – corrected personal data respondent** |
| --- | --- |
| DATE | February 2015 |
| DURATION (minutes) | 31 |
| Collector nr | 6 |
| LANGUAGE INTERVIEW | Krio |

**PERSONAL DATA RESPONDENT**

| Age *(in whole years)* | 39 |
| --- | --- |
| Sex (Female = F, Male = M) - circle | Female |
| Religion | Muslim |
| How much time does it take you to walk from your house to the nearest PHU? (minutes) | 35 |
| Mother tongue: | Madingo |
| Role in the health facility / health: | XXXXXXXXX |
| Education level (circle) | Secondary |
| Do you know anybody who had Ebola? | Yes |
| If Yes, what is your relation to that person? | Family |

**TRANSCRIPT: (M= Moderator, F=Facilitator)**

**M:** When did you hear about Ebola for the first time?

R: “August 15”.

M: Which year?

R: “2014”.

M: That was last year?

R: “Yes”.

M: How was it described to you?

R: “This sickness we were getting case, because I was first at the affected community, but they were complaining they have ulcer, but since that they have been telling us that the signs and symptoms of Ebola, when a patient comes and start bleeding from the nose, get sore throat, fever, “warm bodi”(high body temperature/fever) and frequent stool, that is the signs and symptoms of Ebola, they said we should not touch that patient, let isolate the patient and then send the person to the holding centre. We were seeing those cases, but they were complaining that they have ulcer, some were saying its pressure (hypertension), but in two days’ time we will see the signs and symptoms of Ebola from them. So straight away we will isolate them, to avoid us from getting infected. I was there in August and September, then I went to the treatment centre to train how to wear the personal protective equipment (PPE). I was in that training for two days, from there, they said they will call on me when they put things in place, they put things in place in November, November 13^th^, they called on me that the training I went last how to wear the PPE, I should report, form there I went to (--a hospital in the sam district, with a treatment centre--), were they allocated me to my shifts and I came back at home to go for work the next day, the first patients which I came across, the patient was having difficulty breathing, the patient told me that, she is suffering from cold, the she was once a TB(Tuberculosis) patient, now your result is here from the holding has Ebola positive, I will not say I have to give you TB treatment, until you are cure before we give you TB treatment, as long as you are vomiting I will treat you, then I give the patient ringers lactate (an IV fluid), three litres, I was inside my PPE for three hours, because the training do not call for doing three hours, but I went inside with my buddy(teammate), so I decided, because we were not plenty, from then I give the patient dextrose fifty percent (another IV fluid), that I gave the person zinc oxide for the frequent stool, because at that time the patient has dehydrated a lot, I came and asked if they don’t have vitamin k, that is to stop the bleeding, they said vitamin k was in the drugs, so I said let somebody go inside and administer the vitamin, to stop the bleeding, but the bleeding could not stop, because it has gone to stage three and the patient will end up to pass off, so every day we went inside to give treatment and IV(intra venous fluids) when I am in the night shifts, I will make sure I go inside by ten o’clock, I will take hours and get out, but anytime I went, not normal “sirange” and full litres. If I see that you are better, I will stop give you fluid but oral, oral rehydration salt(ORS), Panadol and routine (medication, usually oral vitamins/minerals), if the person still complain of frequent stooling, I will give you the zinc oxide, I will start a dose of four, Panadol, dextrose (an iv fluid) and ciprofloxazine (antibiotic) and flagyl (antibiotic). So we have been treating those patients, up to date we tell God thanks only few that will not able to survive, because they not report earlier at the holding say they are infected, they will give it, until it goes above our level, those type of cases will ended passing off, but the one that report to the hospital, indeed we will try our own way, because when we enter inside we the nurses, we are the doctors, and everything, there was a time we even ask that now that we are entering inside, we don’t the right took their vitals (vital signs) to check and know, if you are checking the pulse and it is going rapid, you will know how to administer any drip, if you check back the pulse, but we don’t have a seconds hand watch to use. We will not go with phone, we will not go with nothing inside there. From then our Matron (head of all nurses in the hospital) told us she will have a clock, but this clock is there now and the cases have reduced and there is nt patients again in the hospital like November and December we getting like thirty something and forty something, we you go inside, we will not come out, you will be there for three hours, so has we give the ringers lactate (IV fluid) and cipro (ciprofloxazine, an antibiotic) may be typhoid has been in the body again, we will just say to treat you for that frequent stool and vomiting, we just have to back it up with cipro and dextrose (an iv fluid), half litres of each, any two hours nurse much enter inside, we will not say let just leave them that way, we not see when we are doing night, but now let say when the others are doing late duty, then will come out, we will give them some hours before we enter inside. But now we thank God it is getting less, we only get one case and even yesterday we discharged five of the survivors, but thank God now”. (*A very noisy environment*)

M: As a health worker, in which way you think Ebola has affected your community?

R: “The community am living now, I don’t think if Ebola has affected them too much, because they are taking to the advice we are giving them, because when its morning, I will sensitize my neighbours, to know what they will do now, up till now we only got one case, which is my sister and she was a nurse, but she did not affect us here, she infected the regional hospital which is the maternity, when she went to deliver her child there, the woman was positive, from their she got, but she comes she protected us all by telling us and we isolated her and until she was taken to Kailahun (another district) and come back survived and she is with us”.

M: What do you think has spread Ebola all over Sierra Leone?

R: “Is because when this thing started, people did not believe that Ebola exist, they just took as, Ebola is a lie message, Ebola does not exist, until September when Ebola killed people seriously, because when they take you to the hospital, you start feeling your head you don’t report earlier,(*someone shouting*), when you are feeling your body, you will not report earlier until a person is infected. If you have started getting the symptoms, why can’t you report earlier to the hospital, but they are not reporting earlier in to the hospital, they themselves were denying that Ebola is a lie, Ebola does not exist, Ebola started killing in August and September, that was the time people started believing that Ebola exist and it is a sickness that kills”.

*(A shape voice at the background)*

M: What do you think is the better way to prevent Ebola from spreading?

R :( *long silence)*

M: Did you understand?

R: “Yes I got you, but trying to about the answers”.

M: Ok

R: “The only way to stop this thing, that body contact when we gather in group, we need to seized it until end of December, 2015”.

M: Why December, 2015?

R: “Because people are not still taken to what we tell them, if still we get few cases up to December, at least every one should have got it that , if we continuing doing so, this sickness will never end. The public gathering, the body to body, we have to avoid all that, the hand washing should be everyday our priority, should be our priority every day, if you are a nurse and you go and do any procedure, when you return, you see that you wash your hands properly, when you do anything, you even touch, maybe that person is infected and you don’t wash your hands, it means you have catches the sick, because what they telling us about this Ebola, if we are really take it, I think that this Ebola should have finished at this time and we have got freedom now, but people are still not believing, we have sensitized them, but since now we have contact tracers that will move from house to house to sensitize the people and I think, that will look better.”

M: Like how you are a community health worker, what do you think is the best way to treat somebody with Ebola?

R: “The only way I think, as you start feeling your body, you are to go to holding (holding centre), they will do your test, immediately the result comes out positive, the will take you to the treatment centre straight away they start treating you, and you will make it”.

M: So is early treatment?

R: “Yes early treatment”.

M: Are there any local name or terms that you people use to describe Ebola in this community?

R: “No”.

M: You don’t have any other term for it?

R: “No, we don’t have”.

M: In the event of Ebola infection, people will prefer to stay at home, you as a community health worker, why do you think, people this way?

R: “Because some people are taking as, when they go to the hospital, they are afraid of the hospital too much, but I think when you have things that like that, if a person is sick of Ebola in your house, you have suspected that this, ok the sign did not show up quickly, the bleeding is the …. that it has reach when there is no way, when you sees that even if she is my mother that have lie now sick in this house, she is getting frequent fever, complaining head ache, straight away I will isolate her, lock her in the room and call 117, I will make sure when am transferring to the next room if I don’t have gloves, I will use the plastic on my hands and transfer her to the next room, the next room am transferring her, I will call 117 to come and pick her, for then if they check she is negative then ok, but if she is positive, she will be there and start taking treatment, because I have taken her there at the earlier stage, than I kept her when she starts bleeding then call 117, all of us will get infected, because that touch. But even if my mother that complains head ache, I will isolate her in the other room, before I isolate her, I will not use my use my naked hands when transferring to the other room, if am not a nurse, I don’t have gloves, I will use plastic in my hands to touch my mother and transfer, then I will tell my children that you should not go near her, until they take her to the hospital, when the result comes, if she is negative all of us as save, but if it is positive then she will be treated and come back at home*”.(People arguing at the background)*

M: Some people do not believe that Ebola exist, do you know those people in your community?

R: “They will not say that before my face, they will not talk that before my face, but I know everybody, even the layman in the community knows that Ebola exists, no person will deny this time around for Ebola”.

M: When Ebola came they were giving out plenty of messages can you tell me some of those message you have heard or seen?

R: “Well the messages they are giving, they said when you go to the hospital, and they will inject you to death that was the message I

was hearing, they said we are the ones that are killing the people saying that there is Ebola, wrong information they were giving the people, don’t shake hands, no body contact, they said we are just formatting this messages”.

M: Which one do you think is the best Ebola message that will stop the sick?

R: “The only way this sick will finish, don’t keep sick person at home, and when you have a sick person grumbling the head, do not even treat the person at home if you are a nurse, if you are not a nurse, don’t ever buy go and buy even Panadol in the street and give the person to the headache to relief, you will be doing more harm, take the person to the hospital for finding, when the person complaining the head take her to pharmacy than when you will go and buy Panadol in the street and give the person”.

M: As a community health worker what would you think is the good message to encourage people to bring patient to the hospital?

R: “Well, Unless we go house to house and talk to them, in community, that I don’t want you to even keep dead body at home and wash , when someone got a sudden death you people don’t touch the person, see that you have taken person go, but if you can’t go with the person, call 117, they will come and pick the person and even take the blood sample, test it and when the result come out, if it is not Ebola they give you burial permit and you bury, but am advising everybody in the community, do not encourage to keep sick person, do not to touch the dead,if we do this, that will help us finish the sick”.

M: In the event of Ebola infection do you think people would like to go first to a traditional healer, or the existing facility or the new built Ebola centre?

R: “You know, some of our people are difficult to talk to, some people when they have sore throat, they will say, someone has hands in it, so let go to the herbalist, but if the herbalist man know this time around, he will tell you to go to the hospital, that you have protected yourself and the next person, when you go to him say you are feeling your throat I can’t swallow, then he went head to “lukin gron” (conjuring) and says am able and know how to produce medicine. If you have toothache then the herbalist says I know how remove the teeth without you going to the hospital that will never happen, you don’t know the status then you went ahead to remove the teeth, then you come, you don’t know the status of the herbalist man but we have stop all of this things, Ebola will finish”.

M: From all the Ebola message we have been getting, which is the best, any which way do we use to reach the people?

R:”Let the people got the precaution for the sick”?

M: Yes?

R:”this hand washing, anything so far you touched, wash your hands with a water that is having chlorine, the chlorine you will wash your hands with, if you are nurse any procedure you are undertaken make sure the gloves that you have used to touched, make sure you removed and wash your hands and use another gloves, that you have protected yourself and you have protected the next man, you don’t have to used one glove on two patients”.

M: Which channel you think we use for the people to get this message you have just said like through radio, text message which one you think is the best?

R:”Well the best way, saying it on radio text messages, not everybody that have or listen radio, the only way is the house to house sensitization that will take the message through”. (*Motor bike passing*)

M: Which good or bad thing you have heard about the ambulance service?

R:”The ambulance team, they don’t have any bad thing they did, as you call them immediately they will come, the only thing, you will not call them at night then they come but during any hours of the day you will call them and they will come. The only thing were government made mistake is the risk allowance that they do not paid to time, they came first and say verify, this verification they said you should give, you that is on payroll, should give your account number, your telephone number, then they snap you (photograph), you fill form and go. Then the second time, if you are a nurse, but don’t have pin code number, you will give your phone if you want your money be paid through Airtel money if you want, they started that but just few people was having Airtel money service. This started from December, the second week in December, only few receive the money and others did not. This is the problem, but the workers are ready to finish this Ebola. The agreement you made with a person, now come and work I am giving you this amount because not really the money we went for, if you are a medical person, this is our own war, we are not going to the market and sell pepper the only thing unless we work to table the people, we are all Sierra Leonean but if things like this rise, then you say you are not working because of the money, we are not looking at the money, we are at risk, we sacrifice our lives, at least to compensate us, the one they said they are given us, let them give, some of have families, that is urging us more to work”.

M: What about the holding and treatment centres?

R: “It is just the same, the holding and treatment centres; they are not paying people on time”.

M: Do you have anything good or bad for the burial team?

R: “For the treatment and burial team, really we turn up; the burial team don’t have any problem, the treatment centre, staffs, and the porters they really worked. If it was this they pickup in early August or September the cases death should not be plenty, but is only late November they built treatment centres, but we thank God even the late time of building we turn up and the ones that God destine to survive, they survive, only few that die on our statistics”.

M: Any good or bad about the 117 phone you have heard?

R: “There is no bad thing to tell about them, they are really committed to the job, any hour you call them, fuel have never shorten with them, they paid they don’t pay, they will not stop work for that, fuel have never shorten, to say we don’t have fuel, that is why you don’t come, no, and the only problem is the salary payment”.

M: Do you know any Ebola survivor in your community that went to treatment centre and came back?

R: “Yes”

M: How do people react to them, do they encourage them as they come?

R: “When they come”.

M: Or they stigmatised them?

R: “We encourage them, we were glad and play with them, and nobody did not give any comment”.

M: Have you heard of any new treatments for Ebola that may become available soon?

R: “No”

M: Have you heard of any vaccines for Ebola that may be coming soon?

R: “I heard of the vaccines from contact tracers, but it has not reached our own level”.

M: As a health worker, how do you think the knowledge of people of the disease Ebola has improved?

R: “The way they listen to radio, we will also talk to them, sensitize them that this and this is procedure, and through by this, they are aware and they all want this thing to done, because all over the country have become tightened for everybody, so this time around. They are doing what to do to end this thing, they are not under rating it again”.

M: As a community health worker, what you think you have to know to respond to those questions people ask about the Ebola?

R: “When I come they will ask me how many cases we have, how patients are doing, where the person get infected, what is the cause and I will enlighten them about the cause”.

M: is there anything specific about Ebola you may like know and you may think people need to understand better to have solved this problem?

R: “The only thing still let the people get the house to house sensitization, the vaccines they are talking about, let them start using it”.

*(Phone ringing)*

M: Just like I told you, what do you what to know, so that you can explain to the people better I heard your talking of house to house, it there any new?

R: “Yes, you have to say it over the radio, put it on the media, sending text messages to the people’s phone, at least the person that read and write will understand”.

M: Ok thank you very much.
