# Supplementary material for: Development of a set of community-informed Ebola messages for Sierra Leone
Source: PLoS Negl Trop Dis. 2017 Aug 7;11(8):e0005742. doi: 10.1371/journal.pntd.0005742 (PMC5560759; doi:10.1371/journal.pntd.0005742)
Supplement: S1 Appendix — (ZIP) [file pntd.0005742.s001.zip › Ebola messages - FGD and interview transcripts/R2HC Ebola Fieldwork 1/R2HC Ebola F1 HW-Rural5 V2 CORR.docx]

| CODE | **R2HC Ebola F1 HW-Rural5 V2 CORR(rural semi-structured interview with health staff and health volunteers)**  **V2 – 11^th^ March 2015 – correction personal data respondent** |
| --- | --- |
| DATE | February 2015 |
| DURATION (minutes) | 27 |
| Collector nr | 5 |
| LANGUAGE INTERVIEW | Krio |

**PERSONAL DATA RESPONDENT**

| Age *(in whole years)* | 54 |
| --- | --- |
| Sex (Female = F, Male = M) – circle | Male |
| Religion | Muslim |
| How much time does it take you to walk from your house to the nearest PHU? (minutes) | 60 |
| Mother tongue: | Fullah |
| Role in the health facility / health: | XXXXXXXX |
| Education level (circle) | Secondary |
| Do you know anybody who had Ebola? | Yes |
| If Yes, what is your relation to that person? | Workmate |

**TRANSCRIPT: (M= Moderator, R= Respondent)**

M: When did you hear about Ebola for the first time? How was it described to you, and what were your first thoughts about it?

R:”I heard of Ebola on BBC radio, the time it was in Uganda long time back, but you mean inside Sierra Leone”?

M: Well in Sierra Leone, it was around April, the time Nationals were coming around to sensitize when they heard of it in Guinea, at that time our bosses were coming, and we were even part of that team”.

*(People laughing at the background)*

M: How was it described to you?

R:”They said the person will get frequent stool, vomiting and also hosing blood from the openings”.

M:”So when you were that, what was your thought about it?

R:” what I think of that, well, when it was a strange sickness and they told us how a person get it, so therefore I was balancing this ways not to get it, because at that time, they were talking us about stories, since we were going to borders to sensitize people about it, telling them that, they should be very vigilant, this side at that, they have got it there from Guinea border with Dambala axis”.

M: How has Ebola affected your community?

R:”uhumm, it is great, we have lost loved ones, business have dropped, it is really “boku” (plenty), and Ebola have affected all facets of society”.

M: Have you personally seen or know someone who have had Ebola/

R:”Yes I have seen”.

M: So why do you think Ebola has spread throughout Sierra Leone?

R:”Because the frontline ministries were not having the resources to fight the disease and government did not take the appropriate action at the appropriate time that led to the spread”.

M: What do you think is the best way to stop Ebola form spreading?

R:” The best way to stop Ebola from spreading, the area where it begins, especially the rural settings, if they are able to condom that area, they try to isolate and finish it there, it will not spread, because Ebola is a sickness of contact, but if we able to limit the contact, within the Epicentre, it will not spread”.

M: What do you think is the best way to treat somebody with Ebola?

R:” Well the best way to treat somebody with Ebola is to replace the lost fluid, because Ebola makes someone to lose body fluids, so at time to replace the body fluids, when weakness has set in”.

M:”So in your Fullah language, do you have any local term you call Ebola?

R:”No, because, this is a new sickness, that is not common with us, this is the first time it has reached here; there is no name for it”.

M: Some people still don’t believe Ebola exists. Do you know or have those type of people in your community and why they thinks so?

R:”I don’t need to know the person, because as how I told you just now, I am one of the frontline staffs, because my role is the coordinate the burial teams, convey this and that, so nobody will talk it in front of me, but still I have the believe there are those type of people that still don’t believe but me and that type of person has not sat one on one to discuss that, because I know how the thing is burning me”.

M: Yes I understand, but you don’t have any idea as to why people think so?

R:”Well the idea of people having “runbele”(a running stomach or frequent stool), vomiting, people having warm body(high body temperature) we have being having sickness like that, because those sick are signs of malaria and other sickness we have being having, so to just come and say it is signs of Ebola, people will start thinking”.

M: Can you please give me some examples of Ebola messages that you have heard, seen, or read, either on the radio, or over television or on paper, text message you have received?

R:”Yes, to say earlier treatment saves live, safe and dignified burial protect you and your entire family, avoid eating bush meat, avoid body contact, hand washing, all those half stuffs”.

M: what do you think is the best Ebola message of all what you have stated and what you have come across to date?

R:”The Best one, Avoid Body Contact, to me is the best one”.

M: Why do you think is the best message?

R:”Ebola is transmitted through body contact with an infected person, so if I don’t touch you that is having Ebola, I don’t get it, Ebola do not fly, you either go and meet it or someone brings it to you, so if you don’t touch me and you touch you, I don’t think that will make, because even the first index case in Sierra Leone, they said it was a herbalist that went to cure a person and later tote (carried) and bring it to Sierra Leone”.

M: Which of the Ebola messages have not worked so well?

R:”Like the one that says Ebola is transmitted by eating bush meat, because our first index case here does not eat bush meat, the person contacted the actual person that was having Ebola and then come with sick”.

M: So you said avoid eating bush meat did not work well to the people?

R:”uhumm” (No) to me No”.

M: So what do you think would be the best message to encourage people to bring their patients treatment centre if you are sent by the Government?

R:”The message I will give to them, say I went into your community to pass on Ebola messages, I will tell them that anybody that is sick, like Ebola as we just spoken, your body will become warm, you will have a “runbelle”(running stomach or frequent stooling) and those signs have been with us, the sickness that portray those signs have been with us (a phone ringing), so the best message I will tell you, anybody that is sick, let the person go to the hospital, at the hospital they will test either, it is Ebola or not Ebola”.

M: In the event of Ebola infection do you think that people will prefer go first to a traditional healer, to existing health facilities or newly established Ebola health facilities?

R:” like the community person?

M: Yes?

R:”The person has known that he/she has Ebola”?

M: Yes?

R:”The person will straight away go to the Ebola treatment centres”.

M: Ok?

R:”Yap”

M: Some people stay at home, when they think they may have Ebola, why do you think this way, and which message you will give them to encourage them to go to the treatment centre?

R:”I don’t know why they will decide to sit at home, when they may know they have Ebola, but the message I am going to give them is that if you go quick to the hospital you will get “well bodi” (good health), they will treat you and cure you, because we have seen a lot of survivors, but the reason for staying home, I am not able to answer those questions”.

M: What will be the best way to get new Ebola messages, because we have been hearing from Radio, text messages, newspapers and posters, what do you think is the best way?

R:”The best way is to use the community people to pass it on to their localities”.

M: What are the good things and the bad things, you heard people talking about the ambulance service?

R:”Well the good thing about the Ebola ambulance service, as now we have “boku” (plenty) of them, now we don’t have delay in conveying patients either to the holding or treatment centre”.

M: Ok?

R:”The bad thing, the drivers are indisciplined to some level, they over speed, unnecessary putting on of siren those kind of things, the attitude of the drivers”.

M: What are the good things and bad things you have heard of the treatment centres, the holding centres and the community care centres?

R:”Well in the earlier days of Ebola, everyone was fearing Ebola, all man, even the ones at the treatment or holding centres, they are there because they don’t have no place to run, but to say they have that mind to go near the Ebola patient, at that time they were afraid, but now they have much of training on IPCs (infection prevention control) and all other trainings, they are trying and they are doing well”.

M: You said IPC what do you mean?

R:”It is infection prevention control, not to get infected”.

M: What are the good things and bad things you have heard of the burial team?

R:”Well in the earlier days the bad things was, they were not trained, is like they will just go and take, so and so in the earlier, but as time progresses they will just good and talk to the people in a good manner and explain to them why it is necessary for them to bury the dead”.

M: What about the 117 Ebola line, is there any good or bad things you have heard people talking?

R:”Because the 117 is overloaded, because if they do it for fourteen administrative Districts all is reporting at a single place, it will be overloaded, it will be having delay in responds, because assuming somebody is behind (--Name of remote location in the interview district--) calling 117 in Freetown and which (--Name of interview district--) District is to take action, it will go and bounce there first, the people there do not even know the terrain, some the caller over there, the way he/she call the village were the incident happened, then the way they receive the message and send it back here, it is a little bit of delay”.

M: So are the good and bad in any aspects of the existing health facilities/staff that is now working on Ebola care and treatment?

R:”Well I will most of the staff that were working in those existing health centres are working back in the Ebola holding and treatment centre, because these are special institutions that they established, not our own staff did the recruitment. So as I told you earlier, the early days people were afraid, and the thing you are afraid of, you will do the bad thing. Like if you talk with the early survivors, they will tell you, they are just putting the medicines and food, if you are able to eat, if you are able to eat fine”.

M: So how are people treating an Ebola survivor in your community, have you ever seen any Survivor?

R:”Yes *(…….not clear*)”.

M:We here, we don’t have problem, unless now when few survivors are misbehaving fighting loved ones, that lead to people talking about those bad behaviours on the radio the feedback of those programme aired”.

M: What is your message to people that are not acting well to those survivors?

R:”Well me here, I have not got anything on localities that didn’t received the survivors well”.

M: So what the other District, which messages will you give them, for those that react bad to the Ebola survivors?

R:” I will tell them that survivor is your own person and, they are heroes, they do not require any specific treatment, they need symptomatic treatment right, so in the nut shell, the person is the one that is recovering from Ebola, the person is a hero, the person have gone through the fight, now the person came out victorious, so those kind of people we need to encourage”.

M: Have you heard of any new Ebola treatment may become available soon?

R:”Have not heard of any treatment, it is a vaccines not a treatment”.

M: So have you heard of any new ways to prevent Ebola?

R:”Well I heard of the new way, which is vaccines, that is preventive”.

M: From your own perspective, how do you describe the knowledge of Ebola of people in your community?

R:”uhmmmmmm, now the people take the thing very serious, but our culture and passion of touching the sick, this that, is our greatest problem, but nobody will my will not say they do get the message, what you need to do about Ebola”.

M: So what do you think you need to know, to enable you to respond to some people’s concerns and misconceptions, what you need to know to address them?

R:”What do I need to know, uhhmmm that one”.

M: What do you feel you need to know?

R:”I need to know that is Ebola is real, it is a sick of itself, having the similar signs like malaria, and diarrhoea “.

M: Is there anything specific about Ebola that you think need to know and understand better?

R:”specific”?

M: Yes is there anything specific about Ebola that you think need to know and understand better?

R:”Yes, the specific thing I wants to know, Ebola is not flying and land into your community, Ebola either you bring it or somebody bring it to you, is not a sick which is air boned, or walking with rain, it is a “tumbo” (virus) that is brought by somebody or you go and take it from somebody in another area”.

M: So what will be the best way to explain what you just talk to the people?

R:”The best way I have just spoken, if you know that Ebola is that particular area don’t go there or don’t encourage that person to come to that you are”.

M: Thank you very much sir.

R:”Ok”.
