# Supplementary material for: Development of a set of community-informed Ebola messages for Sierra Leone
Source: PLoS Negl Trop Dis. 2017 Aug 7;11(8):e0005742. doi: 10.1371/journal.pntd.0005742 (PMC5560759; doi:10.1371/journal.pntd.0005742)
Supplement: S1 Appendix — (ZIP) [file pntd.0005742.s001.zip › Ebola messages - FGD and interview transcripts/R2HC Ebola Fieldwork 1/R2HC Ebola F1 HW-Urban1 V2 ADD PROBE.docx]

| CODE | **R2HC Ebola F1 HW-Urban1 (urban semi-structured interview with health worker/volunteer)**  **V2 – 11^th^ March 2015 – ADD PROBE** |
| --- | --- |
| DATE | February 2015 |
| DURATION (minutes) | 27 |
| Collector nr | 5 |
| LANGUAGE INTERVIEW | Krio |

**PERSONAL DATA RESPONDENT**

| Age *(in whole years)* | 30 |
| --- | --- |
| Sex (Female = F, Male = M) – circle | F |
| Religion | Christian |
| How much time does it take you to walk from your house to the nearest PHU? (minutes) | 10 |
| Mother tongue: | Krio |
| Role in the health facility / health: | XXXXXXXX |
| Education level (circle) | Secondary |
| Do you know anybody who had Ebola? | Yes |
| If Yes, what is your relation to that person? | Community Member / Neighbour |

**TRANSCRIPT: (M= Moderator, R=Respondent)**

M: When did you first hear about Ebola? (*Motor bike passing)*

R: “I heard of Ebola in May 2014, I heard it from Guinea until it came to Sierra Leone”.

M: How was the disease described to you?

R: “As it was described. They said when you are infected with, you bleed from your nose, mouth, Ear, and even you get bloody stooling”.

M: what were your first thoughts about it?

R: “I thought badly about it, because when I watch the pictures, and I saw how it is, I felt so bad, so I just imagined if someone is infected, how it will look like, so I had a bad imagination”.

M: In which ways has Ebola affected your community?

R: “it has affected us badly, it really put us in a state of no rest, like recently this community was a hot spot, any one or two days, they will come to collect an Ebola patient, so we were afraid, we restricted our movement, we were just at our own places”.

M: “Have you personally seen or know somebody with Ebola?

R: “I have heard but not seen, at times I hear people saying this particular person have got Ebola by (- - name of location close to interview community - -). But I did not go there”.

M: Why do you think Ebola has spread throughout Sierra Leone?

R: “Because people were stubborn, we were serious about it, when they tell us not go this way, we will go. When they tell us to report or sick and dead persons, some people will not. So we did not take this Ebola serious that leads to the spread of Ebola”.

M: What do you think is the best way to prevent Ebola from spreading?

R: “The best way I will prevent Ebola from spreading, one, when someone is sick, getting the signs and symptoms of Ebola in my house, I will tell the person to go to the hospital, and I will not touch that dead corpse, I will call 117 for the burial team to come, I will avoid places where there is Ebola death, I will also avoid touching a sick person, so this will prevent the spread of Ebola”.

M: What do you think is the best way to treat somebody with Ebola?

R: “Well the best way to treat Ebola, I will take the person to the treatment centre or call 117 to come and take the person, so that the person should be isolated and should be treated at the treatment centre”.

M: Are there any local names or terms that people describe Ebola in their local language?

R: “No, there is no other name, it is called Ebola”.

M: some people do not believe Ebola exist. Do you know people in your community and why they think this is?

R: “When it start, some people were saying, there is no Ebola, I will tell say stop, Ebola exists and it is real, unless the Ebola started killing hundreds of people, that is the time they came to believe, even in my compound we were having two to three people that did not believe it exists and it is real. I was arguing bitterly with them, telling them that we at the hospital know what we are facing right now”.

M: Please can you give some examples of Ebola Messages that you heard, seen or read?

R: “Yes, the Ebola message I heard, seen or read, they are going house to house, advising people”.

M: So what are these advises or some of the messages they give?

R: “They said when you are sick, go to the hospital, they said when someone die, don’t touch the dead corpse, call 117 for the burial team to come and collect, don’t visit sick persons, don’t visit a house that someone had die, so these are the messages they were they were telling us”.

M: All these messages you have heard, which one is the best one that have worked well?

R: “The one that has worked best, is the one, when someone has died the burial team will come and collect the corpse and taken to the cemetery, I don’t seen that yet, but it has worked”.

M: Are there any Ebola message that have not worked well?

R: “The one that have not worked well is the one about keeping and treating sick people in the house. People are still doing it, so it does not work well”.

M: What do you think would be the good message to encourage people to bring patients to treatment centre, holding centre?

R: “What will be my message, I will first tell them the importance and deadliness of Ebola, and I will tell them that when someone is sick, don’t be afraid, take the person to the hospital. I will allay their fears towards all the ill thoughts they are having that when you go to hospital they will not treat you good. So I will tell them, people have survived of Ebola when they go to hospital, so go to the hospital when you are sick, they will treat you fine. I will encourage and talk to them to always come to the hospital”.(*noisy background)(motor bike passing)*

M: In the event of Ebola infection, do you think that people would prefer to go first to traditional healers, to the existing health facilities/staff (including community health workers) or the newly established Ebola health facilities, the treatment centre, care centres and the holding centre. Why do you think this is?

R: “Well they will first go to the community hospital”.

M: why do you think this is?

R: “Because it is nearest, they go there first, then they make referral”.

M: Some people stay at home when they think they have Ebola, why do you think this is? What would be your encouraging message to bring them to the treatment centre?

R: “Before now, they were afraid to go to the hospital because of bad news they were having, so I will encourage them to come to the hospital, I will tell them that I have seen someone that was sick, the person went to the hospital and they cured the person, so don’t be afraid to come the hospital because a lot of people were sick, they went to the hospital and they were cured. Come to the hospital earlier, the earlier you come the better chance you have to survive. Some people have not survive because they did not go earlier to the hospital. I will encourage them to come to the hospital, when they get sick”.

M: What do you think would be the best channel to get your new messages to people that are in the remote places?

R: “The best way is through publicity”.

M: How?

R: “Well, we will organise people to be reaching the people up the hills; remote villages, sensitize them about Ebola. Because not everybody has light and radio, so I prefer this particular house to house publicity to be the best”.

M: What are the good and bad things you heard people talk about the ambulance service?

R: “Well the good thing about the Ebola ambulance service, when some is sick or somebody has die in the community, they will come and pick the person up quickly to hospital or the dead body to cemetery, and the bad the thing/ before, when Ebola started. They were wasting time to pick sick person or dead, and the pressure on the was too much, there were a lot of confrontations, even for people to go inside the ambulance, when they come for them was not an easy one (= thing), unless we talk and advise them before they board the ambulance”.

M: What about the good and bad things about the holding/treatment and community care centres?

R:”Well at the holding centre when they come with a sick person, we will isolate, we give you oral rehydration salt (ORS), until we call the ambulance to come and collect the person”.

M: What about the bad things?

R: “Well, I have not seen, but heard people saying that, when they bring a patient to the hospital, they will not touch you, neither talk to the patient, that is what people usually talked, sometimes I tell them, it is a lie”.

M: What are the good or bad things about the burial teams that you heard people talk?

R: “The burial team have tried so far, because I have not heard any bad talk people say about them. When they come to collect either a sick person or dead people, they dress up, take the swab of the person, they take the person along, and they will make sure one of the family members go with them to the cemetery that is the good thing about them”.

M: What about the 117 Ebola phone line; what are the good or bad things people talk about them?

R: “Well the bad thing about them is, before this started, it was really difficult to get them, people will call and call they will not respond, at time, the dead bodies will be at home for three days before the ambulance come and collect. The phone line was highly engaged, so people were every grumbling about their service”.

M: What about the good aspect?

R: “Well the good thing, people are praising them, that their response is very fast, as you call, they will pick and send ambulance to your place, even if you are up the hills so really people are praising them”.

M: What about the existing health facilities and the staffs that work their?

R: “Well the good thing, they are really trying, they did not abandon patient, and they will talk to them in a good well, give you supportive treatment, until they call 117”.

M: How are people reacting to Ebola survivors in this community?

R: “Well, they are not reacting to them badly”.

M: Have you seen an example of the bad reaction to them?

R: “No, I have heard but not seen”.

M: Like what?

R: “ I have heard a lady survive in this community, when she returns, they welcome her very well, they do not push far away from her, they encouraged her, mingle with her also”.

M: what is the message you have for the other community or people that reacts badly to the Ebola Survivors?

R: “I will tell the other communities, that the sickness is not the wish of people who come in contact or infected, so when they have survived, let’s be happy for them and let them well feel at home. It is really not easy, someone to be infected with this virus and then survived, so we have to encouraged them and be happy with them, it may be anyone of us, it is not their wish to get this sickness. I will tell them that, I am encouraging them in my community, so every should do, because if we push them far away, someone of them will be discouraged by any bad treatment, so let us all encouraged them”.(*a motor bike passing*)

M: Have you heard of any new treatments for Ebola, which may become available soon?

R: “Well, we heard of a vaccine that may be coming for a long time now, but up to now, we have not yet seen it”

M: So what are the things you heard?

R: “I heard that they are working on a vaccine for Ebola, but up till now, we have never seen it”.

M: Have you heard of any new ways to prevent Ebola?

R: “Yes”.

M: What have you heard and what do you think of it?

R: “Those who has infected or not”?

M: Yes?

R: “ they sick when you have not sick with Ebola, always wash your hands with soap and water, avoid body contact, you have to avoid public gathering, anything you do, you have to wash your hands, I think, that is the advice they have been given”.

M: As a health worker, what do you think, is the general knowledge of people in your community about Ebola?

R: “In my community, the knowledge of people about Ebola, the way I think”?

M: Yes, the general knowledge of the people in your community about Ebola?

R: “Well, they are thinking that, this is a bad sickness that has come and we should be careful of it, as I think, first we see the signs and symptoms, like vomiting, high body temperature, fever, so we were assuming it to be malaria, typhoid and cold. So that was my thinking”.

M: so what is the general knowledge of the people about Ebola in your community?

R: “The general knowledge of some people in this community, they do not believe Ebola exist, they said it is malaria, some say is cholera, they say cholera is back, these are their ideas and the way they feel”.

M: What do you think you need to know more about Ebola, so that, you may be responding to question of your people?

R: “I need to know, how this sick attacks someone for the first time and I want to know more signs and symptoms, because most of the signs and symptoms of Ebola is the same with malaria and typhoid, so I need to know, if they have the same symptoms, so when I am faced with questions like this, I will advise them perfectly”.

M: Is there anything specific about Ebola that you think people need to understand better?

R: “They have to understand that, when you start feeling your body paining you, joints pain, getting the signs and symptoms, you have to report at any health centre”.

M: What do you think is the best way to explain this?

R: “Well the best way, I will advise them, when you are sick, go the hospital, if you notice any signs and symptoms of Ebola, you have to report, and so I will advise them”.

M: thank you very much for this interview, we pray that Ebola will come to an end.

**ADDITIONAL PART OF INTERVIEW, OBTAINED BY COLLECTOR 2 AFTER CONSENT IN PERSON, March 2015:**

M: The last time you said the people were stubborn and not serious about Ebola, why were they stubborn and not serious about Ebola?

R: “They were stubborn because the way the sickness came in our country, they do not believe, that made them to be stubborn”.

M: Why they do not believe?

R: “As I told you, we had never experienced this type of sickness called Ebola, so the vomiting and frequent stooling (=diarrhoea) was its signs and symptoms, so we were thinking that it is cholera, when they came and talked to them, they said it is cholera, we had never got this sickness in our country, so they usually said, that really mad people to become stubborn, because this is our first time of getting Ebola”.

M: I made to understand later they changed their minds from disbelief to belief, what led to this change?

R: “The reasons to this change is when they saw that, when you got this sick or when a family member is sick or died with Ebola sickness, when they touch the sickness will transferred to them, so they totally changed their attitudes and say this sickness is true”.

M: Can you remember the month this change happened?

R: “Well the sickness came after two months then they changed their minds”.

M: Around July or August?

R: “August 19^th^ “.

M: What is the first date do you registered your first Ebola case in this community?

R: “May”.

M: I mean (- -name of community- -) or hospital?

R: “Well October”

M: Ok, you again said that some people keep their sick patients at home, why do they do that?

R: “Well they were afraid to come to the hospital, why they were afraid to the hospital because they thoughts when they go to the hospital, they will tell them they had Ebola, so that was the reason people were keeping their sick patients at home, unless we went out sensitizing them, so they started coming with their sick patients at the hospital”.

M: Do you have idea about the treatment they were given them?

R: “When they vomit, they bought ORS, Panadol, novalgin (=painkiller) tablets, they will and buy these medicines at the pharmacies”.

M So the community people were doing this?

R: “Yes”.

M: People were going to traditional for cure, why were they going to the traditional healers or have you ever heard of people going to traditional healers for cure?

R: “No”.

M: What about the other communities and or this community?

R: “No, I have never heard of that”.

M: you said again, they have bad reaction towards people that have survived from Ebola, What do you mean?

R: “First when the people that were sick of Ebola went to treatment and returned survived, people started looking at them in a different way, they were not welcoming them properly and also treated them good, thinking that when they go nearer to them, they will get Ebola, but only when they announced that we should treat the Ebola survivors as part of our community and let’s encourage them, so that stopped the people from reacting bad to them”.

M: If you were an Ebola survivor, what are the challenges or problems do you expect from your people in community?

R: “The problems that I will experience, I will be afraid to mingle with people in community, I will not be visiting public gathering fearing that if I go there, they will not welcome me”.

M: Have they accepted the Ebola survivors or not?

R: “They have accepted them because right now everyone is aware”.

M: So is it the same in other communities?

R: “Yes”.

M: You have not heard a bad news about that in the other communities?

R: “No, everyone have accepted them”.

M: Have you heard of any secret burial or secret washing of dead bodies?

R: “Since the starts of this sick, we have never heard of secret burial, people are only doing secret washing”.

M: In this community?

R: “Yes”.

M: So how were they doing it?

R: “When a person is dead they washed the person quickly before they called 117 or raise alarmed about the death”.

M: Is this secret washing of dead bodies still continuing or they have stopped it?

R: “They have stopped it”.
