# Supplementary material for: Development of a set of community-informed Ebola messages for Sierra Leone
Source: PLoS Negl Trop Dis. 2017 Aug 7;11(8):e0005742. doi: 10.1371/journal.pntd.0005742 (PMC5560759; doi:10.1371/journal.pntd.0005742)
Supplement: S1 Appendix — (ZIP) [file pntd.0005742.s001.zip › Ebola messages - FGD and interview transcripts/R2HC Ebola Fieldwork 1/R2HC Ebola F1 HW-Urban2 V3 CORR.docx]

| CODE | **R2HC Ebola F1 HW-Urban2 V3 CORR(urban semi-structured interview with health worker/volunteer)**  **V2 – 11^th^ March 2015 – ADD PROBE**  **V3 – correction personal data respondent** |
| --- | --- |
| DATE | February 2015 |
| DURATION (minutes) | 16 |
| Collector nr | 2 |
| LANGUAGE INTERVIEW | Krio |

**PERSONAL DATA RESPONDENT**

| Age *(in whole years)* | 36 |
| --- | --- |
| Sex (Female = F, Male = M) – circle | Female |
| Religion | Christian |
| How much time does it take you to walk from your house to the nearest PHU? (minutes) | 35 |
| Mother tongue: | Mende |
| Role in the health facility / health: | XXXXXXXXXX |
| Education level (circle) | Tertiary |
| Do you know anybody who had Ebola? | Yes |
| If Yes, what is your relation to that person? | Colleague |

**TRANSCRIPT: (M= Moderator, R=Respondent)**

M: When did you first hear about Ebola?

R: “I first heard it May, 2014”.

M: How was it described to you?

R: “They described it to me as a sickness that kills people and it has no cure, as soon you get hold of it, you will die so that made us to fear”.

M: What were your first thoughts about it?

R: “At first I was confused, not knowing what to do, because our health experts were not passing the correct information to us, so we too, we were unable to take precautionary measures”.

M: How has Ebola affected this community?

R: “Ebola has affected us greatly, being that, they are not adhering to the rules and regulation, it has created a great impact on them, because plenty people have got infected and some have even died, and most of them were women and children”.

M: Have you person personally seen a person with Ebola?

R: “Yes, I have seen”

M: Do you know the person?

R: “Yes”

M: Why do you think Ebola has spread throughout Sierra Leone?

R: “The main reason is because they are not abiding by the rules and regulations, when they said don’t touch sick person, they will touch, don’t wash dead body, they are still doing it, I think these are the main reasons for the spread of Ebola, because they are sensitizing wide across but people are not adhering to it”.

M: Within this community, do you have a local name to describe Ebola?

R: “No, I don’t know”.

M: Some people do not believe Ebola exist, do you have those type of people here?

R: “Yes, One”

M: Do you know why they have this view?

R: “Why, at one time when we come for work, we met two dead bodies lying and you know medically all death cases are classed as suspected until they are tested and confirmed negative, so when we came and met the two dead bodies, when we said we are not working because of those dead bodies, they should come and collect the swab for testing. Most of the people start saying we are lying, that lady went and did abortion, and in fact there is no Ebola. We told them, you did not still believe Ebola exist, with all you are seeing people dying including your love ones dying in this community. But in this community, those who believe, outweigh those that do not, at least 75% believe Ebola is real and the others do not”.

M: Have you heard of Ebola message?

R: “Yes, I have heard a lot”

M: Please can you give me some examples of the Ebola message that you have heard?

R: “I went for a workshop; they told us about the preventions and how to avoid Ebola”

M: What are the preventions?

R: “Well, the preventions are, don’t touch, don’t attend burials, and don’t wash dead bodies, wash your hands at least five times or as much as you can”.

M: What are the signs and symptoms?

R: “The signs and symptoms, we have the wet stage and the dry stage, for the wet stage, we have vomiting, frequent stooling, in the dry stage, we have red eye, sore throat, fever and weakness”.

M: What do you think about these messages?

R: “They are really in place if we work towards the preventives aspects that will stop Ebola, for the area of treatment, if you have notice the signs and symptoms of Ebola, consult the health personnel for advice, because I believe that earlier treatment, more chances to survive”.

M: “Do you understand all those messages well?

R: “Yes”.

M: Do you accept the messages

R: “Yes”.

M: What do you think would be a good message to encourage people to bring their patient to the hospital?

R: “As a medical expert, I will talk to the person, persuade and encouraged the person, I will not force the person to the hospital, I will tell them that not all sickness is Ebola, maybe it malaria or other diseases that has the similar signs and symptoms of Ebola, so go the hospital now and get better answer, if it is malaria you will be treated for it, if it is Ebola also, be rest assured you will be taken care of. So I am encouraging you to go to the hospital, the earlier you go, the more chances you have to survive”.

M: What do you think would be the best channel to get your new Ebola Messages?

R: “All the channels are important, like the media, sensitization, and hand bills, all of this means are important, because the messages will reaches far and wide”.

M: Do you think of any other new channel which should be used now?

R: “No, all of them have being exhausted”.

M: In the event of Ebola infection, do you think people would prefer to go first to the traditional healer, or the treatment centres?

R: “For me, to start with if the person decides to go to the traditional healer, the sickness will spread more, the person should go to the holding/treatment centres and hospitals”.

M: So that is what you think the person would prefer?

R: “Yes, that I think the person should do because at that moment the person do not know his or her condition either negative or positive, if the person decided to go to the traditional healer it will be the worst”.

M: From the general perceptive of the people, what would you think they prefer to go?

R: “Well to be honest some communities are watch dogs for others, if there is anything like that, they will be the first people to inform the health workers, they will call and forward complains, whilst the other communities decide to stay mute and don’t talk. There was a case at the XXXXX XXXXX, which I will always take it is an example, most of the deaths that occurred here are people from another community, they may not want die in their own community”.

M: Have you heard people talking good or bad about the Ambulance services?

R: “Well at first, we were hearing bad news about them, like the delay in collecting corpse or sick people, when someone dies, it will take days for them to collect the dead corpse from the house. But as it went along December and January it was perfect, they come on time. Like in the ( - - another community in the interview district - -), because we have surveillance office, at times they will call them call them in two different location to pick up dead bodies, they will response to both at the same time. They are fast and they are really doing well”.

M: Have you heard people talking good or bad about Ebola holding and/ treatment and /or community care centre?

R: “Well at first, when there was no structured mechanism, there were a lot of complains, that they were not treating them properly, but now it is really better”.

M: What about the burial team, have you heard any good or bad people talk about them?

R: “Well, at first we heard bad news about them, but now, it is better”.

M: Like which type of bad news people talk about them?

R: “Most of the time when there were cases, they will not come earlier, and they have told the people to avoid touching of dead bodies, we were having complains about that, in December a boy died in his house at night, so they call 117, because it was too late and out of working hours, the people decided to lock the his door, so in the morning, the burial team came and collected the dead corpse. They are really working well”.

M: What about the 117 phone line?

R: “Well they are also working well compared to previous days; they respond immediately as you call, previously, there was a lot of delays in responding”.

M: “Any aspects of the existing health facilities/staff that is now working on the Ebola care and treatment centre?

R: “I have not heard about any negative complained, because I am not working there. I worked at the maternity section, there, the patients were complaining that we are not touching them, checking their pressure, but we have be advised not to touch, previously, that was the grumbling amongst pregnant women and suckling mothers. It is better now we touch them, because we put on our personal protective equipment (PPE). We even child deliveries and all the other normal check-ups procedures”.

M: Do you have any Ebola survivors in this community?

R: “Yes we have plenty of them”.

M: How do they react to them in this community?

R: “At first, they were stigmatizing them, but the youths in the community gathered together, going house to house, sensitizing people against stigmatization of the survivors, telling the community people, they should take them as brothers and sisters, they are heroes in the Ebola fight, it is not easy to be infected with Ebola virus and survive, they should not be provoked, they should be encouraged, and the most surprising issue, the survivors themselves took on the street sensitizing others, they should report to the hospital when they feel sick, they should avoid body contact, wash their hands frequently, they don’t need to be afraid, earlier treatment gives great chance to survival, this was their message to the community”.

M: What is your message to those people that stigmatised survivors?

R: “They should stop the stigmatization, as it is not good, stigmatization is even more stressful than the Ebola virus itself, they should encourage them and take them as brothers and sisters in their community”.

M: Have you heard of any new treatment for Ebola that may become available soon?

R: “I have heard of it, but I have not seen it, I heard that there is a vaccines they will be coming but I have not yet seen and I don’t know, it is just by “air say”(= rumour).

M: Is there any new ways to prevent Ebola?

R: “No, so far so good we only have the old preventives measures”.

M: What are they?

R: “They said don’t touch, don’t this, don’t that, that is all, I have not heard of any new ones”.

M: You said you heard of vaccines but you have not yet seen it?

R: “mmm”. (No)”.

M: But you heard of it?

R: “Yes, I heard of it”.

M: What are the point of discussion of people about Ebola, like their fears, confusion and worries?

R: “The discussions that I have witnessed once or twice, the issues of Ebola which people are more worried about, is the survivors, at least when they take you to the holding centre and to treatment centre, their worries here is, the concentration or attention at treatment centre most be emphasised to get more survivors in this Ebola fights, that is their worries, but to God be the glory, we have got a greater number of survivors, because some people are saying “aahh” let them leave them alone if even they take you to the hospital you will not survive, you will die, and other people are saying even the nurses that they took to the treatment centre did not survive, what about we the ordinary people if we go to the hospital when we feel sick , we will also die. So their concern is, why people go to the hospital earlier when they had Ebola but still die? But thank God people are now surviving when taken to the treatment centre”.

M: Is there anything specific about Ebola that you think people need to understand better or yourself?

R: “What I need to know, because the time we went for a workshop at the national school of nursing, the trainers asked us two questions, one objective question they asked, if Ebola is manmade virus or natural something, then we said it is manmade, when we asked then they just shook their heads and smile and did not answer. Then we asked then a question, you said the chain of transmission of Ebola, is from the monkey, the monkey will go up the tree eat the mango then the hunter will go and kill the monkey, take it home, his wife and children will shared, neighbours will buy and so on, why the host is not infected but we the human beings, the trainers could not answered, but there was a doctor in our class (- - name of doctor - -), answered the question, he said why the host is not infected because their DNA is different from the human being, so we learnt a lot about that, he elaborated on that, and made us to understand better. So I said to myself why the bats eats mangoes and later the monkey and the hunter kills the monkey. All of them interact, but only the hunter as a human being will be infected with the virus”.

M: Is that the part you want to understand or you have understand it better?

R: “I understand it better, but it was that was only new thing I learnt from the workshop”.

M: So is that all you want to know, you don’t have any other thing you need to know, to be responding to questions?

R: “Well, yes because Ebola is a deadly disease, I want to know if there are other preventive measures rather than the old ones, and if they are working on ways and means to get a specific treatment for Ebola”.

M: Ok, I thank you

**ADDITIONAL PART OF INTERVIEW, OBTAINED BY COLLECTOR 2 AFTER CONSENT IN PERSON, March 2015:**

M: The last time you said the health experts where not passing the correct information to the people about Ebola, what do you mean?

R: “Well, I mean, the health worker were passing the message, but the people were not accepting the message”.

M: Why?

R: “Because they do not believe Ebola exists or it is real”.

M: Why they do not believe that Ebola exists at that moment?

R: “At that moment the health workers were telling them that Ebola is real and it exists, but they denied it and did not accept the fact, they do not believe Ebola exist, people were taking it as a tradition, people had different ways they view it”.

M: Why the people changed their minds that Ebola exists?

R: “Why they changed, they have seen the reality, they have seen examples of the problems Ebola has caused, and when you go against the precautions, like when people died, when they told them not to wash dead bodies, they were doing that, so later when they noticed that, because they are doing these things and it is leading to the death of people so they stopped and changed their minds, from not believe to believe”.

M: In which month did the people changed their beliefs or minds that Ebola is real?

R: “For the past four months”.

M: That is when?

R: “It was in November”.

M: You said last, you were having two dead bodies in this hospital, what happened with them?

R: “Definitely we did not know, what happened to those dead bodies, when we came, we did not even enter in the compound, we stopped at the street, some of our colleagues called us and gave us the information, so when we came, about forty staffs, we were all standing outside, when we were standing outside, a team of journalists from Sierra Leone Broadcasting Cooperation (SLBC) and other media houses came, so when they were asking, that was the time we made to understood that , one of them, the woman died from abortion, so the bleeding led to her death and the other person was a young baby, they said he passed off at the home of the parent, before they called 117, instead they brought the baby at the hospital, so we met those dead bodies, when we called 117, they came and took the dead bodies and they sprayed the place”.

M: Have you heard or know about people that go the traditional healers?

R: “Yes”.

M: Where they going there?

R: “Yes”.

M: In this community?

R: “Well in this community, I don’t know of any”.

M: But what about the other communities?

R: “Yes, I have seen it on a Television programme “We nus” (our news), communities like (- -name of community - -)”.

M: Where the traditional healers healing the sick people?

R: “No”.

M: You also said people were stigmatizing the Ebola survivors, who are these people?

R: “The community people”.

M: What were their reasons of stigmatizing the Ebola survivors?

R: “It is just about nature, some people when they are not in talking terms with an other people or had a grudge that exists between them and the other person(s), or had a quarrel, when a person(s) has been infected with the Ebola virus disease and survived, when the person is back in the community, they person(s) will be stigmatized by the people they were having grudged, they will looking at you differently”.

M: What do you think will be problems that the Ebola survivors encounter in their community?

R: “Like how I said last, it is just the stigmatization, they stigmatized them, pointing fingers at them”.

M: How are they treating them presently, are they pushing far away from them?

R: “Presently they are not pushing away from them, like even they organised a programme here, at the programme, there two survivors with them, moving from house to the other sensitizing them, this program was organised by (- - name of organization - -) , telling people that they should not point fingers or stigmatized Ebola survivors, they have to embrace them, they had to give the survivors an applaud, that they are heroes, they had gone through the fight and had come”.

M: So they have been accepted in the community now?

R: “Yes”.

M: Have you heard of any secret washing of dead bodies in this community?

R: “No”.

M: What about other communities?

R: “Yes, other communities, like how they says it on the radio, so I heard”.

M: What have heard about secret burials or secret washing, after which they called 117?

R: “Yes, they even showed us in a television programme called “We nus”, they washed the dead then they later called 117”.

M: The way they treats Ebola survivors in this community, is it the same way they treat them in other communities?

R: “It is just the same treatment, like us here, when they come, showing up one or three symptoms of Ebola, we will first isolate them and later call 117”.
