# Supplementary material for: Development of a set of community-informed Ebola messages for Sierra Leone
Source: PLoS Negl Trop Dis. 2017 Aug 7;11(8):e0005742. doi: 10.1371/journal.pntd.0005742 (PMC5560759; doi:10.1371/journal.pntd.0005742)
Supplement: S1 Appendix — (ZIP) [file pntd.0005742.s001.zip › Ebola messages - FGD and interview transcripts/R2HC Ebola Fieldwork 1/R2HC Ebola F1 HW-Urban3 V2 CORR.docx]

| CODE | **R2HC Ebola F1 HW-Urban3 V2 CORR (urban semi-structured interview with health worker/volunteer)**  **V2 – 11^th^ March 2015 – correction personal data** |
| --- | --- |
| DATE | February 2015 |
| DURATION (minutes) | 25 |
| Collector nr | 4 |
| LANGUAGE INTERVIEW | Krio |

**PERSONAL DATA RESPONDENT**

| Age *(in whole years)* | 30 |
| --- | --- |
| Sex (Female = F, Male = M) - circle | Male |
| Religion | Muslim |
| How much time does it take you to walk from your house to the nearest PHU? (minutes) | 10 |
| Mother tongue: | Temne |
| Role in the health facility / health: | XXXXXXXXX |
| Education level (circle) | Tertiary |
| Do you know anybody who had Ebola? | Yes |
| If Yes, what is your relation to that person? | Friend |

**TRANSCRIPT:**

M: When did you first hear about Ebola?

R: “I heard about Ebola, last year, 2014 around May to June”.

M: How was the disease described to you?

R: “They described it as a bad sick, and it caused by a virus, it is a breakout disease that has just come, and you have to prevent yourself from it, how? You don’t have to touch, when someone is sick of Ebola, you have to be careful getting direct contact with the individual”.

M: What were your first thoughts about it?

R: “I thought that, this is a bad sickness that has come, because in our country when someone has got sick, for example a relative you have to be by the person, but they have said, when someone is sick of Ebola, you should not go near the person, I was doubtful and I ask myself what kind of sickness is this that has come in our country, how does it look like”.

M: In what ways has Ebola affected your community?

R: “It has affected us greatly in (- - name of community - -), because they came with the virus, like what we heard, a woman came from up country on a business trip and they lodged her in a house down the (- - name of community - -), and she got sick, the woman was a traditional healer, so her colleagues traditional healer came to provide her healing, so within that moment, they were touching her, not knowing that it is Ebola, since then all those that get in contact with them got sick of Ebola, because after her colleagues have tried but to no avail they decided to call a pastor, the pastor himself got sick, everyone that got direct contact with her fell sick, more than thirty and after tracing all the contacts in this area, more than seventy suspected cases and after tested, thirty confirmed cases, and most of those confirmed Ebola positive that went to the treatment centre, only few them returned all the others died, plenty houses were quarantined, though the quarantine period has almost finished and the virus is still around, we are still afraid”.

M: Have you personally seen or know people who have had Ebola?

R: “Yes I have seen a person that have had Ebola, like the boy which contacted the virus, they came and collected him, we were sitting together at the “Ataya base” (= A place where youths gather together and get fun), when we noticed that he has contacted the Ebola virus disease, we went far away from him, everybody stopped associating with him, we started talking about it, so the boy decided to go home and later the Ebola team came and collected him, took him to (- - name of Ebola treatment centre - -) centre, he survived and came back, he is here with us now”.

M: Why do you think Ebola has spread throughout Sierra Leone?

R: “The reason for the spread of Ebola is because the virus is been moved with, that is reason number one, when somebody has caught the virus, hence the person decided not to stayed in a stagnant position but moved with it from one place to the other, with this movement for place to place, another person will be fortunate to catch the virus. On the side sickness, once you have contacted the virus anywhere you go, when you have been touched by someone, the virus will transfer and that person may catch sickness, especially when someone has vomited and you touched dead bodies, immediately you will catch the sickness, this is the way the virus is being spread “.

M: What do you think is the best way to prevent Ebola from spreading?

R: “The only is, people should put in the practice the precautionary measures stated by the medical people that is, don’t touch, don’t visit a sick person, when someone is sick, take the person to hospital, even if it happens during the late hours of night, make sure before touching the person, you use gloves or plastic to protect yourself as how they are telling us the precautions”.

M: As a health worker, what do you think is the best way to treat somebody with Ebola?

R: “As a health the best way to treat somebody Ebola, when a person has contacted the virus, having seen the signs and symptoms, I will not touch that person, even if I am to go around the, I have to protect myself from the person, I will used protective gears until I take the person to the hospital”

M: Are there any local terms that use to describe Ebola?

R: “Yes”,

M: What are these terms?

R:…………………………..( to listen and transcribe)

…………………………………..

M: Some people do not believe Ebola exists. Do you know people in this community who think this?

R: “Yes, we were having people that do not believe Ebola exists”

M: Do you know why they have this view?

R: “Well, some people said this virus was brought by people and it is a strategy of the government to generate money, some are politicizing it, they said government wants to generate income, we were only having malaria, and typhoid which has the signs and symptoms of vomiting, frequent stooling(=diarrhoea) so they are lying, Ebola do not exist, but until when we had the first case in our community then people came to realised and believe that Ebola is real”.

M: Please can you give some example that you have heard, seen, or read?

R: “Well yes, people were go house to house telling us to wash our hands with soap and water, let use chlorinated water, we have to use hand sanitizer, they said we should also use Dettol to protect our self, we should have bucket of water at your house for visitors to be washing their hands, they were having hand sanitizers sharing it to people in the community, to be using them, for in case you touch any place that is not pleasing, for instance vomit, saliva, you should quickly rush and wash your hands , so you will protected from the virus that moment”.

M: What do you think about these messages?

R: “Well the message is fine, it prevents you from a lot of things, that some of us that have adhere to message, it has prevented us from cont(r )acting the virus”

M: What do you think about the way of disseminating these messages?

R: “The way they are given us the messages are good, they are not doing it violently, although others will come and forcefully give us the message, whilst others will come peacefully and pass the message for you accept and understand the message”.

M: What do you think has been the best Ebola message you have come across to date?

R: “The best one I have ever heard and I believe that it will work, is the way of preventing yourself from the virus, when you have seen virus, you prevent yourself from it, when they said don’t touch a someone that has Ebola, don’t go around the person, when the person is sick, call the team to come and collect the person, that is best rather than playing the person, maybe the moment you touched the person you have contacted the virus and after one to two weeks you have fall a victim”.

M: Are there any Ebola message that have not worked so well?

R: “Because all the messages they told, most of them worked well, I don’t believe if there is any one which have not worked so well, all the message came in different ways, so I don’t believe if there is a message that we did not take granted”.(*people talking at the background)*

M: As a health worker, what do you think would be a good message to encourage people to bring patients to treatment centre?

R: “Yes”

M: What do you have to tell them?

R: “First, I will counsel the person, because in this situation, some people will feel frightened, having in mind that they are going to inject or kill them, in situations like these, I will call the person and say you have to be patience, as it has happen with your relative, let us take the person to the hospital, let do it fast, don’t cry, if you cried, the one you are crying for will be frighten and developed stigma, and with this stigma maybe when the person is taken to the treatment centre will not come back, but if you talk to the person in that encouraging mood, the person will be courage and have hope and the go and come back cured”.

M: As a health worker, in the event of Ebola infection, do you think people would prefer to go first to a traditional healer, or existing health facilities, or the newly established Ebola health facilities?

R: “This depends on the type of individual, the one that does not have the medical know-how, when the person come in contact with the virus if he/she is in the village or up country, the person will decide to go to the traditional healer or any other sorcerer, but rather the health worker who is civilized or any other person that have that intelligence when they think they have had Ebola, the person will straight away go to the pharmacy or rather go to a nurse to get medication, the person will have it in mind to go first to the hospital”.

M: Why do you think people decide to go first to pharmacy and later hospital?

R: “At that moment the they will not have been convince that they have contacted virus, so they will continuing taking treatment themselves until the sickness got worsen, that would be the time they will go to the hospital”.

M: Why do you think people go to the traditional healer?

R: “Well some people believe in traditional practice or medicine, they believe that when they go to the traditional healer and rubbed the traditional medicine they will get heal, they will have these beliefs, they believe that when the sorcerer pray for them and rubbed the traditional medicines and shake their traditional medicine bottles they will get healed”.

M: Some people stay at home when they think they may have Ebola, why do you think, this is?

R: “It is because they don’t have someone to advise them that they should get up and go to the hospital, at the when people get aware that the person is sick to be carry to the hospital, because some people are like creoles very conserved, they will be in their houses alone, they do mix-up with different people, if that kind of person be a victim, before people get the awareness, the sick as got worst, maybe in the process of taking the person to the hospital the person had died”.

M: What do you think have to be done to encourage those kind of people to go to the hospital?

R: “In the event like that, you have to the talk to person, admonished the person that has already infected with the virus that you should go to the hospital, for the sick not to get worsen which endangers your life”.

M: What do you think would be the best channel to get your new message to people?

R: “The number one channel is by telling the people to go the hospital when they are sick, immediately they have noticed that they are feeling continuous head ache they must see the a person that is medically oriented that person will lead them to the hospital before the sickness overcomes them, that is what we should tell them”.

M: What is the other channel?

R: “When you have noticed that you have got the signs get up by yourself and go to the hospital and complain to the doctor, your whole system is not okay, they will assist you better”.

M: What channels will be used for the messages to reach the people in the villages and by the ?boarders??

R: In cases like that, I prefer the town Clayeyer (town crier) , the chiefs and other stakeholders like the youths in the villages, you should tell them that, this is what we want you to tell our people”.

M: Have you ever heard people talking, either good or bad way about the Ebola ambulance services?

R: “The good aspect about them is the fast movement, in situation when someone is sick, they will collect and rush up with the person faster to the health centre for medication, and also they do not stock in traffic, as they hear the voice of the siren, they will allow them to pass”.

M: What are the negative aspect about them?

R: “The negative aspect is the delay in their response to come pickup either the sick or the dead person, when they called upon, and at times, no sooner they board a person in the ambulance they will spray and some of the people that are allergic to chlorine will die, before they arrived, the chlorine would have overcome the person, the person had become weak and the another bad aspect again, they will inject you whilst in ambulance then you will die even if is not virus the you are having”.

M: Have you ever heard people talking either good or bad about holding centre/treatment centre/community care centre?

R: “Well what people are talking bad about the holding centre, they said as you have been taken there either you are having Ebola or not, when you laid down to sleep they will inject you on the biggest toe of your foot, so after injecting you before morning comes, you are died, they said because they want to attained the amount death cases the World Health organization Projected and also because the Ebola is getting to the finish line, they want to increase it again by this, they will used some certain virus which resembles Ebola virus, so the more people at the centre, the more money they get”.

M: Where did you get this information?

R: “From the people, most times as health workers when we are among them, they will say just leave us alone, you guys are benefiting from the Ebola Fund”.

M: In your own experience as health worker have you ever suspected someone doing carrying out this type of deal?

R: “No, I not seen someone doing so”

M: So it is just by Rumours?

R: “Yes”.

M: Have you heard people talking good or bad about the burial teams?

R: “Well they said, the burial team the way they dressed it not easy for them to be infected, and they bury dead bodies at their own time, I mean, dressing, putting into the grave it is done on time”.

M: So what about the grumbling aspect?

R: “They said people are buried in mass grave, one, two and three per grave and in that process they will just swing the dead bodies’ disrespectful in the grave, some family members present at the grave side will cry and feel aggrieved because of the disrespectful their relative was put into the grave”.

M: What about the 117 phone line, is there any good or bad people are talking?

R: “The good aspect, they said when they called them they will response quickly, the bad aspect is, sometimes when they called them, there is a delay in their response, they said why the late response, they may want to assess the gravity of the sickness and at times, when they come, some patient spit on them, for this reason they may delay so that before the arrive the patient has become weak, to avoid confrontation with the patient”.

M: Any aspects of the existing health facilities/staff that is now working on the Ebola care and treatment?

R: “There is no bad attitude about the staff, because we know how to talk to the patients better, when you talk to the patient well that will give the person the courage to respond well to treatment, these are part of their ethics they learnt”.

M: What about the bad aspect, you don’t heard people talking about it?

R: “They said the staff are not talking to people properly, in the night hours they don’t see any of the staff, they will just leave on their own and go about their business”.

M: Is this kind of behaviour of staff is happening in at your centre?

R: “No, we are always present”.

M: How are people reacting to Ebola Survivors in this community?

R: “In this community the one Ebola survivor we have, people are encouraging him, joke and play and with him, he has never been treated badly, we play card, draft to together and he shared with us his experience and encounter is at the treatment centre, when he was taken from the red zone to the recovery room all of those things he shared”.

M: “So they are not stigmatized or treated badly?

M: Have you heard of any treatment for Ebola that may become available soon?

R: “Yes I have heard of it in Liberia that it is being made as sort of vaccines that will be administered to someone, so even if you cont(r )act the virus, it will not overcome you”.

M: What do you think about these vaccines?

R: “As for me who is medically oriented, I will not take it any other way rather than a vaccine to protect myself, but for some other people, they will not take the vaccines fearing that it’s another virus they want to inject into their system for them to die”.

M: That is the concern of the people?

R: “Yes”.

M: Have you heard of any new ways to prevent Ebola?

R: “Yes, the only new ways to prevent Ebola, is to quarantine immediately any area that is has a new confirmed case of Ebola, let there be total restriction of movement of people, from one end to the other until all contacts have been traced”.

M: You said you heard of the Ebola Vaccines?

R: “Yes”.

M: As a health worker, what is the general knowledge of people about Ebola in your community?

R: “In this community there are some people who believe Ebola Exist, there people who did not still believe Ebola exist, they said the issue of Ebola is money generating by the government till now, they don’t believe, does who have not contacted the disease”.

M: As a health worker, what do you feel you need to know to enable you respond more to misconceptions, doubt of the people?

R: “Well, I will tell them, they should believe that Ebola is real, it is no artificial plans, just think of the illness like malaria when you are bite by anopheles mosquito and you get malaria, just believe that it is real”.

M: Is there anything specific about Ebola that you think people need understand better?

R: “Yes, the prevention aspect, people need to prevent themselves, for instance, if you have notice that this particular person has Ebola, limited yourself from touching and playing with the person to prevent yourself from getting Ebola”.

M: What do you think would be a good way to explain this to people?

R: “Well we have a lot of ways, In villages or up country use the media, and in some remote areas in which you find yourself use the town informant to pass on the message using the megaphone, gathered people together at the town “barry” (= an open space where people gather for town meetings) and pass on the message, move from house to house at night, also go to mosque and churches to pass on this message”.

M: What aspect of the media you are talking about?

R: “The Radio, or newspaper”.

M: As a health worker, have you heard or seen people doing secret burial?

R: “No, I have not seen or known about secret burial, and I don’t even believe because we don’t place to bury in this community, you take not throw dead bodies in sea or burial at the back of your house, someone will definitely see you, they usually called the burial team”.

M: What about the secret society members are they not doing secret burials?

R: “No, it is not happening now, even if it had been happening but not now, everybody is a watch dog for each other in this community, anything that went wrong they will call and inform authorities, in fact you may not know, who did the call”

M: I thank you very much for taking you time?
