# Supplementary material for: Development of a set of community-informed Ebola messages for Sierra Leone
Source: PLoS Negl Trop Dis. 2017 Aug 7;11(8):e0005742. doi: 10.1371/journal.pntd.0005742 (PMC5560759; doi:10.1371/journal.pntd.0005742)
Supplement: S1 Appendix — (ZIP) [file pntd.0005742.s001.zip › Ebola messages - FGD and interview transcripts/R2HC Ebola Fieldwork 1/R2HC Ebola F1 HW-Urban4 V2 CORR.docx]

| CODE | **R2HC Ebola F1 HW-Urban4 V2 CORR (urban semi-structured interview with health worker / volunteer**  **V2 – 11^th^ March 2015 – correction personal data** |
| --- | --- |
| DATE | February 2015 |
| DURATION (minutes) | 14 |
| Collector nr | 2 |
| LANGUAGE INTERVIEW | Krio |

**PERSONAL DATA RESPONDENT**

| Age *(in whole years)* | 30 |
| --- | --- |
| Sex (Female = F, Male = M) - circle | M |
| Religion | Muslim |
| How much time does it take you to walk from your house to the nearest PHU? (minutes) | 2 |
| Mother tongue: | Krio |
| Role in the health facility / health: | XXXXXXXXXX |
| Education level (circle) | Tertiary |
| Do you know anybody who had Ebola? | Yes |
| If Yes, what is your relation to that person? | Community member |

**TRANSCRIPT: (M= Moderator, R=Respondent)**

M: When did you first hear about Ebola?

R: “I heard about Ebola since May”.

M: Which year in May?

R: “May 2014”

M: How was the disease described to you?

R: “They described it as a disease that kills human being, and you cannot see the virus with your naked eyes”.

M: What was your first thought about it when it was described to you?

R: “I did not believe it; I thought it was a propaganda by people”.

M: How has Ebola affected your community?

R: “Ebola has affected this community more, compared to the other communities, the (- - name of interview community - -) community”.

M: In what ways has it affected this community?

R: “We have about sixty people that were affected by Ebola; we have twenty-one deaths, forty-two survivals”.

M: Have you personally seen a person who has had Ebola?

R: “I have seen so many people with Ebola, and it was myself that removed most of them and referred them to the hospital, because despite being a contact tracer and also in charge of the surge”.

M: Why do you think Ebola has spread throughout Sierra Leone?

R: “Denials, traditional beliefs and love”.

M: What do you think is the best way to prevent Ebola from spreading?

R: “The best ways to prevent Ebola from spreading is plenty

M: What do you think is the best way?

R: “You have to stop touching people, whether they are sick or not, you don’t have to touch them, that is one, when somebody is sick call 117 the call centre, they will come and collect the person, or if you are not able to call, if you have a contact tracer around, link up with the contact tracer or the DSO, the CDC any one of them, or we have social mobilizers you can either contact them, These are the ways to prevent Ebola, and don’t removed dead bodies after burial or exhumed and finally don’t touch someone that had died”.

M: What do you think is the best way to treat somebody with Ebola?

R: “The best to treat somebody with Ebola, as a matter of fact, if you have already knew the person that had got Ebola, but like definitely when somebody has been infected with Ebola, will not be with you but at the holding centre, and when the person is at the holding where they will confirm that the person is Ebola positive or negative, after, they will referred the person to the treatment centre”.

M: How to treat an Ebola infected person?

R: “You do not have any way to treat the person, because the person is not going to be with you”.

M: In this community do you have any local names to described Ebola?

R: “No, we don’t have any local terms; it is Ebola, no other name”.

M: Some people do not believe Ebola exists, do you know people in your community?

R: “If there is any other community which people believe that Ebola do not exist, it is not in this community, in this community when Ebola broke out during November, it hits them very seriously, in fact it hits all of us, which nobody can have that believe that Ebola do not exist, everybody in this have the believe that Ebola exists, because some of them lost their mothers, sisters, friends, even myself it touches me, I have people that died in the hands Ebola”.

M: I know you heard a lot of Ebola messages, which ones you have heard?

R: “Well plenty, I have heard of wash hands, do not touch dead bodies, do not touch sick persons, when somebody is sick call 117, and the messages are plenty that I have heard about Ebola”.

M: What do you think about these messages? Are these messages clear to you?

R: “The messages are cleared, is just that people lack the understanding, this is the problem, and the only problem we have now is the survivors, the survivors, the messages given to them that when they are back in the community they should not touch, some of them are still touching, so I believe these are some of the ways that is still militating towards the end of Ebola. If it is messages, everybody have the awareness now, even the people that did not go to school have the awareness that Ebola is real and it exists and you should not touch; these survivors which are young boys which cannot abstain for the three months, they told them to abstained, but this is the problem”.

M: Which Ebola messages are the best that people have accepted already or which ones have gone down well to the people?

R: “All the messages are the best, because there is no one that is more value than the other, they are all valued, I believe all is accepted”.

M: Do you have any message that did not work well?

R: “No, all worked well”.

M: What do you think would be a good message to encourage people to bring patients to a treatment centre, holding centre or community care centre?

R: “In the first place, if you love someone, you will not love the person more than yourself, so my first message to that person is that, if this person has got sick and you do not want to take the person to hospital, you may get infected and another person may get infected, so the best way is to take the person to the hospital, you should not love another person more than yourself, that is the first thing you have to do, so when someone is sick, advise the person to go the hospital or you call 117 to come and collect the sick person, or you call 300 for Red cross to come and collect the person”.

M: Ok, in the event of Ebola infection, do you think people would prefer to go first to a traditional healer, treatment/holding centre?

R: “As a matter of fact, they have stopped all herbalist, traditional medicine men, all the treatments in the corners (treatments at drug store or home of a medical personnel) they are not existing again, even some of the places where drugs/medicines were sold, they are not selling again, now unless you go to (- - name of big hospital - -) or (- - name of a hospital - -), these are the only places, I don’t believe there are other places they treat people again”.

M: But do you think, when some is sick, the person will go first to the hospital?

R: “No, the person will go straight away to the hospital, if you go to the hospital, because I have taken people to the hospital they did not accept them, when someone is sick, you have a way a strategy you use, that is, we have a command centre, they have a chain work, you will first call, when you called if they did not answer your call, you have the contact tracer around, the councillor, you have social mobilizers, because if you go the hospital, they will not accept you, for example you are sick, when you go to Connaught hospital they will asked you, if you have done an Ebola test, they will send you back to the holding centre to go and do your test”.

M: In that case, do you think the person will first call the contact tracer?

R: “Is either, the person call 117, contact tracer, social mobilizers or the councillor around the community, if the person do not have phone, the person will called other people to help”.

M: Some people stay at home when they think they may have Ebola, why do you think this is?

R: “Some people are afraid, some are ashamed, some for love, some people are afraid that when they are taken to the holding centre their test result will not prove negative, that is what they are afraid of?

M: So what do you in circumstances like that when they are afraid to go the hospital? (*Mobile phone about to ring*)

R: “We have being going on radio, going to the streets, sensitizing people, telling them that, if they have sick people, let them bring the sick person outside, personally I have being checking people in their houses, is not when a person is quarantined, we have being tracing some people we have information about, I have done that many times”. (*An undue noise at the background*)

M: What do you think would be the best channel to get your new messages to people?

R: “The media, the social mobilization”. (*An undue noise at the background*)

M: So these are the best and the fastest?

R: “Yes” (*An undue noise at the background*)

M: Have you heard people talking good or bad about the ambulance service?

R: “I have heard good talks and I have heard bad talks”.

M: Which is the good and which is the bad?

R: “The good talk is, some people said when they go there, they are well treated but before, they are not treated well, when they come and collect a person, when they tell them to disinfect the place where they took the person(s), they will grumble, saying that is not their responsibility, they have a disinfectant team which is responsible to disinfect places they removed sick patients, secondly the time the sick got worst, I called 117 and talked to the manager or whoever, they sent a team to disinfect this entire community, the team that came do not disinfect the entire community, they only disinfect where they took the patients”.

M: Ok, what about the holding centres any good or bad?

R: “They are just the same, they are praising ( - - name of the centre - -) centre, they said there, when you are returning home as survivor, all what you were having that was discarded, part of it will be given to you, like the other centres (- - name of centres - -) they wouldn’t refund anything to you, but they are all treating people good, because if somebody has been taken to the holding centres and returned back survived, it means they are treating people good”.

M: What about the burial teams have you heard any good and bad about them?

R: “No I have not yet heard any bad about them”.

M: What about the phone line (117)?

R: “Well the 117 phone line, I have called them personally, they respond, if even they do not come the same day to collect the patient, the other day they would respond back to your call it has happened plenty times, they are responding well and they are passing the message on well, but only the chain of command is slow but they are passing on the message, but you know Sierra Leoneans have negative habits”.

M: Any aspects of the existing health facilities/staff that is now working on Ebola care and treatment, have you heard any good or bad things about?

R: “I have not heard anything, rather it is slow when the Ebola is plenty, when we were having overcrowding, the command centre were at first allocating patients but it reaches a time the holding centres were allocating patients, at the time when we had a lot of treatment centre, they were just calling directly, so that was the issues we were faced with at the time when you were having (??name of treatments??). That was the problem”.

M: Since you have survivors. How are people reacting to these survivors in this community?

R: “Well in the first place, the councillor will be the first person that will greet and hugged, he will called a meeting and introduced them as our heroes, most of the time when we are doing our social mobilization, we will make them part of the team, some of them have got fitness, some are young boys and girls, we will joined them to our teams and they will at the frontline because they will not get the sick again. They are highly accepted in community, there is no stigmatization in this community, I don’t know for the other communities”.

M: Have you heard about any new treatment for Ebola?

R: “Ebola do not have any new treatment, the only way you will cured by the power of God, they only treats with affects you, if you complain your head, or malaria that is what they will treat”.

M: You have not heard of anyway?

R: “No. I have not heard of any way, I have heard only the Zmap, but I don’t think it has come into effectiveness”.

M: What about any new way to prevent Ebola?

R: “I don’t think there is any new way that is out”.

M: What about the vaccines?

R: “That is the vaccines I told you about, zmapp, but the zmapp did not work first, because I frequently listened to news, they said to make Ebola vaccines it would take ten years and Ebola is getting done in this country

M: by the power of God said by moderator

R: so how can we wait for ten years”.

M: As a contact tracer, what do you do think people discuss about Ebola, their fears, worries and misconceptions?

R: “People are discussing differently, people are saying the daily Ebola update result is given out by NERC (National Ebola Response Centre), and some think it otherwise, but to me that is doing the job, I always tell them the truth, say this is wrong, sometimes you have to be compulsorily believe it is real, I have made some many examples, if don’t believe Ebola is real follow me to the frontline to touch an Ebola patient and see, if nothing happens with you them you go by your believe. Most of the people that deny in our community died”.

M: Do they have any fears?

R: “No. for me, I don’t have any fear, because I have dealt with many cases, Ebola news, Ebola positive, Ebola survivors, so I don’t have a fears in me, I don’t know for other people”.

M: Is there anything specific about Ebola that you think people to understand better?

R: “I do not have any doubtful issues because am well trained and qualified, I have trained for the past nine months under different organizations besides contact tracing, and I have so much experience”.

M: So you mean everything is cleared to you?

R: “Yap”.

M: I thank you very much.
