# Supplementary material for: Development of a set of community-informed Ebola messages for Sierra Leone
Source: PLoS Negl Trop Dis. 2017 Aug 7;11(8):e0005742. doi: 10.1371/journal.pntd.0005742 (PMC5560759; doi:10.1371/journal.pntd.0005742)
Supplement: S1 Appendix — (ZIP) [file pntd.0005742.s001.zip › Ebola messages - FGD and interview transcripts/R2HC Ebola Fieldwork 1/R2HC Ebola F1 HW-Urban5 V2 CORR.docx]

| CODE | **R2HC Ebola F1 HW-Urban5 V2 CORR(urban semi-structured interview with health worker/volunteer)**  **V2 – 11^th^ March 2015 – correction personal data** |
| --- | --- |
| DATE | February 2015 |
| DURATION (minutes) | 23 |
| Collector nr | 5 |
| LANGUAGE INTERVIEW | Krio |

**PERSONAL DATA RESPONDENT**

| Age *(in whole years)* | 42 |
| --- | --- |
| Sex (Female = F, Male = M) - circle | F |
| Religion | Christian |
| How much time does it take you to walk from your house to the nearest PHU? (minutes) | 5 |
| Mother tongue: | Temne |
| Role in the health facility / health: | XXXXXXXXX |
| Education level (circle) | Tertiary |
| Do you know anybody who had Ebola? | Yes |
| If Yes, what is your relation to that person? | Collaegue |

**TRANSCRIPT: (M= Moderator, R= Respondent)**

*(cell phone noise)*

M: When did you first hear about Ebola? How was the disease described to you, and what were your first thoughts about it?

R:” I first heard of it in April 2014(*Cell phone and door noise*) when the outbreak was in Guinea, but I have heard of it before that, so when they said Ebola has broken out, I thought of the Haemorrhagic fever (*note: Lassa fever is every year in Sierra Leone, Ebola and Lassa Fever are both viral haemorrhagic fevers*) that we have here, I knew about it before that.”

M: So when you told of it, how was it described to you?

R:” Well they said it is a deadly disease; the survival rate is ten percent that is what I knew”.

M: So when it was described to, what were you thoughts?

R: “Well I just said it should not reach us through the power of God, but I was afraid, I was really afraid, when they say Ebola I have read about it, I know about that so I was scared, very, very scared.”

M: In what ways has Ebola affected your community?

R: “In so many ways, because it was a hot spot, had about ten deaths in two weeks, even my children I had to lock them inside, it was yahgba (= a worry) and it was not easy.”

M: Have personally seen or known people who have had Ebola?

R: “I see Ebola person but from afar, I did not go near, the person that died is close person to me, my close friend, and we were in class (together)”

M: Why do think Ebola has spread throughout Sierra Leone?

R:” It’s our attitude”

M: Huh?

R: Yes it’s our attitude, the attitude of us Sierra Leoneans that is, it has spread because we don’t listen, we don’t abide to rules, we don’t take the law that is the very thing that led to the spread of Ebola”

M: What do you think is the best way to prevent Ebola from spreading?

R: “To involve the community let them take responsibility, let them take responsibility for their own health; the thing is that, if clips were shown of Ebola victims in the communities during the sensitisation, it is not just to talk about it on the radio, but if you see it, you can be able to learn better, we hear it over radio and in fact it is not everybody that listen to the radio, even those that go into the communities to deliver these messages, they always tell them that it is because they glad, but if you bring something like a video clip to show a real picture, this is what is happening , involve them, saying after seeing this film what do you think should be done to control this in our community, get their views, and then appoint people from the community to give the messages, don’t get other people outside the community to give the messages say to take a person from (- - one end of the interview district - -) to (- - another end of the interview district - -) to give messages , it will not work”.

M: What do you think is the best way to treat somebody with Ebola?

R: “ For me, the best way is to find a place in the community, and train people in that community; let them don’t go far away, the reason why this thing prolonged was, because there were no treatment centres in the infected communities, they took people all the way to Kailahun (=first district in Sierra Leone with Ebola cases) , if the infected person had vomiting and frequent stool by the time they get to Kailahun the infected person will be in a critical condition, although it has improved, but they need to get more care centres within the communities, and take community health nurses, that are within that community to work in the centres, they should not come from afar.”

M: So do you have any local term in Temne that you use to describe Ebola?

R: “Hah, no I don’t know that because my own Temne, (*respondent laughs*)”

M: Some people do not believe Ebola exists.

R: “Yes”

M: Do you know people in your community who think this? Do you know why they have this view?

R: “No, no, in my own community no, for now there is nobody in my community who does not believe Ebola exists.”

M: So, some of those Ebola messages you have heard and seen, the posters, can you give me an example of them?

R: “I saw one the day before yesterday which says “nar me go don Ebola, Ebola go tap wit me” (= I will end Ebola, Ebola ends with me), so, and those are the few I’ve seen around, there is another which says “for wase you han plenty time for tap Ebola” (=wash your hands always to stop Ebola, another one says “if you get fever call 117” (= if you have fever call 117).

M: Which one of those messages you feel is then best message, which people have used and adhered to?

R: “It is the hand washing, that is what I see plenty people doing, when you go to the offices you will meet water, when you go the shops you will meet buckets, if you pass along the streets you will meet buckets to wash your hands.”

M: Which one do you think that people have to adhere to?

R: “The ABC - Avoid Body Contact because people still crowd together, that particular message is not working, avoid body contact, no, people go to the mosques to pray, people still gather in the churches plenty, as for the market I will just leave that.”

M: What do you think would be a good message to encourage people to bring patients to a treatment centre?

R: “It is not every sickness that is Ebola that is what you should tell the person, mama it is not every sickness that is Ebola, We have had sickness before, if you get sick now does not mean that it is Ebola, but the best way to help the person and yourself, because this sickness, it is not only person that can contract it, it mostly kills one, two, three, four people but if they take the person for early treatment, it will help you and the person, even if it is not Ebola the person can treated, but if it is another sickness, the more you keep that person the more the sickness will bring the person down by the time you take the person would have died, that is the best way to talk to people”.

M: In the event of Ebola infection, do you think that people would prefer to first go to a traditional healer?

R: “No”.

M: To the health facilities that have been in place, or the CCC’s- Community Care Centres where do you feel they will first go to?

R: “The person should not be aware of contracting Ebola, in fact they are afraid of Ebola, the best advice is to go the nearest health centre, and they will advise “okay mama the way we see you go nar the community care centre” (= to go to the community care centre), but you should rush to the community care centre, just as they have been saying Ebola suspect, they are all suspected cases, but if you put them all together, if there is an infected person among them, the others will contract the virus from the infected person, so the best place to go is the nearest health centre, they will advise on what you should do.”

M: Some people stay at home when they think they have Ebola. Why do you think this? What do you think could be done to encourage them to come to a treatment centre?

R: “I will say life is sweet, and you are now sick, if you know that you are Ebola positive, that does not mean that you will die, because you have Ebola does not mean to say you will die, if you go for early treatment you will have the chances to survive, but you will only increase that chances if you go for early treatment, if you have frequent stool and do not know what to do to rehydrate yourself until you are totally dehydrated even if you go to the hospital they might not be able to help you, so go to the hospital quickly so that you can be cured.”

M: What do you think would be the best channel to get your new messages to people (i.e. through the radio, by text message, newspapers etc)? Which one is the best way to let the messages reach the people?

R:” Make (eh eh) community awareness campaign, let the human beings go to the communities, it is not everyone who owns a radio it is not everybody who listen to radio, some people do not take the radio messages seriously, let the human beings go physically to the communities to pass on the messages, and the best of the best is to show video clips, of, at least live stories of what people have gone through, survivors, use survivors more , if you use survivors right now, I think that is the best channel , they say who feels it knows it best, they will be the best to explain to people what happened to them.”

M: Have you ever heard people talking, either in a positive or a negative way, about the Ambulance service?

R:” The good thing is that, now when you call they don’t delay as the use to, they respond timely, the bad thing, is that they run these vehicles as public transports, some do not help the patients even when they are their PPEs, they allow the relatives of the patients to touch them which is very bad, you the health worker who is well protected should be the person to help the sick, these are the two bad things, to put uninfected passengers into an ambulance that is not good.”

M: So what about the holding centres, treatment centres, and CCCs, what are the positive or negative things that you have heard about them?

R: “Yuh, I will not say I have heard bad things about the treatment centres yet, because I know two, three, persons who have discharged from these centres, even one nurse discharged last week I did not hear her say anything bad about the treatment centre.”

M: Okay what are the good things she said?

R: “The good thing she said is about the social side, they talk to them well, they encourage them, they tell not to be discouraged, give them their medicines , at least the symptomatic ones, for them to get well that is all.”

M: What about the Ebola burial team what are the positive or negative things you hear about them?

R: “First the negative thing, I am not going to the positive, is that they don’t dig the graves deep, they handle the corpses very bad, they say they through the corpses, I have not seen it, but that is what I heard, they say they just through them anyhow, the other thing is that when they go to spray, they spray too much, even some people that is why they refuse to call, they say they spray too much of chlorine , it do affect them, these are the three things that I have heard, the good thing is that they protect themselves well, I have not heard the death of any member of our own burial team, that means they protect themselves well, and if they protect themselves well, that means they are also protecting their families and their communities that is one good thing.”

M: But they said when the burial team goes to bury people somehow?

R: “Pay money, yes I have heard that, in fact my own father-in-law complaint to me saying they asked for five hundred thousand (Leones, about 114 USD), one burial team, they had one case up (- - location in the interview district - -), so I told him to Channel the complaint to the supervisor, because I know the supervisor, I told them not to pay, so they ended up not pay, but they asked.”

M: At times when they pay that sum, the team allow the people to bury the corpse?

R: “No the one what I heard of is for the burial team to bury the corpse”.

M: What about the Ebola phone lines, the 117, have you heard anything positive or negative about them?

R: 117, they were not responding quickly, I don’t know if it was the bureaucracy, because when you call the 117, they channel it to surveillance, they contact their field worker, by the time the phone have been made, the people most have grown impatient, that is the complaint people make that they don’t respond quick, but I think it is the bureaucracy that is why 117 do not respond quickly.”

M: What about the existing health facilities and staff, are there any negative or positive things about them?

R: “The good thing is that we are still working, even with all the pressure, we are still working, but the bad thing is that we are afraid of everybody, everybody is afraid we don’t take blood pressure again, you don’t know the status of the patients that come to the hospital, and if you don’t check the pressure of pregnant women, they might become hypertensive during pregnancy, if you leave them without the checks they might convulse at home, the next thing is that even during the delivery, at first people delivery along the corridor, which was very bad, it was after the house to house campaign , that it stopped and when they opened the treatment centre for pregnant Ebola patients, that was when it reduced, but still we are afraid because many nurses got infected during deliveries, so we don’t go too close to patients, not that we bad, but it is personal protection ,you have a distance that you should keep and nursing is about caring, that is it”.

M: In your community, do you have any Ebola survivor or have you come across any survivor?

R: “Yes”

M: How the community reacted to them?

R: “We have them in our community, down at first when they came; people did not go close to them, I am not far away from them, so I went to visit them, when they saw that, they commented, so I told them that they are the safest person here, because they cannot contract it nor infect people again and that they are the people that are at risk, so they started talking to them.”

M: Which message can you give to communities that stigmatize Ebola Survivors?

R:” What I told mine, you should be more afraid of those who are not survivors, because they have the possibility of infecting people, than the survivors, in fact they have been to heaven, they are angels, they don’t infect people, we are those that are still in hell, in fact we should celebrate them, that is what I told my community, that is what we should tell our communities, they saved, if it is to go to heaven they save already, they are in heaven, they are safe than anybody, than those you know”.

M: Have you heard any treatment for Ebola?

R: “No, apart from the drug they brought for Doctor Willoughby, which was used on mammy Colombia (= both names of well known health workers in Sierra Leone), but I have not heard of any treatment as yet, apart from the vaccine that they to bring, which is prophylactics, but I have not heard of any other treatment”.

M: Have you heard of any new ways of preventing Ebola?

R: “No it is just the same message.”

M: About any vaccine for Ebola?

R: “Yes that is what I am saying, they said they want to do the trial here, but I don’t know how soon”.

M: From your perspective as a health worker, how would you describe the general knowledge about Ebola among people in your community?

R: “Now there is the knowledge, in fact I can raise it to ninety-eight percent in my own community or even to the smallest child have knowledge about Ebola, they know how to prevent it, the only problem is the practice, which is the problem, hearing, but practice is the , everybody is aware but practice is the problem.”

M: What do think that you need to know to enable respond to the misconceptions properly that people have in your community?

R: “Ah, what should I know, eh, eh, what again I don’t know”.

M: Is there anything specific about Ebola that you need to understand more, and how will you put it to people?

R: “Yes, what I want to understand is how long the virus stays alive on an object, like book, paper, pen, or clothes, how long it survives outside the human body, when it cannot infect people again, I have not got the answer to this question yet.”

M: What do you think would be a good way to explain this to people?

R: “The best way is to tell them, okay the radio will help but, word of mouth is the best, let people see you physically, and give them the example and in their local languages, if it is Temne areas let them talk in Temne, if it is in Mende areas let them talk in Mende, it is not everybody that understand certain Krio terms I think that is the best way to channel our messages”.

M: “Okay, Sister thank you very much.
