# Supplementary material for: Development of a set of community-informed Ebola messages for Sierra Leone
Source: PLoS Negl Trop Dis. 2017 Aug 7;11(8):e0005742. doi: 10.1371/journal.pntd.0005742 (PMC5560759; doi:10.1371/journal.pntd.0005742)
Supplement: S1 Appendix — (ZIP) [file pntd.0005742.s001.zip › Ebola messages - FGD and interview transcripts/R2HC Ebola Fieldwork 2/R2HC Ebola F2 FGD-Female-Urban1-C.docx]

| CODE | **R2HC Ebola F2 FGD-Female-Urban1-C ((**Urban focus group discussion with younger (<25 years) and older (25+) females, using **topic guide GroupC and Picture set C)** |
| --- | --- |
| DATE | March 2015 |
| DURATION (minutes) | 63 |
| Collector nrs | 1 and 4 |
| LANGUAGE INTERVIEW | Krio |

**PERSONAL DATA PARTICIPANTS**

| Nr | Sex  (*F/ M*) | Age  (*in years*) | Education Level (*e.g. none, Primary, secondary, tertiary*) | Language (*e.g. Mende, Temne, Krio)* | Religion | Job / Employment (*how they earn their living e.g. farmer, teacher, trader*) | Role in community  (*e.g. youth leader*)  ANONYMIZED, ONLY AREA OF ROLE INDICATED |
| --- | --- | --- | --- | --- | --- | --- | --- |
| 1 | F | 57 | Tertiary | Mende | Christian | Civil servant | None |
| 2 | F | 43 | Primary | Loko | Christian | Trader | None |
| 3 | F | 55 | Primary | Mende | Muslim | Trader | None |
| 4 | F | 29 | None | Mende | Christian | Trader | None |
| 5 | F | 18 | Secondary | Krio | Christian | Student | None |
| 6 | F | 21 | Tertiary | Mende | Christian | Trader | None |
| 7 | F | 22 | Secondary | Krio | Christian | Trader | None |
| 8 | F | 25 | Tertiary | Temne | Muslim | Trader | None |

**TRANSCRIPT: (M = Moderator, R= respondent, R1= first person responding to a question, DOES NOT correspond to numbering used in Personal Data!)**

**(NOTE: Topic 12 – Early treatment - Poster – “Na because ar go hospital quick, na dat mek ar well” (poster of survivor))**

M: Good morning my good people

Rs: Good morning.

M: We are here this morning to talk about message we developed from the interview we did last, due to your responses, now look at this poster clearly, it is without texts, and tell me what do you think about it?

R1: “The person on the poster resembles an Ebola victim”.

M: What do you mean by Ebola Victim?

R2: The person was affected by Ebola and the person has survived”.

M: Yes my sister, what is your thinking about this poster?

R3: “The person that was having Ebola, but went for early treatment and survived”.

M: Yes Mama?

R4: “It is a certificate that has been given to a person who was having Ebola and the person has been cured”.

M: Ok, what is this message about?

R5: “The person on that poster was affected by Ebola and he has been cured, that is what this picture is about”.

M: Ok, yes my sister?

R6: “It is a certificate of survivor, it shows that they have certify that the person he/she is free from Ebola”

M: Ok my sister?

R7: “It is a certificate of Survival from the Ebola sickness”.

M: thanks for your responses yes do you want to say anything?

R8: “The picture is telling us when you are infected with Ebola virus, go early to the hospital, you will be like that man in the picture”.

M: We are trying to develop a poster advising people to go for early treatment when they have Ebola, the message reads, “na because ar go hospital quick, na dat mek ar well”. What do you think of this message and what the message is about?

R1: “Well the message is good, because, it makes someone seek early treatment”.

M: Yes, my sister?

R2: “Yes, it is good to go to the hospital early, because if you don’t go early, your chance of survival is less, but if you go there early, you will get better treatment and you will survive”.

R3: “The message is fine”.

R4: “What I want to ask, people will go early for treatment but they will not survive, but really the message is fine”.

M: Yes ma, but do you have any concern or message about your doubt?

R4: “Well the only thing is, let the government work hard to end this Ebola sick, because this Ebola has killed us a lot, then I don’t know the medical people were carelessly handle the sick, but first, people were going to the hospital earlier, but they were still dying ”.

M: Ok, but is there any improvement now?

R4: “yes it is better now”.

M: What about you sister?

R5: “Yes, there is much improvement”.

M: Ok, is there anything about this message that you think people may not like?

R1: “Well, when this Ebola started, the people were not making any serious effort about it, but it is better now, really the message is fine and there is nothing in this message which people may not like, people may like this message”.

M: Ok, my sister?

R2: It is not correct, why, sick people were going to the hospital early but they were still dying, so in that context, the message is not correct”.

M: Ok, that was the reason, I asked if there is much improvement, you said, yes, so , is there anything wrong with this message, that look at the krio and the colour of the picture?

R3: The spelling in krio is not correct”.

M: What spelling is not correct?

R4: “The spelling of because, hospital and well”.

M: Ok, can you give us your own spellings?

R4: “The spelling of because is “becus”.

M: What about the hospital, how do you spell the hospital?

R4: “The krio spelling for hospital is “hospita”.

M: Is this message acceptable in your community?

R5: “No, they will not accept it”.

M: Why?

R5: “Because people are very disgruntled.

M: What are they disgruntled about?

R5: “About the whole Ebola thing”.

M: Like how, be specific?

R5: “Well are fed-up with this whole Ebola issue, we have prayed and fast for it to leave this country, but nothing like has happened, so people are really disgruntled”.

R6: we are tired of this Ebola , everyday people will come with different approaches, we are fed up , tomorrow they will tell you we have six confirmed cases, the other day you will be told one , the next day you will be told zero, we are tired whatever you people want to do you can do it , but we are tired , our voices are not heard , when you talk something different , you are threatened with the state of emergency , had it not been for the respect of (name of moderator) I will not say a word , but we have high regard for you .

R7: have you spoken with the health workers to ensure patients are treated with dignity, and whenever they have patients they should ensure they treat patients with care, love and respect, but this is not done, so if you are going to pass on this message you should ensure the health staff are ready for it, because if people are not treated well they will not take your message serious.

M: let us assume that the government addresses your concern do you think people will accept this message?

R1: well I believe people will accept

M: But if this message is posted at a certain place, will it change the perception of People?

R6: “No, they will not even look at the poster”.

M: Why?

R6: “As my sister said, they are really disgruntled about this Ebola”.

M: Ok, this message shows that early treatment benefits you, when you go early you might survive the Ebola sick, what do you think about this?

R1: “I don’t really see any benefit, because they have sang songs, talked on radio, but the Ebola doesn’t want to leave this country”.

M: Well we want to get your opinion about this message, this message is talking about early treatment, going to the hospital early will give you chance of survival, and we know before now people were going to the hospital early but they will still not survive as you said, you said also, there is much improvement now, so based on this improvement I want to know now, if the message is clear?

R2: “Well the message is clear, but you will only survive if it is malaria you are having”.

R3: “As my sister said, if it is other sicknesses, you will surely survive, sickness like malaria, muscle pain”.

M: what about if it is Ebola sickness?

R2: Believe you me unless you have high luck , let me tell you something , the various treatment centre have different methods they use to treat patients, that you cannot deny , what about the doctors , do you want to say they didn’t go to the hospital quick that is why they died of ebola , (name of moderator) you have to understand that those doctors went to the treatment centre early and they didn’t survive, so we are confused , these are all the issues that made people not to go to the hospital , because 90 percent of the health workers that have the sickness died even though they went for early treatment, so if you want to pass on this message you have to take some of these points

M: Ok, with this message, “na because ar hospital quick, na en mek ar well”, is this message clear?

R4: “Yes, it is clear, if it is malaria, but it is not clear to Ebola?

M: Why it is not clear to Ebola?

R4: “Because if they have test someone positive, they will not allow the person will not come back home, and they will not even treat the person, which will lead to death, if it is malaria, they will treat the person and return back home”.

M: is there anything about this message that you think people may not like?

R5: “Some people will like it and some may not.

M: Why some may not like the message?

R5: “Because people will still have it in mind that when the outbreak started people were going early to hospital, but they were not still surviving, so with this thinking, it does not make no sense to the people”.

R6: “Really for me, some people may accept it, and there is nothing wrong with the message”.

M: Why some people may accept, this message?

R7: “Because really, it has helped people, when they go early, their chances of survival is great. It has save some of this people, so they will accept it.

M: Ok, yes me sister?

R8: “Some of this people may like, because it gives hope. ”

M: Ok, do you think people in this community and the other communities will change their belief after hearing this message?

R1: “Well, our community may accept it and it will change the beliefs of some people not all, because even if something is 100% good, not everybody will accept it”.

M: ok?

R2: “Yes, the beliefs of people will change in this community, but I don’t know for the other communities”

M: Ok, we are not saying you will know every about other communities, but you must have friends that you interact with, so by asking them you will know about their community, so do you think this message will change their beliefs?

R2: “Many people don’t have time to talk to you about Ebola this days, you guys are even lucky, we are wasted our time talking to you about Ebola, so I don’t know about other communities”.

M: But by observing some people do you think this message will change their beliefs?

R3: “Well I don’t know for other people, because everybody has different perceptions, so you can predict for the person”.

R4: “In this case of some people who has lost their parents or love ones, they will not accept this message, because their love ones went early to the hospital but did not survive, so they will not accept this message”.

M: But what about in a case, when this person went early and survived, how will this person see this message?

R5: “They will accept the message, because they have proved it beyond all reasonable doubt”.

M: What about their parents?

R6: “They will accept this message, and even talk to other people about this message”.

M: Who do you think will distribute this message?

R1: “The survivors, because he or she knows the importance of going to the hospital early and the person have gone to the treatment centre and have come back”.

M: Ok, this poster that I have shown to you just now, where do you suggest they should be posted?

R1: “Markets place like (--name of a shopping street--) streets”.

M: Ok?

R2: “they should be posted at police station, schools, because at these places, people are always gathered”.

R3: “At round about, busy streets, radio stations”.

M: ok, apart from the survivors, who do you think will be distributing these message?

R4: “I suggest the health workers”.

M: why?

R4: “Because they are dealing with health issues”.

M: Ok any other people to distribute this message?

R5: “The volunteers”.

M: Why the volunteers?

R5: “They have the courage and patience to do the work”.

M: Ok, my sister?

R6: “I suggest the comedians, and music stars, they will be the right people to do this work”.

M: Why?

R6: “Because with their comic behaviour, they will attract many people, the people will have patience to watch and get the message”.

**(NOTE: Topic 20/21 - Early Treatment and Prevention – “ Noto pass you see before you believe se - Ebola de”)**

M: Ok, let us look at this other message, which reads, “noto pas yu see demy u believ say Ebola dae” what do you think of this message?

R1: “the message is not bad”.

M: Why?

R1: “Because the momentum Ebola has taken if this country, you can’t say unless you see, before you get the belief that Ebola is real, so that makes this message more unique”.

M: Yes mama, what is your take?

R2: “Ebola is real, this no joke, so you don’t have to say unless I see Ebola patient or you are sick of Ebola, before you conclude that Ebola is real, the message is good and straight forward”.

M: Yes ma, how do you see this message?

R3: “It is no joke, Ebola is real, do not say, seeing is believing, because if you make that as a rule, definitely you may not be far from Ebola, the message is fine, I think people may like the message, our Ebola is violent, it is quite different, because it has kill a lot of people”.

M: Ok, yes my sister, what is your contribution towards this message?

R4:”The message is saying true, you can’t say unless you see then you believe, because the Ebola has killed a huge amount of people in this country, so we don’t need to joke with it, it is real and it kills”.

M: Yes my sister?

R5: “So people are still stubborn, so people unless the Ebola affects them or their relatives before they believe the reality of Ebola, they will show this message but they will not believe that Ebola is real but really the messages are good”.

M: But this message is acceptable in this community and other communities?

R6: “Yes, it is acceptable”.

M: Why it is acceptable?

R6: “because Ebola is real, and you have to believe, you cannot say seeing is believing”.

M: Is there anything in this message that you think, people may not like?

R1: “Well there is nothing wrong with the message, the message is ok, Ebola is real and it kills”.

M: Yes my sister?

R2: The message is good, because, you can’t say unless you see before you believe the reality of Ebola”.

M: Ok, my sister?

R3: “Everything about the message is fine, because you can say, unless, you see, before you believe”.

M: Ok, my sister, is there anything that is not good about this message?

R4: “No”.

M: What is no?

R4: “Because the message is good”.

M: Ok, so what about the krio and the colour of this poster or massage?

R1: “The message is krio and English, it is not a proper spelling, “the before spelling in krio is no correct”.

M: Ok, so what about the colour?

R2: “The colour is nice, it is in black and white, and they gave it the right colour”.

R3: “The colour is good, but the font size needs to be increased”.

M: Do you think people will accept this message in this community?

R1: “Yes, they will accept the message”.

M: Why?

R1: “Because Ebola is real”.

M: Yes my sister?

R2: “some will accept the message and others will not accept the message”.

M: Why?

R2: “Because not everyone them have been affected by Ebola”.

M: Yes mama?

R3: “Some may accept and some may not accept”.

M: Why?

R3: “Because not everyone believe in the reality of Ebola, because they have not be affected, even when they said avoid body contact, people were still gathering at pubs to drink”.

M: Ok, yes my sister?

R4: “some of the people may believe and some may not”.

M: “Why?

R4: “Some may believe because they have been affected, and the others because they have not affected, they will not believe”.

M: Yes mama?

R5: “People may accept, because Ebola is real”.

M: Ok, this message is telling us that, some people that have the thought that unless they see, before they believe, should stop thinking that way, because Ebola is real, so it is not a rule that unless you see before you believe, do you think this message is clear?

R1: “It is clear, because you will not sit back and allow the sickness to come to you”.

M: Yes ma?

R2: “The message clear and understanding, because you will see the reality in the message”.

M: “Yes mama?

R3: “It is clear”.

M: Who do you think will distribute this message?

R4: “The survivors, because he/she have been affected by the Ebola sick”.

M: Yes mama?

R5: “The survivors”.

M: Yes my sister?

R6: “The survivors”

M: Why?

R7: “Because they have already been affected and they have survived”

M: Yes Aunty?

R8: “The Youths and community leader”.

M: ok, yes mama?

R1: “Religious leaders, musicians”

M: Like which musician?

R1: “Big Joe”.

M: Ok?

R2: “Yes, my sister, let me take you back, according to the message, it means, you are forcing the people, I want to suggest the message go like this “you have to believe Ebola is real”.

M: Ok, so this is the way you want this message to be?

R2: “Yes, because the message that you have just read depicts that you are forcing the people to believe”

M: Ok, what do you think of the following messengers, like the Ebola survivors?

R1: “They are the right people to be messengers of this message”

M: Yes my sister

R2: “They are the actual people to carry out the message”

M: Ok?

R3: “The survivors”

M: Ok, What about the health workers, are they good messengers to this message?

R4: “Yes, they are good messengers, because they are working within the health care system”.

M: Yes mama?

R5: “Yes, they are good messengers of this message”

M: Ok?

R6: “yes, the health workers should carry out this message”.

M: What about the traditional healers?

R7: “No, they are not good messengers, because they do not know anything about Ebola”.

M: Ok?

R8: “They should not be used as messengers, because they don’t know and since this outbreak, they stopped them from treating people, so if the traditional healer comes and tells me about Ebola, I will say, leave me alone, because you do not know anything about Ebola”.

M: Yes, my sister, are they good messengers?

R1: “No”.

M: Ok let us talk about the channel to this message, is drama a good channel to this message?

R1: “Yes”.

M: Why?

R1: “because everyone will see it clearly and imagine it as real life situations”

M: Yes my sister?

R2: “It helps spread the message, because people may understand the message very well, as it is dramatized”

M: Ok?

R3: “To dramatize this message is the best, when it is dramatized publicly to the people, they will get the understanding that Ebola is real”.

M: Ok?

R4: It is good channel of this messages, but when doing this, used comedians like Sara the great and other comedians, to dramatize this message at public places like (--two names of busy road crossing in the interview district--) and also use the advertisement video screen at public places, play this particular acted drama for people to see”.

M: ok?

R5: “The same as my sister have said”.

M: Ok, what about the radio jingle?

R1: “It is not bad, but not everyone listen to radio, but the ones listen will know what have happen”.

M: “But do you think radio jingle is the best?

R2: “Yes”.

M: Why?

R2: “Because it is a radio and most of the people listen, though not everyone listened”.

M: “Ok, but my sister said not everybody have radio”.

R2: “Yes so people have phones with radio, so they will listen to the jingle and also watch it on television”.

M: Ok, my sister do you think radio jingle is the best channel of for this message?

R3: “Yes, because most of the people listen and understand”.

M: Yes my sister?

R4: “it is fine, but not everyone listens to radio”.

R5: “It is fine, because we have a lot of radio stations now in our community”.

M: Ok?

R6: “It is good”.

R7: “Radio jingles are fine, but not everyone listens to radio”

M: Ok?

R8: “The radio will not spread much compared to the drama”.

M: What about the face to face?

R1: “The face to face is not workable”.

M: Why?

R1 “Because if I come to someone and I met that person is stress, he or she will not listen to my message, but rather say is because you are eating Ebola money that is why you telling me about Ebola, it may not work”.

M: Yes, mama, what do you think of the face to face?

R2: “It is good”

M: Why?

R2: “Because you met us idling today, that is why, you are talking to us, because if were busy, you should not have talked to us?”

M: Ok, what about wrist bands?

R3: “It is not fine, not everyone will see the writing, and some people do not like it”

M: What about the badge?

R4: “Well not everyone will like the badge”.

M: Ok?

R5: But is the badge free of cost?

M: Yes, so if they print message on this badge and give people, will they accept it?

R5: “Yes, if only it is a free of cost, I don’t have problem with that”.

R6: “If even it is free, people may not like it”

M: Ok?

R7: The badge is good, because people may concern and I want to know the message on the badge that you are hanging on you”.

M: What about poster?

R1: “Well, posters are good, because it will attract passer-by”.

M: Ok?

R2: “Posters are good”.

M: Yes my sister?

R3: “Well some people will read and some may not, the educated ones will read and the literate, do not have time to read”.

M: Ok?

R4: People will read, because some posters are good looking, the colour may attract their attention”.

**(NOTE: Topic 24 – Leaflet - “6 steps while you wait for the ambulance for burial”)**

M: OK, let talk about the six steps while waiting for the ambulance, when someone is sick, so look at it carefully and tell me what you think about this message?

R1: “The steps are fine, because it is a law that we should not touch sick people”.

M: OK?

R2: “The message is good, because as the Ebola virus is violent, you should not touch someone that is sick, you should strictly follow the steps”.

R3: “Well, when this Ebola outbreak started, the 117 phone line was not working properly, so people where touching, washing and burying of corpse, but looking at these steps, it is good, because it is given directives to the people”.

M: Ok, yes my sister, how you see the steps, what is your own view?

R4: “Well, the message is fine and they will accept it”.

M: Why they will affect the message?

R4: “Well, a lot of us how conscious and aware that the sickness is real, every day they talked about the sick, so with this, they will accept the message”.

M: Ok, is there anything about this message people may not like?

R1: “People will like the message”.

M: What about the Krio and colour the message is been printed in?

R2: “Well, there is nothing wrong with this message, the krio and colour is perfect”

M: What will the best way to give this message out?

R1: “We are just praying for this Ebola sick to End”

M: Ok, but how do you think, this message will reach the people?

R2: “Well, by radio jingle, posters, radio discussions”.

M: Who do you think will be messengers of this message?

R3: “The comedians, music stars”.

M: Ok, yes my sister?

R4: “health workers, survivors, teachers in school”.

R5: “Religious leaders, community stakeholders, youths”.

M: What about the traditional healers?

R6: “The traditional healers don’t have hands in the dissemination of this message”.

M: Ok?

R7: Traditional healers may not be good messengers of this message, because they are not happy about the message, thinking they will lose their customers”.

M: Ok, what about the pastors and imams?

R7: “They are good messengers, because they talk about Ebola in their sermons”

M: Ok, What do you of the community meetings as a channel?

R1: “It is not a good channel, because they said we should avoid public gathering and body contact”.

M: But this gathering is about Ebola?

R1: “But they told us that we should not gather publicly, so how can you get a community meeting with more than ten people, so it is not a proper channel”.

M: “So what about sermons in mosques and churches?

R2: “That is better”.

M: Why?

R3: “Because in the churches or mosque, people stand at distance and most of the people listen and adhere to what their religious leader tell them, it is better than community meeting”.

M: What about radio discussions?

R4: “It is fine”.

M: Why?

R4: “because a lot of people listen to radio”.

M: What about the house to house campaign?

R5: “It is good, because look in your own instance, you came to our houses toady, you met us and we are talking”.

M: OK?

R6: The house to house is not good”.

M: Why?

R6: “Because it is just the same gathering, now look, we are together talking and we are more than ten, it is very risky, because you will mistakenly took someone”.

M: What of this message is printed on plastic bags?

R7: “People may not like it, because they will not see the message properly”.

M: OK?

R8: “It is not good, because not everybody will read the message written on the plastic bags”.

M: What about the leaflets?

R1: “The leaflets are not bad, because if you take your time and give someone, they will read and understand the message”.

M: How will be the right people to distribute this message?

R2: “Health workers”.

M: Yes, my sister?

R3: It is just the same, the health workers”.

**(NOTE: Topic 33 - 117 prank calls –“Lek Mama Salone – no mek kalo kalo call to 117, e de hambug the Ebola response”)**

M: Ok, let talk about the fake calls people make to the 117, line, the message reads”Lek mama salone- nor mek kalo kalo call to 117, e dae hamburg di Ebola response” what do you think of this message?

R1: “For me, I don’t know if people are wicked enough to do fake calls to 117, but all I have to say, you should don’t make fake calls”.

R2: “For me the message is ok, because if people continue doing fake calls to 117, it will disturb their response to a right call, they may think it is a fake call”.

M: Yes my sister?

R3: “The message is fine, because if you not developed message like this, the fake calls will continue, so the message is fine”.

M: Yes sister?

R4: The message is fine, because it reduces the number of fake calls”.

M: Ok?

R5: “It will warrant them to stop the fake calls”.

M: Ok?

R6: “I don’t really know why people like putting fake in some certain issues, the message, the message is good, because, it reduces the number of fake calls”.

M: Ok, Is there anything about this message that people may not like?

R1: “No, they will like the message”.

M: People will accept this message?

R2: “Yes, because the people knew that the response of 117 was slow due to fake calls, so they will accept this message”.

M: So will this message change the beliefs and behaviour of the people in this community and the other communities?

R3: “Yes”.

M: How?

R3: “Because this message has reached them”.

M: Ok, is this message clear?

R3: “Yes”.

M: So who do you think distribute this message to the people?

R4: “Radio stations”.

M: Ok,

R5: “You as an individual will give out the message as you know fake calls are not good”.

M: What channels do you prefer in giving out this message?

R1: “Radio station”

R2: “Television, house to house”.

M: Ok?

R3: “The jingle, dreams”

R4: “I prefer the music stars”.

M: Why do you prefer these channels?

R5: “Because they will spread the message to the people”.

M: What about if you have a male and a female in your community?

R6: “It is fine, because their communities will listen to them”.

M: What about loudspeaker on cars?

R7: “Well for me, I prefer it to be the best, because when this Ebola outbreak started, they were using louder speaker on car”.

M: Ok, will the people accept it?

R7: “yes”.

M: What about text messages?

R1: Sending text messages is not fine, because not everyone have phone and some have but they are not able to operate their phones”.

M: OK?

R2: “I prefer the louder speakers to the text message, because with the louder speakers it can reach everyone but unless the phone, it can reach only few people”.

M: Ok?

R3: “It may not spread wide”.

M: Why?

R3: “Because not everyone has phone, so people has phone, but they can’t read and understand the message well, like for the vehicle with loud speakers if even you are sleeping, you will hear”.

M: I thank you very much for your time.
