# Supplementary material for: Development of a set of community-informed Ebola messages for Sierra Leone
Source: PLoS Negl Trop Dis. 2017 Aug 7;11(8):e0005742. doi: 10.1371/journal.pntd.0005742 (PMC5560759; doi:10.1371/journal.pntd.0005742)
Supplement: S1 Appendix — (ZIP) [file pntd.0005742.s001.zip › Ebola messages - FGD and interview transcripts/R2HC Ebola Fieldwork 2/R2HC Ebola F2 FGD-Female-Urban2-A.docx]

| CODE | **R2HC Ebola F2 FGD- Female-Urban2-A (**Urban focus group discussion with younger (<25 years) and older (25+) females, using **topic guide GroupA and Picture set A)** |
| --- | --- |
| DATE | March 2015 |
| DURATION (minutes) | 55 |
| Collector nrs | 1 and 4 |
| LANGUAGE INTERVIEW | Krio |

**PERSONAL DATA PARTICIPANTS**

| Nr | Sex  (*F/ M*) | Age  (*in years*) | Education Level (*e.g. none, Primary, secondary, tertiary*) | Language (*e.g. Mende, Temne, Krio)* | Religion | Job / Employment (*how they earn their living e.g. farmer, teacher, trader*) | Role in community  (*e.g. youth leader*)  ANONYMIZED, ONLY AREA OF ROLE INDICATED |
| --- | --- | --- | --- | --- | --- | --- | --- |
| 1 | F | 46 | Secondary | Mende | Muslim | trader | Health |
| 2 | F | 18 | Secondary | Mende | Muslim | Student | Health |
| 3 | F | 26 | Tertiary | Temne | Christian | Student | none |
| 4 | F | 30 | Primary | Temne | Christian | House wife | none |
| 5 | F | 34 | None | Limba | Muslim | none | none |
| 6 | F | 40 | Tertiary | Temne | Muslim | teacher | Youth |
| 7 | F | 20 | Secondary | Temne | Muslim | teacher | none |
| 8 | F | 19 | Secondary | Temne | Muslim | trader | none |

**TRANSCRIPT: (M = Moderator, R= respondent, R1= first person responding to a question, DOES NOT correspond to numbering used in Personal Data!)**

**(NOTE: Topic 7 - Burial - Posters – ”Wi respect dae dae bodi” en “Leh wi join an en gi wi pipul dem gud berrin”)**

M: Good Morning my people, I thank you very much for giving me your consent.

Rs: “Yes, good morning and Welcome”.

M: Look at this poster critically and tell me what you think of this poster?

R1: “By looking at the poster, it is telling the people about the people and the burial team, it shows that the burial teams are doing safe burial now, and they are telling the people not to be afraid”.

M: Ok, yes ma, what do you think about this poster?

R2: “The picture is telling us not to be afraid and let us allow the burial team to bury our relatives that have died, let us don’t bury for ourselves, as people where hearing by rumour that they used mass grave to burial people, but it is not true so let us have a free of mind. Burial process are safe and respectful and they will allow to pray the dead”.

M: Ok, we are trying to develop a poster on burial, how it is carry out, particularly the Ebola burial, now look at this poster carefully, it is having a text printed on it, and the text reads “wi dae respect daedae bodi”, so look at this poster and the message, and tell me what do you think about the photo?

R1: “The message is telling us that, now they respect dead bodies and they used coffin to burial, and they allow the family members to be part of the burial process”.

M: Yes my sister?

R2: “Well the way I have seen this message, now they allow the family members to be part of the burial process, and they respect the dead body, pray for the dead, either Muslim or Christian, they will put you in coffin or burial and you will bury with respect”.

M: Ok, yes my sister?

R3: “Well now they respect the dead, give the dead a befitting burial”.

M: Ok?

R4: “The burial team now, respect the dead body”.

R5: “A befitting burial is carryout, and prayers are offered in both Muslim and Christina way”.

M: Ok, is there anything about this poster that people may not like?

R6: “People will like this message, because now they are using coffin and offer prayers, so people will surely like the message”.

M: yes ma?

R7: “People may like the message, because now they are well satisfy, the message is ok, prayer was the doubt and problem of the people, since prayers are offered now according to the poster, well their satisfaction is guarantied.”

M: Ok, let us look at the krio and the colour of the message, is there anything wrong with the krio writing?

R8: “Well no, nothing is wrong with the krio, it is well written, even the word respect, gives the message an attention”.

M: What about the colour?

R1: “People love the white colour, because it signifies peace”.

M: What about the picture?

R2: “The picture is also good, people may like the picture”.

M: Ok?

R3: “The colour and printed krio is good, people may like it, the krio is written correctly. The word respect makes the message to be unique, as Sierra Leonean when someone tell them about respect, they appreciate you so much, the krio is fine”.

M: Yes my mum?

R4: “Yes, may like the poster, but the process may not be like before, washing of the dead, and dressing of the dead, but the perceptive people were moving along with was very wrong, that they will just throw the dead body and leave it like that, so they want to change this perspective totally that they respect dead bodies”.

M: ok, will people accept this message?

R4: “Yes”.

M: Why?

R4: “They will accept it because the word respect in the message gives the people, a double assurance and change the mind of the people”.

M: Ok, yes sir?

R5: “Yes people may accept the message, because they pray for the dead and the dead corpse is buried with a coffin”.

M: Ok, yes ma?

R6: “Well yes, they will accept the message, as long as they offered prayers for the dead body”.

M: Ok, well we have developed this message to tell people about safe and dignified burial and to discourage unsafe burial. Let us look at the message carefully, is the message clear?

R7: “Yes, it is clear”.

R8: “The message is clear and understandable”.

M: OK, is there anything on this message that people may not like?

R1: “People will like this message”.

M: The people of this community and other communities change their beliefs after hearing this message?

R2: “Yes they will accept this message and then change their belief after seeing this message, now the people will allow the burial teams to bury, like before people were hiding dead bodies and bury for themselves, this was because the rumour that people were buried with plastic bags, and are thrown away, but upon seeing and reading this message they will accept”.

M: Yes ma, will this message change the belief?

R3: “yes”.

M: Why?

R4: Because everybody is conscious and aware of the no touch policy, we should not touch each other and even dead bodies, if we do, it will cause more infections, so with this little message, people will change their belief and will be satisfied with this picture”

M: Ok my sister?

R5 “People in this community and other communities will accept this message, as people were anticipating for a respectable and dignified burial, so by seeing this message, their beliefs will be totally from doing secret burial”.

R6: “Yes their belief will change, because the picture has given them confidence that now, they offer prayers and coffin are used to burial dead corpse”.

M: Ok?

R7: “They will accept and change their beliefs”.

M: Ok, Which best way you suggest to give out this message?

R1: “The best way, these posters should be posted in each community, so when they see this poster, they will that safe, respectable and dignified burial is done”.

R2: “I suggest this poster should be bigger than this, and people will be go house to house sensitizing people, because not everyone has the time to read posters in the street”.

R3: “As my sister was saying, they should go house to house, and this movement should be backup by a megaphone, because most of the people do not have time to read message on poster that is posted”.

M: Who should give out this message?

R4: “The social mobilizers, and the community people, since they are in community, people may accept them and the message”.

M: Yes ma?

R5: “Like my sister just said, they should mobilizers and community health workers, and involve more of the community people, as people may love to see, members of that particular involved in the dissemination of that message”.

M: Ok, yes my sister?

R6: “Let them use the youths to disseminate, because they are more active”.

M: What channel do we use in giving out this message?

R7: “I suggest community sensitization, holding the posters in our hands, moving house to house with mega phones, explaining to the people about the message”.

R8: “Like what my sister said, let us embarked on the community sensitization, this message will reach the people.

M: “Yes ma?

R1: “We also use the media, like radio, televisions, though not everybody have that time to listen or watch, but I believe majority of the people may get this message”.

M: What do you think of the burial teams as messengers or channel of this message?

R1: “Well so people will not accept and believe the burial teams as messengers, because people have not seen this poster or message before”.

M: Yes ma?

R2: “People may to accept them as messengers, because they will be afraid of them, some people do not even like the burial team due to rumour of unrespectable burials”.

M: What about a member of family as messengers?

R3: “Yes, they will be a good messengers, if the family member have witness a respectable bury, so he or she will build the confidence of the people”.

M: Yes ma?

R4: “They may not be good messengers, because some people will become jealous of them in community that they did the selections by favour”.

M: Ok?

R5: “A family members will be preferable”.

M: Why?

R5: “Because they may not give out wrong information, since they may like to protect themselves and their community”.

M: What about the pastors and Imams will they be good messengers of this message?

R6: “Yes, if the pastor have witness this type of burial process, and because they are man of God in their community they people may believe and adhere to the message”.

M: Yes ma?

R7: “Well they will be good messengers, but mind you, not all the people are fervent worshipers, or go to church or mosque every day, and this people may not have time to go house to house, this message is for everyone, so they are not good messengers”.

R8: “Well the pastors and imams are good messengers, but they need to be backup by the community members and the community youths, the pastors and imams are good messengers, because some people believe them so much”.

M: What about the posters, are they good channels?

R1: “Well, as my colleague just said, so people may not have time to read posters that is posted, but if you go house to house with mega phones in the hands, it will be fine”

M: Where do you suggest this poster to be posted?

R1: “The posters should be posted in the communities, streets and public places”.

M: Yes ma?

R2: “The posters should be posted at community centres and pharmacies”.

M: Ok, ma what is your suggestion?

R3: “The posters should be posted at mosques and churches, so the worshippers will see”.

R4: “This poster should be posted at the ataya base and cookery shops”.

M: Ok?

R5: “This posters should be posted at the parks or bus stops, and should be also posted in public transport”.

**(NOTE: Topic 16 – Misconception about health system)**

M: Ok, we are trying to develop a photo album, for the ambulance picking up patients to the hospital, so look at the pictures carefully and give your view, what do you think of this album?

R1: “Well it is a good idea, seeing the ambulance pickup patients, and also the ambulance having only one patient with good ventilations, it is very good”.

R2: “The photo album is very necessary, when people sees it, they will get satisfy, because the rumour about under treatment in the ambulance will be over, because of the comfort in the ambulance, so with this photo their confidence will be build up”.

M: Yes my sister?

R3: “The photo album is good, but the photo album needs to be colourful and good looking”.

M: How do you prefer the message to be on the photo album?

R4: “They message will be, people should not be afraid to go with the ambulance as it is comfortable and the best means of transportation”.

M: Ok, this message is talking about the misconception about the medical system, so look at the album carefully and the steps, what do you think of the steps?

R5: “The steps are very necessary and good, because increase the confidence of the people as the misconception about the health system was in a abundance, so this will build their confidence, this even occur to my sister, she was sick, they took her to the treatment centre, she was there, we were communicating and she was given us updates, so really the photo album is good”.

M: Ok, is there anything about this message people may not like?

R1: “They will like everything about the message, because there are safe measures”.

M: Will the people accept the message?

R2: “Yes, they will accept it”.

R3: “They will accept the message”.

M: Ok?

R4: “The people will accept the message, because this type of care the people were looking for, talking to the patient nice and treating them with care”.

M: The community people will accept this message?

R5: “Yes”.

M: This photo album is addressing the misconception about the health system, and to build the confidence, is this message clear?

R6: “Yes it is clear”.

R7: “The message is clear, and the people will understand the message”.

M: The people will change their belief and behaviour after seeing or reading this message?

R1: “Yes it will change their belief and behaviour”.

R2: “The beliefs of the people will change and their behaviour will change”.

M: Ok?

R3: “Their belief will change, they will not touch the sick, bury the dead, and they will wait for the ambulance”.

M: What is the best way of giving out this message?

R4: “Through sensitization, by community members and youths”.

R5: “Through the community sensitization by youths and community mobilizers”.

M: Ok?

R6: “The youths have to use megaphone to pass on the message”.

M: What is the channel do we use?

R7: “Radio, television and megaphone”.

M: What do you think of the Ministry of health and sanitation as messengers?

R1: “Yes because they care are in charge of the health system, so they will be good messengers and people will believe”.

M: Yes ma?

R2: “Well, the ministry should pass on the message to the community members and the community members will pass on the message to their colleagues”.

M: Yes ma?

R3: “The community health workers”.

M: please give me your view on the following channels for this message like photo album, is it consider as the best channel?

R4: “Yes, the photo album will be one of the best messages”.

M: Who do we give this photo albums for distribution?

R5: “The youth leaders”.

M: Yes my sister?

R6: “We have to give the councillor in the councillor or the secretary to the councillor”.

R7: “The community youths and also religious leaders”.

M: Ok?

R8: “I suggest the community members and mobilizers”.

**(NOTE: Topic 25/26 – Stigma - “Kabo to una homes, una wokplace en una community, una wi broda en sista dem way don survive Ebola” en “Nor Laf or run from porsin way bin don get Ebola” (Do not laugh at or avoid Ebola survivors) en “Nor kongosa porsin way bin don get Ebola” (Don’t Spread gossip about Ebola survivors).)**

M: Ok, now we are coming to talk about the survivors, look at this message carefully which reads ”Nor kongosa poson wae don get Ebola” (Don’t spread gossip about Ebola Survivors). What do you think of this message?

R1: “Well the message saying that Ebola survivors are the hero and when they returned to the community, we have to embrace and encourage them, let us don’t stigmatize them”.

M: OK, yes mama?

R2: “The message is saying, we don’t have to stigmatize them, when the y returned back in the community, they are heroes, they need to be embraced, do everything in common”.

R3: “What do I tell of this message, we have to encourage the survivors, and we don’t need to point fingers on them, or back bite them”.

M: OK, yes my sister?

R4: “The message is saying, we should not gossip the Ebola survivors, it will be create stigma for them”.

M: OK, yes sister?

R5: “The message is telling us to encourage survivors, let us don’t spread gossip about them”.

M: Ok, but is there anything about this message that people may not like?

R6: “No, because the message is an advice, even there is a bye-law which says we should not stigmatized, if you do, it will be detrimental to them”.

M: Yes mama?

R7: “People will be happy for this message, because they have announced it all over that we should not stigmatized, we should gossip them, we should point fingers on them and the people are aware this issue, it is just an additional and they will accept the message”.

M: Ok, what about the krio language this message was printed in, is it correct?

R1: “Yes, it is correct, everybody will understand the krio, than the English, so it preferable and nothing is wrong with the message”.

M: What about the colour?

R2: “The colour is fine”.

M: OK?

R3: The black and white colour is ok, for this message”.

M: The people in this community will accept this message?

R4: “Yes, they will accept the message with open hand”.

M: Why?

R4: “Because these survivors were sick and survived and it is part of the bye law that we should gossip the survivors, we have to embrace them as our brothers and sisters, we should not gossip them, and so they will accept the message”.

M: Yes my sister, why do you agree that people will accept this message?

R5: “The people will accept this message, because they have the awareness of stigmatization, they should not stigmatize, and most of the people are wishing for the end of Ebola”.

M: OK?

R6: “They can accept this message, because the message is good and it will stop stigma in the community”.

M: Well, this message is addressing stigma, to fight against stigma, let the people don’t point fingers, gossip, or do things that is unreal to the survivors, do you think this message is clear?

R6: “Yes, it clear”.

M: Yes my sister?

R7: “This message is clear”.

M: Is there any aspect about this message people may not like?

R7: “No”.

R8: “No, the message is good”.

M: The people in this community will change their belief and behaviours towards the Ebola Survivors after hearing this message?

R1: “Yes, if they were stigmatizing them, it would not happen again”.

M: What about people in the other communities, will they change their beliefs and behaviour after hearing or seeing this message?

R2: “Yes, they will change”.

M: What is the best way that you suggest in giving out this message?

R3: “I suggest radio discussions emphasising the bye-laws”.

M: Yes Mama?

R4: “I suggest community sensitization, moving place to place to sensitized the people”.

R5: I believe much in the sensitization, as with community sensitizations the people may aware, than using radio, not everyone have access to radio”.

M: Ok, are the youth groups’ good messengers of this message?

R5: “yes”.

R6: “Yes, they are good messengers”.

M: Ok. What about you my sister?

R7: Yes, the youths are active and energetic”.

R8: the youths are recognised in the community, and they are part of the community, and very active in any developmental project, so sending them as messengers will be better.

M: What about the women’s group, are they good messengers?

R1: “Yes”.

R2: “Yes, the women’s group are better messengers”.

M: What about the Men’s group?

R2: “They are also good messengers”.

R3: “Both the women and men groups are good messengers of this message”.

M: Ok?

R4: “For me I prefer young men and female a messengers”.

M: What about the Ebola Survivors?

R5: “They are the best people”.

M: What about the radio drama as channel to this message?

R6: “I love that radio drama, it is a good channel”.

R7: It is good”.

M: Why?

R7: “Because it will capture the attention of a lot of people”.

M: Ok, yes my sister?

R8: “It is a better way”.

M: Ok. What about the community drama?

R1: “It is good but some people are afraid of the gathering for them not to touch each other”.

M: What about the radio jingle?

R2: “The radio jingles are also ok”.

M: What about the posters?

R3: “The posters are fine, because when it is posted, a lot of people will see”.

M: Where do you suggest the poster, to be posted?

R4: “At Ataya base”.

M: Ok?

R5: “Mosque, and churches, then community public gathering”.

**(NOTE: Topic 30 – Fear of ambulance – chlorine ” A beliv say di ambulance na di best en safe way fo go hospital. Fine breeze de blow inside the ambulance”)**

M: Ok, let us look at this other message about the ambulance which reads” ar believ say di ambulance na di best n safe way for go na hospital” what do you think of this message?

R1: “This picture is showing going with the ambulance at the hospital when you are sick is the best means of transportation, it has good ventilation, and it is faster than walking on foot”.

M: OK, so the message is good?

R1: “yes”.

M: Ok?

R2: “This message about the ambulance is fine, because using another means of transportation to hospital when you are sick is not good, because you don’t know the type of sickness that you are having and the tendency to transfer the sickness to other people is very simple, so the ambulance is the best and safest means of transport to the hospital when you are sick”.

M: Ok?

R3: “The ambulance is safe, if you are not suffocated with chlorine”.

M: That is not happening again, so with do you think of this message?

R4: “The ambulance is fast and safe means of transportation to the hospital”

R5: “The ambulance is the safest”.

R6: “The ambulance is the best a quickest means of transportation to the hospital”.

M: Ok, we are developing a poster to reduce the fear in people about the ambulance, is there anything about this message people may not like”.

R1: “No, people may like it, the message is good”.

M: Ok. Yes my sister?

R2: “The message is good, people will like it, because it is saying the right thing”

M: What about the krio and colour of the message and the picture?

R3: “The pictures are clear, the krio is ok, and they all appear good”.

M: Ok, this message is about confidence building in people, to reduce their fear of going with the ambulance, is this message clear?

R4: “Yes, it is clear”.

R5: “Yes, this message is clear, it increases the confident of people, because really it has not been happening like this, so if this message is now out, it shall be well”.

M: The people will change their beliefs after seeing this message?

R6: “Yes, they will totally change their minds or beliefs about the ambulance”.

R7: “Yes they will change their minds and belief about the ambulance”.

M: OK?

R8: “Yes, some people will change and others will not”.

M: Why?

R8: “The perception of people are not the same, but really majority will change their minds”.

M: Do the people in the other communities change their beliefs after seeing this message?

R1: “Yes they will change their beliefs, an attitude toward the ambulance”.

M: Ok?

R2: “Yes, they will accept the message and their minds will be totally changed”.

M: Ok?

R3: “Yes, if they have the same pictures and the message, they will change their beliefs”.

M: Ok, Which way is the best to give out this message?

R4: “Well, like how I said, in giving out this message, let used community sensitization, move door to door with the posters in hands, explained to them with is on the poster”.

M: Yes mama?

R5: “I suggest door to door community sensitization with the youths and community health workers”.

M: Ok, yes my sister?

R6: “The youths will carry on the community sensitization, move house to house explaining the content of the picture or posters”.

M: Ok, yes mama?

R7: “The same as my sister have said, community sensitization by youths”.

M: Who should distribute this message?

R1: “The community members, because some people hardly believe strangers, but if they see their community members they will take the message seriously”.

M: Ok?

R2: “The community people”.

M: ok, but can you please give me examples?

R2:”The chief, because people listening to the chief and adhere to whatever he or she says, the councillors are the others members that should give out this message”.

R3: “The youths are very important in distributing this message”.

M: Ok, is the ambulance drivers and other staffs are good messengers to this message?

R4: “No”.

M: Why?

R4: “Because they will be afraid of them”.

M: ok?

R5: “For me, I believe that, they will be much better”.

M: Why?

R5: “Because they are workers of the ambulance, so if they tell you anything about the ambulance, you will believe”.

M: Ok?

R6: “I don’t think they are important in giving out this message, because people will be afraid of them”.

M: But what about a family member, will they be the good messengers of this message?

R7: “If they have undergone the test once and they know much, they will be good messengers”.

M: Ok?

R8: “The family member that have survived from Ebola, when he or she talks, the people will believe”.

M: Ok?

R1: “Even the person is not a survivor, but hence he or she had the experienced, when they talked to me I will believe”.

M: What about the patients?

R2: “Yes, they are good messengers, because they have been infected and they can tell the true story.”

M: ok?

R3: “Yes, because the patient have ride the ambulance once and so they are good messengers”.

M: Ok?

R4: “They should have been the best, but they are not giving them the chance to do the sensitization”.

M: Will posters be the best channel to disseminate this message?

R1: “Yes, it is one of the best channels”.

M: “ok, why you said it is one of the best?

R1: “Some people are not educated, but by looking at the pictures, they can understand”.

M: Ok, yes mama?

R2: “It will be best if it is posted, but the best is one is when they hold it in their hands and move door to door, explaining to the people, that will be ok, but just looking at the picture will not be properly understood”.

M: Yes mama, what is your own take?

R3: “house to house is good, because not everyone will read and understand”.

M: “Ok, my sister?

R4: “well the posters are good, because some people will look at the pictures and understand the picture”.

M: Where this poster should be posted?

R5: “churches, mosques and cinemas”.

M: Ok mama?

R6: “Ataya base, mosques and churches gate and fence”.

M: Ok?

R7: “Ok, Ataya base, Cookery shop and Electric poles”.

M: Who should distribute these posters?

R8: “The community members”.

M: Yes mama?

R1: “The community members”.

M: How do they good about it?

R1: They will posted on the walls and share it to people”.

M: Yes mama?

R2: “The youth members in the community, the imams, pastors”.

M: How do they do it?

R3: “through sermons and community mobilization”.

M: Ok, I thank you very much for your time”
