# Supplementary material for: Development of a set of community-informed Ebola messages for Sierra Leone
Source: PLoS Negl Trop Dis. 2017 Aug 7;11(8):e0005742. doi: 10.1371/journal.pntd.0005742 (PMC5560759; doi:10.1371/journal.pntd.0005742)
Supplement: S1 Appendix — (ZIP) [file pntd.0005742.s001.zip › Ebola messages - FGD and interview transcripts/R2HC Ebola Fieldwork 2/R2HC Ebola F2 FGD-Male-Rural1-A.docx]

| CODE | **R2HC Ebola F2 FGD-Male-Rural1-A (**Rural focus group discussion with younger (<25 years) and older (25+) males, using **topic guide GroupA and Picture set A)** |
| --- | --- |
| DATE | March 2015 |
| DURATION (minutes) | 110 |
| Collector nrs | 2 and 5 |
| LANGUAGE INTERVIEW | Krio |

**PERSONAL DATA PARTICIPANTS**

| Nr | Sex  (*F/ M*) | Age  (*in years*) | Education Level (*e.g. none, Primary, secondary, tertiary*) | Language (*e.g. Mende, Temne, Krio)* | Religion | Job / Employment (*how they earn their living e.g. farmer, teacher, trader*) | Role in community  (*e.g. youth leader*)  ANONYMIZED, ONLY AREA OF ROLE INDICATED |
| --- | --- | --- | --- | --- | --- | --- | --- |
| 1 | M | 19 | Secondary | Krio/Temne | Christian | Student/Trader | None |
| 2 | M | 23 | Secondary | Krio/Temne | Muslim | Student | Youth |
| 3 | M | 21 | Secondary | Krio/Temne | Christian | Student | Youth |
| 4 | M | 34 | Tertiary | Krio/Temne | Christian | Student | Youth |
| 5 | M | 30 | Tertiary | Krio/Temne | Christian | Trader | Youth |
| 6 | M | 62 | Tertiary | Krio/Temne | Christian | Teacher | Traditional authority |
| 7 | M | 56 | Primary | Krio/Temne | Muslim | Farmer | Local government |
| 8 | M | 46 | Tertiary | Krio/Temne | Muslim | Farmer | Mosque |

**TRANSCRIPT: (M = Moderator, R= respondent, R1= first person responding to a question, DOES NOT correspond to numbering used in Personal Data!)**

**It appears - *Topic 25/26 – Stigma - “Kabo to una homes, una wokplace en una community, una wi broda en sista dem way don survive Ebola” en “Nor Laf or run from porsin way bin don get Ebola” (Do not laugh at or avoid Ebola survivors) en “Nor kongosa porsin way bin don get Ebola” (Don’t Spread gossip about Ebola survivors).)* was not discussed, possibly because it was only text and had no picture**

**(NOTE: Topic 7 - Burial - Posters – ”Wi respect dae dae bodi” en “Leh wi join an en gi wi pipul dem gud berrin”)**

M: Can you tell me what this picture is showing you?

R1: “Well this is a burial, because look at the corpse they are praying over it, the man with the spray, I see something like he is spraying, then the Imam is in front of the corpse praying”.

M: Is there any other thing that you can see?

R1: “I can see the man spraying but the corpse has not been put into the grave yet”.

M: Ok, yes?

R2: “Is like it is that traditional way of buying because I am not seeing a plastic on the deceased”.

M: Ok, yes?

R3: “What I understand is, because when someone dies of Ebola they can come and pray over the corpse”.

M: Ok, yes?

R4: “As me, I can see the coffin but I have not seen a dead person I don’t know whether the dead body is already inside it, then I also see the Burial Team on one side and the person spraying on the other side, and there is also a Pastor and the parents on the other side standing behind the Pastor and the Pastor is far away from the corpse, he did not (come) close to the corpse likewise the other side”.

M: Yes my own brother?

R5: “I understand that this is an Ebola process, and I see that the people are bereaved, and I see the action of spraying”.

M: Ok, Yes?

R6: “Just like what my brother has said, it is about the burial process. People have been grumbling that they are not satisfied with the way they are burying their people, so they have made it simple, now you can witness where they are burying your relative, that is the process they are on in this picture”.

M: Ok, yes?

R7: “Is the same thing, the process and the people are keeping far away from the dead body, the Imam is in the front and the people at the back and I can see the corpse and the person who is spraying.”

M: Ok, so is this fine? Do you think people can understand it?

R7: “Yes, people will be able to understand it”.

M: What about this, which reads: “We respect dead body”, and “Let us join hands and give our people befitting burial”. What do you think about this message?

R8: “Well this message is fine because in the past when Ebola broke out the way they were burying people the community was not happy about it but now if they include all the community people they will feel fine because they will see how they bury their people and they will know whether they were buried properly or not”.

M: Yes?

R1: “In the past, the community people were not seeing the corpse, when someone dies they will just come and collect the person and go and bury the person somewhere else, also when somebody dies the Burial Team will just come and dress it without the involvement of any community member and that brings about conflict in the community, but when somebody dies now the Burial Team will come and dress it where the people are present and when they want to bury they give some feet to the corpse and pray over it and see where they bury the person, I think they will feel fine than they just bury the person where they do not know the place”.

M: Yes?

R2: “In the past, the way they used to bury was not fine, but now when they have joined hands with the Community, there is no problem again”.

M: Ok, yes Sir?

R3: “Like what the paper is saying, let us join hands and bury our people. That is just to tell us all to come together and bury our people”.

M: Who is we?

R3: “The Community?

M: Is it only the Community or those who also work with the Burial Team?

R3: “No, the bereaved family, that is what they are trying to tell us that let the bereaved family and the Burial Team join hands to give the corpse a befitting burial”.

M: Ok, yes?

R4: “Like what the paper is telling us that we should respect dead body, I hope that the last respect that they give a person, according to our own tradition, the last respect they can give a person is when they are burying the person who is very important to the people. But during the outbreak of Ebola the way they were treating the dead people were not happy with that but now when they have come with the message that we should join hands, we the Community and the Burial Team should come together. The first picture shows us that the people went to the graveyard but they kept far off while the Pastor prays over the corpse. The message is very fine”.

M: Yes Sir?

R5: “What I have to say is, in the past they were burying people properly but now I see that there will be a Pastor and the family members will be there and also to the Community people to give last respect to the dead”.

M: Yes?

R6: “Well the photo there is trying to tell us that, it teaches us how to respect for the dead and this is the way we should follow to pay last respect”.

M: Yes?

R7: “To me the first thing I see there, I want to say here that in Sierra Leone we need to get a special Burial Team because naturally when somebody is dead the person has no use in this world again the only use the person has is the after dead, where the person is going. We believe that if all of us come together, because at the initial stage we were afraid of the Burial Team because when we see them, the Ambulance and noise but thank God they have thought otherwise for us to come together, the Burial Team are not extra people, they are our own people so they too have the feeling that they have to be very moderate in handling the corpse, so with that I am recommending that minors Ebola now, we should have a special team because we have started seeing some respect because now they treat corpses fine. Let us continue to practice this and have a special team that should be burying people.”

M: So do you think people in this community cam agree for this kind of message?

R8: “Yes, because the picture depict everything”.

R1: “Is not only the Burial Team, we all have to come together and show that respect, I believe they will accept”.

M: So, is there anything about this message that is not clear?

R2: “I don’t think so, everybody understands it, it is clear. This is what that brought the between the Community, the bereaved family and the Burial Team because they did not accept what the Community wants and they were playing with corpse the way people did not want. But when they came together to bury there is no more problem”.

R3: “Like this picture, I can say it is fine because some people were not buried with that white cloth again, as I am now if I die of Ebola they will just out me into the bag but thank God they are now using the white cloth and I think our people are commending that greatly, in fact if you show these pictures to the Community people they can interpret “.

M: So do you think if we take this message to the other Communities they will change their way of thinking and behaviour?

Rs: “YES”.

M: So why do you say they will change?

R4: “I said so because what the Community was doing there is only a slight difference. If they give respect to the dead, now they bury with the white cloth and do everything that they used to do before, they wash it so I think they can accept it”

M: Like you said, do they wash the body now?

R5: “No, no they do not wash it, they will dress it and give it the white cloth like before, so I think if they continue with that there will be no problem in the Community”

M: Yes Sir?

R6: “In fact in the past when they used to come they will not talk to anybody but now the way they are treating them we have no problem with. In the past when they come they will remove all the think of the dead person and put the person naked, but now when they come they will ask if the person was prayerful so everybody is happy and everybody is now aware”.

M: Yes Sir?

R7: “People now appreciate the way they bury people because even the inscription on the paper is saying let us join hands to give our people good burial, you find out that it is written in Krio, if you interpret that to the people in our Community they can understand it well and then if you explain to them and they are present at the burial of their loved ones they will understand and nothing will go wrong again”.

M: Yes Sir?

R8: “Is the same thing, I think the Community which has been affected by the disease is now part of the burial even when they do not take part. According to the information we were getting that people were just flinging body like that, they were not allowing people to even go nearer to the burial site but now I think if the decease family is around he or she can see the way they are going to treat the person, even if somebody wants to misbehave they people will tell that person that things have changed and that we are now part of the Burial Team. Even though we do not take part but they see us and give us the respect so you can feel safe and take it that we have been respected our person who has died. So I think people will accept it”.

M: Yes Sir?

R1: “I think everything is just the same, because if I have seen that they have given my relative a befitting burial I can go out and spread the message that people should not be afraid and that the Team means something”.

M: Ok, yes?

R2: “By this I think there is going to be better changes. I said so because this is part of learning and the way they were treating our people, they were not treating them fine, so by these posters we can learn how to obey the rules”.

M: Yes Sir?

R3: “To me, I just see the Burial Team as now that they have changed because normally before Ebola outbreak when our relative dies you will have taken care and do everything, you will dig the grave and bury but now that we have a Team that is so careful about burial now, I think the people will appreciate. But the only thing I am doubtful of is the area of washing, because we do not know if the Burial Team has the mandate to wash the corpse, I think that is the only difference. Before this time there were special people in the Community who washed dead bodies. I think the Burial Team should also take over that to wash dead bodies because you cannot just go and bury person without washing”.

R4: “Well the other thing is, according to what we hear that when you play with a dead body then the sick can transfer to you so we have to take three or four years before washing dead bodies then if the sickness will go and therefore people should refrain from washing dead bodies. The respect they give the dead is better than when they just go and throw him or her like that”.

M: So which way do you think is the best way for this message to reach the people in these Communities?

R5: “The first thing is we are also supposed to spread the message because message is not spread by one man. Some time when I leave here I will go and sit somewhere and talk about what we have learnt from you and they will get the experience so by doing that the message will go”.

R6: “What I am saying is that we need to have a team, mobile team that goes from village to village to spread the news to the people and ask their consent after that, then we discuss it like we are doing now, that will be more appropriate. We have to ask the people what is their own view from there we give advice as to what to do since we do not know if the sick will come back. We should not just rely on pictures, there should be a team moving from village to village”.

M: Yes Sir?

R7: “What I am suggesting is that, just as you have brought this paper, you photo copy it and take it to the people and show them what to do so that they can change”.

M: Yes Sir?

R8: “Just like what my elder has said, like this paper you have to enlarge it and post it somewhere where people can watch it or even demonstrate and give the information to the people”.

M: What do you mean?

R8: “You have to make this paper in a big form and post it somewhere in the town so that anybody passing can see it from afar, and let there also be a Team to move from house to house and explain to the people what the paper is saying”.

M: Yes?

R1: We can also print handbills and to the Communities and show people then that can be better”.

M: Yes?

R2: “These pictures can be of great help because some people when they see it some people can recall”

R3: “Yes, is about this Burial Team. Because we don’t want to rely on a moving team from one Chiefdom to the other, if we have a team within (--name of interview town--) because here is the Chiefdom Headquarter that it could be very easy for the team because some areas when somebody dies it takes about two or three days to bring the person but if we have a team no sooner somebody dies they can go and bury the person”

M: So what do you think about these people; The Burial Team, if the Burial Team brings this message to you, will you accept it?

R4: “Yes”.

M: Why will you accept it?

R4: “Because they want situation cool down, because there used to be tension between the people and the Burial Team”.

M: Yes?

R5: “The best people to pass the message are the Pastor and the Imam. The people if you are in Church he can pass the message or in the Mosque the Imam can pass the message. And secondly, the Chiefs because the Chief owns the people whatever he tells them is what they believe, that is what I have to say”.

R6: “The Burial Team, yes, but the best people to pass the message is the Community people, because the Team is just for few time because when they come they are always urgent that they have to go and do this and that, they will not pass the message as the people want it but the Chiefs can call general meeting and explain to the people, certain people will be there to explain to the people. If the Burial Team come with the message again is just to brainwash the people”.

M: Would you like the Burial Team to come and talk to people about this message?

R6: “If the Burial Team brings this thing before the people when they are not aware of it is a problem they will take it to be a provocation because the people will always say is because they have given them money that is why”.

M: Ok, do people pay money to the Burial Team to bury their people?

R6: “I think when they come to bury here they pay them for that”.

M: So you are ok with the Imams, Pastors and Chiefs not so?

Rs: “Yes, that is one but is it everybody who prays.”

R7: “Is not everybody who prays but if I go to the Mosque and get the message when I come back home I spread the message”.

M: Ok, what do you think about the ways to carry these messages to the people? Are these posters fine?

Rs: “YES THEY ARE FINE”.

M: So where do you think they should put these posters?

R8: “One is the public places”.

M: Like which public place?

R8: “Or if they are enough we can share it from house to house, after the meeting we can give it to those who are present”.

R1: “Like the Community Centre, ‘Poyo (=palmwine) Bars, those are all areas”.

M: So who do we give these posters to be distributed? Who can be right person to send the message?

R2: “The Chiefs, because he has the Community, he has the Pastors and the Imams, in the Church the Pastor is the Leader and in the Mosque the Imam is the Leader. But the Chief has the town, so wherever you think people gather you have share this paper”.

R3: “Like in this we have the Chiefdom Councillors, you call a Chiefdom meeting and share the message with them so when they go to their own area they will share those things, then if it is here you can even send it to other villages”.

**It appears topic 16 and topic 30 were discussed in one go**

**(NOTE: Topic 16 – Misconception about health system – picture album)**

**(NOTE: Topic 30 – Fear of ambulance – chlorine ” A beliv say di ambulance na di best en safe way fo go hospital. Fine breeze de blow inside the ambulance”)**

M: Ok, so what do you think about messages of the Ambulance coming to take people for burial?

R4: “Some people feel that when they come to take somebody is to go and kill the person”.

R5: “That is the most threat for the Community, the Ambulance, when the Ambulance comes to take the person, the noise makes a lot of people to get confused but it is good to sensitize the people that what they doing and is trying to protect us and the people. If they spread that message everybody will understand that it is good for us, nobody will feel bad. The reason why people were feeling bad about this Ambulance is that when they come and take somebody they will later report that the person is dead and when they come to take another person is the same thing so it reached a point when everybody was confused when they hear the siren they will begin to ask who is the next victim and they will send good bye to the person they are taking away. But when changes came and they brought the Centres close to where they are, when the Ambulance comes and they see it, I believe that the minds of the people are changing”.

M: Yes?

R6: “If I should answer your question, why can’t you make it a leaflet like this, say two or three papers with larger pictures, like this it is durable someone can even put it into his or her pocket and go with it.

M: Ok yes?

R7: “The other advise is, we want the album because the album is not for us but for those coming behind us, even if the sick will not repeat itself it will be an advise to the young ones that there was a sickness like this.”

M: Yes?

R8: “I just want to make an addition to my elders have just said about this Ambulance. The Ambulance is just a thing, without human being the Ambulance is nothing. The drivers drive out people in a manner that is not good that if the sickness is serious the person will die on the way. So the drivers need to be sensitized bout how to go and take Ebola patients to hospital”.

M: Ok, the picture album is like this, one is showing you the Ambulance with the Health Worker inside smiling, while the other one is showing the Health Worker taking s swab and the other is showing you inside and outside of the Holding and Treatment Centres. How do you see that, is it fine for you?

R1: “Yes, it is fine”.

R2: “The idea of the picture is fine, but we should sensitize people towards actions, like you said when they come to take somebody, you see the nurse smiling, That is very important. But according to the information we get, like the phone you talked about, but when they come to take you before entering the Ambulance they will search his or her pocket and take whatever you have, that used to happen. But if they come with the picture album and they see the way they treat people from their homes to the Ambulance and they see the background. But they also told us that when they carry you to even get food is a problem but if they come with the picture and people see the action about the Holding Centre they will know that there is care at the Centre”.

R3: “The information we also get is that they said Ebola has no medicine and even the nurses do not touch people again, but if they see a medical somebody taking care of somebody I believe that people will take the venture to go to hospital or to call for them to come and take the person that will be better”.

M: Yes?

R4: “Some people because of the statement that the survivors to hospital bring home, some are afraid to go. Even the Contact Tracer do hide the patients for fear that when they go they will not come again. So they really need to go and explain to them so that they can believe”

M: But why do you think the boy told the Health Worker that his father is sick?

R5: “Is because of the care that he received”.

M: Do you think people in this community and other communities will accept this message?

R6: “Yes they will like it because it will give them the zeal to go”.

M: So which aspect of this picture message that people do not understand?

R7: “No, even the community, we have to see. When we see, we will believe”.

M: So if this kind of pictorials comes here, do you think it will change peoples’ behaviour and thinking?

R8: “Yes, it will change”.

M: Why do you think it will change the behaviour and thinking?

R8: “Because you have already seen the action the picture is showing so you will learn a lesson and change”

M: So you think the actions will speak louder than just voice?

R8: “Yes”.

M: Yes Sir?

R1: “When this Ebola actually gripped us, the people who in the area where the sickness affected were afraid to come out when they come to take them they refused to come out, but when the survivors came back it changed the perception. Like a woman who came back, the day she reached here, people gathered to see her. That was the time sick people started coming out, although they died later, but that was the time they started coming out, but they were not coming out. When they saw the return of that woman that was the time a lady whose baby had died in her stomach long ago came out”

M: So who would you like to bring this message to the people?

R2: It is very important for the message to pass through the Chief, even if the Chief sends someone the person will tell the people that the Chief sent him or her to disseminate the message so the people will accept the idea”.

M: Yes I see you shaking you head?

R3: “Is just the same thing”.

M: Yes?

R4: “I support”. “.

M: So you think the Chief is the right person to carry the message to the people?

Rs: “YES”.

R5: The other thing I want to add is the can be able to disseminate the message in his own Community but for the other Communities if the back up the Chief with other young people to help relay the message from one village to the other I think it will be much better”.

M: So what do you think about the messengers of these messages like the Health Workers?

R6: “I said it the last time that the people had lost trust in the medical people, they no longer have trust again that is why many died at home because some when they carried them they never came back. So like I am saying, the Chiefs, Imams, and Pastors are very important to talk to the people in the Community. But a lot do not trust the medical people

R7: “No, well like the medical people are important for the pregnant women and lactating mothers because when they to clinic they pass the message to them, and even if the medical people are going to pass the message somebody within the Community should be with them who they can trust. They brought a complaint to us that even when they are sick the nurses do not touch them”.

R8: “The other thing is we have to involve the community people”.

M: Yes? So do you think people in this Community will accept this message?

Rs: “YES”.

M: So is there anything about this message that you do not like or the people might not like?

R1: “No, this one is direct is like preaching, telling how to live with your brother or sister, so I think this is a clear message to everybody”.

M: What if the young men and women bring this message to you, would you like it?

R2: “They are the ones who has the problem, if they come with the message it will spread more than we the elders. The younger ones are plenty if the message reach them we all will be safe”.

M: Yes?

R3: “Like what my brother has just said, the youth are very important to send the message, they are very active”.

M: So it can be fine the youth to carry the message?

Rs: “YES”.

R4: “Yes the youth can be very appropriate for this. They can even dramatize the message for the people to understand better”.

M: So what about the women’s groups?

R5: “The youth can even blend with the women to dramatize the message; the women are very good at that”.

M: What about the Ebola Survivors, if they come with the message will you accept it?

R6: “Well we will accept it because they have to be part of the team to tell people to stop stigmatizing, to stop running away from them”.

R7: “The thing is they (Survivors) come alone to talk to us it will look provoking to them but let them mingle with us, they should be part of us”.

R8: “When they mingle with us those who do not trust them can trust them now”.

M: Yes Sir I saw the hand up?

R1: “Well just like what my brother said, it will be kind of provoking”

M: So will people in the Community like this?

R2: “For the Ambulance, this is a very big Community; it is an amalgamated Chiefdom so there should be an Ambulance here because there are times people feel sick at night and there is no way to carry the person. So now that you are here we are ready to accept everything that you tell us”.

M: So like this message here is telling us to trust the Ambulance, it is the best way to carry safe to hospital and do not be afraid to enter the Ambulance. So do you think the people will accept this?

R3: “They will understand because the person who is carrying the message is familiar and like I said through the drama they see how the Ambulance comes to collect somebody and carry the person”.

M: So do you think this can change peoples’ belief and behaviour if we take this message to them?

R4: “Yes”

M: How do you think it will change their behaviour?

R4: “Is the same thing, if somebody comes and tells you about something which he or she believes I believe too that your own belief can change”.

M: Yes?

R5: “Say if someone is sick here and then the Ambulance comes and carry the person, if the person gets well and comes back he will tell the others about it”.

R6: “Yes, I don’t know if the Organization you are working for can provide us with T-Shirts so that when going for the programme we wear it”.

M: So apart from the Youth and the Chief, is there any other way you think this message can the people?

R7: “The hospitals, the schools, at the PHUs they can see the pictures”.

R8: “Like for the lactating mothers before starting clinic they show it to them during Health Talk”.

M: So if the Ambulance Driver brings this message you, will you accept it?

R8: “It will not hold, you have to involve the community people the Ambulance man should join the youth in the town, but him alone cannot just come to talk to people, people will not even go there. Like one Ambulance Driver who went collect a corpse, he packed the vehicle with the corpse inside and started playing music, people talked but he couldn’t listen”.

M: Did you report the driver?

R8: “Yes they reported him and he was sacked”.

M: So what about the Community members if they too come with the message will you accept it?

R8: “No, with that no problem”.

M: Yes?

R1: “I just want to add something about these Ambulances, since the outbreak of this sickness in the country we only see those white Ambulances and this is an illiterate community it can be very difficult to convince one person that the Ambulance which carried the corpse is not the same Ambulance that comes to collect the sick so they should have colours”.

M: Ok so let them have different colours?

R1: “Yes”.

M: Like which colours would you suggest?

R1: “Like we have white, if we can get Red, blue..”

R2: “Ah, Red is dangerous, if can get yellow, green, can be fine”.

M: Different colours?

Rs: “YES”.

M: Ok I thank you all for your voices and may God help all of us.
