# Supplementary material for: Development of a set of community-informed Ebola messages for Sierra Leone
Source: PLoS Negl Trop Dis. 2017 Aug 7;11(8):e0005742. doi: 10.1371/journal.pntd.0005742 (PMC5560759; doi:10.1371/journal.pntd.0005742)
Supplement: S1 Appendix — (ZIP) [file pntd.0005742.s001.zip › Ebola messages - FGD and interview transcripts/R2HC Ebola Fieldwork 2/R2HC Ebola F2 FGD-Male-Rural2-C CORRECTED.docx]

| CODE | **R2HC Ebola F2 FGD-Male-Rural2-C (**Rural focus group discussion with younger (<25 years) and older (25+) males, using **topic guide GroupC and Picture set C)**  **7^th^ April 2015: corrected participant data** |
| --- | --- |
| DATE | March 2015 |
| DURATION (minutes) | 125 |
| Collector nr | 5 and 2 |
| LANGUAGE INTERVIEW | Krio |

**PERSONAL DATA PARTICIPANTS**

| Nr | Sex  (*F/ M*) | Age  (*in years*) | Education Level (*e.g. none, Primary, secondary, tertiary*) | Language (*e.g. Mende, Temne, Krio)* | Religion | Job / Employment (*how they earn their living e.g. farmer, teacher, trader*) | Role in community  (*e.g. youth leader*)  ANONYMIZED, ONLY AREA OF ROLE INDICATED |
| --- | --- | --- | --- | --- | --- | --- | --- |
| 1 | M | 40 | Secondary | Limba | Christian | Tailor | None |
| 2 | M | 20 | None | Limba | Muslim | Farmer | None |
| 3 | M | 42 | Secondary | Limba | Christian | Farmer | Church |
| 4 | M | 49 | Tertiary | Limba | Christian | Teacher | Health |
| 5 | M | 70 | None | Limba | Muslim | Farmer | Traditional authority |
| 6 | M | 67 | Secondary | Limba | Christian | Farmer | None |
| 7 | M | 45 | Primary | Limba | Muslim | Farmer | Mosque |
| 8 | M | 24 | Secondary | Limba | Muslim | Student | Health |

Audio check done: transcript complete

**TRANSCRIPT: (M = Moderator, R= respondent, R1= first person responding to a question, DOES NOT correspond to numbering used in Personal Data!)**

It appears the last topic of Set C : **Topic 33 - 117 prank calls –“Lek Mama Salone – no mek kalo kalo call to 117, e de hambug the Ebola response”** was not discussed in the group, possibly because it had no picture.

**(NOTE: Topic 12 – Early treatment - Poster – “Na because ar go hospital quick, na dat mek ar well” (poster of survivor)**

M: Ok, I have this picture to show you. What do you think about this picture?

R1: “This is somebody who has been sick, but survived and has now been give paper to show that he is a survivor to prove that she no longer has the sickness”

. M: Ok, Sir?

R2: “Well is the same thing, he was attacked by the sickness but now he is well”.

M: Yes Sir?

R3: “Is somebody who has between but is now well”.

M: Yes Sir?

R4: “Is somebody who has been sick by Ebola but survived so when coming out they gave her a Certificate to prove that she is well from the sickness”.

M: Ok, yes Sir?

R5: “The person I see that he has been given a certificate

M: Yes?

R6: “This is to show the whole country that this a man who has been sick but he is now well and we do not need to discriminate him”.

M: So the message is fine?

R6: “Yes”.

M: Ok, this one says, is, “because I went to hospital quick, it makes me well”, is there anything about this message that you do not understand?

R7: “The message is clear that when anybody is sick you have to go to hospital, you should not wait until you are overcome by the sickness, maybe the person will die at the hospital”

M: Ok, yes?

R8: “Do not keep the sick secretly; else it will be difficult for you”.

M: Yes?

R9: “It is always important for one to take note when the body is not feeling well for two or three day you should go to the hospital, this Ebola sickness is not something that a person should hide. When you go quickly to the hospital we can get well and come back home. That is why it is fine to get information like we are getting it now”.

M: Yes?

R1: “It is not all sickness that is Ebola; some are just common sickness before you wait until it gets chronic you should go quick to the hospital”.

M: Ok, yes?

R2: “This is to tell us that if we have any signs and symptoms of Ebola when you report quickly you can get well like this man in the picture”.

M: Ok, so is there anything about this message that people will not like?

R3: “Yes there is, some people when they are not well they deny because they do not want to go the hospital and when it gets worst”.

M: Ok, this message is saying that “Is because I went to hospital quick so I got well”. Is there anything on this picture that the people will not like?

R4: “No”.

M: Is everything fine?

Rs: “YES”.

R5: “It encourages people to go to hospital, some of them who went hospital were cured and they are better”.

M: So do you think people in this community will like this message?

R6: “YES”.

M: Do you think this message can change the belief of the people in this community?

R7: “There is nothing there that they will condemn”.

M: Yes Sir?

R8: “This picture has shown that everybody has the zeal to go to where they cure Ebola, and secondly this picture has shown that even the compound where you are you should keep you environment and even the water you drink you have to take time. And I know all the women will be glad to go close to the hospital for them be cured”.

M: Ok, yes?

R9: “Well some of them when they see the picture they will know that when they are sick they should go to the hospital quickly”.

M: Yes?

R1: “When the women see this woman holding the certificate they too will be happy to go hospital. Mostly it is the women who die of this Ebola because they take care of the men and children so they were ones hard hit by Ebola, then even in the hospital women are plenty who are nurses”.

M: So do you think people in this community and other communities will change their belief when they see this message?

R2: ”When people see this message it reduces their fear so if they use the survivor to send this message it will work more”.

M: So what do you think about the ways of carrying this message to people? Or where do you think we can put these pictures?

R3: “Well mostly at the Centre, like here we have a Mosque and two Churches and we also have the Barry so I hope when you come with these pictures you paste it at the Centre areas”.

M: Yes?

R4: “At times it is fine we do it house to house, it will help because nobody will leave his or her again to go and see the picture, when I have it at home I can read and explain to my relative”.

M: Yes Sir?

R5: “For us this paper is like the Pastor and the Imam can talk to the people. You can only give us enough of these papers even if it is the last village we can carry it there and talk to the people so that they can understand as for us here even if it is one we can teach our people, even two is enough for the one Church and one Mosque”.

M: Yes Sir?

R6: “It will be fine for any village to have a calendar and if they have to print it let them print it bigger than this so that people can understand the picture more to show that what you about to do”.

R7: “Yes like for me I am at the junction if anybody happens to pass by he or she can read it”.

M: Yes Sir?

R8: “But to spread it all over, these papers has to reach there so that they too can get the message”.

M: Ok, you have talk about the people who should carry the message. How can they carry this message?

R9: “Well you only have to give it house to house”.

M: Why did you say so?

R9: “Well I said so because some do not go to Church or Mosque so if you just leave it at the Mosque or Church those who do not go to Church or Mosque will not get the message so it is fine you give every household that can serve as a reminder”.

M: Ok, yes?

R1: “Like if I have the papers and go to ..........I will first tell the Chief that let every member of a household to stay at home with his or her people when you give the paper you should be able to explain about the pictures because it is not everybody who can read so when you explain about the picture to them they can only use the picture now to remember what you have told them”.

M: Yes Sir?

R2: “It is fine that you came to see us, if did not call us we will never know what you are here for”.

M: So what do you think about this message, is it clear?

R3: “Yes this message is clear for those who stubborn and those who are saying that Ebola is not real”.

**(NOTE: Topic 20/21 - Early Treatment and Prevention – “ Noto pass you see before you believe se - Ebola de” )**

M: This message says, “You don’t have to see before you believe that Ebola is real. Is this message clear to you?

R: YES”.

M: Will people understand it?

R” “YES”.

M: Yes?

R4: “Let us believe that the sickness is real, we should not deny that it is real”.

M: Yes?

R5: “To us, not everybody believes that Ebola is real is only when it killed many people here before they believe it is true, Ebola is real so everybody is now taking time for the sickness not to spread in the country”.

M: Yes?

R6: “Let me help with the Traditional area, to us the Limbas when a person dies if the person is a man we have the traditional way to bury. One Muslim man who had been to Mecca died so he people said he was not buried properly because he was not buried with properties he brought from Mecca so they went and exhumed the body at night. All those who were involved including the woman are all gone. So it was as a result of tradition that they all died”.

M: Yes Sir, so what is it about this message that people do not understand?

R7: “Well for me is not that one has to see before believing that Ebola is real, people needs to know that this sick is real”.

M: Ok, so which way do you think is the best way for this message to reach people?

R8: “The best is when you give us the papers so that we can pass on the information to those who are not here”.

M: So who do you think can take the message to the people?

R8: “Is us the youth, then we have the training, we can be able to pass the information to those who are not here we the youth can do that because we are so active to do those things”.

M: Yes?

R9: “I am still emphasizing on the Churches, the Pastors and the Imams are playing the biggest role although some people two Sundays or Fridays without going to the Church or the Mosque but one two members of the family and bring the message home to the person who did not go to Church or Mosque”.

M: Yes Sir?

R1: “Like the Stakeholders, like this Pa sitting here if he take this paper anywhere and talk to people they will listen to him and as we cannot go to all places we have to encourage people to listen to Ebola messages on the Radio”.

M: Yes Sir?

R2: “This one it has to be each one reaches one, everybody has to volunteer”.

M: Do you think Survivors can be the right persons to carry the message?

R3: “Yes, because they have been affected by the sick so they have vast experience and can explain better everybody will have confidence that it is true”.

M: Yes?

R4: “Well to us it is fine because Survivors have seen and been in the hospital and is now well and back I think he or she can be best to carry the message”.

M: What if the Health Workers and a ‘Meresin man’ (=traditional healer) brings the message to you will you accept it?

R4: “Yes Sir, once he has the paper”.

M: So why would you accept him apart from holding the paper?

R4: “Why I would accept him, he now believes that Ebola is real and we need to control ourselves, so I need to believe him”.

M: Yes?

R5: If a Doctor comes to tell me that Ebola is real I will believe because Doctors too are dying, I will believe”.

M: Yes?

R6: “To if a Doctor or Nurse comes to tell me that Ebola is real whether he comes with this paper or not I will believe because they too are dying. But like the herbalist when he comes to tell me I will not believe because he is not at the Centre (= health centre) what can he tell me about the sick or Ebola and besides government has banned them so I will not believe them mostly”.

M: Ok, is it fine to bring the message to you one on one?

R6: “Yes Sir”.

M: Why is it fine that way?

R6: “Because when you come to teach me I understand better and I will believe that it is true”.

M: Yes?

R7: “It is fine because if it is face to face now I will concentrate on what you tell me than I get it from the Radio. The face to face is fine”.

M:  Yes?

R8: “Well it is fine, but to me the message does not move faster it will consume time, like when you are here now if you say is going to be one on one it will take you a lot of time and energy and you will be here for some days but if it is like a drama you can only talk once and thousands of people will hear you, like say the Radio and the master plan we are hearing about it can cover the whole area”.

M: So what is your own suggestion if we say the face to face way?

R8: “Like what we are saying, go to the villages and announce to the people that they should not touch themselves and have the people who perform I think within 30 minutes you would have educate the whole village then you move to another area, you can even use other people in the community”.

M: Yes?

R9: “When this Ebola started newly some people came and performed drama, they way they performed at the Barry I was think too far about it so instead of doing it face to face, the drama can carry the message within one hour so it is better than the face to face”.

**(NOTE: Topic 24 – Leaflet - “6 steps while you wait for the ambulance for burial”)**

M: This other message says, “Believe the Burial Team when they come they will treat the patient fine, then give all the properties of the dead person and hand over all the things you used on the dead person they will know what to do. If they come with this message will you understand it?

Rs: “YES”.

M: Yes?

R1: “We do understand but not everybody understands the Krio and English writing”.

M: Yes Sir?

R2: “You have to translate it into English”.

M: So do you think people will understand it in the villages?

R3: “Well to me, as long as two or three are there to read it in Krio it is not difficult to understand but in English...hmmmm anybody who went to school can read it. Like this one, ‘Anybody wae die na ouse nor touch am’. Me I want everything to be in Krio”..

M: So, like all what is here, is it clear to people?

Rs: “YES”.

M: Is there anything here that is not clear?

R4: “It is clear for us who read Krio”

M: So what about this other one?

R4: “They are all important, from the Church, house to house, from the Mosque, house to house the message can go faster. If somebody come by and meet the message he or she can carry it to another village”.

M: So where do you think we put these pictures?

R5: “They are mostly fit at the Centre here, at the Barry, the Mosque, the Church then also on the Highway, they should enlarge it to make it fine”

M: Yes?

R6: Then also the Youth Leader is supposed to have it, the youth Leaders are now working in the communities more than the village mostly he solves the problem of the youth so if they have the message they too can spread it”.

R7: “Men and women too should be involved. Like when both of you are here now, where you forget this madam can remind you”.

M: So what do you think about these ways to carry the message to the people? The Radio Jingle, the mounted loud speaker

R8: “Well that one is fine for the urban area but where there is on line they cannot be able to make it, they have to train people to move from place to place, like when you come to sensitize us ii works better than the others”.

M: So what about text messages?

R9: “Well like most of our brothers do not have phone and cannot read text messages”.

R1: “The other way again is by mounting speaker on top of vehicle even if someone is in the bush he or she can hear it”.

M: Ok, I thank you very much.

Rs2: Thank you too”.
