# Supplementary material for: Development of a set of community-informed Ebola messages for Sierra Leone
Source: PLoS Negl Trop Dis. 2017 Aug 7;11(8):e0005742. doi: 10.1371/journal.pntd.0005742 (PMC5560759; doi:10.1371/journal.pntd.0005742)
Supplement: S1 Appendix — (ZIP) [file pntd.0005742.s001.zip › Ebola messages - FGD and interview transcripts/R2HC Ebola Fieldwork 2/R2HC Ebola F2 FGD-Male-Urban1-D.docx]

| CODE | **R2HC Ebola F2 FGD-Male-Urban1-D (**Urban focus group discussion with younger (<25 years) and older (25+) males, using **topic guide GroupD and Picture set D)** |
| --- | --- |
| DATE | March 2015 |
| DURATION (minutes) | 115 |
| Collector nrs | 1 and 4 |
| LANGUAGE INTERVIEW | Krio |

**PERSONAL DATA PARTICIPANTS**

| Nr | Sex  (*F/ M*) | Age  (*in years*) | Education Level (*e.g. none, Primary, secondary, tertiary*) | Language (*e.g. Mende, Temne, Krio)* | Religion | Job / Employment (*how they earn their living e.g. farmer, teacher, trader*) | Role in community  (*e.g. youth leader*)  ANONYMIZED, ONLY AREA OF ROLE INDICATED |
| --- | --- | --- | --- | --- | --- | --- | --- |
| 1 | M | 27 | Secondary | Krio | Christian | Electrician | None |
| 2 | M | 21 | Tertiary | Krio | Christian | Student | None |
| 3 | M | 21 | Secondary | Krio | Muslim | Student | none |
| 4 | M | 24 | Secondary | Krio | Muslim | Student | None |
| 5 | M | 28 | Tertiary | Mende | Muslim | Teacher | None |
| 6 | M | 29 | None | Madingo | Muslim | Trader | Youth |
| 7 | M | 28 | Primary | Mende | Christian | Disc jockey | none |
| 8 | M | 53 | None | loko | Christian | Trader | none |

**TRANSCRIPT: (M = Moderator, R= respondent, R1= first person responding to a question, DOES NOT correspond to numbering used in Personal Data!)**

**(NOTE: Topic 15/17 –Misconception healthcare system- ”Wea yu sick, nor fraid fo go hospital, den go hol yu fine” en “Wea yu sick, nor fraid fo go wit di ambulance, den go hol yu fine”**)

M: We are talking about the misconception in the health care system, now I am showing you this text and I will read it to you, it reads “wea yu sick, nor fraid fo go hospital, den go hol yu fine, nor fraid fo go wit di ambulance, den go hol yu fine” What do you think of this message?

R1: “Well, this point to my own view it is good, because when you are sick, if you stay at home without going to the hospital, you may not know the sickness that you are suffering from, but if you go to the hospital to the medical experts, you will be diagnosed at the hospital and a treatment will be prescribed and administered to you”.

M: Yes pa do you have anything to tell us about this message ,wea yu sick, nor fraid fo go hospital, den go hol yu fine, nor fraid fo go wit di ambulance, den go hol yu fine”?

R2: “Well the message is good, but some people in this community may not see it as a good message”.

M: Why?

R2: “Because we already had sicknesses in this country which are not hospital sickness”.

M: Like which ones?

R2: “In our native homes, people believe some sickness are properly handled by traditional healers not hospitals, so how can the people go to hospital with this kind of thoughts, that they have, the message is good, but to disseminate this kind of message in our local communities is not easy, because they had in mind that a certain sicknesses are not for hospital”.

M: But what do you think about this particular message?

R2: “the message is good”.

M: Why do you think it is good?

R2: “Because I believe that when you are sick, to go the hospital is the best and reliable”.

R3: “This message is very fine”

R4: “Well to me, this message is good because it gives more confidence to the people”.

R5: “The message is good, but it depends to the understanding of the people”.

R6: “I am very much impressed, because I have never seen this type of message of confidence building”.

R7: “To me the message is clear and understandable”.

R8: “Well the message is fairly good”.

M: What is this message about?

R1: “Well this message is about building confidence in the people to go to the hospital when they are sick, because during this outbreak, people were afraid to go the hospital, but this message will build their confidence to go to the hospital when they are sick and get early treatment. The health system was not properly structured, but with this Ebola outbreak it is better, and the message is telling us to go to hospital when you sick, but though some people have that negative idea of traditional beliefs, not considering the hospital as a better place to go with some illnesses, illnesses like “fankay (witch gun), “gbhagbaha”(blockage), because all this sickness has the same signs and symptoms like malaria, typhoid and other sicknesses, , so people may prefer to go the traditional healers when they had this kind of illness instead of hospital, but to me personally, when I am sick, I prefer the hospital to traditional healers”.

R2: “Some of our people in the villages do not believe Ebola exists and with these thoughts, they will never go to the hospital when they are sick, but rather they will go to the traditional healer, and some people were afraid of the ambulance due to the spraying of the chlorine”.

R3: “The message is telling the people to go the hospital when they are sick and to go with the ambulance when they are sick”.

R4: “Going to the hospital when you are sick is the best and the ambulance is a better means of transportation to the hospital when a person is sick”.

R5: “It is the same as what my colleagues have said, hospital and ambulance are the best”.

R6: “Going to the hospital when you are sick, and going with the ambulance is good, but some people are still stubborn, they will tell them all these messages but their beliefs of going to traditional healers may hardly change”.

R7: “I am happy for the message, I hope people may take it seriously”.

R8: “The message is true, clear and understandable, but to change the perceptions of some people is very difficult”.

M: Is there anything about this message people may not like?

R1: “Well the thoughts of human being are very different, some people may prefer hospital, as it is the only place they can diagnose the kind of sickness they have, but really some people may like everything about the message, though the perceptions are different, they will prefer going to the traditional healer, they don’t believe going to hospital”.

M: But is there anything about this message you think people may not like?

R2: “Yes, there is something about the message people do not like, the emphasis they are making, “when you are sick, don’t be afraid to go to the hospital, because before now, people had in mind that when they go to the hospital, they may diagnose sickness, that they do not have or they will tested positive about Ebola, that led to the fear of people not going to the hospital, and also within that period, the nurses were all afraid to come closer to you, fearing not to contact Ebola, due to these thoughts people may not have confidence in this message, but I am advising other people to the other people, because is the best option ”.

M: Yes sir?

R3: “I think there is nothing wrong with the message, people may like the message”.

R4: “Well, some people may like it and some may not, because their perceptions are very different”.

R5: “Well for people to accept this message, government should improve on all the services in the hospital”.

R6: “As for me there is no problem with the message, it is clear and preaches the right information, because, before now people were reluctant towards the treatment in the hospital or going to the hospital”.

R7: “Well, as my colleagues have said, the message is good”.

R8: “The message is well understood, but for me, I don’t go to hospital when I am sick”.

M: Why?

R8: Because I do not just believe in going to the hospital, I believe in the traditional medicine”.

M: Is this message acceptable in your community?

R1: “Well some people may accept”

M: Why some people may accept?

R1: “Because we Sierra Leonean viewed hospital as a place where you spend a huge amount of money on medication”.

M: But now the treatment at the hospital for Ebola is free at the hospital, so what do you think of this message as treatment the hospital is free?

R1: “Well, some messages about Ebola is conflicting, some messages says Ebola has no specific treatment and some say it has treatment, so with this conflicting messages, if you tell someone to go hospital, when he/she is sick, will that person go to the hospital, so let’s just critically watch into that”.

M: Ok, yes sir, will this message be acceptable in this community?

R2: “Well yes, just like what my brother had said, in this community majority of the people may accept this message, because we have made it as a bye law, that when a person is sick, the person must go to the hospital to seek medical attention, and every day we sensitize our community people about it and we do house to house search for sick people, well only few people may not accept the message, but majority will accept”.

R3: “Yes, majority of the people may accept this message, because our community since our bitter experience about Ebola, we do not tolerate people to stay at home, when they are sick, either they like it or not we will force them to the hospital”.

M: Yes my brother?

R4: “Yes people, will accept it”.

R5: “yes”

M: Ok?

R6: “The message will be accepted, because it is clear and it is direct to the point”.

R7: “Well, I personally accept this message, I do not know for the others, because some people are stubborn in acceptance”

M: Why?

R7: “Some people strongly believe in traditional medicines”.

R8: “It will be accepted by almost 90% of the people in this community, because they do not want to die and they said early treatment at the hospital saves life”.

M: This message addresses misconceptions and worries about the health care system. But do you think this message is clear?

R1: “To me this message is not clear”.

M: What is not clear about the message?

R1: “Because at first they said Ebola do not have treatment, so if Ebola does not have treatment, why are they telling people to go the hospital when we they are sick, will people be convinced to go to the hospital with these kind of thoughts that Ebola is not treatable or people may think when they go to the hospital they will diagnosis other sickness which they do not have. So definitely they will not go to the hospital when they are sick, this message is just building confidence in the people about the health care system, but to me personally the message is not clear”.

M: You said at first, what do you mean?

R1: “I mean, when the Ebola outbreak started”.

M: So from that time to now, you don’t think there is improvement in the health care system?

R1: “Well, there is improvement, because the World Health Organization are helping in the fight and I heard of vaccines and other non-governmental organizations are sponsoring financially to help fight the Ebola diseases”.

M: ok, is there any improvement now?

R1: “Yes”.

M: Ok. What really I want to know, your own perceptions in viewing the message, either it is clear or not

R1: “Well what is don’t clear, is the word “nor fraid” (don’t be afraid)”.

M: Why it is not clear?

R1: “Because by using these words, implies something may or may not happen, because if they said Ebola is a deadly disease and they said don’t be afraid, they create doubts in the minds of the people, they should have use “Go to the hospital you will treated instead of saying don’t be afraid”. There was a case in this community, where they took a family of three to the hospital, only one of them three returned, the others up till now, we don’t know their way about, so what all this is stories, people still have wrong perceptions about going to the hospital, now the message is saying don’t be afraid, it means there is a risk at the hospital, but you have to go and they have said again the Ebola had started showing new signs and symptoms, we are totally confused, the Ebola that was in Congo, is not the Ebola we have in Sierra Leone”.

M: What are the different signs and symptoms?

R1: “At first they said, you hose blood from the nose”

M: You said we have new signs and symptoms, so what are they?

R1: “I heard it through television, that the Ebola had changed it form totally and entirely”.

M: Can you give examples of this signs and symptoms?

R1: At first, they were insisting on don’t touch, but now, people are touching and not avoiding body contact”.

M: Well you spoke about the change of signs and symptoms; I want you to give me Examples of these signs and symptoms that had change?

R1: “Well it was not clear and confirmed to me, they just said the signs and symptoms have change but they do not tell us the new signs and symptoms”.

R2: “Well, all what my colleagues had said it is true, because if you look at the word “don’t be afraid” when fell sick to go the hospital, it is scary, instead they should have said “Go to the hospital when you are sick, they will treat you fine”, because “nor fraid”(don’t be afraid)” implies plenty and different perceptions. The beginning of this outbreak, the facilities and some medical personnel, create fear, so with all this things, they are still afraid, so by using the words “don’t be afraid, is not good”.

R3: At one time, they took blood samples to South Africa, we saw that through a television programme called “wae nus”, so with all this people are still afraid to go the hospital, so don’t be afraid, must be removed from the message”.

R4: “Yes, just as my colleague have said, they should remove the “Don’t be afraid” in the office”.

R5: “The message is well clear and readable”.

R6: “Well to me, I do not have problem with the message it is clear, but a little changes should be done, they should remove, “don’t be afraid”.

R7: “It is clear to me, but I don’t know for the other people”.

R8: “I am in line with my colleagues”.

M: Ok, you said they should change the message, from” when you are sick, don’t be afraid to go to the hospital, you will be treated well” to “When you are sick, go to the hospital, they will treat you fine”.

Rs: “Yes”.

M: OK, do you think people in this community will change their belief and behaviour after hearing/ reading/ seeing this message?

R1: “Well if the system changes its own behaviour”.

M: What do you mean by system?

R1: “I mean the health system, if they change their method of handling things and change their behaviours towards the people, the people will have confidence that when the fell sick, they will go to the hospital without fear, but if they still continue to put on negative behaviour, and I don’t think this message will work”.

M: What do you mean by negative behaviours?

R1: “For example, the taking of Ebola blood sample from Sierra Leone to South Africa, and if people are hearing different things about the health system, there will be no confidence, but if the system changes, the perceptions of people will change”.

R2: “Yes their beliefs and behaviours will change”.

R3: “Well with properly explanations and understanding of this message, the belief and behaviours will change”.

R4: “Some people are stubborn, they will not change, they have fixed mind”.

R5: “It will change their beliefs and behaviour from going to the traditional healers to hospital”.

R6: “Well it is really fine and changes their behaviours and beliefs”.

M: Yes My brother?

R7: “Well for me, I don’t have nothing to say”.

M: Ok, why?

R7: “Don’t just have nothing to say about this,

M: Ok. Yes sir?

R8: “Really the message will change their behaviours and belief”.

M: What do you suggest as the best way to get this message?

R1: “By community sensitization, let them use the youth’s groups to pass on the message, people of the same age, the community people, people will listen to them greatly instead of government, people living in the same community may listen to Each other each other, they should not use people that are not in that community, let the parents in that community see their children, relatives involved in passing on the message”.

M: Ok, Which channel will be used?

R2: “As my colleague said, community sensitization is the best way, they have done it here and some organisations are still doing it, this bring people together, as involving of youths of the same age and community people, the message will go down well”.

M: Ok?

R3: “I prefer radio, newspapers”.

M: Ok?

R4: “Posters and hang bill will be the best”.

R5: “It will be best transmitted through jingle”.

R6: “As my colleagues said, community sensitization is the best, because the community should be part of any development in their community”.

R7: “Radio and television, whatsapp”.

M: Ok?

R8: “In mosques and churches, by the imams and pastors in their sermons, as people listen more to their religious head”.

M: What do you think of the following messengers for these messages?

1. Traditional healers
2. Mami queen
3. Youth role model/music star
4. Ebola survivors
5. Youth groups?

R1: “Well, as my brothers rightly said, all the list of people are not do it better, compared to the youth groups, or community members, university students, traditional healers cannot pass this message better, if you go to the traditional healers now, they only need money, they will not do it because, they also need customers, so if you tell them now to give the people message, it will not be properly and seriously disseminated”.

M: Ok, it may not be proper and serious, if this message is to be disseminated by traditional healers?

R1: “Yes, and also if they do a whole documentary of different messages and happenings of Ebola, it will be fine, play it to all facet of people in this country, the message will be understood and it may be better ad reached every person in Sierra Leone”.

M: What about the Mami queens, to be used as messengers for this message?

R2: “Well I will not recommend the dissemination this message by the mami queen, because, they will politicize it, when they are given tasks, they do it on political bases, and with that the opposition party will not take the message seriously, so it is not proper, if they give the mami queens this message to disseminate”.

R3: “The mami queens are not good messengers to disseminate this message, because they will do it based on sentiments, and their subjects may not take it seriously, because some of these mami queens are totally corrupt in dispensations of their duties, I recommend the chairmen of the youth groups to be the messengers”.

M: Ok, you mentioned youths, so what about using youth role model or music stars as messengers?

R3: “That will be fine, musician like Kao Denero, Nasser Ayoub, and Big Joe will be good messengers for disseminating this message, because with the love the people had for them, the people will listen to them”.

R4: “I prefer using the score board at the national stadium, before a start of any international match, they will first play either the Ebola message video or jingle, so that the spectators will see”.

M: ok?

R5: “For me the youth groups are the best messenger of this message.

M: Why?

R5: “Because most of the laws are violated by youths, so if this message is given to them, they will take it very serious and it will disseminated well, they themselves, will not go against the Rule and they will work by what the message says”.

M: Ok?

R6: “Just the same as my brother have said, the youths are the preferred messengers of this message”.

M: Yes?

R7: “The music stars are the best messengers of this message”.

M: Why?

R7: “Because they had a lot of fans, so their fans may support and listen to any message they give out”.

R8: “Youths are the better people to disseminate this message, especially in their communities that they live, as most of the youths are very hard working within this Ebola outbreak, so with this, people will listen to them”.

M: OK, so what about the survivors, are they not a good messengers to this message?

R1: “Well the Ebola survivors may be good messengers of this message, having survived the Ebola sick, and had respect and believe this Ebola sick is real, in disseminating this message, they will share their experience and telling the people the importance of going to the hospital when you are sick, as survivor, the community people may look upon him as hero, so when passing the message, they will take it very serious”.

M: What about the youth groups?

R2: “Well some areas have a lot of youths, so I am advising that they used the social clubs, youth socialization is very good, and like in our community we have a social clubs, this community social clubs, let use the social clubs as messengers to disseminate this message, because all the members of this social clubs are all youths, so if they are given this tasks, it will be proper done

M: OK?

R3: “The youth groups are a very good members, because they have the enthusiasm to disseminate this message, and by seeing those passing on the message will encourage the other youths to put into practice the message”.

R4: “Well the youth groups as my brother have said”.

M: Yes my brother?

R5: “Well looking at the youths groups, hence they are paid properly, they will be good messengers of this messages”.

M: What are they paying?

R6: “Token for being messengers of this message that serves as motivation to do the work”.

M: Yes, brother, I want you to give your view?

R7: “I don’t have nothing to say, my brothers have said it all”.

M: OK, What do you think of the following channels of this message

1. Jingle(radio and loudspeakers)
2. Radio discussions
3. Community meetings
4. Posters and where do you this posters should be posted
5. Who should distribute this posters?

R1: “Well the jingles are fine, but they have to also use the disk jockey in the disseminating this message, because most of the communities have their own disk jockey, like in this our community, we have one Disk Jockey, that wake us up in the morning, he plays jingle and songs of Ebola in the morning, and at this time, the message should be well disseminated”.

M: Ok?

R2: “Well for me I prefer we make use of all the works of life as channels or messengers in this message dissemination because this Ebola had seriously crippled everybody, and most people are sitting down without job”.

M: Ok, what about radio discussions?

Rs: “It is a good way of passing this message?

M: Why?

R1: “Because seventy percent of people now listened to radio programmes”.

M: Ok, What about community meetings?

Rs: “It is also good”.

R1: “In fact let me say something on this, use the churches, the mosques and the community and also jingle, because young people listen more to jingle and some jingles are serves as slogan, which the children we will saying it every moment and it helps disseminate the message”.

M: Ok, What about posters?

R1: “They are also good”.

R2: “Because 60% of the populations of this country read and understand posters”.

R3: “Well some people may tore the posters, because of their wrong perceptions about Ebola, or some people tear it, without any reason”.

M: Where do you think this posters should be posted?

R4: “On electric poles”.

R5: “It should be posted also where people passes frequently”.

R6: “At attaya base, where most of the youths gathered, to drink attaya”.

M: Ok?

R6: “I suggest they also use banners to print this message on and place it where people frequently pass”.

M: OK.

R7: “You spoke about where these posters should be posted, well, I suggest, health centres, court Barry in each community and also the houses of chiefs, notice boards of schools or other learning institutions, because all this place are visited by a greater population of people Every day”.

M: Ok, who should distribute these posters and how?

R1: “I advise they give the young people”.

M: Why the young people?

R1: “Because they are jobless right now”.

M: How will they distribute the posters?

R1: “Before they give them the posters to distribute, get a workshop and teach them the procedures and they will have the know-how”.

M: OK, yes sir, do you have anything to say?

R2: “No”.

R3: “it should be distributed electronically”.

M: How?

R3: “It should be done by mobile companies, sending it as texts or pictures in the phone and also using social media like Facebook, whatsapp, because younger people like social media, they will see and read it”

**(NOTE: Topic 18 –Denial – “No matta di sick, call 117”** )

M: Ok, let look at this other message “No matter di sick call 117” what do you really think of this message?

R1: “Well the message sound good, but the perceptions of the sick person is quite different, because some people are coming from traditional homes, they will not just fell sick and call 117 or even go to the hospital, they have another channels that is used to investigate the sickness they have. The message is saying, no matter the sick, call 117, I am not in line with this message, I will not just fall sick and call 117, I have to go the hospital first, instead of calling 117, I don’t know if the sickness is Ebola or not, I prefer, the message go like this “no matta the sick, go to the hospital”, the hospital will test and diagnosis my sickness, if it is Ebola, they will call 117, the symptoms of malaria is similar to the Ebola, so I will not just call 117, when I fell sick”.

R2: “Like what my colleague said “no matter the sick call 117, not all sickness is Ebola, some sickness are traditional sickness, sickness like “fankay”, Witch gun, with these sickness, you will not call 117 or go the hospital, because, they are not hospital sickness, traditional healers heal this type of sickness, so it is not necessary to call 117, if you have illness like this”.

M: Yes sir, what are your thoughts about this message?

R3: “Yes, on Wednesday I went to my boss, so I fell sick and started vomiting and the wife of my boss, said let them call 117, my boss said no, we should not call 117”.

M: Why he said they should not call 117?

R3: “Because this is my normal illness, it is within our family, so they later took me to the hospital and test my temperature, they said it is not Ebola, so in this message, you have to remove “no matter”, because not every sickness is Ebola”.

M: We have to remove the “no matter”?

R3: “Yes”.

M: Ok

R4: “I don’t like this particular message, because it is similar to the other message which say don’t be afraid to go the hospital, the 117 phone line works, but there is fear attached to them, they said no matter the sick call 117, so when some get insane, we should call 117, let them say, when you are sick, go to the hospital, I don’t even like calling 117”.

M: Why?

R4: “Because it is fearful, anything they measure the name 117, because at first when they call 117 and they come for you and take you to the treatment centre, you will not come back”.

R5: “Well in the message, “no matter” is very harsh, so it they need to remove it in the message”.

M: Yes my brother?

R6: “I am joining lines with my brothers, that the “no matter” should be remove from the message”.

M: Ok?

R7: “Well the message is good, I don’t see nothing bad about the message, because, the message is saying no matter the sick, call 117, in Sierra Leone, we have other sickness that kills, and 117 does not only address Ebola sick”.

M: Ok?

R8: “Well the message is good, and as my brother just said, “No matter the sick call 117”.

M: Ok, you said you don’t like this message, what is the message like you want instead of this?

R1: “Well, the message should be “if you are sick, go to the hospital”.

M: Ok?

R2: “The message should be, when you are sick, call 117”.

M: Ok, yes sir?

R3: “Like for me, I don’t want them even to involve 117, 117 is creating fright in people”.

M: Ok?

R4: “For me the message should be, when you are sick, go to the hospital, the hospital will call 117”

M: OK, yes sir?

R5: “Well, if they are telling us to call 117, some people may not even call 117, because they are afraid, so they have to say when you are sick, go to the hospital”.

M: Ok?

R6: “Well, when you call 117, the ambulance will come for, and when the ambulance comes, it creates stigma and panic within the area, so it is not necessary to call 117, when you fall sick, go to the hospital, you will be treated”.

M: Ok, yes sir?

R7: “Well, the message should remain as it is, no matter the sick, call 117”.

M: Ok?

R8: “As I said the message is good, there is nothing wrong with the message,

M: Ok, Will the people accept this message?

R1: “The community will not accept this message, because people have different perceptions, some of the people will perceived it different, as people were not happy with the 117,and they believe in traditional healers instead of calling 117 or the hospital”.

M: Ok, yes sir?

R2: “Some people will not accept this message because people have in mind that, not all sickness is Ebola, so they will not accept the message, like we had a case, when a boy got insane, they do not call 117, and they took the boy to a pastor in a church, most of the people in this community are educated, so they will not ascribe to this message”.

M: Ok, the message you said, that when you are sick, go to the hospital, will people accept this message?

R2: “Yes”.

M: Ok, yes sir?

R3: “Some people may accept and others may not, because the perceptions are different”.

M: Ok, yes sir?

R4: “They will accept the message, because the medical experts has said it,

M: Ok, the message encourages community members to report any signs and symptoms to 117, because to the differentiate Ebola and others, only the medical experts can do it, do you think this message is clear?

R1: “No, it is not clear”.

M: Why?

R1: “Because it creates dispute between neighbours, not all illness is Ebola, like for instance if someone become insane and you call 117, with the negative perceptions, that people will think his or her neighbour do not like the him, so the message is not clear, because the statement, no matter, the sick call 117, the “no matter” should be removed”.

M: “Yes sir?

R2: Well, I don’t see it necessary to call 117, because 117 does not have any good name in this community, so for the message to be, clear, I prefer, the message be like this “If you are sick call the medical experts, or go to the hospital, most of the people that die in this community during this outbreak, they call 117 and ambulance came and took them to the treatment centre and they never came back, so 117 and “no matter” must be out of the message”.

M: So you think this message will not change the perceptions of the people?

R1: “At all, it will not, once their minds are fixed, they will not change”.

M: No matter the sick, call 117, will other communities accept this message?

R2: “Well, the perceptions are different, some other people may accept this message and other may not, we in this community will not accept but other communities will accept this message”.

M: OK?

R3: “For me, I am generalizing it, any community you take this message, people will not accept it at all, 117 phone line creates stigmatization anytime you call 117, they connect you with the ambulance and the ambulance creates serious panic when they come for the sick person, so this message needs to be rephrased, as “When you are sick, go to the hospital, the hospital will call 117”.

M: OK, so if they use this message that you have suggested, will that change the belief of the people?

R3: “Well some people may change their perceptions, not all, likely 50% of the population”.

M: Why do you say some people but not all?

R3: “Some people are not financially strong to go to the hospital”.

M: But Ebola treatment is free?

R3: “Even it is free, some people are afraid to go to the hospital”.

M: Why they are afraid?

R3: “Because we have Ebola in this country, and there is perception that when you are sick and go to the hospital, you will not survive”.

M: Ok, the message you suggested that, “when you are sick, you have to go to the hospital” What do you suggest as the best way to get this message out?

R1: “Let them use the youth groups to disseminate this message, empower the youths they will do it perfectly most especially the youths in a particular community, not youths from another community”.

M: Ok?

R2: “Well, I agreed with my brother, the youths are good messengers of this message, Ebola is not a sickness that we do away with entirely, so let the youths be permanent messenger of messages as using their energy to move from place to place the message will go across”.

R3: “Well, I prefer the community leaders, as their subjects listen more to them”.

M: Ok, what about you sir?

R4: “The youth groups”.

M; Ok?

R5: “For me, radio is the best for this message to reach the people”.

R6: “Town crier in village with loud speakers”,

R7: “The youths, as my brother said”.

M: Ok?

R8: “Through text messages”.

M: What do you think of the following messengers for this message, a. any female member from a community, b. music artist and c. chief?

R1: “Well involving female members is very nice, because nowadays female motivate the male and make them grow more interest in whatever they are part of, for instance, they said the strength of a woman, women are driving force to men, so it good for the female members to be messengers”.

M: What about the music artist?

R1: “Well, they are also good messengers, because with the love from their fans, they will adhere to anything they say, so they are good messengers, for instance, if artist x talk to his or her fans they will listen and adhere, likes artist y”.

M: Ok?

R2: “Well, as my brothers said, they are good messengers, because some popular artists have a lot of fans, so they will listen to them”.

M: Ok, yes sir?

R3: “They are good messengers, and these music artists are used to make shows and within the shows, they sensitize their fans, like Nasser Ayoub, Kao Denero, and LAJ”.

R4: “They are good messengers”.

M: Ok, What about the chiefs?

R5: “Well I prefer chiefs in the up country, like Makeni, Kenema, not in Freetown, because in Freetown, people do not have respect for chiefs, they believe in magistrate courts, but in the provinces they have respect for their chiefs and their subjects listen to them, so they are good messengers in the provinces but not in Freetown”.

R6: “The chiefs are bias, they only take what the centre Government tell them, so I don’t take them as good messengers, is better you, they use the pastors, imams, youth groups and the artist, as messengers of this message”.

M: Ok, What do you think about the following channels about this message like jingle (radio and loud speaker on car), whatsapp/ sms, Town crier, button/badge or wristband, Posters?

R1: “Well I prefer we use the social media like Facebook, whatsapp or sms”.

M: Ok?

R2: “I prefer the wrist bands, because it appears colourful and most people may like to use the bands”.

R3: “I prefer jingle and radio and loud speakers, because with the message will reach most of the people, even in this community they use loud speakers on car to sensitize”.

R4: “I prefer social media, like whatsapp, Facebook, as a lot of youths use these social Media”.

M: Ok, my brother,

R5: “Well, just as my brother as said, social media”.

M: Ok?

R6: “loud speakers on car helps a lot in sensitizing us during the early times of this outbreak, so I prefer loud speakers on cars as a best channel of this message”.

M: Ok, What about the town criers?

R7: “They are only important in the provinces not in Freetown”.

M: Ok,

R8: “Well for us in this community, we have a radio we call bush radio, it wake us early in the morning at 5:00 am, this is a collective efforts of the youths in this community since this outbreak, they have megaphones they use, they go right round the community, so we refer to them as bush radio”.

M: Ok, What about the buttons, badges and wrist band?

R1: “They are very important and are good channels,

M: Where do you think these posters and badges/wristbands, should be posted or distributed?

R1: “Well, they should be posted where people gather like cinema, attaya base”.

R2: “Well, for me, I prefer junctions, Electric poles”.

M: Ok, yes my brother?

R3: “Where youths gather to play mercury lottery”.

R4: “Churches and mosque will be better place to post the posters”

M: Ok, Who should distribute the posters or badges, or Wristband?

R1: “Pastors and imams, and also the youths”.

R2: “A female member in our community, as women are the good in moving men to support”.

R3: “The person himself that comes with the posters, badges and wrist bands, will do the distributions to avoid chaos”.

M: Ok, What about you sir?

R4: “Same as what my brother said, female member in the community”.

R5: “Music, artist, football stars like, Mohamed kallon, Kei Kamara”.

M: Ok?

R6: “The chiefs in the provinces and the youth leaders”.

R7: “The survivors and other prominent people in the community”.

R8: “I want to suggest that, you also print T-shirts, the people may appreciate it”.

**(NOTE: Topic 19 – Health care system - worries – ”If you go hospital quick chance de say yu go well” )**

M: Ok, I have another message which says “if you go hospital quick chance de say yu go well”, what do you think of this message?

R1: “it is fine”.

M: What is fine about the message?

R2: “I believe and trust this message, when you are sick and go to the hospital early, your chance of survival will increase, but if you stays home and allow the sickness to overcome you, your chance of survival will become less, they said the early the better”.

M: Ok, look at this picture and explain to me your own understanding?

R1: “Well the picture shows an Ebola survivor showing his certificate, and telling the people that when you are sick and you go for early treatment, you will survive”.

M: Ok, so that is what the picture mean?

Rs “Yes”.

M: We are trying to develop a poster, the message on the photo, is saying, when you are sick, if you go hospital early, you have chance to survive, what do you think of this message?

R2: “Well, there is an adage which says, the early the better, if you go early to the hospital, when you are sick, the chance of survival increases, but if you stays at home, it is a problem for you”.

M: Is there anything about this message that you think, people may not like, like the colour, the krio?

R1: “The message is good and clear, I personally like the message, and likewise other people may like it as well”.

R2: “The picture is good, colours are well fitted in, and it is good”.

R3: “For me the conditional word, if and the chance, makes the message more, good and understandable, they chance is 50 is to 50, you are have chance to survive the sick or not and some sickness are by stages, stage one, and two onwards, the more you late for treatment the lesser your chance of survival”.

M: Ok, Which colour do you prefer?

R1: “Black and white”.

R2: “Black and white, no political party colour”.

M: Ok?

R3: “The same as colour as my brother have said”.

R4: “Blue and white”

R5: “For me I prefer any brighter colour, but not red and green (*note: colours of government party and main opposition party*)”.

R6 “Black and white”.

M: Ok, yes pa?

R7: “Blue and black, so it appears nice”.

M: Yes Sir?

R8: “Black and white, the reason for this black and white, white symbolizes peace and black for trouble, Ebola is a trouble, so the black represent it and the survival is for white”.

M: Is this message acceptable, in your community?

R1: “Yes, because the message is clear and understandable”.

M: ok?

R2: “The message gives encouragement to people, to go to hospital early, so people will accept the message”.

R3: “They will accept it, and put it into practice”.

M: this message is encouraging people to the hospital, early and not be afraid of the hospitals, but is this message clear?

R1: “Yes, it is clear”.

M: Ok?

R2: “Yes”.

M: Ok, yes my brother?

R3: “Just as my colleagues have said, this message is clear”.

M: ok, is there anything of the message that is not clear?

R1: “No, it is clear”.

M: Ok?

R2: “There is no problem with the message, it is clear”.

R3: “No. the message is clear, there is no problem”.

M: Ok, do you think people in this community and other communities, will change their belief, after hearing/ reading, /seeing this message?

R1: “Yes, their beliefs and behaviour will complete change from the negative aspect to positive towards treatment at the hospital”.

M: Ok?

R2: “Yes, their belief will change”.

M: what do you suggest as the best way to get this message out?

R1: “Radio, Youths”.

M: Ok?

R2: “Female music stars, the town criers in the villages, and also jingles”.

M: What do you think about the following messengers for this message, health workers, or survivors?

R1: “Well I prefer the survivors, because people may listen more to the survivors than the health workers, because the survivors are successful warriors from Ebola war”.

M: Ok, what channel do we prefer?

R1: “Radio, whatsapp, television, poster, just like our father said”.

M: Ok?

R2: “Pinups and badges, buttons are a good channels of disseminating this message”.

M: Ok, Yes my brother?

R3: “Posters are very essential in giving out this message

R4: “Ebola survivors are very importance in giving out this message”.

R5: “I want to also emphasized the use of women as messenger, because there is an adage which say when you educate a woman, you have educated a generation, the women will give the message to her children and also other family members

M: What do you think about the poster, where do you think these posters should be posted and who should distribute the posters?

R1: “Well the posters are fine and they should be posted on schools notice boards, mosques, churches and public places, community centres”.

M: Ok?

R2: “These places are very secure, when the posters are posted there, they will not tear them”.

**(NOTE: Topic 28 – Stigma –Health care workers – “Nurse Susan na hero – E hep plenty Ebola survivor dem fo liv” en ”Di Ebola workman den na i champion den”)**

M: Ok, Look at this picture and tell me what you think of it?

R1: “Well, on this poster, you have a medical person, and some family members standing”.

M: Is this medical person a woman or man?

R2: “A woman”.

M: When you see this picture, what comes into your mind?

R3: “What comes into my mind is to live as one family”.

M: Ok, yes sir?

R4: “Well, the picture is depicting, to fight Ebola, we must be a united family”.

M: Ok, we are trying to develop a poster, so look at the poster and texts, the texts reads, “nurse Susan is a hero, she help plenty Ebola survivors to live”, what do you think about this?

R1: “Well, it is good, because Nurse Susan as a hero sacrifices her life for other people to live, though as a medical practitioner it is your duty and onus to risk yourself in saving the lives of other”.

M: Ok?

R2: “They said unity is strength, so the medical people and the community people are working together to fight Ebola out of Sierra Leone”.

M: Ok, is there anything about this message that you think people, may not like?

R3: “No”

M: Why?

R3: “Because everybody in Sierra Leone is well aware of the fact that, the work of medical practitioners is very great and saving lives is not easy, so they will accept this message”.

M: What about the colour?

R4: “Well the colour is good”.

M: Ok, what colour should be used?

R4: “Black and white, Nurse Susan should be given a white colour and the other people around her black, blue”.

R5: “Black and white is a nice colour, they should use it”.

M: Ok, yes sir?

R6: “The same colour as my brother said”.

M: Ok?

R7: “Well, I prefer blue, white and a little mauve”.

M: Ok?

R8: “If they use green, red, yellow and white”.

M: Yes sir?

R1: I just want to make an observation, nurse Susan is a hero I agreed, but why only a nurse Susan, we don’t have male Nurses to be use also, because in our community, people are very controversial, they will must ask this question, so I suggest let there be another picture or poster with a male nurse as a hero standing among people, because this Ebola fight was fought by both male and female medical practitioners”.

M: Ok, this is just a sample, but it will be noted. Is this message acceptable in your community?

R2: “Yes, because it is not easy to save life, and the community people knew the efforts and sacrifices of the medical people have done to save others’ lives”.

M: Ok, this message is addressing stigma of people working in Ebola response (HCWs, burial team members, ambulance staff, staffs at the treatment centre and holding centres), what do you think of this message?

R1: “They will accept this message, though people have different perceptions, but I guess they will accept it”.

M: Ok, do you think this message is clear?

R1: “It is clear, because risking your own life for others is not easy, the message is clear and people will understand it”.

M: Ok, do you think people in this community and other communities will change their belief and behaviour after hearing/ reading/ seeing this message?

R1: “Well, some people beliefs and behaviour will be changed but not all the people, because the perceptions of people are different”.

R2: “Well yes, the message will change the belief and attitude of people, Nurse Susan has risked her life to save people, I knew some nurses since this Ebola outbreak, they stopped working at all, so people will accept this message because of nurse Susan’s sacrifice in the Ebola fight”.

M: What do suggest as the best way to get this message?

R1: “The same as you we told you early”

R2: “The same as my brother just said”.

M: What do you think of the following messengers for this message, a. people involved in the Ebola response?

R1: “Yes, it will be good for them to be messengers because they have helped the people greatly and they will accept them as messengers”.

M: Ok?

R2: “Well I will not 100% support that they will accept them as good messengers, because some people may say, the nurses or medical people help in the killing of most the people during the outbreak, so they will perceived it on that basis, but on a whole, the majority people will appreciate the message”.

M: Ok?

R3: “Yes, if you use them on posters, the people will accept it”.

M: Ok?

R4: “The same as my brothers said”.

M: What do you think of the channels and where these posters should be posted?

R1: “The same place we told you early”.

R2: “The same as brother just same”.

M: Ok, but you don’t think of any new place?

R3: “No”.

**(NOTE: Topic 32 – Fear of ambulance - “For trus di ambulance” en “Di ambulance na di best en safe way fo go hospital” en “Di healthcare wokman den frienly, den go hol yu fine” en “Fine breeze de blow insai di ambulance”)**

M: What do you think of this message “For trus di ambulance en di ambulance na di best en safe way fo go hospital en di healthcare wokman den friendly, den go holy u fine en “finebreeze de blow insai di ambulance”

R1: “Well, the message is clear, because when you have call 117, the ambulance will come and when the ambulance comes, there is no options but to go with the ambulance and so you have to only trust the ambulance, but for me, I don’t trust the ambulance”.

M: Why?

R1: “Because of the chlorine they spray, and the speed of the ambulance is high”.

M: Ok?

R2: “In some situations, you just have to trust the ambulance, because there is no option, because when you are sick of Ebola, the best option to go to the hospital is with ambulance, so unless you trust the ambulance”.

M: Ok, but what this message is talking about?

R2: “That we have to trust the ambulance as a safest means of transport to the hospital”.

M: Ok?

R3: “Well the message to my understanding is, you should not board public transport, when you think, you may have Ebola, because if you do, you may infect other people and we want to break the chain of transmission, so the best means of transportation to the hospital, is the ambulance”.

M: Is this message acceptable in your community?

R1: “Yes, they will accept it, just like what my brother said, the ambulance is the safest and comfortable means to go to the hospital when you fall sick”.

M: Yes sir?

R2: “My own suggestions here, the use of the ambulance, in the message, the word trust as already created doubts so I suggest we remove the trust and say go with the ambulance that will be fine”.

R3: “Just as my father as said , when they use trust, it means something bad have been happening, it creates doubts, so the trust should be removed and we say, go with the ambulance”.

M: Ok?

R4: “What I want to suggest, let the medical people be using different ambulance for different case, let there be one ambulance for Ebola positive and one for suspected cases, and one for other illness, this will be good and increase the people’s confidence”.

M: Ok, we just want to increase trust in the ambulance as a safe means of transport to the hospital with friendly workers, but you said this message is not clear?

R1: “Yes, the word that is in the message, “trust” creates doubt in the mind”.

M: Ok, but you think the ambulance is best transport to the hospital?

R2: “Yes”.

M: Why?

R2: “Because there is no option but to go with the ambulance and other vehicles gives way to the ambulance, this make the movement of the ambulance faster to the hospital and they also administer treatment in the ambulance, which does not happen with public transport ”.

M: So what is the message will you suggest that we use?

R2: “I suggest you use “Go with the ambulance is the safest means of transportation to the hospital”.

M: So you don’t like the word “trust” in the message?

R2: “Yes”

M: Why?

R2: “Because in any message there is trust, there is doubt, so let remove the trust”.

M: So if they use the message you said, will that change the perfection of the people?

R2: “Well some people not all”.

M: Why not all the people?

R2: “Because people have different perception, your own perception and mine is very different”.

M: Ok, who do you think should give this message?

R1: “Well, the nurses, survivors, music stars”.

M: Ok?

R2: “Just the same as my brother said”.

M: Ok?

R3: “Social media, radio and television”.

M: Ok, what do you think of the following messenger for this message: a. religious leaders b. youth leaders c. women’s leaders?

R3: “they are all good messengers of this message, because they play a vital role in the community, so people listen to them and adhere to what they say”.

M: Ok, what do you think of the following channels for this message, community meeting, sermon and radio discussions?

R1: “They are very good channels, and by using them the message reaches majority of the people”.

M: Ok?

R2: “These channels are very good, with the sermon, since it is coming from their religious head, people will take it as a good one”.

M: Ok, I thank you very much for given me your attentions and time.
