# Supplementary material for: Development of a set of community-informed Ebola messages for Sierra Leone
Source: PLoS Negl Trop Dis. 2017 Aug 7;11(8):e0005742. doi: 10.1371/journal.pntd.0005742 (PMC5560759; doi:10.1371/journal.pntd.0005742)
Supplement: S1 Appendix — (ZIP) [file pntd.0005742.s001.zip › Ebola messages - FGD and interview transcripts/R2HC Ebola Fieldwork 2/R2HC Ebola F2 FGD-Male-Urban2-B.docx]

| CODE | **R2HC Ebola F2 FGD-Male-Urban2-B (**Urban focus group discussion with younger (<25 years) and older (25+) males, using **topic guide GroupB and Picture set B)** |
| --- | --- |
| DATE | March 2015 |
| DURATION (minutes) | 60 |
| Collector nrs | 1 and 4 |
| LANGUAGE INTERVIEW | Krio |

**PERSONAL DATA PARTICIPANTS**

| Nr | Sex  (*F/ M*) | Age  (*in years*) | Education Level (*e.g. none, Primary, secondary, tertiary*) | Language (*e.g. Mende, Temne, Krio)* | Religion | Job / Employment (*how they earn their living e.g. farmer, teacher, trader*) | Role in community  (*e.g. youth leader*)  ANONYMIZED, ONLY AREA OF ROLE INDICATED |
| --- | --- | --- | --- | --- | --- | --- | --- |
| 1 | M | 25 | None | Temne | Muslim | Carpenter | none |
| 2 | M | 23 | Primary | Limba | Christian | Welder | none |
| 3 | M | 21 | Tertiary | Fullah | Muslim | Trader | Youth |
| 4 | M | 24 | None | Koranko | Muslim | None | Health |
| 5 | M | 30 | Tertiary | Mende | Christian | Teaching | Local government |
| 6 | M | 40 | Tertiary | Limba | Muslim | Trader | Local government |
| 7 | M | 36 | Primary | Susu | Muslim | Teacher | Local government |
| 8 | M | 39 | Tertiary | Temne | Muslim | Teacher | None |

**TRANSCRIPT: (M = Moderator, R= respondent, R1= first person responding to a question, DOES NOT correspond to numbering used in Personal Data!)**

**(NOTE: Topic 10/11 – Burial – Poster - ”Safe berrin na free – e no right fo gi or tek moni fo am” )**

M: Let us look at this message “safe berrin na free- e nor right fo gi or tek moni fo am”, what do you think of this message?

R1: “When the outbreak of Ebola starts, the burial teams where asking members of family for money, as a token for them to wrap the dead body with “kansagay”(=white burial cloth), as a respect to the corpse, so the message is telling us to stop given money to the burial team as safe burial is free of cost”.

M: Ok, any other person?

R2: “Well, to my own opinion, people want to maintain their tradition by giving last respect to the dead, but when this outbreak started, there was no washing of the dead, no “kansankay”, people were given money to the burial teams to observe this respect to their love ones, so the message is telling us not to give money again to the burial teams, as safe burial is free of cost”.

M: Is there anything about this message that you think people may not like?

R3: “There is nothing wrong with the message, the people will be happy for this message, because before now people were grumbling about this money transaction between the burial team and the community people, they will accept this message,”

M: Do you think the community will accept this message?

R4: “Yes”.

M: Why?

R4: “Because the message has sensitized them not give money again for safe burial as it is free, even after Ebola, this safe burial will continue”.

R5: “For me, not everybody will be happy, when they have said, we should not give money to the burial team, the reason is, hence they have made this pronouncement publicly, people will stop giving money and the burial team will roughly handle the dead body, because they know, people will not give them money again, if even they ask for it, but thank God now the Red cross have taken over the burial”.

M: Well our reason for this message, is, family members have been offering money to burial teams, and burial teams have been asking for money, we want to make it clear that burials are free, but do you think this message is clear?

R1: “The message is clear”.

R2: “The message is clear

R3: this message is clear is because it will send signal to the burial team that you should not ask neither they allow any favour from the sympathized family, so am happy about this message, this is one of the main reason you see the high rate of infection of Ebola in this country, so if the burial team adhere to this message then we are sure of winning the battle of Ebola .

R4: (=name of moderator) , let me inform you that one of the main reasons for the spread of Ebola is lack of dignified burial and the money that involved, family members would like to see their loved ones given their last respect on planet earth, and therefore they would give secretly money to the burial team just to allow them do it, but those that don’t have the money to give , would prefer to wash the corpse before the arrival of the burial team, so if you come up with this message poorer family will be happy for this message , meaning they will not bother to give a cent to the burial team.

M: any other person wants to give his own opinion?

R5: we don’t want to repeat the same thing, I think my brothers have said all we intend saying.

M: Ok, do you think people in this community and other communities, will change their beliefs?

R1: “Yes, it will change their beliefs, but the only additional thing you have to do, you must monitor the burial teams to ensure they do the right thing, because people still give money for proper care and respect for their love ones who have died”.

R2: “The burial teams need special monitoring for them not to take money for the bereaved family”.

R3: the people will accept this message, this is what the community wants, you guys are the only organisation that has come to test the messages whether they are good or not, trust me if only the burial team accept this message in good faith, I will tell you this will end Ebola.

M: Yes my brother at the corner, what is your take on this?

R: Am really impressed with this kind of message that, the reason is I have been a victim, my aunt died of Ebola , and one of the burial team member met me and told me that man if you want to give your aunt her last respect, then you should give me something and I will talk to the team members not to be at hurry and they will ensure that they will take the corpse with respect , but at that time I don’t have money , and I told the burial team member that for now I don’t have money , but am just appealing to them to please bury my aunt with respect , but he later accepted I don’t know what they did, but if this message comes out , then people will have confidence that government has come to address their concerns, so this message will be accepted to the community , but please you have to train the burial team to be more professional , because if you send this message and the burial team, knowing Sierra Leonean for taking bribes , then the message will be meaningless

M: Who do you think should give this message?

R1: “Well, we have mobilizers, CHW, HFCs, teachers, students in that particular community”.

R2: “I suggest the religious leaders to pass on this message on Fridays and Sundays, but if they use young people to pass on this message, the older people will not take them seriously”.

M: Which channel should we use?

R3: “Well, according to statement from my brother about religious leaders, it is fine, but let me ask this question, how many people usually go to church or Muslim, only few, some I don’t believe if the religious leaders will be the actual people to pass on this message that will reach all the people but if they, use the mobilizers, the media and the religious leaders, that will be fine”.

M: Any other person?

R4: “I want to suggest, in passing on this message, let the religious leaders pass it on to their congregational members, let the youth leaders target the youths, old target the old, teachers and lecturers pass on it on to their students, with this moves, it will be nice and the message will pass on to everyone”.

R5: “Like with my brother was saying, peer groups have more influence and saying over their peers, so if they pass on this message, it will be good, also guys in the ghetto only listen to the head of the ghetto, you may expect them to listen to religious leaders ”.

M: What do you think about the burial team to be messengers of this message, will they be good messengers of this message?

R1: “No”.

R2: “No, they will not be good messengers of this message”.

M: Why?

R3: “Because they have stopped them from requesting money, they would not do it well, because they are not happy and also not all community have a burial team worker”.

M: Ok, why the burial teams are not good messengers of this message?

R3: “Because they already have job to do, and their job will not allow them to pass on this message, but the mobilizers are always available and ready to do the work”.

R4: “Well in my opinion, they are best messengers, because, when they go to collect the corpse, if the bereaved family wants to give them money, they will say no, safe burial is free of cost but 60% of the people in the burial team are doing it in their own selfish-interest, so with this selfish interest, if they be messengers of this message, the purpose of the message will be achieved, because they will not even give out the message”.

M: Ok, what about the family members as messengers?

R1: “Well the family members are the best messengers, as they say, the family is the first agent of socialization, if my mother or father tells me to do or not do certain things, I will obey, so also with this message,, if they tell their family members not give money to burial teams, they will comply with their advice”.

M: Ok. What about one male and one female in the community

R2: “It is also nice, because, it shows the involvement of all categories of people in the message dissemination; they will be good messengers also”.

R3: “To add to what my brother said, in any household, the head of the family is very important, if you give the head of the family to pass on the message, he or she will pass it on to the other family members and they will be listen to him, than other different people coming to them and pass on the message”.

M: What about the town crier?

R1: “Well they are more important in the villages not in big town”.

R2: “They are good messengers but in the villages or provinces, not in Freetown”.

M: Ok?

R3: “The town criers are very good messengers in villages, they move up and down in the village to pass on the message and all the villagers will get the message”.

M: So it is recommended for only villages?

R4: “Yes, I have never seen town criers in Freetown, and in Freetown they don’t have much respect for chiefs or even the town criers”.

M: What do you think of the jingle, radio and louder speakers on car?

R5: “Well yes, that was the first thing in my statement, the media is an important channel to be use in passing on this information or messages to the people, we have our radio stations here, so if they use this station, the message will reach all the people in the community”.

R6: “Yes, for me I prefer the loudspeakers on cars, because, they help us a lot during this outbreak, so they are very important in passing on the message, as they move up and down, so the people will get the message”.

M: Ok, what about the posters and where do you think these posters should be posted?

R1: “Well, the posters are good, and they should be posted at public places, schools, churches and mosque and also places where people gather together like bus halt, Shell Company”.

M: Ok?

R2: “Ok, for me these posters should be posted at ataya base, schools, churches, cinemas, these places people are always in majority”.

M: Anybody has anything to say?

R: we all accept, that is why we don’t want to repeat the same thing over and over again

M: Who should distribute the posters?

R1: “Well, the social mobilizers, and youth groups”.

M: How they will do the distribution?

R1: the distribution be done in the following area, ataya base, community centres, junctions, churches, mosques,

R1: “Well it depends upon how the people may want the distribution to go, so if this messengers are encourage and they give them instructions, they will work by that instruction”.

M: OK?

R2: “Well in we are talking of posters here, not everyone can read or write, so I prefer, you give the mobilizer the message, let them go house to house to pass on the message, they will hold the poster and show the people, and also not everybody has a radio and some people don’t have time to listen to the radio, you have to note that also”.

M: you talked about, it depends on how the people may want the distribution, what does that mean?

R3: I mean the messengers must be given a token to do the distribution,

(NOTE: **Topic 13 – Early treatment and prevention – Jingle)**

M: Ok, let look at this message, it is a jingle, and it reads, “My name is Sallay. I survived the Ebola sick because when I contracted the virus, I did not try to cure myself at home, I went straight to the hospital. That is very true my sister, that is even why we the doctors say the earlier you go to the hospital, the greater your chance of survival. That is the spirit my people, please let us all work together to drive this sickness from our country.”- What do you think of this?

R1: Well, people were reluctant to go to the hospital early, this leads to the death of many people at the beginning, this reluctant attitude of people was happening because of the bad news about the staffs in the hospital, that when you are sick and go to the hospital, the staff, you may not be treated properly, but this message is building our confidence to seek early treatment at the hospital”.

R2: “As my brother said, the same nurse that were telling people to go to the hospital, when they sick, are the same ones that administer treatment to people at home, so how do you expect the people to visit the hospital, but with this message now, they will be visiting the hospital, as people have totally change”.

M: “Ok, but how do you see the message?

R1: “Well, this message should be played as jingle over the radio, and we should contact a person that has public address system, so that, we will be using the public address system in the community to pass on this message”.

M: OK?

R2: “To add on what my brother just said, when this outbreak of Ebola started, the first information they gave to the people about Ebola, created panic and fear in the people, so I blamed the government and World Health Organisation (WHO), for misinforming people, they said Ebola is a dangerous disease that kills, it do not have medicine and do not cure, so with this message made the people to stop going to the hospital and concentrated to the traditional healers, because they know, when they go the hospital, they do not have cure”.

R3: It is a nice message,

M: What makes it nice message?

R3: is the wording which say, we the doctors say, the earlier you go to the hospital, the greater the chance of survival., why I like that wording the doctors too were afraid to treat people, because they have seen a lot of their compatriot, that became sick and died, so if they are calling on people to go early, then we hope they will be there to treat people with respect.

R4: It is a good message, this will encourage people to go to the centre because Sallay has come out of the treatment centre, so also any other person who might be affected might also be cured, so to me it will encourage people to go to the centre.

M: Ok, let us assume that the doctors do take their work seriously, is this message a good message to the people?

R1: “Though the Krio sounds like English and not everybody is educated in this country, but if government put everything in place, the message will work, and people will put it into practice”.

M: Do you think people may accept this message in the community?

R1: “Yes”.

M: Does anyone has anything to say?

R2: “To buttress, what my brother was saying, as long as it is in the jingle form, they will accept the message, if you play the jingle of this message in a particular area, the people in that particular will get the message properly”.

M: Ok? This message wants to discourage home treatment and encourage people to go for treatment early to have a higher chance of survival, but is there anything in this message that is not correct?

R3: “Well no, but let them just try to implement ways and disseminate the message, it will work?

M: What do you mean by implement?

R4: “Well, it is a suggestion, when you test the message, then you can see, if it can works”. Now you have come to us to test the message, while we have accepted we must see these message out, that is what I meant by implementing what you have done.

M: Ok, That is the main reason we are here asking on your opinion about this message, but how do you see the message?

R4: Let me add to what my brother, the health workers do not treat patient properly, so with these concepts, it creates fears in the minds of the people, so, they are always afraid to go the hospital, but with this message, they will definitely go to the hospital, because early treatment saves life”.

R5: “This message is good, and very perfect, but there should be another message again that will sensitize the health workers, to be treating patient with care, so this will increase their confidence of going to the hospital but if they are not treating them properly, the message will not make any impact, because with all effort of disseminating the message people, people may not put it into practice”.

R6: “Let us don’t blame the doctors, they also have life, when this outbreak started early, the know-how was very low, so the doctors were dying, so they were also afraid, for this message to work well, the government should create more facility for the medical workers, so that they will not be afraid and they will encourage the patient”.

M: Any other person wants to add?

R7: “I want to suggest that they use the survivors, to go round and tell the people that Ebola is real and when they go early, they have a greater chance of survival, with this, this message will work well”.

M: Ok, you spoke of the survivors as Messengers, what about the health workers, are they good messengers?

R1:”Well, the health worker nowadays do not treat us good, so they will not be good messengers of this message, because some people do not favour them, due to the treatment they gave the people, when this outbreak started newly”.

R2: “Well the health worker are more vital in the community, so in giving out this message, if they team up with the survivors, people will accept the message, because the survivors will explain his or her experience likewise the health worker will tell the people that they are now treating patient with care, so when they sick let go to the hospital “.

M: yes the man in the middle, I see you want to say something, please, don’t hesitate, we don’t have wrong or right

R3: “Well, as my brother is saying, people have grown hatred for the health workers, because of previous treatment”.

M: Why?

R4: “Before the outbreak of Ebola, they were not talking and taking care of the patient properly, they were just doing what they want, so with this treatment, they do not even want to encourage the health worker, so if you use them as messengers, the people will not accept this message”.

M: Is this attitude of the nurses not treating people properly still happening?

R5: “Yes, it is happening, so how do you think people will, go to the hospital, because this treatment is still going on, so with this treatment, if even is the world best message for people to go to the hospital, they will not go, so let government try to put better mechanism in place”.

M: Like which kind of mechanism?

R5: “Caution the nurses, pay their salaries on time, and also create other additional facilities at the health centres and hospital”.

R6: “Well not all the nurses are bad, some are kind and friendly, but I am telling some of this bad nurses, in this world you have to treat people with care and love, no matter the condition, that will even create a good human relationship between you and people, some really, so with this bad attitude, people will not accept them as good messengers of this message.

M: What about the traditional healers, are they important to act as good messengers to this message?

R1: “Yes, they have a vital role to play, most of our people believe in traditional medicines and are always with the traditional healers, due to this beliefs, we had huge number of Ebola positive cases, so if they use them as messengers it will be very good, the chain of infection will become less, and they will not encourage people to go for treatment to them, with them sensitizing or giving the message to people, the people will say, since the traditional healers him or herself is telling us to go the hospital, they will surely go, and the message will worker well”

M: anybody wants to say anything?

R7: we are supportive of what our elders have said, we strongly believe that the traditional healers have a say to this , they must be involved in passing such message , telling people that the first point of contact should be the hospital and not the traditional healer, they will surely listen to them, no matter what you say these people have large followers, so if they are now in front of telling people to go to the centre with sincerity and honest y we are of the opinion that it will yield dividend.

M: Ok, with about the religious leaders?’

R2: “They are also key players in given out the message to the people, it will necessary for them to be messengers to this message”.

R3: Religious leaders are very important, they will quote some sections of their holy books and people will listen to them, and this country is a country where people have faith in God, so whatever the religious people say they will accept.

R4: it is a very good channel, they must talk to a large number of their congregation on weekly basis, so the information will pass on regularly.

M: any other person wants to make his view?

R5: we are ok with this

M: Ok, what about the following channels, TV sports, jingles, radio?

R3: Well, for the television sports, we don’t have that in the (= name of the interview community) Freetown, I do not now for (=name of other parts of) of Freetown, and so it will not be good channel”.

R4: “Well, I prefer radio to other channels, because more people in this community, listen to radio, so by playing as jingle over the radio stations, the message will filter well to the people and they put it into practices”.

**(NOTE: Topic 22 – Leaflet - “9 steps while you wait for the ambulance for pick up”)**

M: Now look at this leaflet properly, they are nine steps while you wait for the ambulance to pick up, look at it critically and tell me what do you think of this?

R1: “Well by looking the leaflet, it tells us what to do while waiting for the ambulance, this normally happens when the sickness get worst, by looking at the steps it is very good, because, you will not see you love one seriously sick to the point of death, you will not come around to the help, so if even you help, if mistakenly touched, these steps are serving as a directives. People will like this message, but only that, not everybody is able read and write, so they have to take mobilizers to move house to house, with the leaflets in their hands, enlighten the community people about the steps”.

R2: Well, I have problem with step one, if I put the sick person in a room now, and unfortunately the person vomits on me, automatically I will become infected, as they are saying, so I don’t ascribe with step one, the sick person him or herself will move on their own and be isolate him or herself from the other people until the arrival of the ambulance”.

R3: “Well, in line with what my brother was saying, well you will not be permanently in close doors, with the sick person, they said you have to isolate the sick person, and do everything to the sick person at a distance, so there is nothing wrong with step one”.

R4: “It doesn’t mean that you have to be closer to the person, there should be a distance between you and the sick person, so there is nothing wrong with the message”.

R5: “Well, because some sick are uncontrollable, I suggest that step changes to isolate the sick person and help the sick person at the distance”.

R6: “Well, step one is in place, I have attended workshops, that they taught us something similar to step one, let when some is sick, whilst waiting for the ambulance, let there be one person to assist and isolate the sick person, the only thing, the person will put on plastics gloves and when moving towards the person, you have to move from the back of the person, the first step is in place, there is nothing wrong with the first step”.

R7: “After the whole process give the plastic you have used to the ambulance team and wash your hands properly, they will disinfect the whole place, and also you have to give them your clothes you were wearing at that moment of helping the sick persons”.

M: Ok, when you have seen this message, do you think people will accept it?

R1: “Yes, they will accept it, I like this community for one reason they are very cooperative in terms of messaging, they accept all the messages that they come with, for this reason we have 48 survivors in this community, so people in this community have the culture of acceptance to messages”.

M: OK, well this message is telling us about prevention for caring safely for people after calling 117. This is a set of steps for keeping a household safe in a stressful situation, do you think this message is clear?

R1: “Yes”.

M: Why?

R1: “because the message explain clearly the steps, and if you go by this steps, you will prevent yourself from Ebola, when a sick person is in your house”.

M: Ok?

R2: “Yes, I agree with brother, the steps are clear, readable, the colours are ok, and people may understand this message properly to their understanding”.

M: Ok, yes my brother?

R3: “The same as my brothers have said, it is clear”.

M: Yes, what about you sir?

R4: “For me this particular message is the best”,

M: Why?

R4: “Because this steps addresses issues that was the problem leading to the transmission of the virus, to family members, so if they work by these steps, the chain of transmission will break”.

R5: “The message is fine, but they have to tell the people that, when you are given water, you should give water from the back, don’t walk in front of the person, because you might not know, so to avoid the contact of the fluids, use the back”.

M: Ok, do you think people in this community and in the other communities will change their belief, after hearing/reading/ seeing this message?

R1: “Yes, it will totally change their beliefs and behaviour, because almost everybody in the country have awareness of the Ebola, and the destructions it has caused, so they this message will change them totally”.

M: Ok?

R2: Yes, as my brother rightly said, it will change the beliefs and behaviour, the message is good”.

M: Ok, yes my brother?

R3: “Yes, it will change the belief of the people”.

R4: Well, the people will change their belief, because everyone is aware at the moment, so they will happy to receive this message”.

M: Ok, yes sir?

R5: “Well, the only problem, when the burial team or the ambulance comes to collect a sick, person, they only take along the mattress and leave the every other items, the clothes, the plastic or gloves, so for the acceptance of this message, government through the Ministry of Health, have to tell them that they should not leave any items, the sick person has used, but the message is fine and it will be accepted, and help transformed the people”.

M: Ok?

R6: “Well, the message is good, it is good to survive Ebola, because they said when you survive Ebola, unless after ten years before, you are prone to infection again of the Ebola virus. This message will change the beliefs and behaviour of the people”.

M: Ok. Where did you get the information that when you have survive Ebola, unless after ten years, before you are after with the virus?

R6: “Well, they told me this information in a workshop we attended about Ebola”.

M: What do you think about the following messengers, MOHS, and partners, traditional authorities and community leaders, pastors and imams, do you think, are these people are the right messengers for this message?

R1: “Yes, but it only remains, one category of people”.

M: Who are this people?

R1: “The youths”.

M: Ok, meaning the youths must be involved?

R2: “Yes”

M: Ok, yes sir?

R3: “Well these people are good messengers of this message”.

M: Ok?

R4: “They are all good messenger, but these people, like the traditional authorities will be very good messenger in their traditional settings, likewise the pastors and imams in their various areas”.

R5: “Like in this community there is an imam that sensitizes us at the mosque, anytime we go for prayers, so they are good messengers”.

M: What do you think of the following, channels, for these leaflets explanations: 1. Community meeting, 2. Sermons, 3.radio discussions, 4. Ose to ose, 5. Plastic bags, and 6. leaflets?

R1: “All these channels are very important”.

R2: “For me, I prefer the house to house”.

M: Ok, why the ose to ose?

R2: “Well during the house to house sensitization, they bring out a lot of sick people and among them were positive Ebola cases, so the house to house is very important”.

M: Ok?

R4: “well, the radio discussion is also importance, but not everybody listen to radio”.

R5: “I prefer the youths, let us don’t forget about the youths”.

M: Ok, Who do you think this leaflet should be given?

R1: “The youths, worker”.

R2: “It should be given to student, teachers, and bankers”.

M: Ok?

R3: “It should be given to all category of people, hence you can read and understand the leaflet”.

M: Ok, yes sir/

R4: “For me, I prefer the police”.

M: Why?

R4: “All of this people are role models and can pass on these messages to their companion”.

(NOTE: **Topic 29 – Chlorine – Poster - ”Me na yu paddi Mr. Chlorine, ar go hep yu fo fet Ebola” )**

M: Ok, look at this picture carefully and tell me what do you think of this?

R1: “Well, it tell us to wash our hands with chlorine”.

R2: “I saw a man spraying the chlorine”.

M: Ok?

R3: “This resembles Mr Condom, as in the HIVs posters,”

M: Ok, now, look at the image and texts, the text reads “me nay u paddi Mr. Chlorine, ar go ep yu fo fet Ebola” what do you think of this message?

R1: “Well, to my own understanding, if you use chlorine to wash your hands, you may not contract the virus”.

R2: “The chlorine is saying that, he is Mr Chlorine, a friend of everybody, so people should always use it to wash their hands, so that they can fight Ebola.

M: Ok?

R3: “Well at first, there were chlorinating water all over the community, but it has become less, and people are not washing their hands again, it has become lesser, so from the picture, to my own understanding, Mr Chlorine is telling them not to forget him, he is a good fighter of Ebola virus”.

M: Ok, but do you think people will accept this message?

R1: “Yes, because the message is clear and understandable”.

R2: “Everybody knows the danger of Ebola, and this chlorine protects you from Ebola, so they will accept this message”.

M: Ok, this message wants to take away the fear of chlorine in ambulance and with burial teams. But do you think this message is clear?

R1: Well, as my brother was saying, when they started using the chlorine, the mixture was high and it was affecting the patients, but it is better now, but really the message is clear”,

R2: “well when this outbreak started, the chlorine was affecting people, because, they do not know, how to mix the chlorine, so this was affecting the patient, it is ok now and the message will be clear to them”.

R3: “The intervention of the Red Cross burial teams was a blessing, because they were using chlorine, which do not affect, they will spray it here, you will not know that they have sprayed it here, so this will help in the acceptance of this message and the clarity”.

M: Ok?

R4: “Accepting this message by the people is fine, but they need to tell the people how to use, the quantity to be mixed, with all this directives, the message will go down well to the people”.

R5: “Well, if you put the right persons to distribute and teach the people, the message will work well”.

M: What do you mean by the right persons?

R5: “Like the survivors and youths in the community, if they give out this message, it will go down properly”.

M: Do you think people in his community and the other communities will change their belief and behaviour, after, hearing or reading this message?

R1: “Yes, it will change their beliefs unless someone that is naturally stubborn”.

R2: “yes, it will change their behaviour and belief”.

M: Ok, yes sir?

R3: “Well in certain communities we have people that are very stubborn, everybody will change but they not, so these will be the people that will not change their belief after hearing this message”.

M: Ok, what do you suggest as the best way to get this message out?

R4: “Well, it should be done through radio, ose to ose”.

M: Ok?

R5: “By using popular music star like Kao Denero, big joe”.

R6: “by using the mobilizers, and youths”

M: Ok, but is the wording of this message ok?

R1:”Well for me it ok, and I like the words”.

M: Ok?

R2: “Well, it is readable, though not everybody is a literate, but the picture make them understand better”.

M: Which channel should be use?

R1: “I suggest radio, house to house, mosque and church, talk to youth at the ataya base”.

R2: “The mobilizers, the mobilizers comprises of the imam, pastors, they are also trained by the CHW, so that they will updated in passing on the message”.

M: So each communities have CHW?

R2: “yes, they have HFC and CHW, these set of group of people are there to train all category f people, as mobilizers”.

M: So they are called mobilizers?

R2: “Yes”.

M: What do you think of a poster as channel of this message?

R3: “It is good, because it is posted at public places for the people to see”.

M: Where do you think, these poster should be posted?

R4: “They should be posted at ataya base, schools, and cinemas”.

R5: “For me I prefer the religious houses, schools colleges”

M:”Ok, thank you very much.
