# Supplementary material for: Development of a set of community-informed Ebola messages for Sierra Leone
Source: PLoS Negl Trop Dis. 2017 Aug 7;11(8):e0005742. doi: 10.1371/journal.pntd.0005742 (PMC5560759; doi:10.1371/journal.pntd.0005742)
Supplement: S1 Appendix — (ZIP) [file pntd.0005742.s001.zip › Ebola messages - FGD and interview transcripts/Topic guides_final revised.docx]

1. Topic guide for key informant interview with community leaders (Imam/pastor, Traditional community leader, youth leader, women's group )
2. When did you hear about Ebola for the first time? How was the disease described to you, and what were your first thoughts about it?
3. In what ways has Ebola affected your community? Have you personally seen or known people who have had Ebola?
4. Why do you think Ebola has spread throughout Sierra Leone?
5. What do you think is the most effective way to prevent Ebola from spreading?
6. What do you think is the most effective way to treat somebody with Ebola?
7. Are there any local terms that people use to describe Ebola? What are these terms? Please explain their meanings, and how they came to be used in the context of Ebola?
8. Some people do not believe Ebola exists. Do you know people in your community who think this? Do you know why they have this view?
9. Please can you give some examples of the Ebola messages that have you heard, seen, or read? What do you think about these messages? (Probe for clarity, acceptability, understandability, and the way the message has been disseminated).
10. What do you think has been the most effective Ebola message you have come across to date? Why? Are there any Ebola messages that you think have NOT worked so well? If so, why not?
11. What do you think would be a good message to encourage people to bring patients to a treatment centre?
12. In the event of Ebola infection, do you think that people would prefer to go first to a traditional healer, to the existing healthcare system, or to the newly established Ebola health care system? Why do you think this is?
13. Some people stay at home when they think they may have Ebola. Why do you think this is? What do you think could be done to encourage them to come to a treatment centre?
14. What do you think would be a good message to support people treating infected family members at home, so that they could stay safe?
15. What do you think would be the best and most effective channel to get your new messages to people (i.e. through the radio, by text message, etc)? Which radio stations/newspapers etc do you think would be the best for transmitting your Ebola messages? Explain why.
16. Have you ever heard people talking, in either a positive or a negative way, about the:

- Ebola ambulance service;
- The Ebola holding and/or treatment centres;
- The Ebola burial teams;
- Any aspects of the existing healthcare system that is now working on Ebola care and treatment?

If so, what was said?

1. Have you heard of any new treatments for Ebola that may become available soon? If yes, what have you heard, and what do you think about them? What kinds of concerns do you think your people will have about any new treatments for Ebola? Please explain.
2. Have you heard of any vaccines for Ebola that may be coming into the country soon? (Vaccines are used to prevent people from catching Ebola in the first place, as with polio vaccine, or measles vaccine.) If yes, what have you heard, and what do you think about them? What kinds of concerns do you think your people may have about any vaccines for Ebola? Please explain.
3. What are the most common questions about Ebola that you are faced with in your capacity as a local leader/priest etc? How do you respond to these questions and concerns? What do you feel YOU need to know to enable you to respond more effectively?
4. Is there anything specific about Ebola that you think people need to understand better? What do you think would be a good way to explain this to people?
5. Topic guide for semi structured interview with health staff and health volunteers (Medical staff, including in hospitals and Primary Health Units; Community Health Workers; Health Management Committee)
6. When did you hear about Ebola for the first time? How was the disease described to you, and what were your first thoughts about it?
7. In what ways has Ebola affected your community? Have you personally seen or known people who have had Ebola?
8. Why do you think Ebola has spread throughout Sierra Leone?
9. What do you think is the most effective way to prevent Ebola from spreading?
10. What do you think is the most effective way to treat somebody with Ebola?
11. Are there any local terms that people use to describe Ebola? What are these terms? Please explain their meanings, and how they came to be used in the context of Ebola?
12. Some people do not believe Ebola exists. Do you know people in your community who think this? Do you know why they have this view?
13. Please can you give some examples of the Ebola messages that have you heard, seen, or read? What do you think about these messages? (Probe for clarity, acceptability, understandability, and the way the message has been disseminated).
14. What do you think has been the most effective Ebola message you have come across to date? Why? Are there any Ebola messages that you think have NOT worked so well? If so, why not?
15. What do you think would be a good message to encourage people to bring patients to a treatment centre?
16. In the event of Ebola infection, do you think that people would prefer to go first to a traditional healer, to the existing healthcare system, or to the newly established Ebola health care system? Why do you think this is?
17. Some people stay at home when they think they may have Ebola. Why do you think this is? What do you think could be done to encourage them to come to a treatment centre?
18. What do you think would be a good message to support people treating infected family members at home, so that they could stay safe?
19. What do you think would be the best and most effective channel to get your new messages to people (i.e. through the radio, by text message, etc)? Which radio stations/newspapers etc do you think would be the best for transmitting your Ebola messages? Explain why.
20. Have you ever heard people talking, in either a positive or a negative way, about the:

- Ebola ambulance service;
- The Ebola holding and/or treatment centres;
- The Ebola burial teams;
- Any aspects of the existing healthcare system that is now working on Ebola care and treatment?

If so, what was said?

1. Have you heard of any new treatments for Ebola that may become available soon? If yes, what have you heard, and what do you think about them? What kinds of concerns do you think your people will have about any new treatments for Ebola? Please explain.
2. Have you heard of any vaccines for Ebola that may be coming into the country soon? (Vaccines are used to prevent people from catching Ebola in the first place, as with polio vaccine, or measles vaccine.) If yes, what have you heard, and what do you think about them? What kinds of concerns do you think your people may have about any vaccines for Ebola? Please explain.
3. From your perspective as a health worker, how would you describe the general knowledge about Ebola among people in your community? What do you feel YOU need to know to enable you to respond to people’s concerns misconceptions more effectively?
4. Is there anything specific about Ebola that you think people need to understand better? What do you think would be a good way to explain this to people?
5. Topic guide for focus group discussion with young/old male/female (6-8 'ordinary people' per FGD)
6. In what ways has Ebola affected your community? Have any of you personally seen or known people who have had Ebola?
7. Why do you think Ebola has spread throughout Sierra Leone?
8. Are there any local terms that people use to describe Ebola? What are these terms? Please explain their meanings, and how they came to be used in the context of Ebola?
9. Some people do not believe Ebola exists. Do you know people in your community who think this? Do you know why they have this view?
10. Please can you give some examples of the Ebola messages that have you heard, seen, or read? What do you think about these messages? (Probe for clarity, acceptability, understandability, and the way the message has been disseminated).
11. What do you think would be a good message to encourage people to bring patients to a treatment centre?
12. What do you think would be a good message to support people treating infected family members at home, so that they could stay safe?
13. What do you think would be the best and most effective channel to get your new messages to people (i.e. through the radio, by text message, etc)? Which radio stations/newspapers etc do you think would be the best for transmitting your Ebola messages? Explain why.
14. In the event of Ebola infection, do you think that people would prefer to go first to a traditional healer, to the existing healthcare system, or to the newly established Ebola health care system? Why do you think this is?
15. Have you ever heard people talking, in either a positive or a negative way, about the:

- Ebola ambulance service;
- The Ebola holding and/or treatment centres;
- The Ebola burial teams;
- Any aspects of the existing healthcare system that is now working on Ebola care and treatment?

If so, what was said?

1. Have you heard of any new treatments for Ebola that may become available soon? If yes, what have you heard, and what do you think about them? What kinds of concerns do you think your people will have about any new treatments for Ebola? Please explain.
2. Have you heard of any vaccines for Ebola that may be coming into the country soon? (Vaccines are used to prevent people from catching Ebola in the first place, as with polio vaccine, or measles vaccine.) If yes, what have you heard, and what do you think about them? What kinds of concerns do you think your people may have about any vaccines for Ebola? Please explain.
3. What are the most common points of discussion about Ebola that you hear and/or take part in in your community? Are there things that people disagree about, in terms of both prevention and treatment, and if so, what are these?
4. Is there anything specific about Ebola that you think people need to understand better? What do you think would be a good way to explain this to people?
5. Topic guide for focus group discussions field testing draft messages

For each draft message, presented to FGD participants as intended to be distributed: written (e.g. newspaper, text message), audio (e.g. radio), or visual (e.g. TV, billboard):

1. What do you think is the message being put out here?
2. What, if anything, do you like about this message?
3. What, if anything, do you *not* like about this message?
4. Do you think you will remember this message tomorrow? If yes, what will make you remember it? If not, how should it be changed so you might be able to remember it?
5. Do you think this message will result in people taking the action that is suggested?
6. From your previous experiences of discussing Ebola, do you think this message would be something that people will talk about? Do you think they will like it, dislike it, or not have any strong opinions?
7. Do you think there are any groups of people (i.e. adult men, adult women, or youth) who will particularly respond to (like or dislike) this message?
8. What would be the best way to send this message out to people? (Radio, newspaper, billboard, text messages etc)
