# Supplementary material for: Development of a set of community-informed Ebola messages for Sierra Leone
Source: PLoS Negl Trop Dis. 2017 Aug 7;11(8):e0005742. doi: 10.1371/journal.pntd.0005742 (PMC5560759; doi:10.1371/journal.pntd.0005742)
Supplement: S2 Appendix — (PDF) [file pntd.0005742.s002.pdf]

A set of empirically-derived Ebola messages for Sierra Leone

FINAL DRAFT
April 15 2015

UMEÅ UNIVERSITET

CHaRT SIERRA LEONE
Centre for Health Research and Training

Medical Research Centre

r2hc research for health in humanitarian crises

elrha enhancing learning & research for humanitarian assistance

wellcome trust

UK aid

Produced by:

Epidemiology and Global Health Unit, Umeå University, Sweden

John Kinsman (john.kinsman@umu.se)

Medical Research Centre (MRC), Sierra Leone

Abdul Jalloh (abduljalloh@mrc-sl.org)

Heidi Jalloh-Vos (hjallohvos@mrc-sl.org)

Alpha Jalloh

Elsie Aye-Femi Lisk

Theresa Rhodes

Salamatu Moigua

Rodney Lambert

Alhaji Nyakoi

Mohammed Bockarie

Amara Ndoeka

Centre for Health and Research Training, Sierra Leone (CHaRT-SL)

Muriel Harris (muriel.harris@louisville.edu)

Kars de Bruijne

Titus Boye-Thompson

Hussainatu Abdullah

Kalilu Totangi

Artists: Amara Tengbeh (Tee Designs)

Joseph Mambu (Wisdom Graphics)

And in consultation with:

Ministry of Health and Sanitation

National Ebola Response Centre

Focus 1000

Centers for Disease Control

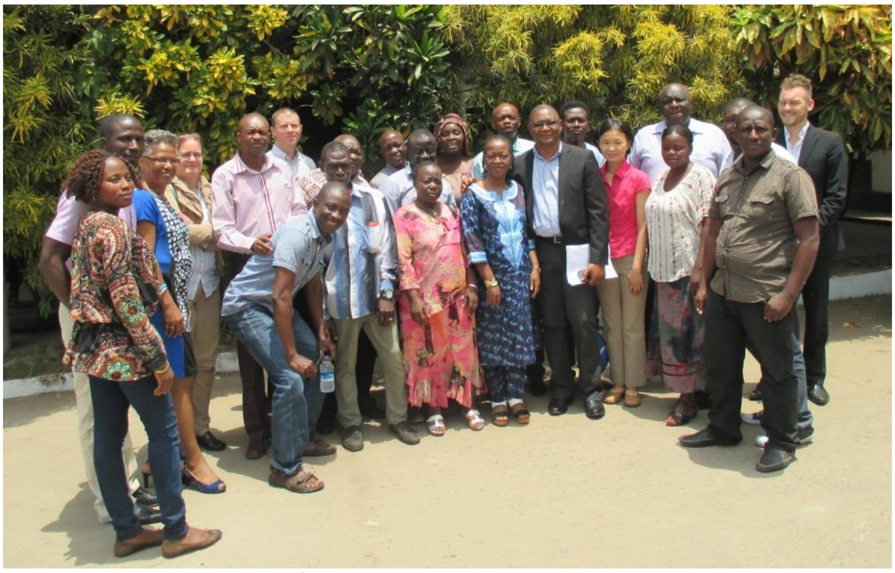

Contents

Introduction 5
Methods 6
A methodological challenge in message refining . . 9
Notes about the messages . . . 9
Notes about the messengers and channels . . 11
The Messages 13
Topic 1, Burial teams and respect (*Priority topic*) . . 14
Topic 2, Burial teams and bribery (*Priority topic*) . . 17
Topic 3, Fear of ambulances (*Priority topic*) . . 20
Topic 4, Misconception, killing of patients (*Priority topic*) . 24
Topic 5, Distrust of health system (*Priority topic*) . . 25
Topic 6, Fear of chlorine (*Priority topic*) . . . 29
Topic 7, Stigma against survivors . . . 31
Topic 8, Stigma against Ebola workers . . . 33
Topic 9, Get early treatment . . . 35
Topic 10, Call 117 39
Topic 11, Caring for the sick while waiting for ambulance . 41
Topic 12, Staying safe while waiting for burial team . . 44
Topic 13, Ebola denial 46
Topic 14, 117 prank calls 48

Introduction

This document presents a set of Ebola messages that are based on the findings of qualitative interviews and focus group discussions conducted in 'hotspot' areas of rural Bombali District and urban Freetown (see Figure 1). This field work, which took place between January and March 2015, aimed to provide an empirical basis for developing culturally contextualized messages that will promote Ebola treatment-seeking behaviour. All the messages presented below are derived from issues that emerged through the data, and they respond directly to community concerns about various aspects of the Ebola response and about Ebola itself.

The project has been conducted by a consortium including the Epidemiology and Global Health Unit, Umeå University, Sweden; the Medical Research Centre (MRC), Sierra Leone; and the Centre for Health and Research Training, Sierra Leone (CHaRT-SL). Financial support was provided by Research for Health in Humanitarian Crises (R2HC), as part of their £8 million Emergency Ebola Health Research Call, funded equally by the Wellcome Trust and DFID. R2HC is managed by Enhanced Learning and Research for Humanitarian Assistance (ELRHA).

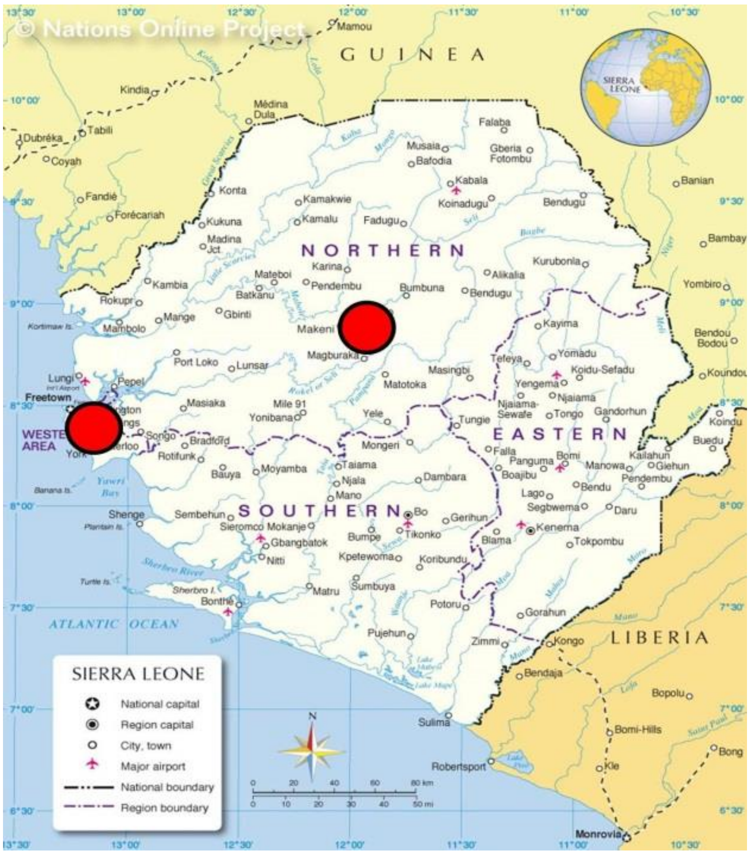

Figure 1: Map of Sierra Leone showing the two study areas

Based on discussion with Stephen Gaojia, the National Ebola Response Centre (NERC) National Operations Coordinator, we have followed two core principals while developing this messaging document: (i) It is critically important that the *messenger* is trusted if the message is to have any validity; and (ii) Messages must reflect the constraints of the infrastructure that is available. In other words, it is important not to promise something in a message that cannot be delivered by the available resources.

Methods

The starting point of this applied anthropological study has been that the voice of the Sierra Leonean people should be heard in the development of messages to promote Ebola treatment-seeking behaviour in the country. On this basis, an iterative approach was taken, as indicated in Figure 2 below.

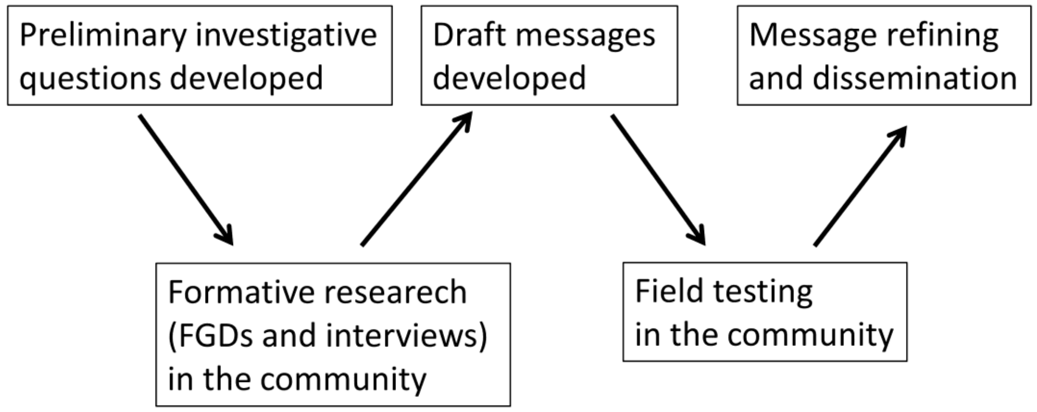

Figure 2: Schematic representation of the study design

The project started formally with a stakeholder meeting in Freetown on January 17, 2015, attended by representatives from NERC, the Ministry of Health and Sanitation (MoHS), CDC, and Focus 1000. Seven MRC research assistants and transcribers, all experienced in qualitative research, were then trained, after which field work was undertaken in the two study areas: urban Freetown, and rural Bombali. Sixteen Focus Group Discussions (FGDs) were conducted with 'ordinary' people (i.e. those without an official leadership position in their respective communities, but who were interested and able to talk openly). The 16 FGDs were stratified by age and sex, and were attended by over 100 people. We also held a total of 24 individual, in-depth interviews with community leaders of various sorts, 12 in each district. These included religious leaders, traditional leaders, traditional healers, women's and youth leaders, and medical staff engaged in the Ebola response. The informant categories are presented in Table 1.

Questions in the FGDs and interviews were concerned with awareness of Ebola itself, of Ebola treatment, and also issues to do with the Ebola response, such as ambulances and burial teams. Interviews were conducted in Krio. The data were transcribed directly into English, and data quality was ensured by comparing audio recordings with the English language transcriptions, with all transcription anomalies being corrected. All informants participated on a voluntary basis. Ethical clearance was provided for the study by the Sierra Leone Scientific and Ethics Committee on November 21 2014.

Interviews	Northern Region	Western Region
Imam/pastor, Traditional community leader, youth leader, women's group	5	5
Medical staff, including in hospitals and Primary Health Units; Community Health Workers; Health Management Committee	5	5
Traditional healers	2	2
**FGDs, 6-8 'ordinary people' per FGD**	**Northern Region**	**Western Region**
Male, <25	2	2
Male, >25	2	2
Female, <25	2	2
Female, >25	2	2

Table 1: Participants in the formative research

The data were subjected to thematic analysis by members of the research team with collective expertise in Sierra Leonean culture and history, communications, and appropriate methodological skills. This process identified specific areas of community concern, which in turn enabled us to ascertain which topics required messages to be developed. A workshop was then held in Freetown from March 12-17 2015, and in consultation with stakeholders from MoHS and CDC, draft messages for promoting Ebola treatment-seeking behaviour were developed, and suitable dissemination channels identified. Both English and Krio versions of the messages were produced.

In order to contribute to the 3-day national stay-at-home on March 27-29 2015, we were requested by NERC to fast-track the messages into operation when they were still only in draft form, and when they had not yet been field tested and validated. We therefore rapidly developed a working document that could be used by stakeholders in this process, and submitted it to the appropriate authorities.

During late March and early April, the draft messages were field-tested in a series of 8 FGDs. Four FGDs were conducted in both of the study districts; these involved many of the same people who had participated in the initial FGDs, and who were therefore already familiar with the project and its objectives. The principle we applied was for each message to be discussed in two different FGDs, by both women and men, and by both urban and rural respondents. For example, one message might be reviewed by rural men and urban women,

while another could be discussed by rural women and urban men (see Table 2). In order to keep the discussions focused, and to avoid the informants becoming tired, each FGD covered up to five different topics. Field testing sought to assess understanding, acceptability, likely effectiveness of the messages, and appropriate distribution channels.

	Male Rural; Female Urban	Female Rural; Male Urban	Male Rural; Female Urban	Female Rural; Male Urban
**Burial teams**	X			
**Burial team - bribes**		X		
**Early treatment**			X	
**Early treatment**		X		
**Misconception: killing**				X
**Misconception: killing**	X			
**Ebola denial**				X
**Health Care System**				X
**Seeing is believing**			X	
**Stay safe - waiting ambulance**		X		
**Stay safe - waiting burial team**			X	
**Stigma - survivor**	X			
**Stigma - HCW**				X
**Mr Chlorine**		X		
**Fear of ambulance - chlorine**	X			
**Fear of ambulance**				X
**117 prank calls**			X	
**NUMBER OF TOPICS / FGD**	4	4	4	5

Table 2: Field testing of the message topics, by informant category

Based on feedback from the field testing, the messages were refined by the research team, and a final version of the messages was produced – this is presented below. Many of the messages we have developed fit into the categories given in the Social Mobilisation Action Consortium's Consolidated Message Guide for Ebola Communication: Burial teams; Get early treatment; Stay safe and protect your family while you wait; and Celebrate survivors, and addressing stigma. However, we also identified some issues that the Ebola response system is doubtless aware of, but which do not appear in any of the accredited messages in the SMAC Consolidated Message Guide: Fear of chlorine; Fear of ambulances; and '117' prank calls.

This final document will be presented and submitted at a meeting at the Ministry of Health and Sanitation on Wednesday April 15 2015, the last day of the project. We include both

English and Krio versions of the messages, and we hope that local languages will be used wherever possible when they are disseminated.

We are aware that our final dissemination is taking place at what appears to be the tail end of this epidemic. However, past experience from Central and East Africa suggests that once Ebola strikes in a given geographical location, it is likely to return at some stage in the future. We feel it is therefore of great importance that our validated messages remain on file in the Ministry of Health and Sanitation as a resource that can be instantly accessed in the event of a future Ebola outbreak in Sierra Leone.

A methodological challenge in message refining

A principle we adopted in the message refining process was that if both groups independently rejected a given message, we would drop it from the list altogether. This did not happen with any of the messages, however, and we did not need to implement this principle.

We did nonetheless face a situation in which one group was very positive about a particular message, while the other group was quite negative. Careful review of the critical transcript indicated that the person who had spoken first may have set the tone for the others, who followed and then developed her critical view. This is a recognised methodological challenge with FGDs, whereby the first comment from an informant can define the way the rest of the group speaks. Importantly, however, this does not mean that the critique should be rejected. In the case in question, we looked closely at the critical comments and concluded that there were indeed some valid issues raised, and we adjusted the message accordingly. Criticism in field testing, even if it goes counter to the views of others, should always be considered seriously, and acted upon if appropriate.

Notes about the messages

1. *All the messages are empirically derived*: Everything we have included in this document has been developed out of the data we have collected. As such, these messages represent a truly bottom-up messaging strategy, which explicitly aims to address concerns raised by the community about Ebola itself and about different aspects of the Ebola response.

2. *Prioritisation of key topics*: Through our formative research, we identified a range of areas of real concern that people have about the Ebola response, some of which are already recognised in the Social Mobilisation Action Consortium’s official messaging database, but some of which are not. With regards to the latter, we were surprised by the strength of feeling about ambulance drivers, which were perceived, especially

early in the epidemic, as being insensitive towards patients. It was also striking to note that people really disliked the chlorine that has been such an integral part of the Ebola response: there were many informants who believed that chlorine has been deliberately used to kill people. Strong concerns were also raised about the health system more broadly, as well as about the way burial teams have been treating corpses. These apparently widely held feelings have acted as significant barriers to Ebola treatment-seeking behaviour, and through this they have likely fuelled the epidemic by creating conditions whereby people have decided to treat sick family members at home, thus continuing the cycle of infection in the community. We have therefore prioritized these topics in this document, and we recommend that they are adopted more intensively and/or before the others.

3. *The importance of using Krio*: We have provided both English and Krio versions of all the messages, in order to promote the use of Krio. The high illiteracy rate in the country makes it necessary to use Krio where possible: as one informant explained (in Krio), "*Me, I want everything to be in Krio*".

4. *You can't please all of the people all of the* time: We recognize that not everyone will support all the messages, and, as we found during the field testing, a few might strongly oppose messages that the majority like. Although the reasons for their feelings should be investigated and understood, the fact that some people don't like a message does not necessarily mean it should be excluded.

5. *Different levels of field testing for different messages and channels*: Visual messages and those with simple text phrases were of course easier to field-test than, for example, community meetings. With these latter channels, we used comments from the field testing on related topics to fine tune some aspects of them; but, by definition, they remain conceptually more fluid then the more tangible messages.

6. *Poster colours*: During field testing, our informants stressed that posters and leaflets should not be coloured predominantly in *either* red *or* green, as these are the colours of the ruling party and the main opposition party respectively. If a given message was associated in people's minds with one or other political parties, it may undermine the validity of that message. Informants voiced a preference instead for posters and leaflets to be *either* in other bright colours, *or* in black and white. This principle should be born in mind for all posters and leaflets.

7. *The need for parallel work in the health system*: Messages to increase confidence in the health system must be matched by good services, otherwise the validity of those messages, as well as any others that may be associated with them in people's minds,

will be undermined. Addressing parallel operational issues is therefore an essential component of the messaging strategy.

Notes about the messengers and channels

1. *Messenger and channel – definition of terms*: In this document, we define 'Channel' as the means of distributing a given message, such as poster, leaflet, radio jingle, house-to-house meetings etc. The 'Messenger' is either an individual (such as a traditional leader, or a community mobiliser) or an institution (such as MoHS). If the messenger is an individual, they will likely be speaking, face to face or on the radio, or they will be depicted visually on a poster or leaflet. If the messenger is an institution, it will probably be on the basis of a logo, or some other official endorsement of the message.

2. *Messengers and channels for urban vs rural populations, and for women and men*: Analysis of our formative research and field-testing data indicated that the messages themselves were more or less generic for women and men, and for urban and rural dwellers: in most cases, there was no real need to provide different messages for different target populations. However, both the messengers and the channels do need to be carefully considered according to the target population. For example, chiefs and town criers were seen as being far more relevant channels for rural populations than for urban populations. As one informant explained during field-testing: "*In Freetown, people do not have respect for the Chiefs, but in the provinces they have respect for the Chiefs, and their subjects listen to them, so they are good messengers in the provinces, but not in Freetown*." By contrast, social media were seen as an attractive means of reaching to urban youth.

   It was also clear that we need to consider the gender of the messengers, whereby, for example, a message aimed at promoting early treatment should depict a woman, since women are the primary care givers, and it would be important for them to relate to the message. These points are reflected in the messages presented below.

3. *The importance of utilizing complementary messengers and channels*: During the field testing, one informant stated the following: "*I want to suggest, in passing on this message, let the religious leaders pass it on to their congregational members, let the youth leaders target the youths, the old target the old, teachers and lecturers pass on it on to their students. With this, it will be nice and the message will pass on to everyone.*" This use of multiple, mutually reinforcing messengers and channels is a key principle of our messaging strategy, as we recognize that no single messenger or channel can reach all members of the population. Rather, an array of voices needs to be heard in order to get the points across to as many different audiences as possible.

4. *The importance of the community engaging with the messengers***:** A major finding that has emerged through the formative research and the field-testing is the fact that *people want to hear messages in the flesh from people they trust*. In addition to suggesting passive, non-interactive messaging strategies such as radio discussions, jingles, posters and so on, we therefore strongly recommend that the messages should also be disseminated through community meetings, house to house visits, and other such face-to-face dissemination strategies. In addition to the desire of people to engage and discuss these issues on an active, two-way basis, there are also strong practical reasons for pursuing such approaches: by no means everyone has access to a radio, especially in rural areas; many people say they don't have time to stop and read a poster; and the country's very high illiteracy rate means that text-based posters will simply be missed by a large proportion of the population.

   Wherever we have suggested a poster as a means of disseminating a particular message, we have therefore also indicated that leaflets with a similar message should be disseminated by knowledgeable and trusted community members via house-to-house visits and other face-to-face meetings. Such leaflets could be printed on a large scale and left with people to read and discuss with friends and family at home. For logistical reasons, it may be necessary to focus these intensive efforts on hotspot areas, and not necessarily to work on a nationwide basis. Even so, it would of course require an effort to mobilise all these people, and it would be critical that efforts are made to ensure that the message does not evolve away from what was intended as it goes down the chain and into the community.

5. *Traditional healers should not be used as Ebola messengers*: Although traditional healers are undoubtedly trusted by many in the community, our analysis of the formative research and field-testing data indicates that including them as messengers for promoting Ebola treatment-seeking behaviour may be counter-productive. Comments about them included, for example: "*If a Doctor or Nurse comes to tell me that Ebola is real, I will believe, because they too are dying. But like the herbalist when he comes to tell me, I will not believe because he is not at the Centre (= health centre). What can he tell me about Ebola, and besides government has banned them so I will not believe them mostly*"; and "*Traditional healers may not be good messengers of this message, because they are not happy about the message, thinking they will lose their customers.*" We have therefore not included traditional healers as messengers in this document.

THE MESSAGES

BURIAL TEAMS – PRIORITY TOPIC

Topic 1(a): There were many complaints in our formative research about disrespectful behaviour of burial teams towards the dead, and, to some extent, towards the bereaved families. Many comments referred to incidents early in the epidemic, since when protocols have been improved; but doubts still remain in some people's minds. This message complements 1(b), and it aims to reduce unsafe, family-led burials by promoting officially designated burial teams.

Audience: Burial Teams, Communities (rural and urban)

Channels: Note the potential mutual reinforcement of these two channels together.

- Posters distributed by NGOs, CBOs, DHMTs through PHUs, using local youth group, chiefdom councilors, and social mobilizers. To be posted at community gathering points, ataya base, court barrie, pharmacies, parks/bus stops, inside/on public transport, but NOT on people's houses.
- Leaflets combined with community house to house visits/meetings using own community members

Messenger: Produced by MoHS and partners, with the logos of MoHS and partners visible to show who has produced it.

Messages:

1. *Poster/leaflet*: "We treat corpses with respect" / "wi respect dae dae bodi
2. *Poster/leaflet*: "Let us work together to ensure safe burial" / "Leh wi join an en gi wi pipul dem gud berrin"

People depicted in image: Burial Teams, family members, pastor and imam

Possible amendment to image that could be considered, if redrafted:

- Based on feedback during field-testing, suggest to remove depiction of chlorine spraying, as this may be seen as a negative point by some in the community.

Operational issues:

Burial teams must be trained and cautioned on how to behave to bereaved families and/or communities, and how to perform a respectful burial:

- Consult with family
- Wrap properly
- One identifiable grave
- Respectfully putting into the grave
- Work with pastors/imams
- Workers not under influence of alcohol
- Respectful attitude of workers

BURIAL TEAMS – PRIORITY TOPIC

Risk: If good operational standards are not met, communities will not be willing to participate in safe burials, and the risk of secret burials will continue.

“wi respect dae dae bodi”

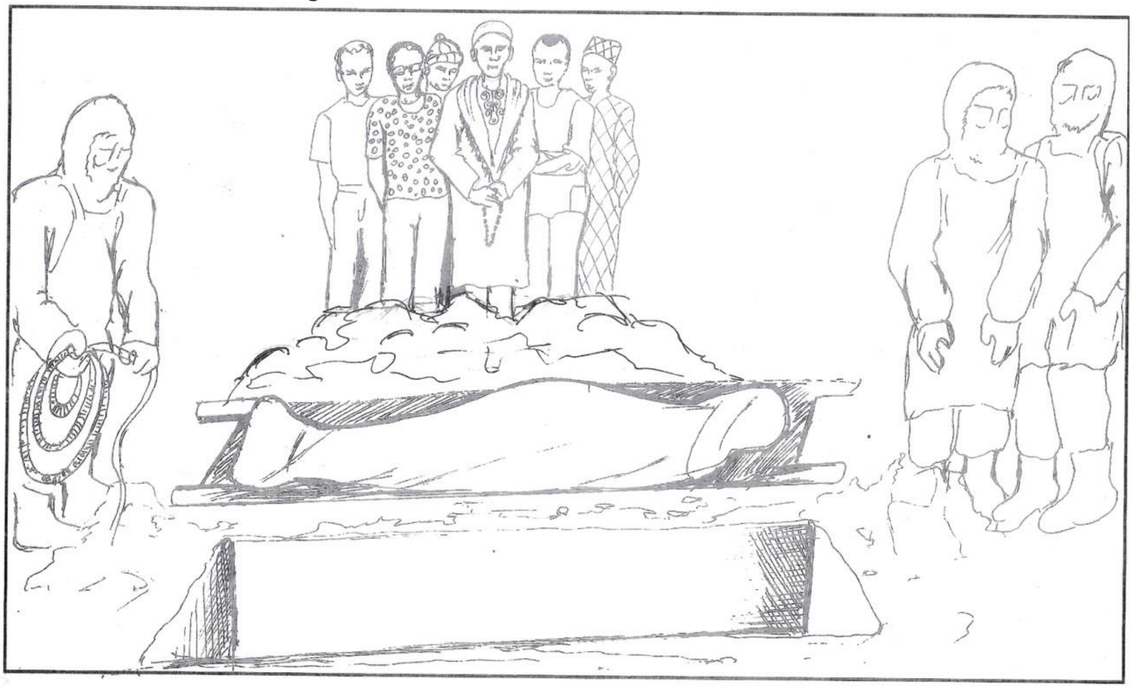

“wi respect dae dae bodi”

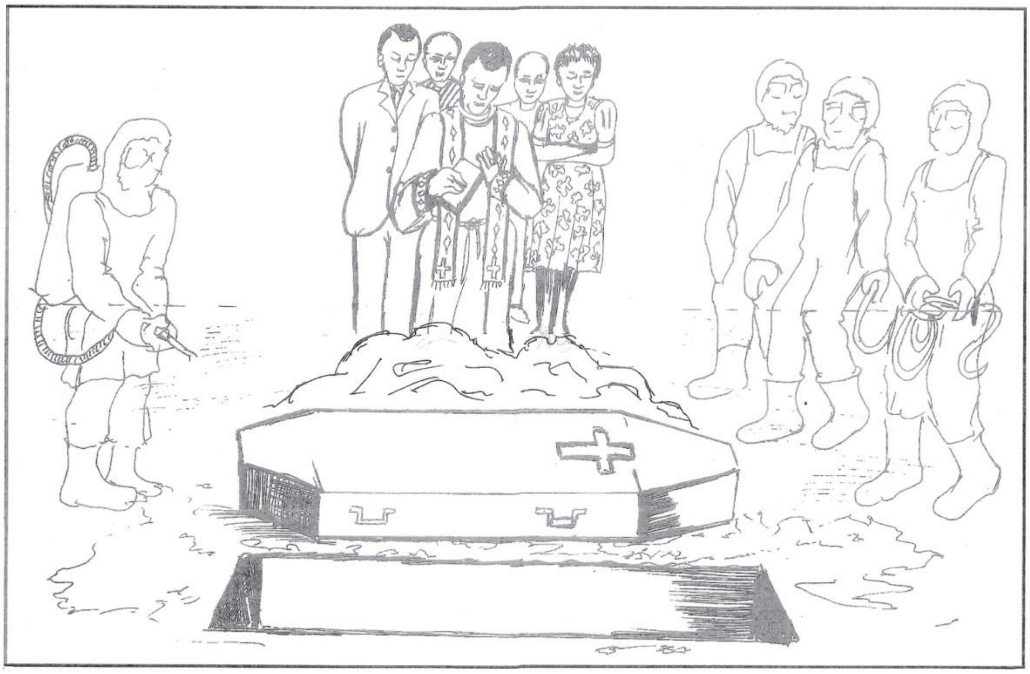

BURIAL TEAMS – PRIORITY TOPIC

Topic 1(b): There were many complaints in our formative research about disrespectful behaviour of burial teams towards the dead, and, to some extent, towards the bereaved families. Many comments referred to incidents early in the epidemic, since when protocols have been improved; but doubts still remain in some people's minds. This message complements Topic 1(a), and it aims to reduce unsafe, family-led burials by promoting officially designated burial teams. **Note:** This was not field-tested, but we have incorporated comments from Topic 1(a) as appropriate.

Audience: Communities, with focus on older men and women (e.g. those involved in the washings / burials); both urban and rural; youths involved in harassing burial teams

Channels: Community meetings, sermons, radio

Messenger: Religious, youth and traditional leaders including cultural society leaders, both male and female; chiefs in rural areas – ideally have people who have taken part in respectfully conducted burials with Ebola Burial teams.

Message (talking points):

1. "The Burial Teams are doing their job to keep our community safe. Let us cooperate with them." / "Leh wi join hand wit di burial team fo keep wi community safe"
2. "Trust the burial team: they will bury your loved one respectfully" / "Believ say di burial team go berr yu fambul wi respect"
3. "Be patient, wait for the burial team, or you will be infected" / "Duya una patient en wait fo di burial team, or una go catch di sick"
4. The imam/pastor/chief says: "Not washing loved ones is acceptable in our tradition."

Operational issues:

Burial teams must be trained and cautioned on how to behave to bereaved families and/or communities, and how to perform a respectful burial:

- Consult with family
- Wrap properly
- One identifiable grave
- Respectfully putting into the grave
- Work with pastors/imams
- Workers not under influence of alcohol
- Respectful attitude of workers

Risk: If good operational standards are not met, communities will not be willing to participate in safe burials, and the risk of secret burials will continue.

BURIAL TEAMS – PRIORITY TOPIC

Topic 2(a): Our formative research indicated that family members have been offering money to burial teams, and burial teams have been asking for money, in contravention of the bye-laws. This message complements Topic 2(b), and it aims to show that money should not exchange hands between the families of Ebola victims and burial teams. The red 'X' over the image is intended to speak to people who do not read.

Audience: Communities, burial teams

Channels: Note the potential mutual reinforcement of these two channels together.

- Posters distributed by NGOs, CBOs, DHMTs through PHUs, using local youth group, chiefdom councilors, and social mobilizers. To be posted at community gathering points, ataya base, court barrie, pharmacies, parks/bus stops, inside/on public transport, but NOT on people's houses.
- Leaflets combined with community house to house visits/meetings using own community members (e.g. religious leaders and community/peer mobilisers)

Messenger: Produced by MoHS and partners, with the logos of MoHS and partners visible to show who has produced it.

People depicted in the picture: Burial teams, family members (male and female)

Messages:

- Picture of burial team and family exchanging money with red 'X' through it
- Text: "Safe burial is free; Do not pay or receive money for safe burial" / "Safe berrin na free, e no right fo gi or tek moni fo am".

Operational issues:

- Burial teams should be properly trained and supervised; and disciplined by the appropriate authorities (e.g. DERC, Red Cross) if involved in corruption.
- No equivalent supervision can be mounted to ensure that the community does not offer bribes.

Risk:

- If the bye-laws are not enforced then people will continue to pay/receive money and/or conduct secret burials

Safe berrin na free –
e no right fo gi or tek moni fo am

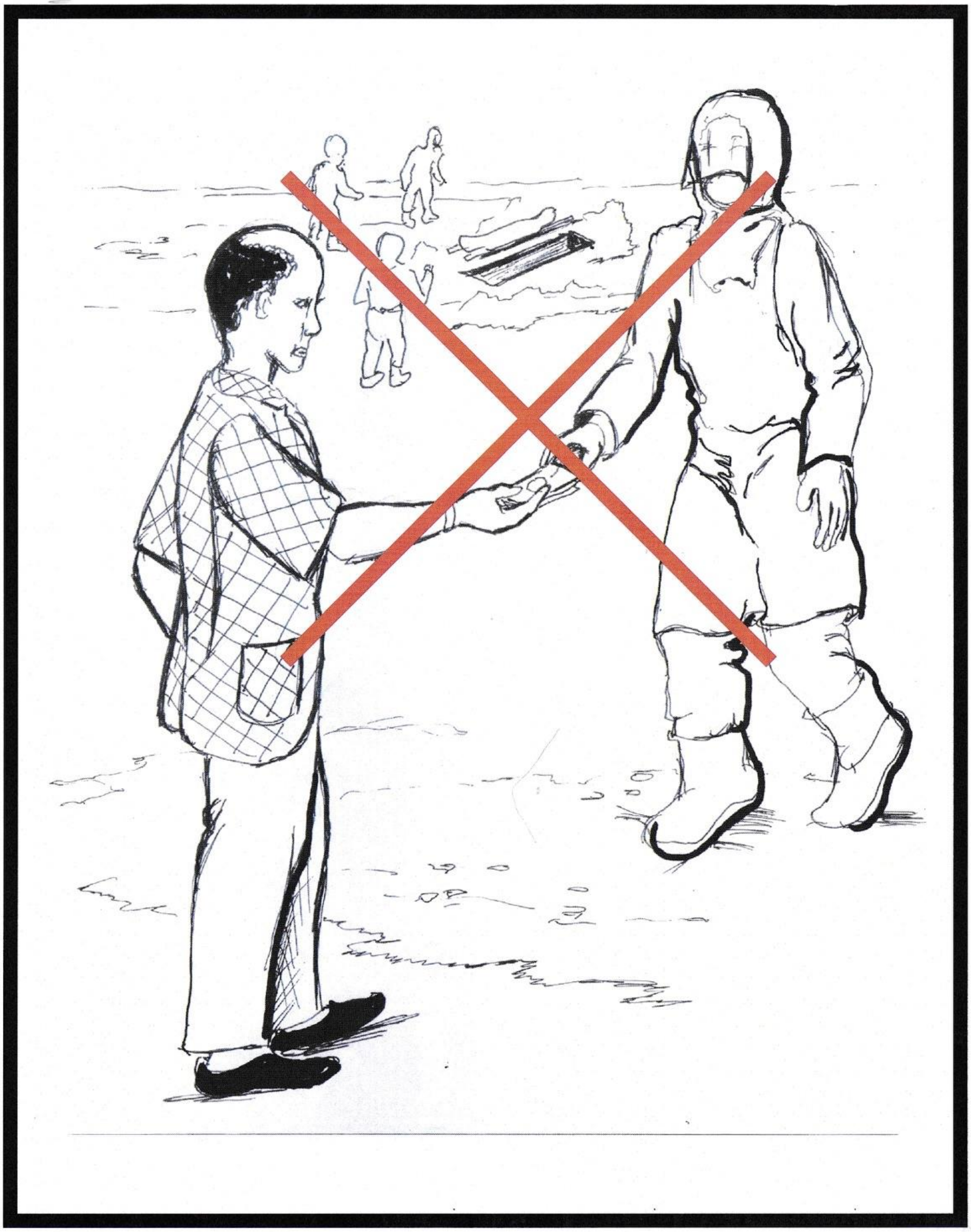

BURIAL TEAMS – PRIORITY TOPIC

Topic 2(b): Our formative research indicated that family members have been offering money to burial teams, and burial teams have been asking for money, in contravention of the bye-laws. This message complements Topic 2(a), and it aims to show that money should not exchange hands between the families of Ebola victims and burial teams.

Audience: Communities, burial teams

Channels:

- Urban areas: Radio jingle, Mobile PA system
- Rural areas: Town Crier

Messenger: Neutral, friendly voice, both male and female

Message:

- "Safe burial is free; Do not pay or receive money for safe burial" / "Safe berrin na free, e no right fo gi or tek moni fo am".

Operational issues:

- Burial teams should be properly trained and supervised; and disciplined by the appropriate authorities (e.g. DERC, Red Cross) if involved in corruption.
- No equivalent supervision can be mounted to ensure that the community does not offer bribes.

Risk:

- If the bye-laws are not enforced then people will continue to pay/receive money and/or conduct secret burials

FEAR OF AMBULANCES – PRIORITY TOPIC

Topic 3(a): Many people complained in the formative research about different aspects of the ambulance service, including over-speeding, drunk driving, disrespectful drivers, lack of air, and excessive use of chlorine while in transit. This message aims to address these concerns – which have inhibited people from going for treatment – by showing the ambulance to be a safe and reliable means of getting to the treatment centre. It complements 3(b) and 3(c).

Audience: General public

Channels: Note the potential mutual reinforcement of these two channels together.

- Posters distributed by NGOs, CBOs, DHMTs through PHUs, using local youth group, chiefdom councilors, and social mobilizers. To be posted at community gathering points, ataya base, court barrie, pharmacies, parks/bus stops, inside/on public transport, but NOT on people's houses.
- Leaflets combined with community house to house visits/meetings using own community members

Messenger: Produced by MoHS and partners, with the logos of MoHS and partners visible to show who has produced it.

Message: Picture + accompanying text:

- People depicted: Ambulance staff / family members / patient; AC/airflow also shown in the ambulance
- Text: "The ambulance is the best and safe way to go to the hospital to receive treatment. The ambulance is well ventilated so that the patient will not have trouble breathing" / "A beliv say di ambulans na di best en safe way fo go hospitul. Fine breeze de blow inside the ambulans".

Operational issues:

- Ambulance staff must follow protocol as follows:
    - No over-use of siren
    - No over-speeding
    - Adapting speed to the road conditions
    - No alcohol on the job
    - Respectful behavior
    - Not taking bribes
    - Not taking passengers or loads
    - Being on time
    - Not overdosing chlorine
    - Drivers know the terrain
    - Explain to patients and family about the ventilation in the ambulance

A beliv say di ambulance na di best en safe way fo go hospital.

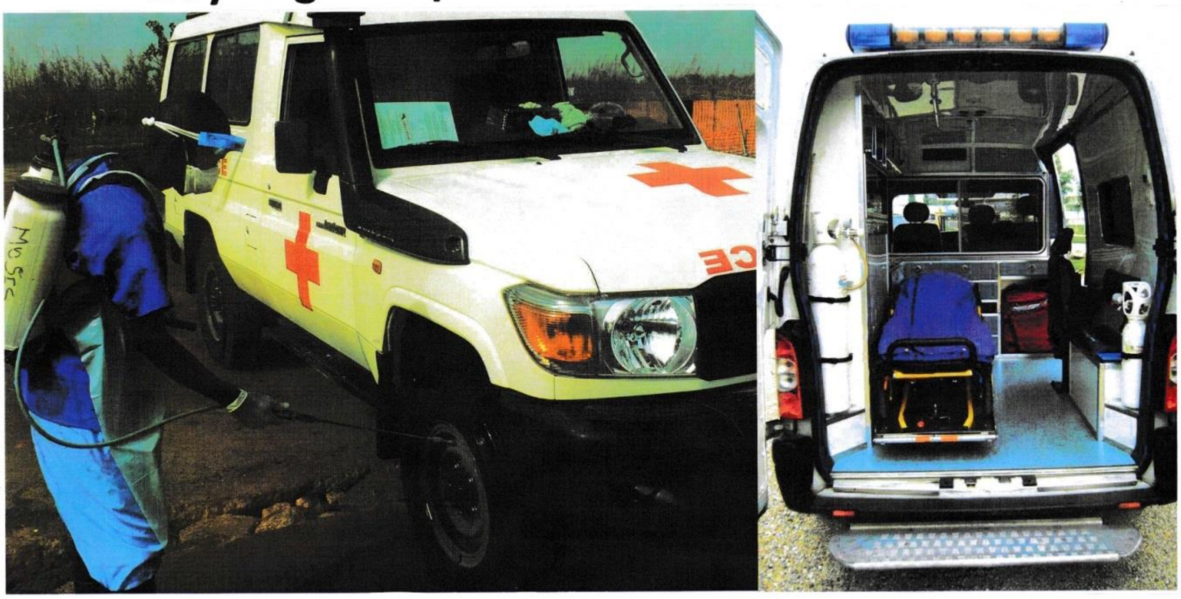

Fine breeze de blow insai di ambulance

FEAR OF AMBULANCES – PRIORITY TOPIC

Topic 3(b): Many people complained in the formative research about different aspects of the ambulance service, including over-speeding, drunk driving, disrespectful drivers, lack of air, and excessive use of chlorine while in transit. This message aims to address these concerns – which have inhibited people from going for treatment – by showing the ambulance to be a safe and reliable means of getting to the treatment centre. The use of survivors as the messenger is intended to allay people's concerns by drawing on the experiences of those who actually used the ambulance service when they fell ill. The message complements 3(a) and 3(c).

Audience: General public

Channel: Jingle (radio, mobile PA system)

Messenger: Voices of both male and female survivors in one jingle

Message: "The ambulance is the best and safe way to go to the hospital to receive treatment. The ambulance is well ventilated so that the patient will not suffocate" / "Min a survivor, A beliv say di ambulans na di best en safe way fo go hospitul. Fine breeze de blow inside de ambulans.

Operational issues:

- As 3(a)

FEAR OF AMBULANCES – PRIORITY TOPIC

Topic 3(c): Many people complained in the formative research about different aspects of the ambulance service, including over-speeding, drunk driving, disrespectful drivers, lack of air, and excessive use of chlorine while in transit. This message aims to address these concerns – which have inhibited people from going for treatment – by showing the ambulance to be a safe and reliable means of getting to the treatment centre. The use of community leaders of different varieties, as well as music stars and survivors, is designed to reach different segments of the population. The message complements 3(b) and 3(c).

Audience: General public

Channel: Community meetings, sermons, radio discussion

Messenger: Religious leaders, youth leaders, women's leaders, Chiefs (especially in rural areas), survivors, music stars (combination of male and female)

Message (talking points):
- "The ambulance is safe and reliable" / "Di ambulans na di best en safe way fo go hospitul"
- "The health workers are friendly. They will take care of you in a nice way" / "De healthcare wokmen dem frenly, den go hol yu fine"
- "There is ventilation inside the ambulance" / "Fine breeze de blow inside di ambulans"

Operational issues:
- It is essential that the people leading these discussions are knowledgeable and trained in the subject.
- As 3(a)

FEAR OF HEALTH SYSTEM – PRIORITY TOPIC

Topic 4: This message addresses misconceptions about the killing of patients by the health system, as raised during our formative research, by showing what actually happens when people are taken for treatment. The working principle is that transparency about the process reduces doubt, denial, and fear, and thereby treatment-seeking behavior will be promoted. This message tackles a broadly similar issue to Topic 5, and is therefore complementary.

Audience: General population, rural and urban

Channels:

1. Photo album showing the different steps in the process of Ebola treatment, disseminated through the Chief/community volunteers, CHWs, youth leaders, or other 'person-to-person' volunteers. One album made for urban areas, and one for rural areas, so people relate to the context of the photos. A photo album will be an attractive, high status channel, though it will be relatively expensive – should be focused on hotspot areas.
2. Leaflet, with the same images (rural and urban), disseminated using similar means as the photo album. Cheaper and easier to produce than the photo album; for wider distribution.

Messenger: Produced by MoHS and partners, with the logos of MoHS and partners visible to show who has produced it.

Messages: Flow of photos showing the process from falling sick to either confirmed Ebola or not. (Note that we field-tested the *concept* of this message, but we did not show actual photos during the validation exercise). The photo album and leaflet needs to show:

- I. Ambulance interior
- II. Smiling medics
- III. Blood tests and/or swab
- IV. Holding centre (inside and outside)
- V. Ebola Treatment Centre (inside and outside)
- VI. Lying in bed with drips, water/ORS and/or tablets
- VII. Food
- VIII. Phone conversation with family members
- IX. Going home.

Operational issues:

- Enough need to be produced so as to ensure wide access
- Photo album needs to be robust, laminated, colourful and good looking
- Needs to be presented in a very non-threatening, compassionate, and positive way
- In reality, medics may not be so friendly and smiling, and this could undermine the message

FEAR OF HEALTH SYSTEM – PRIORITY TOPIC

Topic 5(a): This message, alongside 5(b) and 5(c), addresses beliefs about general mistreatment of patients within the health system, as raised during our formative research, by showing what actually happens when people are taken for treatment. Based on the principle that transparency about the process reduces doubt, denial, and fear, and by indicating that you will be treated well in the ambulance and in the hospital, this visual message aims to promote early treatment-seeking behaviour. This message tackles a broadly similar issue to Topic 4, and in that sense it is complementary.

Audience: General population, urban and rural

Channels: Note the potential mutual reinforcement of these two channels together.

- Urban and rural:
  - Posters distributed by NGOs, CBOs, DHMTs through PHUs, using local youth group, chiefdom councilors, and social mobilizers. To be posted at community gathering points, ataya base, court barrie, pharmacies, parks/bus stops, inside/on public transport, but NOT on people's houses
  - Leaflets combined with community house to house visits/meetings using own community members
- For urban areas, add: SMS, Facebook, Whats App, in order to reach out to youth.

Messenger: Produced by MoHS and partners, with the logos of MoHS and partners visible to show who has produced it.

Messages:

1. "Go to the hospital when you're sick. You'll be well looked after." / "Wae yu sick, go na hospitul, den go hol yu fine."
2. "Go in the ambulance when you're sick. You'll be well looked after." / "Wae yu sick, go wit di ambulans, den go hol yu fine."

Operational issues:

- Generic issues to do with quality of health system: sufficient qualified HCWs with supervision to ensure validity of message; good ambulance workers; HCWs should have counseling skills and give feedback to families; treatment centres should have enough food + medicine etc.

FEAR OF HEALTH SYSTEM – PRIORITY TOPIC

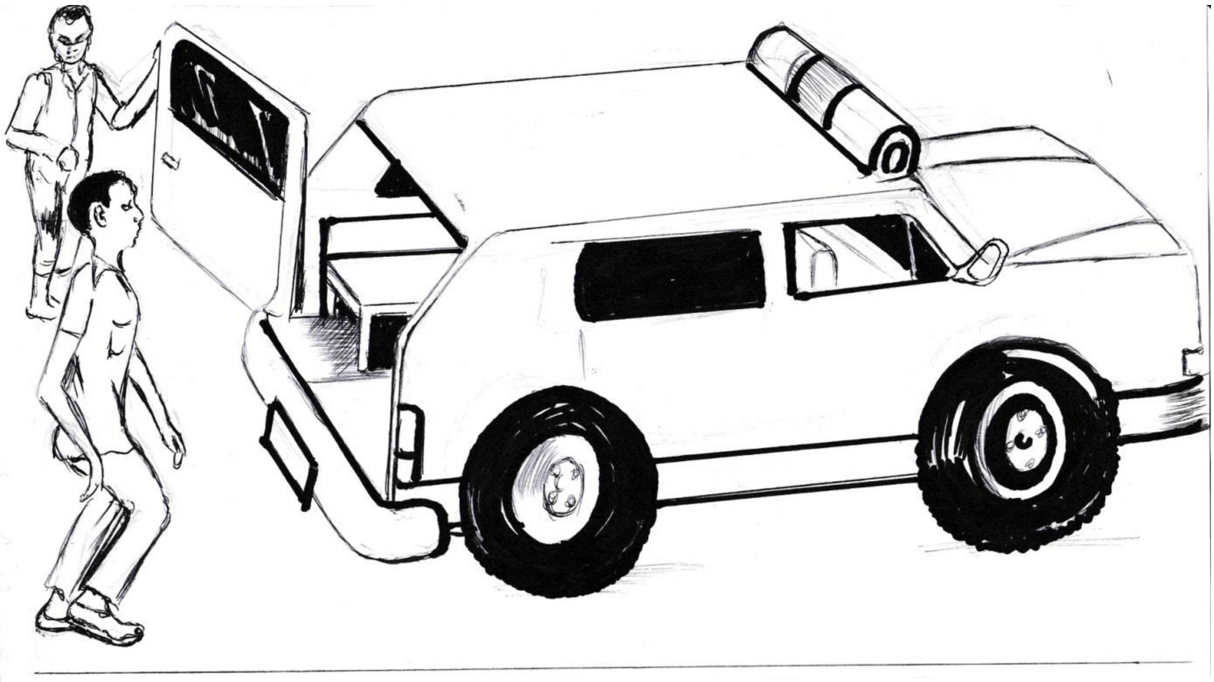

"Wea yu sick, nor fraid fo go wit di ambulance, den go hol yu fine"

NOTE: We removed 'nor fraid fo' from the final text, as there were indications during field testing that any reference to fear would immediately provoke doubt in the readers' minds, as if we were trying to cover something up. The drawing itself is not as developed as we would like, either, so we recommend developing another image for this.

FEAR OF HEALTH SYSTEM – PRIORITY TOPIC

Topic 5(b): Complement to 5(a) and 5(c); addresses beliefs about general mistreatment of patients within the health system, through audio media.

Audience: General population, urban and rural

Channels:

- Rural and urban: Jingle (radio, mobile PA system); radio discussion

Messenger: Voices of both male and female messengers in one jingle/discussion:

- Youth role model/music star [suggestions included: Kao Denero, Nasser Ayoub, and Big Joe]
- Survivors
- Pastors and Imams
- Disc jockeys
- Youths and community members

Messages/talking points:

1. "Go to the hospital when you're sick. You'll be well looked after." / "Wea yu sick, go na hospitul, den go hol yu fine."
2. "Go in the ambulance when you're sick. You'll be well looked after." / "Wea yu sick, go wit di ambulans, den go hol yu fine."

Operational issues:

- Messengers must be known and trusted, as well as knowledgeable / trained on Ebola
- Giving feedback to families about patients is needed; the national database of patients must be used effectively so that families are informed about where their loved ones have been taken, as well as if they die
- To facilitate two-way communication, we suggest a channel (117 or another number) for people to enquire about family members who are in treatment, and give the opportunity to report concerns
- Generic issues to do with quality of health services: sufficient qualified HCWs with supervision to ensure validity of message; good ambulance workers; HCWs should have counseling skills and give feedback to families; treatment centres should have enough food + medicine etc.

FEAR OF HEALTH SYSTEM – PRIORITY TOPIC

Topic 5(c): Complement to 5(a) and 5(b); addresses beliefs about general mistreatment of patients within the health system, using a participatory approach at community level.

Audience: General population including traditional, religious, cultural, youth, women leaders

Channel: Community meetings/participatory theatre

Messenger: Youth groups

Messages: Drama for community meetings*:* script to include:

- One bad story: patient gone too late for treatment, dies at gate of treatment centre;
- One good story: patient goes early to the centre, gets treatment and food, explains about IV treatment and injection for pain, survives, gets a certificate and care package.

Operational issues:

- Professionals need to be available to train youth groups how to do participatory theatre
- Generic issues to do with quality of health services: sufficient qualified HCWs with supervision to ensure validity of message; good ambulance workers; HCWs should have counseling skills and give feedback to families; treatment centres should have enough food + medicine etc.

FEAR OF CHLORINE – PRIORITY TOPIC

Topic 6: Fear of chlorine in ambulances, and by burial teams, was a major issue in the formative research. Several informants indicated that they believed chlorine was killing patients in transit to treatment centres, which would clearly act as a significant disincentive to go for treatment if it was necessary. This message aims to address these concerns by presenting chlorine as a friendly support in the fight against Ebola.

Audience: General public

Channels: Note the potential mutual reinforcement of these two channels together.

- Posters distributed by NGOs, CBOs, DHMTs through PHUs, using local youth group, chiefdom councilors, and social mobilizers. To be posted at community gathering points, ataya base, court barrie, pharmacies, parks/bus stops, inside/on public transport, but NOT on people's houses.
- Leaflets (tri-fold, step-by-step pictorial), combined with community house to house visits/meetings using own community members

Messenger: Produced by MoHS and partners, with the logos of MoHS and partners visible to show who has produced it.

Message: Cartoon character with friendly character, 'Mr Chlorine'.

- Text: "I am Mr. Chlorine, your friend in the fight against Ebola". / "Mi na yu paddi Mr. Chlorine, ar go hep yu fo fet Ebola"

Operational issues:

- Ambulance and burial teams should use chlorine in the right way and with the right dose.
- Ventilation should be provided inside ambulances, and teams should wait for some time after spraying before people board the ambulance.

Possible amendments to image that could be considered, if redrafted:

While people were generally positive about Mr Chlorine in the field-testing, they saw more focus on washing hands and spraying than the fact that he is intended to be a friend in the fight against Ebola – which is the image that we wanted to produce based on the formative research. Therefore:

- Have Mr. Chlorine interacting with others, for example, children?
- Make the face more welcoming and happy?

FEAR OF CHLORINE – PRIORITY TOPIC

Me na yu paddi Mr. Chlorine, ar go hep yu fo fet Ebola

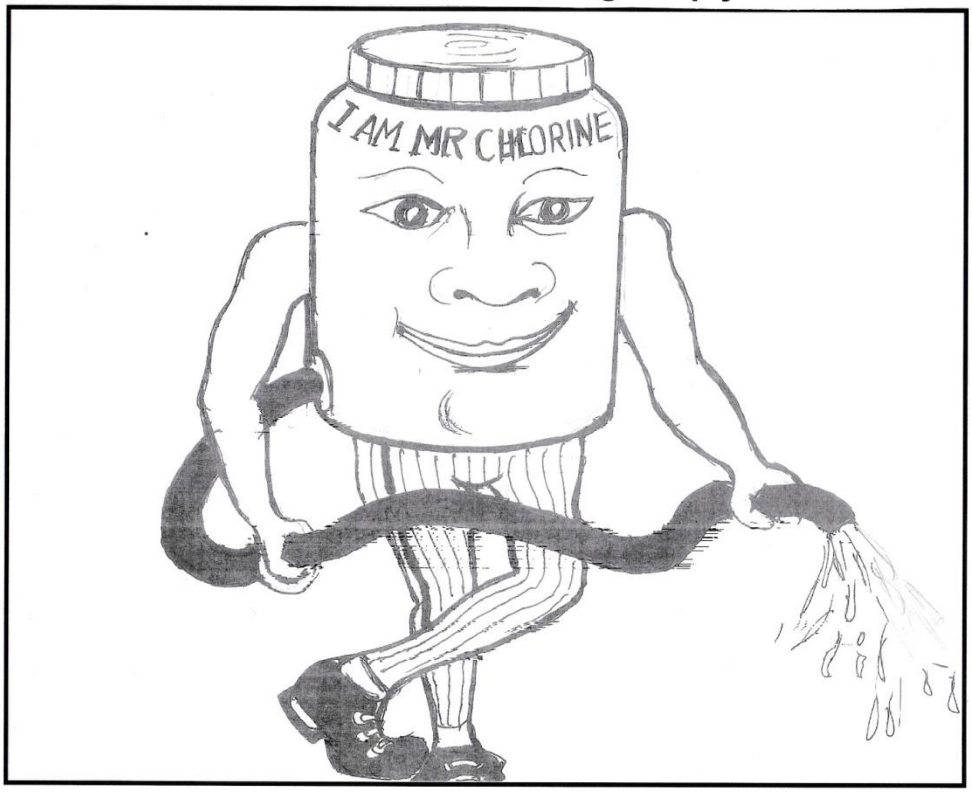

STIGMA: SURVIVORS AND EBOLA RESPONSE WORKERS

Topic 7(a): In spite of considerable efforts to reduce stigma against Ebola survivors, it was clear from our formative research that stigma still persists in some communities. This message complements 7(b), and it aims to contribute to these stigma-reduction efforts.

Audience: General Public including traditional, cultural, religious, youth leaders

Channel: Radio drama and drama in the community. (Note that we field-tested the *concept* of this message, but we did not conduct actual drama shows during the validation exercise. Radio drama in particular was seen as a very popular channel for the message).

People depicted in the drama: Ordinary community members, and Ebola survivors (where possible involving actual Ebola survivors as actors)

Messages: Two scenes:

I. The first showing the community not welcoming an Ebola survivor (gossip, laughing, turning their back etc.);
II. The second showing the whole community coming out dancing, clapping, general jubilation, welcoming the survivor back into the community.

The drama includes a song with the following phrases:

- "Welcome our brothers and sisters who have survived Ebola back into their homes, jobs and communities" / "Kabo to una homes, una wokplace en una community, una wi broda en sista dem way don survive Ebola"
- "Do not laugh at or avoid Ebola survivors" / "Nor Laf or run from porsin way bin don get Ebola"
- "Don't Spread gossip about Ebola survivors" / "Nor kongosa porsin way bin don get Ebola".

STIGMA: SURVIVORS AND EBOLA RESPONSE WORKERS

Topic 7(b): This message complements 7(a), and it aims to contribute to stigma-reduction efforts.

Audience: General public

Channel: Jingle (radio, mobile PA systems)

Messenger: Friendly voices, male and female

Messages:

- "Welcome our brothers and sisters who have survived Ebola back into their homes, jobs and communities" / "Kabo to una homes, una wokplace en una community, una wi broda en sista dem way don survive Ebola"
- "Do not laugh at or avoid Ebola survivors" / "Nor Laf or run from porsin way bin don get Ebola"
- "Don't Spread gossip about Ebola survivors" / "Nor kongosa porsin way bin don get Ebola".

STIGMA: SURVIVORS AND EBOLA RESPONSE WORKERS

Topic 8: Stigma against people working in the Ebola response (HCWs, burial team members, ambulance staff) was noted in our formative research. This message aims to address the issue by pointing out that Ebola response workers are heroes, and they should be embraced, not stigmatised. Note that we use the word 'hero' here on the basis that Ebola workers have made an active choice to contribute by doing very dangerous work. Survivors are not heroes in the same sense, since they did not choose to be infected.

Audience: Communities

Channels: Note the potential mutual reinforcement of these two channels together.

- Posters distributed by NGOs, CBOs, DHMTs through PHUs, using local youth group, chiefdom councilors, and social mobilizers. To be posted at community gathering points, ataya base, court barrie, pharmacies, parks/bus stops, inside/on public transport, but NOT on people's houses
- Leaflets combined with community house to house visits/meetings using own community members

People depicted in the images: People involved in the Ebola response: (HCWs, burial team members, ambulance staff); important to have *both* female and male workers represented in different versions of the poster.

Messages:

- *Poster 1*: An Ebola response worker in his/her work setting, for example, taking care of a patient: "Di Ebola workman den na i champion den!" (Our Ebola workers are our heroes!)
- *Poster 2*: Nurse in his/her uniform, wearing gloves, next to several survivors + certificates in front of one of the centres saying "Nurse Susan na hero, e hep plenty Ebola Survivor dem for liv" (Nurse Susan is a hero, who helped many Ebola patients survive)

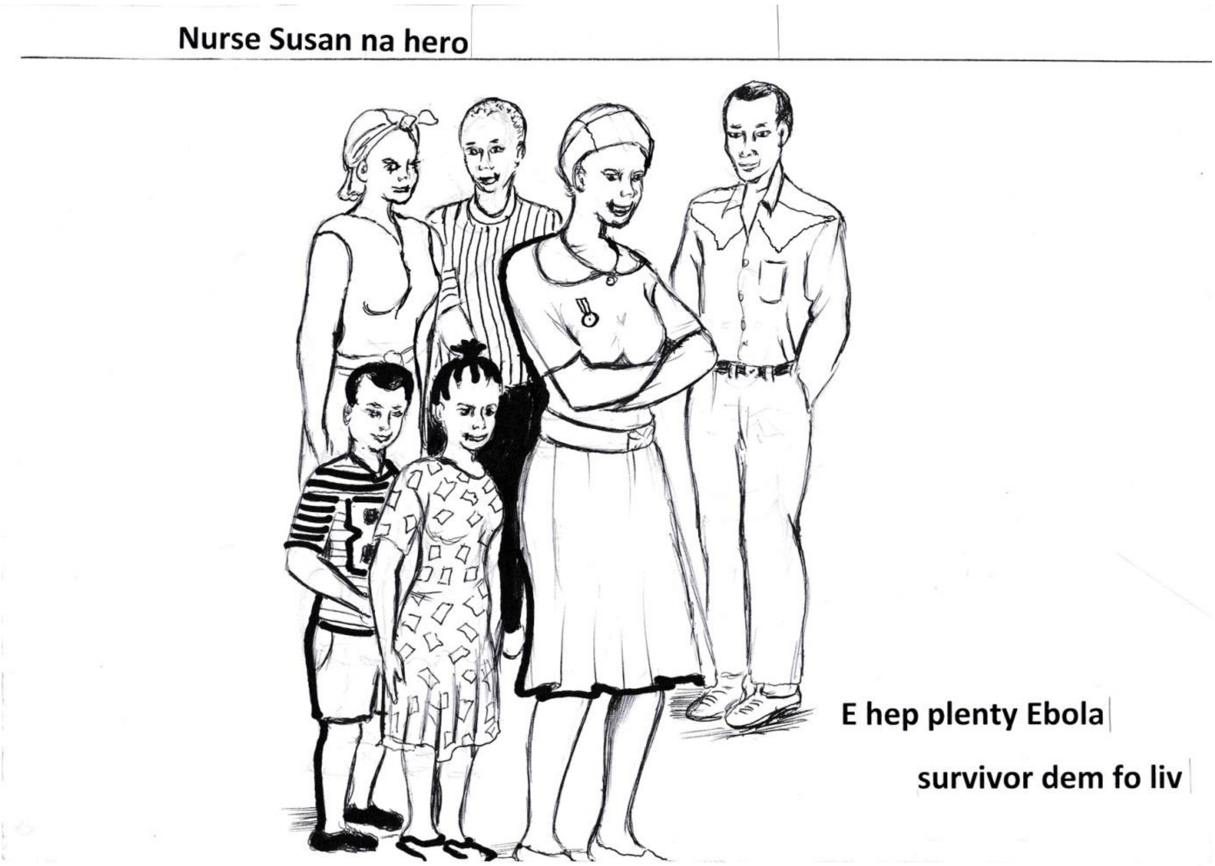

Note: During field-testing, 'Nurse Susan' was not immediately recognized as a nurse until people read the text; rather she was simply seen as part of the family. Since many people do not stop to read text on posters, or they are unable to read, the message here may therefore be lost. A redrafted image should make the nurse/ambulance driver/burial team member visually more distinct from the family.

GET EARLY TREATMENT

Topic 9(a): For a number of reasons, as indicated elsewhere in this document, people do not always go for early treatment, which reduces their own chances of survival and also risks spreading the infection to their families. This message aims to encourage people to go early for treatment if they fall sick, by using the voices of people who have direct experience of Ebola (survivors and health workers), and of influential and respected leaders. This message complements 9(b).

Audience: Communities, especially urban

Channel: Radio jingle, Mobile PA system

Depicted in the message: Ebola survivor, health worker, traditional/religious leader

Messages: *In one radio jingle (90 seconds commercial, three people talking):*

1. Testimony of survivor: “My name is Sallay. I survived the Ebola sick because when I contracted the virus I went to the hospital early.” / “Mi name na Sallay, a survive de Ebola sick becos wae e ketch me, a go trait na hospitul.”

2. Corroboration by a health worker: “That is very true, my sister. That is even why we the doctors say the earlier you go to the hospital, the greater your chance of survival.” / “Na tru me sister, na dat mak we den docta kin say, if yu go hospitul quick, yu get big chance fo survive.”

3. Endorsement from religious /traditional leader: “That is the spirit, my people. Please let us all work together to drive this sickness from our country.” / “So na de word dat o me fambul den. Duya una mek we wok with all man fo don dis sick ya na we kontri.”

Operational issues:

- Serious points were raised in our field testing about the poor attitudes and practices of some health workers, and the effect these could have on the validity of the message if they persist. These messages should therefore only be used if a good, welcoming attitude of health workers can be expected towards clients and the community.
- Overall, health care workers should show a good, friendly attitude; services should be accessible and available; and the authorities should treat rumors promptly and provide adequate information. An environment should be created to improve patient confidence and trust, which may include ensuring that all patients are treated with dignity, and that a patient-centered approach is fostered in all health care settings.

GET EARLY TREATMENT

Topic 9(b): This message is a complement to 9(a), and it aims to encourage people to go early for treatment if they fall sick by using the experiences of survivors to promote the fact that those who go early for treatment have a better chance of survival than those who delay.

Audience: Communities

Channels: Note the potential mutual reinforcement of these two channels together.

- Posters distributed by NGOs, CBOs, DHMTs through PHUs, using local youth group, chiefdom councilors, and social mobilizers. To be posted at community gathering points, ataya base, court barrie, pharmacies, parks/bus stops, inside/on public transport, but NOT on people's houses.
- Leaflets combined with community house to house visits/meetings using own community members

Depicted in the picture: Ebola survivors (male and female)

Messages: Picture / caricature of survivor with certificate saying: "If you go to hospital early, you improve your chances of recovery, like I did" / "If yu go hospitul quick, chans dae say yusef go well lekeh me".

Note that the text is slightly different from that in the pictures, as it became clear during field testing that the original text was misleading: it indicated that it you went early for treatment then you *would* recover, which clearly is not always the case. The text here is technically more accurate.

Operational issues: Similar to 9(a): It is important that health workers are motivated to treat patients well, and that trust in the health system is revived.

"Na because ar go hospital quick, na dat mek ar well"

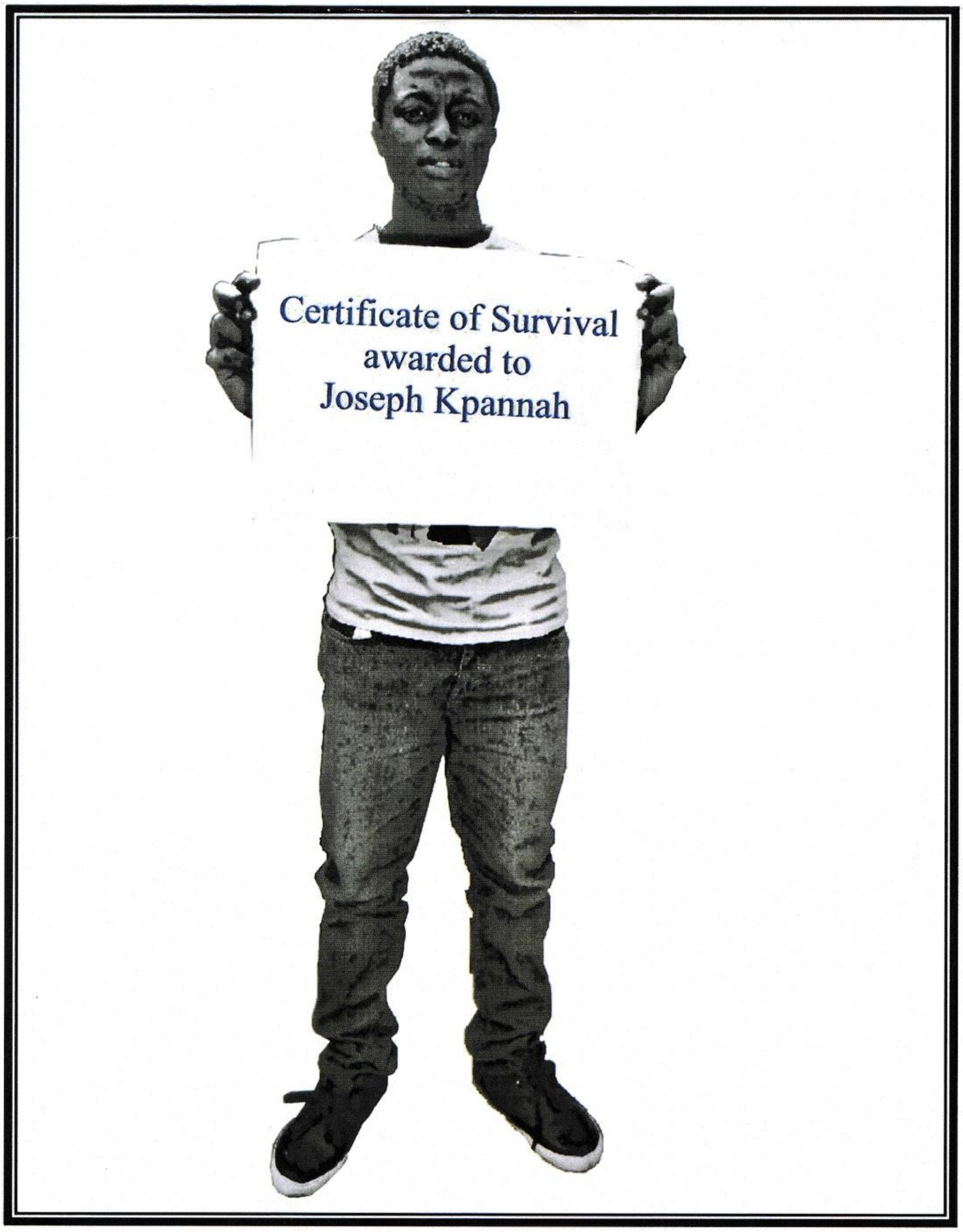

GET EARLY TREATMENT

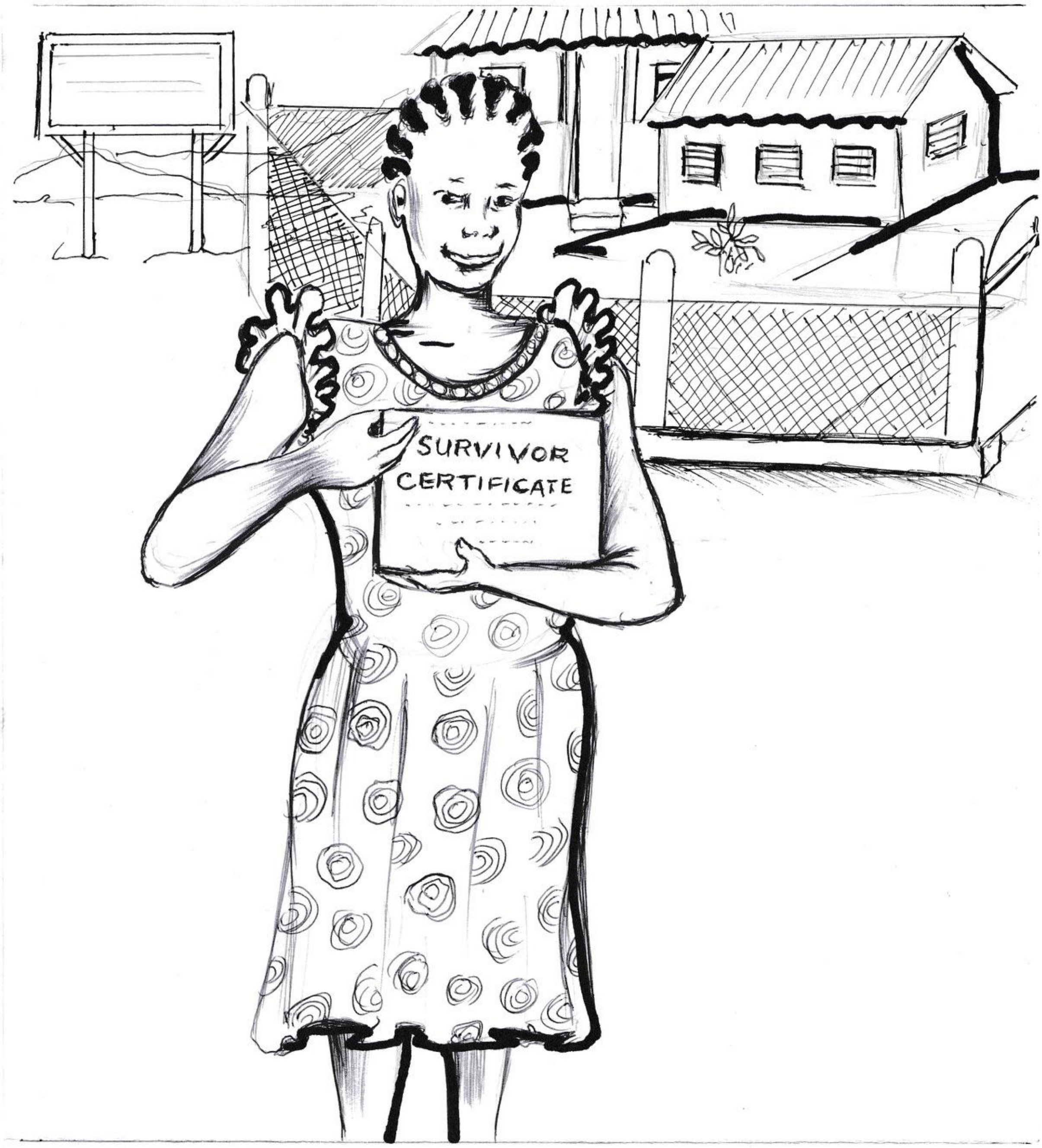

"Na because ar go hospital quick, na dat mek ar well"

GET EARLY TREATMENT

Topic 10: One of the challenges mentioned by our informants was the early, non-specific symptoms of Ebola, which can be similar to those of, for example, malaria. These make it difficult for people to know how to respond if they or someone in the family falls sick. In order to improve Ebola survival rates, and to reduce the risk of continued transmission in the community, it is essential that people go early for treatment. Using a few very simple words, this message aims to encourage people to call 117 as a first step towards receiving proper medical attention.

Audience: General population: rural and urban

Channel:

- Rural: Radio jingle, Cars with loud speakers, Town criers/Chief
- Urban: Radio jingle, Posters, Facebook/What's app, Cars with loud speakers, Button and/or wristband

Messenger:

1. POSTER: Ordinary woman calling 117 (because women are the primary care givers)
2. AUDIO:
    a. Music artist [A female artist would be ideal for the concept, but all those suggested during field testing were male: Nasser Ayoub; Kao Denero; LAJ]
    b. Football stars: Mohamed Kallon; Kei Kamara were specifically named

Messages: *No matta di sick, call 117* [Call 117 for any illness you have]

Operational issues:

- What training or additional resources for 117 may be needed to cope with this?
- If 117 extends beyond Ebola, additional training would be required for operators.
- 117 could be overloaded.
- There may be resistance to this message in areas where doubts about hospitals continue to exist.

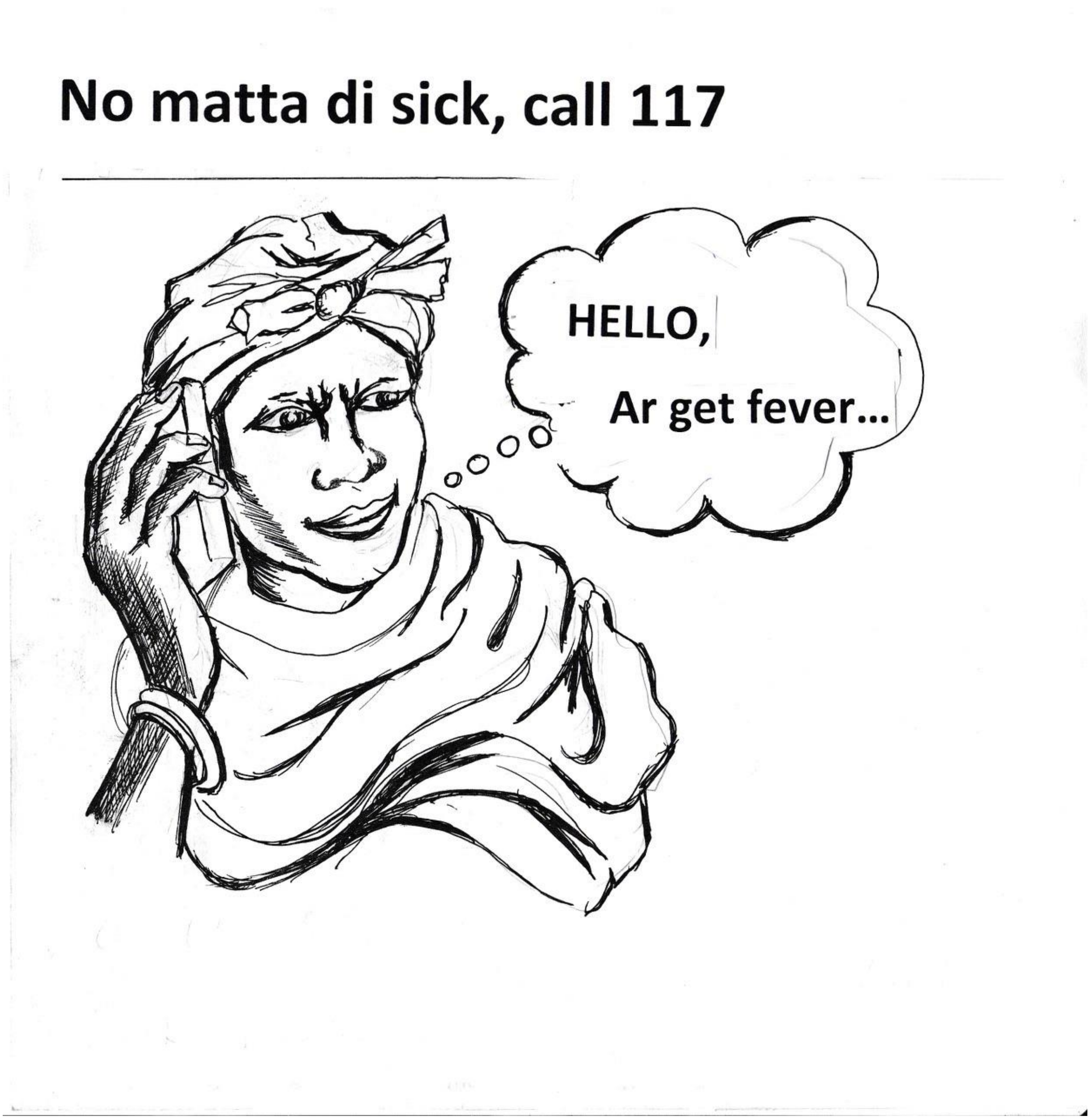
No matta di sick, call 117
HELLO,
Ar get fever...

STAY SAFE AND PROTECT YOUR FAMILY WHILE YOU WAIT

Topic 11: This is a prevention message aimed at helping people caring safely for those who are sick at home after they have called 117**.** The message provides an actionable set of steps for keeping a household safe. People want to have directions for what to do in this highly stressful situation.

Audience: Household and family members of suspected cases; therefore the general population must be targeted, especially in hotspots. Main focus on women, as primary care-givers.

Channel: Different channels should be used for this message, as mutual reinforcements.

- Posters distributed by NGOs, CBOs, DHMTs through PHUs, using local youth group, chiefdom councilors, and social mobilizers. To be posted at community gathering points, ataya base, court barrie, pharmacies, parks/bus stops, inside/on public transport, but NOT on people's houses.
- Leaflets (tri-fold, step-by-step pictorial), combined with community house to house visits/meetings using own community members
- Plastic bags; these may be available at the home, and they could fulfill a dual role, both as information givers and as barriers for when giving water to the patient etc. Distributed by market women when selling food.
- Radio discussion and Community meetings covering the topics, led by Pastors, Imams, and community leaders.

Messages: While waiting for the ambulance:

1. Nor touch di sick posin [Don't touch the patient.]
2. Leh di sick posin go wan sai, en mek wan posin de wach am. [Let the person go somewhere by themselves, and choose one person to take care of them.]
3. Gie di sick posin wata. [Give water to the patient.]
4. Wea u gie wata en if u get for see bot am, wer klin plastic bag nay u an, en nor touch u face. [When giving water and cleaning up, cover your hands and arms with clean plastic bags, and don't put your hand up to your face.]
5. Wea u de pull di plastic, duya try nor for touch di wata en oda tin wea de komot na di bodi. [When removing the plastic, make sure you don't touch any of the fluids.]
6. Wen u pull di plastic, was u ol an wel wit soap en wata. [After removing the plastic, wash your hands and arms well with soap and water.]
7. Use stik fo put all watin u use, wisi posin nor go touch am. [Use a stick to transfer any soiled materials in a place where nobody will touch them.]
8. Wae di ambulans kam, duya gie dem all watin u use pan di sik posin, den go no watin for do wit am. [When the ambulance arrives, be sure that they take the plastic and anything used by or for the patient for safe disposal.]
9. Tel u fambu en u padi den [Pass this message on to your family and friends!]

Operational issues:

- Ambulance staff must give full information about (not) using spaces and materials that have been sprayed once the patient has been removed
- Rural: fewer rooms to isolate people, and less access to plastic covering
- Logistics and production of posters, leaflets and plastic bags
- Posters and leaflets should have bright colours and be large enough for people to see clearly what the images show and the text says.
- Facilitating community work to disseminate the message, and ensuring credible spokespeople etc

Risks:

- We are accepting that people *will* touch their loved ones – it is unavoidable. Therefore we need to provide people with materials and means to do this safely.
- While giving water and cleaning up, some people may not adopt the recommendations and may thereby become infected.

STAY SAFE AND PROTECT YOUR FAMILY WHILE YOU WAIT

This is an example of a two-sided, tri-folded leaflet showing the message. As a poster, the formatting would be different.

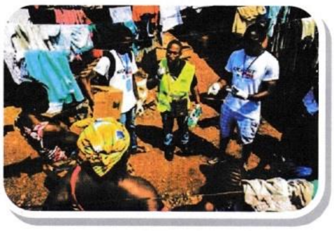

Tel u Fambul

en u padi dem

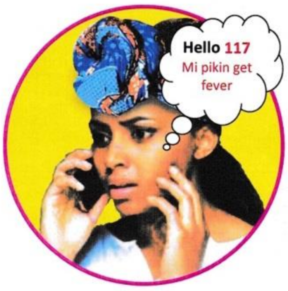

While you wait for the

ambulance

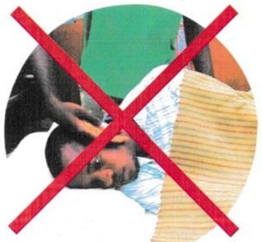

Nor touch di sick posin

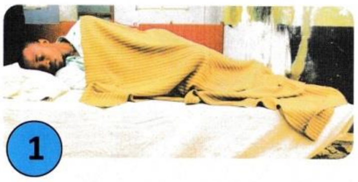

Put di sick posin wan sai,
en mek wan posin
de wach am

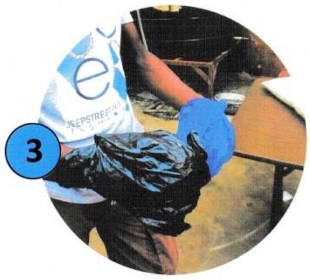

Wae u de pull di plastic, duya try nor for touch di wata en oda en oda tin wea de comot na di bodi

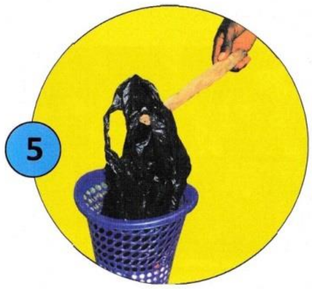

Put all watin u use,
usai posin nor go touch am

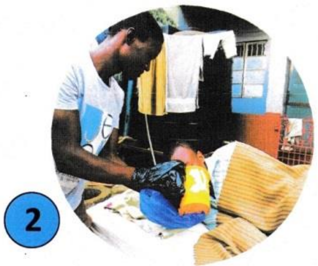

Gie di sick posin wata.
Wae u gie wata en if u get for see bot am, wer kiln plastic en nor touch u face.

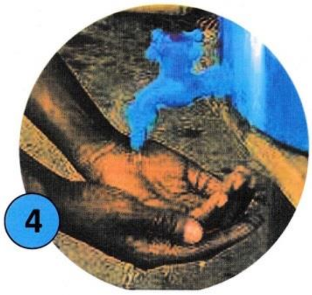

Wen u pull di plastic, was u wan ol an wit soap en wata

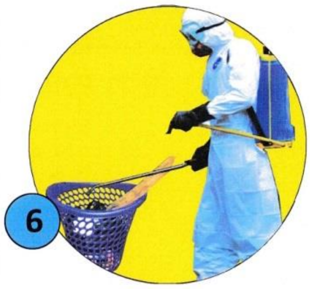

Wae di ambulance kam, duya gie dem all watin u use pan di sik posin, den go no watin do wit am

STAY SAFE AND PROTECT YOUR FAMILY WHILE YOU WAIT

Topic 12: This message addresses the need to provide practical guidance to families when somebody dies at home.

Audience: General population, especially where unsafe burials persist, both urban and rural

Channels:

- Posters distributed by NGOs, CBOs, DHMTs through PHUs, using local youth group, chiefdom councilors, and social mobilizers. To be posted at community gathering points, ataya base, court barrie, pharmacies, parks/bus stops, inside/on public transport, but NOT on people's houses.
- Leaflet (tri-fold, step by step pictorial) – combined with community house to house visits/meetings using own community members
- Plastic bags
- Radio discussion; Community meetings; Sermons – conducted by MoHS and partners; Pastors, Imams; youth and community leaders (male/female) as appropriate, and discussing the sequence of points presented visually in the leaflet.

Message:

1. If posin die na os, nor touch im bodi. [If someone dies at your home, do not touch their body.]
2. Call 117, bia wait teh den kam. [Call 117 and be patient while you wait.]
3. Beliv say wea di burial team kam, den go treat u posin wit respect. [Have confidence when the burial team arrives, they will treat your loved one respectfully.]
4. Duya lisin gud gud wan to watin di burial team di tell u en duya du watin den say. [Listen carefully to the advice that the Burial Team gives you, and follow what they say.]
5. Gie di burial team all tin wea di die man bin use en all tin wea u use pan di die man, den go no watin for do. [Be sure that they take the plastic and any soiled materials for disinfection or safe disposal.]
6. Tel u fambu en u padi dem [Pass this message on to your family and friends!]

Operational issues

Burial teams must continue to ensure that they are doing respectful burials:

- Consult with family
- Wrap properly
- One identifiable grave
- Respectfully putting into the grave
- Work with pastors/imams
- Workers not under influence of alcohol
- Respectful attitude of workers.
- Inter-religious councils and the Council of Paramount Chiefs to be informed and engaged

STAY SAFE AND PROTECT YOUR FAMILY WHILE YOU WAIT

Risk: Burial teams may not always conduct respectful burials and this can undermine community confidence in the Ebola response, while simultaneously increasing the chance of unsafe burials.

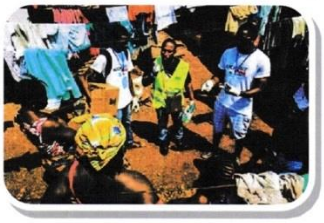

Tel u Fambul
en u padi dem

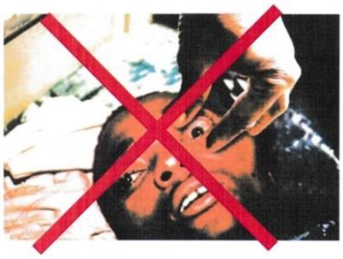

If posin die
na ose, nor
touch am.

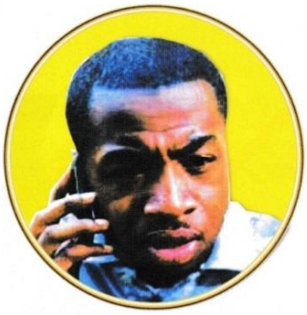

Call 117,
bia wait teh den kam.

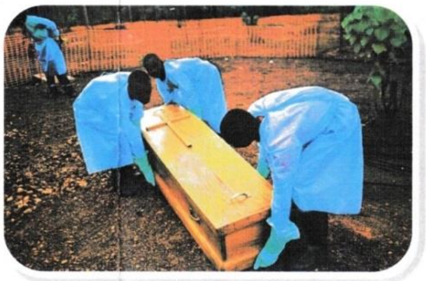

Beliv say wae d burial team kam,
den go treat u posin wit respect

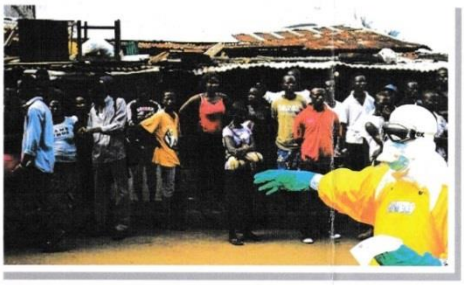

Duya lisin gud gud wan to watin di burial team de tell
u en duya du watin den say

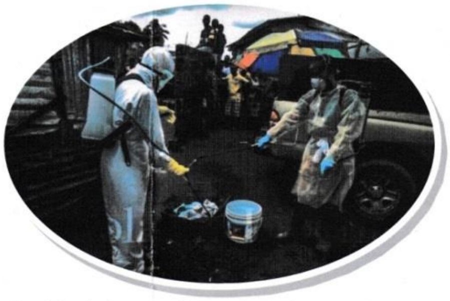

Gie di burial team all tin wea di die man bin use
en all tin wae u use pan di die man, den go no
watin for du

EBOLA DENIAL

Topic 13(a): Addressing doubts and disbelief. Our formative research indicated that many people who initially did not believe in Ebola, but who do believe in it now, have had direct experience of the disease. We want to make use of these people's experiences to convince other doubters of the reality of Ebola. This is a complementary message to the more visual 13(b).

Audience: General population, urban and rural

Channel: Testimonials/stories, tracking the speaker's evolution from non-belief in Ebola to belief, disseminated through:
- Face to face discussions
- Drama
- Radio jingle
- Button and/or wristband

Messenger: Survivors, health workers, religious and traditional leaders who previously hadn't believed that Ebola is real, but who now believe it because of their own experiences.

Message/theme of the testimonial: "You don't have to see someone with Ebola to believe that it is real" / "Noto pass yu see bifo yu beliv se - Ebola de."

Operational issues:

- Need to recruit survivors, health workers and traditional leaders who previously hadn't believed that Ebola is real to tell their stories. May be challenging to identify health workers to stand in public and say that they didn't believe in Ebola.
- Speakers need to be screened to minimize the risk of their perpetuating misconceptions to do with Ebola.
- When explaining why they hadn't previously believed, this could perpetuate their previous thoughts in other people's minds. Can be minimised by focusing on what made them believe in the end, not on the reasons they didn't believe in the beginning.

EBOLA DENIAL

Topic 13(b): Addressing doubts and disbelief, using the principle that 'Seeing is believing'. This is a visual complement to the more verbal Topic 13(a).

Audience: General population, urban and rural

Channels: Note the potential mutual reinforcement of these two channels together.

- Posters distributed by NGOs, CBOs, DHMTs through PHUs, using local youth group, chiefdom councilors, and social mobilizers. To be posted at community gathering points, ataya base, court barrie, pharmacies, parks/bus stops, inside/on public transport, but NOT on people's houses.
- Leaflets combined with community house to house visits/meetings using own community members

Person depicted in the poster/leaflet: Female survivor holding her certificate as she is discharged. Note that we did not have an image for this message during field-testing, so we discussed the concept and not an actual poster.

Message: *"*You don't have to see someone with Ebola to believe that it is real." / "Noto pass yu see bifo yu beliv se - Ebola de*."*

Operational issues:

- Possible stigma for the survivor being depicted, unless it's a cartoon caricature
- Recognition that this concept is difficult to portray in a poster for non-readers, and it will therefore likely only reach literate populations who can understand the text accompanying the poster.

117 PRANK CALLS

Topic 14: Up to 85% of all calls to 117 are defined as 'prank calls' – our informants also discussed this issue during the formative research. They recognised that such calls slow down the Ebola response, and they suggested that people will be more sympathetic towards 117 if they knew the extent of this problem. This message therefore aims to raise awareness of prank calls while also attempting to dissuade people from making them.

Audience: General public, urban focus

Channel: Jingle (radio, mobile PA system)

Messenger: Neutral, friendly voice both male and female in one jingle

Message: "Be a good citizen – don't make fake calls to 117, it disrupts the Ebola response." / "Lek Mama Salone – no mek kalo kalo call to 117, e de hambug di Ebola response."
