# Supplementary material for: Development of a set of community-informed Ebola messages for Sierra Leone
Source: PLoS Negl Trop Dis. 2017 Aug 7;11(8):e0005742. doi: 10.1371/journal.pntd.0005742 (PMC5560759; doi:10.1371/journal.pntd.0005742)
Supplement: S1 Appendix — (ZIP) [file pntd.0005742.s001.zip › Ebola messages - FGD and interview transcripts/R2HC Ebola Fieldwork 1/R2HC Ebola F1 COM-Rural1 V3 ADD PROBE.doc]

| CODE | **R2HC Ebola F1 COM-Rural1 V3 ADD PROBE**  **(rural semi-structured interview with community leader)**  **V2 15^th^ Feb 2014 - Probing added**  **V3 1^st^ March 2014 – Probing added, personal data added** |
| --- | --- |
| DATE | January 2015 |
| DURATION (minutes) | 25 |
| Collector nr | 6 |
| LANGUAGE INTERVIEW | Krio |

***PERSONAL DATA RESPONDENT***

| Age *(in whole years)* | 62 |
| --- | --- |
| Sex (F = Female, M= Male) | M |
| Religion | Christian |
| How much time does it take you to walk from your house to the nearest PHU? (minutes) | 20 |
| Mother tongue: | Temne |
| Education level: | Tertiary |
| Role in community: | Town Head – V3: Village Chief |
| Do you know anybody who had Ebola? | Yes |
| If Yes, what is your relation to that person? | Neighbour |

**TRANSCRIPT: (M= Moderator, R= Respondent)**

M: When did you hear about Ebola for the very first time?

R: “It was from May”.

M: May last year?

R: “May last year”.

M: When you first heard about it what were people saying about it?

R: “Well when it started people did not take it seriously because by then it has not reached here yet”.

M: Ok, then when you heard of the disease what first did you think about?

R: “Well, I first thought that it was a cholera outbreak”.

M: Ok, you as a community Head, in which way do you think this sickness has affected your community?

R: “Well, during that time myself it was a hard time for me and when I see that people were dying that was the time I realized that it was very serious matter and I use to go to the Primary Health Care for assistance but we tried to control it and it only affected one household, although there was denial”.

M: You are a leader, alright, why you think Ebola got spread in Sierra Leone”.

R: “People never believed that is a deadly disease”.

M: But what do you think can be a better way to stop Ebola?

R: “We should abide by the laws they have put that you should not greet a person, you should not wash dead body, do not touch somebody who is sick, immediately when somebody is sick let them report to the hospital. If we abide by the laws put this sickness will not spread in Sierra Leone. When you feel your body do not allow your family to come close to you again. Isolate yourself and try to find hospital where they can cure you. Do not say you are going to traditional healer that brought about the spread of this thing”.

M: Like which way do you think can be a better way to cure someone who has Ebola?

R: “Since we don’t know how this sickness came about, only the hospital can tell because when the hospital comes in they can determine. But it is about to go because there are people who can cure them”.

M: You said this sickness is strange; do you have any local name for it?

R: “When the thing became worst now, people got sick, when they go they will come again. So people were going the traditional way. Some will mix lime with honey and give it to them. I have also seen the person with the signs and symptoms when they gave him the lime and honey it cut off. He did not toilet again and he did not vomit again”.

M: Do you have any local term or name for it?

R: “Ebola”?

M: Yes, this Ebola?

R: “Well, we do not have any local name because local name some say in Temne “ka bom”.

M: What does that mean?

R: “When someone vomits and toilet”.

M: Ok,

R: “This is what we know; we have not got any local name that we can give because this is the first time for it to come to this country”.

M: Then do you know of anybody in this community who does not believe that Ebola is real?

R: “Still they are there, even though the thing has not affected them, except now but partially there are some who still do not believe that the thing is real. But we have seen the problem and we have seen the way it is killing people we believe that it is real. That is why we leaders were the first to believe that the thing is real because we make sure that anybody who gets affected we advise him to go to hospital. Note that we have started seeing the symptoms we too have been talking to them that the sick is real so let nobody over sight it. We were the first to believe that the sick is real, so we forced them to believe and they have seen the thing so what we are saying is true. Some were even abusing us, some nearly fought us”.

M: Ok.

R: “But we still persist that the sick is real”.

M: When this Ebola came they were sending out messages. Did you hear any of those messages?

R: “Yes, we have people who come to tell us how Ebola is like because they do give advert where they show everything that if you do this thing meet you these are the symptoms and these are the signs. People come here to tell us, “Well Body” (medical) people also come, Primary Health Care comes. So even us the local people we do organize ourselves to show the people who comes to the village”.

M: You have heard them? But do you think there is any one better than the others which people can practice and believe in?

R: “Yes because after I have explained there is one man from Health came and explain to us, he explain to us in detail. So after everything when they told us to avoid a sick person, when somebody is sick, even your child, your wife or your mother you have to avoid her for certain time, isolate her to see to it that, don’t touch, we should not greet our self, we should not wash dead body and we should not touch each other”.

M: You have said they are plenty but is there any one specifically that you think is a fine law that they put?

R: “It was fine law that they put, you should not play with a sick person. We have also put a law against washing dead body. We have seen that any group that washes dead body many people will die and you know that this is the kind of sickness that killed the person and we realized that it is working”.

M: It works?

R: “Yes it is working, if we avoid washing dead body the sick will not spread”.

M: Among the laws that they made, is there any one that you think is not working?

R: “Yes, even that greeting, some people are unable those some will say ay man you are my “fambul” (relative) this thing is not true. We have been trying it but we are unable. Some people even if you run away from him he will just take thing light and downplay it”.

M: As a community leader what do you we can do to encourage people that when their relatives are sick let them carry them to the hospital?

R: “Well, me too I used to come out. Some people used to hide, I myself used to come out to make a house to house visit, we mobilized people at first we were working alone but later we formed a Task Force before the coming of the Contact Tracers because we divided the town into groups so that we can go house to house since people were afraid to come out, except we force them to come out for them to go for cure, even up to this time”.

M: Like how many groups did you have?

R: “We had of about four (4) group”.

M: Four (4) group?

R: “Yes”.

M: Then up to this time when a person has Ebola, where do you think he can go? Will he prefer to go a “meresin man” (traditional healer) or goes to the hospitals that they are building for Ebola

R: “We here, we have CCC (COMMUNITY CARE CENTRE) ,

M: Ok.

R: “When somebody is sick here we refer the person to be carried, then they can carry him or her to the government hospital where they cure Ebola. For traditional healers, we condemned them, if we meet anyone try to cure somebody is Le 500,000.00’.

M: That is when the by-law came in?

Rl “when the by-law came in’.

M: But before that by-law, what was happening?

R: “Well, we were unable because before the by-law, when the problem was there when some of them go they will not come again. Any sick man they carry away they will report that the person is dead. So people were afraid to come bring their own people out. I know that they are carrying them secretly to go and cure them the traditional way. It is traditional healers that cure them. When people go to the hospital they would not return, so that is why they are not coming. Some are afraid to come out to go for cure because when his or her companion goes and does not come back, he too will be afraid to come. The person will prefer to go and find a traditional healer than they carry him or her away to die where they do not know him or her”.

M: Em, just like we have been saying, when Ebola came a lot of messages came out. Like you as a community leader, which way do you think we can try to let people know about the thing?

R: “Well is to continue to talk to them and tell the President plenty thanks for opening numerous Centres where they treat Ebola. So this has made us strong that when they carry a person the person will come back without anything happening to him or her and we too can be happy for the person. We still continue to them to carry people where they should carry them. But when it was not there, we were getting a hell of time to convince them to come out and go for cure. But as it now the sickness will end because even myself, these days I had a problem here, immediately I contacted the CCC (COMMUNITY CARE CENTRE) who carried him, right now he is been cured. I know that this will end because hospitals are now scattered all over the country”

M: When this Ebola business started you know that government put a lot of things in place for us to help fight the Ebla. Have you ever heard of any good or bad thing about the Ambulance Service?

R: “Yes”.

M: What are the good things?

R: “The good thing is somebody is sick they come and take the person away, the bad thing is when the siren keep blow over them. It reached a point immediately people hear the siren everybody will come out in grief saying that o they have carried him away maybe he would not come again. So therefore, because of the siren that blows over them, they are afraid to come out.”.

M: What about the Holding Centres and Treatment Centres?

R: “Well at first when they took them away, because people have the impression that they should not touch them, sometimes they languished and die. But by now when they brought the CCC (COMMUNITY CARE CENTRE) immediately somebody home with at home is sick you buy a plastic, cover yourself , buy ORS and give it to the person continuously until they come to take the person. So this thing has made people not to be too afraid now like before. So immediately when the thing affect a person or suspect that somebody is sick we go the traditional way until the Ambulance come to collect the person or we send the person to CCC (COMMUNITY CARE CENTRE) . I think from that time on to now the sickness has been reduced”.

M: What about the Burial Team?

R: “The Burial Team”?

M: Mhmm.

R: “Well at first the siren of the Ambulance on the Burial Team was disturbing when the Burial Team comes to take somebody and the plastic where they put people, we had brought in this of our town here they feel offended. But at first the way they were treating dead bodies was not fine. They strip the person naked to carry him or her to go and bury. One day there was a problem, a family feel offended that they did not treat the corpse fine. Well we the Chiefs who called the Ambulance the people have been abusing us, they have even jailed three people for their performance. But after that according to the Pa’s observations (the president) he decided that they should be dressing the body, put it into the bag and carry it. I think that has reduced the problem for us the Chiefs. They were bitter because they were not seeing where their relatives are buried. Because when somebody dies they just come and take it, they don’t know where they take it, but now when they come to bury in that respectful way, I feel that we the Chiefs do not have problem again and I feel they too are now used to it. Immediately when somebody dies they report it to us. Even if the person is born today and dies they will come to report to us that somebody has died there and we can call the Burial Team to come and bury the person so we too do not have problem again”.

M: What about the Ebola Line which they set up, that 117? Is there anything bad or good which you as a Community Leader would remember even if it has not happen to you but happened to Community people which they report to you?

R: “Like”?

M: Like the 117 Line, you know now when something happens they say call 117.

R: “Well, 117 we used to call them and they would respond to our calls but later when we called they won’t come. The other one I asked him the other day I said what happen? He said people call them and lie for them. Immediately when they call them when they go there they will not meet what they called them for. So that is why. Even these days I call 117 I was not able to get them, I called them they told me to hold on but I did not see them but they later came and carried the person”

M: Where was that?

R: “Here, it was two day ago, I think it was on Monday”.

M: You said you passed another way, which other way do you pass?

R: “Well, I called the section chief here, the Section Chief alerted the Centre in (- - name of the district headquarter town of the interview district - -) then they came and took him. But now we have Contact Tracers, they help us, when we have problems we put it to them.

M: They do it faster for you?

R: “They do it faster for us than ourselves, so any person who comes now. So in that area they have helped us”.

M: Do you have anybody who was Ebola affected in your Community who they carry away but has been cured? Which they call Survivor?

R: “Yes, I have, we have them”.

M: How does the Community respond to them when they come back?

R: “No, they really welcome them like their sisters and brothers because we told them. Whatever they tell us we too will call them and sensitize them. Immediately the person comes we call the Community and tell them that this person who they carried away is back, in fact they too admired because some people they carried them away and they did not come back. Even one elderly woman when they came back with her as a survivor the whole town gathered to go and see her and they prayed God for her to join us back in the family and she is still with us. So they are treating her as how we were before, they did not isolate her because she was sick by Ebola and should not join the Community, no! We still take her as part of us”.

M: Then, eh have you heard about any new medicine or a new way to cure Ebola?

R: “Yes well now because we have been seeing way to cure Ebola because most of the first people who went did not come back again that means that there was no method to cure them but at the later part now when they carry people they come back and that tells me that they have discovered a cure for Ebola”.

M: Do you know about that medicine?

R: “Well, I have heard about it but I don’t know about it. But when I see people go and come I feel that is true but I have not seen it with my naked eye”.

M: Have you heard about any new marklate for Ebola?

R: “I have heard about it but it has not come to this part of the country yet. I do hear about it”.

M: Like when you are a leader in this Community, I know that people look up to you, is there any question that people come to ask you about Ebola that you think they should know?

R: “Yes”.

M: Like which one?

R: “Like when they come to ask whether Ebola is real, even when we continue to tell them about the signs and symptoms of Ebola. Because there are some who do not still believe even we advise them they do not believe. Except when they come ask us we force them, that is why we keep the CCC (COMMUNITY CARE CENTRE) to tell the people not to do so. The thing is real we have been seeing it when you carry a person you meet others who thing has affected. So we trying to tell them they should take this thing serious”.

M: Then what do you think you should know so that they too can understand?

R: “Well, I doubt, when they give us those books we read them after reading them we interpret it to them that the thing is real”.

M: What do you think people should know about Ebola to be able to stop this sick?

R: Well, the good way is that when the thing affects a person and he or she is cured, and the other thing is although the thing is bad but it brings awareness to the people. Now the country was just going, no future, children were just dancing all the time. But now even merriment is reduced no sooner night fall everybody is at home. So, on the other hand, though the sickness is bad but it helped us, it is able to control the people”.

M: Ok Sir, thank you very much.

**ADDITIONAL PART OF INTERVIEW, OBTAINED BY COLLECTOR 1 AFTER CONSENT BY TELEPHONE:**

M:  In your interview with one of the researchers you did mention about denial, can you please tell me what you mean?

R: Yes the denial I was telling your researcher was the fact that our community people didn't believe that the sick kills people, if you can remember ,the issue that was broadcast about the burial team went to (- - name of interview village - -) to collect the corpse of Ebola victims, and stones were pelted on the burial team that came to collect the corpse, as I speak to you those that were involved were sent to prison, so that was what I refer to as the people denied , that this sick kills if you don't report early.

M: If I can ask permit me, chief, if you don't want to answer the question you have the right to say no I am not going to answer

R: OK, what is that you want to ask?

M: In your own view why do you think, the young boys in the village resist the burial team

R: so many factors are responsible for the actions they took to resist the burial team, one could be they don't want their loved ones to be buried in an undignified way, because that was how people reported that at the initial stage the burial team were not digging the grave as expected,  another one could be Muslims believe that , if a Muslim dies , he should be washed by a Muslim , buried by a Muslim and not infidels, as they reported that many of those  who are in the burial team do drink alcohol, and allowing someone who drinks alcohol to collect the corpse of a  Muslim is against the teachings of Islam , while if you look at the cultural aspect , you will find out that an active member of any society should be buried by those member of that society , but thank God these issues are no longer happening here now .

M: in your interview you keep on referring that some were abusing us, why were people abusing you?

R: As I told you Government has informed us that we should not bury any corpse, and whosoever caught doing so, that chief will face the full penalty of the law and since we are telling them to avoid , they said we have got money from government that is why we no longer protect them, even though we explain to them that this sickness is new to us , so let us follow the advice of the health officers in order  to put this sickness behind us, they didn't listen, they continued washing dead bodies and that made a lot of them are no longer here to tell the story, one house more than eighteen people died , so this was the reason , but now things have changed , they now accept that this Ebola is real and it kills.

M: did you hear any secret burial?

R: what I am aware of that they washed the dead body and call for 117 , but for secret burial I am not aware of it

M: How were you able to resolve the burial issue?

R: well thank God a lot of intervention have helped , one is the byelaws set by government ,and also the sensitization done by some NGOs to educate the Imams and pastors to preach about Ebola ,all these have helped us greatly

**ADDITIONAL PART OF INTERVIEW, OBTAINED BY COLLECTOR 1 AFTER CONSENT IN PERSON, February 2015:**

M: Why do you think has changed the mind of the people, from disbelieve to believe that Ebola is real?

R: “Well, during the time, when there was a problem, they were moving up and down, they saw some things that happened, which made not to believe. But we were also meeting with them, telling them, this is not a play, it is something that is true, and we were going round, summoning the community to a meeting and explained to them the dangers of the sick, so they have started seeing and realizing it. The section chief(- - name of chief- -), was the first person to know about this, people were telling us abusive words, but still we were telling them that the sickness exist, so they need to believe, so they said no, Government want to killed the people, we told them, the government needs the people, if they kill them now, how would they come back into governance, we were talking to them, but when they saw some problems at the same, they denied, but they got the believed now, they totally changed their actions”.

M: Is there anything specific you did, for the people to have this change of mind?

R: “Well there was a time when a person died here, they revolted against us, even the burial teams, so government took actions, the ones who took the active part in rebelling against the burial teams and the chiefs, and they were jailed, there was no judgement, they gave them twenty-seven months each in detentions, from this time, Everybody was cautioned that if we revolt again, so it may happened to us, so then changes started coming, I was not having any head ache again about this issue, because when someone to revolt, the person would be reminded of the people they jailed, they if you revolts they will do the same time to you, so I was relieved on the issue”.

M: What did they do for them to be jail?

R: “They revolted against the burial teams and the chiefs, they threw stones on us”.

M: Throw stones on you?

R: “They threw stones on us and the used abusive words on us unless we ran away”.

M: What was their motive of throwing the stones?

R: “They said they were not satisfied the way, the corpses were treated, they said the corpses are dressed naked, because of that, they revolted against us, they said we are not talking on their behalf, we have to fight for them, but we did not, like for me, unless I ran away, I was about to collapse because I am a hypertension patient, even the vehicle of the burial teams, I instructed them to run to the junction, they met us at the junction again, throwing stones on us. What help us that day was the soldiers that came to our rescued, they rescued the people, and it was over, so from then, the Local unit commandeer came and arrested three of the ring leaders and they jailed them, so from that time they have cautioned and they have even been supplying us rice”.

M: Chief, what is the role of the traditional healers in this Ebola response?

R: “Well they were afraid, when people were died, they were doing private issues to themselves, minus been a traditional healer, when you saw the symptoms, mixed lime and honey and drank, you will really see the changes”.

M: But specifically, do the traditional healers have any role?

R: “If the Government had said let no traditional healer healed any person, you will see, nobody will attempt to meet them, but if they met them, I don’t know, maybe they are doing it secretly, but, it is not happening now”.

M: Why do they stop?

R: “They stopped because they said, any person that is sick, should be taken to the hospital”.

M: Who stopped them?

R: “Well made the announcements ourselves, and we people went out to search for issues like that an anybody they find doing that will be arrested, so they have the fear that, this is serious”( *People talking at the background*)

M: If you are now an Ebola survivor, what do you think is the treatment given to you?

R: “From them”?

M: Yes?

R: “They have to be much closed to me, to let me feel at home, but if I am isolated, I will still have it in mind that I am sick, but if they come closer to me I will have the feeling that I am part of the community”.

M: Which problems would you encounter if you were an Ebola survivor?

R: “People should have isolated me and my powers would not be as much stronger to them, if I should be a sick person, the people will be afraid of me, unless I go closer to them.

M: How are they treating Ebola survivors in your community?

R: “The Treatment is fine, they are embracing them, they are living together, and they are even assisting people at the Community Care centre”.

M: Do you think this positive treatment of Ebola survivors is happening right across the other communities?

R: “Since I have not gone there and we are not heard any alarming issues, I thought is the same time, because it is something they are telling us, advertising it on the Radio, that is what should happened, we are following it up”.

M: Ok, I thank you very much sir.

R: oooh.
