# Supplementary material for: Development of a set of community-informed Ebola messages for Sierra Leone
Source: PLoS Negl Trop Dis. 2017 Aug 7;11(8):e0005742. doi: 10.1371/journal.pntd.0005742 (PMC5560759; doi:10.1371/journal.pntd.0005742)
Supplement: S1 Appendix — (ZIP) [file pntd.0005742.s001.zip › Ebola messages - FGD and interview transcripts/R2HC Ebola Fieldwork 1/R2HC Ebola F1 FGD-FEOLD-Rural2 V2 CORR.doc]

| CODE | **R2HC Ebola F1 FGD-FEOLD-Rural2 V2 CORR(rural focus group discussion)**  **V2 – 12^th^ March 2015 – correction personal data** |
| --- | --- |
| DATE | January 2015 |
| DURATION (minutes) | 50 |
| Collector nr | 4 |
| LANGUAGE INTERVIEW | Krio |
| **TYPE FGD** | Older Females |

***PERSONAL DATA PARTICIPANTS***

| Nr | Sex  (*F/ M*) | Age  (*in years*) | Education Level (*e.g. none, Primary, secondary, tertiary*) | Language (*e.g. Mende, Temne, Krio)* | Religion | Job / Employment (*how they earn their living e.g. farmer, teacher, trader*) | Role in community  (*e.g. youth leader*)  ANONYMIZED, ONLY AREA OF ROLE INDICATED |
| --- | --- | --- | --- | --- | --- | --- | --- |
| 1 | F | 48 | Secondary | Temne/Krio | Muslim | Trader / Farmer | None |
| 2 | F | 47 | Primary | Temne/Krio | Christian | Farmer | None |
| 3 | F | 49 | None | Temne/Krio | Muslim | Farmer | None |
| 4 | F | 51 | Tertiary | Temne/Krio | Muslim | TBA | None |
| 5 | F | 53 | Tertiary | Limba/Krio | Christian | TBA | None |
| 6 | F | 27 | Tertiary | Limba/Krio | Muslim | Student / Farmer | None |
| 7 | F | 25 | Secondary | Limba/Krio | Muslim | Student / Farmer | None |
| 8 | F | 28 | Secondary | Limba/Krio | Muslim | Trader | None |
|  |  |  |  |  |  |  |  |

**TRANSCRIPT: (M = Moderator, R= respondent, R1= first person responding to a question, DOES NOT correspond to numbering used in Personal Data!)**

M: How has Ebola affected your community?

R1: “Ebola?

M: Yes, how has it affected your community?

R1: “How has it affected you in this community?”

M: Yes, how has it affected your community?

R1: “It has done us something, because of our relatives has been destroyed here, we lost our uncles, our brothers; almost we lost four people”.

M: Yes, mama, how has Ebola affected your community?

R2: “This Ebola has made us to suffer our suffering is that the child of my elder sister just died in this Ebola then he left a problem, the children he left behind, this is a real problem, it is difficult for us”.

M: Yes, sister, can you add something to that?

R3: “Well we thank God because some of nothing is wrong \with us, my mother is alive, my father is alive except my sister”.

M: Yes Mama, you too?

R4: “Ebola is difficult for us. As I am sitting here my father is not alive and I did not see his grave and I don’t know about him and it is three months now when I have not seen him. My small brother also has all gone. It is very difficult for us our house is empty. There is no big one; all the big ones have gone”.

M: Yes my sister can you too add small thing?

R5: “Well, Ebola is difficult; it affected us because now you don’t have to go to people’s compound, you have to sit at home. You no longer go to (- - name of district headquarter town of the interview district - -) to find market; you just sit down and suffer. They came here the other day and took our uncle away and we did not see him again”.

M: Yes my friend over there?

R6: “We used to be with our sisters but since the outbreak of this sick we are no longer seen each other, you no longer go to peoples compound, you just have to sit at home, that one is difficult for us”.

M: Let the Mami too add something?

R7: “What has Ebola done is that when someone dies you cannot go to the burial, you just have to sit down, no more greeting you just sit down and listen to what is coming, that is what I know”.

M: Yes the Mami what can you add?

R8: “Just like what my mother said, they are my brother, they are my fathers, they just lost and we are here now crying. Right now to even take something to go to (- - name of district headquarter town of the interview district - -) they have stopped. We can sit up to two days or three days without going anywhere unto to this day, in that light Ebola is difficult”.

M: Yes Aunty, do you know anybody who had Ebola?

R1: “No”.

M: Yes Mama, you too have you seen anybody who had Ebola?

R2: “Well we have not seen anybody who had Ebola because they told us that we should …?.. accept anybody who we do not know?

M: Aunty have you seen anybody who had Ebola?

R3: “No”

M: Yes my sister?

R4: “Yes, we had seen one boy when they took him away and cured him and bring him back”.

M: Yes my sister?

R5: “Like what my sister just said is only that one boy that we have here went and was cured so he came back”.”.

M: What I mean, have you seen or heard of anybody who had Ebola?

R6: “That boy they took him but he came back we saw him”.

M: Yes, just like what they said they took him away and he came back, and is not only to be cured but also for supply that was what we saw".

M: Why do you think Ebola is spreading in this country?

R7: “Why is Ebola spreading?”

M: Yes.

R7: “Ee ah well you just see now people are just dying, we did not see the sickness but to us here we are just seeing people dying that is how see that Ebola is spreading”.

M: The reason, I want to know the reasons?

R8: “Is stubbornness, when they tell you that when somebody dies don’t go there, they said when somebody is sick don’t touch the person if you go there you will see Ebola. That is why Ebola is spreading”.

M: Yes Aunty, can you too add small thing on why is this sick spreading in this country?

R1: “Why is Ebola spreading? They told us that when a person is sick you should not touch him, and a person dies we should not wash id and they also told us that we should not go to burial, they told us that when a person is sick we should call for them to come and take the person but we are not agreeing that is why Ebola is spreading”.

M: Ok, yes my Aunty, you too can add small thing to that?

R2: “Is just the same about what my sister is saying, they said we should not touch, we should not wash dead body, when somebody is sick they should call for them to come and take the person. So is because of the stubbornness that Ebola is spreading”.

M: Yes my Aunty?

R3: “They said even if is your child when the is warm you should not touch him or her, we should call for them to come and take him or her but we are not agreeing to that we go ahead to touch our child do you want me to leave him or her to die, that is why Ebola is spreading in Sierra Leone”.

M: Yes my sister, do you have any reason that you can add as to why is Ebola spreading?

R4: “Ebola is spreading because of the stubbornness that we have, when they tell us we do not agree except we do it that is why Ebola is overcoming us”.

M: Yes my Aunty please add small to that ya?

R5: “We are causing it for ourselves because we are to what they tell us not do. They told us to wash our hands with soap and water and take care of the children”.

M: Yes Aunty, do you have anything to say?

R6: Ebola is spreading because when they said when a person is sick you should not go nearer to him or her, but here we always say this is my person I cannot leave him or her alone, all this is stubbornness, but again if you look at it is because of the sorry heart that we cannot keep to it”.

M: Do you have any local name for Ebola in your language?

R7: “Yes”.

M: How do you call it?

R7: “Ebolahe”.

M: What do you mean by that?

R7: “Ebola”.

R8: “The meaning in Limba is something which they go to buy”.

R1: When they ask you to stop and you cannot, then you are going to by Ebola”.

M: “Mhmm, yes Aunty you too want to say something?

R2: “Yes just like when they say you go to buy something, if they say do not go there, you say you are going there, and then you go and buy it and bring it for your family if you die who you blame”.

M: So that is why you call it that way en?

Rs: “Yes”.

M: Some people do not believe that Ebola is real; do you know such people who do not believe that Ebola is real?

R3: “No”.

M: What about you?

R4: “No I have not seen anybody”.

M: What about you Aunty?

R: “I don’t understand”.

M: Yes Aunty?

R5: “Ebola?”.

M: Yes my Grand Ma?

R6: “Ebola is real’.

M: You don’t know anybody who is denying that Ebola is not real?

R7: “No there are people who are saying Ebola is not real but anybody who does not believe that Ebola is real can say that but for us who have seen it we know that it is real”.

M: Those who are saying that it is not real, why do you think they feel so?

R8: “They have not touched it if they touched it yet”.

M: What do you mean you have not touched it yet?

R1: “Because they have not been affected by the sick that is why they are saying is not real”.

M: So can you give me some examples of those Ebola messages that you have been hearing? What are the Ebola messages that you have been hearing?

R2: “They come and tell us that when a person is sick we should not touch the person, you should not go near the person, when some dies we should not wash it, we should not touch it that is what they tell us that when a person dies we should not go to the burial when you go there you should not sit close”.

M: So what do you think about those messages that you hear?

R2: “For me, it is fine for me”.

M: Yes, Mama, can you give me some examples they have given you about Ebola?

R3: “They told us that this Ebola is coming from birds and beef that is where it came from to affect us in our country”.

M: So what do you think about the message that it came from birds, what do you think about that?

R3: “That is true”.

M: My Aunty can you too give me an example of messages you have heard about Ebola?

R4: “They said we should not eat bush meat which have been caught by trap or has died by itself, we should stop eating those things”.

M: So what do you think about those messages?

R4: “Yes, that advice is good”.

M: So what do you say about those messages?

R4: “They said we should not eat bush meat, we should not eat Baboon, or anything that is in the bush. We should only eat fowls and fish if we continue to eat that Ebola will be far from us.

M: So what do think about that message?

R4: “Ee, so, me too that is what I am doing, so when they came and advice us”.

M: What did they advice you?

R4: “That you should not touch a person who is sick; even if the person is sick you should not go to the burial when you continue to be close to each other you can get the sick and you spread it”.

M: What do you think about that talk?

R4: “That is a good advice”.

M: What about you?

R5: “The sick is reducing”.

M: Yes my Aunty, you too?

R6: “They said we should not touch each other and when morning comes you have to wash your hand with water and soap we see all that as a good advice for good health’.

M: Yes Aunty?

R7: “They said we should not greet each other, nobody now as you can see, you cannot shake hands, they told us all of this. On that part we tell God thanks for that’.

M: Yes Grand Ma, what have you heard?

R8: “Just like what the Mami said, they trained us there at the Barry (= community meeting place), the moneys, the baboons, and that half, half beef we should not eat them said we should not eat them and we have stopped that, if they kill it in the bush there except they go and sell it, we no longer, eat it we thank God for that”.

M: En, so what do you think about the messages they have been telling you?

R8: “Well we feel fine”.

M: Yes my sister what do you think about the messages?

R1: “We are happy 100% for that, that is what we are fighting for, we are happy for that”.

M: Yes Aunty, you with the red head tie?

R2: “We are just saying that we are happy that is what we have been fighting for”.

M: Yes my sister what do you think about the messages?

R3: “We are happy about it because it is for our lives it is fine”.

M: Yes Aunty, you too, with the red head tie?

R4: “We just want to say that we are happy. All those who have left, is out of stubbornness that is why they left”.

M: Ok, yes my sister?

R5: “They come here to give us the messages so that we can listen”.

M: Yes Grand Ma, the ways they tell you the message, what do you think about it?

R6: “We thank God on that part because if you do not understand something when they tell you, you have to take it”.

M: Yes Aunty?

R7: “We too we are happy because you have your life but when they tell us we don’t do it”.

M: So, I want to tell me, which message we can give to the people for them to take somebody who is sick of Ebola for them to take the person to hospital or to the Treatment Centre or Holding Centres?

R8: “If the person is sick you should take the person to the hospital for them to cure the person”.

M: What can you tell the person for him to have the courage to take the person to hospital?

R1: “You should tell the person to take courage to take the person to the hospital. When the person is sick do not allow the sick to overcome the person you should take her to the hospital, when you realize that the person’s temperature is warm we need to carry the person”.

M: Ok, yes Mama

R2: “What I made of that, when you have people at home, and you begin to see some changes you have to let the person go to hospital for the person to regain life”

M: Ok, yes my sister, what messages can you give them to give them courage and hope to take the person to hospital?

R3: “You have to sit with the person, talk to him or her; cajole the person so that they can take her for her to get medicine for him or her to get good health”.

M: Yes my Grand Ma?

R4: “In the past when someone is sick they would be afraid to go to hospital he or she will sit down until the sick gets worst but now if someone is sick they take that person to the hospital so that they can cure him or her”.

M: What message can you tell people to encourage them to take their sick person to the hospital?

R5: “What I will tell them is that when a person is sick, because right now that thing is difficult, if you have the courage to talk to the person to have the courage to go hospital that nothing is going to wrong with him or her”.

M: Yes Mama, can you too add small thing?

R5: “In the past we were afraid but now we are not afraid, if a person is sick they rush with the person quickly for the person to be cured”.

M: Yes, Grand Ma, what do you think you can tell people for them to have courage to carry sick people to the hospital?

R6: “Whether the person is your person or not when you meet a person in a way that is not used to be you have to courage the person and tell him instead he or she remain home and die, tell the person go to the hospital. They have told us that people have come to cure the sick. You should not just go to the hospital and they say it is Ebola and you become afraid to go. So for all that when you a person you have to courage the person to go and find medicine”.

M: Yes Grand Ma, what can you tells somebody to give him or her courage to take a sick person to the hospital?

R7: “Ah that is it. In the past when somebody is sick they took him or her to the farm and hide the person even when they have talk to us that if a person, even if it is today that fall sick you have to take him to the hospital to be cured?

M: Ok, so what do you think is the best way to give Ebola messages to people? Which way is the best way that we can take the message to the people?

R8: “Ok, is to call 117 and say someone is sick and is vomiting, his head is warm, and his head is warm”.

M: What I mean is that what is the best way to tell people about Ebola messages so that they can get it quickly?

R8: “You have to call”.

M: Yes Ma?

R1: “The best way if you have a sick person here you have to call, even when somebody dies we will call”.

M: Yes sister the best way for us to able to give new Ebola message to people?

R2: “When a person dies you should call quickly for them to come and take the person”.

M: The messages, which best way can we give it to people?

R3: “The only thing to do now when you see a person lying down, the moment you have to send to the Doctor in (- - name of location ??--)”.

M: I am talking about the Doctors; I am talking about the messages?

R4: “You have to tell them that when a person is sick you have to take the person to the hospital”.

M: How do we tell them?

R5: “You have to courage them”.

M: How to tell them?

R5: “You go and meet them and tell them they will not just say it is Ebola they will check the person first so that they will know whether it is Ebola or not Ebola”.

M: So, Mummy said we have to go and meet them, what about you what do we do?

R6: “Well that is just it when a person is sick you don’t have sit down”.

M: Yes what about you Mummy said we have to walk to meet then about you which other one what do you think we can tell them?

R1: “We, some time we have here we go and tell them that Ebola is less now but that they should take care of themselves since then Ebola started get less”.

M: Yes Mummy which other what do you think we can get the Ebola messages to the people?

R2: “Where you meet them you advise them”.

M: Wherever you meet a person, isn’t it? Yes Aunty you too

R: no is just the same like my sister said”.

M: No, talk your own your sister said her so you too day your own?

R2: “When you meet with your companion you have to tell her”.

M: What if you cannot meet with her she lives behind the mountains how do you get the message to her?

R2: “If she stays far, if you have a phone you can call her and advise what she must do to have long life”

M: Why do you think we have to use the phone to talk to the person?

R2: “Well maybe the person is far away and you do not have money to go and meet her but if you have Le 2,000 you can buy, credit and put it in your phone and tell the person who is far away so that you can talk”.

M: Yes Aunty, apart from phone, what do you think we can use to give people the message?

R3: “If I don’t have a phone when I meet with person who knows my relatives I will the person to go tell my parents that this sickness that is around when you meet with a person do not shake hands don’t do anything so that you cannot be affected by the sick

M: So if a person has Ebola where do you think the person will first go? Would the person go to a ‘meresin man’ (= traditional healer) or to the existing Health Centres or to the newly established Centre for Ebola?

R4: “The person will first go to the hospital”.

M: Yes my sister you too, where do you think the person will go first?

R5: “As for us we go to (- - chiefdom headquarter town with a CHC - -), there is where they carry people when they are sick”.

M: What are the good things that you hear people say about the Ambulance?

R6: “Well I cannot tell you about the Ambulance because none of my relatives have entered inside it yet”.

M: So you too haven’t you heard anything about the Ambulance? Bury people fine”.

R1: “We thank good they come and …?...

M: What about the bad things, do they talk any bad thing about them?

R1: “Non Ma”.

M: Yes my sister?

R2: “The Ambulance can come for a sick person and they go and cure the person, they also come to bury the dead, now they do bury fine”.

M: What about the bad things?

R2: “Well I have not seen them doing any bad thing”.

M: Ok, Mama, do you hear any good thing about the Ambulance?

R3: “Yes because when they come to work they do their work”.

M: Do you hear ant bad thing about them?

R3: “Well, I have not heard that yet”.

M: Yes Aunty you too, the Ambulance, have you heard anything about it?

R4: “Yes the Ambulance they used to announce about it that when the sickness was worst when they take people to Kailahun (=first district in Sierra Leone with Ebola cases) they were saying that on the way they do not give them water or food. There are some patients that need food but there will be no food so by the time they reach the patient’s life will be disturbed”.

M: What about the Holding Centres where they keep Ebola patients is there any good thing that they talk about them?

R5: “No”.

M: What about the bad things?

R6: “Well we say thanks to God, because those who go there they treat them fine”.

M: What about the bad things, people are not saying bad things about them?

R6: “I have not heard anything”.

M: Yes my sister?

R1: “Now when they carry a person they treat the person fine”.

M: Yes my sister over there?

R2: “Well I have not heard anything about them, it was only in the past when they carry somebody you will not see the person again but now when your person die it is left with you for them to bury the person there”.

M: Yes my sister, the Holding Centres do the talk good about them?

R3: “No”

M: So we want to talk small about the Burial Team, what do they talk about them?

R4: “Well the Burial Team at first when they come they will take the body and carry it and they would not cover it after burial, when they put the body into the grave they will not cover; they will ask people to go and cover it”.

M: Is there any good thing that they talk about them?

R4: “Yes”.

M: What do they talk about them?

R4: “No, that is not a good thing. They will only tell them that if they leave the body there we do not have the hand gloves to put the body into the grave but they have the medicine which they out in the body not but when they come they leave it and tell us to cover it, there is where we get the sickness that is why we do not attempt to do it”.

M: Yes Mama?

R5: “Well we used to hear that people used to molest the Burial Team but to us here we don’t have that habit because we know they are doing their job, they have sacrifice their lives?

M: So is there anything good thing they are doing that people talk about?

R5: “Yes, the good thing they are doing is when they come they touch the body and bury it, so I know that is a good thing”.

M: Yes Mama?

R6: “The good thing is when they come they sacrifice their lives, we don’t touch, we don’t go closer, they will enter the place and do whatever they are going to do, they will go and bury and they dress the body and go and put it into the grave”.

M: Is there any bad thing that you hear people say about them?

R6: “We tell the master thanks, there is no bad thing”.

M: Yes Mama?

R1: “Well no bad thing because at first when a person dies they will not pray for the person they will just come and take the person to the grave but when they come they ask for dressing, they dress the body fine and bury it fine just like we used to bury our people and cover it fine”.

M: Yes my sister?

R2: “Well the Burial Team has no problem”.

M: Yes granny?

R3:”The Burial Team, at first when they call and when they come they would not ask for anything but now when they come they ask for dressing if you have wrapper they can dress it so we thank God for that. At first they had a bad name”.

M: So do you know about the 117 line which government said you should call when somebody is sick? What is the good thing that you hear about that phone Line?

R4: “Well if a person calls 117 you can save your life”.

M: What is the good thing that you hear people say about them?

R4: “Now when a person goes there we can see the person and that is a good thing”.

M: Yes the Mammy?

R5: “Well the good thing that they do is to rush to come and take the person and carry the person if God says that person is not going to die the person will not die, if God says the person is going to die then the person must die”.

M: You don’t hear any bad thing about them?

R5: “No”.

M: Yes sister what can you tell me about the 117?

R6: “We thank God because when you call they will come”.

M: Yes Mummy?

R6: “Well by now when you call them they rush up to come”.

M: Yes my sister over there?

R1: “Is just in the past when they were not coming on time a body can be lying down for up to two days before they come for the body but now we say thanks to God”.

M: So you don’t have any bad thing about it?

R1: “No”.

M: Yes granny you too haven’t you heard anything bad?

R2: “No, we sat thanks to God”.

M: Ok, so my sister, have you heard about any Marklate (=vaccine) that is coming for Ebola?

R3: “No I have not heard about it yet”.

M: Yes granny, have you heard about any Marklate (= vaccine) that is coming for Ebola?

R4: “No, I have not heard”

M: Yes Mama, is there anything specific for people to know about this Ebola?

R5: “Well we have understood that Ebola is real, but it is the stubbiness that made many to die they did not believe that the sickness is real?

M: How do we tell them that this sick is real?

R5: “Like for us who are in the village here when you go to your companion you send her to your relative in the village to go and tell them that this sick is real and that let them take their time”.

M: Yes Aunty’.

R6: “You have to advise the person”.

M: What do you advise the person about?

R6: “To advise the person take time and not to touch another person, when a person is dead do not go there, when a person sick don’t touch the person, you talk to the person to go to the hospital”.

M: Yes my sister can you add small thing to that?

R1: “I will tell her to keep herself and take care of herself”.

M: Yes Mama

R2: “You see when you do not know something and they tell you not to do this”

M: What is it that you should not do?

R2: “Do not greet a person when you go to burial, do not shake hands with a person, when you hear that someone is sick do not go near the person advise the person that what they have said is true”.

M: So which way can we tell them?

R2: “When a person is sick you should not touch the person, you should not wash the person, the person would just be lying there.”.

M: So I want to thank you all for patiently coming here to discuss this thing for the betterment of our country. So like I told you we did not come with money to give it to you immediately but we hope that all the information we are gathering well be a better thing for our country. So I thank you all may God keep all of us alive, ya?

Rs: “Ok.
