# Supplementary material for: Development of a set of community-informed Ebola messages for Sierra Leone
Source: PLoS Negl Trop Dis. 2017 Aug 7;11(8):e0005742. doi: 10.1371/journal.pntd.0005742 (PMC5560759; doi:10.1371/journal.pntd.0005742)
Supplement: S1 Appendix — (ZIP) [file pntd.0005742.s001.zip › Ebola messages - FGD and interview transcripts/R2HC Ebola Fieldwork 1/R2HC Ebola F1 FGD-FEOLD-Urban1 V2 CORR.doc]

| CODE | **R2HC Ebola F1 FGD-FEOLD-Urban1 V2 CORR(urban focus group discussion)**  **V2 – 11^th^ March 2015 – correction personal data participants** |
| --- | --- |
| DATE | February 2015 |
| DURATION (minutes) | 60 |
| Collector nr | 4 |
| LANGUAGE INTERVIEW | Krio |
| **TYPE FGD** | Older females |

***PERSONAL DATA PARTICIPANTS***

| Nr | Sex  (*F/ M*) | Age  (*in years*) | Education Level (*e.g. none, Primary, secondary, tertiary*) | Language (*e.g. Mende, Temne, Krio)* | Religion | Job / Employment (*how they earn their living e.g. farmer, teacher, trader*) | Role in community  (*e.g. youth leader*)  ANONYMIZED, ONLY AREA OF ROLE INDICATED |
| --- | --- | --- | --- | --- | --- | --- | --- |
| 1 | F | 57 | Tertiary | Mende | Christian | Civil Servant | None |
| 2 | F | 43 | Primary | Loko | Christian | Business / House wife | None |
| 3 | F | 39 | None | Mende | Christian | Trader | Youth |
| 4 | F | 29 | None | Loko | Christian | Trader | None |
| 5 | F | 55 | Primary | Mende | Muslim | Trader | None |
| 6 | F | 47 | Tertiary | Krio | Muslim | Civil Servant | None |

**TRANSCRIPT: (M = Moderator, R= respondent, R1= first person responding to a question, DOES NOT correspond to numbering used in Personal Data!)**

M: In which way has Ebola affected your community?

R1**:** “Well, the Ebola is only recently that it has affected this of our community, this area which I know and even that it was the mother and the child and both of them are nurses but thank God they have returned yesterday, they are well and they came back”.

M: Yes Ma, can you too add something?

R2: “Well what I can say is that we tell God thanks for this area where we are for the Ebola. Because the place I used to know was over there where those Limbas are staying, there it affected four people there and also the nurse down there together with her daughter but thank God they are going to be free from the quarantine”.

M: Yes Aunty you too, in which way has Ebola affected your community?

R3: “In many ways, because it makes the children not being able to go to school, work is not going on normally. So those are the ways”.

M: Yes my sister, can you too tell us the way Ebola has affected your community?

R4: “In many ways”.

M: Can you give me some?

R5: “Because one, medical facility is lacking, now when people get sick they are afraid to go to hospital, then there is no school business going on and things are not normal any more like in the past when we things in common but now that is not possible so it has really affected our area”.

M: Yes my Aunty, can you add something to that as to how Ebola has affected you?

R6: “Ebola has made a lot of things to go backward, our businesses have gone backward, our children have gone backward. And plenty of people have lost their lives just because of this thing. So we don’t like it”.

M: Yes can you add small thing to that; how has Ebola affected your community?

R1: “Well it has made things very difficult, it has made the economy stiff, things are not coming into the country again if you go to buy anything in town it is very expensive”.

R2: “More if they say it is a lockdown”.

R1: “All prizes will go up”.

M: So Mummy, have you seen somebody or know person personally who has Ebola?

R2: “I have not seen a person with Ebola”.

M: Yes my sister you too,

R3: “Since they started talking about Ebola I have not seen a person who has Ebola”.

M: Yes Ma?

R4: “I have not seen it by myself but we see it in television but I have never seen it”.

M: Yes my sister?

R5: “We have heard but we have not seen it”.

M: Yes Ma?

R6: “I have heard but I have not seen yet”.

M: Yes my friend over there?

R1: “Me too I have heard but I have not seen”.

M: So, I want to know the reasons why has Ebola spread in Sierra Leone?

R2: “I really do not know how Ebola spread in this country, I don’t know how it came and how it affects but we are praying to God for it to end because we do not want it. Maybe it can be poor sanitation, the environment, then the compound in which you live you are supposed to be cleaning it I think that will help, say the area you need clean water, eat clean food and live in a clean place”.

M: Sister, can you too tell me the reason why has Ebola spread in this country?

R3: “Because the advise which they are given us we are not taking it, when they said we should not wash dead body, we should not be touching our fellow men but still people are still stubborn they are washing dead body , they go about touching people”.

M: Yes Mummy can you add small thing to that? Why has Ebola spread in the country?

R4: “Like us Sierra Leoneans, it is difficult for us to take advice. Some people even when you tell them not to touch they still go ahead to touch and that is not fine when they say “Don’t touch”, don’t touch”. And then some people have garbage in their compound and it is not fine. They always tell us to clean our compound and (when) you are ready you should wash your hand with soap and water. That is what I can add for now”.

M: Yes my sister, why has Ebola spread in Sierra Leone?

R5: “Is because people do not abide by the law’.

M: Which laws?

R5: “They said we should wash our hands with soap, we should not wash dead body, wear long sleeves, do not go where people are gathered. So those are the things”.

M: Yes Ma, can you give me reason why is Ebola all over Sierra Leone?

R6: “Is just the same thing, when they put a law not to wash a dead person then you go and wash a dead person, do not go to burial, do not go to crowd. So when you break the law you don’t know who has it, so when you go there you get it from the person and you come back you share it to other people so that is how it spread”.

M: Yes my sister can you add small to that?

R1: “Some people can get the Ebola and they know they have it yet they allow people to touch them, so obviously the thing will spread”.

M: When I was taking your personal data you told me that you have different languages that you talk. Do you have any way you refer to Ebola in the languages that talk? Like when you said you are a Mende do you have any way you refer to Ebola in your language?

R2: “Ebola is real”.

R3: “Mmm, mhm”.

M: They way you refer to it in your language?

R2: “O ho, “Hegbae nyamui gita, ar try wu....”

M: What I mean is that do you have any name for it in Mende?

R3: “Is just the same, Ebola”.

M: What do you mean by that word?

R2: “Hegbae nyamui?”

M: Yes?

Rs: “That this is bad bad sick”.

M: Why do you refer to it as a bad bad sick?

Rs: “Because it kills people”.

R3: “It doesn’t waste time even”.

M: Ok my sister do you have any way you call it in your language?

R4: “(*Speaking local language which Transcribers do not understand*)

M: What I mean do you have any name that you call Ebola?

R4: “Is just Ebola”.

M: So what I want to say is that, some people do not believe that Ebola is real. So you know such people who do not believe that Ebola is real?

R5: “No I don’t know of anybody”.

M: Yes my sister do you know of anybody?

R6: “No I don’t know of anybody”.

M: Do you know of anybody who does not believe that Ebola is real?

R1: “I don’t know about anybody but I know that Ebola exist”.

M: Yes my sister?

R2: “No I don’t know”.

M: Yes Ma, do you know?

R3: “I don’t know”.

M: So I want you to give me small, small examples of those Ebola messages that you have been seen and hearing. Which Ebola message have you ever seen or heard?

R4: “When they sing that when you come from toilet you have to wash your hands with soap clean”.

M: Is that the only message you have heard? Apart from the wash hand, have you heard any other message?

R4: “They also said you should not go where there is too much crowd, they also told us not to touch dead body”.

M: Sister, you too can you give me a small Ebola message that you have heard?

R5: “They do sing that we should not go where there is crowd, we should not wash dead body”.

M: Yes Mama?

R6: “Well the Ebola message which I do hear is that when someone is sick you call 117, so anyone who has Ebola has to call 117, and has to keep his or her place clean”.

M: Yes my sister, give me some examples of the Ebola messages that you have heard?

R1: “(*burst into song on Ebola*)” (……………………………...)

M: Yes Mama?

R2: “Well the message on Ebola is plenty because they said when somebody is affected by Ebola he or she must not have sexual intercourse because it can transfer to the other person then you should not go to a burial or where there is crowd those are some of the messages”.

M: Yes my sister, you have been smiling all along?

R3: “When somebody is dead you should not wash the person or touch the person you should call 117”.

M: Yes Mama, what is your personal view about the messages that you have just told me?

R4: “Well is a good thing when you come to interview us and to know our views and what we think about Ebola”.

M: What do you think about those messages, what do you think about them?

R4: “It is good”.

M: Yes my sister what you think about the messages?

R5: “Bravo”.

M: Yes Ma you too

R6: “Sweet”.

M: So apart from when you are saying the message is good, what about the ways by which they send the message?

R6: “Well is an advice to us”.

M: The ways in which they give you the messages: “

R6: “It is a warning”.

R1: “The way they give the messages”.

M: Yes Mama”.

R2: “It is good for those who are stubborn”.

M: Yes sister?

R3: “For us to listen that is why they give us the message”.

M: Yes Mama, the ways they are giving the message to you?

R4: “It is good, for us to put it into practice”.

M: Yes Mama?

R5: “It is good”.

M: So I want to know what do you think is the good message for us to give to people to encourage people to take their sick people to the hospital, particularly Ebola patients?

R6: “Well you have to encourage that person, talk to him; you have to give him the word of God for him or her to have faith. If the person listens to the advice and goes to take treatment’.

M: What can we tell people to encourage them to take their sick patient to the hospital especially Ebola patients to the hospital?

R1: “Well when you know that somebody has contacted Ebola, you just have to talk to the person and talk to him that, that is not the end of his life and try to call 117”.

M: Yes Mama, which words of encourage would you give to somebody to give him courage to take their sick persons to the hospital?

R2: “What I am saying is that you have to talk to the person, you encourage the person to come and see his or her family for him or her not to discourage that, that is the end of his or life’.

M: Yes my sister, which words of encouragement do you think we can give to people to encourage them to take their patient to the hospital?

R3: “Just tell them that they are not going to die, they are not going to kill him or her, that they are going to treat him or her, if he or she hasn’t got Ebola then they will release him to come back”.

M: Yes my friend?

R4: “You have to talk to the person fine”.

M: Which kind of find talk can you tell the person?

R4: “You have to tell the person that when he goes there they are not going do him anything. They used to tell us that when somebody is sick and goes to the hospital they would inject him but we have come to know that they were lying. So we thank God for that. So when you go can talk to him fine for him or her to call 117 for them to come and take him for treatment since it is free’.

M: Ok, so which best channels can we use for people to get Ebola messages?

R5: “Like what you are doing now to go house to house, encourage people and go place to place, talk to people and teach them about Ebola, the good and the bad after it”.

M: Ok, so why do you think we have to do the house to house talk?

R5: “Well is for this thing to end”.

M: Yes Mama, can you show me any other best way that we can use for Ebola message to reach people quickly?

R6: “Well the way which I think is simple is by using the musicians because some people need music, you know Sierra Leoneans like music, so they put it into music maybe some people will take to the advise’.

M: Yes Mama do you know any way which we can use for Ebola message to reach people quickly?

R1: “Like you are now plenty and you scatter all over if you can go house to house it will be fine because some people do not have chance to go to the radio and talk so is fine when you go to house to house”.

M: Why do you think the best way is to go from house to house?

R1: “Because some people do not leave their home, they are always at home so if you go to meet him or her that is the only time you can get him or her at home”.

M: Yes Mama?

R2: “Through radio”.

M: Through radio not so?

R2: “Yes”.

M: Why do you think we should use the radio?

R2: “Because even the phones now have radio so you can even sit at your market table and tune a radio station you must hear it’.

M: Ok, yes Mama?

R3: “Well I believe that when you when you walk around from Saturday to Saturday is better because at weekends not everybody goes to work or to do business he or she must be at home”.

M: Ok, so what I want to know again is that, when somebody has Ebola, where do you think the person will prefer to go first? Would he or she prefer to go to “meresin man” (= Traditional Healer) or would prefer to go existing Health Centre or to go to Ebola treatment centres, where would he or she prefer to go first?

R4: “He must go to the hospital”.

M: Yes, my sister if a person has Ebola where would the person prefer to go first?

R5: “The person should run to the Centres of the hospitals’.

M: Yes my sister do you think the person should go first?

R5: “The person should go to the hospital first, when he or she goes to hospital they will sound him or her and tell whether it is Ebola or not if it is Ebola then they can send the person to the Ebola Centre’.

M: Yes Mama?

R6: “The person first goes to the hospital”.

M: Yes Mama?

R1: “The person goes to the hospital for him or her to know whether he or she has Ebola”.

M: I want you to tell me the good and bad side of those Ebola Services. Which good thing can you tell me about the Ambulance Service?

R2: “Well sometimes when the Ambulance comes people becomes scared, it is heart-beating to people. It is good but it is not good because every day they hear the sound of the Ambulance to come and take sick people those who are hypertensive can be affected and get worried, so the best I can say is that if they are coming let them come in that quiet manner without any alarming, they come to the area and take the people, the Ambulance is good but the too much noise that the Ambulance makes does irritate people, they don’t like it”.

M: Yes Mama can you tell me small good thing about the Ambulance Service?

R3: “Because when they call them they do come. At first when I get the noise of the Ambulance my heart jumps so I used to be afraid as I hear its voice(=siren) I go away but now it ok, by right if they say there is Ebola patient here they should come quietly and collect the person. But when they come with the noise those who have pressure when they hear the voice their heart jumps”.

M: Yes sister, can you tell me the good side of the Ambulance Service?

R4: “When you call them they come at once to the person if it is dead body or a sick man they come and take it and carry it away”.

M: Is there any bad thing that you hear people say about them?

R4: “Is just the horn (=siren) which can be so sharp”.

M: Yes my cousin you too?

R5: “When they come they do well because when they call them as soon as they come they come with speed but the only thing is when they come with a heavy speed, they should less the speed a little’.

M: Yes my sister; the Ambulance which good things can you talk about the Ambulance?

R6: “Well when they come they treat the body fine because they care of the body they spray the place they will not allow anybody to go closer to the place. The only thing is the noise which they come with, is too loud and everybody can be afraid”.

M: What about the Holding Centre where they carry Ebola patients for them to await their result, is there any good thing that you hear people say about them?

R1: “Ah, I don’t know about those Centres but from what I hear people talk, some people say the Chlorine which they spray them is too much and then in the Past they said when they are carrying people they said there was no care, they were not looking at the people they would not go near to them”.

M: Yes the Mami, the Treatment and the Holding Centres, have you heard people say anything good or bad about them?

R2: “Well like Jui , those who go there say they are treating them well but like me when I listen Radio in the morning some say they treat them fine, they eat so I am glad for that because at first they said they would just go and leave them like that without giving them food, that was at the beginning of the Ebola but now they say they are treating them fine”.

M: Yes Mama, the Treatment and the Holding Centres, do you hear anything good or bad about them?

R3: “No I have not heard anything yet”.

M*: (Intruder interrupts)*

M: Yes my sister do you hear anything that they say about the Holding and Treatment Centres?

R4: “Except when they blow the horn (=siren) but I have not heard anything bad about them”.

M: So is just the good thing that you hear about them not so?

R4: “Those who have relative there Say they take care of them good them food’.

M: Yes my sister, can you add anything, the good things that you hear about the Holding Centre?

R5: “Well I don’t normally hear anything because I am a person who cannot listen”.

M: Mummy, what about the Burial Team, is there any good thing that they talk about them?

R6: “Mhmm, the Burial Team does not waste time when they come they will just toat (=carry) you and spray the place and carry the body away”.

M: Yes Mummy, the Burial Team, the good side and the bad side?

R1: “Like they said, the Burial Team has no time to waste when they come they will just carry it away, so whether it is Ebola now or not Ebola they carry you away”.

M: Yes Mummy, the Burial Team do you hear anything about the good side and the bad side?

R2: “Is just the same thing because at first when they used to come they will go and bury you without result when result is out at times it is not Ebola by then they would have buried the body but now they will keep you and wait for result when result is out if it is Ebola they carry you the Ebola way”.

M: Yes my sister the Burial Team?

R3: “They way they bury is not quite right, whether you are Ebola or not they just bury you the same way they go and dump all of you in one place but now when they are doing blood test and some other half things the Burial Team is ok now”.

M: So you have not heard any bad thing yet about them?

R3: “No”.

M: Yes my sister can you add something to that on the Burial Team?

R4: “I have not heard anything except at one time when we lost our child when they came to take him buried first before they brought the result; that is just it”.

M: So, Mummy, in this area do they do secret burials?

R4: “I have never seen it before”.

M: But you know that there some society people when they die a person cannot touch them?

R4: “We do not have society in this area’.

M: Ok so what about the Health Facilities, the Staff, what can you tell me about them?

R5: “I don’t know”.

M: Yes Mama can you tell me small thing about the staff who are at the Ebola Treatment Centres?

R6: “No, I have never seen them because I have never been there”.

M: Yes me too, I do not go nearer to those places, I don’t know anything about them”.

M: Yes Mama?

R1: “I have never heard anything about them”.

M: So how do people react to the Ebola survivors?

R2: “Well we have not got it yet, even the people that they quarantined we showed them love, it is the church and the community we all showed them love, we prayed for them and we gave them something, you know we courage them”.

M: Yes Mama, those Ebola survivors how do people react to them?

R3: “Well I am saying we have not ,got them in our area but those who were around us we lift them up in prayer and when morning comes we go down to them because there is the police, there is a rope between us and we do take our time”.

M: Yes Mama, how do they treat Ebola survivors?

R4: “They tell us to treat them well because they have been affected and they [are now] well so we should to talk them fine and embrace them and not go far away from them”.

M: Yes my sister can you tell me small thing as to how they treat Ebola survivors in this community?

R5: “They treat them well”.

M: Yes Mama?

R6: “They treat them well”.

M: Yes my sister?

R1: “They treat them fine”.

M: So Mama, have you heard about any Ebola treatment that would be available soon?

R2; “I have not heard anything about that”.

M: Yes Mama, haven’t you heard anything about that?

R3: “The one that is available now?”

M: No, the one that is to come soon?

R3: “Yes, a Marklate (=vaccine) ”.

M: What did you hear about the Marklate (=vaccine)?

R3: “They said they are coming with Marklate (=vaccine) for this Ebola”.

M: So what do think about that Marklate (=vaccine) ?

R3: “Well I think that it is a good thing”.

M: Don’t you have anything to worry about?

R4: “No”.

M: Because the one who have started taking it if they survive, if anybody takes it again he or she will survive, it is for our health”.

M: So, yes Ma. Have you heard of any treatment that they are about to give to cure Ebola?

R5: “No, I have never heard”.

M: Have you heard about any new way how to prevent Ebola?

R6: “I have not heard”.

M: Yes Mama?

R1: “No”

M: Have you heard about any new way to prevent Ebola?

R2: “Is just the same way”.

M: Which is it?

R2: “I have not seen it”.

M: Yes Mama, have you ever seen any new way to prevent Ebola?

R3: “Is just the same thing”.

M: Mama?

R4: ‘I don’t know any”.

M: Yes back to this Marklate (=vaccine), have you heard about any Marklate (=vaccine) that is to come for Ebola?

R5: “I have not heard about Marklate (=vaccine)”.

M: Yes sister?

R6: “I have not anything about that”.

M: Yes Mama?

R1: “I have not about a Marklate (=vaccine)”.

M: So what is the common point of discussion about this Ebola business, like when you are passing and you hear people talking about it?

R2: “Well as me I do not listen to discussion, from my office when I come I go to church and I come back I am inside my house”.

M: Yes Mama, what do people discuss about this Ebola business?

R3: “Is just the hardship, people are grumbling that things are difficult. Even me sitting here all my market is gone, things are not going fine”.

M: Don’t you know about anything that they say about this Ebola?

R4: “Normally people talk about education, it is now one year and the children are still sitting at home”.

M: Yes Mama, what do you hear people say about this Ebola?

R5: “Education has gone backward and things are difficult right now things are going normal’.

M: Is there anything specific about Ebola that you think people need to understand better?

R6: “Is for them to teach people more and tell [people more “.

M: What specifically do you want them to teach people?

R6: “The way this Ebola came, how it affected people, how did we get it in this country because wecnever had it in this country”.

M: So what do you think is the best to explain all these things you are explaining?

R6: “Is for them to teach people about Ebola”.

M: Is there anything that you think people need to understand about Ebola?

R1: “Just like when you are scattered all over, you go around teaching people. The people, it’s like they do not understand anything but when you talk to them, you teach them they can take the advice”.

M: I want to know one thing which you think that people need to know and understand about this Ebola?

R2: “Just like this wash hand business, we always need to be doing it, wherever you meet it at the Centre you have to do it”.

M: Like which way do you think we should be explaining this to the people for them to understand?

R3: “When you meet a person you have to explain to him or her that this is what he or she should do?

M: Yes my sister, is there anything you think people can understand about this Ebola?

R4: “Well the thing they should understand about this Ebola is for them to explain to people about this Ebola. Some people did not believe that they were going to get it not until when the thing got spread. I would like them to go over radio, and spread people around to talk about this particular disease because this disease has killed people”.

M: I want to know specifically, what would people like to understand about this Ebola?

R3: “Well like the check points that they make to wash hand people become afraid when they point the thermometer on them they shout. Even one elderly woman has to go hay wire for them not to point the thermometer at her face but we were able to coax her and explain to her why we were doing it, that it was for her own safety”.

M: So which way can we use to explain all these for the people to understand?

R4: “Well some people when they get onto them some can go hay wire except they talk to them for them to listen to what you are going to tell him or her”.

M: Yes Mama, we were trying to say which particular thing do you think people need to understand about Ebola?

R5: “(*Not clear)(………………….)*

M: What I mean is that, is there any specific thing that people need to understand about Ebola?

R5: “When you sensitize them about Ebola that will make them understand, it has to be in different languages if it is through Television, if it is Radio “.

M: Ok, I tell all of you thanks for spending your time to talk to you ya?
