# Supplementary material for: Development of a set of community-informed Ebola messages for Sierra Leone
Source: PLoS Negl Trop Dis. 2017 Aug 7;11(8):e0005742. doi: 10.1371/journal.pntd.0005742 (PMC5560759; doi:10.1371/journal.pntd.0005742)
Supplement: S1 Appendix — (ZIP) [file pntd.0005742.s001.zip › Ebola messages - FGD and interview transcripts/R2HC Ebola Fieldwork 1/R2HC Ebola F1 FGD-FEYOU-Rural1 V3 CORR.doc]

| CODE | **R2HC Ebola F1 FGD-FEYOU-Rural1 V3 CORR (rural focus group discussion)**  **V2 – 1^st^ March 2015 – Probing added**  **V3 – 11^th^ March 2015 – correction personal data participants** |
| --- | --- |
| DATE | January 2015 |
| DURATION (minutes) | 33 |
| Collector nr | 2 |
| LANGUAGE INTERVIEW | Krio |
| **TYPE FGD** | Female youths |

***PERSONAL DATA PARTICIPANTS***

| Nr | Sex  (*F/ M*) | Age  (*in years*) | Education Level (*e.g. none, Primary, secondary, tertiary*) | Language (*e.g. Mende, Temne, Krio)* | Religion | Job / Employment (*how they earn their living e.g. farmer, teacher, trader*) | Role in community  (*e.g. youth leader*)  ANONYMIZED, ONLY AREA OF ROLE INDICATED |
| --- | --- | --- | --- | --- | --- | --- | --- |
| 1 | F | 18 | None | Temne/Krio | Muslim | trader | none |
| 2 | F | 20 | secondary | Temne/Krio | Christian | student | none |
| 3 | F | 20 | tertiary | Temne/Krio | Muslim | Student | none |
| 4 | F | 21 | secondary | Temne/Krio | Christian | Student | none |
| 5 | F | 20 | secondary | Temne/Krio | Muslim | student | none |
| 6 | F | 20 | primary | Temne/Krio | Muslim | student | none |
| 7 | F | 19 | none | Temne/Krio | Muslim | farmer | none |
| 8 | F | 19 | none | Temne/Krio | Christian | trader | none |

**TRANSCRIPT: (M= Moderator, R= Respondent, R1= first person responding to a question, DOES NOT correspond to numbering used in Personal Data!)**

M: I am (name of moderator) from Freetown. One University in Sweden called Umea in collaboration with CHaRT Sierra Leone and the Medical Research Centre Sierra Leone (MRC - SL) have sent us to come and talk to you about this sickness called Ebola. We are here to talk to you about the messages on Ebola.

M: (- - interview code - -), Ok, we are coming to start now. The first question I want to ask is; This Ebola we have been hearing about, how has it affected this Village? Yes my sister, I want you to answer that part for me, how has this affected your community??

R1: “Is more about those students, likewise our sister who has got pregnant and our Aunties who have died”, (*Not clear*)

M: Yes my sister over there?

R2: (*Not clear*).

M: I am not hearing you.

R2: When this came to Sierra Leone, it has affected us greatly. Our Aunties have died and our friends. In one of our houses, people died there over twenty (20). So they are saying the way Ebola affects is that when one is infected blood oozes out of his or her ears, his mouth and nostrils, but the cases I have seen have not shown those symptoms”.

M: Yes my sister, do you want to add something to what she has said? How has Ebola affected this Community?

R3: “Some of them when they are affected by this Ebola, they vomit, they toilet”.

M: So how has it affected this Village?

R4: “People are dying, it killed our brother and sisters, our parents have died”.

R5: “Some have. Some have dropped out of school. Some, when school reopens

M: So how has this affected this Village?

R6: “Some have become orphans”

M: Please let’s talk one after the other”

R7: “Some of them, we now begin to imagine, when Schools reopen, what are they going to do to go school when they do not have parents to take care? How are they going to pay their fees? The uncle is dead, the father is dead “.

M: Yes my own sister, you who have just arrived, how has Ebola affected this Village? I want you to talk louder so that I can hear you.

R8: “It has affected us a lot”.

M: Mhmm?

R8: “It has killed our sisters, our uncle, so it has affected a lot”.

M: Have you ever seen a person who has Ebola and do you know somebody who has died of Ebola?

R9: “No, I have not seen any”

M: Yes sister over there?

R10: “I have not seen, but I have heard”.

M: Yes, have you heard or seen?

R1: “Yes I have seen”.

R: “As for me, I have not seen but I do get the kind of message that come out about people affected by Ebola”.

M: Has the person died by Ebola?

R2 “Yes, the person died of Ebola”.

M: Yes my sister, you haven’t said anything, have you seen or heard of anybody who has Ebola?

R3: “Me, I have not seen”.

M: My own sister, have you seen or heard of any?

R4: “I have not seen but I have heard of a person who died of Ebola.

M: Yes my sister, have you seen or heard?

R5: “I have not seen it, but I have heard people say a person died of Ebola. Some of them who are affected when they come and take them to the hospital they will not come again, they die there”.

M: So this Ebola, why do you think it is spreading in Sierra Leone?

R5: “Eee”, they said when somebody has Ebola, and touches another person they can transfer it, because they said the Ebola virus is easily transmitted when the person dies. The virus moves away from the dead person onto the person who is not yet dead. So when he or she touches it, it can transfer onto you”.

M: That is what you think is responsible for the spread of Ebola in Sierra Leone? Yes, my own sister, do you want to add something there?

R6: “What did you say?

M: I said, why do you think Ebola has spread in Sierra Leone?

R6: “They said to move from place to place; when people travel you might get it”.

M: Yes sister with the white - do you want to add something to what she has just said?

R7: “And they also said that we should wash our hands always, whatever we do we must wash our hands, but some are not doing it”

M: Yes my own sister in the black? Why do you think Ebola has spread in Sierra Leone?

R8: “They said when going to the market you should wear long sleeve so that you cannot touch another person, may be that person has Ebola if you touch him or her you can get Ebola”.

M: Yes, my sister at the back there, I have but heard your voice yet, how do you think Ebola has spread in Sierra Leone?

R9: “They said we should avoid touching each other, when sitting close we must avoid touching one another. Right now, a lot of things have been stopped. So they said everybody should stay at home until Ebola is finished”.

M: Yes my sister there close to you?

R10: “They said if a person dies we should not wash the body we should not wash dead bodies.

M: Ok, the other question is, in Temne, how do you call Ebola? Yes my sister?

R1: “Some call it “Kolbola”.

M: What is the meaning of that?

Rs: “Ebola that they call so”.

M: Why do they call it that name?

R2: “Some do not understand how to call the word “Ebola”, so they call, they cut it short and mispronounce it that way”.

M: Some people believe that Ebola is not real, are there people in this community who do not believe that Ebola is real? Yes my sister?

R3: “At first when people were not dying they said Ebola is not real, but now people are dying too much, in one house when one begins to die, they will die in numbers. This made them believe that Ebola is real. Now they are not denying that Ebola is not real”.

M: Yes sister, do you want to add anything?

R4: “No”.

M: But you were saying something?

R4: “Well, now we are seeing the way people are dying”.

M: I know you must have heard a lot of Ebola messages. Which of them have you been hearing? Or what do you think about the messages you have been hearing? Yes, sister with the white - do you want to add something? Are there people here who do not believe that Ebola is real?

R5: “Well, now we are seeing how people are dying.

M: I know that you must have heard plenty of Ebola messages, what are the messages and what do you about the messages you have been hearing? Yes sister with the white - you have been hearing Ebola messages not so? What are the messages and what do you think about them? Be it on radio, paper, which ones have you had? Let me hear from you.

R6: “I have heard”.

M: What did you hear?

R6: “I heard that when somebody dies of Ebola, you should not touch the person”.

M: Yes sister, have you had any Ebola message?

R7: “At first when Ebola came we just hear that when you are affected by Ebola you will just be vomiting and toileting (going to toilet frequently, diarrhoea), we thought it was Cholera but now they said when somebody is vomiting, toileting, has high fever then it is Ebola”.

M: Mhmm... So those are some of the messages you have been hearing? Sister closer to you, have you heard anything about Ebola message? Which ones have you been hearing?

R8: “That when a person has Ebola, they carry him to the hospital for treatment, him or her when he or she gets well, he or she has to wait for three months before any “Geh dat” (= sexual intercourse). If you fail to abide with the time they give and you go ahead to have sex with your partner, it will transfer again”.

M: So that is the message you have heard?

R8: “Yes”.

‘

M: So what do you think about that message? When they said if somebody has been affected by Ebola she must not touch his wife for three months, en (= isn’t it)?

R8: “Yes”.

M: What do you think about that message?’

R8: “Because when he comes back and you decide to touch him, then it is going to affect again”.

M: Ok, yes sister with the pink, it has taken long I have not heard your voice? Do you want to add something there?

R1: “They have told us not to eat bush meat; Baboons monkeys we should not eat them”.

M: Is that only thing you have heard?

R1: “Yes”.

M: Sister with the orange, have you heard Ebola messages not so?

R2: “Yes”.

M: Which ones have you being hearing?

R3: “Yes, we have been hearing Ebola messages. They said we should avoid body contact, all that can make us get Ebola so we should avoid that. They also told us on the radio that when a person dies, we should call the burial Team quickly to come and take the body for it not to sleep at home, they should come and take it for burial”.

M: Ok, I thank you, yaa, This other says, those who have sick relatives at home, which message can you tell them for them to carry their sick relatives to the hospital? Yes my sister?

R4: “I will encourage him or her and say to go to the hospital that nothing is going to happen to him or her. That he or she is going to get well and come back. So if he or she goes. You have to talk to him or her in way that would let him or her take courage to go hospital or you call the 117 to come carry him or her”.

M: Yes sister over there, you were saying something? Do you want to add to what she has said? If a person is sick, what can you tell the person for her to go to the hospital?

R5: “Is encouragement”.

M: How would you encourage him or her to do?

R6: “You will encourage him or her that when they take him or her, he or she is going to come back. That they are going to cure him or her. When once that is done, if a person get sick he or she will have the zeal to go”.

R7: “For instance, those who were first infected by Ebola, died. Anyone who is affected will not come back. So even they get sick they will not go”.

M: Ok, sister do you want to add anything? If a person is sick, what would you tell that person to go to the hospital?

R8: “I will tell him or her that you are affected by Ebola you don’t have to sit at home with. Because when you are infected it will spread. So I will encourage him or her to go to hospital. In the past, when somebody goes he or she will return, even one old woman when they took her she came back and she is still living. Some other when they carry them they did not survive, only one child came. So now with the coming of the white people know that when they go they will survive. So now everybody goes to the hospital”.

M: Yes sister over there, I see you bowing your head. Do you want to say something? So if a person is sick, what would you tell him or her for her to go to the hospital?

R1: “I will tell him or her to go and let them see to him. Some of them when they sick, they call 117 all by themselves”.

M: So that they can carry them away?

R1: “Yes”.

M: Ok, this other one says, for instance in ( - - Name of the interview village - -), if you have Ebola message, which way is the best way to send Ebola messages? Which channel can be used to send the message to the people?

R2: “In the past, they gave out phones (*note: they were given to female headed village/area committees that were linked to the 117 line and linked to the health centre in the area)*, if anybody is sick they can go and tell the person that somebody is sick for them to call 117 to come and take him or her”.

M:Mhmm, so like we hear from the radio, Television, which one do you think can take the message to the people quickly?

R3: “The town crier”.

M: Town crier hmm?

R3: “Yes”.

M: For instance, if somebody has Ebola, what do think that person can do first? Do you think the person will go to a “Mresin man” (=herbalist) or will go to the Ebola hospital to be cured. Where do you think the person will first go?

R4: “He or she will first go to the Ebola Centre”.

M: There will be the first place to go, “en” (=isn’t it) ?

R4: “Yes”.

‘

M: Why do you think he or she has to go to the Ebola Centre first?

R4: “Well he or she can be cured quickly. But if they carry him or her to the herbalist to be cured, maybe he or she will die there, but if they go with him or her to the Ebola he or she might live”.

M: Sister you were about to say something?

R5: “Maybe when they take him or her to the Centre they are going to check him or her whether he or she has the sick. If he or she has the sick they can cure him or her there, if he or she hasn’t, they give him or her some medicine and discharge him or her. So he or she will begin to go to the herbalist little by little”.

M: So right now if a person fall sick here, do you think that person will go to the hospital?

R5: “Yes”.

M: He or she doesn’t go to “Merersin Man” = (herbalist) for cure?

R5: “No”.

M: I know you must have heard about the Ambulance not so?

Rs: “Yes”.

M:Mhmm. You have being hearing good and bad things about the Ambulance en? My sister, have you heard any .good thing this Ambulance?

R6: “No, I have only heard about the bad thing”.

M: Ok, what are the bad things you have heard about the Ambulance?

R6: “Some of our Aunties who has Heart attack, when they hear the Ambulance, you know it has some sound it produces – wion-wion!! (imitating the hutting of the Ambulance Sirene)So some people when they heard that their heart beats”.

M: Yes sister this Ambulance business, have you heard any good or bad thing about it?

R7: “As for me, I take it that if a person is sick, her husband died”.

M: But you have not heard bad thing about it yet?

R7: “No”.

R8: “I have heard about it”.

M: Mhmm?

M: Sister over there, have you heard any good thing about it?

R1: “When the Ambulance is coming it put the horn on, so some who have problem it affects them. Some can be afraid. Now when they just come quietly and take the person away. So I know that when they put the person in there they will do him anything until they take him or her where they want to carry him or her

M: Mhmm, we want to talk?

R2: “They said when the Ambulance is coming; they spray it with Chlorine so people become afraid to enter the Ambulance, that is why they say let them hold them”.

M: Yes you want to add something sister?

R3: “At first, they will spray the motor car and also spray the place where they took the person. But now they don’t spray into the Ambulance, they only spray where you step”.

M: Mhm, do you want to say something sister?

R4: “No”.

M: Ok, have you heard about the Ebola Centres not so?

Rs: “Yes”.

M: Have you heard anything good or bad about the Ebola Centres?

R5: “Me, I know the Ebola Centre, they said the Holding Centre is where they hold people. But if you are sick and it is the sickness, they give you medicines to make you improve, if you cannot make it then, they can take you, they can call the Ambulance to come and take you”.

M: Mhmm, so that is what you heard? Yes my sister do you want to add?

R6: “I have not heard anything bad about the Centre”.

M: Ok has anybody heard about the bad side of it yet? Good or bad things about the Holding Centres? Sister with the Africana, it has take long I have not heard your voice o? Do you want to add something?

R6: “No”.

M: Sister with the pink, you want to add something? Ok the Burial Team, those who bury the dead, have you heard anything good or bad thing about them?

R7: “Yes, in the past when a person dies they will just come with a black plastic and put it inside and drag it”.

M: Who wants to add to that?

R8: “Because some of them when they die, they have something; that something come out of them so they said people should not touch them or wash them. When they come they will spray it all over and spray the plastic and when they are finished they carry ity for burial; they will not wait”.

M: Have you heard anything good or bad thing about the Burial Team?

R1: “When a person die they just come put it and begin to spray it. They also spray the house both inside and outside, they will spray all over the place. So, all that can affect people”.

M: So, (Moderator coughs) Have you heard any good or bad thing about the 117 call line? My sister over there?

R2: “No, I have not heard anything”.

M: You too, you have not heard anything about that number?

R3: “No”.

M: So, do you hear any good thing about hospital that were there before and the news ones now?

R4: “No, our own hospital we have not heard anything bad about it”.

R5: “Like hospital now when you take your child there they will tell you to go to the Centre”.

R6: “Except if the sick (Ebola) affect the child, but if it is any other sickness they can treat the child at the hospital before sending him or her to the Ebola Centre”.

M: Mhmmm, ok. In this Community, is there anybody who has been cured from Ebola?

R7: “Yes”.

M: Yes my sister over there, how do you react to people who have survived Ebola? How do you treat them?

R8: “We encourage them, talk to them fine and tell them about the thing that they should do. Like when they told him not to do have “Mami en dady bisnes” = (sexual intercourse) until after ninety days. I will Talk to them to adhere to what they tell them to do”.

M: Yes sister, have you, how do you treat people who have survived Ebola?

R1: “They said somebody survived Ebola, once he or she has been cured, he or she cannot transfer it again”.

M: Once the person has been cured even if he or she touches another person he or she cannot transfer it again. So they take them around to people that they have survived Ebola”.

M: Here in ( - - Name of interview village - - ), do you know about any way to cure Ebola? Or have you heard about any new treatment for Ebola? Or have you heard about any new medicine to cure Ebola?

R2: “Except that Malaria medicine which they brought for us (*note: there was a mass malaria drug distribution recently*), they said because many people dying now are because of Malaria, some of them is because of typhoid and when they come with the medicine they supply us and we drink it so the malaria cannot affect us that much”.

M: Sister over there, you want to say something?

R3: “No”.

M: Ok, have you heard about any new way to prevent Ebola? What did they say?

R4: “Because in the past, they closed the road to Freetown road. They said en…., when people move from place to place it transfers Ebola. One elderly woman (“Mami”), a house was quarantined in the village where she went to by (…name of??……….) not knowing that the house was an infected Ebola house. She contracted the sickness and now she is dead. So that is why they are saying let people stay where they are, you should not roam about, do not touch a person”.

M: Ok, that is what you have heard?

R5: “Yes”.

M: Ok, have you heard of any “marklate” (=vaccine) that cure Ebola?

R6: “No, we have not heard about it yet”.

M: In this (- - name of interview village - -) village what do you hear people say more about Ebola?

R7: “Sometimes, most people are saying that the sickness has disturbed their children’s schooling and some have been pregnated”.

M: So that is what they discuss in the village? Who again want to add to that?

R8: “In the past they used to give the children that “Captain Band” (= type of implantable contraceptive), but now they say they don’t want to touch a person especially to play with their blood. So a lot of the children are getting pregnant. Like us, they have disturbed our schooling, we are no longer going to school”.

M: So is that the only thing they talk about? Sister with the white, do you want to add something?

R1: “People are saying education has gone backward, look at us we are supposed to sit BECE (= type of exam), we are unable to sit now. At first people never believed that Ebola is real. People were vomiting and toileting and dying. Now anything happen to you they say is Ebola. So now they do believe that Ebola is real”.

M: Among all the messages you have been hearing about Ebola, are there people who do not agree with those messages or do not believe those messages?

R2: “Some are say they don’t care they can touch anybody”.

M: So they do ague about that?

R2: “Yes”.

M: Yes sister you want to say something? What do people argue about more on the Ebola messages? Is there anything the people need to know about Ebola? Or what do you think the people have not understood about Ebola?

R3: “I don’t like to see when they catch a person with blood oozing out from him or her. Even the ones that have died you have to take time”.

M: Who again wants to add to that? What is the doubt on peoples mind about Ebola? What do they need to know more this Ebola?

R4: “They also say that before a person dies he or she will have rash on his or her body. But plenty of the people who die do not show those symptoms”.

M: So that what want them to come and explain it more to you, “en” (=isn’t it)?

R4: “Yes”.

M: I thank you very much ya?

Rs “o”.

**ADDITIONAL PART OF INTERVIEW, OBTAINED BY COLLECTOR 1 AFTER CONSENT IN PERSON, February 2015, with two of the FGD participants:**

M: My sister, according to what you people said last, some people do not believe Ebola exists, why do they think this is?

R1: “It is because, according to the papers we were watching, they showed us plenty things some people do not believe, they said when a person is sick of Ebola, rashes appeared on the whole body, vomiting of blood, hosing of blood from the nostrils and was not happening to plenty people, so that was the reason people do not believe Ebola exists”.

M: Ok?

R: “But is real”.

M: OK, you mean the messages?

R: “Yes, through the messages”.

M: OK?

R: “According to the papers you saw rashes on a person’s body, vomiting of blood, hosing blood from the nose and most of the ones that have died from Ebola, I have not seen that symptoms from them, so plenty of them do not believe Ebola is real”. (*A vehicle passing by)*

M: The spray they said it affects people, what is the spray?

R: “According to the information, people said when the sprayed, it affects and torment people”.

M: What is the spray?

R: “When they went to collect a corpse for burials, they will spray the place where the dead corpse was laying and they will spray all over the room, because before when this Ebola started first, they were spraying all the rooms and closed the rooms, whilst at the same time people slept in the rooms, so they said that affected the people more which led to their death”.

M: Do people know what they spray?

R: “They said it is chlorine that they sprayed”.

M: So when they spray, what really happens?

R: “According to them, because I have not seen, according to their information when a dead body is picked up, they sprayed and sprayed, they will even spray the people and it was affecting them, so we heard that information that it led to the death of people”. (*A heavy noise of a vehicle)*

M: So when it affects?

R: “Led to their death, because they said the spray is not good”.

M: Do you know or seen a person that I have gone to the traditional healer for healing?

R: “No, I have never heard of that”

M: Not exactly this area, but you sat down with your colleagues young ladies and they spoke that somebody as went to traditional healer?

R: “To cure Ebola”?

M: Yes?

R: “NO, no, I have never heard of that”.

M: Have you heard of any secret burial?

R: “Secret burial”?

M: Yes?

R: “No”.

M: Like when a person is burial secretly not precisely in this area but other area?

R: “No”.

M: You have not heard of that?

R: “Like when somebody has die, then they burial the person secretly”?

M: Yes?

R: “Yes, we heard of it, so they told the people that secret burial is not fine they should stop it, so they stopped it and when someone died now they called the burial team”.

M: Do you know why they were doing secret burial before?

R: “Because some still do not believe the sickness exists, that was what happened but when they saw the plenty deaths of people they came to believed that the sick real and they started calling the burial teams, when some died”.

M: That was before now?

R: “Yes, first time, but right now, it is not easy again, when someone died, they call”.

M: What has brought the change?

R: “Because they are seeing the type situation, people were just dying, but now they have believed that Ebola is real”.

M: Yes, you were telling me of a man?

R: “Yes a man died one of these days on the street, they did not even touch him, until they called the burial people at around twelve o’clock, they picked him up and went with him, and nobody did not touch him, so the people have believe that Ebola is real”.

M: May the Lord forbid it, if you are an Ebola survivor, which problems do you expect to encounter?

R: “Well according to people that had survive and come, some of their colleagues that were embracing them and were talking together, that will not happened, as they saw them coming towards them, they will get up and leave and say, this is an Ebola survivor, but they advised them to stop doing that but I am well assured that many people have stop doing that”,

M: But this same situation, is it happening in the other areas about the survivors?

R: “Yes, I felt so”.

M: You got any information or you heard someone talking of it?

R: “No, I have not got any information”.

M: Ok, but I have you seen any survivor in this community?

R: “Yes, I seen”.

M: How are they treating the survivor?

R: “Well, since the survivor came, they welcomed the survivor welled, although we are not intimate but I am seen the colleagues of the survivor are walked together and embraced him”.

M: Thank you very much my sister, I appreciate.

R: oooh
