# Supplementary material for: Development of a set of community-informed Ebola messages for Sierra Leone
Source: PLoS Negl Trop Dis. 2017 Aug 7;11(8):e0005742. doi: 10.1371/journal.pntd.0005742 (PMC5560759; doi:10.1371/journal.pntd.0005742)
Supplement: S1 Appendix — (ZIP) [file pntd.0005742.s001.zip › Ebola messages - FGD and interview transcripts/R2HC Ebola Fieldwork 1/R2HC Ebola F1 FGD-FEYOU-Rural2 V2 CORR.doc]

| CODE | **R2HC Ebola F1 FGD-FEYOU-Rural2 (rural focus group discussion)**  **V2 – 11th March 2015 – correction personal data participants** |
| --- | --- |
| DATE | January 2015 |
| DURATION (minutes) | 24 |
| Collector nr | 2 |
| LANGUAGE INTERVIEW | Krio |
| **TYPE FGD** | Young females |

***PERSONAL DATA PARTICIPANTS***

| Nr | Sex  (*F/ M*) | Age  (*in years*) | Education Level (*e.g. none, Primary, secondary, tertiary*) | Language (*e.g. Mende, Temne, Krio)* | Religion | Job / Employment (*how they earn their living e.g. farmer, teacher, trader*) | Role in community  (*e.g. youth leader*) |
| --- | --- | --- | --- | --- | --- | --- | --- |
| 1 | F | 30 | None | Limba | Christian | Farmer | None |
| 2 | F | 27 | None | Limba | Muslim | Trader | None |
| 3 | F | 25 | Secondary | Loko | Christian | none | None |
| 4 | F | 20 | Secondary | Limba | Muslim | none | None |
| 5 | F | 20 | None | Limba | Christian | Trader | None |
| 6 | F | 21 | Primary | Limba | Christian | None | None |
| 7 | F | 27 | None | Limba | Christian | None | None |
| 8 | F | 18 | Secondary | Limba | Christian | None | None |

**TRANSCRIPT: (M = Moderator, R= respondent, R1= first person responding to a question, DOES NOT correspond to numbering used in Personal Data!)**

M: How has Ebola affected this community? Yes my sister let’s begin with you, how has Ebola affected this community?

R1: *(silence)*

M: Ok, yes my sister you want to say something?

R2: “Well how this Ebola has affected our community we are feeling it now because our younger sisters who were going to school to learn, Ebola has send them behind, works that were done by people normally right now everything is backward, money problem is backward, money is a problem, so that is how the Ebola has affected us the system is too rough in this Ebola”.

M: Yes, you nearer to her, you want to say something?

R3: “So me what I have to talk this Ebola that has come there is no money, there is no food to eat, so, me I have a problem my husband is dead, my father is dead, my uncle is dead, that is the only talk I can talk here, if I am just sitting like this I will not feel fine. That is what I have to say”.

M: Yes my other sister, how has this Ebola affected this community?

R4: “Ebola has affected us here. What I am trying to talk in the past everything was there but now everything has changed. In the past people used to pay Le 15,000 (16feb2015 about 3.40 USD) to go for Freetown but now you pay Le 25,000 (16feb2015 about 5.70 USD) to go to Freetown and where ever you reach there is check point, anywhere you reach there is a check point (where) they will test you (= *check your body temperature with infrared thermometer*). So even if you father ??ides?? you cannot go closer to him. If a person is sick even if the person is your mother you don’t have to touch her, we are doing this because of the law they put that we should not touch person even if that person is “your person” you should not touch her, you should go to the hospital, and you should call 117”.

M: Yes my sister, how has Ebola affected this community?

R4: “This! What I have seen that is bothering me is that right now all of our relatives, some are far away. They usually come here and meet but right now we cannot leave here to go and meet them and they too cannot leave to come and meet us here. Well that suffering is what that is bothering me. My mother is not here, she is far away and my father is dead, she used to send for me during December month but whether my mother is alive or not I don’t know, so that is what that is paining my heart right now, is paining me. That is all what I know”.

M: Yes my sister with burette, how has Ebola affected this community?

R5: “How has Ebola affected me? Because, right now I as a business woman I used to go to “Loma” (=Trade fare) but now with the outbreak of Ebola there is no “Loma”, where we used to go and try to make ends meet, is no longer there, so that is paining us. There is no business any more, things are just lying like that”.

M: Yes sister, how has Ebola affected this community?

R6: “This Ebola has affected all things, because anything we used to get free we are no longer getting it free again. Our freedom has “between ceased”, even if you want to go anywhere you will not have the chance to go. Because anywhere you want to go now they said you have to take pass (=permit to travel out of the area/district) and if you want to get the pass they said you have to spend money before you will get the money or you will not even be able to get the money, you do not have business but in the past wherever you want to go you can go free, but now nothing even if you want to go greet your person they nothing; how can we sit in this country?”.

M: This other question here says: has anyone among you here seen Ebola patient?

R7: “Yes”.

M: Yes have you seen somebody who has Ebola?

R8: “Yes”.

M: Who again has seen?

R8: “Yes”.

Rs: “All of us here have seen”.

R1: “I have heard but I have not seen”.

R2: “What about those who lost their parents

Rs: There is one (- - name of a male person - -) (*Respondents talking in chorus*)

M: Let’s talk one after the other. The other question says: why do you think Ebola has spread in Sierra Leone?

R3: “They are denying”.

M: They deny that is why Ebola has spread in Sierra Leone?

R4: “Is the...is the denial. Because they said one, we should not touch dead body. Two, sex but we do not agree, we do it”.

M: Who again wants to add something?

R5: “Like just how that girl said, is denial, because if they say don’t do this, when a person is dead don’t go there, then you took upon yourself and go there may the person who dies has a relative if you go there and happens to touch that person the sickness will transfer, you see. So, it is true this denial”.

M: Yes?

R6” “Yes my sister, this Ebola has affected us. All our sisters who were going to school because they have been seated (=sitting) for long, they have got pregnant’.

M: In this community, do you have any name for Ebola in your local language?

R7”: “Because we call it “Mbola”, the mothers call it “Aboli”.

M: “Aboli”, why do you call it htat way?

R8: “Is the “Mbola” they call “Aboli” because they do not know how to pronounce it. The meaning of that in Krio is “You going to buy it” and if you look at it, it is true if they say this person is sick of Ebola and go there to greet you are surely going to have your share”.

M: How do they call it again?

R8: “Aboli”, that is in Limba, in Krio it means “going to buy it”.

M: Yes my sister is there any other name for Ebola?

R1: “That is the only name”.

M: Some people think that Ebola is not real. Are there people in this village who think that Ebola is not real?

R2: “Well at first they used to deny, but by this time there is no more deny”.

M: So right now everybody knows that there is Ebola?

Rs: “Yes, because they have seen it with own eyes”.

M: I know you must have heard about Ebola messages, you must have seen them on paper; you have heard it in the news. I want you to tell me which ones have you been hearing?

R3: “I have heard that when you affected by Ebola you vomit blood, you toilet blood but a lot that are affected by Ebola does not vomit blood but they are saying that you will vomit blood, they said you can even have rash and all your joints will ache. But some of them who fell sick said that is not the way the sickness affects”.

M: So what do you think about the messages that you have heard?

R3: “We as for me I am just confused because they say that is how the sickness affects so I am just observing”.

M: Who wants to add something to what that woman has just said?

R4: “The first time they came, they came with a concert’.

M: What and what did you see in the concert?

R4: “They came and showed us how the sick begins, you will vomit blood, toilet, you have rash on your body. But when a boy got seriously affected by the sick they came and took him away they said it is Ebola”.

M: Who again has heard any Ebola message?

R5: “They came and thought us here about the sickness how it enters your body...”

M: How does it enter the body?

R5: “They said is according to the symptoms that you get, they said you begin to experience fever, you will have ache, your joints will also ache that should go to the hospital to go and do test”.

M: What would you tell somebody who is sick to encourage the person to go to the hospital?

R6: “You call 117 to come and take the person to the hospital”.

M: Which good message would you give the person, because as some people do not like going to hospital, what message would you give to that person for him or her to go to hospital?

R7: “Me, if the person is at home I will go , when I go I will ask for the person to neighbours if they tell me that the person is not well I will ask why has she not gone to hospital. So the people who are there will encourage the person to go to hospital so if the person has Ebola they can cure it”.

M: Yes me sister, if somebody is sick what can you tell the person to encourage the person to go to the hospital?

R8: “You just encourage the person to go hospital, should encourage the person so that he or she cannot be discouraged at heart and talk to the person fine and give him or her full encouragement”.

M: So a person has an Ebola message, how do you think the message can quickly reach to the people in the village?

R1: “Unless they call”

M: To call is it everybody in this village that has phone?

Rs: “No”.

M: So how can you get the message quickly to the people?

R2: “You call the people in a town meeting”.

M: So by that the message can reach quickly?

R2: “Yes”.

M: If person has Ebola what do you think the person will do? Will he or she go to the hospital or to a “meresin man” (traditional healer)?

R3: “The person goes to the hospital”.

M: Why do you think he has to go to hospital first?

R3: “Because the way the person feels and they way they see him or her will make them carry him or her to the hospital”.

M: Why won’t he or she go to the hospital?

R3: “They do not go to a “mrersin man” (=medicine man – a traditional healer) because right now wherever the person goes they will tell him that they cannot cure that sickness that is why they go to the hospital but they have the chance they will go there if God happens to take his or her life there then that is it”.

M: Yes, you wanted to talk something just now?

R4: “At first if you have ORS temporally until the person goes to hospital”.

M: So you believe that when somebody is sick he or she goes to hospital for cure?

Rs: “Yes”.

M: It is because of the fear. The “meresin man” (traditional healer) is afraid and even the sick person is afraid. Maybe of you go somewhere else they will tell you it is Ebola so is better to go to the hospital”.

M: So people no longer go to the “meresin man” again?

Rs: “No, in fact when some people see it that the sickness is pressing them they walk by themselves to go to hospital

M: I know you must have heard about this Ambulance. Have you heard any good or bad thing about the Ambulance?

R4: “Yes”.

M: Yes what have you heard?

R4: “I have heard that when taking the people to hospital they run with the person at break-neck speed and two again, when they want to put the person inside they spray inside, they say they even spray the person well by the time they reach with the person they spray would have affected the person, maybe he or she will not reach and will die on the way”.

M: So Have you heard any good thing about them apart from the bad ones?

R4: “Yes’.

M: My sister over there you wanted to talk something?

R5: “Is just the same talk”.

M: Which one is that?

R5: “When they come (to take) person”.

R6: “Then when they come to bury. When they come to take a person, they know that the person is a patient they will guide themselves they tie their noses, wear gloves and wear everything and they know that spray is too dangerous and wherever the person step they spray the place until he or she enters the “motor car” (= Ambulance). They know that the spray affect human being also. The patient has nothing to safe guard him or herself inside the “motor car” (= Ambulance). That is why the Ebola sickness is now worst in this country”.

M: Haven’t you heard any good thing about the Ambulance except the bad one?

R7: “There is also the good thing about them because some of them with respect and some do not come with respect”.

M: Lactating mother (=nursing mother), you want to say something?

R8: “At first they were not doing well, but now we thank God because now they come to bury and can do anything, now we thank God at first they were not doing well’.

M: What do you mean by at first?

R8: “Ay first when they did not understand they will just take a person and put him or her into the “motor car” (= Ambulance) and go ahead spraying. Well by now we thank God they can come and carry the person safely, there are times the way they come they can make the patient panic. Because as you see the “motor car” (= Ambulance) will begin to alarm (=put on the siren) so even you the patient will panic”.

M: Yes do you want to add?

R1: “No”.

M: What about the hospitals you have now do you hear any good thing or bad thing about them? The Holding Centres, the Treatment Centres are you hearing any good or bad things about them?

Rs: “No”.

M: What about the Burial Team, are you hearing any good or bad thing about them? Yes my sister with the black?

R2: “Well the Burial Team when they come to bury, we at first Ebola when Ebola was not around when a person dies they wash it fine and dress it but at least when they said they should not wash the body but they should dress the body even though they give them clothes to dress but they do not agree to dress the body. And our people do feel it, they do not dress the body and when they are ready to burry as if they are going to throw away garbage, that is what they do. After which they spray into the grave and spray the road they walk on wherever they step they spray there until they reach the grave and throw away the body if it like it lie down fine or bow down they don’t care, they will not even cover the body, they call people to cover the grave knowing full wee that they have sprayed and the spray is dangerous, they call them to cover the body, people who do not have anything to cover their body, they will just untie themselves and get into the “motor car” (= Ambulance) and go away”.

M: Who again wants to add to that?

R3: “Even with us here we used to argue them when they bury the man they did not over it and removed everything they were wearing and put it into the grave and told the people that were there that they should cover the grave. And the people said no saying that they are assigned to the work and they have the right clothes to bury maybe the man has the sick if bury him the sick will transfer they went into their “motor car” (= Ambulance) and went away. Those who were in the town went and cover again”.

M: Yes Aunty, pregnant woman, have you heard anything?

R4: “Just like what this girl said that is how not happened, when they put the body into the grave they left they did not touch it. Everybody told them to cover the body and they refused they did not cover it, those who were there were the same people who cover it, they left it and went”.

M: Yes, my Aunty there with the burette, have you heard anything good or bad about the Burial Team?

R5: “No”.

M: Yes me own Aunty at the far end there, the Burial Team, have you heard good or bad thing about them?

R6: “Is just the same thing that they have explained”.

R7: “They themselves who comes to bury are responsible for the spread of the sick maybe the person they come to bury has Ebola you might not know and you just come and throw the body away and you don’t know maybe the person has won’t it transfer? They too are responsible for the spread of the sick. Even in another village it happened there, when they buried the Pa they only returned after three days to cover the Pa, when the Pa would have burst inside the grave and the flies go there and fly back and die in a maybe a water pot standing nearby when the people drink that water the sick will affect them. Then they will call them again to come and bury and leave it like that again.

R8: “When that fly come from the dead body and rest on your body you will get the sick they said it is a germ, this Ebola is a germ, if that fly go and get that germ and bring it to your body you will get the sick”.

M: What about the 117 Line have you heard anything good or bad about them?

R1: “No”.

M: “Combra” (= nursing/Lactating mother) haven’t you heard anything”

R2: “Those ones when when you call them it takes long before they come”.

M: Is that all?

R3: “A girl died at ( - - name of another village in the area - - ) at around 7 O’clock, not until after 2 O’clock before they could come’.

M: Mhmm?

R3: “That lady strained for long”.

M: That number, haven’t you heard any good thing about it yet?

Rs: “No”.

M: Mhmm?

R4: “They are doing good, they are doing bad at times when they call them they don’t come there”.

R5: “When you enter there quickly you will come out safe if you meet everywhere slack well the way they mix you up when going that is it”.

M: What about the hospitals that were here before and the ones built now for Ebola, have you heard any good or bad thing about them?

Rs: “No”.

M: In this village, do you have anybody who have survived from Ebola?

Rs: “Yes”.

M: How do you treat such people? How do you behave to them?

R5: “We do not behave bad to them, we talk to them fine. Like when one person died to them when they quarantined they place we go to them we talk to them bou we do not near them we stand afar we fetch water for them we put it down, fetch wood for them go with it”.

M: What about those who had Ebola and they took them away they now cured and back in the community?

R6: “Yes we have one”.

M: How do you treat them?

R7: “We talk to him we, play with him fine, even the time when they came with him we were all happy because they way they carried him we all felt sorry for him. Everybody said he was not going to die. They brought him back at night, when they brought him some people were eating everybody converged to see him. Even now we play with him”.

M: So you accepted him?

Rs: “Yes”.

M: Have you heard any new way to cure Ebola?

R8: “Well the new way which we heard they said when you are affected by Ebola if you go to hospital earlier you can get medicine for it but if you allow it to get chronic there is no medicine for it’.

M: That is what you have heard?

R8: “Yes”.

M: Have you heard of any new way how to prevent Ebola?

R1: “Well the way which we heard about how to prevent Ebola is by washing our hands with chlorine or with soap that is why they supplied rubber buckets, all here there were check points when you come from any end you have to wash your hands. We used to put that surf is what we put inside the water. Even in the homes the rubbers are there, you should nlot touch, we no longer gather where in people will go about drunk’.

M: Yes, Lactating mother do you want to add anything?

R2: “Is just the same thing”.

M: Have you heard of any marklate(=vaccin(ation)) for this Ebola?

Rs: “No, we have not heard”.

M: In this village, what do people mostly talk or argue about concerning Ebola?

R3: Some what they argue about concerning this Ebola, they say they do not believe that it just the virus which enters a person’s body that kills a person. Some also say it is an arrangement so you see is too plenty, when sickness broke out at your place this person will be saying his or her own, this other one will be saying this and that, everybody is talking his own.

R4: “Some other people are arguing that the Ebola and the war which is better, some are saying the war some are saying is Ebola’.

M: Why are they saying so?

R5:”Only they know but we don’t know”.

R6: “Some are saying the war because when they kill your person maybe you can see where he or she is laid but now when they come and take a patient here except next world if ever you will be able to see”.

M: Yes you want to add?

R7: “When our uncle fell sick they went with him, he was just loaded on a bike and carried away from that day we only heard after that he is dead so we don’t know his grave”.

R8: “We don’t know where they flung him even”.

R1: “We don’t know where he was buried or whether he was even covered”.

M: Mhmm, it has taken a while I have not heard from you mhmm?

R2: “As for me, when my husband died I did not know where they buried him..

M: Did he die of Ebola?

R2: “Yes, that was what they said because I did not see. When my father also died, I don’t know where they buried him. Is only my uncle that I know where he was buried”.

M: So you do talk about those things in the village?

R2: “So that is why there is no believing that the people are been killed by Ebola, because if it is true you have to come with the suspect some time the only suspect they come with is to tell you that the person is dead and has been buried. Some say when they come to take the patient when they go with him or her they get into arrangement, they said when they go with the person they will draw the blood and go with it they said they don’t even take care of the patient in the hospital”.

M: What is it about this Ebola that is not cleared and you want them to come and tell you more about? What would you like to know about this Ebola?

R3: “Like if my person is sick and they come to take him, when they take him away for treatment they have to allow me to go and see where they are treating him, the room they are going to admit him to make sure that yes he is Ebola patient, I should go and watch the room where they are going to admit him then I can return now to tell the others that indeed it is Ebola but when they carry your patient even when you want to go there, they will return you. So we do not believe that they go to cure Ebola’.

M: Who again want to add to that?

R4: “Like for even me, I don’t believe, I don’t believe it was Ebola that killed my husband because if it was Ebola I could have got it because when he was sick I did everything for him so that is why I don’t believe, it was not Ebola”.

M: So is that what you want to know more? Why is it that your husband was infected but you are not en?

R4: “Yes”.

M: Well thank you very much ya?

Rs: “Mhmm.
