# Supplementary material for: Development of a set of community-informed Ebola messages for Sierra Leone
Source: PLoS Negl Trop Dis. 2017 Aug 7;11(8):e0005742. doi: 10.1371/journal.pntd.0005742 (PMC5560759; doi:10.1371/journal.pntd.0005742)
Supplement: S1 Appendix — (ZIP) [file pntd.0005742.s001.zip › Ebola messages - FGD and interview transcripts/R2HC Ebola Fieldwork 1/R2HC Ebola F1 FGD-MAOLD-Rural2 V2 CORR.doc]

| CODE | **R2HC Ebola F1 FGD-MAOLD-Rural2 V2 CORR(Rural Focus Group Discussion)**  **V2 – 11^th^ March 2015 – correction personal data participants** |
| --- | --- |
| DATE | January 2015 |
| DURATION (minutes) | 85 |
| Collector nr | 6 |
| LANGUAGE INTERVIEW | Krio |
| **TYPE FGD** | Older males |

***PERSONAL DATA PARTICIPANTS***

| Nr | Sex  (*F/ M*) | Age  (*in years*) | Education Level (*e.g. none, Primary, secondary, tertiary*) | Language (*e.g. Mende, Temne, Krio)* | Religion | Job / Employment (*how they earn their living e.g. farmer, teacher, trader*) | Role in community  (*e.g. youth leader*)  ANONYMIZED, ONLY AREA OF ROLE INDICATED |
| --- | --- | --- | --- | --- | --- | --- | --- |
| 1 | M | 45 | None | Limba | Muslim | Farmer | mosque |
| 2 | M | 35 | Secondary | Limba | Christian | Tailor | church |
| 3 | M | 37 | Secondary | Limba | Muslim | Farmer | youth |
| 4 | M | 46 | Secondary | Limba | Christian | Farmer | church |
| 5 | M | 49 | Tertiary | Limba | Christian | Teacher | church |
| 6 | M | 57 | None | Limba | Christian | Farmer | church |
| 7 | M | 78 | None | Limba | Muslim | Farmer | none |
| 8 | M | 56 | Secondary | Limba | Christian | Farmer | church |

**TRANSCRIPT: (M = Moderator, R= respondent, R1= first person responding to a question, DOES NOT correspond to numbering used in Personal Data!)**

M: When did this community first hear about Ebola?

R1: “July”.

M: Which year?

R1: “2014”.

M: That is last year?

R1: “Last year”.

M: Ok, any other person?

R2: “Yes Sir, I got the message from August 2014”.

M: Ok, is there any other person?

R3: “Yes Sir,

M: Mhmm?

R4: “(*Not clear,,, Cock crows*)

M: Apart from that, how has Ebola affected this of your community?

R5: “It has affected us badly because we did not understand what to do, a lot of people died”.

M: Yes Sir?

R5: “Eh it affects the economic activity, farming is not going on properly, people are unable to find money because people are not moving to go and sell, there are no rumours so things are really difficult for people”.

M: Yes Sir?

R6: “Emmm, to make addition to what this man said, because the worst of it now is concerning your girl children who were going to school.”

M: Mhmm.

R6: “Presently these children are unable to go to school and they way they are sensitizing them on the radio is not everybody that has the facility to have radio to put it down and tell his son or daughter to listen, so now the children are going out of control you see, so right now the children in the villages are getting pregnant too much because of this education that is not functioning, that is another big problem we have here”.

M: Yes Sir, I saw your hand up Sir?

R7: “Emm is the same contribution because is not everybody who has radio because there are some children a teacher can stand before them and teach but they would not understand how much it is on radio when he or she is not seeing the person who is talking or writing. So now it is very difficult for the children and even the boys some are now thieves, where there is communication between him and his companions he will forget many things the morning he goes to the farm and from there when he comes he will do what he is not supposed to do, because there is no school, but at the time when there was school going on; when the teacher retires, when he comes home he must have something to do, but when they are just like that there is no control’.

M: Yes Sir?

R8: “Is just the same thing, when Ebola came it was very difficult, you understand”?

M: Yes Sir.

R8: “But the only thing that can make Ebola go fine in Sierra Leone is the government needs to help us that wherever we are we should clean ourselves, we need to wash fine and after that we understand that this virus is water bound, they said the virus lives in water but we have to ask government to tell any village that has a stream nearby and they use water frequently to drink everybody should have concern that any water that you want to drink we have to boil it fine and after that the centre town (=central part of the town) here where we are - government must ensure that there is a hospital here so the surrounding villages would know that human being want to drink water; he or she has to boil it, there are some water that has this virus inside. so if human being uses that water without boiling that virus is going to affect him or her and the sickness will affect him or her more”.

M: Ok Sir, yes Sir?

R1: “We tell you thanks for coming”.

M: Yes Sir.

R1: “This sickness has given us hard time, we should come together to finish it”.

M: By God’s power.

R1: “If we come together in unity this sickness will get out...”

M: Ok.

R1: “but if we did not take advise they are giving us this sick will give us hard times because they have told us not to wash dead body, you should not touch a sick person if we did not keep those laws the sick will not finish”.

M: Ok, yes Sir?

R2: “Actually Ebola has us mostly in the villages, like when your relative dies at least you have to see where they bury him or her. But when our people here are sick they just take them away; we don’t know where they carry them to, how can we make ceremony for them when we do not know their graves? We are feeling it because it is this Ebola that has caused it. In those days they can bury a person when December comes or any other time you can be able to go and clear your mother’s grave or your father’s grave so that you can make ceremonies but now no ceremonies because we do not know where they buried them; we don’t know their graves so that is one effect for us. That is how Ebola has affected us”.

M: Ok, yes Sir?

R3: “Just like they are trying to say, this sickness is a hard time for us Sierra Leoneans and the world as a whole because really it is giving us hard times. Where we are feeling it more is about our school children or even the pregnancy business that is rampant in this country is because of this sickness. Because where schools were operating children were afraid that she has to sit her exams or sit her BECE (=exam) but just because of this Ebola business ( ………?................) and it is you and I that should come together to kill it totally in our country. So, especially this time round where it is now we should take more action and the advices which the medics gave us so that this sickness can leave us soon”.

M: Ok, then the other question is, why do you think Ebola has spread in Sierra Leone?

R4: “Yes”.

M: Mhmm?

R4: “The only thing is when you touch a person who has Ebola you are going to get the sick. You will not know that when you go to somebody’s burial you will get the sick that is it in this town. The sickness came from (- - name of another chiefdom in interview district - -) and entered here and killed three people here and that was surprising to us. But we and the government can put guarantee, one, like not to go to burial, you should not greet, maybe that can save us from this sick, Sir”.

M: Ok Sir, yes Sir?

R5: “However, to what the Pa (=the other man) is saying, this sickness like for us on the traditional way when the person comes from afar we don’t know where he or she is coming from being used to “grip” (=embrace) ourselves to say “hay long time” and we are laughing that is why when the white man came for his own protection he will not extend his hands to greet you; he will just say ‘Hello’, we never knew we felt they were making indifference on us because they are White and we are Black; behold it was because of this sickness, so we never knew that is why this sickness is spreading because this person can come from “yanda” (=somewhere) and you come and greet him or her that is how this sickness can pass (=transfer, move)”. (*Motor bike hums*)

M: Ok, yes Sir?

R6: “Ebola is spreading because people are stubborn people (that) do not believe, many people did not even believe that it is true that Ebola is real. Then the other thing is we had no strong medical facilities.”

M: Ok.

R6: “Even the Doctors, the Nurses who were trying they too had no understanding about it; that is why majority of them died. Then the first statement when they said Ebola has no medicine, so those who fell sick went into hiding; they were not going to hospital, so people hide with sicknesses from one village to another village when they knew they had the disease then they went and infected other people”.

M: Ok, yes Sir?

R7: “This sick that has come to our country is denial and “sorry heart” (= ……….), just like what our man has said because our tradition even when the person is dead there will be people at the parlour who keeps turning it over and over throughout the night from there they can take care of the body until they go to bury it and they are telling us that with this sickness we must not touch or go to take the sick; because this does not walk, we go for it all these problems has made this sick to spread in our country and even when it entered newly May 25th in our country as a whole many started politicizing the thing how it is about money making and this and that you know and by the time we get to believe that the sick is real; it has killed many people over 2000 and something. Denial and sympathy is what that caused this sickness to damage most of our brothers and sister, may God sorry for those who God have taken”.

M: By God’s power, then the other is - do we have any local name or term call or compare this sickness to like in our local language? Is there any name? Yes Sir?

R8: “This sick we have the kind in Limba which we can compare to this sickness. This sickness had come from 1960 they call it “Bomboroto”.

M: Bomboroto?

R8: “Yes”.

M: What does it mean?

R8: “When you are affected by that sick they will say that person is not fit to be among people you have isolate the person anyone who goes closer to that person will get the sick even if they want to give the person medicine it has to be a survivor who has to do it they call it “Bomboroto”.

M: Bomboroto?

R8: “Mhm”.

M: Yes Sir?

R1: “We can also compare to that which they call “Gbokitigbo”.

M: Gbokitigbo?

R1: “There are times it affects fowls. If that sickness is in (- - name of bigger village with a PHU in the area - -) and affects one fowl it will destroy the whole fowls here, in Limba we also call it “Gudutugu”.

M: Gudutugu?

R1: “Mhmm”

M: Ok, yes Sir?

R2: “In Limba we can also call it “Nunguru”.

M: Nunguru?

R2: “Yes, a virus in Limba we call it “Nunguru” if it enters you it will destroy your body and then later it will kill you. That is why we have this Limba program which they call “Yengetehba”.

M: Yengetehba

R2: “They use it to sensitize, they call it “Nunguru” that should not allow this Nunguru or virus enter us”.

M: Any other person?

R3: “Yes”.

M: Yes?

R3: “I want to make addition to Mr. (- - name of one of the previous respondents - -)’s contribution the easiest way to get rid of this sick is to give it that name “Gudutugu”

M: Gudutugu?

R3: “Gudutugu”, It means any Limba who hears the word ‘Tonagita gudutugu’ he will really know that it is Ebola’.

M: What does it mean?

R3: “Gudutugu means just like he said if the fowl in the next village has the sick and comes here all the fowls in this area will be affected so they call it “Gudutugu”.

M: Ok, yes/

R4: “Why did I say we must call it “Bomboroto”, it affects mortal man. If that mortal man is affected by that “Bomboroto” in this town everybody in this town will be affected and they will all die, but our people have had the understanding that when such a person is sick they carry him into bush where he will be cured. And they will go around asking who have had the sickness before so that he can cure him. If the person dies they do not wash him, they will not ‘kasankae’ (=wrap) him; they bury him in “burkburk” (=Ant hill). I am sure if they call this sick “Bomboroto” everybody will understand that, anybody in (- - name of the interview chiefdom - -) who knows about ‘Bomboroto” sickness in 1960 will know that that is how the sickness affects’.

M: Well there are some people who do not believe that Ebola is real, are they still living among you? Yes Sir?

R5: “All of us here believe that Ebola is real”.

M: You now believe that Ebola is real?

Rs: “Yes, because it kills.”

M: And you have seen it killed people? (*Cock crows*) all right. Yes Sir?

R6: “I will say the community people and Chiefs do believe that Ebola is real but few are still denying because we are hearing an information from inside (-- name of district headquarter town of the interview district - -) in (- - name of a section of the district headquarter town of the interview district - -) Section somebody died in a quarantined house instead of proving it, they took it and dumped it into a toilet so that they cannot say the person is dead until they prove people are still denying it. Well the deniers are responsible for making the thing to spread. Because if you believe something you will be able to prevent yourself, if you don’t (believe) in something that is how you make your death. Even the number of people who died in Sierra Leone here is as a result of that, just according to what my brother said. We here in (- - name of interview village - -) really do believe that the thing is real because only three people died, so we tell God thanks for that. We do not go to people’s burial, we do not touch ourselves, we do not keep ourselves to say we make any gathering, is just like this which we are doing and nobody can touch his companion and most of the villages people are there who do not believe and there is where the Ebola is existing”.

M: Ok, yes Sir, I saw your hand up?

R7: “Yes Sir, we are not or I am not talking about other places I am talking about (- -name of interview village - -) where I am, I have known actually, all of us have known that Ebola is real.’

M: Ok.

R7: “Because when a man got sick here the bike man who took him to hospital got affected too and he died so all of us now know that this sickness is real”.

M: Yes Sir?

R8: “Now even a small ‘pikin’ (= child), my child own of these days it happened he asked me saying “Papa, what is Ebola”? and the child when I said you too - what do you mean by this is Ebola? He said he heard that this sickness is killing people that you should not touch and you should not go to burial, thenI said well, that is why they say it is Ebola. So I do believe that and the community as a whole we have come together with the good idea that this sickness is real and it is killing so for now we tell God thanks the sensitization is going through and our people tell all of us get the sensitization that, in fact the country as a whole’.

M: Ok, then when this Ebola breakout we were hearing a lot of messages, people were saying a lot of things about Ebola, which one of them do you think is the better way that we can listen to - for us to stop this sickness?

R1: “Yes Sir.

M: Yes Sir?

R1: “When Ebola breakout government spent a lot of money but what we want the government to do for us let them stop spending a lot of money on the workers, some workers can go and tell lies to people to do so and so just for Ebola not to finish; when the month ends they pay him. They are not carrying the correct thing’.

M: So you are saying that people come and tell lies what they are supposed to come and tell people about the Ebola for them to aware they are not telling them.

R1: “As long as they are paying him money they are doing it”.

M: So are you saying that they are doing that because they want the sick to be around so that they can get more work?

R1: “Yes”.

M: Ok, yes Sir?

R2: “The best way I have seen, one of the best way is to train people just like you have come in this way you talk to people how Ebola affects, what are the signs and symptoms then the other way through the radio like (- - 3 names of local radio stations - -) when they do Ebola programs a lot of people tune their radios and they can hear”.

M: Ok, yes Sir?

R3: “What can bring this sickness to an end?”

M: Yes Sir.

R3: “Right now it is affecting us, we have heard that Doctors have come but for us here in this village we do not know where the Doctors are; that is one. Number two, even the medicines which they send, we, it is only God who cure us. One day they have not brought anything here, we don’t have supplies, I am not saying the one which they brought the last time, but they have never brought for us here, we don’t have medicines, right now close to us we heard that they giving them medicines but nothing for us. We are not seeing any Doctor to cure us. Let government remember that we are also part of the country”.

M: Ok, Yes Sir I saw your hand up?

R4: “What can make this sickness to leave our country is that we need more trained Doctors and two let us keep to the laws they have said. When you prevent yourself is good, you prevent yourself at the time when you are not sick. But even if a lot of Doctors come if we do not keep to the laws and keep to the prevention, which they tell us then it will be easy for this sickness in our country”.

M: Yes?

R5: “Well they are emphasising, but if we keep to the laws, we do not go to burial, we do not treat yourselves, we do not make public gathering where we can rub against ourselves even God will help us then by that time we will able to get Doctors who can tell us what is wrong with us and what is in our body. We hear that they do test on people but we in this village we have not seen any Doctor”.

M: Yes Sir?

R6: “What can finish this sick is that we should listen to the advice of the medics because however they said when you are sick you cure yourself, even when you are curing yourself if you listen to what the person who is curing you “that you should not do this you should not do that”. I believe that if you listen to Doctor that is how you cure yourself. So the best solution is that we are praying now that we put more action even what we were not doing when the sick was worst, is by now we should put more things in action so that we can be able to eradicate this sickness to finally leave our country but if you see we are not able to do that the sickness will always be with us and what this sick wants is that it is stubborn and always attacks someone who is stubborn but anybody who listens to advice and does what the medics say that sickness cannot make you friend at all and this sickness has told us more about hygiene business. This sickness has come, if you look at the other side it is good, the other side it is bad. I said so because it has brought more understanding, the kind of interaction we used to get like to “grease” ourselves, touch, touch, this and that, this has made that even after this sickness there is going to be a lot of changes in this country and to the life of we Sierra Leoneans who are going to remain”.

R7: “Yes Sir”.

M: Yes Sir?

R8: “So we thank God just like how this man has talked. What can remove this sickness from Sierra Leone is one (1) the poverty. Like here we do not have sufficient water to drink and two (2) we do not have hospital. The whole section this is the centre if the government want to help like this village it should build one so that people can go there to give them medicine. They can give one Nurse, one Doctor who is perfect one that can control us how to move, how to sit, so that anybody who is sick can go to hospital when he or she leaves there will go and tell the other what they have said. Like for us what they tell us here like the meeting we attended today, when Friday reaches when I go to the mosques I will tell them that this is what government have said, that to drive this Ebola this is what to do for Ebola to go far from us. But if you say you are not going to give us a Doctor, a perfect one it will be fine. But as for us in this Chiefdom since the outbreak of this Ebola we have not had any Doctor here and this section is the centre for the whole Chiefdom. Ok, tell me the truth, if that happens in two months, three months, four months to one year ok tell me do you think this sick will leave? If I have the sick and I went to greet the person who they gave the medicine, tell me true will that sick not go to the person. Ok if another one comes who they did not give the medicine and come here to keep time and I don’t have the medicine he will surely go with the sick. So please we want government to put it down heavily that any little village get this medicine so that the sick can go if they don’t do it will not cure”.

M: Yes Sir?

R1: “Please, this sick will leave us very soon we should have that faith because anybody who has the belief that God’s power it will heal, it will be so all of us Sierras Leoneans that this sickness is going to leave us from today to tomorrow it will leave us. This is hope we should get but we are not praying that let the sickness spread more or come to our country again when it is finished but they should advice the survivors because otherwise these people, the survivors they will be the ones to bring the sickness again that it will not finish so we would like....’.

M: Patience, I have to come to that side I will ask question on that. Is there any other person or let’s continue, not so?

Rs: “Yes let us continue”.

M: Ok, so we have heard a lot of messages on (*Cock crows*) the radio is talking it, people come and talk about it like myself here I have talked about Ebola. Which way is the fine way, the best way we can tell people the message for them to know about Ebola so that we can drive it out of our country? Yes Daddy?

R2: “This is fine, just like how we are continuing let them give us the medicine for us to know that government so that we finish thing for us, Sir. But for us here we cannot get that medicine here because for the treatment, to go to burial, let them give us medicine for this sick to leave us Sir”.

M: Ok Sir. Yes Sir?

R3: “The most thing that will enable us get rid of this sock is let us have perfect Doctors and they make sure that wherever they are two or three miles away let them be able to make Health Centre there and put a perfect Doctor there because even the medicines we are talking about is it used to kill them because they do not know how to use them. They said to take one after the other, one in the morning and one in the evening, but some take three, some take four so it used to affect them but if you have good Doctors or Nurses who used to supply those medicines and direct people how to take it, it will not bring affect to anybody. So the best thing is inside the last village let there be a Health Centre there and a perfect Doctor and a Nurse who can prevent the people”.

M: Yes Sir?

R4: “For us to fight for this sick to leave this country, the way what I have seen because presently there are people who are sick but are not affected by Ebola. At one time we heard it in the Media when one person goes to do a test the Doctor told him that he is positive as the woman felt discouraged and started to cry he told her that to give him Le 30,000 (15^th^ Feb 2015: about 6.89 USD) for me to make you negative so I feel that government has to look for Godly people who are sincere who knows that if the person is affected let him show that this is positive but if the person knows that he or she is negative let them remove him or her from there entirely so that we would be able to get the right information. But really the way the Doctors are too many, the Nurses are too many it will make this thing not to collapse. And we want this sick to end today, today. Some are praying for it not to end because people have turned it to be money making. So I think if they find Godly people, sincere people this sickness will end’.

M: Yes Sir?

R5: “For this sick to end we should still continue to like to go by what the President told us to do in his speech that we should not go to burial, we should not touch a person who is sick, we should not wash dead body then even this shake hands business we should stop it then we should wash our hands always with soap’.

M: Yes my daddy, I saw your hand up?

R6: “You say for this sickness to end”?

M: Mhmm.

R6: “You see for this sickness to end government is helping us and we need to help ourselves. Then we had one sick which is Malaria, even myself it used to affect us, you hear me Sir”?

M: Yes.

R6: “ Government is supplying this medicine for the Ebola let them also give the Malaria medicine, this Malaria is a bad sickness now, now if it affects me I will not be able to walk, I will not be able to eat, my stomach will dry heavily and that is the number two about this government but this sickness is affecting us (?too?)”.

M: Yes?

R7: “Just like what the Pa is talking many sicknesses are in our body but it has all been taken for this only sickness but we are not Doctors; how do we know what we are suffering from?

M: Yes Sir?

R8: “So we say thanks to Allah, just like what the Pa was saying. But this one is now difficult for our lives. A person can get Malaria, a person can get chronic Malaria, a person can get fever, a person can just sit and vomit but all these have been taken as Ebola. Even if a person has Malaria now when they carry the person we do not have real Doctor to prove will just say it is Malaria, even if a person goes there vomiting we don’t have real Doctors to prove that this is not Ebola, that is what that is creating difficulty for us in Sierra Leone and up to date we do not have Doctors who can prove that this is not Ebola; it is Malaria, this is not Ebola; it is gonorrhoea, is whatever sickness now they go with. Well now when they take that person away you will never see him or her again.”.

M: Yes Sir?

R1: “What will end this sickness?”

M: Mhmm.

R1: “Just like what I said before, we should listen to the advise then we should we should stop going to burials, we should stop shake hands then when your person is sick do not keep the person secretly, let him send the person direct so that they can be able to go and cure you. We should stop the secret otherwise the more we keep secret, the more the sickness will befriend us. This is what I am telling everybody “let us take the advise so that Ebola can leave us very soon”.

M: Ok, Yes Sir?

R2: “The other way we have avoid those secret societies to play ‘Gbangbani’ or ‘Bondo’ because through that again we do sweat and touch ourselves and other people, we have to leave all these things. Then we should continue this contact tracing, when something happens you call 117 or you advise the person to go to the nearest Health Centre”.

M: Ok, well you know that there are some people when they are sick with Ebola, where do you think they prefer to go? Do they prefer to go to a “Medicine man” or Traditional Healer or go to the previous hospitals or go to the Treatment Centres that have been established? Yes Sir?

R3: “Over the time now he can go to the Treatment Centre that they have built recently so that he can go and test him; if they know that it is Ebola negative but he has other sickness they can cure him but they see that he is positive and has Ebola then they can put him where the Ebola cases are, then they can begin to cure him again. He cannot go to a herbalist or no other hospital”.

M: Yes Daddy?

R4: “For me since this sick breakout we were no longer going to medicine man again because this sick can kill even the medicine man so if you hear that even the medicine man, it is this sickness that killed him, if you go and take another medicine man to cure you then is for nothing. The ones that we here (hear??) if anyone is sick he or she find there, Sir, if they say it is the alright if it not then we can know”.

M: Ok, Yes Sir?

R5: “I want to make an addition again about this herbalist business, eh, some people who are sick with Ebola or there are some who do not have sickness Ebola but can go to the Holding Centre, well they prove that person to be positive. But when once they take that person to the Centre is would not be easy again for them to handover that person to the family maybe that person could be able to treat that person because some people are used to ‘Fanke’ (= shoot with a witch gun) people especially in those village they do ‘Fanke’ people and that ‘Fanke’ is not used to injection. When you inject the person you have put a fire into the person automatically you have killed the person you see. So I think the herbalists, those who cure with native herbs let be drawn little by little for them to observe them for cases that not concern with Ebola especially when they go with a sick person whether it is positive now or negative now they will not free the person again”.

M: Ok, so you are suggesting that let the Medicine men be seeing what the medics are doing for them to know whether it is Ebola or not Ebola?

R5: “Yes Sir”.

M: Yes Sir?

R6: “No, I support that which Mr..(- - name of one of the previous respondents - -).said there are some sicknesses especially that vomiting and stomach ache some times in our own traditional way if you put something for another person if the person eats it the person will vomit and complain stomach ache until the person dies. So if they take that case and carry it to the Doctor, no Doctor will understand and he will say it is Ebola but they invite those herbalists to go and look at that person and tell whether he can cure the person so they can bring the person to him for cure”

M: Ok.

R7: “Especially like you have said it is true that a man can just see his companion passing then he takes the witch gun and ‘fanke am’ (= shoot him) after that ‘fanke’ (=shot) blood will be oozing from every part of his body, so if Doctor sees that sickness he will say it is Ebola but that Native Doctor who is a herbalist when he sees it no sooner he touches him and smells his hands he will know that the person has been hit. So he can take him to his hospital for cure”.

M: Yes Sir?

R8: “I support what these ones are saying about that part. Like me who is sitting here I was sick last year, somebody has to lay me down even to sit by myself I cannot sit down, I went to hospital and took there one month and eight days. The money which my child spent besides food and transport what I was able to estimate is about Le 550,000 (15^th^ Feb 2015, about 126 USD) just on medicine..”

M: Aaaa!

R8: “When they pulled me and brought me back there was no betterment but as I am talking to you here today it was this native medicine was what they did for onto two weeks and I was able to get up and sit down. So it is not a joke when they talk about people who go about poisoning another. It is good that even they the herbalist let them have a corner at the hospital to investigate which type of sickness, if they know that the sickness cannot be cured at the hospital then they can tell the Doctor to give them the person so that they can go and try the native way. Because when you go and say it is not Ebola they will arrest you then they say you are the ones who do not believe that Ebola is real. So people are afraid, even one of our brothers who died at (- - name of village in interview district - -) they took him to the Ebola Centre and they found out that it was not Ebola, but they are afraid to say “give him to me, let me go and cure him” and they went and tested him he was negative but there is no way to get him out because people are afraid to go and say that the man does not have Ebola until the man died. So it is a good suggestion for the herbalists to be near by the hospital, Sir”.

M: Ok, Sir, yes Sir?

R1: “Again to add to what we can do to stop this Ebola..”

M: Mhmm.

R1: “It is easy when a person is sick here they carry him away to go and test him; if they treat him for at least 8 days and there is no improvement let them give the person to the people to come go and cure him. So we can stop this Ebola but he remains there until he dies; they will say it is Ebola that is how we are promoting this Ebola. Let them go and test the person, check for other sicknesses if it is not it let them discharge him. That is the only way they are promoting this Ebola because when someone goes and ‘fanke’(=shoots) his companion and disturb him and when they do not know by the time they try to do test on him, he dies and they can say it is Ebola. So instead of the about promoting this Ebola, Ebola when a person is sick, if they test him, it is not Ebola let them leave the person to return to the traditional healers for them so that they can try. That can make us stop Ebola. But if we say whosoever die there - they say it is Ebola, so how can we know now?”

R2: “And in addition to what he is trying to say even if when you go there they delay, they can draw somebody’s blood and it will take four, five days they would not be able to produce the result and life is life. So when you go and take somebody’s blood to go and do a test you should not waste time. If it takes a day that means it has taken a long time you cannot bring the result back, it is so, so but if they could not bring the result until after days by the time they bring the result the person would have died’.

M: Yes Sir?

R3: “Tell those who are helping us that?”

M: Yes Sir.

R3: “That this thing you should not just leave it like that those who have been affected by the sickness there are some children you cannot block them not to do (= have sex) it until after three months some are unable.”

M: Ok, Sir patience I will come to that side, I have a question, is two of you who have now that talked about that. Ok, when this Ebola business began government did a lot of things, they provided Ambulances to come, they provided Treatment Centres and they Have this Burial Team and the telephone line which is 117, but let us do it one by one what do you think is good or bad about the Ambulance drivers and the Ambulances that come to take people here? Yes Sir?

R4: “Well for me now the bad side of the Ambulance.”

M: Yes Sir?

R4: “They ove speed that is what I see that is bad, they do over speed that even the person is sick the beds keep on hitting him or her. If they are taking someone from here to Kenema (= one of the first districts in Sierra Leone with Ebola cases and Ebola treatment centre) the way they will ‘bukaneh’ (= gallop) you, by the time they reach with you, you would have collapsed. So this is bad”.

M: Yes Sir?

R5: “As for me it is the Ambulance that has all the problems in this thing. They close all the windows, they won’t open it, they have some medicine which they spray, that chlorine when they spray that inside the motor car by the time they reach, that he would died. All that Doctor did not sign so, the Doctor said when you have a sick person inside the motor car you should not gallop the person, you should not hit the person until you reach at the hospital but now when the Ambulance comes they winch all the glasses and pump the chlorine inside”.

M: Yes Daddy?

R6: “One man loaded our children here to carry them to Kenema (= one of the first districts with Ebola cases and Ebola treatment centre in Sierra Leone) there were three drivers as they went they kept galloping; the other held fast to the rope but the three drivers could not and they died because of the gallop, when they asked them they said they were going fast so that they can reach quickly. Well if you are running with any person who is sick and you don’t check for gallop, won’t you die on the road? Number two, those people who they bury...”

M: Wait and be patient, ya?

R7: My observation again on the Ambulances is that they delay to come and collect somebody. Like if there is a sick person here and they call them, they will begin to delay to come and collect the person. Like here now if there is sick person and call them they will continue to delay till the people will give up. They will tell you they are coming but they will delay until and this life we are not the owner of it and we believe that Ambulances are now in our country, so I believe that they should always be alert as they get a call then they should know that there is a case there, so at least they should be worried so that when they come and carry the person quickly, I think they will be able to go and know how to solve this problem. But the delay is what I have observed from them”.

M: Yes Sir?

R8: “At first when this sickness began they were making it too fearful because wherever they go they blow the siren now so that people can know and get fear and some were not happy to get into the ‘motor car’ (= Ambulance)’.

M: Do you we have anything good that we can talk about them?

R1: “Yes Sir”.

M: Yes Sir?

R1: “The good side about this is what the government has done to create the Centres for Ebola, it has made us not to fear too much that is a good part of it. Like for us here now when a person is sick before they carry the person to Kailahun or Kenema (= first districts in Sierra Leone with Ebola cases and Ebola treatment centres) they carry the person to (- - name of a larger town in the interview chiefdom with the highest level PHU, a CHC – community health centre - -) to test, from there if they look at him and send him to (- the name of the district headquarter town of the interview district - -). But we thank God for that”.

R2: “The Ambulance is good because now when they have treated them they have now changed the method. Then we are preventing for the sickness not to spread to other people because it can only carry Ebola patients or suspected Ebola cases (*Cock crows*)”.

M: Ok Sir, Yes?

R3: “Eh the good part again I have seen about the Ambulance is that because I am remembering the olden days when a person is sick for you to get a transport to carry him was a problem to take him to the Centre but now as they come, they come and collect the person for you, if you are lucky they can carry the person free and cure him free for you. So on that side they have tried, it is good the government has tried”.

M: Yes Sir?

R4: “In addition to that we tell government plenty thanks because we know that those motor cars are there for us then we are happy because just like what my brothers have said, at first there was delay but for now we see that they are really trying and we have seen that the work they have given them we are asking God that they will always listen so that when they hear a call let them respond quickly and they are really trying now”.

M: Yes Sir?

R5: “In addition to that Sir.

M: Mhmm.

R5: “The other part again that is good about the Ambulances is that now, when a person dies they carry the person back where they took him then they bury him, so in a long run whether it was Ebola that kills the person or not Ebola it is fine when they come to bury him where he was born so in a long run they can say “so and so person was buried here and it was so and so sickness that killed the person”. That is a history to our children then when we do not know where they bury them. So that is one good part about the Ambulance”.

M: Yes Sir

R6: “One thing that the Ambulance are now doing is that,’

M: Mhm.

R6: “They have changed the method, in the past,”

M: Mhm.

R6: “They will not allow us to wash it or pray over it but now they tell us to come and pray over it and for the burial they are now doing it fine is not like when they just go and dump it on that side, we tell God thanks”.

M: Yes Sir?

R7: “Really now the Ambulance are doing fine. Now, now, now - God forbid if a person dies here if they send to them they will not delay again, they will come quickly, when they come, they will bury the person and they will allow us to dress the person. In the past they do not allow, when they put them into that Ambulance they just carry it away and you will know where they carrying it. But now the Ambulances come to us in the town even if a lorry cannot reach the place they will park the motor car and walk their way. In that way they are doing well”.

M: Ok, then when this Ebola happened they gather people to get a Burial Team, who goes to bury is there anything you have observed that is bad about them? Yes Sir?

R8: “One they were not burying fine they will just take the body and go and drop it from there they will just splash mud over it and leave the place”.

M: What do you mean by they will just splash mud and leave? They were not covering the grave fine?

R8: “Yes, when they go they will just take the body and drop it into the grave then from there now they will not have time to cover the grave when they leave then the family can go and cover it fine”

M: So they were not covering the graves?

R8: “They were not covering them fine, that was what happened”.

M: Ok,

R8: “We have seen here when they take a dead body and put it into that Ambulance and go when they go drop the body and removed their shoes and go away even if you call them to come back and bury properly they will not do it. Even here it brings those arguments between us, they will tell us that have to bury then we too will tell them what are they paid for? They will tell you they only have to bring it but we have to bury and they will not do it then they will go”

M: Ok,

R8: “At first they will not even ask the consent of the people they will just go, there was no respect, they did not allow for people to pray. At first I know it was out of fear”.

M: Yes Sir?

R1: “Just like what the Pa is saying because the way they used to bury at first it was not good, especially some of us whose families were affected, they come, they dressed before us and took the body out of the Ambulance and went into the hole; removed everything they were wearing after spraying themselves and allowed the family to go and cover. Well, some of us in the family raised the concern that we don’t have gloves, we don’t have anything to protect ourselves to cover the mud should there be anything scratch on your body while playing with the mud you can get affected from there, not so (=isn’t it) ?”

M: Yes.

R1: “They said they are concerned with covering the body, they left the body there and went; it was the family who managed to cover it. This was bad for us we did not feel fine”.

M: Any other person?

R2: “Mhmm”.

M: Yes?

R2: “They way they were doing at first it was fearful. If somebody dies here even the owner of the house has to leave and find another house. When they come they have a machine which they can send, the machine will go and jug the body and put it into the Ambulance; they close it and go. All the people were now afraid - so even if someone is sick, he will be afraid to report; he will remain in hiding till he or she dies. According to how they come to collect the dead body, it was not respectful even for us now we know that if we get sick now is a problem we will not have the mind to report that we are sick. That is why many people have been affected by this Ebola. They are afraid”.

M: What about the 117 Telephone line, when government said that if you have sick person you should report, you should not do anything to the person you can call 117 so that can send Ambulance to come and take the person?

R3: “Yes Sir”.

M: That 117, do you have anything good or bad about it?

R4: There are some bad that they do”.

M: Mhmm?

R4: “The man who has access to the phone, when a person is sick here if the person who has the phone call to come and take the person when the person dies they will blame the person who called. So, some of them had to report to government that this has to stop. The man who makes the call is the life saver, if you say he should not call then the sick will not leave the village. So people were now afraid, no sooner the man calls they take it that he is an enemy, they are taking it that they are going to find ways to ‘fanke’ (=shoot with a witch gun) that man. That is why people are saying those who are herbalists should be closer to where they are curing this sickness. Some of the sicknesses are not of Ebola it is ‘Fanke’(=shot with a witch gun). Some have the grudge mind that who make the call are responsible for the death of their relative so some will find ways to ‘Fanke’ (=shoot with a witch gun) the man so that he too will die. When they carry the person government will not know it is ‘fanke’ (=shot with a witch gun) they will just class it to be Ebola but those herbalists are closer to people who cure this sick; they will be able to know that this is not Ebola. People have been afraid to call”.

M: Yes Sir?

R5: “In most villages there is no coverage, you can walk around to call but you cannot get coverage. Then the other bad part is if you are able to call because it is National line - it takes two or three days, you will not see anybody”.

R: “Yes Sir”.

M: Yes Sir?

R6: “Especially in the villages the villagers are giving us bad name; some are of the opinion that when you make the call you are going to take money after making the call, so they were against somebody who makes calls to come and take patient or to say somebody dies here and you call 117 to come they will try by all means to know the person who called so that they can quarrel with the person. Even to us it happened except the Paramount Chief had to send somebody to come and talk to the people that the man who make the call owns your lives, he makes the call because he doesn’t want somebody to die here by the sickness, so that the sickness cannot spread. He came three times to come and sensitize people but still people did not believe and that was creating problem. When they said they were coming to check us here, up to date they have not come yet. They only come to ask us how many people do we have in our household whether it is two or three apart from that they have not come to tell us whether this is a dead person or a sick man”.

R7: “Actually the 117 is good on our side it is good except when we hear in the media that there are times when they call them they will go there until late sometimes the body can be there for one or two days but for us here in (- - name of the interview village - -) we have never called them and (then) they fail to come, even when the sick entered here they were not delaying, we don’t have problem. What they are talking about now is the personal phone that we used to get problems, when you call with it and the person happens to die there they will say you are the cause so there lies the problem. But in the office we don’t have any problem”.

M: Ok, yes Sir?

R8: “The fine part on the 117”.

M: Mhmm.

R8: “At first when this sick started if you have a sick man at home except you have to find a bike and wear long sleeved clothes on yourself and carry the person and we have experienced that over two times when they carry the people although those people did not survive again they never came back but since with 117 when you call people will come, it is fine. There was one time when we had a case here when one of our brother died. I was here when one of our sister called to the office but is just that when you call the office they do not respond quickly, but it is fine really because when she called they came is just that they come on time. To call somebody and the person did not come on time I think at any hour the person comes is fine than you did not see the person at all”.

M: Well, do you have anybody who has survived Ebola, who they carried away, treated and got well and came back?

Rs: “Mhmmm”.

M: Ok, so how is he behaving in the community and how are you too behaving towards him?

R1: “Well the boy is (- - name of the survivor - -)”.

M: No do not call names here.

R1: “When he came from Kailahun (= first district in Sierra Leone with Ebola cases) on that day he was welcomed by the whole village because the way he went we were not expecting that he would come again. So when he survived and came back we welcomed him and embraced him; in fact me personally I used to eat with him, I will invite him to my home. So now he is with the community; he goes to the farm with his people”.

M: Ok, minus him now is there anybody who is a survivor and his behaviour is giving problem?

R2: “Well he is the only one we have”.

M: He is the only survivor, but do you know of anybody who is a survivor he went and got well but his behaviour is giving problem?

R: “Yes, actually we are getting it from the media, I have not seen but we hear it on the media when they come out they tell them that they should avoid sexual intercourse for three months. Some do not adhere to it and we hear that anyone they have sex with the person will die, now that they have their armoured car which protects them, so that side is bad about them”.

M: Ok Sir.

R3: “Sir, we went for a Workshop in (- - name of a larger town in the interview chiefdom with the largest size PHU, a CHC = community health centre - -) on Ebola Sensitization and it proved negative, when they came most of their friends were offended so when they were explaining they were crying. We do feel some people some people somewhere they will not welcome them. But here we tell God thanks, the one we have is here with us we play with him, we sleep together”.

M: Yes Sir I saw your hand up Sir?

R4: “Yes, I was trying to support what pastor was trying to say. In the media we hear a lot of rumours. Even there are some people who survive and come back even the Paramount Chief should not reject him because the law says anybody who survived from the sick must be welcomed. Even the boy here with us is common and he is proud, when the crowd come around him we do not shorn him, he is with us “neck-neck” to us, no problem”.

M: Ok, any other person? Well since the outbreak of this sickness, have you heard of any medicine for it?

R5: “Yes Sir”.

M: Yes Sir?

R5: “We heard that they were supplying medicines for Ebola but since they have started supplying none has come here’.

M: They said there is a medicine for it?

R6: “Emm, yes Sir”.

M: Yes Sir?

R6: “We did not hear that there is a medicine for it, we only heard by rumour that the American people say they are going to send a vaccine for that; by this February they were going to test the vaccine”.

M: To test the vaccine en?

R6: “No marklate (= vaccine)”.

M: Ok, is the marklate (= vaccine) that you have heard about?

R6: “Yes, we heard that a medicine has come for that”.

M: Ok, yes Sir?

R7: “We were not able to get the medicine, it is only the Doctors now who knows the medicine that can help us, those who have it and those who cannot get it, that is how government should help us, it will be fine”.

M: Ok, yes Sir?

R8: “When we went for the Workshop on this Community Health Volunteer, one of the Nurses said there is a drug but they are going to test it first to the Nurses, the Doctors are going to be the first to test the drug to see whether it will work, but we do not know if that drug has come. Then we were also hearing about one which they call ‘Zee-map’ (=note: referring to Z-Mapp)

M: Ok which is the American own?

R8: “Yes, according to the information we heard those who first got it complained about joint pains”.

M: Ok, Sir any other person?

R1: “We were really getting the news that there is a medicine that is coming for this sickness but all along we were even hearing about China and another tablet which they call ‘Abigan’ or “B-Something” like that so they were saying they were going to try to make it “dear medicine for Ebola” but we have not heard anything like real medicine for Ebola”.

M: Ok, yes Sir?

R2: “So the only thing Doctors would have to do is to get a perfect Doctor to look after the water for us, if we are able to control the water the chlorine will not affect us. Or let us put a law that, be you a Doctor or not, any water we are using has to be boiled after boiling if there is the virus there it will die. And two, where you have come now, let government understand that the population, that Centre is fit to have hospital there, if they do that here together with the bosses they will be able to control the people and tell them what they should do. But if government did not take its time to build a hospital here to cure people this sickness will not end in Sierra Leone”.

M: Yes Sir?

R3: “There is no medicine for this sick, if there was medicine like the Americans would not have poured money instead they would have just sent the money, they would have come with it”.

M: Any other person? Like how we are now in this community, what are the common things that we think we should know about Ebola and how do we take our time not to get Ebola?

Rs: “Come again?”

M: We are all in the community, what are the things that we should be doing to prevent Ebola?

R4: “Like how they told us that we should not touch dead body, we should not go to burial, no greeting then we should wash hands if we take that law maybe, in fact is not maybe, the sickness will end”.

M: Ok, yes Sir?

R5: “Emm, just like what the Pa said as for us from time when there was one burial in ( - - name of village/town in another chiefdom in the same interview district - -) people did not believe when they said they should attend burial, all the share that God gave us about the sickness is from there. Since we have understood that we have stopped going to burial. I think God has decided to us we only have to keep to the laws. The whole medicine I know about Ebola is just to keep to the laws, don’t touch, don’t wash dead body, don’t go to burial, don’t touch another man’s body if we do that I believe this sick will go, although some people were asking the question that why is it that white people are not dying of this sick is only us? But the white man take the law, when they say this is the law, when a white man comes when you greet him, he will just say hello and pass but for us we will begin to ask you how about your father, how is your child, how about your wife even when you want to leave he will be holding your hand not knowing that you want to leave. But if we are able to keep to the law which they have said, I think Ebola will go far away from us in this country”.

M: Yes Sir?

R6: “The reason why Ebola is going finish in this country and we will not get it again is that when you are married you just have to stay with your wife, the government has to put a law that if you know you are not married who always go to ‘Katakumbay’ (= Prostitute House), if the government is able to stop that, that you have to stick to one partner you should not tamper with any other woman except your own, you should not have sex with a woman who you just met today. If government is able to close that you and I will “bear” then Ebola will end”.

M: Ok, yes Sir?

R7: “We should continue to do the contract tracing. Well me I am suggesting that like the survivors we have a lot about how they have started infecting their women or their men”.

M: Through sex?

R7: “Yes, so it will be fine if government can camp them in one place for the ninety days which they give them before they release them. They are now spreading the sick’.

M: Ok, that is a good idea. Yes Sir?

R8: “Just another contribution, if we avoid all those things that they have said that we should not go to burial, we should not touch each other, we should not wash dead body, you should not bury for yourself and again we should not forget the animals in the bush which they say we should not eat like the bat, baboon, the Chimpanzee; all those wild animals in the bush maybe like for us now we tell God thanks the thing has taken it easy. But some people will say it has taken a long time when the things have been here, let us eat them maybe as you break the law the animal you are going to eat is the one that has the virus and when you eat it finally you have contracted the disease. So the best thing is that you should abide by all the laws they have put, I think Ebola will be finished”.

M: Yes Sir?

R1: “Eh, I just want to understand. If this people who survivals, the woman and the man are survivals (=survivors)”?

M: Yes they are survivals (=survivors).

R1: “If there is any contact between them will they infect each other?”

M: Mhmm, I cannot answer that question because I am not a Doctor, but it is a finer question, me too I will tell my bosses to find out. This is our last question now, is there anything which our people need to know about Ebola?

R2: “One, this old fire wood, let us forget about it”.

M: That which our brother was just talking about?

R2: “Yes, when your friend has been away for a long time and the person comes back then you don’t know where the person has been, so we want those in this village to know that if you have a girl friend in Freetown who comes once in a year. Let us know now that any time your old fire wood comse back take the person to the chief then we try to avoid them”.

M: Ok, Yes Sir?

R3: “Secret Society with circumcision, we should avoid it because it deals with blood”.

M: Fluid?

R3: “So really for this to end, those children who are coming up who do not know anything we should be telling them that there is sickness around because we who are elders know now that the sick is real, we should tell the children more, those who wants to forget that they should take their time”.

M: Ok, yes Sir?

R4: “More so, let them try to find a medicine for this sickness because I can remember in the first meeting they held at the Barry they said the sickmess came from Congo. When it came there after thirty years it reappeared again last year and when it reappeared this time it destroyed mostly parts in West Africa. People forgot that the sick has finished in Congo not knowing that it is hidden, now it has proven and when it has proven it is now spreading. The best way is let Scientists try to be able to get a medicine. Although measles used to disturb our children “nut” it was so it used to kill most of the children but when God blessed it scientists have made a medicine to cure it they started marklating (=vaccinating) the child at the time of pregnancy until five years, we tell God thanks for that. So if the scientists are able to make a medicine and begin to vaccinate people I think if Scientist are able to get a medicine I think it pushes this sickness out but if it remains like this no medicine, then it can appear at any time’.

M: Ok, yes Sir?

R5: “While we are thinking about Ebola we should still not forget to be telling our children. I want government to make a whole subject for the school children especially the children they are giving birth to presently maybe they will not meet the sickness but if they are learning about it in school I hope they will not forget about it , they will be able to know that such problem has existed”.

M: Ok, Yes?

R6: “As a Family we all should be reminding ourselves that there is a sick (=sickness) which is Ebola, let us take this topic as a song in our own life style now to remind ourselves that this sick has been there after it has finished. Like they are saying even the medical people let them be alert now in case of any eventuality about this sickness and we too should be reminding ourselves that this sicnessk is real for to stop certain things so that this sickness can leave us finally”.

M: Yes Sir?

R7: “We should be able to produce or compose local songs in our native language like the Limba which explains how Ebola affects, what the symptoms are then what you should do when you suspect that you are sick. Like what we hear in the ‘Yengetehba’ (= ………….), they have Limba songs that they sing when you listen when they are singing it you will understand fine. In fact we do not sleep at night they sing for the whole of the night”.

M: Yes Sir?

R8: “So Ebola will have to finish but government has to do something. Government is going to feel it and we are also going to feel it, nothing did not go on this year, there is nothing to eat”.

M: Farm work?

R8: “Be it farm work, nothing, did not go on. There is going to be hunger, like from this year onto three years, and if this hunger comes if there is nothing to eat everybody will die, there is no work if the government did not help by sending rice like they used to do for people to farm if government did not do that even the medicine they are coming with we will not be able to take it because when you take it on an empty stomach we will die”.

M: Ok, Sir, yes Sir?

R1: “In addition to what my brother said concerning the songs relating to Ebola I want let the government put good effort of calling on all tribes be you Limba or so to give a song in their own tribe concerning Ebola so that these songs can help us. So any tribe let the government call on all tribes to have songs so that Ebola can be ashamed of us”.

M: Yes Sirs, I thank you Sirs.
