# Supplementary material for: Development of a set of community-informed Ebola messages for Sierra Leone
Source: PLoS Negl Trop Dis. 2017 Aug 7;11(8):e0005742. doi: 10.1371/journal.pntd.0005742 (PMC5560759; doi:10.1371/journal.pntd.0005742)
Supplement: S1 Appendix — (ZIP) [file pntd.0005742.s001.zip › Ebola messages - FGD and interview transcripts/R2HC Ebola Fieldwork 1/R2HC Ebola F1 FGD-MAOLD-Urban1 V2 CORR.doc]

| CODE | **R2HC Ebola F1 FGD-MAOLD-Urban1 V2 CORR (urban focus group discussion)**  **V2 – 11^th^ March 2015 – Correction personal data participants** |
| --- | --- |
| DATE | February 2015 |
| DURATION (minutes) | 65 |
| Collector nr | 6 |
| LANGUAGE INTERVIEW | Krio |
| **TYPE FGD** | Older males |

***PERSONAL DATA PARTICIPANTS***

| Nr | Sex  (*F/ M*) | Age  (*in years*) | Education Level (*e.g. none, Primary, secondary, tertiary*) | Language (*e.g. Mende, Temne, Krio)* | Religion | Job / Employment (*how they earn their living e.g. farmer, teacher, trader*) | Role in community  (*e.g. youth leader*)  ANONYMIZED, ONLY AREA OF ROLE INDICATED |
| --- | --- | --- | --- | --- | --- | --- | --- |
| 1 | M | 29 | Tertiary | Mende | Muslim | Teacher | Youth |
| 2 | M | 26 | Secondary | Mende | Christian | Student | None |
| 3 | M | 28 | Tertiary | Mende | Christian | Student | None |
| 4 | M | 29 | Tertiary | Temne | Muslim | Teacher | Youth |
| 5 | M | 27 | Tertiary | Krio | Christian | Electrician | None |
| 6 | M | 28 | Primary | Loko | Christian | Disc Jockey | None |
| 7 | M | 26 | Secondary | Limba | ? | Student | None |
| 8 | M | 28 | Secondary | Temne | ? | Student | None |

**TRANSCRIPT: (M = Moderator, R= respondent, R1= first person responding to a question, DOES NOT correspond to numbering used in Personal Data!)**

M: In which way has Ebola affected your community?

R1: “Of course we all know that Ebola is an infectious disease, very dreadful, it has affected our community in different ways. In the first place it has created a lot of unemployment, one there are people who were employed by the Public Sector when Ebola breakout they had to lay them off and two we have had a lot of idle youth because of Ebola so that has created a lot of problems, it has created a lot of idleness in our community and the standard of living of Sierra Leoneans has also fallen because of Ebola. Before this time people were employed and were getting money to take care of their family but with the outbreak of Ebola a lot of incomes were cut which has created a lot of problems that people cannot meet their standard of living that is the idea that I have”.

M: Yes Sir?

R2: “The way Ebola has affected our community is great, when there was no Ebola water facility was there though the pumps are not enough, but with the outbreak of Ebola it is more than one hundred people who are staying at home jobless, so is like to get water in the community is difficult. Then two, like every last Saturday in the month we used to go door to door to get some contribution to clean the community but now as we take such venture everybody will tell you that there is no money because of the Ebola, people are not working. Like as you can see the community is not so nice for everybody”.

M: Any other person?

R3: “Ebola has affected the community, like the girl child, we did a research recently, the school going children, especially the girl children they are all pregnant because of this Ebola, and you get me? The children are going backward, the educational level has gone down, and people are suffering. There are a lot of young men, are around the area who are jobless, some people are not able to do anything when night come they [go] about damaging and destroying. So that is my own”.

M: Any other person?

R4: “I am blaming the government.”.

M: Why are you blaming government?

R4: “From the first day when thing came and entered the North the government could have blocked that area, it was a time for our younger brothers to sit to the BECE (Basic Education Certificate Examination, taken in secondary school) but all that has gone backward. Now if you look over (- - names of two other areas in the interview district - -), the amount of children who are getting pregnant is a lot so you see. So I want to the government to take good care for this sickness in our country:”

M: Is there any other person?

R5: “When this Ebola came especially for we the students, when we from school we used to go for lesson but they have just stopped everything they said because of Ebola, that we should not gather because of the Ebola, so we have all gone backward because we are just sitting at home, then again they said we should do not do operation, I even lost one of my friend because of stomach ache but they said they cannot do operation because of the Ebola. Then even if someone is sick he or she will be afraid to go to hospital because when you go they are going to give you injection to kill you, so you see everybody is afraid now to go to hospital especially that which has to do with teenage pregnancy. So you see because they are sitting idle that is why that is happening”.

M: Then the next question is why do you think Ebola is spreading all over Sierra Leone?

R6: “In the first place I w ant to shift 50% of the blame to the government of Sierra Leone, our government was not proactive enough they relied on few people to give them information. Government of any country has the responsibility to protect its citizens, before this time we have heard about a lot of viruses affecting people in other countries, like what happened in Asia and another sickness that broke out in Germany we know one thing sickness, is only one person that would get it if you did not take your time everybody will be infected. When this broke out in one of the villages in Kailahun (=first district in Sierra Leone with Ebola cases) around the Kissy Teng area, the government would have relied on the Ministry, the Ministry I remember there was an interview they did with the Minister and he told the people that all the capacity and security is in place to cut down this sickness. Just to surprise us that all that he told us was a lie. And besides it was very ridiculous to see that sick which was not suppose to move from one place over to another and you take somebody who is sick and they have told you that if somebody is sick, if you are taking that person from one location to another, for example to take the person from Kenema to Kailahun and Kenema is a populated place, it is a business centre then you take a sick man from Kailahun and bring him or her to Kenema when you bring the person to Kenema there is the possibility that the people in Kenema will become infected because it is a highly populated area, I saw that it was necessary for them to barricade the location where the sickness started in Kailahun. When they were saying that people were denying, of course in any society you have people who would deny but the government has the responsibility to stand by itself and say no we have to protect our people. I saw it that it was necessary for the government to have blocked Kailahun as the thing started so that they could have been able to cut down on this. One of the major reasons that lead to the spread of this sickness is the transportation of people sick of (with) Ebola from one location to another”.

M: Any other person?

R1: “For me why this thing spread is that some people did not believe that the sickness is real, like the time when it entered Kailahun (= first district in Sierra Leone with Ebola cases) some people were taking it that is a lie [it] is just Malaria, so when a person feels sick they take the person, they touch the person, so when they take the sick person now the sickness transfers to them and they too can take transport to come to town so that is how thing transfers, people do not take this thing seriously so the thing decide to spread to the whole country. So right now it is causing a whole lot of damage in this country”.

M: Any other person?

R2: “When this sickness broke out about one month, people were taking it to be APC and SLPP (the government political party and the main opposition party) and we are not here for that, the politics, it is this sickness now that is our country because 90% never knew Sierra Leone for sickness, so at the moment when the sickness broke out it made the whole world to know Sierra Leone we know that the government and the people should come together, when they say ‘Don’t Touch’, and they come with all this kind of stuff such as wash hands you know”.

M: Any other person?

R3: “This sickness spread into this country, the thing is that, they had told the President that the sickness is in Guinea, the person left here and went to Guinea to attend a burial ceremony from there when the person came back he got infected and died in the village but people were still going up and down, so when the sickness spread in the town that was the time they told the Pa that the sickness has reached so, so, so area. By then there was no awareness, people were not abiding to the law so later we saw student having the sickness and they told him to go to the hospital the person will run away. They send it to Facebook and WhatsApp and we see it. Maybe when the person runs away and come to the bush and stays there, then enters into a transport and come to town that is how Ebola spread into the town”.

M: Yes?

R4: “One thing that (…?…) know which makes the Ebola to spread in the city is 100% blame is to the government”.

M: Why?

R4: “Because when the thing started the way and manner in which they talk about the sickness, the symptoms of the sickness and when the sickness started in Kenema it never had those symptoms, the government would have quarantined the whole of Kailahun District (=first district in Sierra Leone with Ebola cases) and lock the border with neighbouring countries to try and protect the citizens but I am looking at it that the first money that was involved in this Ebola business made the people of the Republic f Sierra Leone never believe that the sickness is real. It was only after the death of Dr. Umar Khan (*The first doctor to die of Ebola in Sierra Leone, head of the Lassa Fever (another Viral Haemorraghic Fever ) ward*) that was the time people realized that the sickness is real when we lost the only man (*note: considered as only specialist on VHF in country*), so like I am blaming government 100%”.

M: Yes Sir?

R5: “Ok, the spreading of Ebola, denial is what that caused the spread of this Ebola because one thing when this Ebola broke out from Kailahun (=first district in Sierra Leone with Ebola cases), come to Kenema by then the government was aware and they started telling people that they should not touch, you not go near a sick person, you should not wash dead body, you should not do so, you should not do so. We, the people of this country when the government has said so we need to do it. Like when they said you should not wash dead body if we had done that I think the thing would not have spread. From when the thing happened they took as a political issue they said it is so, .so, so and so. That made Ebola to spread all over the fourteen Districts, that made the thing to spread people were not abiding to the law even in the city, everybody was a care free saying that it was a lie. So that is my small idea that I have”.

M: Yes, I saw your hand up?

R6: “I like what everybody have said about this Ebola, like the government, I blame the government small”.

M: Why?

R6: “Why I am blaming the government small like my colleagues have just said at the time when this sickness started in Kailahun (=first district in Sierra Leone with Ebola cases) I was expecting the government to quarantine the whole of Kailahun for some certain period of time to contain the disease for them to be able to get rid of this thing. Secondly this Ebola exposed the Health Sector in the country, the Health Sector was poor, we do not have hospitals in this country. When Ebola came we saw that they started creating Holding Centres because they were not able to contain the sickness that shows that the Health Centre was not active. Then the movement from one place to another, people move from one to go to another place that is one reason why the sick spread. And fourthly, Kailahun is a business place where they used to have “luma” (=special market day), I know Kailahun like “luma”, people come from Liberia and the neighbouring countries and come to Kailahun to come and do business, all that area could have been locked down from the border to contain the sickness from spreading. This government has the right to protect the citizens of the country despite the economy or whatever, or because of food or whatever, it is better you contain the sickness than you open door because of food of whatever because is like Sierra Leone is not economically sufficient, maybe that is why government did not lock the borders for fear that people might starve. But is like it is better that you contain the sick than you incur the amount of death. Then another thing is you can never allow a virus to enter the capital city. If you have heard it in the provinces at first it should be there so that you can contain it because when you watch and those things are coming into the city which is very populated area then you cannot contain it, that is what that caused this Ebola business in this town and the country up till now”.

M: Any other person?

*(Silence)*

M: Ok, in this community, do we have another name which we can liken Ebola to?

R1: “We call it “Taebukor””.

M: “Taebukor”, what does that mean?

R1: “That you should not wash”.

M: Are you trying to say that it is because of dirt that a person can get Ebola?

R1: “Absolutely, not out of dirt but “the sick of itself is dirty””.

M: Ok.

R1: “Because when a person is sick you just have to wash him or her for his body to get light then when a person dies you just have wash him or her but because fo Ebola you don’t have to wash the person, when a person is sick you don’t have to clean that person, so in this community it is ‘Taebukor’, that you do not wash”.

M: Is there any other person with any other name?

(…………?.....................)

M: Are there people in this community who do believe that Ebola is real?

Rs: “No”.

M: Ok, it is not there at all?

Rs: “No”.

M: Then if they have that view, why do you think that it is not real?

R2: “Through the sensitization I will now tell you that majority of the people in this community now believe that Ebola is real because like we have had few cases people have just been released from quarantined home. So you see, some people believe that Ebola is real but some others who do not believe, everybody has right to his or her opinion because like you cannot force somebody to believe something. But I can assure the government that majority of the people in the community do believe that Ebola is real and that it is something which is a deadly virus and killing people”.

M: Any other person?

R3: “Ah, I can say over 95% of people in the community now believe that Ebola is real. The few who are still in doubt who are in between is for a simple reason”.

M: What simple reason is that?

R3: “I discussed with some of my friends who told me that they were still in doubt. They said before this time people were dying, but when anybody dies now they say it is Ebola and two people are dying of a very weak background but they give them Ebola burial but when somebody dies in a well-to-do home the government will produce the body to that family for them to give it a befitting burial. It happened when this man died...’.

M: (- - name of the man from the “well-to-do-home” that died - -)?

R3: “(- - name of the man from the “well-to-do-home” that died - -), which I am questioning. In the first place his son died who was a / al ?? priest and they buried him the Ebola way and some days after the father had to died, some Minister make convoy of motor cars to go and bury somebody in Kono instant, then it ?dhows? some of in the community to ask why should this happen when government had put out a release that when somebody dies once the person dies at home they bury the person the Ebola burial [way] so that created a lot of the [confusion]? on the minds of people in this community but like when we have some educational background we often tell them that in a society no matter what happens there are people who have opportunity. So that is it”.

M: Has anybody anything to say?

R4: “Yes Sir, well me as for me I am involved in this Ebola issue because for me I believe that Ebola is real but some people do not have the belief that Ebola is real because why? The way nurses and Doctors do treat people when they are sick is not what that happens to people. I have the belief that Ebola is real. They when the sick a person you see the person vomiting and the person catches fever and the person vomit blood you understand but I have never witnessed that with my eyes not even to the Doctors. That is what I have to say”.

M: Yes?

R5: “To add to what my man has just said, they say ‘seeing is believing’, the government has never shown us an infected Ebola person that is one. Then the signs and symptoms of Ebola coincides with and is the same with a person somebody with Malaria which is also connected with the signs and symptoms of Ebola, like vomiting, as for dysentery, it is the order of the day. Then in addition they are taking the figures of normal deaths and adding it to Ebola death. Because if like Ebola Ambulance comes ,to bury somebody I know that they will take it under tally of Ebola (= will count it as Ebola) and one thing I know is that it is fine for them to differentiate Ebola death from normal ailment let them put it that the person died of this ailment and put it under Ebola death. Let there be normal death because before this time people were dying, every day people are dying let them differentiate it for us to know who died of Ebola and let them know who died of normal sickness. That is what I want them to do”.

M: Ok, any other person? Ok, then when Ebola came there were a lot and lots of messages, they will tell you not to touch, not wash dead body, when the person is sick you carry him or her to the hospital, which one of those messages like you in the community do understand very well?

R6: “Well the simplest message which this community can understand is ‘Don’t Touch’, even they have said we should not touch we have those who are stubborn. Then two, those symbols that they put around, those billboards or those signboards which they build around showing the bat, monkey and the man, well like 80% of the community was able to understand that message”.

M: Any other person?

R1: “All what they are saying, as a community we had agree to the ‘No Touch’ but like we are here now people can, ah, forget you are my brother. But the only law which took as serious is when they said when somebody dies you should not touch the person. If that happens you call 117 because if you go bury on your own government will hold you responsible”.

M: Any other person?

R2: “Yes Sir, I want to make a contribution into this talk because like the government said to people that there should be no touch, yes we too know that there should be no touch but still people are touching. Do you know why? Even when you are in the various transport you must touch a person or even when you pass around the ( - - names of three market places in the interview district - -) you must touch your companion or even when you take ‘Okada’ ( = Motor bike) you must touch your companion so they do not talk about all those areas. And I have never got the experience that somebody who is on ‘Okada’ has fever or in a transport you touch your companion and then you get Ebola, I have come seen that. This could have been stopped but still the thing is persisting but I believe that the law did not hold; people are still touching. That is the area we want Papa government to look into”.

M: Which Ebola message do you think you can give somebody to encourage him to go to hospital for treatment?

Rs: “Go over again?”

M: Among all the Ebola messages, say ‘Don’t Touch’, when a person is sick you should not touch the person, when a person dies you should not wash it. But which one do you really think will encourage people that when they are sick let them go to hospital for treatment?

R3: “The awareness is there now, that is one. Then what would make people go hospital the government has to show something like even those sign boards if they show an Ebola person who has actually been infected by Ebola like with blood oozing from the ears, nostrils I believe that people will believe the more, there is awareness now. People no longer treat people at home because like everybody is trying to protect his or her family so I believe that everybody goes to hospital now ; it is just because of the way the Nurses behave will make a person to be afraid to go to hospital for them not to class him or her as Ebola because they will be afraid to touch you to attend to you quickly so some people will prefer to treat him or herself at home because he or she think that is the way to prevent him or herself”.

M: Yes Pa?

R4: “Well the sensitization method was very nice, the people has to have the confidence government to improve the Health Sector. The first information which government out that when you go to hospital and you have Ebola they can cure you, if that was the information government brought out quickly, I believe that Ebola would have ended by now. Before this time most people were afraid to go because they think when you go they are going to inject you and kill you then you go and die at the hospital. We have got people now who are expatriates who have told us that if you go to the hospital and you have Ebola, they have some basic treatment which they can give you, Ebola does not have any specific medicine but they have basic treatment which they can give you that could save your life and this sensitization has gone into a lot of the communities, we have seen people have been infected they went and came so people have belief that if you go early to hospital there is possibility that you can save, so many people now go to the hospital voluntarily when they become suspicious of Ebola, you see some people complaining about fever, some say they are not feeling well so voluntarily they go to hospital. It is the sensitization network, the way how they coined the sensitization is what that is creating doubt on people’s minds. But this later part now they created that people have become confident in the institution that anytime they feel sick they go to hospital”.

M: Well with all the Ebola messages that have been around, which channel do you think would be the best way for people to be able to understand these messages?

R5: “The involvement of the local community is one of the best ways”.

M: How do you do that?

R5: “For example somebody who is living with his or her companion in the same community and he or she comes and tell him or her a message he will do it more than the person who is coming and does not know. Message can come from different person but the person who carries the message can be a very good source, if you trust that person any message he or she is giving you, you can rely on it and believe in it. So I believe that the involvement of the local community could be one of the best way to sensitize people of any dreadful disease not only Ebola’.

M: Any other person?

R6: “Like what my colleague said, the involvement of the local community plays a great role; like to go from house to house. I see that it can be more beneficial to the government to go house to house to tell people more about Ebola because like it is not everybody has Radio or Television to listen to the messages that come from WHO. But if they coordinate with the community people and go house to house that can be better”.

M: Ok, then in the case when a person has Ebola do you think they would first go to the ‘meresin ma’ or they would go to the hospital, do they still hold that belief?

R1: “Some people do not hold that belief , some people do take their sick people to the hospital quickly for the person to go and get medical for them to get well some people do not do that they keep the person at home and that what that is bad now when you take an Ebola patient to treat him or her at home it can be better for you to take him or her to the hospital so that they can treat him or her quickly maybe if God has his or her life he or she can save”.

M: Yes Sir?

R2: “Is all about the belief. Some people by the signs and symptoms they way they think it is a “witch gun” or whatever, so some people what they had believe is what they follow so they prefer to go to a ‘meresin man’ (= Traditional Healer) when they are sick. Is all about the belief”.

M: Yes Sir?

R3: “Some people believe to go to the hospital because now many ‘meresin men’ (=traditional healers) have died. One of them a patient went to him not knowing that it was Ebola so he treated the man, but the man died and later he too died, so when the news was on radio and on the community radio they too became aware that Ebola is real and that when a person is sick he or she must go to the hospital”.

M: Yes Sir?

R4: “I want to contribute in this talk because we so many ‘meresin men’ (=traditional healer) who are in the community. When they say traditional healer they are not there to kill people. In this Ebola fight so many, many people have died sometimes through this “witch gun” business. I have somebody towards (- - name of another location in the same interview district - -) but this person when he comes from his home he goes into his house, when he goes into the house he started feeling warmth in his body but by the…. they called one herbalist but the herbalist was afraid to go and see this person and try to see what was really wrong with the man and this herbalist had the way to go and cure the person because they had put a law that no herbalist should not hold a sick person so you cannot afford to run seven years or eight years jail for just own person. They guy said he felt like there was metal moving in his body so by then they call 117 and they came and took the guy, they sprayed him they mixed the chemical so high and when they were going with him his last words were that he knows they were taking him and he know he was not going to come back and indeed, he did not come back, and later the ‘meresin man’ (=traditional healer) confessed that it was not Ebola it was a “witch gun” so the guy died”,

M: When this Ebola came government set up a lot of services like the Ambulance Service, the Holding and Treatment Centres and the 117 phone line. From all the things government has set up have you heard anything good or bad about them? Let us start with the Ambulance service, is there anybody who have heard good or bad things about the Ambulance Service?

R5: “Lie I Have heard one, when they come to house where there is Ebola patient they do take bribe, it has happened in some part. That is one”.

M: What about the good things?

R5: “Good thing to them they think if come to them early they might get infected so when you call then it takes at times three or four hours before they come to the place they do not come on time, that is second thing and all is about the bad but it is good thing they can come and take the body because they are risking their lives and that is a good thing they are doing to his life on the line, it is a good something”.

M: “Yes Sir?

6: “The bad thing I have heard about the Ambulance is that like from the provinces if there is nobody to come with they use it as transport. They can open the Ambulance and out in there and travel with them, I believe that is not good, the vehicle might have virus and if put somebody in there who does not have the virus/sickness he or she is just going to be infected”.

M: Yes Sir?

R1: “Yeah, in the first place, I have a question with the availability of the Ambulance. The government told us that since the Ambulance were not sufficient initially, the spokes went to the and told us that they were trying to import 30 Ambulances initially, later on they said is twenty but the actual number that came was 16 and the quality, a lot of Civil Society Organizations questioned the quality of those Ambulances understand?”

M: Mhmm.

R1: “Up till now as I am talking the four Ambulances which left behind when I listened over FM and SLBC when they said they should bring, up till now as I am talking the four did not come only 16 came. So we raised some questions as citizens we are concerned, we raised a lot of question. Then secondly, is the response time of the Ambulances drivers. It was too poor initially, when they call 117 for the Ambulances reach where the sick person is it would take 2,3,4,5,6 hours and that used to create a lot of problem maybe before the Ambulance come the infection would have spread, people would have touched their friend, or their colleague or their good person. it is the time of the response that has created a lot of problem with this Ambulance business, but it is a bit better”.

M: Ok, yes?

R3: “The bad side of the Ambulance, like I was in (- - name of another district - -) not only ( - - same district -- ) but I came to (- - name of another district headquarter town - -) where in these drivers because they give them full tank fuel when they run for the day, when coming back to the holding Centre they go and draw 1 ‘bata’, 2 ‘bata’, I saw the two cases the one in (- - other district - -) and the one in ( - - another district headquarter town - - ) when they caught the driver on the spot drawing fuel from the Ambulance. That is a very bad thing because they give you this motor car to go and take patient with full tank and then go and draw fuel I don’t believe that you are willing to do the job is just because you know that there is money that is why you took the job. That is the bad side”.

M: What about the good side?

R3: “Well the good side is that when somebody risks his life on it the line it is not easy for anybody because even your mother at certain stage she can run away. So they call those people and they come and take them and go I will congratulate them for that”.

M: Any other person? What about the Treatment and Holding Centres, is there anything good or bad about them?

R4: Eh, the Treatment Centres when people go to the hospital the nurses and the Doctors will be afraid of you and that is not good when somebody goes there who is sick they should take care of him or her even when night come they leave the people there and run away so when their friends and colleagues go and touch him or her all those things can make the sick spread more and more”.

M: Yes any other person about the Treatment Centre, like the one at ( - - name of treatment centre location in the interview district - - ) I visited somebody there and the person next door to him said he wanted to eat meat and the Doctors said it is not what he wanted to eat that they can give to him and he told them that the food they gave him the previous day made him felt bad, vomited so he said if that was the particular food they were going to give him then he was not ready to eat it and the Doctor told him that what they have marked for him is what they would give him. And I believe that if somebody is sick is what he or she feels, what his or her system, you cannot give me something and when I eat it vomit and you want to give it to me again that is not possible that is the bad side”.

M: Ok, yes Sir?

R5: “Well the bad side about the Centres is that like the one in (- - name of another treatment centre location in the interview district --) when they built it I know a very good Doctor who went there and got affected by the sick, within the few days when he knew that he has been infected he told his driver to drive to the place and the people told him that if he was an American citizen they could have attended to him but they said as long as he from here they were not going to treat him. They made it clear that they could not be able to do anything for us here and I think that is the bad side for the Centres”.

M: Ok, what about the Burial Team is thing anything good or bad that you know?

R6: “Well the Burial Team I can say it is worst among all three sectors”.

M: Why did you say so?

R6: “It doesn’t mean that when a person dies of Ebola when you go to bury the person you can just fling the person like that. Each and every mortal man the last respect which you give you fellow man is when he or she dies, no matter what kills him or her but when laying him or her to rest do it in such a way that the person can know that he has left this world for eternity but not when they say because this person died of Ebola that can hold the hand and the other hold the foot and just fling it like that. Nobody can be glad for that. Because whether there is Ebola or not some people they put them into casket you would not have the mind to fling it, is not because the poor ones because they do not have money so you have to fling the body, that is a bad side about the Burial Team”.

M” Ok, Yes?

R1: “The Burial Team, when they go to bury they take bribe when they bribe them the people will take the body to Church and they go to bury again and that is not nice so that is what I have to say”.

M: Ok, yes Sir?

R2: “Yes eh, the Burial Team, initially when it all started they had a lot of problem, when somebody loses his or her loved one or somebody close to him or her he or she would always want to give the person his or her last respect. When the Burial Team go they do not take care to even take the body in that respectful manner they were not doing it. They were very arrogant, they didn’t even know how to talk to people and you know takes a lot of confrontation because somdy’s person would die and they want to go and bury the person then you do not talk to the person fine, you will create a lot of confrontation. At least they have created a situation wherein the Burial Team can satisfy and the person too can satisfy. Now they can even allow you to pray over it and say what you want to say to the body before they take it”.

M: Ok,

R3: “I want to buttress what my brother has said, you get me?”

M: Mhmm.

R3: “When the thing started the Burial Team was poor, in the provinces when a person dies they call the Burial Team they will not go there quickly, you understand me?”

M: Mhmm.

R3: “Like some area bodies used to decompose. When they call them they will this, they do not have this and that, and government has not given them sufficient vehicles. They had a lot of problems in the provinces because I used to go there. It reached a point the thing was poor. But for now we thank God from the time government put hands and put modality in place the thing is now going well. Like one thing I am trying to say the Burial Team in the past used to take bribe, like what my brother was saying mortal man when you die they have to honour you. But my brother if go to (- - name of a burial location in the interview district - - ) and see the kind of way they fling the body. At one time I went there again when I lost my friend, the graves are not properly dug, when they take the body again they will not put it gently, they would just fling it, this one will hold this way and one the other way and they will just fling it like that. Form the time when people started going to the media and started complaining about that I think now it is better”.

M: Ok, yes Sir?

R4: “One thing about the Burial Team, I listened one interview when they went to take a boy who had lost 11 people in his family to Ebola; he was the only survivor but like there were other people infected so all of them were loaded into the Ambulance as they were going on the way other died, and the other one fell and the other one which was there said driver people have died here, he replied telling the boy not to cause noise for him, so the boy said all those who were at the back of him were dead bodies and that before they could arrive they were all going to die. So you imagine if you are sitting near a dead body you can feel bad. The other side on this sickness is that for them to tell you that there is no medicine for it and the worst thing when the person is dead the sickness becomes violent because you who is going to touch it you get the more. Well if you see someone volunteer to bury that person, I think the person did well, he tried”

M: We have Ebola survivors in the community not so?

Rs: “Yes Sir

M: Does the community react to them? Does the community treat them well shuns them?

R5: “At first in some areas they used to stigmatize them. But like when the government put mechanism in place to stop that I don’t think that is happening anymore because like for us here we have a case where two people were affected and they quarantined down (- - another location in the same interview district - -) they went and came but I see that the community did not shun them because they are living normally as before, I noticed that government has succeeded in sensitization on stigmatisation”.

M: Ok, yes?

R6: “I believe that those who survive at the Ebola Centre and came they are treating them well because I even have of my brother who got infected and went to the Centre he was there for about one month and he came back, every week I go to him. I asked him about how is the place; he told me the people are strong doctors have given him the Certificate that he is well and I believe that the people held him fine at the Centre: “

M: Any other person? Ok, yes?

R1: “I want to add another thing which I want government attention to, these male and female survivors who are coming back they gave them a stipulated date which is ninety days for them not to have sexual intercourse but some people are not heeding to that advice and they are having sexual intercourse because like I learnt about a case when a male survivor forced his wife to get sex, the woman died of Ebola and later I did not know what they did to the person. I they cannot stop that I am suggesting to government that let them create a confinement where they can put those people that ninety days and after which they can allow them to come”.

M: So they should not just live them like that?

R1: “They need to have a new face on the Ebola transmission”.

M: So, that is the suggestion you are making that let them be confined until after ninety days?

R1: “Yes in a solitary confinement until after the ninety”.

M: Have we heard any new treatment for Ebola? Like say, Marklate (=vaccine) or medicine?

R2: “Yes of cause, they had said they were working on “Zee Map”, they are working on “Chicken 05”, eh we have heard a lot of things but I have not seen it yet”.

M: Ok, yes?

R3: “Yes eh I learnt about experimentally tested vaccine which they say is on the way and that our shipment has come but it in Liberia. I want to ask this question, what is happening to our own country, why didn’t divide it send some to Liberia and send some here. I listened to VOA (Voice of America – radio) and they talked about it that they have tested it on twenty students so like it proved active so I want them to send it here it will help greatly, because like government is now saying schools should reopen if that happens it will minimize the cases of Ebola in this country.

M: In this community, what is the point of discussion about this Ebola? What do people have doubt over which you think people need to know?

R4: “This Ebola the main thing which people have doubt over is when it first entered Guinea they showed us a picture of like something will appear on your hand, they said when you have Ebola that is the sign and then you will have blood oozing from every part of your body where there is a hole. But the Ebola in Sierra Leone we have never seen such a sign on anybody so that is why most people they have doubt”.

M: Any other person?

R5: “Yes signs and symptoms of Ebola, that they were initially talking about is not what we are seen this days, that has created the doubt because when Ebola came they were showing us some photos which is quite different from what we are seen now and every day they are adding signs and most of these signs are the same signs that used to hold people and they die. Today you can hear that when you have scabies they say is Ebola, when your stomach is running is Ebola, when you have too much head ache is Ebola, you know every day there is no specific sing so that has created doubt and is confusing people’.

M: Is there anything specifically that we need to know as a community about this Ebola that can help to prevent it?

R6: “Yes we have”.

M: like which one?

R6: “They first thing we should try to know is how actually is Ebola transmitted and easily, that is the first thing they should do for us to know as community let everybody know in the community. Then what are the effects when somebody has died what the effect of that body to the survivors especially when they suspect the body that it is Ebola?”

M: Ok, yes?

R1: “One thing we want to know what is the exact days that Ebola can take into your system when you cannot survive again”.

M: Any other person? Well gentle men thank you very much ya?

Rs: You are welcome”.
