# Supplementary material for: Development of a set of community-informed Ebola messages for Sierra Leone
Source: PLoS Negl Trop Dis. 2017 Aug 7;11(8):e0005742. doi: 10.1371/journal.pntd.0005742 (PMC5560759; doi:10.1371/journal.pntd.0005742)
Supplement: S1 Appendix — (ZIP) [file pntd.0005742.s001.zip › Ebola messages - FGD and interview transcripts/R2HC Ebola Fieldwork 1/R2HC Ebola F1 FGD-MAYOU-Rural1 V4 CORR.doc]

| CODE | **R2HC Ebola F1 FGD-MAYOU-Rural1 V4 CORR (rural focus group discussion)**  **V2 18feb2015 – corrections to personal data participants**  **V3 2^nd^ March 2015 – Probing added**  **V4 – 11^th^ March 2015 – corrections to personal data participants** |
| --- | --- |
| DATE | January 2015 |
| DURATION (minutes) | 81 |
| Collector nr | 5 |
| TYPE FGD | Young males |

***PERSONAL DATA PARTICIPANTS***

| Nr | Sex  (F/ M) | Age  (in years) | Education Level (e.g. none, Primary, secondary, tertiary) | Language (e.g. Mende, Temne, Krio) | Religion | Job / Employment (how they earn their living e.g. farmer, teacher, trader) | Role in community  (e.g. youth leader)  ANONYMIZED, ONLY AREA OF ROLE INDICATED |
| --- | --- | --- | --- | --- | --- | --- | --- |
| 1 | M | 20 | Secondary | Temne | Christian | Student | none |
| 2 | M | 22 | Secondary | Temne | Muslim | Student | none |
| 3 | M | 24 | secondary | Temne | Christian | student | none |
| 4 | M | 20 | secondary | Temne | Muslim | farmer | none |
| 5 | M | 20 | Secondary | Temne | Muslim | student | none |
| 6 | M | 23 | Secondary | Temne | Muslim | student | none |
| 7 | M | 23 | Secondary | Temne | Muslim | student | none |
| 8 | M | 20 | Secondary | Temne | Christian | student | none |

**TRANSCRIPT: (M = Moderator, R= respondent, R1= first person responding to a question, DOES NOT correspond to numbering used in Personal Data!)**

M: How has Ebola affected your community?

R1: “Ebola has affected us greatly, Ebola is not only party of our vicinity, is also all over Sierra Leone as a whole. The Ebola attacked us particularly in (- - Name of interview village - -) here, there are about three quarantined houses. In the first quarantined house Ebola has killed more than seventeen (17) people in there who are our brothers and we have personal relationships with them and we felt it to the extent that Ebola has destroyed them and we are not seeing any improvement . Till this day we are not seeing any improvement in the lives of their people. So that is the way Ebola has affected our community”.

M: Yes any other person?

R2: “What I can add up to that, like my brother said, we just heard of Ebola from Kailahun (first district with Ebola cases in Sierra Leone) when they said Ebola, Ebola. It has affected us greatly. As I am talking to you so, all my family is dead. So, almost I felt it. Like I am talking to you now, if I tell you I am happy, I am lying to you.

M: I UNDERSTAND!

R2: “Even the house we are building, the materials, the cement, the zinc that we bought they stole everything. I have been laying down for three months and some weeks. When medical people come for our people they take them to government hospital they lock them up, no medicine, no food and no communication with us not until the Doctor comes. My brother was there until they took them to Kailahun (first district with Ebola cases in Sierra Leone) where they died. Of all my family members, there is no one whose grave I was. This is my own family problem. So that why for me, Ebola damaged me”.

M: Ok,

R3: “Like what my two brothers have just said, Ebola affected the whole (- -Name of interview region- -), it affected even us who did lose our loved ones but we must have sympathy and are to empathise with those who lost their loved ones. So, it affected all of us in (- - Name of interview village - -). Because of you see you losing your friend or your friend losing his or her loved ones, means a lot to all of us. So the Ebola really caused a pain to all of us".

M: Ok, Yes.

R3: “We have been affected. In the whole of (- - Name of interview district - -), we are the first people to be victim of Ebola. When you go out at night you see it, even when you go to (- - Name of district headquarter town of the interview district - -) we have that stigma and we are not feeling happy at all because in the town anywhere we right now they say “aaa, look at them, “(- - Name of interview village - -) Ebola”, that is how they are calling us now, “(- - Name of interview village - -) Ebola”, so we feel embarrassed we do not feel happy at all”.

M:Yes?

R4: “Well to throw light towards that, I feel offended towards the situation because when you look at the way we see they attacked the issue we don’t feel glad about that. Eh, the way and manners in which they treated our people, I believe that discouraged the people not to have the zeal to come out and go for medication again, that caused people to die, and, when you look at the way those who come to collect dead bodies for burial; be it Ebola or not, they have to come bury you. I saw most of the people were suffocated by this chlorine and that led to the destruction of most of the lives in this country especially here in (- - Name of interview village - -) because if I can give example, at one time they came to spray, rather unfortunately they met the people preparing food and according the confirmation that heard, they spray all the food so they said the food stuffs that they brought for them look like a poisoned one and when the people ate the food they were rolling on the ground like... they were saying that this has never happened to them. See how empathetic that is. They were rolling on the ground. I listened to one time to the Resident Minister stated publically on TV that he held a meeting in (- - name of town, location unknown……….- -), even the first case that we had, we made a .......dis...respectful manners and went to the extent that the boy whom they said had Ebola was taken to the Centre, according to what we understand they went with him to the Centre, then the boy’s Father went there with cutlasses and hoes and we went and took the body in his truck and brought it here, so all that was an insult to come and give us action to our people that has not been part of us. All that is just to scandalize the situation and that is bad record towards the situation. We do not feel fine towards that. So, and we are not even glad for that so.We are appealing to government and all stakeholders who are in charge of this Ebola fight for them raise concern towards the situation in which we find ourselves in this country especially in this of our community”.

R5: “The Ebola actually affected (- - Name of interview village - -) town, Ebola affected many households. So the way they come to bury the corpses their families are not glad about it. They take the body and throw it away so there was a riot”.

M: Ok, I want to ask you something how did Ebola affect development in (- - Name of interview village - -)?

R6: “There is no business going on”.

R7: “They gave us curfew order, soldiers were all over if you come out they will arrest you and take you to the police. So”

M: What do you think is responsible for the spread of Ebola in Sierra Leone?

R8: “According to my own understanding, I thought it fit that this Ebola, the way and manner which caused the expansion (spread) of this disease is the way they handled the disease. And you cannot see a person who is not normal and you take him and bring him later without any output toward the situation, then you will not have that affluence to send your own loved ones to the hospital. So, all that gave more emphasis towards the spread of this disease. And since they come to collect people here, the way we see they take care of the people, the corpses to be specified, the way they handle the corpses gave us tendency to cause the riot. Some police and soldier men and the Burial Team were stoned. When later they brought the police and soldiers some of us were not able to sleep here. All that is responsible for the spread of this disease because if they had handled the situation like how they are doing now, if that was the way they attacked the situation that would have brought a respectful and humbleness people who wanted to give themselves to fight the Ebola but since they know that they are not feeling bright they can go and report themselves. But that way which they first attacked it, that caused the riot. And still to date people do not have the confidence that when they go with them they will come back”.

M: Yes, my own brother, I see you want to say something?

R1: “Well the way how Ebola affected us here in (- - Name of interview village - -), you can see a police man comes and lock all our brothers in a cell. They cease all our freedom, even to go school we cannot. We are not hearing anything again, so we don’t even know when are schools going to reopening, we are just seen this and that. As I am sitting here we are supposed to sit BECE (=exam), but we don’t even know whether time table or school is going to reopen”.

M: But what do you think is responsible for the spread of Ebola in Sierra Leone?

R2: “Well, the way they were attacking people, because if they were attacking as if when this sickness, they should have come with something that people can hear. If you get to know that when they took your brother, they too can give themselves up to government and go to the hospital but when they carry them we will not hear anything about them and they will not come again”.

M: Yes, my brother?

R2: “In addendum to what my brother has just said, I see some areas like in the villages, where there is no vehicle, I saw one Soldier when they went to take one man whom they say died of Ebola, I cannot say it was not Ebola. But the way they threatened the people that everybody is going to die here. How can somebody be able to give him or herself to government when he or she is sick for him or her to be taken away, it will not be easy”.

M: So, you are saying because of that threatening remarks made Ebola spread in Sierra Leone, en (=isn’t it)?

R2: “Yes”.

M: Ok, my brother?

R3: “Yes my brother thank you very much for asking this salient question. Eh, the spread of Ebola, one thing that we are made to understand, we the people are reluctant, very reluctant and it has a positive and negative effect. When the medical people said “Don’t Touch”, you are supposed not to touch. If I can look to the question, the direct purpose of this question according to the way you are asking on the spread, when they say don’t touch, don’t touch. And secondly, avoid washing dead bodies, you should avoid. Then they said stop doing all this things but people are still reluctant. This is another cause for the spread of Ebola. Then if I can also talk on the medical side, the way they are treating our people at first when this sickness entered into Sierra Leone. When they come particularly at (- - Name of interview village - -) for instance, when they are coming, they come to a house which they understand that there is an Ebola patient there, they will take that person and carry him or her away and they will never come with the result as earlier and some of our people we do not even know about them who are our brothers. Then if you understand that they took my brother away and they killed him, because some people believe that they killing people. Then if you are here and you get sick, you will be afraid to go because you know that your first brother whom they went with is not alike any more, they have killed him. So you can see that if there is any other person who has Ebola in that house then it is going to spread because they are afraid, so these are the instances”.

M: Ok, Yes?

R4: “As you have thrown the question to us, the thing which caused the spread of Ebola is ‘unbelieve’. People never believed that Ebola exists. That\t is the main factor which made Ebola to spread. Because people never had the belief that Ebola is real. So even when the people come to sensitize then they just think is a waste of time because they do not have the belief that Ebola is real. When Ebola first attacked people took that it is Bondo this, Bondo that, because they do not have the belief real and that cause the spread of the disease”. And that Ebola is what that is responsible for the spread of Ebola. That is one main factor which caused the spread of the disease".

M: Ok.

R5: “Yes, let me add to what other has said. Ebola, Sierra Leone, let me say the whole of Sierra Leone is negligence. Some men, except they see it with their own eye before they believe. Like when the Ebola broke out, if we had believed we would not have reached this point. But some men likes deny, they said oh, they don’t believe. Some say it is witch plane crash that is why people are dying. So all these things makes them go along with the perception that Ebola is not real. So the Doctors have given us the precautions that we should not do. When a person they should not wash the person, they should not touch the person let them call 117. But some men are still doing it. So this is the reason is spreading and spreading and the other thing again is when they say this person is dead, the Doctors should come and check the person to ascertain that the person has Ebola and then they carry the person. But if when the person was at home they were touching the person, they were playing with the person and the person dies, so when they are going with the person they will not come back with the result until, if other person get it, there is no security contact, they come with other people in the community, that is how the Ebola spread, because the medical people are not able to tight those people for them for them to know that whether the person who died has Ebola, even the ones who do not know that they have Ebola. That is the spreading".

M: Yes, my brother?

R6: “I am going to add up like my brothers have just said, the first medical people. I am saying so because Ebola first started in Kailahun (first district with Ebola cases in Sierra Leone), you see, with one “Sowe” (*note: A Sowe is a title from the female traditional society indicating a women’s leader who plays a large role in girls’ initiation rites into the traditional society*). By then Government actually knew that Ebola had attacked Kailahun (first district with Ebola cases in Sierra Leone), why didn’t they isolate them quickly? It is the delay from the medical people that caused the spread of Ebola. Because if they had isolated at that time Ebola wouldn’t have spread.”

M: Ok, do you have any name that you call Ebola in your language?

R7: “Well we don’t any yet. We only knew about Ebola

M:Yea?

R8: “Some men still do not believe that Ebola is real”.

M: Do you know anybody in your community who does not believe that Ebola is real? And do you know why he or she does not believe that?

R1: “Because people did not know the secret of the sickness. Some say is money making”.

R2: “Is business”.

R1: “Business”.

M: Ok, yes?

R3: “Well as for me, to this time I don’t believe that there is anybody who does not believe that Ebola is real because people have been seeing how this thing has attacked the country. So, if there is somebody who still do not believe this Ebola, believe me, I have not known that person yet”.

M: Ok, yes? ,

R4: “This Ebola, why people are saying that they do not believe is because, government is pouring money, but this money, wherever money flows, it is criminal business. People have actually understood that government have said don’t do this, don’t touch, people have really understood, but why? People have sent money, but all that people do not agree. People are throwing money away, spraying chlorine all over the place and this and that, people are just going around vomiting, toileting! All this is affecting our people”.

M:Ok, yes?

R5: “Like for me I believe that Ebola is real but it is due to the way they exaggerate this disease and to the extent to put politics into it. And the politicization of the disease led people not to believe that Ebola is real. Because they were living a peaceful life, but the moment Ebola came, it was only one medical Doctor, a famous man who died out of this disease (*note, most likely pointing to the Kenema Lassa ward doctor that died*). They said Doctors were dying of this disease but if you look at the way they are going about saying that it is a slow poison injection that they are giving to people. The nurses and doctors are giving injection to people at the Holding Centres that could lead them to death. So in that confusion, that led many people not to believe that Ebola is real. But now, they know that Ebola is real, for those who understand the music can listen to the song that they sing about Ebola, Ebola, Ebola. Even though you do not know what is Ebola. So a lot of people know that Ebola is real, nut the way now they went towards the situation many nig men and the way we see people are moving in and out, it only means that is just money finding that they are after”.

M: Yes, my own brother, do you know anybody who do not believe that Ebola is real and do you know why he or she doesn’t believe that?

R6: “Well, I know that right now everybody believes that Ebola is real. We do not have anybody in this town who can deny that Ebola is not real because we now seeing the signs and symptoms of Ebola and we have seen what has happened. So they cannot say again that Ebola is not real.”

M: Yes?

R7: “Some men deny that Ebola is not real because at first when they spray the chlorine, people were dying, so now they are seeing the chlorine again and people are not dying. Some men are taken it that it was the chlorine that was killing people and they said it isEbola, and now that there is no chlorine people are not dying again. So some men are no longer afraid again, they can call 117. But in past they used to spray the chlorine, so you will not have mind to go. This has made people not to still believe that Ebola is real. That is the reason”.

M:Mhmm, Have you heard , read, or seen those Ebola messages on posters that they do hang and the ones you hear on the radio. Can you tell me some of the messages that you read or heard?

R7: “Well according to the pictures that they are giving us, they said that we got this sickness from animals like chimpanzee, we can also get it from body contact and saliva and from fluid that a person produce from parts of his or her body, and that if anybody bleeds and you get contact with that bleed you will be infected. We have been hearing that on the Radio and they used to come here to sensitize people. But all that brings about doubt on our minds”.

M: Yes?

R8: “Yes, this again is another salient point, eh, government has made it possible for us telling our brother over wires, over radio telling people about this Ebola, presenting papers on how do you get Ebola, then from where do you get this Ebola, ways of preventing this Ebola from spreading right now”.

M: So what are some of the messages on how do you get Ebola, how do you prevent Ebola?

R1: “Ok, ya, thank you. How to get Ebola is from body contact. When you contact with a person who has Ebola then you find out that you will get the repercussion. The prevention is that we need to stay away from that. If they sat you to avoid body contact, avoid body contact. If they say avoid washing dead bodies, avoid washing dead bodies”.

M: Yes?

R2: “Yes, they also said to avoid sex for three months then also you should stop eating monkeys and chimpanzee, and also you should stop washing dead bodies. If stop doing all that, you have prevented yourself from Ebola”.

M: Yes?

R3: “Just like what my brothers said they also came with a program called ABC which means ‘Avoid Body Contact’. We hear it in the media when they say ‘Do not touch a person, avoid shake hands, those things, and avoid eating baboon, monkeys and bush meat because they can transfer Ebola’. So I think if we stop all those things Ebola will finish”.

M: Ok, yes,

R4: “All those things, I think the least child in this country to (…know??...) that there is Ebola so the simplest way we can prevent Ebola is if you see a sick person with signs and symptoms you call 117 which is a simple number that everybody know”.

M: Ok, yes?

R5: “One thing there is hardship and then medical people said anybody who survived Ebola should not have sexual contact for three months.”

M: So eh, which message can you give to encourage people to carry their sick people to the hospital?

R6: “Because, the first message I go and tell the person is that since government has said that to keep a sick person is dangerous and will advise him or her to take the person to the nearest Centre as there is chance for him to survive. Why, because they told us that the earlier you go the more chances you have to survive. So that is the key message that I will tell the person. I will tell him or her not to discourage at all to take the person to hospital. The more time you have with the person, the more you get infected and the earlier you take the person to the Treatment Centre, the earlier the person has chance to survive”.

M: Ok, Yes?

R6: “Well em, A way also to spread this disease is like when they say you the man or the woman when you contact the sick and survive, you should not have sex with your partner for three months. But we are seeing some men and women here doing those kind of things, so all those things can lead to the spread of Ebola”.

M: So like, which message can you give to somebody whose relative is sick to encourage him or her to carry the sick person to the hospital?

R7: “Well, I will just encourage him or her to take the person to hospital because when go on time, you will have time to treat your own life to survive but if stay at home you will lose your life because they said when you contact the sick it can be in your body for 21 days en? If you stay at until after the 21 days you will infect the other people and we will lose your life because the time has passed. But if you go to hospital quickly for early treatment for sure you can survive”.

M: Ok, yes?

R8: “Eh, toward this situation, if they give me the opportunity to go and spread this message to everybody or if I see anybody with this disease I will first give him or her word of God because anything you are doing you have to put God first. So if I go to a house and I meet somebody who has contacted the disease, I will first pray for them and give them a counselling talks then I will be able to cajole (=to convince) them, then I will give them the message that they have to do this and this because if they do this they will prevent hundreds of people including the unborn generation coming. So if I am able to tackle this situation then the people will know that what I am telling them is right. It gives us faith, the business of pod will enter into their hearts and they will know that since God is with them. Anything whatsoever, we should not give up as we believe that only God can save us in trouble we encounter in this world. So if I go with the word of God to them and preach to them that they should do this for that is what God wants. Instead of you destroy hundreds of people let the one life go, when it goes is his wish whatever God marks will happen. So they will decide that since God is with their brother hr will bring us back”.

M: Ok, yes?

R1: “Ok, likewise, like me sitting so, throughout our quarantine, when we were there the symptoms I was seeing in people, they will lay down toileting, vomiting. I even called 117 and they came and took them. When they went with them they were there for two weeks. They only gave me call and told me that they were all dead. At one time my own wife was lying down and her pregnancy turned, eight months pregnancy, when the pregnancy turned I was confused not knowing what to do, I was under quarantine and there is no medicine so we called 117 and they came and took her away at around 5:00pm, when they took him away, they called me again early in morning and told me that my wife is dead. Where I was sitting it was only God who made me up. I went off at first. So as for me, anywhere I go. If I see any person sick, well government has said you call 117 or show him or her the road to the medical people. So that is what I have”.

M: Ok, so which way do you think is the best way to take these messages to the people because we hear it on radio, we see forms with message on it EBOLA, there are newspapers, so which one do you think is the best way that you can take these messages to the people about Ebola?

R1: “Eh, to my own understanding because even in this country the difference between the literate and the semi-literate are plenty in this country. So I thought it fit that the best way we can advocate for this thing is that, we have been advocating for long, we have advocated for Girl Child Advocacy. So the best way we think people can get our messages is what we are acting upon now, through drama, gather them and act concerts, but our people used to feel it when we act those concerts they cry that the message they are looking is a real thing and that it happened. If they do those kind of things, begin to act concert through TV and this or that, they go to local villages acting concert dressed in local attires, so the people feel that indeed are feeling the pain. So they will feel they will know that what we are telling them is something that existed. Even though it has not happen to us yet, but it exists”.

M: Yes?

R2: “Yes, the best sensitization is, brother we are all Sierra Leoneans and we need to have a brotherly feeling for fellow Sierra Leonean. If you understand that this person has Ebola, the message you can give to him or her is a polite message, an encouragement message that would encourage him that if he has a person who is sick of Ebola let him carry him or her to the hospital. That’s all”.

M: Ok,

R3: ‘like what my brothers have just said, there are some villages they don’t have radio, newspapers does not go there, if they go there personally and begin to advertise. So I think these are all ways they can do to try and finish in our country”.

M: Yes?

R4: “Like what the government has done to introduce the Contact Tracers, they have done a very good job. To introduce the Contact Tracers you find out that the work they are doing is very great because they contact the infected areas, the houses that has already been infected by Ebola or to contact a home wherein they have sick people but they are feel shy that if they take this particular person to the Treatment Centre he or she will lose. So these Contact Tracers go there they can talk to the person in a cool and fine way and give him or her the confidence that nothing is going to happen to him and nothing is going to happen to his or her loved one when they come to the Treatment Centre. So the introduction of the Contact Tracers is very important”.

M: Ok, em.. in case somebody has Ebola, where do you think the person can go first? Would he go to a “Meresin man” (=Traditional healer), or the (- - Name of interview village - -) Hospital here or to the Treatment Centre?

R5: “Well, we expect the person to go to the Treatment Centre”.

M: Well that is what you expect, but where do you feel the person would want to go first?

R6: “Like me my own brothers I took them to the Government Hospital. There was the first place I took them”. (*Bats fighting*)

M: Yes?

R7: “They can Take the person to the hospital first then the hospital, it is the medical people who can think fit that this person is an Ebola person so we have to take the person to CCC (=Community Care Centre) or ......”

M: What does the CCC (=Community Care Centre) mean?

R7: “The CCC (=Community Care Centre)?

M; Yes.

R7: “Eh Community Clinic Centre”.

M: Ok

R7: “So the hospital people who clinic people can now take the person if they suspect that the person positive they can take the person to the Centre. So now when a person if sick of Ebola they take the person to the Centre”.

M: Ok?

R8: “As the government has made it possible to have Holding Centres everywhere. There are Holding Centres in many Chiefdoms, if you find out that this person has Ebola encourage the person to take him or her to the Holding Centre, is only the Holding Centre that can detect that it is Ebola or it not Ebola. From the Holding Centre now you take the person to the Treatment Centre. The first thing is the Holding Centre”.

M: Ok.

R1: “How many chiefdom have Holding Centres so instead of loading the person on a motor bike to go with him or her to the Treatment Centre maybe he or she will get a way to remedy the situation that can be able to take care. So to abstain from all those things, they prefer to go to the Holding Centre because they have told him or her that there is the place where they are going to observe him or her. Then If there is the opportunity for them to be able to forge ahead but if you say you are going to pull it direct, direct then some people do not have affluence they find it difficult to move the person and maybe if the distance if far they do not care if you have money or not all they care for is money and not to save life, but if the distance is not far they can be able to go with the person there. No matter what happens the family will “hibhib” (=contribute) and do something”.

R2: “Yes my brother I would like to make an addendum to what my brothers have just said. Yes when somebody is sick and you have the person at home the first thing to do before ever taking the person carry him or her to the hospital maybe you do not the sickness he or she has, maybe it is Ebola or is Malaria or any other sicknesses in the country, so you found out that you should call 117 they have to prove you right or wrong”

M: Ok, let me ask you what are some of the good things and the bad things you are have been hearing about the Ebola Ambulance service?

R3: “Ah, I found out that some people were afraid to go to hospital. One thing that made them afraid is that they said when they get into the Ambulance it has a lot of chlorine that damages people so that is why many people are afraid to get into the Ambulance’.

M: Ok?

R4: “Eh, towards this process the way we hear people talking about those Ambulance drivers is not encouraging because some people say during the time when they are taking people from here to Kailahun (first district with Ebola cases in Sierra Leone) the Ambulance drivers stop on the way to drink, they do misbehave, they even avoid first to go with the patient to the Treatment Centre and prefer to go and sit down at the ghetto for them to enjoy forgetting that they have a person who is in a critical situation. Then Ambulance again, really the way they blow the serine is not encouraging. Some men have heart complain. But that frequent blowing of the siren will make a person go off. You see? But now when they have been talking about them in the media, now they have been minimising. This ambulance again maybe when the person they are loading there has no Ebola or maybe someone who has not been affected by this Ebola and you load him or her into that Ambulance, maybe he or she can contact the disease there, you see. But in all that before they call the Ambulance they call 117 before carrying him or her to the Holding Centre then they can do any test to prove that the person has Ebola. If you put him or her into the Ambulance, if God is going to save his or her life then he or she can save”.

M: Ok

R5: “The negative and positive difference of this people is, now if I may talk about the positive effect now this people when they come if they know that this person has Ebola, when they come the way of spraying will disturb the people who are at the place and for a person who is sick, that chlorine has after effect, if the person get the effect of that chlorine you find out that it is the chlorine that will kill the person. The negative effect now is that when they out into the vehicle, the way they rough you, the way they drive you can make you go out of mind you know. So these are some of the ?enen?.....”.

M: Yes?

R6: “Just like my brother have said, we are not glad, sometimes even if the patient is not yet dead but the way the behave to put him into the motor car. That is all”.

M: Yes?

R7: “Just like my brothers said, the Ambulance drivers do come, they do come to our house and talk to you fine when loading the person but as they go they have a stopping point where they “pap-pap” (= drink alcohol), they can be there “tayyyyyyyy” (= for long). Just like they tell us, from here to Kailahun (first district with Ebola cases in Sierra Leone) is a far distant. If the person is sick and did not carry him or her in that urgent way, when, tell me true that person will not survive!

M: Yes?

R8: “The Ambulance really they are trying because one thing about them is that if a person is sick and you call 117 by the time you think about it they would have come. But the bad thing that they do now is that when they come they cajole you, talk to you fine, talk to you politely until they try and put the person into the Ambulance. Wao! When they put the person into the Ambulance now then trouble begins. They will continue to blow the siren even if person do not want to talk you will talk because you will just hear “voom, voom, voom” (= blowing of the Ambulance siren). All that is not fine, it makes people discourage”.

R1: “Yes Sir”?

M: Yes?

R1: “To what my brothers have just said, I am going to commend the medium, the medium is so very important in the society. Like one presenter in a radio station said the Ambulances do take the Ambulance car to the very community well to be washed there. That is very bad because you find out that Ambulance has been infected by the virus so if take the Ambulance there to be wash at a community well, community water side you find out that the people in that community will get infected. And you find out that the glove which they used to take the dead people is also a presenter told the people that it is in the streets for sale. That is very bad of the Ambulance people, very bad”.

M: Ok, what about the Holding and the Treatment Centres and the Community care Centres; have heard any bad or good thing about them?

R2: (*Bats squall*)“Like in (- - Name of interview village - -) here we have not heard any bad news yet about them. When they held our brothers they treat them fine”.

M: Yes?

R3: “Like my brother have just said, we (- - Name of interview village - -) there is no problem we tell God thanks. Is only the first cases which happened to us, but we thank god when our brothers and sisters are trying”.

M: Ok, yes?

R4: “Ya, like for (- - Name of interview village - -), we have never got an experience like that, but at the Treatment Centre were complaining that the food which they give them at times is not sufficient and not too fine for them”.

M: Ok, what about the Burial Team, is there any good thing or bad thing that you have heard?

R5: “The Burial Team at first did not look encouraging to all of us because that even gave us zeal to go and stone the other Burial Team members. The way how they come and attack people, they way how they come to collect corpses. They met the woman’s corpse has already been washed and finely dressed. When they came they undressed the corpse and left her with only under wear then they dressed her with their own. All that, if you say when you touch this person you will get infected why do you go and undress the corpse again after it has been dressed? This of their plastic business, we don’t care about their plastic. People give their dead good thing to wear when they come they .give their own. Now people have the confidence that if I go to the hospital I cured. If you are here and you see the way they are treating your companion what do you think you will do? They don’t even care about you whether you are there or not there”.

M: So can you tell me why people do mix the body?

R6: “At first when they come, there was woman here when she dies all her thing were with her, when they came they off loaded the woman and dressed her with plastic. Things she was wearing never had contact with again. People are not feeling good about that. When they come if a person is dead they dress the person in plastic and throw it into the motor car; they do not care about how you are going to lay down or not. Even at the grave side is the same thing, they just throw you in the grave and send dirt over you”.

M: Ok, yes?

R7: “Like my brother has just said, I am going to talk about my own mother. When she died then I told my Aunty. She died around 4:00pm; she laid there until 5: 00. They came later, even when the woman was already dressed, they had to strip her naked, and we asked why are doing this? You met the woman wrapped in wrapper and you took her to bury her in your plastic. After that they use their rain boot to kick the dead body until they wrapped it. If I am stranding there and you play with the corpse or my mother or father being kicked, my brother in my heart I will not feel fine that one has affected us to this time. But, everything is government “.

R8: “Yes my brother this is a very important question. And again for the Burial Team, they have a negative and positive difference. Then if may talk about the negative difference, those people when they come to Bury someone they way how the treat the people is not good to talk, particularly the corpses we all know, we do not feel fine for those who remain. Like the example which my brother gave you about his mother when she died here the way she was treated we did not feel fine. And even when they sent to bury her that woman was the woman I saw, they were standing far from the grave and they sent her into the grave. We felt that and that is not the was. If you have come to bury somebody you have to be patient not to bury where some people will feel happy. Then if I may mention the positive effects, these are people who are our brothers and it is their right to help stop the spread of Ebola. You know that when somebody died of Ebola you should not touch that person when you touch the person you will contact the sick and at that time the virus has become very salient to transfer by that they have the right to bury. My only advise is that let them treat our people fine”.

M: Ok, yes?

R1: “As you threw the question to my brother when you asked why do they put the body into the plastic? The answer is that the Burial Team do that because they do not want to be infected by the disease that is why they put the body into plastic so that the disease cannot affect or infect them and then. The other aspect for them to off load the body and the clothes, because they have advised that when a person dies don’t touch it, wait for the Burial Team, although they have the wrong that they do, but if you look to the other side they are really trying, because they are instructed to this and they must do that to avoid the spreading of the disease. I am not against the burial in plastic but they are just doing what they are instructed to do’.

M: Ok, what about the Ebola lie 117, have you heard any good thing or bad thing about it?

R2: “Yaa, the good thing is that when you call 117 they respond but you find out that they give information that people call them and disturb them because some people call to cuss them, some people call to give them bad remarks. That is the bad thing the Burial Team is undergoing. Some person can just call 117 without any relevant thing to tell them. They are really getting problems with the community people”.

M: Alright, yes?

R3: “Eh, the 117 they are trying but sometimes they don’t try. Because they will give report and call 117 that a person has died in some place they will not go there. But they respond that they are coming but they won’t come”.

M: Ok, alright. Yes sir?

R4: “To me (*Not clear*)

M: Ok, how do the people of (- - Name of interview village - -) react to people affected by Ebola?

R5: “A person who has suffered from Ebola should be encouraged again in the home so that they can feel good back. Although the person lost their family, we can encourage him or her back into the community”.

M: Yes?

R6: “For kind like me my aunty and my younger one survived, we call them and encourage them back, we talk to them, we do not push them (*Not clear)*

M: Yes Sir?

R7: “Is like 117 is vice visa, they are sometimes good, sometimes bad”.

M: So like what is the good?

R7: “The good is that at times when you call them they respond quickly but time is not good they will not come”.

M: Ok, so like how do people react to Ebola survivors in your community? Do you anybody in this community who has survived Ebola?

R s: “Yes”.

M: How do people react to them?

R8: “When someone survived from Ebola we encourage him or her back in the home so that let him feel good back, although he has lost his family we encourage him back”.

R1: “These survivors who are coming from the Ebola Centre, we just took it that we are all one, we take it that we are in an epidemic war. So for them to be like a refugee to this sickness doesn’t mean we have to abandon them. So the only thing we can give them is to encourage them. We lost them for some time and we see them again we should be glad for them. You cannot lose your person and you see him later then you feel offended by that which means you have gone against the will of God. Because God said he makes us in his own image and likeness. So if God like us anyhow you commit against him he is ready to forgive us. also however our people has that kind of disease which is not their wish, so the only way we can do is to encourage them, give them encouragible heart, even if he is in difficulty, you can assist him just for him to be able to get a rest for him not to get another pain that would discourage his heart”.

M: Yes?

R2: “Like me my sister is a survivor. So she is not happy about the way she got Ebola. But when they came with her, we began to encourage her, we get water for her, we call her on a daily bases, we play with her and her give her fun. So I think if we are doing those things the survivors will feel happy”.

M: Ok, so like which message would you like to send to the other communities in Sierra Leone who have Ebola survivors but like they are pushing them out. Which message do you have for those people?

R3: “If I have to talk on this the message is to talk to the people in the community that those people are not bad people they are our brothers and sisters so we must not stay away from them. We need to be close to them. Since they had had the virus, they have been cured so no need for us to treat them nonchalantly. So we need to draw them towards us. That is all about it”.

M4: “Me the message I would like to send nationwide is that Ebola disease is not a person’s wish to get the disease so if person has the disease you have to know that is not his fault, so if you become a victim to provoke that person I take it that you are not a Godly human being, so to avoid all those things if you do not have to mock the person. If anybody so far mocks a person is better him or her abstain from those things, we should see them as brothers because we are all living the same way. The same life which they have is the same life that we have also. No matter the situation they encounter is not their wish. This Ebola is anybody going to take it and place it on him or herself. If it was for money everybody would have just come to take it but this is not money finding situation it is an uncertain situation in which we find ourselves, nobody was expecting it but when it has come now we have nothing to do but to just say I must survive. So if the person survives and comes back to the society we must take the person he can give us more ideas that this is what we should do. This is the idea we that we don’t need to push them. But if we drive them how are we going to educate the others how to handle those things. So I think that we should ask those questions and encourage them to tell us how they were able to undergo the entire situation. I think if we take those steps, we will be able to get health in the community”.

M: Yes brother, so which message you would like to send those communities who do not treat Ebola survivor’s fine?

R5: “Like me when my sister survived from Ebola, when we go to any area and a person is sick they should take that person to the Health Centre, so that they treat the person, get well and come back to the community”.

M: So like when the person comes back to the community, when can the community do to make sure that the person feels at home?

R6: “Like when the person comes back we will ask him what kind of treatment did he or she get at the Health Centre so after explaining we will encourage him or her, talk to him or her and we give him or her everything he or she wants”.

M: Yes?

R7: “I will tell the people that survivors are good people. They have better chances of survival than us. That fact that they have survived and come back we need to encourage them as brothers and sisters, talk to them encourage them for them to forget what has passed. Even though it is hard to forget what has passed but the encouragement can make the person not to feel discourage too much”.

M: Yes?

R7: “In response to the music by my brother Nasar Ayoub on survivors, I think we all have to do what Nasar Ayoub is doing. We have to stop stigmatizing them, we should stop pointing fingers at them. These are good people who can show us how to take care and prevent Ebola”. (*note: most likely referring to Nasar Ayoub’s song with title “Salone will rise again”)*

M: Ok, so have you heard about any treatment for Ebola that will come soon?

R8: “No”.

M: Mhmm?

Rs: “No, no, no”.

M: Ok, have you heard about any new way to prevent Ebola?

Rs: “No”.

M: Ok, or have you heard about any new vaccine which they are going to bring for Ebola in the country?

R1: “We have been hearing about vaccines but we don’t know the name of the vaccine that is coming to treat people because they were telling people that we were going to get vaccine but this 2015 WHO has been making sample towards the situation. They have been testing, like we heard that in Liberia and Guinea they took the prevention and went for the test and you hear that people survived, we also heard that 17 people in Senegal or elsewhere went for the test and 14 survived. So we are hearing about the vaccine but we do not know the name. And we do not know whether it has come or not”.

M: Ok, so what are some of the common thing that you discuss about or hear about Ebola? And do people agree with some of these things?

Rs: (*Silence)*

M: What about the prevention, do people agree with it?

R2: “Well, according to people even when we are here medical people will come with tablets and say use this, I will use it and see that it is not the same because we plenty in this country that are affected by Malaria, Typhoid. Some people it can be chronic in their heart and they begin to vomit and toilet. But the medical people “sabi” (know). When they ask you to use this and this, if you use it you can see. So that is all”.

M: Ok, yes?

R3: “This vaccine we are trying to talk about..”

M: No, I am talking about the common thing that people discuss about Ebola?

R4: “The common thing that we take from these things especially we the boys, we take it that we have to abstain from sexual intercourse because this is most dangerous issue not to spread this Ebola business. So if we the men educate ourselves that we should stay with one partner, even if you are staying with one person, you should not do it frequently. Maybe by the time you leave her for few times she can go and get contact with other person. They are also bringing tablets for us here they say it is Malaria tablets and when we take them we see that though it has a side effect but we can cope with it maybe that will prevent us from doing other things so far”.

M: Yes?

R5: “Also when we are interact with ourselves, we advise ourselves that when you come from toilet you have to wash your hand with soap and water. This are some of the ways we do interact in group”.

M: Ok, yes?

R6: “Some men would try to touch me and I will say ay “oman” (= my friend) don’t touch me there is Ebola – there is the ABC (= Avoid Body Contact)”.

M: So, do you have anything on Ebola which you would like to understand fine? and how would you explain these things to the people?

R7: “Yes, this is another very salient question.eh mainly for these survivors. These survivors lie I said just now people are thinking that since they have survived they should not be close to them, if they stay with them they can get the virus. But the medical people have told us that any person who has survived Ebola that person will never spread virus again. Except because they told us that after the treatment all the virus would have finished in the body and only remain in the private part. So the only advice I can give now, if you have a boy friend or a girl friend who has survived has to be very careful with intercourse’.

M: Ok, yes?

R8: “The only thing we can do to be able to solve this problem is that we should be ready to adhere to the precaution which they give us because you as human being if you are not sorry for your own but you have to remember that there are other people. If you follow the sweetness of the world you will be destroying yourself. This world is vanity, no matter how you keep yourself in this community you have to go. So, the only thing we can do to abstain from those thing is that we have to adhere to the medical precaution they give us. If we are able to do this nothing can stop us from finishing this Ebola.’

M: Yes?

R1: “Just like what my brother said, some people can still underrate saying that Ebola is about to finish so they can do anything they like. So we still have to take the precaution and see what will happen. Avoid body contact, when a person is sick you should report. I think all these things if we are able to do them I am sure Ebola will leave us”.

R2: “Yes my own brother, what is it about Ebola that you would like people to understand more? And how can you tell the people

R3: “Well, the way they have given us the prevention of Ebola. If I see any of my companion want to do things like that I will advise him to stop sexual intercourse and also to stop seeing a person whom had not known before and don’t touch the person, if he sees the person vomiting he should not go closer to him. I will tell him to avoid all that”.

M: Yes?

R4: “I will just continue to advise my fellow citizens to continue to hear the advise the medical practitioners are giving until the day when government and WHO will declare that Ebola has left us”.

M: Ok, yes?

R5: “To make an addendum to what my colleagues have said, the main thing I am focusing on is the gathering, people are still gathering, they cluster themselves. So through this gathering they can easily contact the disease. So I am advising person to abstain from gathering”.

M: Yes?

R6: “The only thing I am saying is that let them abstain from sexual intercourse that can lead to Ebola. So let them abstain first”.

M: Ok, yes brother?

R7: “Just like brothers have said, personally I am still observing myself. I don’ have contact with woman, I don’t keep a woman until WHO declare that Sierra Leone is Ebola free, thenI will know that I am now safe”.

M: Yes brother?

R8: “Even though government has declared that is free to travel from place to place, but I am telling them that even the criss-crossing in transport is risky and dangerous as you don’t know who has the virus who can later transform it to you.’

M: Ok, well, I want to thank all of you and I hope next time when we come again we can see.

**ADDITIONAL PART OF FGD, OBTAINED BY COLLECTOR 1 AFTER CONSENT IN PERSON with all original participants, February 2015:**

M: Yes Sir, what I want to know is the belief, why do people believe that Ebola is like Bondo (=women’s secret society)?

R1: “Well Ebola to me is not like Bondo (=women’s secret society) per se, but I believe that in this State of Emergency in which we are I believe that nobody should think about Society yet, because think about society is like think to associate and when you associate with people definitely you have to contact the disease and that is why we are saying that all society business, be it Bondo, or Poro (= men’s secret society) everything should be shot down and after the Ebola we can commence”.

M: This Bondo (=women’s secret society) and Poro (=men’s secret society) and Bondo you are talking about, what are they?

R2: “These are Societies, they are our own traditional Societies during which people gather and make merriment and do some other activities but in this State of Emergency on Ebola, I think we have to refrain from these things because there are a lot of things which needs to be displayed and involves touching so definitely if we mingle with those things of cause Ebola is our own”.

M: So for now is this Society business is it still carry on or not?

R2: “It is not carrying on, everything, even the Poro (=men’s secret society) we have what we call the ‘farr’, by now you should be seen some dancing but no, because of the State of Emergency for the Ebola”

M: So it is the State of Emergency that blocks all these things?

R2: “Yes”.

M: What I want to know again from you as Male Young, have you been hearing of any secret burial?

R3: “Well it used to happen”.

M: Can you give me examples?

R3: “Like in one village they call (- - another community in same interview district - -), people were dying there but as you know because they are deep in the interior they take advantage of the situation somebody dies they bury the person secretly without reporting, it happens in villages that are far off the street, those closer to the Street can be afraid because of the law and people keep passing”.

M: But who are the people that carry out these secret burials?

R4: “Is the community people, they bury their loved ones, they will not allow other person to bury their Father. I can give you one example, we went on a field trip in one of the villages, we were passing by but we saw the mood, we suspected that there was a funeral but the people pretended as if nothing has happened but the way you see the atmosphere where everybody was sitting quiet, some feeling sorry, some were crying but when you attempt to look at them they will keep quiet. When they saw us they thought we were Contact Tracers, they all kept mute. We only got to know that somebody had died there was through the toilet when we met three to four people standing in the toilet washing somebody. We met the gathering but when some of them saw us they pretended as if it is not a burial. And that is the thing starts especially for those who are inside so you see they chance”.

M: But do you know the reason why they do that?

R5: “Oh yes, there are many reason because after when I suspect the situation I called one young man and interviewed him about what had happened and he denied saying that nothing had happened but I told him is a lie, and I asked him why are some of the people sad and the others happy? People take of the risk because of the respect they have had for the people either the father, the mother or your beloved one, so definitely some people do not know that when somebody is dead he or she is dead. They still bury the person with the that respect that can make the person go to heaven or that makes him the survivor feel convenience that is why they do that. Trust me when you bury the person that respectful way that can be fitting for the person, even you the survivor will remain convenient, that is the only reason because it is something that we have been accustomed to, we are now used to it that when my person dies I have to pay the last respect to the person. But in this State of Emergency it is not happening. There are people if you say you are going to their tradition thy will lose their life. If you say you are going to change the state in which he or she is he or she is, they will not agree, instead he or she doesn’t mind to lose his or her life or he or she follow the person who has died. That is what some of them tell us that if they say they have to bury his father then, but if believes that his father did not die of Ebola and so, nobody cannot prevent him from burying his father. So I decided to tell him what he needs to know that you cannot take your father back to life”.

M: But why do people think that chlorine kills or it is harmful to people?

R6: “Oh definitely it is very harmful because it has a stipulated time. Every medicine has a dose, every medicine has a targeted time within which it operates in the human system and I believe that this chlorine, they told us that if they spray your room you have to wait until three days without entering or sleeping in the room, according to what the Medical Practitioners told us from Ministry of... Primary Health Care after the three day any virus or any other thing that is there would have avoided the place, then you can make use of the place. But what happened when the people were coming, especially here in (- - name of interview community - -) where they come they just spray the environment, even the pots they spray everything but what is causing the massive death is what I am telling you, up to 23 death in just one house”.

M: 23 people died?

R6: “In the same house, the reason being that we had one of our brothers here who is dead now and he married my sister, he didn’t know that this chlorine has a negative impact if it is too much and if you fail to adhere to its own protocols, he thought it was a Shelltox (brandname, commonly used insect/mosquito repellent in spray flacon for inside rooms/houses), so he was just telling the man to spray every corner thinking that the spray was to kill mosquitoes and the man pumped the thing thoroughly and the very night they slept in the same place. They told to lie down and sleep and that nothing will happen”.

M: “Who told them so?

R6: “Those who came, the Burial Team, they told them to sleep and that nothing will happen”.

M: The same day?

R6: “The same day and so because of that saying the people trusted them and they slept there. By then, my sister was pregnant and after that they begin to see her breast filled up not knowing that the chlorine has killed the baby in her stomach but they took her to (- - name of headquarter town of the interview district - -), when they tested her the baby inside her stomach was dead and we have also lost the woman during that same time. After the woman died, later the husband who was saying to pump the room also died and it continues. And so the people looked at the whole thing and concluded that it was not Ebola that killed the people but it is only because they are not sensitizing people to follow the right procedure of the things”.

M: “And who can you point fingers at?

R6: “I am pointing fingers at the medical people because if they way they are sensitizing people about this thing, about this Ebola on air, is the same way they could have sensitize people about this chlorine so that if they spray into your room will know when and how to enter there but they did not do that people so people blindly enter into their rooms and use the pot which they have sprayed maybe even the pot she will not wash it, even the cover she will not clean it.

M: How do you think can be a reliable messenger for this Ebola messaging?

R7: “Somebody whom you trust who can tell you the truth”.

M: Somebody who you trust?

R7: “Yeah, I am just supporting you, for you to be able to get the truth about Ebola, the only people whom I believe are the Contact Tracer, they are very important because they are in our midst and I believe that whatever they think is affecting us or has a link with Ebola they will alarm it. So we trust them a little. They are the only people we trust now”.

M: But who are the most reliable people now you can trust that they can pass messages to that the community can get it?

R7: “One, the youth”

M: The youth?

R7: “Yeah, the youth, the youth have role in this. Two, the Stakeholders”.

M: Who are the stakeholders?

R7: “The stakeholders like the Headman and any other important person in the environment, who people can respect, who people can listen to, I think it is very paramount and then the religious people. The religious people go to church and people listen to them and I believe that if they preach about Ebola they can adhere to that”.

M: Eh, which way do you think is the best way to channel information if somebody wants to give out information?

R1: “To me the only channel which I think is necessary is the radio”.

M: The radio?

R1: “Yes, the radio is very, very important because it goes far and wide”.

M: So your own community here, you think when they use the radio is the best channel to pass the message?

R1: “Yes the radio, which is one other area”.

M: Like which radio station?

R1: “Well like all the radio station all over the country people do listen the radio, (- - 2 names of local radio stations - -) and so on and mostly again (- - name of another local radio station - - ) radio again like ( - - name of 2 other local radio stations - -), especially the most interesting Radio which people like to listen is Radio (- - name of one of the previously mentioned local radio stations) because they are always talking about Ebola, they sensitize us about Ebola and give us update about and then people are always adhering to everything so people did not see the need again for more sensitization because people have already learnt about Ebola and so there is a lot of sensitization going on about Ebola giving to people, so there is no need again to poke our nose into Ebola discussion but only it is the way out now”

M: But when you are passing the message, who do you think the people believe more?

R2: “Well they believe the young people”

M: Are you saying they believe the youth?

R2: “Oh yes, they believe the youth because you an elderly somebody and you are respectful, when you see your own biological child telling you about Ebola, believe you, it will touch you because you have more sense that him, you are supposed to advise him or her but if she comes to advise you I think you have to adhere to the advice, because you are supposed to advise him”.

M: Ok, I understand that people were getting Ebola messages that bring doubt into their mind, what were the things that created the doubt?

R3: “In the area of the posters?”

M: Yes?

R3: “Well the posters, like for us who are a bit educated you can look at it and understand but that lay man who did not go to school, will only look at the picture but the writing cannot tell him or her anything so I
