# Supplementary material for: Development of a set of community-informed Ebola messages for Sierra Leone
Source: PLoS Negl Trop Dis. 2017 Aug 7;11(8):e0005742. doi: 10.1371/journal.pntd.0005742 (PMC5560759; doi:10.1371/journal.pntd.0005742)
Supplement: S1 Appendix — (ZIP) [file pntd.0005742.s001.zip › Ebola messages - FGD and interview transcripts/R2HC Ebola Fieldwork 1/R2HC Ebola F1 FGD-MAYOU-Rural2.doc]

| CODE | **R2HC Ebola F1 FGD-MAYOU-Rural2 (rural focus group discussion** |
| --- | --- |
| DATE | January 2015 |
| DURATION (minutes) | 55 |
| Collector nr | 5 |
| LANGUAGE INTERVIEW | Krio |
| **TYPE FGD** | Young males |

***PERSONAL DATA PARTICIPANTS***

| Nr | Sex  (*F/ M*) | Age  (*in years*) | Education Level (*e.g. none, Primary, secondary, tertiary*) | Language (*e.g. Mende, Temne, Krio)* | Religion | Job / Employment (*how they earn their living e.g. farmer, teacher, trader*) | Role in community  (*e.g. youth leader*) |
| --- | --- | --- | --- | --- | --- | --- | --- |
| 1 | M | 18 | Primary | Limba | Muslim | Mason | None |
| 2 | M | 20 | Secondary | Limba | Christian | Student | None |
| 3 | M | 20 | None | Limba | Christian | Mason | None |
| 4 | M | 23 | Secondary | Limba/Krio | Muslim | Student | None |
| 5 | M | 21 | Secondary | Limba | Christian | Student | None |
| 6 | M | 18 | Secondary | Limba | Christian | Student | None |
| 7 | M | 17 | Primary | Limba | Christian | Student | None |
| 8 | M | 19 | Secondary | Limba | Christian | Student | None |
| 9 | M | 22 | Secondary | Limba | Muslim | Student | None |

**TRANSCRIPT : (M = Moderator, R= respondent, R1= first person responding to a question, DOES NOT correspond to numbering used in Personal Data!)**

M: Can you tell me how has Ebola “yagba” (affected) you in the community? Have any of you seen or know anybody affected by Ebola?

R1: “Yes”.

M: Have you seen? But how has Ebola “yagba” (affected) you in the country?

R: “Actually, it is difficult; it has dropped business, we as student we cannot forward our education, right now it is going about one year. Then our people, where they used to get is blocked; no market, so even food business is a problem for us’.

M: Yes my own brother?

R2: “To my own idea, Ebola has tormented us really. It has, a lot of businesses has fallen”.

M: Have you ever seen a person with Ebola?

R3: “Yes”.

M: So why do you feel Ebola spread in Sierra Leone?

R4: “Yes, is because of the denial we have in this country. Then two, we do not take order, then three, when they said “No Touch”, when a person has the virus you should not touch that person.

M: Yes my own brother?

R4: “Well to me, I give the fault to the government. Because when the sickness reached Sierra Leone and entered Kailahun (the first district in Sierra Leone with Ebola cases), they should have rushed there and blocked the road; the sickness would not have spread this way”.

M: Yes my own brother?

R5: “Is the denial that we have, because even when they said we should not touch, some of deny - we still touch the sick, that is why Ebola has spread”.

M: So, in Limba do you have any way you call Ebola?

R6: “Yes”.

M: How do you call it?

R7: “We say “Mbola”.

M: There are some people in this town, in this community who do not believe that Ebola is real, do you know anybody who does not believe that Ebola is real and why do you think that person does not believe that it is real?

R7: “No, I cannot detect that, I have not seen that person yet”.

. M: Yes, I saw your hand up?

R8: “At first when we first heard the sickness, many people were denying, but for now people have believe that it is real, then we also have a patient which we saw with our own eyes, so people now believe’.

M: I know you have seen some of the Ebola messages on posters which they paste on the wall, you have seen it on television like when you go to (- - name of the district headquarter town of the interview district - -), you have heard it on radio. Can you tell me some of the messages that you have heard so?

R1: “We have actually heard that when you are affected by the sickness you begin to feel weakness, blood will come from your nose, blood will come from your mouth and free stooling (= frequent stools, diarrhoea), then vomiting. Then they are advising that when a person shows these signs you should not keep the person, you go with him or her direct to the nearest Health Centre. So this is what I have for now”.

M: Ok, yes?

R2: “Then, they told us that they are showing the virus, but we here, we don’t have TV, we only get the information so”.

M: You hear it on the radio?

R2: “Yes, you should not touch, any kind of way, even if somebody is sick you go with him or her to the Health to do the operation”.

M: Ok, yes my own brother?

R3: “Because we have seen the Ebola when it came into this our country. I saw this of my brother here; the sickness affected him and they went with him. He was there nearest (= close) to ten months. So that is everybody should not deny because everybody have seen it now”.

M: But what are some of the messages that you have heard, seen and read which tells you about Ebola which ones have you seen or heard?

R4: “I have seen it on paper and I have seen the symptoms when the virus attacks people”.

M: Ok, so which message do you have for people who keep sick people at home? Which message can you give to the person to take the sick man to the hospital?

R5: “Eh, actually is to advise the person. When you go you talk to the person that the person is not well and you are still closer to the person, so that best thing I want to you to do is try and call 117. If you cannot get 117 direct, you can go to the Health Centre and tell the TBA there to come and collect the person. Then you who is closer I will advise you to wear gloves, then you can give the person that jelly water if there is no ORS because to sustain his life before the Ambulance come to collect the person but you should not keep any sick person or dead body”.

M: Yes brother I see your hand?

R6: “Why I believe is because of the advice which they gave us for this Ebola. When this sickness came newly, they printed some poster where they draw a human being with blood oozing from the mouth, vomiting blood, sheet (=defecate) blood but they came with the method that we should not touch, we were on that method and we are still using that method”.

M: Ok, well if you go to somebody’s place to talk to him and convince that person not to keep sick person at home let him take the person to the hospital. How can you go and talk to that person? Which message can you give the person to convince him to carry that sick man to the hospital; do not keep him or her at home?

R7: “Well to convince mortal man (=human being) now - you can’t do it by force, you don’t have to say “paopa” (= at all cost) you have to do this, you just have to “bell nor mor” (= cajole, convince) him and encourage him little by little (= bit by bit) and tell him that what he is doing is a bad thing because if he has not seen but you give him some examples that has happened, he can take that advice”.

M: Ok, yes?

R8: “When somebody is sick and go to advice the person he or she will take you as enemy, the person will say you want to spoil his life”

M: But like .how can you talk to that person?

R8: “You can go and talk to him and advice the person that this is Ebola time and government has said that if somebody is sick you have to report it, if you do it that way and leave he will make you enemy”.

M: Yes?

R1: “You have to go to the family and tell them that this sick man that you have, please don’t touch him call 117 or you go with him at the Health Centre. That is what I have”.

M: Yes, my own brother, so which message can you tell a person who keeps a sick man at home to take the person to hospital?

R2: “You just warn the person not to touch him, call 117 to come and take the person and go with him to the hospital”.

M: Yes?

R3: “That we should not keep people at home but to call 117, if you take me as enemy I will leave it so because some of when they told us about this we did not believe that is why when you tell your companion not to touch a sick man he or she would not believe”.

M: Like which way is the best way that you think people can use to talk to you about how to give you Ebola messages because you hear it on radio , some men have phone and they send text message. So which way do you think is the best way for the message to reach the people who are far away from in the village?

R4: “Well thank you for asking that question”.

M: Ya.

R5: “Well, me before I answer that question I heard it through radio. Like what they have done to you now, to send you to those villages to talk to people

M: Ok.

R5: “Then you can see the person “gbain” (=live). So you cannot just hear that you should not touch and so, so. As for me before they talk about it on the radio, let them send people to the villages to go and sensitize them. For me that is best thing for me”

M:Ok, yes brother?

R6: “Is just the same thing like what this boy said because if you are in station, you cannot believe. But like when government have sent you (=the moderator) to come and enlighten us is fine”.

M: Ok, yes?

7: “Well I support what my brothers said; you have to come around to sensitize us?

M: Yes?

R8: “Some men except they see with their own eye before believing that Ebola is real. Especially those who talk about it on the television they can give more information on that”.

M: Ok, so, in case..?

R1: “I want to add to this man’s point because this man’s point is the most important point. Like those who talk on the television, when cholera came there were people moving around with the television (=video) they put it down for people to watch, then you can see that this clean. If that is what they are doing to form that kind of group and go to the villages to play those television (= video) for people to watch; they can know that Ebola is real. But if they don’t do that , they just go about talk on television, even when you come to talk to us; some men can take it to be true, some will not believe but if they see it now face to face, they will believe that it is true”.

M:Ok, just in case somebody has Ebola, where do you think that person will go first? Some men would want to go to “meresin man” (Traditional Healer), or to the Health Centre that has been there while some men would want to go to the new ones they have built to treat Ebola where do you think if a person has Ebola, where can the person go for treatment?

R2: “To the Health Centre”.

M: Which one, the new built one or...?

R3: “The newly built Centre, where the Chinese (*note: most likely referring to the new big hospital close to Freetown, built/run by the Chinese*) work”.

M: Ok, what about you where do you think people would want to go?

R4: “Just there”.

M: Where, the Health Centre?

R4: “Yes, the Health Centre”.

M: So now I want to tee me the good things and the bad things you have heard about the Ebola Ambulance Service? What do you think about it?

R5: “Well those who are driving the Ambulance have caused so many problems .The way they speed with the Ambulance, they have caused so many accidents. At times they blow the horn of the Ambulance (= siren) when there is no patient inside it. I have witnessed it one time, the other one loaded charcoal inside Ambulance just by ( - - name of a road to a neighbouring district - -) road; the driver came blowing the horn when he reached the check point they gave him way and when they watched the back of the Ambulance it was full of charcoal. The police wanted to trail him that day but they forgot about him. They gave the report over the media and they advised them not to blow the alarm (=siren) again whenever they are going for patient”.

M” Yes, brother?

R6: “The Ambulance you are talking about, when we were at the hospital they took us to Kailahun (= first district in Sierra Leone with Ebola cases), but when we were going before entering into the motor car (= Ambulance) they spray first and when we are in there our heads ache then again the kind of way they speed with the motor car (= Ambulance) even if they meet gallop, they will not check (= when they meet bumps they will not reduce their speed). When you talk they will “cuss” (= abuse) you. That is what I have”.

M: Ok, yes?

R7: “Just like how the boy said, so it is because some of them when they load a patient they don’t check (= don’t watch where they are going) anywhere they reach. Because the other time they came and bury one patient here, it was the same thing back when coming, they nearly had an accident”.

M: (*Moderator coughs)*

R8: “This Ambulance people what they are doing is good. Because when there was no Ambulances it is difficult for us to bury the dead except we had to manage to bury ourselves and that how the sickness spread. But when the Ambulance came when a person dies they call the Ambulance; they come and take and bury it. So that is good I see that these men are doing”.

M: Yes brother?

R1: “The other bad they do is when they come to bury, after burying instead of putting their clothes into the grave before covering the grave, it remains with them and they always drop it along the road. When the children are going to (- - name of district headquarter town of the interview district) to sell wood if they see it they will take it because they like playing with it, that is how the sickness spread’.

M: Yes?

R2: “The other bad they do is like those suburb villages when they enter there to bury; they will just drop the dead body and leave it, those in the town who do not have protector (= PPE) will bury that man’.

R3: “Yes, I want to ask”?

M: Yes?

R3: “What my guys have said I want to add small but I am coming to about the good first about this Ambulance and the Burial Team. Actually they have tried. In fact when they normally come there are times there can be no road so they block here after here they and bury the body so that is the good way. Then the bad way is the speed that they take when they take a patient, sometimes even if the person does not want to die; they will cause the person to die because of the too much speed. They don’t check for gallop they just go and “werr” enter “gbum”. That is how they act”.

M: What about the good things and bad things that you have heard for the Treatment Centre, Community Clinic Centre (CCC), and the Holding Centres what have you heard about them?

R4: “Me like for me so, when we went there, as for me they did good to me there. When I was pressed by the sickness they gave me water to wash, they gave me food to eat, they gave me medicine to drink. This is what I have to say”.

M: Ok?

R5: “Me I used to get some grumbles going on between some of the survivors. They said (- - name of district headquarter town of in the interview district - -) Health Centres and Kailahun. One survivor was grumbling that the way they treat them in Kailahun (= first district in Sierra Leone with Ebola cases) is not the same in (- - Name of district headquarter town of the interview district - -) here. They said (- - Name of district headquarter town of the interview district - -) h0ere they treat them as if they are not their relatives or not their brothers. But in Kailahun when they go their they “bayo-bayo” (=pamper them, they gave them food three times per day any kind of food they wanted they gave it to them”.

M: Yes my brother, what are the good things you have heard about the Treatment Centre, the CCC (= Community Care Centre), the Holding Centre?

R6: “As for me what I have to say, at first when Ebola came when they carry you it is not easy for them to give you tablet just like that. But thank God we can get tablet, when they go with you they give you tablet.”

M: (*Moderator coughs*) What about the Burial Team, what are some of the good things and bad things you have heard about them?

R7: “The Burial Team at times they do well, at times they don’t do well. We, one case here when we lost our brother, when they came to bury they only put the body into the grave and left it. They who have the protection gloves did not cover the grave. They asked us that we should cover the grave and that we must use shovels with our bare hands. ?Ewe? made the argument here, later they went, brought some gloves and left it with us. They only put it inside the grave and leave it there”.

M: Yes?

R7: “Some of the bad things which those working at the Health Centre do, when this sickness came newly when you go with a patient there, be it Ebola or not Ebola they will not come near to him or her they will be afraid to treat the person. When Ebola came people died because of this kind of style. When you go with a person they don’t touch him or her, they don’t even come near to him or her. Even if the person is crying for water they won’t give you. That is a very bad thing which led many people to lose their lives”.

M: What about the Burial Team, what are some of good things and bad things you have heard about them?

R8: “The good things I have been hearing when the sick had not come yet, people used to bury for them, you did not have protectors (PPE) or gloves to wear to bury from the time when they put that in place the sickness has reduced and the amount of Ebola patients we used to get has reduced”.

M: Yes, brother?

R1: “Some of the bad things that they do just like what my brother said, that was what they did again with one guy. When they sprayed the guy and the grave they said we have to go and cover the grave so our uncles too denied that those/they who are protected should go and that because they have the permission to come and bury; so we told them that if they leave it there we call for them that was why they just covered it like that and went”.

M: What about the 117 Line what are some of the good things and bad things that you have heard about 117?

R2: “117 so e times the good side the sooner they call them they answer and come immediately. But the bad side, sometimes when you call them the phone rings and rings they will not come on time or it will take long before they come”.

M: Yes?

R3: “I want to support what my brother just side, 117 at times you can call them and they answer you but for them to respond to your call it won’t be easy”.

R4: “Just like what these ones have said when you call 117 they delay you some time when you call they wait until let’s say 2 O’ clock, 3 O’ clock, 4 O’ clock or sometimes at 5 O clock before they come to bury”.

M: Ok, yes?

R5: “Like what my men are saying, 117 has a lot of problems especially in this work. Because they call for a patient let’s say today they will not come until maybe tomorrow. Maybe by the time they come the person might have died and when one dies there is no way you can do even when they are saying we should not touch; you just have to do it and when you touch you get the sickness. That is the only problem they have when they do not respond to time when you call them”.

M: Ok, how do people in the community react to those who are survivors of Ebola? Do you have any case like that here?

R6: “Like here”?

M: Yes.

R6: “The time when they brought me from Kailahun (= first district in Sierra Leone with Ebola cases) they encouraged, they touched me, they played with me they were glad to see me but some were not happy”.

M: Some of your relatives were not happy?

R6: “No”.

M: How do you react to a person who has survived this Ebola?

R7: “When they came with this boy we were all happy in this village because he has survived from this sickness so that makes us happy. I don’t know for the others but we the young ones coming up we are happy for him”.

M: Yes brother, I see you want to talk?

R8: “No really like this boy when they brought him into ( - - name of a village in the area of the interview village - -) XXXX XXXX, when they came with him we were happy. We don’t know for other people but we the young ones are happy”.

M: Yes?

R1: “Like this boy is a boy who gives respect to “big one” (= elder) he quickly talks to person”.

M: So which message would you like to send to the other villages who do not welcome those who have survived Ebola?

R2: “We talk to the person and encourage him or her back”.

M: Which message would you like to send about a person who have survived Ebola, what should they do to that person?

R3: “We just tell them that any person who survived this sickness any time he or she goes to his or her village they should welcome him or her, be happy for him or her”.

M: Ok, yes?

R4: “Any survivor so far, as long as he or she survived, when they come to collect the person your heart will be broken but I hope the message I am sending to the other people so far is that if they have any survivor let them talk to him or her fine if they have anything to give him or her let them give him or her, let them play with him or her like they used to do before. So I don’t if a survivor come to the village let them look at that way. The message I am sending to the other people is that I am begging them any survivor so far let them put hand under him or her let them play with him or he like they used to play (= interact) with him or her”.

M: Yes brother, so which message can you send?

R5: “Well the message I will send is that any person who sees a survivor must welcome the person, should laugh and play with the person anyone who class him or her that he or she has Ebola let them fine the person Le 500,000 (16feb2015 about 114 USD).

M: Yes?

R6: “Well as for me the day they brought this boy we were so happy because two of them went but the other one did not come; so like he felt so sorry because all of them went - only he came back. So we look glad for him but we did not see the other one up to now; so we are not happy for that”.

M: So like which message can you send?

R6: “We are getting information that if somebody survived Ebola that has strong body more than we who do not have any sign of the sickness. That person now has soldiers who fight in his body (*note: most likely referring to antibodies*) so we don’t need to be afraid of him we have to welcome”.

M: Ok, yes?

R7: “Some men who are married now when he falls sick and they went and cure him when he comes back they advised him not to go close to his wife they said when you are sleeping you should not share the same bed until for three months”.

M: So have you heard about any new treatment which they said have come or will be coming for the treatment of Ebola? If you have heard, what have you heard about that new treatment?

R8: “Because like Malaria, diarrhoea and eh.. when they said if you have Malaria you have to complain so that they can give you the treatment then if you have diarrhoea you can go for the treatment, typhoid if you have that you should go for the treatment’.

M: Yes?

R1: “Ya, the other treatment is the Malaria medicine which they give us although it did not reach here but some people praised that medicine although some people said they have brought the medicine to come and kill them but some people were praising it that it was good for them. The medicine when it came the amount of patients they were getting reduced. So it is the other treatment that came after. The other is the marklate (=vaccine, vaccination) for Ebola”.

M: What and what did you hear?

R1: “Well they said they will come and give treatment by marklate to anybody for Ebola. Even if you are disturbed by Ebola, it will not affect you again”.

M: So have you heard about any new way that somebody can fight Ebola after from when you have heard about this “Avoid Body Contact”, they have another slogan which they say ABC, Avoid Body Contact, they have APC, Avoid People’s Compound (*note: APC is also the acronym for the government party*), so have you heard of any other way to prevent Ebola or for you not to get Ebola is there any other one?

R2: “Yes, when they said to wash hands, we in Sierra Leone here we like this greeting that when we meet except we have to shake hands and begin to bind each other. So we are unable. That is why they came with this method that we should wash our hands, before we greet we have to wash our hands or after greeting we have to wash our hands”.

M: Ok, yes?

R3: “Because we too, what is our problem here is they do not send Chlorine here. Sometimes they only send soap here for us to wash our hands. When you come from the toilet you have to wash your hands. Those are the messages that we have”.

M: Yes, My own brother?

R4: “The one method which we understand about it is that only not to touch a person who is sick for a long and not to greet somebody who is a stranger who is coming from somewhere if you do so you have to wash your hands, you should not accept a stranger as the person reaches you have to carry the person to the Chief “.

M: So like, have you heard about any vaccine they are coming with to prevent Ebola? We know that ok, you have this polio marklate (=vaccine, vaccination), you get measles injection. So have you heard about any other injection which they say will be coming very soon to treat Ebola?

R5: “We have no idea about that yet”.

M: Ok, yes?

R6: “ I had raised up that point just now when they discussed that the marklate (=vaccin(ation)) will be available to everybody”.

M: Alright, ok. What are some of the common things that you discuss about Ebola, which you might have heard or taking place in your community? Do some men agree with those things?

R7: “When we meet at times we just talk about this sickness”.

M: Like what and what do you discuss?

R7: “When we sit and discuss we advise ourselves about this sickness that this sickness is real. People should not keep roaming like before, since we listen to radio sometimes. So the precaution which doctors have given we must abide by them. So those are the discussion we do. There are some time someone would say aay “Sam” (= man) this sickness is true because it is killing our people. So we believe that it is true, we do not near ourselves (= come close to each other)”.

M: Yes?

R 8: “There are times when we sit and talk about Ebola, some people take it that it is true, some men take it that it is a lie, they can be on that argument until. We are praying to God for Ebola to finish, that is our topic now”.

M: So, like what are some of the things that you talk about that made some men say I don’t believe and I do not agree this is not they said or this is what they said? What are some of the things that you talk about?

R1: “Well at times people try to talk about this body contact, some people say when you touch your companion the virus transfers, some will deny that “no, this not true, how can you just touch somebody and the virus transfers”. At times we can be on that topic for some time”.

M: Ok, yes?

R2: “Is the same thing just like what my brother said stubbornness we have in this country. If they say don’t touch and we should not gather so as we are now so close to each other, because when we are close if a person has the virus he can transfer it. Now a person can just lie down and die then they say it is Ebola. Most people used to deny that the sick is not real, that they want to play criminal so that the sick is not real because the symptoms which they showed us is not there again”.

M: Alright, yes?

R3: “Most where the argument comes from is that when they say you should not eat “beef” (= meat), some men when you say not to eat beef brings argument he says “how can an animal in the bush get Ebola?” You see the problem, so that one is not easy, these are some of the discussions we get so far. This is the concern I have right now, I summarize there”.

M: Yes brother?

R4: “ At times some men can find it difficult, you know though they mentioned all the different kinds of beef we should not eat some men think that is not right to say we should not eat beef what about the fish which has the same blood? We do make bitter argument about that”.

M: Yes brother

R5: “Like the survivors just like they have been saying on the radio that any person who survived the sickness should wait for three months before having sexual intercourse, some people do deny, so at that point at argument enters there”.

M: Ok, yes?

R6: “About that bush business when tell person he will tell you that beef (animals) did not die in the bush from Ebola. If I kill it for myself why should I not eat it, some of them can say so”.

M: Yes?

R7: “Like the bush meat, like goat you can meet a dead one, some say it is Ebola, some say it is not Ebola, they deny and they eat it, some can throw it away”.

M: Yes?

R8: “Ok, like the argument I just raised concerning this beef issue, they heard that before March the sick will end, then is government going to send people to inject all the animals in the bush? I don’t know, but, it can be difficult when they told us not to eat it they said there is where Ebola starts”.

R1: “Yes Sir”

M: Yes?

R1: “Eh most of the argument that we get here which comes up quickly is that some men are saying the Ebola, Ebola is going to end. I think they are going to inject all the animals in the bush. Then those bats we are seen in (- - Name of district headquarter town of the interview district - -) they say they are the carriers of the sick, but they leave all those thing there when that bat comes and “kaka” (= excrete) here then the “Kondo” (= Lizard) or fish eat it, it cause that problem even dog they said dog is a carrier of Ebola, monkey, mango through that you can get Ebola not so? Because they say bat eats mango but most of the animals also eat it, fowls, goats they eat it. Then fowl does not have Ebola, we eat fowl. All those things bring argument among us. Some men believe that there is Ebola some men do not believe that it is real”.

M: So, do you have anything that you would like people to know more? How do you feel to go about it to do it? Anything on Ebola which you would like people to understand more and how do you think they have to do it?

R2: “Is just advise, we advise ourselves, like how they are talking this virus you don’t have to touch your companion, you should not bind. When he had got the virus and has been cured and returned to the village he should refrain for three months without having “woman bisnes” (=sexual intercourse) but if he doesn’t adhere to that and have sex with his wife that is how the sickness spreads”.

M: So, how would you want, let’s say government sends you to go and talk to a person, how would you go about telling that person how to go about it?

R3: “Is just advice”.

M: That’s it, how do you go about advising the person?

R3: “Let him don’t touch”.

M: Let him not touch his woman for three months, how would you go and talk to that person that government has said this is what it, how can you go about it?

R3: “Is to talk to him”.

M: How would you talk to him?

R3: “Bel am” (= cajole, convince him)

M: How do you ‘bel am’ (cajole, convince him)?

R3: “You can cajole (convince) him by talking to him in a polite way, talk to him in an intelligent way, with respect for him not to do this”.

M: Yes?

R4: “Like for us now in this village no person is going to advise us too much again because the cases we have had in the town even if Ebola catch you or us who have suffered it know that it is there. Then the advice they try to give you for that particular person. Try to explain to him the symptoms of Ebola especially that touch and when you are with a person you must touch the person, that body contact all will come in so if you explain all that to the person if he realizes it then he has to avoid for that three months”.

M: Yes?

R5: “Ok, survivor, you have your woman at home who has just survived and come. If I am going to advise you will go there and “bayo-bayo” (=pamper) you, and tell you that I how you have been advised you when coming, and he says yes, what and what did they advise you and then he will tell you that they told him not to have “woman bisnes” (=sexual intercourse) until after three months (90) days. So please boys, now that you have listened to that do not mistake because we are getting the examples now, we listen through radio that there are people who sick by Ebola, some have died some have survived, so please I beg when you are back this of your woman do not touch her for three months, you have to bear whatever it is be patient or if you have another room leave her in her own room and you go to another side because although it is not easy to be with your woman lying behind you for three months it is not easy. But if you have your own separate room and the woman too has her own separate room you will interfere into your woman’s room”.

M: Yes?

R6: “Because, when you are going to advise somebody, because they have been advising us for long now they tell us but we do not believe. The only message which I feel we can use when going to advise a person is only when we put it into action like when we make concert, through television (= television or video show) you sit down and watch what is happening but to just to hear an information without seen you can believe and we cannot believe again because except we see now before we believe. So we have now turned to Thomas now when Jesus went and met him and said I am the man whom they liked and I have risen again he denied except when he appeared to him again before he believed. So that how we are now except we see with our eyes before we believe. We who are going to advise if it is the government that is giving us those thing we make it like a concert they will go and act if it is one town they act(=play drama, sketches) how the sickness affects, the damage, it destroys or they put on the television (=video) and play it around the suburb villages people will believe that the sick is real so that they can take the advise which they give them but without that I am sure if they will take it”.

M: Yes brother, so what do you think people need to know more about where do they need to know which they need to tell them that this is this is this about Ebola and how would you go about it to tell people for them to understand that thing that it is real.

R7: ‘Just like my brother said because there are some people, I have seen it to my brother I cannot deny again but there some [people who are still denying they do not take it to be true”.

M: So like how can you go and talk to that person for him to like stop denying?

R7: “If I meet him I can go and talk to him, we sit down I explain to him because how the Ebola is now everybody is seen the papers which they give to us which they paste on the wall so I will explain to him how the virus walks”.

M: Yes?

R8: “If I am going to advise a person the first thing I will do if he gives me his hands to greet I will not accept it, I will not even sit at his house so that he can know that there is death”.

M: Yes?

R1: “To me to advise a person if that person is doing bad, you have to befriend him first, be with him until you know how he walks you can know about him, so he can tell you what concerns him in life that is how you get him small, small”.

M: Yes brother?

R: “Like for me when I am going to advise the person, when I go first I will give him talk so while we are discussing I will bring the advise talk so by that he can listen to get more idea”.

M: Ok, well I want to tell you all thanks for taking part in this discussion and I pray that we will see each other again and this disease can leave us and we all will begin to go back to school.

Rs: “Isha Allha” (By the grace of God).

M: I thank you ya?

Rs: “Mmmm”.
