# Supplementary material for: Development of a set of community-informed Ebola messages for Sierra Leone
Source: PLoS Negl Trop Dis. 2017 Aug 7;11(8):e0005742. doi: 10.1371/journal.pntd.0005742 (PMC5560759; doi:10.1371/journal.pntd.0005742)
Supplement: S1 Appendix — (ZIP) [file pntd.0005742.s001.zip › Ebola messages - FGD and interview transcripts/R2HC Ebola Fieldwork 1/R2HC Ebola F1 FGD-MAYOU-Urban1 V3 CORR.doc]

| CODE | **R2HC Ebola F1 FGD-MAYOU-Urban1 V3 CORR (urban focus group discussion)**  **V2 – 10^th^ March 2015 – ADD PROBE**  **V3 – 11^th^ March 2015 – correction personal data participant** |
| --- | --- |
| DATE | February 2015 |
| DURATION (minutes) | 34 |
| Collector nr | 5 |
| LANGUAGE INTERVIEW | Krio |
| **TYPE FGD** | Male Young (<25 years) |

***PERSONAL DATA PARTICIPANTS***

| Nr | Sex  (*F/ M*) | Age  (*in years*) | Education Level (*e.g. none, Primary, secondary, tertiary*) | Language (*e.g. Mende, Temne, Krio)* | Religion | Job / Employment (*how they earn their living e.g. farmer, teacher, trader*) | Role in community  (*e.g. youth leader*)  ANONYMIZED, ONLY AREA OF ROLE INDICATED |
| --- | --- | --- | --- | --- | --- | --- | --- |
| 1 | M | 27 | Secondary | Krio | Christian | Electrician | None |
| 2 | M | 21 | Tertiary | Krio | Christian | Student | None |
| 3 | M | 21 | Secondary | Krio | Muslim | Student | None |
| 4 | M | 24 | Secondary | Krio | Muslim | Student | None |
| 5 | M | 24 | Tertiary | Krio | Christian | Student | None |
| 6 | M | 27 | Secondary | Mende | Muslim | Student | None |
| 7 | M | 24 | Secondary | Krio | Christian | Student | None |

**TRANSCRIPT: (M = Moderator, R= respondent, R1= first person responding to a question, DOES NOT correspond to numbering used in Personal Data!)**

M: Can you tell me how has Ebola affected your community? Or have seen a person affected by Ebola?

R1: “Before I go further, believe that (- - name of interview community - -) is one of the communities spotted out, one Ebola patient who has just left hospital, because like when we saw that she has the sign and symptoms we called 117; we even took her to hospital and they came and quarantined the place for 21 days but the problem that we have in the community is too great”.

M: So like what and what?

R1: “Well like the water facility. Because the way we used to get water and now is different. The way and manner in which we used to go to the water side is different now like if one person goes to get water until that person finishes before any other person go there to get. Then if you look at that for the day, only four or five people will be able to get water because like the community does not have enough tap and two, like the dirt in the community because at first we were going house to house telling people to give us financial help for us to clean up the community. But like now when we go door to door for that the feedback which they give us is not good. You have seen for yourself when you came maybe you cannot talk about it but you can see the dirt is different from any other community when you walk around. That is affect that Ebola has caused we suffer a lot in this community”.

M: Yes my brother?

R2: “Well I don’t have much to say but the main issue is that the impact that it has created is too great because we keep denying but we have seen people lost live because in the neighbourhood close to me two people died in the same house based on this Ebola. So just like what my brother is saying this Ebola, even there were some people who showed signs of Ebola but they have been on probation for three weeks and I think that they have since not shown further signs, so like the impact is very great just like what my brother is saying the problem of water is difficult, just like what brother said, there is also stigmatization people are afraid to go to peoples compound now because they think the person might have Ebola. So let me we straining in the community”.

M: Ok, So what do you think is responsible for the spread of Ebola in Sierra Leone?

R1: Like me to my own idea, like Ebola when they noticed it at first in Kailahun (= first district in Sierra Leone with Ebola cases) they could have quarantined the whole of Kailahun. They could have closed Kailahun nobody should enter and nobody should go out. But the government allowed, like trade, trade is one thing responsible for the spread of Ebola. Then secondly, there was no awareness, the sickness was something new to us the government was not aware of how to tackle it because like it has never appeared in our country. So like that is the way Ebola got spread. Then thirdly, here people deny too much, because even though they have seen the thing affecting people here but they are still denying, I am even one of them but when I have seen the way it has killed people I now have the believing that Ebola is real and then what the International Community is talking about it I now believe that it is real but like the Ebola that is in this country is different from the one which happened in 1976 in Congo, is far different because we have not seen Ebola people oozing blood from the nostrils and the eye, we have not seen any, the symptoms are different because Ebola has signs and symptoms of Malaria, typhoid and fever so you see? But I also noticed another thing like they were taking the estimated cases of people who died of normal sicknesses and adding it to Ebola”.

M: So you think that is one of the ways Ebola got spread in Sierra Leone?

R1: “Yes”.

M: Yes brother, I see the mouth shaking do you want to say something? Why do you think Ebola is spreading in Sierra Leone?

R2: “To my own common idea as to why Ebola has spread in Sierra Leone, some are saying it is denial and others say it is stubbornness. The professionals have said that when a person dies people should not touch or wash the body but by the way you can see people washing dead body and you cannot tell what killed the person, if it is Ebola that killed the person then automatically all those who washed the body have become infected. So that is one of the reasons why Ebola has spread in Sierra Leone’.

M: Ok, so do you have any local name to call Ebola?

R3: “Ebola Tae bukor”.

M: What do you mean by that?

R3: “It means ‘Ebola...you should not wash’”.

M: Some people still do not believe that Ebola is real, do you have people in this community who do not believe that Ebola is real and do you know why he is thinking so?

R4: “We have plenty of them in this community, like we have one thing here, there are some men who are SLPP (= main opposition party) and some men who are APC (= government party). Some men are looking at it that the first outbreak in Kailahun (=first district with Ebola cases in Sierra Leone) the first 80 or 90 million Dollars or Pounds which they sent they are looking at it that is a free landscape think that they can just eat or do anything with it that gave cause to the sick to spread because like the money was too big for the Ministers to handle. Then the first thing is we have people who look at it in that aspect, that SLPP / APC aspect, then the money which the government have received for Ebola and up till now the sick as not ended so people are taking that it is not proper’.

M: Ok, yes my brother?

R5: “Just like what my brother has said a lot of people are looking at it as a political thing, saying like it is APC / SLPP something. But as it now, the thing is damn too real so let us put everything aside, there are people who are still denying and if they continue that this thing will not end. I just take an example from the HIV, HIV has killed millions of people and even now some people hold the denial that Ebola is real. although you cannot coarse somebody to believe that the thing is real and like with the sensitization that has gone on in the country is too great so I know that denial is like you can’t change somebody’s mind, opinions everybody has right to his or her opinion. But like I said people wanted to take it that political way, but I know that Ebola is real”.

M: Ok, so have you heard the messages that they do give on the radio, TV, Newspaper, can you give me some of the examples of those Ebola messages?

R6: “One, they said when somebody dies nobody should wash it, two you should not go to burial when you do not know what killed the person, then three when a person is sick tell the person to go to hospital and then four whenever you see a person when you do not know where he or she is coming from you have to let him or her go to the Chief if you have Chief in the area or you try to ask his or her main purpose of visit”.

M: Yes?

R7: “There is one message which they use to give when they said nobody should touch his or her companion but some of us in this community we are very stubborn not only this community but the whole country because they said don’t touch your companion then two, they said when a person is sick do not go closer to him or her, if you tell the person to go to hospital and he or she refuses just call 117. So that is the talk”.

M: Ok, what are some of the Ebola messages you have been hearing, you have been reading? Can you give me some examples?

R8: “They said when a person dies you should not wash the person you should call 117 but we have some stubborn people here when a person dies they wash it first before they call 117 that is why the sick is spreading”.

M: Ok, so like if the government come and meet you and tell you that some people are still keeping sick people at home, and they said let you go and talk to those people to go to hospital, how would you talk to that person?

R1: “Well is not too easy. Like because of tradition because some people are coming from the provinces they always have it at the back of their mind that they should be closer to their people when they are sick so when going to talk to them you should put yourself in their position, you should encourage them to do that so that they can cooperate with you”.

M: Yes, I see, my man want to say something?

R2: “Some people still have that stereotype way from their villages or the provinces, some men are still holding on to their traditional beliefs. If you’re loved one, or somebody close to you dies. If a man like me is to go and talk to them, just like my friend was saying you need to put yourself into their position, persuade the person to see the need to go to hospital”.

M: Yes brother, if government send you to go and talk to go and talk to a person at home, how can you talk to that person?

R3: “Like my own idea is just like what my own brother said you have to go politely and deliver what you have been asked to do in a way by encouraging them and tell them that the one who is the head has said that when somebody is sick you take him or her to hospital, you should not go close to the person, you should encourage him or her but you should not be that close to the person and to report to the nearest police station to carry the person for a better treatment”.

M: So like we have channel we use to send messages which is the radio, television and newspaper, postal, so like which one do you think is the best way to carry these messages to the people behind the villages of up the mountain to get the message?

R4: “Well like for me I will suggest that like we have a radio station here which is very close to us and I see that if anything happens you have to go there first and report what is happening in the environment so that they can know what is going on for them to know about that. Then we also have “Wae Yus” (name of a TV program), you can go and tell them what is happening in the environment which you have suggested that it should go on which you do not like. Those are the two things which I suggest”.

M: Yes?

R5: “Well to me the house to house sensitization is more effective because it is not everybody has the facility to watch television or listen to radio but if you go house to house and sensitizes people telling them that this sick is real and that when somebody is sick they should not wash the person you might not know the cause of sickness of the person. If the person has Malaria let the person go to the hospital for Malaria treatment, if the person has Ebola let the person go to hospital so the professional can cure the person but I think the house to house sensitization is the best”.

M: Yes?

R6: “Well through my own idea, the poster is one, because the thing which each and every Sierra Leonean has at the back of the???? is that what they see is what they believe. Sierra Leonean does not believe in radio even when you are, there is no difference between it and the film. You should have the poster give it to them as you go house to house and tell them that when a person has Ebola this is what you should do. That can make the individual to be afraid, then two when Ebola came it has destroyed a lot people, because if can take you back during the war, while the war was raging on in the provinces when you tell people in ( - - name of headquarter town of the interview district - - ) they will ask you if a rebel is a human being, a monkey or a baboon? So if the Ebola has come to ( - - name of headquarter town of the interview district - - ) people should try to believe, there must be one person to be a true case so people should believe; government should have let the people believe. If you did not believe that this sick is real because ever body has it in mind that what you see is what you believe and that was what caused the spread of the whole thing, the Ebola. So I believe that the door to door sensitization is necessary than the radio and television programs”.

M: Ok, supposed somebody has Ebola now, where do you think the person would like to go first? Will the person go to a ‘moray man’ (Traditional herbalist) or the hospital? Would the person go to the hospital that has been here or the ones they have newly built?

R7: “Well this question is a two-fold question you have asked. People have the perception that normally they can get fever, normally people can get head ache so they can take it that if their temperature warms up especially those coming from the provinces they will have the mind to go to ‘meresin man’ (=traditional healer). So as of now when the thing is a national awareness I think people will go to the CCCs (= Community Care Centres)”.

M: Ok, yes?

R8: “As for me, there are people who gets fever and decide to ‘pepe doctor’ (= cure themselves without doctor’s advice) for two or three days. Based on what professionals are telling us if Ebola takes one minute in your system it produces one thousand of its kind. So you think about it if it takes three days in your system you will die, so that is another thing that makes people die through this Ebola. So if you start feeling the sign and symptoms do not ‘pepe Doctor’ yourself go straight to the hospital or the facility Centres”.

M: Like when you sit together, what are the good things and bad things that you hear people talk about the Ambulance Service?

R1: “Well like what I have heard about them, they said time there was when the government was not paying them they used to take dead bodies and leave them in the street, some of them even when they call them to come and respond quickly because of this Ebola they will say no because the government has not paid them and that they are not responsible for that and they will not go there to take care of that person. So that is what I have been hearing”.

M: Yes?

R2: “The other understanding I have about them is that in Lunsar, when they call 117 to come and take the body they said the first treatment which they give to them the treatment is not proper then if they take them to the Centre the injection which they give to them is what that kills them”.

M: What do you hear about those Ambulance drivers?

R3: “One, like for the drivers, the bad side of them is that they are not enough then two, they do not respond to time, three when they come they have protocols before taking the body. That is the bad side of them”.

M: Yes?

R4: “Well, the bad side of them is that where we are now is a hill side people die up there and when the Ambulance comes they park here and there is no track for motor car to go up there, so is like they always have doubt to go and take that person because they take that they are going to climb a hill. So that is another stigma, on that side it is a stigma to go and take a person up that hill”.

M: Yes?

R5: “Well the one I want to talk about is about those Ambulance drivers. Some of them drive very badly in the street because they are driving Ebola Motor Car so that is why they drive bad ion the street. Look, the last time someone died in the street here in (- - name of interview community - -) it was lying there for over how many hours before they come to take it”.

M: What about the Holding Centres, the Treatment Centre and the CCCs (Community Care Centre), is there and bad or good thing that you hear about them?

R6: “I see some people hold Ebola patients with their two hands, go tell them thanks because they risking their lives to save people. They really know that this thing kills but they still risk curing them. So I give them credit for that”.

M: So what about the Burial Team, what are the good things and bad things you have been hearing about them?

R7: “Actually the cause which they take to bury is serious because if a person is affected by Ebola and then you go to bury that person it is likely that you can get it because Ebola is not a child’s play that is why they are paying them huge money, is just that I am getting it by rumours because is like the men are trying because taking the body and burying it is a great challenge so they are trying”.

M: Yes, Yes Sir, the Burial Team which good or bad thing do you hear about them?

R8: “The bad thing I hear about them thing which I know about them is that they take bribe. Like if Ebola worker come here and the person bribes him, he will not take the person he will leave him there and go. Then two, the one which I suggested even though “Wea Yu” (TV program) show it, there comes a time was they buried somebody and then after they have buried they did not undress there and leave the used PPE on the streets. It happened in (name of a section of Freetown) and Wae Yus showed it, they left the PPEs everywhere around the field and children go there to play and that is not nice. They have told you that this thing is bad, an Ebola worker when you finish burying you have to leave the PPEs there you have to put it where somebody cannot even go near to it. But they went and put it somewhere exposed where children can go and play with it and those flies even the water when they wash their hands with in the street if a fly touches that water and come back and touch you must have the sick once that fly went and touched the water of the dead person they washed in the street so that is the bad thing which I think they are doing”.

M: Yes brother?

R1: “I don’t have much to say because the people are doing well because the thing they are doing they too are risking their own lives, to carry and bury Ebola patients and people who have died of Ebola is a risk. They are doing well actually, that is all I have to say”.

M: Ok, What about the 117 Line what are some if the good things and bad things that you hear about them?

R2: “Like some of the bad things which they were doing, they were giving comment that people were mocking them, they can call the team and tell them that there was an Ebola patient somewhere and when they go there to check is a lie. Some people are doing those things and that was not right. They put it that if that is how it is happening when they call them they will not respond. It happened again in (- - name of interview community - -) at one time somebody called but when they came the person was not an Ebola patient. It has happened not one time, not two times people cal call 117 and say this local, this location, they tell lie to the people. So there was a time when they put a stop to that. So that was what they were doing that is bad”.

M: Yes, what are some of the good things and bad things you have been hearing about the 117 Line??

R3: “117, one good thing I know about them is when you call them they respond. Then the bad thing is, just like my brother said is the ??morkry?? of the land, one time a woman called that she was infected by Ebola when they came for her she ran away, what is all that for? My brother the needs to bite the people who do those kind of things so that it won’t be repeated”

M: So what about the hospitals that has been here and the people who work there, what are some the good things and bad things you hear about them when trying to treat somebody who has Ebola?

R4: “The bad thing which we hear about them is that even concerning pregnant women, if I can remember some months back when Ebola was rampant here in (- - name of headquarter town of the interview district - -) when some pregnant women go to hospital they won’t get somebody to respond to them and that I think is not fear. I even see a day before yesterday I saw at the government hospital like Connaught you have the negative and the positive, like one Doctor to respond to a two hear old child I don’t whether the child was an Ebola patient or not, but he was not patient with the person he even shouted at the person and he too has his own ??chuid?? so people even gave comment about that say ay what you have done is not right if it were your own baby you will not do that and that he should not have done that. So he told them to go to some other place. They postponed the girl but like the child was in pain and the other one which I saw this morning was that a guy went they do not know whether he was an Ebola patient or nor they took care of him and talk to him. Like he was not getting frequent stool, he has stomach ache, his body gets warm he even said his joints were aching but like his eyes were pale and sunk in but like they way I see it the Doctor who was around took care of him fine. So that was the positive thing that I saw on that day”.

M: Yes?

R5: “Well like I am talking on negativity because some of them the way they behave to people like a person shows sign and you got panic and call 117 and they come and take him to the Treatment Centre which is a nice approach but secondly now the main thing is that the stigma now, the fear of the Health Worker that the person has Ebola makes him treat the person as the person has no life. When that is happening like when a person comes with a sheet problem do it for the person when you want to give him or her something give it but you keep a distant from him or her you will killing the person you know, that kill people so they have to adjust that side”.

M: Ok, so like in this community how do people react to Ebola survivors? Have you got anybody who has survived Ebola?

R6: “Yes, they just came with them yesterday, two of them her and her daughter but actually we have not gone closer to them yet but down there they held them fine some people welcomed them sit near to them try to talk to them for other people not to be afraid ”

R7: “When they came yesterday because I was at the scene when they came some people came out of their home to come and hug them, they talk to them just like they were before because here we have heard that anybody who has survived Ebola can neither infect nor can someone infect him or her again that they were heroes they cannot transfer the disease again so people had the awareness yesterday. People went and hug them talk to them fine even the officers who were there said they talked to people fine”.

M: Yes?

R8: “Well I cannot say but they are survivors because there were people in the compound who had Ebola so they too were under threat that they too had Ebola though people in the neighbourhood were afraid of them at first but now things are going on well. Three weeks back the people were under quarantine but that Has been lifted so is like”....(*not clear*)

M: So like which message would you like to send to like other communities who do react badly to those Ebola survivors?

R1: “Well as for me the message that I want to send to people out there is that they should know that those people who survived from the Ebola are our hero we should hold them like we used to do before, we should not push far away from them, nobody should provoke them , the “smae GHaod” made all of us, we are all the same human being”.

M: Ok, yes?

R2: “Well as for me my word is that those people should they should talk to them fine and hold them like they used to be before. Then secondly my own approach is that people who survived Ebola, government and medical people are saying that when you survive Ebola you have to avoid sexual intercourse for three months so is like people in the community should know that and should not go towards him or her with sex they should talk him...”.

M: Yes?

R3: “As for me, I want to go contrary to what they are saying. We have seen another ??wifery somethong?? about Ebola which I feel has to be tackle first if we do not tackle it faster it will be ....”

M: What do you mean?

R3: “That is why I said I am going contrary, there was a time when an Ebola survivor forcefully gets sex with a woman who transferred the sick to the lady and the person died. So like what I am advising government to do is that the Ebola survivors should not be allowed to come back into the community again, they should find a confinement where they can be for them to take that three month duration which they have given them after that three months they can allow them to come back, that I am thinking about the males , of even both sexes”.

M: Yes Sir?

R4: “Urr, just like what my brother said, like somebody who has survived from Ebola just there is no need for you to get panic towards the person, is a sick if it affect that person and it doesn’t affect you does not mean that the person is a devil or an extraordinary somebody, is just that he or she happens to get it. If that person survives never mind he or she has the stigmatization that the person has had Ebola but survived, you have few people who would believe and others would not believe in that but like if they have said the person has survived and returned to the environment I think you have to encourage the person talk to him or her fine for him or her not to take to mind. Because it can happen, you can have somebody who survived talking about that person is a back biting, at least when you begin to talk bad about the person he or she will not feel good he or she will feel bad about that and when he or she thinks about it the more he emaciates but if you ate talking bad about him or her and you go close to him or her, his or her body will come up fine like how it was. So that is the thing I know”.

M: Ok, have you heard about any new treatment that is coming soon for Ebola?

R5: “Well I am not yet aware of that because I only know about the first Ship of vaccine, an experimental vaccine in Liberia and not for Sierra Leone. But I am hoping that after using it in Liberia they will send it here, the vaccine”.

M: Ok yes?

R6: “The fact that Liberia has got a quota maybe they are going to send some to Sierra Leone”.

M: So like have you heard about any new way to prevent Ebola apart from the ones you have been hearing?

R7: “Well I cannot deny that fact, our people in Sierra Leone, our mind set is based on myth, different stories; the “Bitter Cola stuff” (*note: Bitter Cola - officially “Garcinia Kola” is a tree of which the nuts are chewed /used for medicinal/stimulating purposes*) all this is rubbish because where there was Bitter is people are still dying so is like it is not necessary to bring those kinds of talks. So I believe that, that is one point why people were not going to the hospital because there is no cure for the disease, it is a comic business how can that be? People are just using their philosophy but Ebola is not real”.

M: So like when you are sitting at the junction in the evening what are some of the thing that you argue or discuss about Ebola?

R8: “Like at first were saying that it is an experimental disease yes that can be too but on the other hand we cannot tell but they said it is a virus and that is another part which brings controversy because virus has no cure but that the question is, is it natural? It is not because natural disease always has a cure”.

R1: “We were hearing that the thing is a Lab business and it is not but some people were raising the question that why can how government know when this sick will come to an end when it is a natural something, you can never know when it is going to finish but when the government say the sickness can finish so, so, so time that means they know about it that it is we ourselves who made it and that it not a nature from God. Those are the common things I used to get”.

M: Ok, so is there anything about Ebola that you would like to understand more?

R2: “Yes, one of the stigma and problems is that when the sick came at first in mid May last year is that the sensitization was so poor that to an extent that people did not take the sick with seriousness because let me say for instance will didn’t know the types of Ebola. The people know that the sensitization was when you are affected blood will come from your nose and your ears then people are dying not as a result of that. I just want to know whether there are different types of Ebola or is just affecting us based on the local sicknesses that we have

M: So like you have now got the message, how can you take this message you have understood to other people, how can you do it?

R3: “Dissemination of information in the community base on the way you used to wrap. So like if we get good rapport between you and your people we will be able to know that you are going to tell us something serious. If you have seen the intensity in me that I want to tell you something serious you will always welcome me. So is like this thing is a serious thing now people should be serious it is a nationwide campaign now, so if I go to tell you is a great privilege is because I like you to bring that kind of information to you”.

M: Ok, I want to thank you for giving me to talk to you, I thank you.

**ADDITIONAL PART OF INTERVIEW, OBTAINED BY COLLECTOR 5 AFTER CONSENT IN PERSON with two (2) of the original participants, March 2015:**

M: In our last discussions, someone mentioned the shortage of water in the community, so I want to if this shortage of water happened before the outbreak of Ebola or during the Ebola outbreak?

R1: “Well the problem of water shortage has been before the Ebola outbreak but within the Ebola outbreak, it has become more difficult, because schools are closed as at now, and most of the school pupils are sent by their parents to fetch water, so the pressure and tension for fetching the water increased at pump or water site, before the Ebola outbreak we were having fifty people at the water site to fetch water but now in the Ebola crisis the population of people to fetch water increases from forty to hundred and fifty people at the water site, so for a person to fetch water at the water well or pump, you will take a whole day”.(*noise at background*)

M: “Another person raised up a concern towards the daily estimated Ebola result updates, that the number of this estimated cases is being added up, I want to know, who are they referring to that adds the number”.

R1: “Well birth and death I knew, they are in charge of deaths and birth cases, so if they had got the statistics, when there is no Ebola sickness, if they should decide that we are going to give death certificates at this particular amount but now when the Ebola has broken out, now if a person had died of Ebola or not, births and deaths department just add up the number to a particular figure”.

M: You said again people do not believe that Ebola is real, and they had denial and they are stubborn, so why do you think they are stubborn and the denial that this Ebola sickness does not exist?

R1: “Majority of the people, the community people, the people of this country, and you know everybody is entitled to his or her opinion, so people were denying that the sickness does not exist, but even if there was the sickness, it should have come to a standstill now but it is just continuing, imagine they are paying somebody five hundred thousand Leones per week (= about 113 USD 10mar2015) if the person is working at the Ebola centre or the Ebola office, some of these people do not want the Ebola to end again, because of the amount of salary they are receiving per week. So that is reason. When people are saying the Ebola sickness has come to end but people are adding up the numbers for them to continuing eating money”.

M: You said again, that people washed dead bodies before they call 117, can you tell me more about or the have you seen or know the person community that have done this?

R1: “We don’t know anybody that had done that”.

M: Ok, but who were you referring to?

R1: “Well, what we heard on the media, is what we talk about”

R2: “Apart from the media, it happens in (- - name of another district - -_) , wherein they buried the chief and child unknown to 117, the time they called 117 the people had already washed the body, they told the burial team that let them allow them for 30 minutes, so within duration, the people end up burying the corpse behind the compound”.

M: This was done secretly or publicly?

R1: “It was done publicly, because they washed the body before they called the burial team, so when the burial team came they told them to handover the dead body, it happened in the (- - name of another district- -), they told the burial team to give them 30 minutes to do what they feel, not knowing that the 30 minutes they asked, was to go and burial the dead body, among the people were doctors, when this thing happened”.

M: Have ever you heard or seen anybody in this community that have done secret burial?

R1: “Since the outbreak of this Ebola, if I could remember, in this community we have only registered three positive cases, the cases are not too much in this community, and so we don’t had secret burial issues in this community” (*noise background*)

M: Somebody mentioned the word “taaboku”, what language is it, and what is the meaning?

R1: “It is a Temne word meaning, someone that do not bath “taaboku”.

M: Another person mentioned again that, they feel that they will just come and eat, who are they referring to and what is meaning?

R1: “They just want to come and eat”?

M: Yes, I don’t know who are the person was referring to? (*Sound of dishes*)

R1: “Well, Ebola is a thing that comes and eat then go, if you can closely watch the Ebola sick, it has killed a plenty people, it will just disappear, so that is meaning, Ebola will kill the people and go”

M: You said again that, this thing is APC and SLPP something, what is the meaning of that?

R1: “Sierra Leone, everything about this country, has been politicised”.

M: Who did the politicising?

R1: “The government had politicised everything, we knew in this country, we only have two political party that compete greatly, SLPP and APC, if we had got sickness like this, a Sierra Leone Peoples party (SLPP) man we say the sick is brought by the All Peoples Congress party (APC), so they had took it that political level, so they are taking it”. (*A mobile phone ringing*)

M:There were people who do not believe Ebola exists and something happened that changed their perception from to not believe to believe, what is the thing that happened which leads to change in perceptions?

R1: “Like any sickness or anything in life, if a person does not see with his or her eyes, they will not believe, when they were talking about Ebola from Kailahun (=first district in Sierra Leone with Ebola cases), they do not believe but if something does not come to your dwelling, you will not know the importance, when Ebola came in some of the people’s households, and we the community saw it around and how the sickness has killed one or two people, from that time, our perceptions have changed and believed now that this sickness exists”.

M: Can please give me the exact date your registered the first Ebola confirmed case in this community?

R1: “The first case we got, it was up there, the person went to “upline” (= the rural areas of the country, upcountry), the person went on a burial ceremony, when the person returned, the person was infected with Ebola virus, it was last year November, during the time, when the cases of Ebola was in exuberant, it was from the 10^th^ to the 15^th^ November, when Ebola was wide, it is around that time duration”.

M: Ok, another person made a statement that, the stereotype way of the villages, what do you mean by this?

R1: “Stereotype way was talking about Belief”.

R2: “What did the man said?

M: Stereotype way from their villages?

R1: “But the person did not talk about anything first before he mentioned the stereotype, someone should have used a word, before someone used stereotyping”?

M: He said some people will be closely to their people when they die, that stereotype?

R1: “Stereotype is talking about belief, When someone was your friend before Ebola outbreak, so in some communities or villages, they had their own stereotype beliefs, they believe that when someone died, in terms of sadness or happiness, they have to carry out all ceremonies but if now we had got this Ebola breakout and they want to stop them doing all these ceremonies that they were doing, it will be difficult for them to change”.

M: When I asked the last time, you said some people from the provinces have mind or zeal to go to traditional healers?

R1: “Yes we were talking of the witch-gun”.

M: Ok?

R1: “More in Africa, we had a lots of magical things that happened, I will say witchcraft, witch-gun, and all the sorts, all these things when someone comes across them, and they had similar symptoms to Ebola, so if someone had got these things and had gone to the hospital but they were not able to cure, they will go to the spiritual healers, when you are there with these symptoms what do you expect”?

M: What do you think about these set of people going to the traditional healers and the others going to the hospital?

R1: “Because some people from the community they heal from, they believe that anything happened to them they should go to the traditional healers, as we say in our local language “Morayman” (=sorcerer) but if you were born in Freetown, we may not think that when you feel sick, you should go to the traditional, you should go straight away and seek medicine assistance first before you go to any place”.

M: Do you have a person that had got this signs and symptoms and had gone to the traditional healer in this community?

R1: “No, we had not got that person yet”.

M: Another person said, some people prefer to stay at home when they think, they may fall sick and continue “pepper doctoring” (illegal / unqualified treatment) themselves at home, why they are doing this?

R1: “Because they were afraid”.

M: Who are the people that are doing the “pepper doctoring” (illegal / unqualified treatment)?

R1: “I am talking about the whole country, some people are not going to hospital again, like me, I will take myself as an example, if am sick right now, I will not go to the hospital, I will prefer going to the nearest clinic or the clinic that is around then take medicine, I will not go to the hospital, because some people are thinking when they go to the centre, they assert Ebola on him or her, wherein the person does not have Ebola, they will take the person as an Ebola positive, which is not”.

M: You were talking of injections they were administering to people, that lead to their death, who are the people administering this injection, and why are the administering the injection and what is the rationale behind the injection?

R1: “At first when this Ebola thing started, sixty percent (60%) of the people that went to the hospital died, so people were having the philosophy that the injection, treatment that given to them to cure Ebola was not pure, so they ran away from the injections, claiming that the injections kill”.

M: Ok, but can you tell me exactly the person(s) or people that gave the injections?

R1: “Well the people that were controlling the Ebola issue, at that time the national Ebola response committee (NERC) was not formed, so at first it was the government, if you mentioned that sickness does not cure but where do you got an injection to resuscitate that person for that moment, is a injections that kills”.

M: But has the idea about the injection changed now?

R1: “Yes, because we had international help, people from abroad had come, and the spread of this Ebola thing has become wide, we had aware that the sickness is real, because we have seen the Ebola had affected other countries, we have seen the things about Ebola they showed on television, and the other sensitizations but towards the end of the this Ebola now, we had knew, how to break the chain of transmission”.

M: Well, you said again when the burial teams come to collect dead bodies, they had a protocol, I want to know, what is the protocol that they perform before they take the body along, and what is bad about the protocol?

R1: “They will come first with a motor bike, and later the ambulance, they will stand together for some time and later come and spray, these are all protocols, they sprayed and sprayed before the sprayed, they will put on their gears, the person will be lied down sick, after, they will go and pick up the person, and even inside the ambulance the suffocation of the chlorine is too much in the ambulance, it suffocates even the patient”.

M: Ok, the spraying is the bad thing about the ambulance?

R1: “Yes”.

M: Someone mentioned that before, the burial teams were taking bribes, I want you to tell me, why they took the bribe, and who is taking the bribe, if you have examples of this happening you can give them and how are you comparing it from that time when Ebola started and to now, are they still taking bribe or not?

R1: “Well I will not say they have stopped taking bribe or not, because I am a victim, my grandmother died at the age of ninety-five years, all this time she was at home, she died at (- - another area in the interview district - -), they said she died of Ebola, we were not having problem with that, they called the swab team. They came and do the swab, because she was a Muslim we do not want her body to stay longer, so one of the member of the team said, if we want her test results within one to two days, we should give five hundred thousand Leones (= about 113 USD, 10mar2015) so they will speed up the result, they took the swab, so the next twenty-four hours, or so ever, why should they demand for five hundred thousand Leones, for them to result bring the result quickly, so that is all bribing”.

M: Are they still taking the bribe or they had changed from that?

R: “Yes, they had changed from that, because other stakeholders have involved in the Ebola response now, and all of us are aware that they are paid for the job they are doing, so there is no need for us to give them money, now we have machines that do the swab and test samples faster, like in fifteen minutes or so I heard”.

M: You also said people mockery of the 117 line, what do you mean and why were they doing that?

R1: “At first they feel it is joke sickness that we had, somebody will just take a phone and say, let me come and call and disturbed these idlers, because they said this Ebola business is about fund, it happened with a woman in our community, she called the 117 line , when they came, she hide”.

M: She called that, she had Ebola?

R1: “She called and tell them that, she had Ebola and she is living at a particular address, I had forgot the address, so the Ebola team came and asked for her name, and within that process, she hides”.

M: People at first were afraid of the Ebola survivors, I want to know, if they are still afraid of them or it has changed?

R1: “At first they do not know how this Ebola sickness transfers but now we are aware, the Ebola survivors from the treatment centres should keep to the ninety days without sexual intercourse, some may want to have sex, that was creating the problem, but now we are aware of it, we know now that even if you touched the survivors, the Ebola sickness will not transfer to you, they have cured, even before discharging them, they will give each Ebola survivor certificate, and the medical people will have a hand shake with Ebola survivors, because it is not easy to see people shaking the hands of the Ebola survivors, especially when they come into the community, there is stigmatization, people stigmatise them, at first not everybody will be around you”.

M: Ok, if you were an Ebola, what are the problems you will be faced with in the community?

R1: “I will face with a lots of problems, my girl lovers may not love me for some time and my friends will provoke me, they will call me all sorts of names, these are problems and myself will not go out, I will be ashamed, fearing of provocations and stigmatization, these are the problems, I will face”.

M: How are other communities treating Ebola survivors?

R1: “I do not know much about Ebola survivors that are around, but (- - name of community- -) at first when the Ebola broke out, it kills a lot of people and some people survived it in (- -name of community - -), I have enquired and asked people, they are still there, and the other youths are sensitizing the people about a proper treatment of Ebola survivors”

M: Is the situation of Ebola survivors in that community, the same to your community or it is different?

R1: “It is the same, because our own people that went to the treatment centre and survived, the girl child played with the boys who are survivors, they walked together, they had friends, we had a case down here, but they are still mingling with the survivors, they have adapted and joined us”.

M: Someone mentioned about bitter kola, what do that mean?

R1: “At first when the sickness broke out, some people feel that when they drank something that is warm, it will kill the Ebola germs, so they thought, that was why people were taking bitter kola, always smoking “jamba” (= marijuana), but when they were aware that, this is not a solution, but when drinking and smoking is a habit to people, whether there is Ebola or not Ebola, this is their usual habit, they are still doing it, though not for the prevention of Ebola”.

M: Ok. Thank you very much
