# Supplementary material for: Development of a set of community-informed Ebola messages for Sierra Leone
Source: PLoS Negl Trop Dis. 2017 Aug 7;11(8):e0005742. doi: 10.1371/journal.pntd.0005742 (PMC5560759; doi:10.1371/journal.pntd.0005742)
Supplement: S1 Appendix — (ZIP) [file pntd.0005742.s001.zip › Ebola messages - FGD and interview transcripts/R2HC Ebola Fieldwork 2/R2HC Ebola F2 FGD-Female-Rural1-B.doc]

| CODE | **R2HC Ebola F2 FGD-Female-Rural1-B (**Rural focus group discussion with younger (<25 years) and older (25+) females, using **topic guide GroupB and Picture set B)** |
| --- | --- |
| DATE | March 2015 |
| DURATION (minutes) | 120 |
| Collector nrs | 2 and 5 |
| LANGUAGE INTERVIEW | Krio |

***PERSONAL DATA PARTICIPANTS***

| Nr | Sex  (*F/ M*) | Age  (*in years*) | Education Level (*e.g. none, Primary, secondary, tertiary*) | Language (*e.g. Mende, Temne, Krio)* | Religion | Job / Employment (*how they earn their living e.g. farmer, teacher, trader*) | Role in community  (*e.g. youth leader*)  ANONYMIZED, ONLY AREA OF ROLE INDICATED |
| --- | --- | --- | --- | --- | --- | --- | --- |
| 1 | F | 56 | Tertiary | Temne | Christian | Teacher | Women |
| 2 | F | 35 | Primary | Temne | Muslim | Farmer | None |
| 3 | F | 35 | Primary | Temne | Christian | Trader | None |
| 4 | F | 35 | Tertiary | Temne | Christian | Teacher | None |
| 5 | F | 19 | None | Temne | Muslim | Farmer | None |
| 6 | F | 19 | None | Temne | Christian | Trader | None |
| 7 | F | 18 | None | Temne | Muslim | Trader | None |
| 8 | F | 20 | Secondary | Temne | Muslim | Student | None |

**TRANSCRIPT: (M = Moderator, R= respondent, R1= first person responding to a question, DOES NOT correspond to numbering used in Personal Data!)**

**(NOTE: Topic 10/11 – Burial – Poster - ”Safe berrin na free – e no right fo gi or tek moni fo am”)**

M: We are trying to create some message, about Ebola, and we want to get your own view about this message, the first message reads “Safe burial is free, it is right to take or give money” Yes my what do you think about this message?

R1: “They are trying to tell us that when someone dies, it free of cost to give that person safe and dignified burial, we do not need to give money to the burial team”.

M: Yes mama what is your understanding about this message?

R2: “Yes the same, when someone dies, we do not need to give money to the burial teams”.

M: Yes my sister?

R3: “It is just the same, when some dies, they should take money from the relatives of that person,

R4: “When someone dies, they should give neither take money for safe burial”.

M: Ok?

R5: “The same, the burial teams, should not take money from the bereaved family for safe burial”.

M: Ok, yes mama?

R5: “Is just the same message, when someone dies, should not take money from the family of the dead corpse for safe burial”.

M: Ok, yes mama?

R6: “We should not give money for safe burial, it is free”.

R7: “We should give money to the burial teams”.

M: Ok?

R8: “Well as I have seen that picture, it appears to me that is a method of burial that they are coming to train us about.

M: What people may not like about this message?

R1: “The dress of the burial team?

M: Why?

R1: “We like the free of cost for safe burial”.

M: What is wrong about the dress of the burial team that you don’t like?

R1:”Dresses of the burial teams are scary”.

M: Ok, yes my sister, what do you think people may not like about this message?

R2: “Like what that woman just said, some are afraid of the dresses of the burial team”.

M: why are they afraid?

R2: “Because they are not used to it”.

M: Yes my sister, what do people may not like about this message?

R3: “Some are afraid of the burial team, when they come, they will spray the dead with chlorine”

M: Ok, but this message is saying, safe burial is free, you do not need to give or take money, so what will people don’t like about the message?

R4: “They just afraid of the sprayed and the dresses of the burial teams”.

M: Ok my sister, what do you think?

R5: “Like how the others have said, they are afraid of the ambulance and the dresses of the burial team?

M: ok, yes my sister?

R6: “Is just the same”.

M: What is the same?

R6: “When the burial teams come, they dress in white, they become fearful”.

M: Ok, what do you think Ma?

R7: “May God help us all, the habits of the burial team is very bad, because the way come to collect dead bodies, it does not appear like they are coming to bury a human being”.

M: How do they come?

R7: “They wear white cloths overall, and they come unfriendly and very harsh, and they spray chlorine overall, but some people are also stubborn they still wash dead bodies, and when you wash dead bodies, it is easy to detect, because the dead body smiles, but thank God, because of the bye-laws, some people have reduce it ”.

M: Yes my sister?

R8: “one the ambulance and two, all kits that the burial team used”.

M: As you have seen this message, do you think the people in this community will accept it?

R1: “They just have to accept”.

M: Why?

R1: “Because, it is stated in bye-laws that, nobody should bury, rather the burial team, if you do, you are going to pay five hundred thousand Leones or you go to jail”.

M: Yes mama, do you think people may accept this message?

R2: “They will not accept?

M: why?

R2: “Because the burial members protect themselves, and do not protect the people, that is why, we have to enforce the bye-laws that you accept or do not accept, the law will take their effects”.

M: Yes my sister?

R3: “They will accept, because government has implemented their bye-laws, you like it or not, you must accept”.

M: ok my sister?

R4: “it is just the same you like it or not, you must accept it”.

M: Yes Ma?

R5: “when the Ebola outbreak, they were paying the burial teams for safe and respectful burial, so this is the reason they have developed this message, people will accept the message”.

M: Ok?

R6: “Unless the people accept, because you accept or not the president as already put in place the bye-laws, for safe burial and bribing”.

M: Yes mama?

R7: They will must accept, the reason is if you are rich or not, they will; bury with the body bags, so no one will deny this message”.

M: Ok, this message is saying we should give burial team, for safe burial, it is free of cost, the people will understand better or is there is any better way we should put it?

R1: “Well the people is having X on the man trying to pay money, for me I will understand, but for other people that do not understand, they may be thinking that man is giving money to the burial team, and also the arrow is showing that they are giving money”.

M: How do you want it to happen?

R1: “They should write no instead of X at the point where the man is giving money or don’t received”.

M: ok, so they will understand that better?

R1: “Yes”.

M: Ok my sister?

R2: “It is just the same, no don’t received should be written there, instead of X”.

M: Ok, yes my sister?

R3: “Yes, is just the same as my sister have just said”.

M: Yes mama, what do you think?

R4: “yes”.

M: What is yes?

R4: “Just like what woman have said, they should remove the X and put no don’t receive, the message is good, but that have never happened in this community, nobody have given neither taken money, we have been witnessing all process of the burial teams”.

M: Ok, the people will change their beliefs and behaviour after seeing or hearing this message?

R5: “Well, they are practising, because first, when someone dies, they carry out a lot of process, but now they not do, Ebola have really reduced a burden, no ceremony again, everybody is waiting to host a memorial service for their loved ones”.

M: So they have changed their belief and behaviour now?

R5: “Yes”.

M: Yes my sister?

R6: “Yes they will change their beliefs, after seeing this message”.

M: Why?

R6: “They have said let them don’t bribe, they will follow that”.

M: Ok, yes?

R7: “They will change after hearing this message, because they have told them not to give money, as the issue of money these days is difficult”.

M: Yes mama?

R8: “It is just the same thing”.

M: What is just the same thing?

R8: “They will change, because they have passed the laws and also by seeing or hearing this message”.

M: Yes my sister. Do you think, the people will change?

R1: “Yes, because they know bribing is a crime, either giving or taking”.

M: Yes my sister?

R2: they change, because it is the reduction of burden”.

M: What do you think is the best of t give this message out?

R1: “Let the government print more posters, so that it should be distributed in the community members”.

M: “Yes mama?

R2: “the message should be posted all over the community”.

R3: “This message should be given out through radio”.

M: When it is broadcast on radio, everyone will hear?

R3: “Yes”.

M: Ok, yes my sister?

R4: “I prefer radio, because not everybody knows how to read and write, but if it is aired in local in the different local languages, it will be fine”.

M: Yes my sister?

R5: “So villages do not understand much, unless they use the town crier to be announcing, and telling the people about this, so they will understand it better”.

M: Yes my sister, is there any way to give out this message?

R6: “Let them use loud speakers on cars, they will also post the poster on the car”.

M: Ok, yes mama?

R7: “It is not about the best way only, you have two stages, it should be dramatized in schools and communities clearly, they will understand and change, because they have seen through drama, that paper is just a picture, but once, someone have seen people dramatizing it, they will change”.

M: ok, you said two and you have given one, so what is the next stage?

R7: “This poster should be posted at the mosque, churches, junctions court Barry, hospital and community centres, it should be posted at these sensitive areas”.

M: Ok, yes my sister?

R8: “Well, the best way is house to house visit”.

M; Ok, who do you think, will give out this message?

R1: “Yes, the teachers, pastors and imams should give out this message”.

M: Why?

R1: “Because anytime they gather together, they give out this message, in schools, colleges, mosques and churches, like in the schools, pupils listen and adhere to what their teachers, say, even the mosque and churches, the worshippers listen to their religious head”.

M: Ok, yes mama?

R2: “One the section chiefs, they call meetings, and in this meetings, they give out the messages”.

M: Yes my sister?

R3: “The youths”.

M: Yes my sister?

R4: “Like how our colleagues have just said, the teachers, pastors and imams”.

M: Yes my sister?

R5: “It is just the same”.

M: We suggested the burial teams as messengers of this message, will you accept them?

R1: “No”.

M: Why?

R1: “Because whenever they come, they may just be concentrated at the place, where the corpse is, they are not good messengers to this message”.

M: Yes mama?

R2: “The burial teams are not good messengers to this message”.

M: Why?

R2: “I prefer the section chiefs, imams and pastors to the burial teams, because people are afraid of them also”.

M: Ok, yes my sister?

R3: “The burial teams are not good messengers to this message, I prefer the head men, because people are afraid of them. If it given to headman, the headman will give to contact tracers, and the contract tracers will give it the people”.

M: Yes mama?

R4: “The burial teams are not good messengers to this message”.

M: Why?

R4: One, they are not approachable, because when see someone and talk to the person, you may understand, but I am afraid of the person, how do I talk or listen to the person when you are dressed like masked dancer”.

M: Yes, my sister?

R5: “They are not good messengers to this message”.

M: Why?

R5: “Because the way they are dressed, I can’t see their face, so I cannot understand the message they may be passing on to me, so I prefer the town criers”.

M: So what about a member of your family?

R6: “Yes, if they trained and they come with the message, they will accepted, and they will listen to them”.

R7: “The family members are ok, once they have been trained”.

M: Yes mama?

R8: “You have said are our family members, so when they come, they are our family, we will accept them in with opened hands”.

M: What about a male and a female in this community?

R1: “Yes, we will accept them, as they are also part of the community”.

M: “ok”

R2: “Like how that woman just said, they trained me and my colleague as contact tracers, so we always do our work and the people accept and appreciate us, the community will accept a male and a female”.

M: Ok, yes my sister?

R3: “Yes, they are good messengers”.

M: Why?

R3: “Because they are from the community”.

M: Ok, yes my sister?

R4: “Yes, the community accepts their members in passing on this message”.

M: What about the radio jingle?

R5: “It will be fine, but not everybody have radio in the villages, so u prefer the town chief, he or she will distribute the posters?

M: “Ok, yes my sister?

R6: “The radio jingle are fine, but the loud speakers on cars are not fine, because not all villages have access road for vehicle”.

M: Yes mama

R7: “The radio is not good, I prefer someone on the motor bike, with loud speakers”.

M: Ok, yes my sister

R8: “The radio jingles are ok, because most of the house now have radio, so people will get the message from the radio”.

**(NOTE: Topic 13 – Early treatment and prevention – Jingle)**

M: Ok, let look at this other message but it is like a jingle” my name is Sallay, when I was sick, I did not take treatment at home, I went to the hospital. It is true my sister, that is the reasons the doctors always advice that when you are sick don’t stay home, go to the hospital, if you go to the hospital, your chance of survival increases, that is the message people, let work together to end this Ebola sick” what do you think of this message mama?

R1: “The message is fine for me and other people with radio”.

M: So what do you understand from this message?

R1: “The message is about a person that have survive from the Ebola sick, and they said home treatment is not good, the best solution is go to the hospital”.

M: Yes my sister?

R2: “Yes my sister, it is fine but in the villages not everyone has radio, if even they have radio, they don’t have money to buy battery, so they can able to hear the message?

M: Yes my sister?

R3: “It is just the same, the radio jingle is fine, but not everybody has radio and the message is trying to say, that when you are sick, go the hospital for early treatment”.

M: Yes my sister?

R4: “The radio jingle is good, but not everyone have radio to listen, that is the reasons when”.

M: This message is fine, it is good to go early to the hospital when you feel sick, when you feel sick, at the hospital they will diagnose the type of sickness”.

M: Yes my sister?

R5: “The message is good, and the message is trying to say that pepper doctoring is not, when you are sick go to the hospital”.

M: The people will accept this message?

R5: “Yes, they will accept to go the hospital, they will avoid pepper doctoring (home Treatment)”.

M: Why?

R5: “The message is out, that they should go to the hospital early to increase your rate of survival”.

M: Yes my sister?

R6: “Now they will accept, because they are seeing people going early to the hospital and survive, as I am sitting now, if I feel my head, I will go to the hospital”.

M: Yes my sister?

R7: They will accept this message”.

M: Why?

R7: “Because when the outbreak started, people were going to the hospital early, but will not survive, but now it is better, people are going early now and survive, so they will accept this message”.

M: Yes my sister?

R8: “Some people will accept and some will not, because some are still afraid to go to the hospital fearing that they should not come back, we will accept, because we know it is the best”.

M: The people in this community and the other communities will understand this message?

R1: “Well people will understand the Krio, instead of big English, some if even they don’t understand kKio, but their children will listen and explain in the language they understand, so there is nothing change, they will understand”.

M: Yes my sister

R2: “There is nothing to change, they will understand, the Krio is ok than English”.

M: Yes mama, is this message clear?

R3: “Yes it is clear, they understand the message better, and why people were afraid to go to the hospital, the spraying of the chlorine was really the problem”.

M: but do they spray now?

R4: “No, it is better now”.

M: “Ok, my sister is this message clear?

R5: “Yes, they will understand, even they can’t read or write”.

M: The people in this community and the other communities change their beliefs after hearing or seeing this message?

R6: “Yes, because Sallay has survived Ebola, so their beliefs will change and they will go to the hospital when they fall sick”.

M: Yes my sister?

R7: They will change their belief, because the knowledge about the sick now is improve, so they will accept this message”.

M: Yes my sister?

R8: Yes, they will change their beliefs and attitude, after hearing this message, because we have got a huge number of survivors now, and Sallay has also survived the Ebola sickness, so they will change”.

M: Ok, Who will the best person to disseminate this message?

R1: “The survivor, will be the best person to disseminate this message”.

M: Why?

R1: “The person has survived and has the experience, the survivor will share his or her experience with the people”.

M: Yes ma?

R2: “The same, the survivors, are the best people to disseminate this message”

M: Yes my sister?

R3: “The survivors”.

M: Yes my sister?

R4: “The survivors”.

M: Ok, what about the health workers, are they not good messengers to this message?

R1: “Yes, it is in place, they are good messengers”.

M: why?

R1: They operate the affairs of people’s health and also advice the people about Ebola”.

M: Ok, my sister?

R2: “The health workers are good messengers”.

M: Why?

R2: “They advise use to go to the hospital, let us don’t be afraid, like compare to beginning of the outbreak, because the people were afraid to go to the hospital”.

M: Ok, yes my sister?

R3: “Yes, they are good messengers of this message, because they always advise us to go to the hospital when we fall sick”.

M: Yes my sisters, how do you see it?

R4: The health worker are good messengers of this message, because they are in charge of people’s health”.

M: Ok, yes mama?

R5: “They are right people, because they are in-charge of health affairs in the country, so they are good messengers”.

M: Yes mama?

R6: “It is fine, they are good messengers but more of the nurses that work at the treatment centres, because they have vast knowledge in the treatment of Ebola virus disease”.

M: Ok, what about the chiefs, the pastors and imams, are they good messengers of this message?

R1: “Yes”.

M: Why?

R1: “Because they are stakeholders in the community, and people listen more to them”.

M: Ok, yes mama?

R2: “They are good messengers, we have one imam in this community that sensitized us about Ebola, every Friday, so the people will hear”.

M: Ok, are they good messengers?

R3: “Yes”.

M: What about the television programme, is it a good channel?

R1: “It is the best, because, you will see and hear, and some people believe in seeing then reading of hearing”.

M: ok, yes my sister?

R2: “It is fine, but many people do not have access to television, but it is good”.

R3: “As my sister has just said, the best way is, you help us come with the television, put it publicly, for everyone to watch, so people we see and understand”.

R4 “I prefer projectors, it is normally use in workshops, so if they come with it here, and placed it publicly, people will see,”

M: Yes my sister?

R5: “It is just the same”.

M: what is the same?

R5: “To come with television and put it publicly for us to see”.

**(NOTE: Topic 22 – Leaflet - “9 steps while you wait for the ambulance for pick up”)**

M: Look at these pictures, it is the nine steps, while waiting for the ambulance, what do you think of this message?

R1: “They said plastics, now we have new system now, I know one nurse that picks plastics every day, when they don’t have gloves, and they use it instead of waiting to get the gloves, so this plastic system, we just have to adapt to it until the end of Ebola, and even after Ebola we continue”.

M: Yes my sister?

R2: “They are advising us, after calling 117, if you don’t have plastic, wait for them to come, don’t touch”.

M: Yes my sister?

R2: “It is just the same thing, while waiting for the ambulance, you go about the following steps”.

R3: “It is fine, when someone dies to use plastic, because you don’t know the cause of the death, so it is better to use the plastic”.

M: Yes my sister?

R4: “When someone is sick while waiting for the ambulance, these are the steps, you should follow, while waiting, the message is fine”.

R5: “As my sister said, the message is fine”.

M: Yes my sister?

R6: “They are advising us that when someone is sick, you don’t have plastic, wait the ambulance team will come and arrange it”.

M: Yes Ma?

R7: The only thing about sick, when you are sick, just stay where you are and call 117, they will come and take you”.

M: yes my sister?

R8: “This message is trying to say, you have to isolate someone that is sick and call 117, use plastic to whilst attending to the person and later wash your hands, before and after removing plastic”.

M: The people in this community and other communities will accept this message?

R1: “Yes, they will accept to this message, and isolate the person, because if they don’t accept, the person will die also”.

M: Yes ma, do you think, people will accept this message?

R2: “Yes, because first, people were denying that we don’t have Ebola, but now, everybody is aware, so they will accept this message”.

M: Do you think, they will accept this message?

R3: “They will accept, because, they don’t want to die, when someone dies, use plastic to attend to that person”.

M: ok?

R4: “Some will accept and some will not accept”.

M: Why?

R4: “Because some will say, I will not leave my child to suffer, so am going to attend to her, either with plastic or no plastic”.

M: Yes ma?

R5: “This steps unless I practice, people will accept it, because we don’t know, Ebola is a sick that controls human being”.

M: ok, as we have seen the picture, these are the things to do while waiting 117, will the people understand this message, or is there any changes you think we have to do?

R6: “Yes, the person will understand, there is nothing to change”.

M: Yes ma?

R7: “Let the picture be bigger than this one”.

M: Yes?

R8: “Let the picture be brighter than this”.

M: ok, the people will change their beliefs, after seeing this message?

R1:”Yes, they will change”.

M: Yes my sister, will they change their beliefs?

R2: “Yes”.

M: Why?

R2: “The person knows that a lot of people have died from Ebola, so they will change and accept any message”.

M: Ok, yes my sister?

R3: “They will change, because the person don’t want to die, so you must follow this steps”.

M: Ok, my sister?

R4: “They will change, because the people don’t want to die, so they will change and accept this message”.

M: ok my sister?

R5: “They will must change and follow what this message is saying, because they don’t want to die,”

R6: “They will change, they will follow these steps, because they don’t want to die”.

M: Ok?

R7: “The people will accept this message and change their attitude and beliefs”.

M: What is the best way to give this message out?

R1: “Through jingle”.

M; Ok?

R2: “The Imam and pastors and section (chiefs)”.

M: ok?

R3: “Pastors, Imams and town crier”.

M: Ok, yes my sister?

R4: “Pastors, Imams, and through radio”.

M: Yes my sister?

R5: “If they make it bigger, it should posted at Town Barry when people go for meetings, they will see the message”.

M: Ok?

R6: “Let them enlarge the picture, they them posted at community centres and schools, even after Ebola, people will see, and the children dramatize it in schools”.

M: We were suggesting the court Barry meeting, is it a good one?

R1: “Yes, because people gather there, for meetings”.

M: Ok, yes my sister?

R2: “I prefer the court Barry, because when it is posted everyone will see”.

M: Yes my sister?

R3: “It is good, because everyone sees the message”.

M; Ok?

R4: “That was what I said right now, the section chiefs to spread this message, at the court barry”.

M: Ok. What to about the house to house?

R1: “It is fine”.

M: Why?

R1: “Because, when they go home, they will meet all the family members and deliver the message”.

M: OK, what about you?

R2: “The house to house talk is good, because we will meet, all the family members at home”.

M: Ok, yes my sister?

R3: “yes the house to house talk is good, because they have said let us avoid body contact, and when we crowed at the court barry, we touch each other”.

M: Ok?

R4: The house to house is good”.

R5: “Yes, the house to house talk is fine”.

M: Why?

R5: “Because you gather together in the evening at the house, then they will meet you home with the picture and explain the message to you”.

M: ok ma?

R6: “The house to house talk is fine, like I said, let’s train people in the community and they will do the house to house talk, they played a great role during the three days lockdown, sensitizing and distributing soap. The house to house needs trained people, so they will explain the message properly to the people”.

M: What about the message printed on plastic bags?

R7: “It will be fine, because the little person may want it as shopping bags, with that, they will get the message”.

M: Ok, yes ma?

R8: “the plastic bags are good, because most people use for marketing, they will see the message”.

M: Ok. Yes sister?

R1: It is fine, as I am not educated, but my child will read the message for me and explain to me”.

M: Who are the people to distribute this message?

R2: “For the plastics, the market woman should distribute it”.

M: Ok, yes my sister?

R3: “For house to house, the youths in the community”.

M: What about the radio jingle?

R4: “Some youths in this community also do radio jingle”.

M: yes ma?

R4: “The nurses should be used to give out this message”.

M: ok, yes my sister?

R5: “Like the radio jingle, let them use the journalist”.

M: yes mama?

R6: “The youths in the community, because they are very active”.

M: We suggested the MOHS and partners, are they good messengers?

R7: “Yes, because they know more about Ebola, so they will pass the message, be it contact tracers, nurses, they are good messengers”.

M: What about the chiefs, the pastors and imams?

R8: “They are good messengers”.

**(NOTE: Topic 29 – Chlorine – Poster - ”Me na yu paddi Mr. Chlorine, ar go hep yu fo fet Ebola” )**

M: let us look at the picture critically, what do you think of it?

R1: “This is chlorine”.

M: What the picture is saying exactly?

R1: “Telling us how to spray the chlorine, and in the container we have chlorine”.

M: What do you think of this picture?”

R2: “I think the man is spraying the chlorine to protect the other side”.

M: Ok, what do you think of this picture

R3: “I saw one big headed man standing”.

M: Ok?

R4: “I saw the man spraying and controlling the other foot for the chlorine not to reach it”.

M: Ok?

R5: “This is the work of Mr Chlorine and he is happy of the work”.

M: This picture is saying” I am your friend Mr Chlorine, I will help you fight Ebola, What do you think of this message?

R6: “It fight the virus”

M: Yes Mama?

R7: “Mr. Chlorine is my friend, he is helping me to fight Ebola”.

M: Yes my sister, what do you think of this message?

R8: “He is my friend, any place where I have vomit, Mr Chlorine my friend will come and spray there”.

M: Ok, Yes my sister?

R1: the man said, he is my friend, so when my family member or am is sick, he will help by spraying the chlorine”.

M: Will the people accept this message in the community?

R2: “No, because they are afraid of the chlorine, because they were thinking that the chlorine was the course of their death”.

M: Yes mama, what do you think of these message?

R3: “They will not accept, because the chlorine is bad, it does not have a friend”.

M: But the message is telling us that, we should not be afraid of Mr. Chlorine, he is friendly, and it is use by burial teams in the ambulance, what do you think?

R4: “Mr. Chlorine is not friend because it is not friendly”.

M: The people understand the picture?

R5: “They will understand from the picture, but they will not accept the message”

M: So is there any change to be made?

R6: “Yes, let them designed a person fully dressed in the protective gear instead of that man smiling”.

M: Yes my sister?

R7: “The need to change that drawing from the puppet to human being, so the people will understand”.

M: Yes my sister?

R8: “let the drawing resembles like the burial teams, as they are coming to bury, it should be drawn more bigger, so the people may understand”.

M: So they will not understand this picture?

R1: “Yes, let the diagram be a real human being”.

M: Yes my sister?

R2: They will not understand, they may think that, the man is watering, so let them change the picture to real human being dressed up like the burial team, so the people may understand”.

M; Ok, the people in this community and other communities will change their beliefs after seeing this message?

R3: “No, because in the picture, the man is laughing, so they will not understand and they will not change their beliefs”.

M: Ok?

R4: “Like the man in picture, he is laughing like we are not serious, it really can’t change the belief of the people”.

M: Ok. What is the best way to give this message out?

R5: “through radio, and television “.

M: Ok?

R6: “The town crier”.

M: Ok?

R7: “The burial teams”.

M: Ok?

R8: “The burial and the pastors”.

M: How will they distribute this message?

R1: “House to house and they give us the paper in our hands”.

M; who should do it?

R1: “The community people”

M: Ok, I thank you very much”.
