# Supplementary material for: Development of a set of community-informed Ebola messages for Sierra Leone
Source: PLoS Negl Trop Dis. 2017 Aug 7;11(8):e0005742. doi: 10.1371/journal.pntd.0005742 (PMC5560759; doi:10.1371/journal.pntd.0005742)
Supplement: S1 Appendix — (ZIP) [file pntd.0005742.s001.zip › Ebola messages - FGD and interview transcripts/R2HC Ebola Fieldwork 2/R2HC Ebola F2 FGD-Female-Rural2-D CORRECTED.doc]

| CODE | **R2HC Ebola F2 FGD-Female-Rural2-D (**Urban focus group discussion with younger (<25 years) and older (25+) females, using **topic guide GroupD and Picture set D)**  **7^th^ April 2015: corrected participant data** |
| --- | --- |
| DATE | March 2015 |
| DURATION (minutes) | 120 |
| Collector nrs | 2 and 5 |
| LANGUAGE INTERVIEW | Krio |

***PERSONAL DATA PARTICIPANTS***

| Nr | Sex  (*F/ M*) | Age  (*in years*) | Education Level (*e.g. none, Primary, secondary, tertiary*) | Language (*e.g. Mende, Temne, Krio)* | Religion | Job / Employment (*how they earn their living e.g. farmer, teacher, trader*) | Role in community  (*e.g. youth leader*)  ANONYMIZED, ONLY AREA OF ROLE INDICATED |
| --- | --- | --- | --- | --- | --- | --- | --- |
| 1 | F | 53 | Tertiary | Limba/Krio | Christian | TBA | none |
| 2 | F | 49 | None | Temne/Krio | Muslim | Farmer | none |
| 3 | F | 28 | Secondary | Limba/Krio | Muslim | Trader | none |
| 4 | F | 47 | Primary | Temne/Krio | Christian | Farmer | none |
| 5 | F | 25 | Secondary | Limba/Krio | Muslim | Student | none |
| 6 | F | 19 | Secondary | Limba/Krio | Muslim | Trader | none |
| 7 | F | 23 | None | Limba/Krio | Christian | Trader | none |
| 8 | F | 25 | None | Limba/Krio | Christian | Farmer | none |

**TRANSCRIPT: (M = Moderator, R= respondent, R1= first person responding to a question, DOES NOT correspond to numbering used in Personal Data!)**

**(NOTE: Topic 15/17 –Misconception healthcare system- ”Wea yu sick, nor fraid fo go hospital, den go hol yu fine” en “Wea yu sick, nor fraid fo go wit di ambulance, den go hol yu fine”**)

M: So we are going to start now, this message says, when you are sick, go to the hospital they will take care of you. Yes Ma, what do you think about this message?

R1: “I think this people have come to help us for this sickness”.

M: So is that what the message says? The message says, when you are sick do not be afraid to go to hospital they will take care of you. What do you think about this message? Yes Ma.

R2: “Is for good health”.

M: Yes, me other friend over there?

R3: “That you have to go the hospital to be cured”.

M: Yes Ma over there, what do you think?

R4: “Is about good health?

M: About good health en?

R4: “Yes, I know that when I am sick if I go to the hospital I can get medicine to cure me”.

M: Yes, what do you think about this message?

R5: “This message is fine, when you are sick you should go to the hospital, the message is fine”.

M: Yes Mummy?

R6: “For good health”.

M: Yes?

R7: “For us to get good health”

M: Yes?

R8: “When you are sick you have to go to hospital”.

M: What is this message about? Yes Ma, is there anything about this message that you do not like?

R1: “In this part, they have told everybody not to be afraid to go to hospital when they are sick, at first we used to be afraid to go to hospital”.

M: Yes my own friend, is there anything about this message that you do not like?

R2: “No, they have told us that we should not be afraid, that when we are sick let us go to hospital”.

M: Yes my own sister over there, is there anything about this message that you do not like?

R3: “Well, there is nothing about the message that I do not like”.

M: So you like everything about this message?

R3: “Yes”.

M: Yes my own sister over there?

R4: “Because when the sickness first broke out, when a person is sick they take that person to Kailahun (=first district in Sierra Leonw with Ebola cases) and the person will not come back, they will say the person is dead. But now, they have brought it closer to us when a person is sick they can take the person there and the person will come back. So, we are feeling happy now”.

M: So there is nothing wrong with the message that you not like? You like everything about the message?

R4: “No”.

M: Yes my own sister over there?

R5: “As for me I like the message because when the sickness broke out we were afraid to go to hospital because they said when a person is sick if the person goes there the person will not come back. So we were afraid to go to hospital but now we are not afraid again, we go there”.

M: Yes, my own pregnant woman?

R6: Like I said, there is nothing there that I do not like. Now when you go there you come back cured, you can get your medicines. There is nothing bad about it. They can go with you and come back with you. You eat free and come back”.

M: What is it about this message that you do not like?

R7: “We used to hear about this sickness far away, but now we believe that this sickness is real.

M: So if they bring this message into your community, would the people like it?

Rs: “YES”.

M: So like we are saying, this message is trying to tell us that when we are sick we should go to hospital we should not be afraid. Do you think people will understand this message if they come and tell them, would they understand this message?

Rs: “YES”.

M: What is it about this message that they people will not agree with?

R1: “There is nothing about it”.

M: Yes my sister over here, is there anything that they will not agree with?

R2: “There is nothing they will not agree with”.

M: Yes my sister is there anything they will not agree with?

R3: “No they will agree with everything”.

M: They will agree with everything?

R3: “Yes”.

M: So do you think if they hear this message, they change their perception about the Health Care System?

R3: “Well, just like we are seeing, they used to tell us about hand washing, they tell us that when we are sick we should go to hospital for cure. So as we are seeing it now, nobody is afraid to go to hospital, they are no longer keeping sick men, sick women at home, when a child’s body is warm they take the child to the hospital”.

M: So do you think this can make people change their beliefs?

R3: “Yes”.

M: Yes Ma, over there, do you think people will change their perception about this Ebola business when they get this message?

R4: “Yes”.

M: Is that all you have to say, I thought you were going to explain small?

R4: “Yes, because they have told us a lot about this Ebola business so people are changing now. We used to go to the dance and attending burials, but we no longer attend those things, so people are changing”.

M: So when they get this message they will change?

R4: “Yes”.

M: Yes my own pregnant woman, do you want to add something?

R5: “Now everybody has changed, because people are not afraid again”.

\

M: Yes, do you want to add something; will the people change their idea after getting this message?

R5: “Yes, they will change”.

M: Here, what about the other community?

R5: “All of them have changed because in the past when the sick broke out, when your child is sick they will be afraid to carry the child to hospital, but now when they see the paper, where ever they go with the child we will not be afraid again”.

M: Yes, you lactating mother over there, do you want to add something to this? Do you think if people hear this message they will change their belief about this Ebola business when they get this message?

R6: “Yes”.

M: Ok, like we are seeing this message, who do you think can carry this message?

R7: “Is us”.

M: You, who?

R7: “We who are sitting here”.

M: You can you take them to the other communities?

R7: “Yes”.

M: Yes Ma, you want to add something? Who do you think is the best person to carry this message?

R8: “When anybody leaves here and go to any other place, you have to tell them that people should not be afraid again, there is a hospital free for us. If they carry anybody there they will bring the person back so we should not be afraid. And that let all of us focus to the hospital”.

M: Yes, who do you think can carry that message?

R8: “Is us”.

M: You, who?

R8: “We whom you have come to tell”.

M: Are you the only people who should carry this message?

R8: “When we give them, they too can pass it over”.

M: Yes Ma, do you want to add anything, is there any other person you think can carry this message?

R1: “Is only some of us who can carry the message for our relative who are not here and those who are far inside the interior, so even if you cannot go there you will be able to send the message”.

M: That you can even call the person on the phone and pass the message?

R1: “Yes”.

M: Yes my own sister over there, do you want to add something? Who do you think can carry this message to other people?

R2: “Is only us who can share such message to our companions. We can tell our people that when someone is sick do not keep the person at home, take the person to the hospital, now there is no fear when you take the person to the hospital now everything has changed”.

M: Yes Madam over there, who do you think can carry this message?

R3: “Like in this community where we are now, if I am leaving here to go to the village if I meet any of my relative when going inside and meet those attending burials, we will tell them that we have been attending workshop and they are telling us that we should not touch, even if a child has head ache, you give the child panadol once and he doesn’t get better, carry that child to (--name of nearest town with a clinic, a CHC--) after (--same town --) you carry the child to (--name of Ebola treatment centre--) because the message comes from (--town with the CHC--) to carry the child to (-- name of Ebola treatment centre --)”.

M: Yes do you want to add something? Who do you think can carry this message?

R4: “Is only us who have to carry it”.

M: You, who?

R4: “Like us who are in the community. Like yesterday one of our relative died but when we went there they called the team but they did not come on time, the people waited until when they tem came around 5 O’ Clock and they went and buried, nobody did not touch it and then everybody left the place. Even if I meet my companion there I will tell her about it”.

M: Which best way can we use to carry this message?

R5: “By phone”.

M: By phone?

R5: “Yes”.

M: Is phone the best way to carry the message?

R5: “Now we know that when you are not closer to the person if you have phone when you call you can pass on the message”.

M: Yes Ma, do you want to add something? Which best way do you think we can use to carry this message?

R6: “Like for our relatives who are inside the villages, like for those ones here everybody has got the message, but for those inside, they are supposed to get the message”.

M: How can we carry this message?

R6: “You have to send somebody, if you are not able to send somebody; if you have a phone you can use it to call the person and tell the person about good health”.

M: Yes Madam over there?

R7: “As for me if I have my relative inside there and I hear such messages I can go and my relative that what is happening now we have to take our time and know how to move about”.

M: Yes Madam over there, do you want to add something?

R7: “Well as for me I have my small sisters, I can send them to call anyone for me, when she comes, I will tell her to take time with her husband and the children and the neighbours, that is what I will do”.

M: Yes my own lactating mother, how do you think we can carry this message to people? Which way is the best way for you?

R8: “Well, just like how we listen to you that is the way we are going to work with it”.

M: Is that the best way to send the message to people?

R8: “Like after this meeting here, when we go inside if I meet a sick person and they want to keep that person at home I will advise them to take the person to the hospital”.

M: Is like you are saying it has to be face to face to send the message?

R8: “Yes so I can tell them to call a meeting and tell the people that anyone who is sick they have to take the person to the hospital and they can be cured for free and that they will give them what they want and they can cure them and bring them back”.

M: Yes my own friend over there, which way do you think is the best way to send this message?

R1: “Is just the way as we hearing now, because there was fear but now there is no fear, when you meet your companion you have to tell her to go to hospital even if it is for head ache”

M: Yes Madam over here?

R2: “Well as for me, some who are in the nearby villages when they come to buy we tell them and then on Fridays the Imams will tell us in the Mosque and in the churches”.

M: Do you want to add something to that? Which best way do you think we can send this message?

R3: “Yes, well just like what my sisters are saying, we are here now, so when we enter inside those we meet will be able to explain to us like you are doing now”.

M: We had selected certain people to carry this message. We suggested giving the message to Traditional Healers, if we say letting Traditional Healer to carry the message, would you accept the person?

R4: “No”.

M: Why?

R4: “Because they said now no Traditional Healer should cure a person, they should not tell us message that is coming from a Traditional Healer, if it is coming from the hospital or you who are coming from there I will believe”.

M: So this message which says when you are sick go to the hospital they can take care of you, if a Traditional Healer comes and tell you, you will not believe?

R4: “No”.

M: What about you can you agree?

R5: “As for me I will agree”.

M: Why will you agree?

R5: “Because I want to get good health”.

M: So if he comes to tell you, you will agree?

R5: “Yes”.

M: Yes my sister, if a Traditional Healer comes to tell you about it will you agree?

R6: “Yes”.

M: Why will you agree?

R6: “Because they have told us that when you are sick you have to go to hospital. If they come and tell me to go to hospital I will go”.

M: Even if is the Traditional Healer you will agree?

R6: “Yes Madam over there?

R7: “Me, If I am sick, even if it is my child we first go to the hospital if they say there is no medicine for me then I can go the native way. But when I am sick and my child is sick, I first go to test if they ask me to go the native way then there is no problem”.

M: What if the Traditional Healer come and tell you to go to hospital won’t you agree?

R7: “No”.

M: What if we want to send them to come and tell you the message?

R7: “If it happens that I have been to the hospital and I am well I will tell my companions that I was sick but I am now well and then Ill pass that message to them”

M: But like this message now which says when you are sick do not be afraid go to hospital, if I give it to the Traditional Healer can you agree?

R7: “ No”.

M: Why won’t you agree?

R7: “The traditional Healer?

M: Yes.

R7: “Because he did not do it through test, I will not agree”.

M: But he will come to tell you that when you are sick you go to hospital?

R7: “I will agree”.

M: You will listen to him?

R7: “Yes”.

M: Yes my sister over there, will you agree if a Traditional Healer comes to tell you this message?

R8: “I will agree, as long as it the English way he brought it”.

M: Yes pregnant woman, if a Traditional Healer comes and tell you this message will you agree?

R1: “When he comes and tell me I just have to go because I know if I go I will be cured”.

M: So if we use the Mami Queen in this village, will you agree?

Rs: “YES”.

M: Why will you agree? Yes Mama? If the Mami Queen comes and tell you that if you are sick you go to the hospital, will you agree?

R2: “I will listen to her if she is coming from hospital and tells me that when I go there they can cure me”.

M: Yes Madam over here?

R3: “If I see that my companion does there and get cured, I will agree to go for me to be cured”.

M: So if the Mami comes to tell you will you agree?

R3: “Yes I will agree”.

M: Yes my sister over there, if comes and tell you that when you are sick go to hospital they will treat you fine, will you agree?

R4: “Yes”.

M: Yes Mama over there?

R5: “I will agree”.

M: Why will you agree?

R5: Because she has seen how they have cured people that is why if she comes to tell me I will agree”.

M: Yes my lactating mother over there?

R6: “As for me I will agree”.

M: Yes, you want to add something?

R7: “If the Mami Queen comes from the hospital and tell me to go there they will cure me, I will agree because they will treat me fine, anything I want they will give me so I will agree to the message”.

M: Yes my own pregnant woman?

R8: “I will agree because she is the Mami Queen”.

M: Why will you agree?

R8: “Because she comes from the hospital, what she sees there is what she will come to tell us, she cannot put us on a track”.

M: Ok, we also want to use a youth or musician in the village that we can use to send this same message. Will you agree?

R1: Yes we will accept it”.

M: Yes my Mama over here?

R2: “Yes hence they have attended the training when they come and tell me I will agree, that is why they went for the Workshop”.

M: Yes my friend over there, if we use musicians or the youth to send this message, will you agree?

R3: “Yes”.

M: Yes my friend over there?

R4: “Me as long as he is coming from the training and comes to tell me, I will agree, I will accept it”.

M: Yes my pregnant woman?

R5: “Whatever the Mami Queen comes and tells me I will take it, is the same work”.

M: Yes my friend?

R6: “As for me I will agree, when they go and tell her what she should come and tell us, I will take it”.

M: Then we also said we want to use Ebola survivors to come and send this message, will you agree?

R7: “Yes because he or she is now well, if he or she comes back to tell me I will accept”.

M: So if a survivor comes to tell you the message you will agree for the message?

Rs: “YES”.

M: Then we also think that the best way to send this message is by jingle, they play the message with music at the background on the radio, do you think that is the best way to send the message?

Rs: “YES”.

M: Yes Mama, why do you think that is the best way to send the message?

R1: “Because except someone who does not have radio, but anybody who has radio can get any information that is going round that is why we said that is the best way to send the message. Like some children are clever when they hear it on the radio they will get the understanding about this sick”.

M: Yes my sister, apart from the jingle which way is the best way to send the message?

R2: “Because when they play it, they can turn it over and over and explain to you how you should take care of yourselves and understand about that”.

M: Yes my sister.

R3: “Like me, I don’t have radio at home but I wake up by 6:30 when they tune the radio I will go and sit by it for me to hear it what they say about good health I will return and tell the person at home”.

M: So you do agree to the radio jingle?

R3: “Yes”.

M: Yes my sister over there, do you think that is the best way to send the message?

R4: “That one too is fine”.

M: When we use the radio?

R4: “Yes”.

M: Yes my own pregnant woman?

R5: “Even if I do not have radio in the morning I will get somebody who will tell me that this was what they said on the radio for this sickness”.

M: Yes My friend?

R6: “Radio is the best”.

M: What about if we use the Court Barry meetings to bring this same message, will you agree?

Rs: “YES”.

M: What about if we want to paste these pictures, where do you think we can post them?

R7: “Like here because the place is opening when you come you can see it”.

M: So you think this is the only place they can put it?

R7: “Yes”.

M: Where again do you think we can put it?

R7: “Like in the villages”.

M: Yes Mama, where do you think we put these messages?

R8: “They are supposed to be on each and every house in this village so that they can get the understanding about it”.

M: Yes my friend where do you think we can put these messages?

R1: “This they are supposed to be even in the Mosque and Churches, when a person enter there who knows how to read and write can understand what is written there”.

M: Yes the lactating mother over there?

R2: “Like here you can put it there, at the store you can also put it there, if a person cannot read and write when you meet someone reading you can ask the person to explain to you”.

M: Yes Madam? Where do you think they put these messages?

R3: “Like those who knows they can put one here and one down there, when they scatter it around, those who know can read it. Like for us we only know the pictures, if we see the picture we can understand them. But this one is not e everybody that knows it, only those who went to school can understand it”.

M: Yes my sister, where do you think we can put these messages?

R4: “If they put it here is fine, if they put it down there it is fine, when you meet someone who reads they can explain to you what is written there”.

M: Yes pregnant woman you want to add something?

R5: “On the doors, so when you see it you can know what they are talking about, it also fine to put it down the road on the first house so when you are entering you can see it”.

M: Is that the only place?

R5: “No”.

M: So like you have said, who do you think can be posting these messages?

R6: “Is you, when you reach any town they direct you”.

M: Are we the only people who can put it?

R6: “Those who you meet can help you; even if you give it to us we can share it for you”.

M: Who again apart from the ones you have called?

R7: “If you give it to me right now I will go and paste it”.

M: Then we also want to use vehicles with mounted speakers to send the message from village to village. Do you think that is a better way to send the message?

R8: “It is fine even if you are in the bush”.

M: Why do you think it is fine way?

R8: “Because the message is not for one person, it is for everybody that is why it is good”.

M: Yes Mama?

R1: “Even if you are in the farm you can get the message, when a child sees the vehicle he or she will come running to come and relay the message about good health”.

M: Yes my sister over there, do you want to add something?

R2: “No”.

M: So do you agree with the vehicle and the mounted speakers to pass the message?

R2: “Yes”.

M: So why do you think that is the best way?

R2: “Because If is only one person coming to talk to us it will be difficult, but if the vehicle comes everybody will hear it even if you are in the farm you can hear it”.

R3: “Like for me, what you are telling us when I leave here and go and tell my companions they will tell me to leave them alone, they will take it lightly but if the vehicle comes as long as it is making the noise, even if they are in the bush they will come to listen, the person who is not able to listen they can go and tell that person”.

M: Yes my lactating mother, do you agree with the vehicle with the loudspeaker?

R4: “Yes”.

M: So you think the loudspeaker vehicle is a fine way to pass the message?

Rs: “YES”.

**(NOTE: Topic 18 –Denial – “No matta di sick, call 117”)**

M: This message says, no matter the sick call 117. What do you think about this message?

R5: “When a person is sick you can call for them to come and carry the person”.

M: Yes, what do you think about this message? No matter the sick call 117

R6: “For them to come and take the person”.

M: The message says, no matter the sick call 117, what do you think about this message?

R7: “For them to come and take the person for treatment”.

M: Yes my sister over there, what do you think about this message?

R8: “They have told us that when someone is sick you should not touch the person, you call 117 for them to come and collect the person”.

M: No matter the sick call 117, yes my sister over there?

R1: “I am not well I can explain to someone to call 117 for them to come and take me”.

M: So even if you have head ache you should call 117, that is what you understand by this message?

R1: “Yes”.

M: Yes my other friend, what do you think about this message?

R2: “If I am feeling any part in my body, even if I do not have phone I can tell somebody to call 117 for me”.

M: Yes my sister over there? What do you think about this message? The message says no matter the sick call 117

R3: “They said when a person is sick you should not touch the person you should call 117”.

M: Yes my own sister?

R4: “That when you are sick you have to call 117 for them to come and take you”.

M: Yes my lactating mother?

R5: “Is just the way you feel your body, you cannot just take any kind of medicine, if you see that it is getting worst you have to call 117 to come and take you”.

M: So if you feel your body, you should call 117 for them to come and take you?

R5: “Yes”.

M: Ok, yes?

R6: “You should not wait until the sick overcome you, you have to call 117

M: Do you think the community will agree for this message?

R6: “Yes”.

M: Why do you think the community will accept this message?

R7: “Because now we are seeing it that when they call 117 they will come and take the person to cure him or her”.

M: Yes my lactating mother?

R8: “Just like what my sister said when you are sick you should call 117 for them to come and take you”.

M: Yes my sister?

R1: “It is a fine message because when a person is sick you have to call 117”.

M: Yes my lactating mother over there?

R2: “In the past when a person is sick they take him to the bush they were afraid, but now when you are sick they will carry you, when you are cured they bring you back. The vehicle that carries you is the same vehicle that will bring you back”.

M: You said they used to carry the person in into the bush, why do they take the person to the bush?

R3: “When the sick broke out newly they will take the person into the bush and tie herbs around the person and they will get well and come back to town but now they do not take the person to the bush, when you are sick they will take you directly”.

M: Who were the people tying the herbs?

R3: “Not that is any specific person, our ancestors when a person is sick they tie herbs around so even when your head ache they tie herbs around your head. At first I used to be afraid because they carried my uncle away and I did not see his body. But now when I have head ache I will go, more especially when I am a wood cutter, I will go”.

M: So do you think if they come with this message, will the people agree to it?

R3: “Yes”.

M: So do you think if people see this message they will change their idea about this Ebola business?

Rs: “Yes”.

M: What will they say?

R4: “At first when a person is sick and you call them, when they come they will come and take the person, so now anybody who has a sick person when they ask him or her to carry the person they do it. But now we have seen two, three, four five people go there and come back”.

M: So, can that change their idea about this sick? Yes my own sister there?

R5: “At first, just like what my sister said, they used to go with our brothers and when they die there we will not see their dead bodies, but now when they carry someone for treatment if he or she dies there they will bring his or her body to us for burial”.

M: Yes my own sister, do you think this message will change peoples’ idea about this Ebola business?

R6: “It has started changing people, if they carry you and you die they will come with you and bury fine they will pray for you and bury you. They even send to us to dig the grave for somebody who is dead, they will give you a befitting burial. So now we are changing”.

M: Yes Mama, do you think that if people see this message they will change their idea about this Ebola?

R6: “Yes, (*not clear, baby crying*) When she was feeling the pain when she went she said she was going to go, so when she went what she saw, they way she was treated, the things that she went with was burnt and they give her other ones and she came with them so I will know that there is nothing to fear”.

M: Yes my friend, do you want to add something to that?

R7: “At first when the sickness broke out, people were afraid to go to hospital because here they took two people away and we did not see them again, but the last time one old men died here, when he died they called them at night and they came in the morning and when they came they took the body outside and prayed for him and did everything for him and buried him fine. So when we have seen that when anyone is sick now we go to the hospital, we are no longer afraid because it is not like in the past when one dies they will see the person or see his or her grave but that is not happening again”.

M: Yes my pregnant woman, so do you think if people get this message it will change their ideas?

R8: “Why did you say so?

R8: “Is the way, in the past when somebody goes he or she will not come again but now when a person goes, he or she will come back, no one is afraid now because he or she have seen those who have returned so you too cannot be afraid”.

M: Yes my friend, do you want to add something to that?

R1: “No is just the same thing, now people are not afraid to go to hospital even if you die there they will come with you. But in the past when you die there they will not see you again, but now they will come with you so we are afraid again”.

M: Do you think if they get this message they will not be afraid again?

R1: “No”.

M: Yes my own sister over here?

R2: “Is just the same, now when a person is sick they can go with the person and cure the person and will come back so right now we can say there is a change about the sickness”.

M: So like this message, which method is the best way for this message to reach the people?

R3: “If you have a radio, if someone is not able to listen you can tell the person what they said”.

M: So you think that is the best way?

R3: “Yes”.

M: Yes Ma, so which way do you think is the best way to send this message?

R4: “The best way, just like you said, they put it on the mike and announce it. If there anybody in the bush when they hear the vehicle they will come”.

M: So that is the best way to send the message?

R4: “Yes”.

M: Yes Ma?

R5: “If a person is far away, like those who are Freetown or we who are here, someone can call Freetown to report what has happened here for people to take time how they move”.

M: So let them tell them by phone?

R5: “Yes”.

M: Yes, lactating mother over there do you want to say something? Which way do you think is the best way to send this message?

R6: “When I see my companions I will tell her is happening”.

M: So you are saying is by face to face that we have to send the message?

R6: “Yes”.

M: Yes do you want to add?

R7: “Like when we meet here at the barry when people gather whatever they are saying everybody will get it”.

M: So like Barry meeting is the best way to send the message?

R7: “Yes”.

M: Yes my own pregnant woman, which way do you think is the best way we can send this message?

R8: “If I meet my companion I will tell her what we heard in the meeting and that she should take time, when they come with soap and give it to you, use it to wash your hands fine, do not greet your companion, and do not touch”.

M: So, face to face is the best way to send this message?

R8: “Yes”.

M: Yes?

R1: “Through the radio”.

M: Through the radio?

R1: “Yes if my husband heard what they said he can tell me in the morning and I will in turn tell my mother and my children”.

M: Yes my sister?

R2: “Just like what we are saying if you meet your companion doing what is not right, you can tell her that it is not right to do it”

M: So that person to person is the best way to send the message?

R2: “Yes”.

M: Like we have said, who do you think can carry these messages? Like when you said the radio, who do you think can talk on the radio?

R2: “Is us”.

M: You mean all the people?

R2: “Those who understand about Ebola business”.

M: They are the ones to send the message?

R2: “Yes”.

M: Yes Mama, who do you think will send the message?

R3: “Except when they go around telling the message because we cannot walk around”.

M: Yes, is there anybody who wants to add something? Who do you think are the best people to send this message across?

R4: “Except those who talk on the radio”.

M: Who are the people that talk on the radio?

R4: “Like you, when you talk on the radio, you can say it for everybody to hear, in the morning someone will tell his or her companion”.

M: Is there any other person who can send the message?

R5: “Just like you said, the Mami Queen, and those who goes for training, they when they send the message people can believe the message”.

M: Ok, like for us, we said that we can use any woman in the community to send this message. Do you agree?

Rs: “YES”

R5: You are doing the work”.

M: We said any person who to person tell you so and so, will you agree for that person?

Rs: “YES”.

M: So you all agree that if we use a woman it would be fine?

Rs: “YES”.

M: Then we said we want to use musicians to send this message, do you also agree for the musicians?

R6: “Well, except if the person knows about Ebola business, when he talks we will agree”.

M: What if the person puts it into a song?

R7: “You cannot just come anywhere to come and talk on Ebola, I will not agree”.

R8: “When I am ready I go to my farm”.

M: Yes my own pregnant woman over there?

R1: “Well like for us now, I have come here and listen, when I get over there to my elder sister I will tell her what you have told me. But if happens that you have no training, you are not coming from (--name of the headquarter town of the interview district--), you just come from the bush and you go about playing music I will not listen you”.

R2: “They will say he is a drunkard”.

M: Yes Ma, you were saying something? Do you agree for the person who will send the message?

Rs: “Yes”.

**(NOTE it appears two topics were dealth with at one time – we are trying to check with audio and data collectors:**

**A.: Topic 19 – Health care system - worries – ”If you go hospital quick chance de say yu go well”)**

**B. Topic 28 – Stigma –Health care workers – “Nurse Susan na hero – E hep plenty Ebola survivor dem fo liv” en ”Di Ebola workman den na i champion den”)**

M: I have this picture, do you see it? Mama, what do you understand by this picture?

R3: “I don;t know that one”.

M: Buy do you understand from it even if you cannot write?

R3: “(not clear)

M: To talk to people?

R3: “Yes”.

M: Yes Mama, what do you think about this picture when you first saw it?

R4: “Is a message that they are giving that when you see it you know that is an Ebola message”.

M: Yes my mother over there, what first came to your mind when you see this picture?

R5: “What came to my mind is that as if the woman is coming from the hospital”.

M: Coming from the hospital?

R5: “Yes”. (*Respondents speaking Limba*)

M: What first came to your mind?

R6: “This is a woman who they carry and has been cured, and well dressed and they brought her back and also the man, this is the first man who they carry him as Ebola patient, he is well and he is back”.

M: So that is how you understand it?

R6: “Yes”.

M: Yes my pregnant woman, what came to your mind when you saw the picture this message?

R7: “Well as I saw the woman, they went and cure her and brought her back so now she is showing her result”.

M: Yes my sister, what do you understand by this message?

R7: “Is just the same thing, she is coming from the hospital for cure so that is the result”.

M: Yes my own pregnant woman?

R8: “Is the same thing just like what the others have said”.

M: We agreed that the Health Workers should send this message, do you agree to that?

Rs: “Yes, we agree”

M: Yes the Mami, do you agree to that if we use the Health Workers?

R1: “Yes”.

M: Why would you like that?

R1: “Because if they tell us everybody can understand it better”.

M: Mama, will you agree if you used the Health Workers to send this message?

R2: “Yes”.

M: Why will you agree?

R2: “If she comes here and tell how to do to get good health I will agree”.

M: What is it about this picture that you do not like?

R3: “At first we used to be in doubt, but now when you have enlightened us we have understood”.

M:Yes?

R4: “As for me, there is nothing there that I hate because the nurse has made those ones to survive”.

M: My own pregnant woman, do you want to say something?

R5: “There is nothing I hate there because the nurse has tried for those one to get well and we should like the nurse. When somebody cured you, you should like the person”.

M: Yes my own sister?

R6: “There is nothing there that I hate because the woman has cure those ones”.

M: Yes my sister, do you like this picture?

R7: “Yes I like it”.

M: Is there anything about it that you do not like?

R7: “No, because I have not seen a corpse lying there, I am seeing them alive”.

M: So do you think if we take this message to the community people will agree to this message?

R8: “Why would they agree to this message?

R1: “Because this has helped a lot of families”.

M: So they will agree to this message?

Rs: “YES”.

**(NOTE: Topic 32 – Fear of ambulance - “For trus di ambulance” en “Di ambulance na di best en safe way fo go hospital” en “Di healthcare wokman den frienly, den go hol yu fine” en “Fine breeze de blow insai di ambulance”)**

M: This last one says, to trust the Ambulance, to believe in the Ambulance, the Ambulance is the best and safe way to go to hospital, the Health Care Workers are friendly they will hold you fine, a fine breeze will blow you in the Ambulance. Mama, what is this message talking about?

R1: “At first we were afraid of the Ambulance because when the Ambulance is coming and starts to blow the siren everybody will get panic, so that was what we used to be afraid of”.

M: So are you still afraid of the Ambulance?

R2: “The Ambulance if it the sickness overcomes me I will walk and go”.

M: Yes my sister what do you understand by this?

‘

R3: “What I understand is that when a person is sick, the Ambulance will come and take the person”.

M: Question says you should trust and believe the Ambulance, the Ambulance is the best and safe way to go to hospital they will hold you fine and there is cool breeze in the Ambulance and the Health Workers are friendly they will take of you. So what do you think about this message?

R4: “At first when they come and take you here and carry you to (--name of town with closest PHU, a CHC--), from there to Kailahun, if you do not have long life you will die there”.

M: Why?

R4: “Because the kind of speed which they take some times they get accident”

M: Is accident still happening?

R4: “Well no, it a bit better now”.

M: It is better now?

R4: “Yes”.

M: So you now believe the Ambulance?

R: “Yes”.

M: Yes, do you want to add, the Ambulance?

R5: “I am not afraid of them again”

M: You are not afraid of them again?

R5: “No”.

M: Why were you afraid of them at first?

R5: “Because at first when they and take a person, they will close all the doors and windows of the Ambulance, no breeze will enter there, you will not be able to breathe again, and also the speed, that was why we used to be afraid of them”.

M: So who are the people to talk on the radio?

R6: “Those who had the training and know how to talk about Ebola”.

M: Yes Mama, you want to say something?

R7: “You who are working there”.

M: We who are working there to send this message?

R7: “Yes”.

M: So like we were saying we are going to use the Pastors and the Imams to end this message, do you agree to that?

R8: “Yes, once they have gone for the training”.

M: You will agree to them?

R8: “Yes”.

M: So do you agree for the Pastors and the Imams not so?

R1: “Yes, because if I do not know you to be a liar when you tell me I will believe”.

M: Yes Ma you stand, do you agree for the Pastors and the Imams?

R2: “Yes I will agree if I see the person goes for training”.

M: Yes my sister over there do you agree for the Pastors and the Imams?

R3: “Yes”.

M: Why do you agree?

R3: “Because if they go for the training, when we meet in the Church they can preach it to us”.

M: Then they also said the Youth Leaders; if we use them will you agree to that?

R4: “Yes, once they have gone for the training, if I meet him sitting ideally and he calls me I will not agree”.

M: Yes Ma, do you agree for us to use the Youth to pass on this message?

R5: “If he comes from the training and comes to tell me I will believe”.

M: Yes my mother over there?

R6: Like for us now we have one youth here when they call him to a workshop, when he comes back he will let them announce for us to gather, so when he talks to us we believe because he is coming from there. And I always see him go there, some tie s he comes with books”.

M: So you do believe?

R6: “Yes”.

M: What if we use women’s Leader or Chair Ladies?

R6: “We will believe because when they send for her she will tell us that she is going for a workshop, and she goes they will give her books to show to the people, so I will agree”.

M: Yes Mama, will you agree for the women or the Chair Ladies?

R7: “If she goes for the training I will agree”.

M: Why will you agree?

R7: “Because when she goes for the workshop, even if she does not know the paper but when she comes with the paper and announce in the evening, I will agree”.

M: Then we also said we will use the Court Barry meeting to pass the message, do you agree for that?

Rs: “YES”.

M: Yes Mama, why do you agree to that?

R8: “Because there is where everybody gathers”.

M: Do you that is the best way the message can reach the people?

RS: “YES”.

M: Yes the woman standing what do you think about the Court Barry meeting?

R1: As for us here when they announce here for meeting if you did not go there is five thousand and it is difficult to get five thousand so no sooner they announce everybody will go there”.

M: Yes my sister with the hand on the jaw? So is the Court Barry meeting fine?

R2: “Yes”.

M: Why is it fine?

R2: “Because we all meet there so anything they tell us everybody will hear”.

M: So do you also agree for the Court Barry meeting?

Rs: “Yes”.

M: Why do you agree?

R3: “Because is the place where we meet”.

M: Ok, I thank you very much.
